# Formation of spermatogonia and fertile oocytes in golden hamsters requires piRNAs

**DOI:** 10.1038/s41556-021-00746-2

**Published:** 2021-09-06

**Authors:** Zuzana Loubalova, Helena Fulka, Filip Horvat, Josef Pasulka, Radek Malik, Michiko Hirose, Atsuo Ogura, Petr Svoboda

**Affiliations:** 1 Institute of Molecular Genetics of the Czech Academy of Sciences, Prague, Czech Republic; 2 Bioinformatics Group, Division of Molecular Biology, Department of Biology, Faculty of Science, University of Zagreb, Zagreb, Croatia; 3 Bioresource Engineering Division, RIKEN BioResource Research Center, Ibaraki, Japan; 4 Bioresource Engineering Laboratory, RIKEN Cluster for Pioneering Research, Saitama, Japan; 5 Present Address: Institute of Experimental Medicine of the Czech Academy of Sciences, Prague, Czech Republic

**Keywords:** Piwi RNAs, Spermatogenesis, Oogenesis

## Abstract

PIWI-interacting RNAs (piRNAs) support the germline by suppressing retrotransposons. Studies of the pathway in mice have strongly shaped the view that mammalian piRNAs are essential for male but not for female fertility. Here, we report that the role of the piRNA pathway substantially differs in golden hamsters (*Mesocricetus auratus*), the piRNA pathway setup of which more closely resembles that of other mammals, including humans. The loss of the *Mov10l1* RNA helicase—an essential piRNA biogenesis factor—leads to striking phenotypes in both sexes. In contrast to mice, female *Mov10l1*^–/–^ hamsters are sterile because their oocytes do not sustain zygotic development. Furthermore, *Mov10l1*^–/–^ male hamsters have impaired establishment of spermatogonia accompanied by transcriptome dysregulation and an expression surge of a young retrotransposon subfamily. Our results show that the mammalian piRNA pathway has essential roles in both sexes and its adaptive nature allows it to manage emerging genomic threats and acquire new critical roles in the germline.

## Abstract (web summary)

A set of three papers reports that the piRNA pathway is essential for mammalian female fertility based on genetic perturbation experiments performed in golden hamsters.

## Main

The piRNA pathway is a key germline-specific silencing mechanism that is crucial for defending the integrity of the genome against transposable elements (reviewed previously^1,2^). Mammalian primary piRNAs originate from specific loci (piRNA clusters) as long precursor transcripts that interact with the essential and conserved helicase MOV10L1, which feeds precursor transcripts into the piRNA biogenesis mechanism^3–5^. Mammalian piRNAs fall into the following four categories: (1) 26–28-nucleotide retrotransposon-derived piRNAs produced mainly in fetal testes; (2) 26–27-nucleotide postnatal piRNAs from non-repetitive sequences including the 3′ ends of mRNAs; (3) 29–30-nucleotide mostly non-repetitive highly abundant pachytene piRNAs produced from ~100 loci in spermatocytes and spermatids; and (4) oocyte-specific 19–20-nucleotide piRNAs that are enriched in antisense sequences of recently evolved transposable elements^6–11^. Mature piRNAs associate with the PIWI subfamily of Argonaute proteins^12^. Mice (*Mus musculus*), the leading mammalian model for the piRNA pathway, use three PIWI proteins—PIWIL1, PIWIL2 and PIWIL4 (also known as MIWI, MILI and MIWI2, respectively). Loss of MOV10L1 or PIWI proteins in mice revealed specific essential roles of piRNAs in spermatogenesis but not in oogenesis^3,4,13–16^. However, it is unclear whether the dispensability of piRNAs in females is common among mammals. piRNAs target retrotransposons during mouse oogenesis^17,18^, but the maternal piRNA pathway is partially redundant with RNA interference (RNAi)^19^. High endogenous RNAi activity specifically evolved in mouse oocytes^20^ and seems to be absent in bovine and human oocytes^10,21^. Furthermore, mice lack PIWIL3, which binds to 19–20-nucleotide piRNAs in human and golden hamster oocytes and exists in many mammals, suggesting that there is a major difference between oocyte piRNA biology in mice versus other mammals^10,11,21^. However, it is unclear whether differences in the piRNA pathway set-up are associated with biologically important roles.

To delineate the conserved and derived aspects of the mammalian piRNA pathway, we selected the golden hamster (*Mesocricetus auratus*) as an optimal experimentally tractable comparative model that is amenable to genetic manipulations^22^. Despite ~24 million years of independent evolution^23^, hamsters share many anatomical and physiological features with mice, including fast zygotic genome activation, short gestation and a large litter size^24,25^. At the same time, the hamster outclassed the mouse model for specific aspects of human biology^25,26^. Crucially, in contrast to mice and similar to humans, golden hamster retained four PIWI paralogues expressed in the germline and its oocytes probably lack highly active RNAi^27^.

## Results

### Hamster piRNAs and retrotransposons

**Fig. 1:**
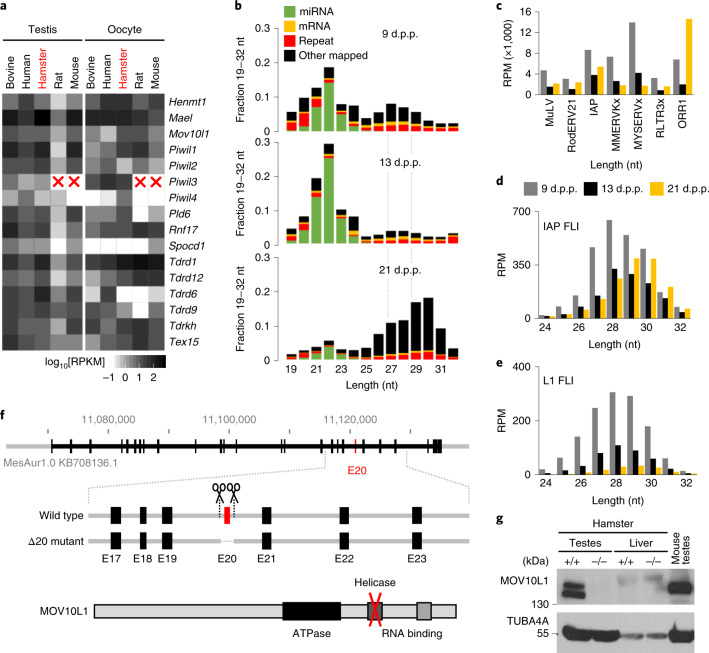
Golden hamster piRNA pathway and the *Mov10l1* knockout. **a**, The expression of piRNA pathway factors in the testes and oocytes of five mammals. Mouse and rat lack *Piwil3*. RPKM, reads per kilobase of transcript per million mapped reads. **b**, The distribution of 19–32-nucleotide-long RNAs from testes at 9, 13 and 21 d.p.p. nt, nucleotide. **c**, Testicular 24–32-nucleotide small RNAs mapping to LTR retrotransposon groups selected for low nucleotide exchange rate and high abundance of putative piRNAs (Extended Data Fig. 2a). The *y* axis displays reads per million (RPM) of 19–32-nucleotide RNAs. **d**,**e**, The distribution of 24–32-nucleotide antisense RNAs from testes at 9, 13 and 21 d.p.p. that perfectly map to FLI IAP (**d**) or L1 (**e**) insertions, respectively. The *y* axis displays the RPM of 19–32-nucleotide sequence reads. For **b**–**e**, values were calculated as the mean values of two biological replicates of wild-type testis samples (Supplementary Table 10). **f**, MOV10L1 protein organization and the knockout strategy. The CRISPR–Cas9 cleavage positions (scissors) flanking exon 20 (red rectangle; E20) are shown. **g**, Western blot analysis showing the absence of MOV10L1 in mutant adult testes. Liver and mouse testes were used as negative and positive controls, respectively, for antibody specificity. This experiment was performed once; the impact of the mutation on *Mov10l1* expression in the testes was confirmed using RNA-seq analysis (Extended Data Fig. 3c). Source data

To develop the golden hamster into a model for the piRNA pathway, we first mapped the expression of the piRNA pathway components and the properties of golden hamster piRNAs (Fig. 1a–e). We focused on testicular piRNAs as there was plenty of comparative data from other mammals, particularly from extensively studied mice. Ovarian hamster piRNAs have recently been investigated^11^.

**Extended Data Fig. 1:**
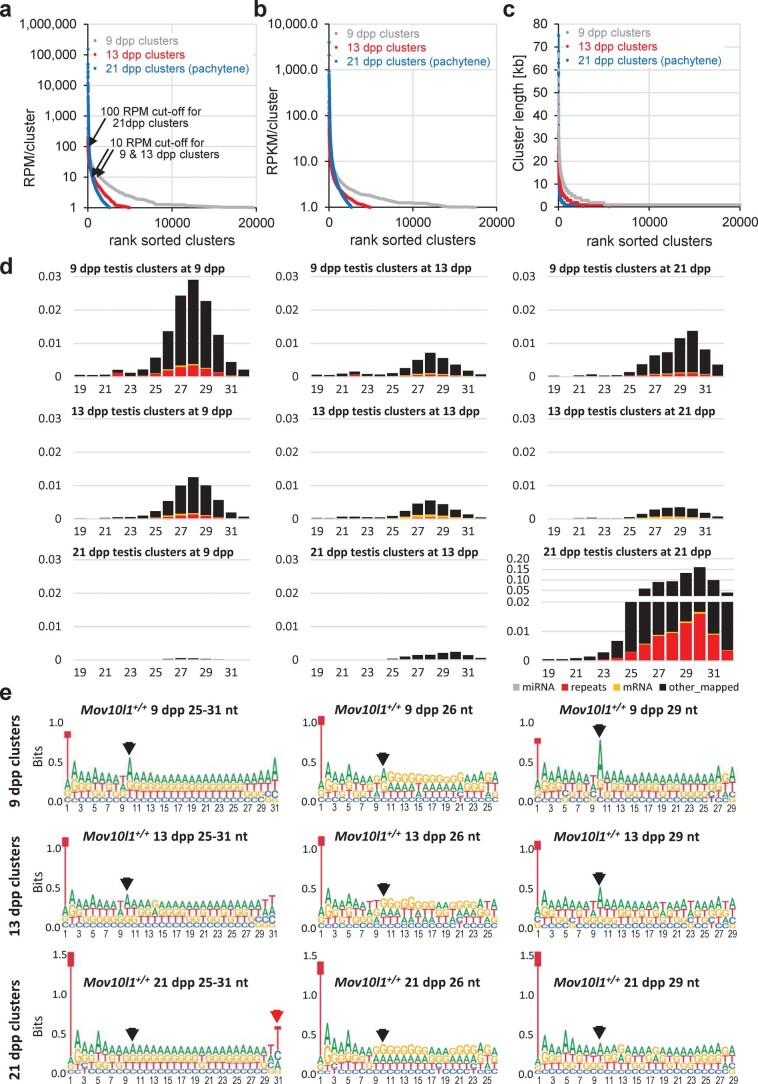
Analysis of golden hamster’s testicular piRNA clusters. Analysis of golden hamster’s testicular piRNA clusters. (a) Rank-sorted annotated pre-pachytene and pachytene clusters according to the amount of small RNAs per cluster (RPM). (b) Distribution of piRNA clusters according to average piRNA density (cluster per RPKM). (c) Distribution of piRNA cluster sizes. (d) Graphs depict frequencies of 19-32 nucleotides-long RNAs mapping to annotated pre-pachytene (9 d.p.p., 13 d.p.p.) and pachytene (21 d.p.p.) clusters (Supplementary Tables 1-3) at the three studied time points. Coloring of bars indicates proportions of miRNAs and small RNAs derived from repetitive sequences, mRNA, and other sequences. (e) Sequence logos for piRNA clusters from 9, 13, and 21 d.p.p. testes (Supplementary Tables 1-3). U at position 1 is a common piRNA feature^2^, preference of A at the position 10 (black arrow) in the sequence logos of 29 nt long piRNA clusters from 9 and 13 d.p.p. testes is a “ping-pong” signature of secondary piRNAs^2^. 29 nt long piRNAs from 21 d.p.p. and shorter piRNA clusters from all three time points show little if any signs of the “ping-pong” signature. The red arrow points to apparent U-tailing of pachytene piRNAs. Three biological replicates of wild type 9 d.p.p. testes and two biological replicates of wild type 13 d.p.p. and 21 d.p.p. testes were used for small RNA analysis presented in this figure. Source data

We examined testicular piRNAs at 9 days postpartum (d.p.p.) when spermatogonia form; at 13 d.p.p. when testes contain spermatogonia but not meiotic spermatocytes; and at 21 d.p.p. when meiosis in primary spermatocytes reaches the pachytene stage^28^ (Extended Data Fig. 1a–c and Supplementary Tables 1–3). Pre-pachytene piRNAs were broadly dispersed at a relatively low density in intergenic and genic regions, while most pachytene piRNAs mapped to ~100 loci, many of which were syntenic with mouse, cow and human testicular piRNA loci. Unique and repetitive 27–29-nucleotide pre-pachytene piRNAs and highly abundant non-repetitive 29–30-nucleotide pachytene piRNAs had typical mammalian piRNA features, including the presence of uridine at the 5′ end (Fig. 1b and Extended Data Fig. 1d,e). Notably, sequence analysis of 29-nucleotide pre-pachytene piRNAs revealed an increased frequency of adenosine at nucleotide 10 (Extended Data Fig. 1e), which is a signature of the ‘ping pong’ mechanism that generates secondary piRNAs^2^. Together, hamster postnatal testicular piRNAs shared features with those of other mammals.

**Extended Data Fig. 2:**
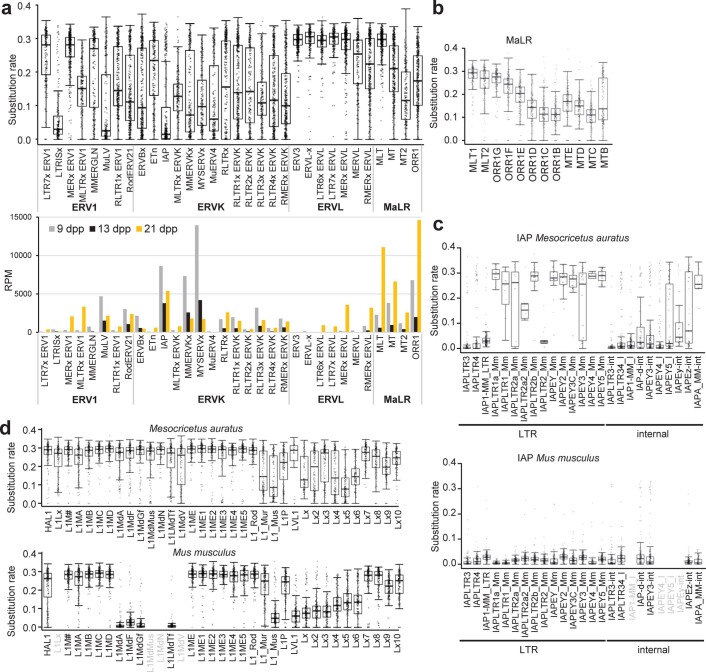
Nucleotide substitution rates of retrotransposons sequences. Nucleotide substitution rates of retrotransposons sequences. (a) Boxplots show nucleotide substitution rates in selected LTR subgroups and abundance of 24-31 nt RNAs carrying depicted retrotransposon sequences. The lower graph depicts RPMs for 24-31 nt small RNAs per million of 19-32 nt reads from wild type 9 d.p.p., 13 d.p.p. and 21 d.p.p. whole testes small RNA sequencing. Sequencing was performed in three biological replicates from 9 d.p.p. and two biological replicates from 13 d.p.p. and 21 d.p.p. testes. (b) Nucleotide substitution rates in MaLR elements. (c, d) Nucleotide substitution rates in IAP and L1 subfamilies in golden hamster and mouse. Young IAP and L1 subfamilies, which supply most FLI elements show minimal nucleotide substitution rates. All boxplots were constructed from 200 randomly selected insertion sequences (or all insertions if there was less than 200 of them). The internal line in the boxplot represents median and bounds of box the 1^st^ and the 3^rd^ quartile; whiskers extends to values no lower (minima) or higher (maxima) than 1.5 * IQR (interquartile range). Source data

As piRNAs provide an adaptive defence against transposable elements, we investigated which golden hamster retrotransposons are the main targets of the piRNA pathway. Using an improved golden hamster genome assembly^11^, we determined the entire complement of hamster retrotransposons, identified potentially active retrotransposon subfamilies and estimated the abundances of retrotransposon-derived piRNAs. An analysis of mutation rates of long terminal repeat (LTR) retrotransposons in hamster and mouse genomes revealed divergent evolutionary paths of specific subfamilies (Supplementary Text, Extended Data Fig. 2 and Supplementary Data 1–3). We observed that the ERVK class, exemplified by rodent-specific intracisternal A particle (IAP) and MYSERV retrotransposons, expanded during the evolution of the hamster (Supplementary Data 1 and Extended Data Fig. 2a). Notably, MYSERV and IAP-derived piRNAs were also the most abundant piRNAs targeting autonomous transposable elements (Fig. 1c and Extended Data Fig. 2a). In contrast to MYSERV retrotransposons, IAPs are well characterized transposable elements that evolved from a retrovirus in the common ancestor of hamsters and mice^29,30^. Thousands IAP insertions can be identified in mouse and hamster genomes, but only a small fraction are full-length intact (FLI, hereafter denoted as intact) insertions, possibly supporting retrotransposition. In the golden hamster genome, we identified 110 intact IAP insertions, classified as the IAPLTR3/4 subgroup (Supplementary Data 4), whereas a different subgroup (IAPE) is mobile in mice^31^ (Extended Data Fig. 2c). Interestingly, piRNAs antisense to intact IAPs were abundant at all three of the tested time points (Fig. 1d). In contrast to the ERVK class, there was no notable recent expansion among hamster’s autonomous elements from the ERVL class (Supplementary Data 2 and Extended Data Fig. 2a); this class underwent substantial expansion in mice with a major impact on gene expression in oocytes and zygotes^27^.

The most relevant non-LTR retrotransposon is the long interspersed nuclear element L1, the most successful autonomous transposable element invading mammalian genomes^32^. L1 analysis revealed 110 intact L1 elements from the Lx5/6 subfamily (Supplementary Data 5), which is comparable to the 146 intact L1 elements in the human genome but much smaller than the 2,811 intact L1 elements in the mouse genome^33^, which come from a different L1 subfamily (Extended Data Fig. 2d). Analysis of piRNA sequences suggested that hamster intact L1s are most targeted by pre-pachytene antisense piRNAs (Fig. 1e).

### Sterile phenotype of *Mov10l1*^–/–^ females

**Extended Data Fig. 3:**
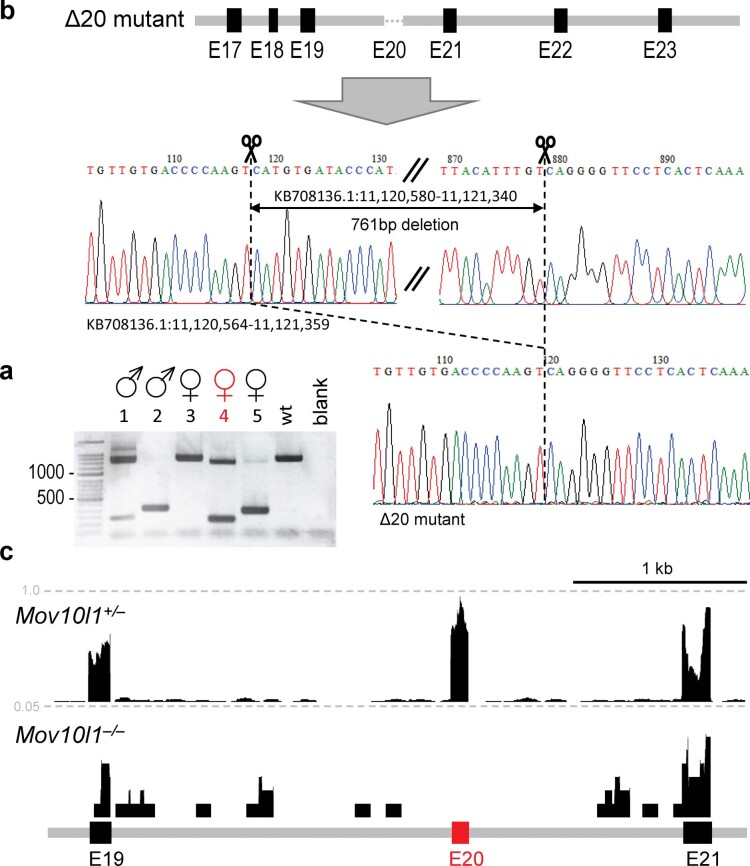
Mov10l1^–/–^ hamster model production. *Mov10l1*^*–/–*^ hamster model production. (a) PCR genotyping of five F0 animals. The upper band >1 kb corresponds to the wild-type allele. 4/5 animals carried at least one mutated allele. Female #4 was heterozygous and transmitted the mutated allele into F1. (b) Schematic position of CRISPR-Cas9 cleavage sites and validation of the deletion induced in female #4 by Sanger sequencing. (c) A UCSC browser snapshot showing absence of *Mov10l1* sequences mapping to the removed exon 20 in RNA-sequencing data from 9 d.p.p. testes. Dashed lines depict counts per million (CPM) of normalized (per library size) expression data. Two biological replicates of 9 d.p.p. testes with each genotype were analyzed by RNA-seq, displayed tracks were constructed from merged RNA-seq data from each genotype. Source data

To examine how the biological significance of the hamster piRNA pathway compares with that of the mouse, we knocked-out *Mov10l1* by deleting exon 20, which encodes the helicase domain (Fig. 1f and Extended Data Fig. 3), generating a deletion analogous to one studied in mice^3^. Western blot analysis showed the lack of MOV10L1 in *Mov10l1*^–/–^ testes (Fig. 1g). Heterozygotes were fertile and segregation of genotypes did not deviate significantly from the Mendelian ratio but homozygotes of both sexes were sterile (Supplementary Table 4). Male *Mov10l1*^–/–^ sterility could be expected, but female *Mov10l1*^–/–^ sterility was surprising given the normal fertility of female *Mov10l1*^–/–^ mice^3,4^.

**Fig. 2:**
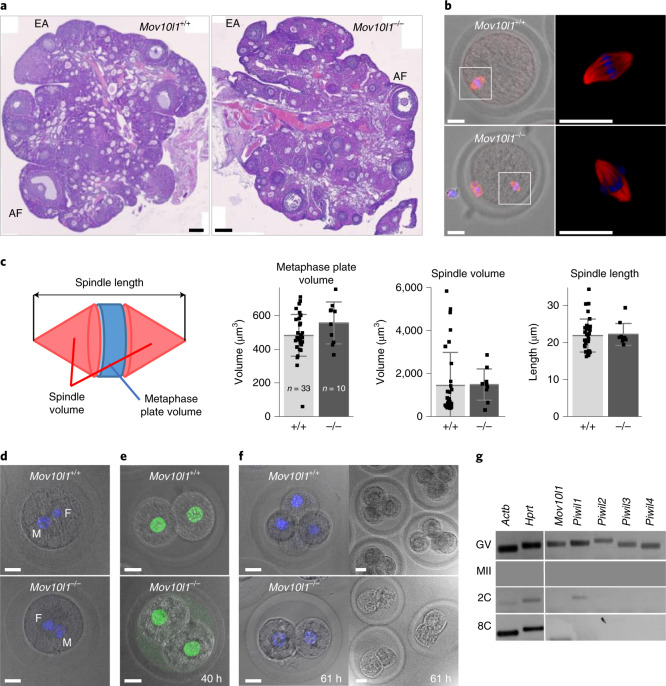
Female *Mov10l1*^–/–^ phenotype. **a**, Haematoxylin and eosin (H&E) staining of ovarian sections. Antral follicles (AF) and early antral follicles (EA) indicate normal follicular development. The ovaries of four *Mov10l1*^–/–^ and two *Mov10l1*^+/+^ female hamsters were analysed and representative images are shown. Scale bars, 200 μm. **b**,**c**, Mutant oocytes mature to the MII stage with a normal spindle 17 h after injection of human chorionic gonadotropin (hCG) (red, tubulin; blue, DNA (4,6-diamidino-2-phenylindole (DAPI))). Images of spindles are shown (**b**) and quantitatively analysed (**c**). **c**, Quantitative analysis of MII spindle traits. The spindle length, spindle volume (tubulin staining signal) and metaphase plate volume (DAPI signal) of MII eggs (isolated 17 h after hCG injection) were quantified using three-dimensional spindle reconstruction from confocal optical sections. Data are mean ± s.d. from 33 *Mov10l1*^+/+^ and 10 *Mov10l1*^–/–^ eggs. **d**, *Mov10l1*^–/–^ eggs can be fertilized and form one-cell zygotes. Bright-field confocal images are overlaid with DNA staining by DAPI. Larger male (M) and a smaller female (F) pronuclei are shown. **e**, *Mov10l1*^–/–^ eggs give rise to two-cell zygotes 40 h after mating. Bright-field confocal images are overlaid with H3K9me3 staining (green) showing that two-cell zygotes from *Mov10l1*^–/–^ eggs have no major heterochromatin defect. **f**, Fertilized *Mov10l1*^–/–^ eggs do not develop beyond two-cell zygotes. Zygotes were isolated 61 h after mating. Representative bright-field confocal images overlaid with DNA staining by DAPI are shown. Mating and zygote isolation at 40 h or 61 h after mating was performed twice with the same results. For **b** and **d**–**f**, scale bars, 20 μm. **g**, RT–PCR analysis of transcripts of piRNA pathway genes in oocytes and zygotes. This analysis was replicated four times for germinal vesicle-intact (GV) oocytes, twice for MII and three times for two-cell (2C) stages. Analysis of the eight-cell stage was performed once. Source data

We first determined the basis of *Mov10l1*^–/–^ female sterility. *Mov10l1*^–/–^ ovaries appeared to be histologically normal, suggesting that mutant oocytes enter the first meiotic block and develop to preovulatory oocytes in the antral follicles (Fig. 2a). *Mov10l1*^–/–^ oocytes ovulated and matured in vivo into apparently normal metaphase II (MII) oocytes (Fig. 2b,c). However, zygotes from *Mov10l1*^–/–^ females did not develop beyond the two-cell stage (Fig. 2d-f), a stage during which the major zygotic activation occurs^24^. As the breeding of heterozygotes yielded a Mendelian frequency of *Mov10l1*^–/–^ progeny (Supplementary Table 4), this implied a maternal effect phenotype whereby *Mov10l1*^–/–^ oocytes retain meiotic competence but lack the ability to support development irrespective of the genotype of the zygote.

**Fig. 3:**
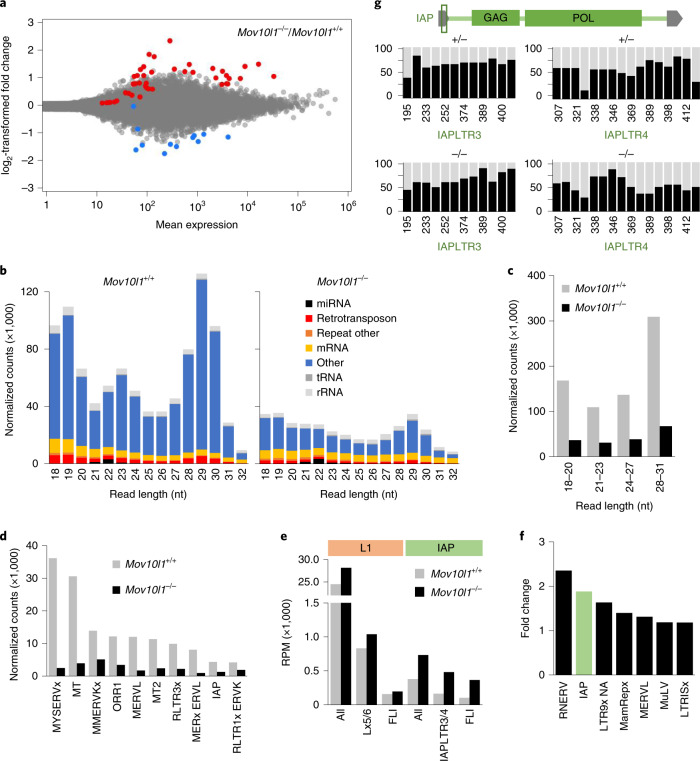
Transcriptome changes in *Mov10l1*^–/–^ oocytes. **a**, MA plot of differentially expressed protein-coding genes (DESeq2, *P* < 0.01). The red and blue points depict genes of which the transcripts were present at significantly higher or lower levels in fully-grown *Mov10l1*^–/–^ oocytes, respectively. The full DEG list is provided in Supplementary Table 5. **b**, Composition of an 18–32-nucleotide segment of RNA-seq libraries from fully grown *Mov10l1*^+/+^ and *Mov10l1*^–/–^ oocytes. The abundance of small RNAs in the wild-type control corresponds to the RPM of 18–32-nucleotide reads (average value from two libraries). The *Mov10l1*^–/–^ library was normalized to the amount of maternal miRNAs. **c**, Reduced levels of different classes of piRNAs in *Mov10l1*^–/–^ oocytes. The abundance of reads of different sizes mapping to annotated oocyte piRNA clusters (Supplementary Table 6) is shown. *Mov10l1*^–/–^ values were scaled by miRNA abundance. Read sizes were divided into categories to separate putative PIWIL3-bound piRNAs (18–20 nucleotides), Dicer products (21–23 nucleotides), and smaller and longer piRNAs (24–27 nucleotides and 28–31 nucleotides). **d**, Reduction of LTR retrotransposon-derived piRNAs in *Mov10l1*^–/–^ oocytes*.* **e**, Changes in RNAs from L1 and IAP families and subfamilies. The RPMs of RNAs mapping to L1 or IAP elements (all), active subfamilies and FLI only are shown. Data are the mean values of two (*Mov10l1*^+/+^) and three (*Mov10l1*^–/–^) biological replicates. **f**, LTR retrotransposon groups ranked by the highest transcript upregulation in *Mov10l1*^–/–^ oocytes. Data are the mean values from two (*Mov10l1*^+/+^) and three (*Mov10l1*^–/–^) replicates. **g**, DNA methylation of intact IAPs. The vertical bars represent methylation (black portion) observed for the indicated 5′ CpG dinucleotides covered by at least ten sequence reads; the analysed region corresponds to the central and 3′ part of the 5′ LTR, as indicated in the IAP scheme and by CpG position. Data are from a single genome-wide bisulfite sequencing experiment. Source data

**Extended Data Fig. 4:**
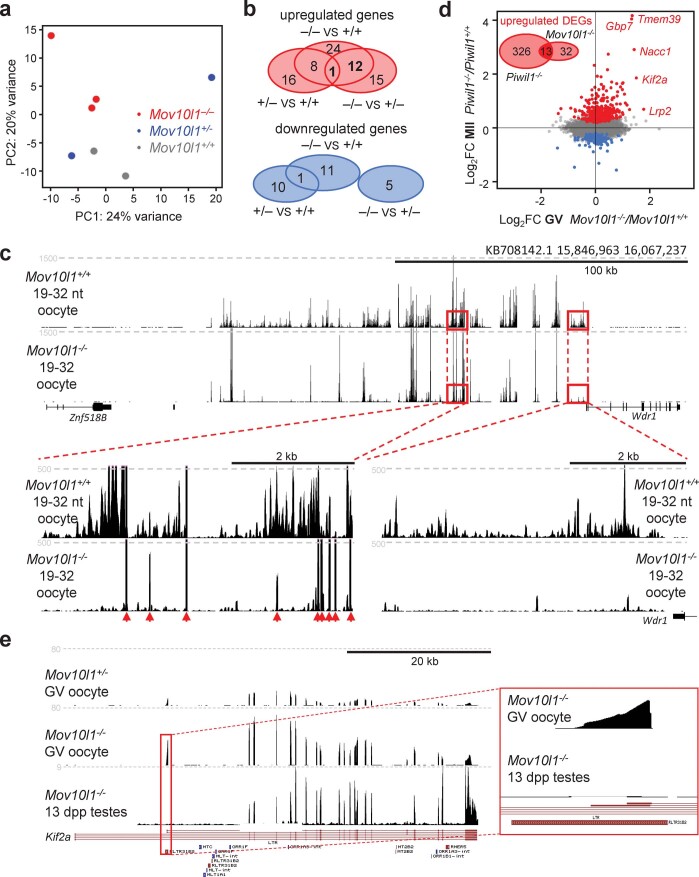
Transcriptome changes in Mov10l1^–/–^ oocytes. Transcriptome changes in *Mov10l1*^*–/–*^ oocytes. (a) Principal Component Analysis (PCA) depicting distribution of individual RNA sequencing libraries (produced as biological replicates) used for transcriptome analysis. (b) Venn diagrams show overlaps among transcripts with increased or decreased relative abundance when comparing different *Mov10l1* genotypes. (c) Snapshots from the UCSC genome browser depicting a female-specific piRNA cluster localized between *Wdr1* and *Znf518Bb* described previously^11^. While the majority of piRNAs along the cluster become strongly reduced in *Mov10l1*^*–/–*^ oocytes, some specific piRNAs (red arrows) remain present. This contrasts with piRNA changes in male piRNA clusters at 9 d.p.p. (compare with Fig. 6c and Extended Data Fig. 7c). The *Mov10l1*^*+/+*^ track is a “merged” track from two independent RNA-seq libraries, *Mov10l1*^*–/–*^ track is derived from a single RNA-seq library. (d) Comparison of relative changes in *Mov10l1*^*–/–*^ fully-grown oocytes and ovulated *Piwil1*^*–/–*^ MII oocytes^34^. *Piwil1* analysis was done in triplicates, *Mov10l1* libraries are the same as in the panel a). Colored are genes differentially expressed in *Piwil1*^*–/–*^ MII oocytes (DESeq2 p-value <0.01). Clustering of red points (significantly upregulated genes) in the upper right quadrant implies a common pool of upregulated genes. The Venn diagram shows overlap of upregulated DEGs (a single gene was common for downregulated DEGs). (e) A UCSC genome browser snapshot of RNA-seq data and genomic structure of *Kif2a* locus with an RLTR31B2 LTR-derived oocyte-specific promoter. The *Mov10l1*^*–/–*^ track is a merged track from three independent RNA-seq libraries, the remaining two tracks were constructed from two independent RNA-seq libraries.

As *Mov10l1* and *Piwil* genes are expressed maternally (Fig. 2g), we hypothesized that transcriptomes of fully grown oocytes, the final stage of ovarian oogenesis, might exhibit early signs of the loss of developmental competence. We identified 57 differentially expressed genes (DEGs) in *Mov10l1*^–/–^ oocytes (Fig. 3a, Extended Data Fig. 4a,b and Supplementary Table 5). By contrast, 1,612 DEGs were reported in *Piwil1*^–/–^ ovulated MII hamster oocytes, which also fail to support zygotic development^34^. However, maternal transcriptome remodelling may differ because piRNA populations in *Mov10l1*^–/–^ and *Piwil1*^–/–^ oocytes are affected differently. *Mov10l1*^–/–^ oocytes have a massive but incomplete loss of piRNAs (Fig. 3b–d) whereby the production of specific abundant maternal piRNAs remains even in the absence of MOV10L1 (Extended Data Fig. 4c). Furthermore, we analysed pre-ovulatory oocytes while *Piwil1*^–/–^ analysis concerned a later MII stage^35^ at which meiotic transcriptome remodelling^36^ could enhance transcriptome changes that are considered to be statistically not significant in fully grown oocytes. A direct comparison of *Mov10l1*^–/–^ and *Piwil1*^–/–^ data filtered with the same stringency implies a common trend for upregulated genes (Extended Data Fig. 4d). DEGs that are commonly upregulated in *Mov10l1*^–/–^ and *Piwil1*^–/–^ oocytes did not exhibit common features, suggesting diverse control by piRNAs. For example, *Kif2a* was unique in employing an LTR-derived promoter to control its expression in oocytes (Extended Data Fig. 4e), suggesting that *Kif2a* expression control could occur through retrotransposon-targeting piRNAs.

**Extended Data Fig. 5:**
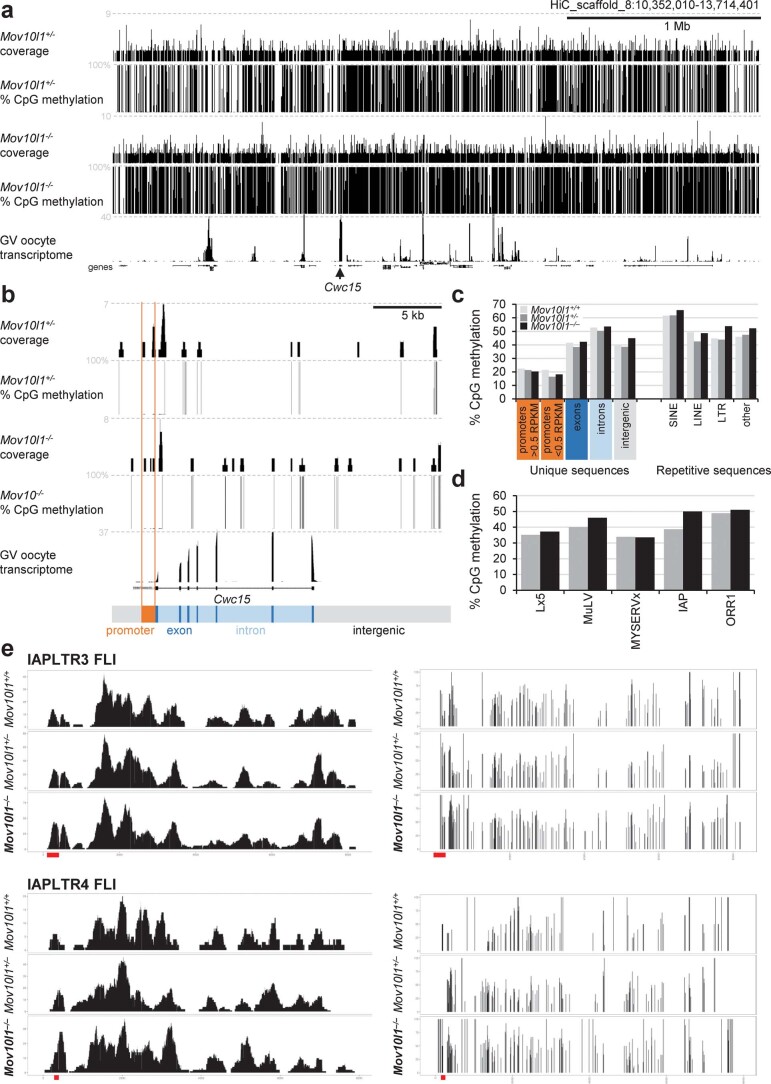
Bisulfite sequencing analysis of Mov10l1^–/–^ oocytes. Bisulfite sequencing analysis of *Mov10l1*^*–/–*^ oocytes. (a,b) Snapshots from the UCSC genome browser depicting coverage of the genome by bisulfite-sequenced fragments and CpG methylation frequency. For each genotype, one sequencing library was constructed from 10 oocytes. (b) A detailed UCSC genome browser snapshot depicts expressed *Cwc15* gene with apparent absence of DNA methylation in the promoter. (c) Quantification of distribution of CpG methylation in hamster oocytes in unique and selected repetitive sequences. Only regions covered by at least four fragments in *Mov10l1*^*+/–*^ and *Mov10l1*^*–/–*^ libraries were included in the analysis. Promoters were considered regions 1 kb upstream of an annotated transcription start sites and were divided into two groups according to their activity using an arbitrary expression threshold of 0.5 RPKM. (d) CpG methylation of selected retrotransposon subgroups in *Mov10l1*^*+/–*^ and *Mov10l1*^*–/–*^ libraries. (e) CpG methylation in IAP full-length intact retrotransposons. Panels on the left depict “coverage” of the retrotransposon genome (number of sequencing reads), panels on the right display methylation frequency at CpGs (vertical lines). Different coverage of 5’ and 3’ LTR sequences (which are identical) comes from the fact that sequencing was done as “paired-end sequencing”, hence if one read mapped exclusively to an LTR, the second read typically allowed to distinguish between 3’ and 5’ LTRs. Red lines depict CpG positions shown in Fig. 3g. Source data

Developmental competence could also be affected by derepression of TEs. A small increase (~25%) in reads mapping to intact L1 transcripts was observed in *Mov10l1*^–/–^ oocytes (Fig. 3e). RNA-sequencing (RNA-seq) analysis also revealed a limited increase in LTR retrotransposon transcripts in *Mov10l1*^–/–^ oocytes—a 2-fold increase for all IAP sequence reads (Fig. 3f) and a 3.5-fold increase for reads perfectly matching intact elements (Fig. 3e). This suggests impaired repression of intact IAPs in *Mov10l1*^–/–^ oocytes, without global IAP derepression. This notion was supported by whole-genome bisulfite sequencing (Fig. 3g). The limited amount of material yielded information about ~10% of the hamster genome (Extended Data Fig. 5). While the loss of DNA methylation at unique loci would have escaped detection (including those identified in *Piwil3*^–/–^ hamster oocytes^34^), bisulfite sequencing of *Mov10l1*^–/–^ oocytes yielded enough unique sequencing reads to examine global methylation levels of retrotransposon subfamilies (Extended Data Fig. 5d). Overall DNA methylation of intact IAP elements was preserved, although several CpG positions in LTRs could have reduced methylation frequency (Fig. 3g and Extended Data Fig. 5e). Furthermore, retrotransposon subfamilies that recently expanded in the hamster genome and were associated with high amounts of piRNAs in hamster testes (Extended Data Fig. 2a) did not show reduced DNA methylation either (Extended Data Fig. 5d).

### Sterile phenotype of *Mov10l1*^–/–^ males

**Fig. 4:**
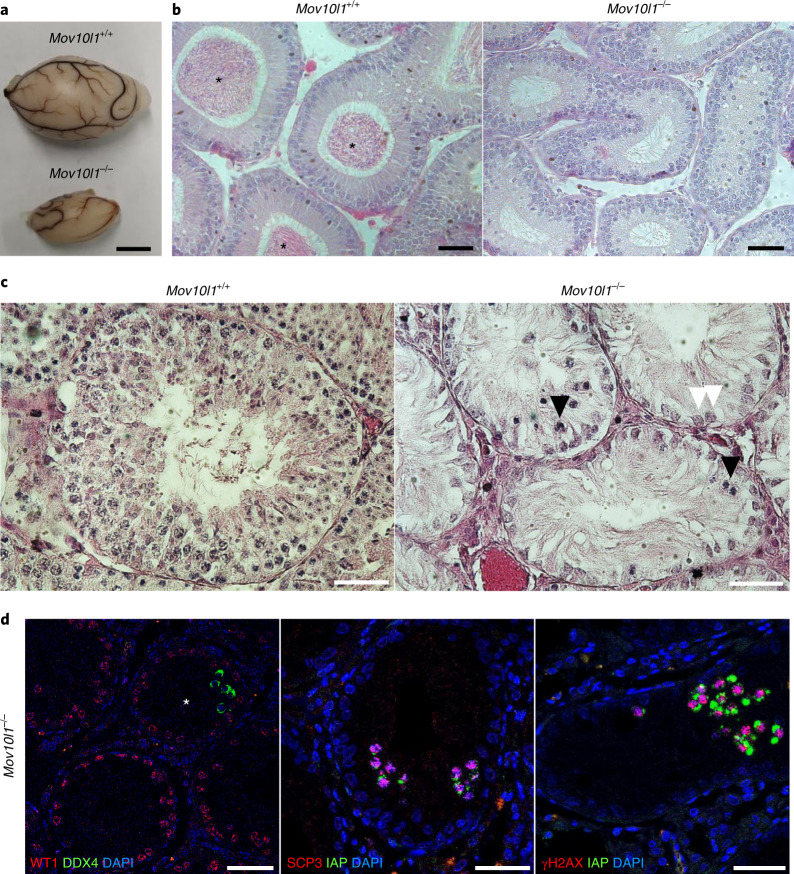
Male *Mov10l1*^–/–^ phenotype. **a**, *Mov10l1*^–/–^ males have atrophic testes. Scale bar, 5 mm. **b**, H&E staining of epididymal ducts shows a lack of sperm production in adult *Mov10l1*^–/–^ testes. Sperms in *Mov10l1*^+/+^ epididymal ducts are indicated (asterisks). Scale bars, 50 μm. **c**, H&E staining of testes from *Mov10l1*^+/+^ (aged 102 weeks) and *Mov10l1*^–/–^ (aged 63 weeks) male hamsters. The white arrowheads indicate Sertoli cells and the black arrowheads indicate degenerated cells (a large field of view is provided in Extended Data Fig. 6). Scale bars, 50 μm. **d**, Analysis of residual clusters of spermatogenic cells in seminiferous tubules in *Mov10l1*^–/–^ adult testes (asterisk). Left, somatic Sertoli cells (WT1, red) and a cluster of germ cells (DDX4, green)^37–39^. Middle, SCP3^+^ clusters exhibit IAP expression (green). Right, cells expressing IAP (green) have DNA damage (γH2AX, red). Two animals with each genotype were used for histological analyses, three sections from each animal were stained and representative images are shown. Scale bars, 50 μm.

**Extended Data Fig. 6:**
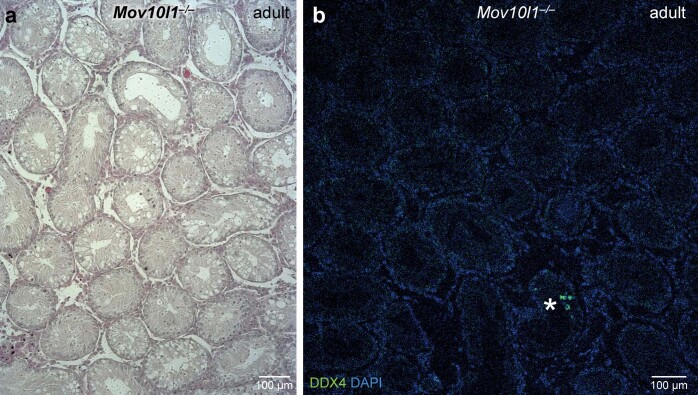
Sterility in Mov10l1^–/–^ males. Sterility in *Mov10l1*^*–/–*^ males. (a) Most of the testicular seminiferous tubules in adult *Mov10l1*^*–/–*^ testes (53 weeks) appear aspermatogenic in H&E staining. (b) Immunofluorescent staining with germ cell marker DDX4 (VASA) reveals rare clusters of spermatogenic cells (97% of tubules do not contain any germ cells). For both panels, testis sections from two adult males (three sections from each testis) were individually stained. Representative examples are shown.

Adult *Mov10l1*^–/–^ males were sterile and had atrophic testes (Fig. 4a) as well as epididymal ducts devoid of sperm (Fig. 4b). Although the sterility of *Mov10l1*^–/–^ male hamsters was consistent with the mouse *Mov10l1*^–/–^ model phenotype^3–5^, we sought to determine whether there is the same spermatogenesis defect. Histological analysis of old *Mov10l1*^–/–^ males (>50 weeks) revealed aspermatogenic seminiferous tubules (Fig. 4c and Extended Data Fig. 6a). Approximately 3% of adult seminiferous tubules contained small clusters of cells positive for the germ cell marker DDX4 (VASA)^37^ and the marker of meiotic cells synaptonemal complex protein 3 (SCP3)^38^ (Fig. 4d and Extended Data Fig. 6b). These clusters were also positive for the IAP GAG protein and γH2AX, a common marker of DNA damage suggesting compromised genome integrity (Fig. 4d). Derepression of L1 retrotransposons was absent or the anti-ORF1 antibody did not cross-react with the hamster protein. The clusters of rare survivors of germ cell ‘atresia’ therefore seem to develop a secondary phenotype that is similar to the main phenotype of *Mov10l1*^–/–^ mice, whereby spermatogenesis fails after entry into meiosis^3–5^. By contrast, the primary spermatogenesis defect in *Mov10l1*^–/–^ hamsters appears before the entry into meiosis.

**Fig. 5:**
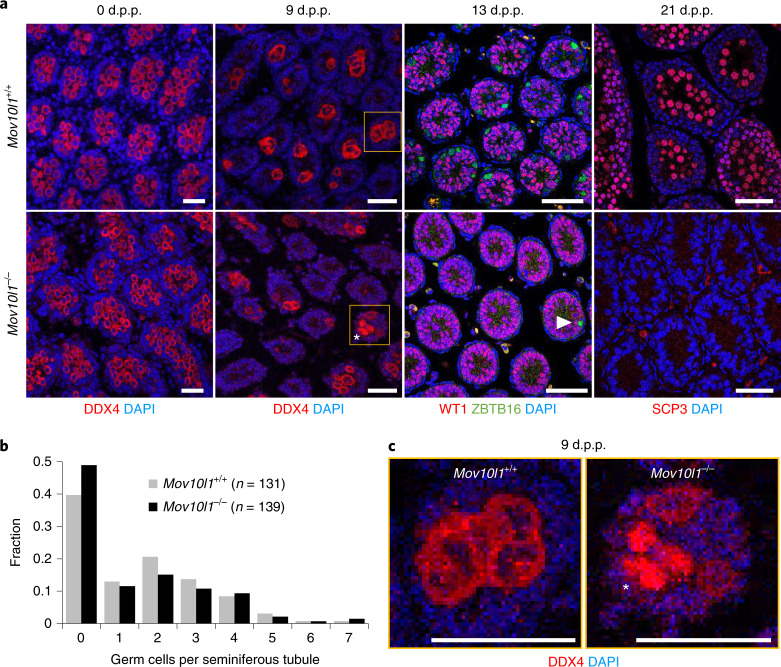
Analysis of the timing of the spermatogenesis defect in *Mov10l1*^–/–^ males. **a**, Immunofluorescence staining of *Mov10l1*^+/+^ and *Mov10l1*^–/–^ testes at 0 d.p.p., 9 d.p.p., 13 d.p.p. and 21 d.p.p. to examine the presence of germ cells (marked by DDX4), undifferentiated spermatogonia (marked by ZBTB16)^39^ and spermatocytes (marked by SCP3)^38^. DNA was stained with DAPI. Several histological sections from two different animals were analysed at all stages. Aberrant DDX4 staining observed at 9 d.p.p. is indicated (asterisk) (a magnified image is shown in **c**). The arrowhead indicates a single ZBTB16^+^ spermatogonium in a *Mov10l1*^–/–^ 13 d.p.p. sample. Scale bars, 50 μm. **b**, Quantitative analysis of germ cell distribution in sections of 9 d.p.p. seminiferous tubules. For each genotype, the indicated number of seminiferous tubules on several sections was examined for the presence of DDX4^+^ germ cells. There was no statistically significant association between genotype and tubules being empty or non-empty (*χ*^2^ test, *P* = 0.138). **c**, Higher magnification of the seminiferous tubule cross-sections shown in **a**. *Mov10l1*^–/–^ germ cells are indicated (asterisk), which show deviation from the normal DDX4 staining pattern in which strong cytoplasmic staining would surround a nucleus with a minimal signal. Scale bars, 50 μm. Source data

To understand the loss of germ cells in *Mov10l1*^–/–^ hamster testes, we examined new-born (0 d.p.p.), 9 d.p.p., 13 d.p.p. and 21 d.p.p. animals (Fig. 5a). Previous research has shown that new-born testes contain mitotically quiescent gonocytes that reinitiate mitosis and move to the seminiferous tubule periphery by 9 d.p.p. and give rise to spermatogonia by 13 d.p.p. (ref. ^28^). In 21 d.p.p. testes, spermatogenesis proceeds as far as the pachytene stage of meiosis I (ref. ^28^). We detected DDX4^+^ cells in new-born and 9 d.p.p. *Mov10l1*^–/–^ testes (Fig. 5a). Although 9 d.p.p. *Mov10l1*^–/–^ testes appeared normal (Fig. 5b), some seminiferous tubules exhibited aberrant localization of DDX4 (Fig. 5c), suggesting that the main spermatogenesis defect precedes the formation of spermatogonia. Accordingly, 13 d.p.p. seminiferous tubules were almost devoid of ZBTB16^+^ cells, the marker of undifferentiated spermatogonia^39^ (Fig. 5a). At 21 d.p.p., we observed smaller testes, altered seminiferous tubule architecture and a complete absence of SCP3^+^ meiotic cells (Fig. 5a). This implied that the surviving *Mov10l1*^–/–^ spermatogonia were probably compromised as they did not enter the first wave of meiosis on time.

**Fig. 6:**
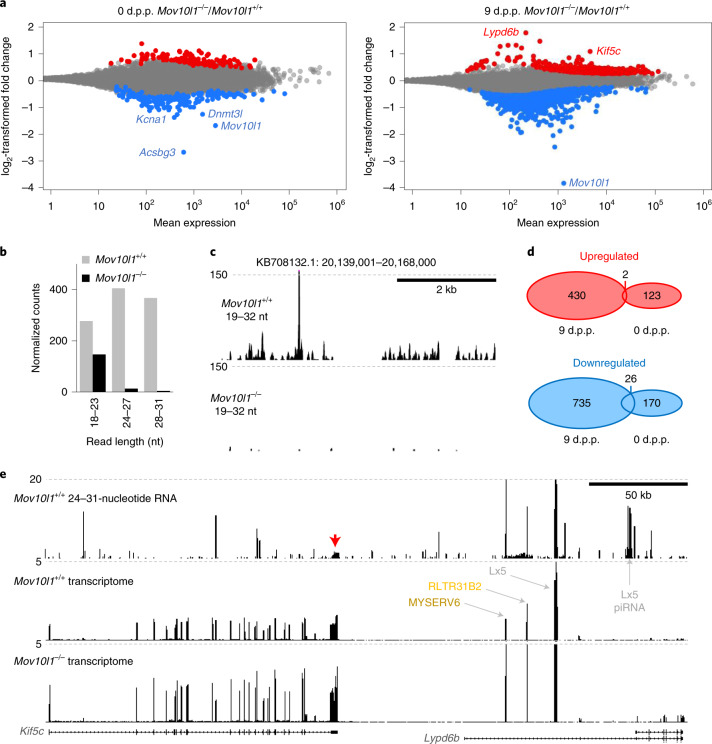
Spermatogenesis defects in *Mov10l1*^–/–^ testes. **a**, MA plots showing differentially expressed protein-coding genes at 0 d.p.p. and 9 d.p.p. (two sequencing libraries per genotype and per time point; DESeq2, *P* < 0.01). The red and blue points depict genes of which the transcripts were present at significantly higher or lower levels, respectively, in *Mov10l1*^–/–^ testes (DEG lists are provided in Supplementary Tables 7 and 8). **b**, Loss of piRNAs in *Mov10l1*^–/–^ 9 d.p.p. testes. The abundance of reads of different sizes mapping to annotated 9 d.p.p. piRNA clusters (Supplementary Table 1) is shown. Data are the mean values of three (*Mov10l1*^+/+^) and two (*Mov10l1*^–/–^) RNA-seq libraries. **c**, A snapshot from the UCSC genome browser revealing a loss of piRNAs in the top 9 d.p.p. cluster. **d**, The number of DEGs and a minimal overlap between significantly upregulated (red Venn diagram) and downregulated (blue Venn diagram) DEGs in *Mov10l1*^–/–^ 0 d.p.p. and 9 d.p.p. testes. **e**, A UCSC browser snapshot showing small RNAs and longer transcripts in *Mov10l1*^+/+^ and *Mov10l1*^–/–^ testes at the *Kif5c-Lypd6b* locus. The red arrow indicates a cluster of unique piRNAs derived from the *Kif5c* 3′ UTR. Three retrotransposon insertions in intron 1 of *Lypd6b* with increased density of mapped reads are also shown. Only perfectly mapping RNA-seq reads were used to construct the image. Source data

**Extended Data Fig. 7:**
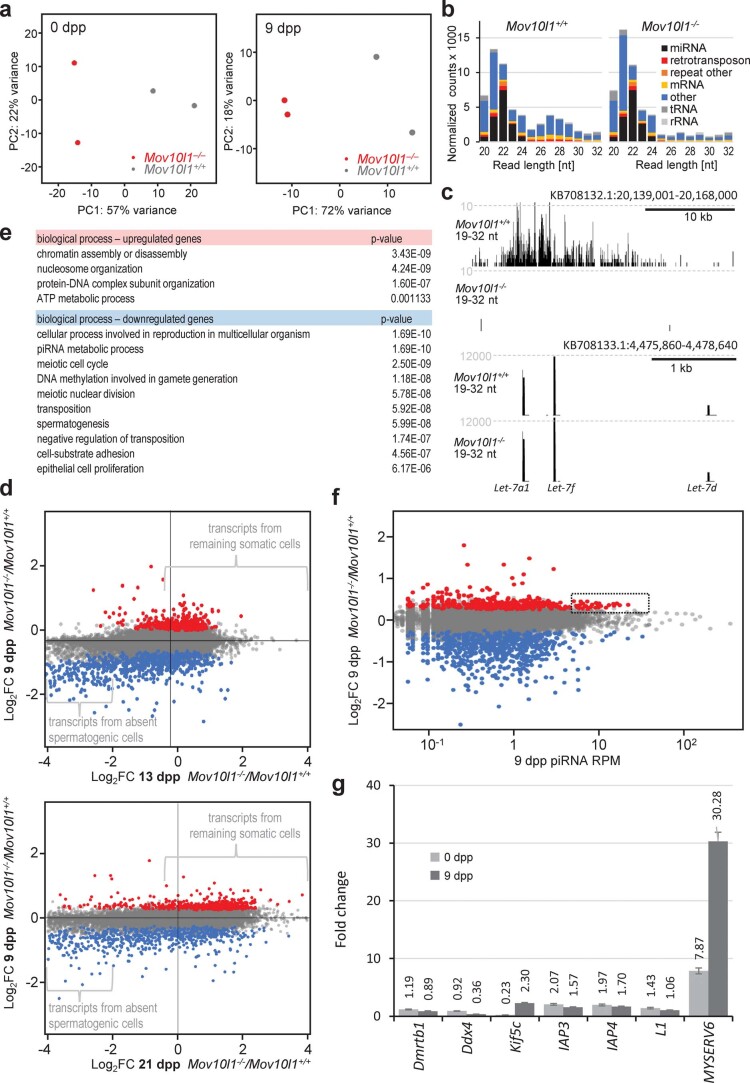
Transcriptome changes in Mov10l1^–/–^ 9 d.p.p. testes. Transcriptome changes in *Mov10l1*^*–/–*^ 9 d.p.p. testes. (a) PCA analyses of individual RNA-seq libraries produced in biological duplicates from newborn and 9 d.p.p. testes. (b) Composition of 20-32 nt RNA population in *Mov10l1*^*+/+*^ and *Mov10l1*^*–/–*^ 9 d.p.p. testes. Small RNA abundance in wild-type controls corresponds to reads per million of 18-32 nt reads. *Mov10l1*^*–/–*^ libraries were normalized to the amount of testicular miRNAs. (c) Snapshots from the UCSC genome browser revealing loss of piRNAs in another top 9 d.p.p. cluster (in addition to the one shown in Fig. 6c) and the *Let-7* miRNA locus demonstrating intact miRNA expression. Three libraries from *Mov10l1*^*+/+*^ and two from *Mov10l1*^*–/–*^ testes were produced and used for analysis in b) and c). (d) Germ cell-specific genes are among downregulated DEGs at 9 d.p.p. Red and blue points depict significantly upregulated and downregulated DEGs in 9 d.p.p. testes (DESeq2 p-value <0.01). Gene expression changes in *Mov10l1*^*–/–*^ 9 d.p.p. testes (y-axis) were plotted against gene expression changes in *Mov10l1*^*–/–*^ 13 d.p.p. (upper graph) or 21 d.p.p. (lower graph) testes. Since 13 and 21 d.p.p. *Mov10l1*^*–/–*^ testes lack most germ cells, germ cell-specific genes will be among strongly downregulated DEGs on the left side. (e) Gene Ontology analysis of enriched biological processes among DEGs in 9 d.p.p. testes. (f) Weak relationship between upregulated DEGs and piRNAs. Gene expression changes in *Mov10l1*^*–/–*^ 9 d.p.p. testes (y-axis) were plotted against rank-sorted abundance of 24-31 nt reads mapping to exons of these genes (multimapping reads were weighted). The rectangle frames a small upregulated fraction of DEGs associated with more abundant piRNAs (> ~5 RPM). (g) Quantitative RT-PCR analysis of expression changes of selected genes and retrotransposons in *Mov10l1*^*–/–*^ newborn and 9 d.p.p. testes. Numbers above each bar represent fold changes. One biological sample from each genotype was analyzed in a technical triplicate. Data are presented as relative fold changes of normalized median expression values in mutants and wild type controls calculated with the ΔΔCT method in the REST tool^53^. Error bars delineate 95% confidence interval estimated by REST. Source data

The cause of the germ cell loss was further investigated by transcriptome profiling of 9 d.p.p. testes, which identified a complete loss of piRNAs and >900 DEGs (Fig. 6, Extended Data Fig. 7 and Supplementary Table 7). Many downregulated genes were factors expressed in spermatogenic cells (Extended Data Fig. 7d,e), including germline factors *Sohlh1* (ref. ^40^) *Ddx25* (ref. ^41^), *Ddx4* (ref. ^37^), *Dazl* (ref. ^42^) and many piRNA pathway components (Supplementary Table 7). By contrast, new-born *Mov10l1*^–/–^ testes exhibited ~300 DEGs (Supplementary Table 8 and Fig. 6a) but there was minimal overlap with 9 d.p.p. testes (Fig. 6d) and no significant enrichment of any biological process according to a Gene Ontology analysis. This shows that the loss of piRNAs affects gene expression already in new-born testes, but germ cell development fails later and the failure involves a different set of DEGs. Thus, the prespermatogonial germline failure may have two causes.

First, the loss of 26–27-nucleotide postnatal piRNAs originating from non-repetitive sequences, including mRNA 3′ untranslated regions (UTRs)^8^, may increase gene expression that is otherwise restricted by the piRNA pathway. However, we observed that only a small fraction of upregulated genes was associated with higher amounts of non-repetitive piRNAs from the same loci (Extended Data Fig. 7f). Most of the observed increases in gene expression appeared small (Fig. 6a), but expression changes could be under-represented when genes are also expressed in cells other than spermatogenic cells (Extended Data Fig. 7d). Notably, *Lypd6b* and *Kif5c*, two of the three most upregulated genes are genomic neighbours, suggesting a common regulation of the locus, which might involve *Kif5c* 3′-UTR-derived piRNAs and/or derepression of retrotransposons in the first *Lypd6b* intron (Fig. 6e).

**Fig. 7:**
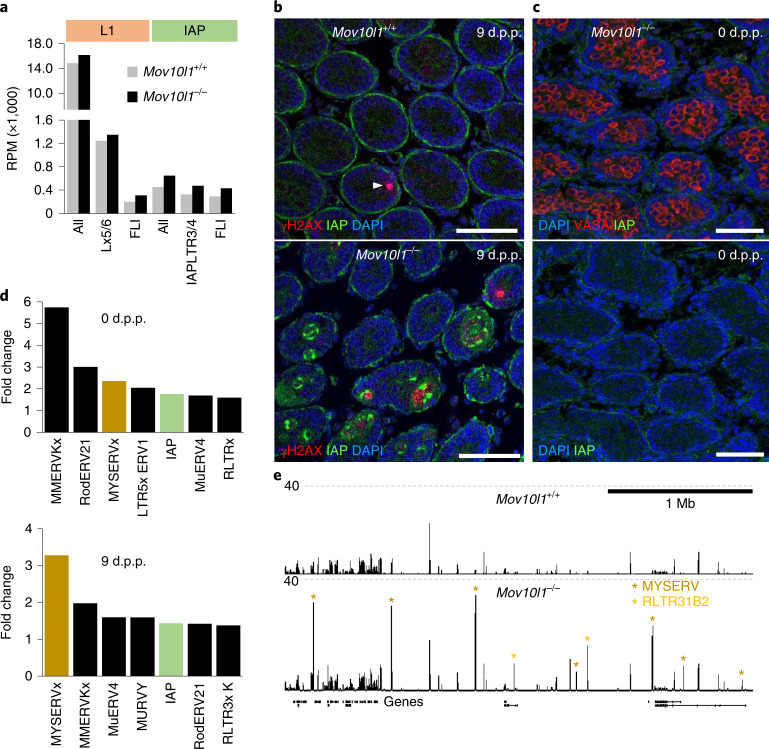
Retrotransposon mobilization in *Mov10l1*^–/–^ testes at 9 d.p.p. **a**, Changes in RNAs from L1 and IAP families and subfamilies. RPM values of RNAs mapping to L1 or IAP elements (all), active subfamilies and FLI elements only are shown. Data are the mean values of two biological replicates. **b**, Immunofluorescence staining of IAP GAG (green) and γH2AX (red) in *Mov10l1*^+/+^ and *Mov10l1*^–/–^ testes at 9 d.p.p. suggests IAP expression and DNA damage in germ cells in seminiferous tubules. Scale bars, 50 μm. **c**, 0 d.p.p. *Mov10l1*^–/–^ testes show a normal presence of the germ cell marker DDX4 (VASA) and no mobilization of IAP expression. Four (**b**) and three (**c**) sections from one testis with a given genotype were individually stained. Representative images are shown. **d**, Changes in retrotransposon expression. The graphs rank the most upregulated LTR retrotransposon groups in *Mov10l1*^–/–^ testes at 0 d.p.p. and 9 d.p.p. Values were calculated as RPM mean values from two RNA-seq libraries (Supplementary Table 10) from each time point and genotype. **e**, MYSERV and related RLTR31B2 LTR-derived transcripts are upregulated in *Mov10l1*^–/–^ testes at 9 d.p.p. A UCSC browser snapshot shows a 3 Mb genomic region with upregulated retrotransposon loci (asterisks). Only perfectly mapping RNA-seq reads were used to construct the image. Source data

**Extended Data Fig. 8:**
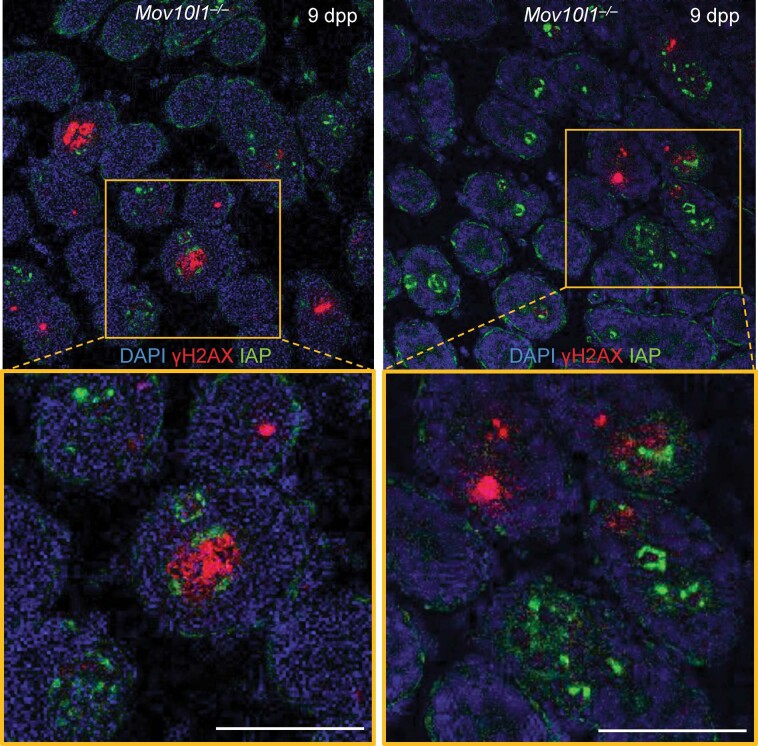
Increased expression of IAP GAG protein in Mov10l1^–/–^ 9 d.p.p. testes. Increased expression of IAP GAG protein in *Mov10l1*^*–/–*^ 9 d.p.p. testes. Fluorescence staining of IAP GAG and γH2AX in two different stained histological sections show that increased IAP expression in germ cells in seminiferous tubules is not necessarily accompanied by immediate formation of γH2AX foci in the nucleus. Scale bars = 50 μm. Four sections from one testis were individually stained, two examples from two different sections are shown.

Second, formation of spermatogonia in 9 d.p.p. *Mov10l1*^–/–^ testes could also be perturbed by derepression of specific retrotransposons. An analysis of intact L1 and IAP transcripts suggested respective approximate increases of 56% and 48% in their abundance (Fig. 7a). This mild increase contrasted with IAP and γH2AX signals in *Mov10l1*^–/–^ seminiferous tubules that were detected at 9 d.p.p. but not at 0 d.p.p. (Fig. 7b,c). However, a similar situation concerning IAP transcript levels and immunofluorescent staining occurred when comparing mouse *G9a*^–/–^ and *Mili*^–/–^ (Piwil2) spermatogonia^43^. Notably, IAP and γH2AX patterns differed among 9 d.p.p. seminiferous tubules. Some contained mostly IAP signal, some massive γH2AX, and some both, probably capturing the demise of germ cells at 9 d.p.p. (Fig. 7b and Extended Data Fig. 8).

An analysis of MYSERV LTR retrotransposons provides even stronger support that the mobilization of retrotransposons may explain the loss of control over spermatogenesis. MYSERV elements give rise to highly abundant 9 d.p.p. piRNAs (Fig. 1c and Extended Data Fig. 2a). In 9 d.p.p. *Mov10l1*^–/–^ testes, MYSERV-derived reads were 3.3-fold more abundant (Fig. 7d), but MYSERV6 subfamily insertions across the genome exhibited a ~20-fold increase (Fig. 7e), which is consistent with quantitative PCR (qPCR) data (Extended Data Fig. 7g). Derepression of MYSERV retrotransposons, which was already apparent at day 0 (Fig. 7d), implies that there was genome-wide failure to silence MYSERV insertion loci, which could subsequently contribute to the failure of germ cells to form spermatogonia.

## Discussion

Our analysis of *Mov10l1*^–/–^ golden hamsters, complemented by analysis of additional piRNA pathway mutants by Hasuwa et al^34^. and Zhang et al.^44^, reports critical pre-meiotic, meiotic and post-meiotic functions of the mammalian piRNA pathway. Our results substantially expand the known roles of the pathway in control of the mammalian germline cycle. Furthermore, we provide an evolutionary perspective for the piRNA pathway roles while refuting the notion that the mammalian piRNA pathway is important only for the male germline.

The post-meiotic and post-zygotic sterility observed in female *Mov10l1*^–/–^ hamsters adds to the repertoire of critical roles of the mammalian piRNA pathway contribution to formation of developmentally competent oocytes, which can support early development. This contrasts with mice, in which the females remain fertile even in the absence of the piRNA pathway^3,4,13–16^.

One of the notable differences between mice and golden hamsters is that mice lost *Piwil3* while golden hamsters have four different PIWI effector proteins like most mammals, including humans. *Piwil3* seems to represent an oocyte-specific aspect of the mammalian piRNA pathway^10,11,21,34,44^. However, female subfertility of *Piwil3*^–/–^ golden hamsters is a weaker phenotype^34^ than female sterility observed in mutants in other components of the pathway in golden hamsters, which include *Mov10l1* reported here and in ref. ^44^, *Piwil1* (refs. ^34,44^) and *Pld6* (ref. ^44^). This suggests that, although the *Piwil3* is an important factor, the piRNA pathway has additional important non-overlapping functions. What these functions are remains to be clarified. Although we observed changes in *Mov10l1*^–/–^ oocytes, which possibly contribute to the sterile phenotype, the mechanism of the loss of developmental competence of *Mov10l1*^–/–^ oocytes remains to be further delineated.

Changing requirements for the piRNA pathway during evolution could lead to the apparent insignificance of the piRNA pathway in mouse oocytes. However, our results provoke the question of whether the piRNA pathway in mouse oocytes could have a similar role in formation of developmental competence, which would be masked in piRNA-pathway mutants by overlapping functions of the murine RNAi pathway^19^. Unfortunately, massive transcriptome changes and meiotic defects in oocytes lacking RNAi^45,46^ preclude the examination of the functional significance of the piRNA pathway in mouse zygotes in the absence of RNAi.

An analysis of *Mov10l1*^–/–^ male hamsters showed that MOV10L1 is required for the postnatal formation of pre-meiotic spermatogonia. The massive loss of germ cells before the spermatogonia form differs from the phenotypes in mice with mutations in the piRNA pathway, which occur after the spermatogonia form. Although *Miwi2*^–/–^ mice revealed that piRNAs regulate DNA methylation in prospermatogonia, this role is not important for the establishment of spermatogonia, which form in *Miwi2*^–/–^ mice but are then lost progressively until the testes become aspermatogenic by 9 months^15,47–49^.

The failure of male *Mov10l1*^–/–^ hamsters to produce spermatogonia appears to be associated with the derepression of the MYSERV, hamster-specific retrotransposon subfamily expressed in juvenile testes. First, the absence of the piRNA pathway in the male germline yielded a failure at a stage at which MYSERV expression peaks. Second, an outstanding abundance of MYSERV-targeting piRNAs in juvenile testes implies that the element is a major target of the piRNA pathway at that stage. While most retrotransposons in mouse and hamster genomes probably descended from the common ancestor, recently expanded subfamilies represent independently evolved retrotransposon pools, which would provoke an independent adaptive response of the piRNA pathway. Hamster-specific retrotransposon derepression could therefore explain why spermatogonia form in mice lacking *Mov10l1* (refs. ^3–5^). The model of retrotransposon-induced failure of spermatogenesis is also consistent with a rapid demise of mouse spermatogonia whereby retrotransposon derepression was enhanced by mutating *G9a* in addition to *Mili*^50^.

Taken together, our work does not just demonstrate that the mammalian piRNA pathway is important beyond spermatogenesis. Equally important is that the divergent hamster and mouse *Mov10l1*^–/–^ phenotypes illuminate the adaptive nature of the piRNA pathway, which leads to new gene regulations and flexibly protects the germline cycle against retrotransposons whenever they would pose a new threat.

## Methods

### Animals

Golden (Syrian) hamsters *M. auratus* were purchased from Japan SLC (for knockout production and initial breeding) and from Janvier Labs (subsequent breeding). Animals were housed under controlled lighting conditions (daily light period 7:00 to 21:00 and 6:00 to 18:00 in Japan and Czechia, respectively) and provided with water and food ad libitum. The animals used for experiments were euthanized by intraperitoneal injection of a lethal dose of Euthasol (Samohyl). All of the animal experiments were approved by the Animal Experimentation Committee at the RIKEN Tsukuba Institute (T2019-J004) and the Institutional Animal Use and Care Committee at the Institute of Molecular Genetics of the Czech Academy of Sciences (approval no. 42/2016 and 70/2018) and were performed in accordance with the law.

### Production of *Mov10l1* mutants

The production of knockout hamsters was carried out using an in vivo electroporation CRISPR–Cas9 system as described previously^22,51^. Pairs of sgRNAs were designed to cleave the *Mov10l1* genomic sequence in intron 19 (sequence of DNA targets: 5′-GGGTATCACATGACTTGGGG-3′; 5′-GGTGTTGGGATCATAGTGGGG-3′) and in intron 20 (sequence of DNA targets: 5′-TCTCCACTCTTCCATGTGGGG-3′; 5′-TACCATTACATTTGTCAGGGG-3′) to delete exon 20 (Fig. 1f).

Five animals were born, of which one did not exhibit any deletion, two were homozygous for the deletion and were not used for breeding, and one male and one female showed modification of one allele (Extended Data Fig. 3a). Male 1 was fertile but did not transmit the allele, whereas female 4 transmitted the allele into progeny (number of progeny, 10; 3 males and 4 females carried the mutant allele) when bred with a wild-type animal. Subsequent breeding of heterozygotes for two generations with wild-type outbred animals was performed to minimize possible off-targeting and inbreeding effects when heterozygotes were mated to produce homozygotes. The allele bearing a 761 bp deletion comprising exon 20 (KB708136.1: 11,120,580–11,121,340) was confirmed by sequencing (Extended Data Fig. 3b). RNA-seq analysis showed a loss of signal over exon 20 and a strongly reduced transcript level of the mutant transcript (Extended Data Fig. 3c).

For genotyping, ear biopsies were lysed in PCR-friendly lysis buffer with 0.6 U per sample proteinase K (Thermo Fisher Scientific) at 55 °C, with shaking at 900 r.p.m. until dissolved (approximately 2.5 h). Samples were heat-inactivated 10 min at 90 °C and lysate was used for nested PCR reaction. Genotyping primers are provided in Supplementary Table 9.

### Superovulation and zygote collection

Female golden hamsters were induced to superovulate by intraperitoneal injection of 15 IU or 25 IU pregnant mare’s serum gonadotropin (PMSG, ProSpec Bio) at 10:00 on the day of post-oestrous vaginal discharge (day 1 of oestrous cycle). hCG (25 IU; Sigma-Aldrich) was injected 76 h later (14:00 on day 4 of the oestrous cycle) and females were mated with fertile males at 18:00 on the same day.

Zygotes were collected 40 h after mating and four-cell embryos 61 h after mating (without previous superovulation) by flushing oviducts with M199TE medium (M199 medium with HEPES, sodium bicarbonate and Eagle’s salts, Gibco), supplemented with 5% fetal bovine serum (Sigma-Aldrich) inactivated for 30 min at 56 °C, 5 mM taurine, 25 μM EDTA and pre-equilibrated with 5% CO_2_, 5% O_2_ and 90% N_2_ at 37 °C. Zygotes were isolated in a dark room with red filters on the microscope light source and were used immediately for experimental analysis as there was no highly efficient culture system for hamster zygotes and preimplantation embryos.

### Oocyte collection

Preovulatory fully grown GV oocytes were collected from ovaries by puncturing antral follicles with a syringe needle in M2 medium (Sigma-Aldrich) containing 0.2 mM 3-isobutyl-1-methyl-xanthine (Sigma-Aldrich) to prevent resumption of meiosis. In the absence of a highly efficient culture system for meiotic maturation of hamster GV oocytes in vitro, ovulated unfertilized eggs arrested at MII were collected from the oviducts of superovulated females approximately 17 h after hCG injection. MII oocytes were released from cumulus cells after incubation with 0.1% bovine testes hyaluronidase (Sigma-Aldrich) in M199TE medium at 37°C for 1 min and washed three times in equilibrated M199TE medium kept under paraffin oil.

### Western blotting

Hamster and mouse tissues were homogenized mechanically in RIPA lysis buffer supplemented with 1× protease inhibitor cocktail set (Millipore) and loaded with SDS dye. Protein concentration was measured using the Bradford assay and 60 μg of protein was used per lane. Proteins were separated on 6% polyacrylamide gel and transferred onto a polyvinylidene difluoride membrane (Millipore) using semi-dry blotting. The membrane was blocked in 5% skim milk in TTBS, MOV10L1 was detected using anti-MOV10L1 primary antibodies^4^ (gift from J. Wang) diluted 1:250 and incubated overnight at 4°C. Anti-rabbit-HRP secondary antibodies (Thermo Fisher Scientific) were diluted 1:50,000 and the signal was detected using SuperSignal West Femto Substrate (Thermo Fisher Scientific). For TUBA4A detection, samples were run on 10% polyacrylamide gel and incubated with anti-tubulin (Sigma-Aldrich, T6074) mouse primary antibodies diluted to 1:10,000 and anti-mouse-HRP secondary antibodies (Thermo Fisher Scientific) diluted to 1:50,000.

### RT–PCR analyses

For oocyte and embryo expression analysis, five to ten oocytes or embryos were collected per sample in 3 μl of PBS and snap-frozen in liquid nitrogen; the number of oocytes/embryos was kept constant in individual sample sets. Next, the samples were lysed by mixing an equal volume of the 2× lysis buffer^52^. Crude lysate was used for reverse transcription with SuperScript III (Thermo Fisher Scientific). An equal fraction of total RNA per oocyte/zygote was reverse transcribed using SuperScript III RT (Thermo Fisher Scientific) with random hexamers according to the manufacturer’s recommendations. To avoid genomic DNA amplification, primers were designed to span multiple exons.

cDNA was amplified by ExTaqHS (TaKaRa) using the following program: 94 °C for 2 min; 35 cycles of 94 °C for 10 s, 60 °C for 30 s and 72 °C for 30 s; and a final extension at 72 °C for 3 min. The PCR products were resolved on 1.5% agarose gels and visualized using ethidium bromide. All PCR products were sequenced after cloning into pCR4 TOPO vector (TOPO-TA cloning kit for sequencing; Thermo Fisher Scientific). A list of the primers is provided in Supplementary Table 9.

For qPCR analyses, a 1 µg aliquot of total RNA used for NGS library preparation was reverse transcribed in a 30 µl volume using LunaScript RT SuperMix Kit (New England Biolabs) according to the manufacturer’s instructions. A 0.5 µl cDNA aliquot and the Maxima SYBR Green qPCR master mix (Thermo Fisher Scientific) were used for the qPCR reaction. qPCR was performed on LightCycler 480 (Roche) in technical triplicates for each biological sample. Average *C*_t_ values of the technical replicates were normalized to the housekeeping genes Ma*HPRT* and Ma*B2MG* using the ΔΔ*C*_t_ method^53^. A list of the primers used for qPCR is provided in Supplementary Table 9.

### Histology and immunofluorescence analysis of histological sections

Ovaries and testes were fixed in Hartman’s fixative (Sigma-Aldrich, H0290) or 4% paraformaldehyde in PBS for 1.5 h or overnight at 4°C. Tissues were dehydrated in ethanol, embedded in paraffin, sectioned to a thickness of 2.5–6 μm and stained with H&E or used for immunofluorescence staining.

For immunofluorescence staining of testes, sections were deparaffinized and then boiled for 18 min in 10 mM pH 6 sodium citrate solution for antigen retrieval. After 45 min blocking with 5% normal donkey serum and 5% bovine serum albumin (BSA) in PBS, sections were incubated for 1 h at room temperature or overnight at 4 °C with the following primary antibodies used at 1:200 dilutions: anti-LINE1 ORF1p (provided by D. O’Carroll, University of Edinburgh), anti-SCP3 (Abcam, ab976672), anti-ZBTB16 (Atlas antibodies, HPA001499) and anti-ɣH2AX (Milipore, 05-636); and at 1:400 dilutions: anti-DDX4 (Abcam, ab27591 and ab13840) and anti-WT1 (Novus Biologicals, NB110-60011). Anti-IAP GAG antibodies (gift from B. R. Cullen) were used at a 1:500 dilution. Anti-mouse or anti-rabbit secondary antibodies conjugated with Alexa 488 or Alexa 594 (1:500; Thermo Fisher Scientific) were incubated for 1 h at room temperature. Nuclei were stained with 1 μg ml^−1^ DAPI for 10 min, slides were mounted in ProLong Diamond Antifade Mountant (Thermo Fisher Scientific) and images were acquired using the DM6000 or Leica SP8 confocal microscope.

### Immunofluorescence staining of oocytes and zygotes

Oocytes and zygotes were fixed and permeabilized with 0.2% Triton X-100 in 4% paraformaldehyde for 30 min at room temperature followed by blocking in 2% BSA in PBS for 1 h or kept in blocking buffer overnight. To visualize the meiotic spindle, MII oocytes were stained with mouse anti-Tubulin (Abcam, ab7750) diluted 1:100 for 1 h at room temperature. To visualize H3K9me3 histone modification, rabbit anti-H3K9me3 (Upstate, 07-442) was used at 1:1,000 dilution overnight at 4 °C. MII oocytes and zygotes were incubated with secondary antibody conjugated with Alexa 488 or Alexa 594 (Thermo Fisher Scientific) diluted at 1:500 for 1 h at room temperature. DNA was stained with 1 μg ml^−1^ DAPI for 10 min. The Leica DM6000 microscope and SP8 confocal microscope were used for data collection, LAS AF LITE 3.3 software (Leica) was used for image processing, and Imaris v.9.6 (Bitplane) was used to determine the length and volume of the spindle and metaphase plate by three-dimensional reconstruction.

### RNA sequencing: sequencing library preparation

For oocyte transcriptome analysis, total RNA was extracted from 6–12 fully-grown oocytes using the Arcturus Picopure RNA isolation kit (Thermo Fisher Scientific) according to the manufacturer’s protocol. RNA-seq libraries were generated using the Ovation RNA-Seq system V2 (NuGEN) followed by the Ovation Ultralow Library system (DR Multiplex System, NuGEN) according to the manufacturer’s protocol. cDNA fragmentation was performed on the Bioruptor sonication device (Diagenode) with 18 cycles of 30 s on and 30 s off at low intensity. Libraries were amplified by 9 cycles of PCR and sequenced by 100-nucleotide single-end reading using the Illumina NovaSeq6000 platform.

For small-RNA-seq analysis of oocytes, at least five oocytes were collected from each animal and were incubated at 75 °C for 3 min to release small RNAs. Small-RNA libraries were prepared using the NextFlex Small-RNA-seq v3 kit (Amplicon) according to the manufacturer’s protocol; 3′ adapter ligation was performed overnight at 16 °C, 25 cycles were used for PCR amplification and gel purification was performed for size selection. For gel purification, libraries were separated on a 2.5% agarose gel using 1× lithium borate buffer and visualized with ethidium bromide. The 140–170 bp fraction was cut off the gel and DNA was isolated using the MinElute Gel Extraction Kit (Qiagen). Final libraries were sequenced by 75-nucleotide single-end reading using the Illumina NextSeq500/550 platform.

For analysis of testicular transcriptomes, total RNA was extracted from adult, 21 d.p.p., 13 d.p.p., 9 d.p.p. and new-born (0 d.p.p.) hamster testes using the Sigma-Aldrich mirPremier microRNA isolation kit according to the manufacturer’s protocol. Ribosomal RNA (rRNA) was depleted from RNA used for transcriptome analysis using the Ribo-Zero rRNA Removal Kit (Human/Mouse/Rat) (Epicentre) or the QIAseq FastSelect–rRNA HMR Kit (Qiagen) according to the manufacturer’s protocols. rRNA depletion was confirmed using the 2100 Bioanalyzer (Agilent Technologies). RNA-seq libraries were generated using the NEBNext Ultra II directional RNA library Prep kit for Illumina (BioLabs, E7765S) according to the manufacturer’s protocol. RNA-seq libraries from adult, 21 d.p.p. and 13 d.p.p. testes were sequenced by 150-nucleotide paired-end reading and RNA-seq libraries from 9 d.p.p. and new-born hamster testes were sequenced by 75-nucleotide single-end reading using the Illumina NextSeq500/550 platform.

For small-RNA-seq analysis of testes, total RNA isolated as described above was used with the NextFlex Small-RNA-seq v3 kit (Amplicon). Libraries were prepared according to the manufacturer’s protocol with 3′ adapter ligation overnight at 16 °C, 15 cycles of PCR amplification and NextFlex beads or gel purification (described above) was used for size selection. RNA-seq libraries were sequenced by 75-nucleotide single-end reading using the Illumina NextSeq500/550 platform or 100-nucleotide single-end reading using NovaSeq6000 platform. A list of the analysed sequencing libraries is provided in Supplementary Table 10. Raw data were deposited at the Gene Expression Omnibus (GEO: GSE164658).

### Bisulfite sequencing: library preparation

For bisulfite sequencing, ten fully grown GV oocytes (an equivalent of 40 haploid genomes and 80 single-stranded DNAs after bisulfite conversion) were directly subjected to EZ DNA Methylation-Direct kit (Zymo Research) for bisulfite conversion with the following modifications: samples were digested with proteinase K at 50 °C for 35 min and bisulfite conversion was performed as follows: 98 °C for 6 min, 64 °C for 30 min, 95 °C for 1 min, 64 °C for 90 min, 95 °C for 1 min and 64 °C for 90 min. DNA libraries were prepared using the EpiNext Post-Bisulfite DNA library Preparation kit according to the manufacturer’s protocol, with 22 PCR cycles used for amplification. The final DNA libraries were sequenced by 250-nucleotide paired-end sequencing on the Illumina NovaSeq6000 platform.

### Bioinformatics analyses

#### RNA-seq and differential gene expression analysis

Raw RNA-seq reads were mapped to mouse (mm10), human (hg38), cow (bosTau9), rat (rn6), golden hamster (mesAur1) and the newest golden hamster (PRJDB10770)^11^ genomes using STAR v.2.7.3a^54^ with following parameters:


STAR --readFilesIn $ {FILE}.fastq.gz --genomeDir $ {GENOME_INDEX} --runThreadN 12 --genomeLoad LoadAndRemove --limitBAMsortRAM 20000000000 --readFilesCommand unpigz –c --outFileNamePrefix $ {FILENAME} --outSAMtype BAM SortedByCoordinate --outReadsUnmapped Fastx --outFilterMultimapNmax 5000 --winAnchorMultimapNmax 5000 --seedSearchStartLmax 30 --alignTranscriptsPerReadNmax 30000 --alignWindowsPerReadNmax 30000 --alignTranscriptsPerWindowNmax 300 --seedPerReadNmax 3000 --seedPerWindowNmax 300 --seedNoneLociPerWindow 1000 --outFilterMultimapScoreRange 0 --outFilterMismatchNoverLmax 0.05 --sjdbScore 2


Those parameters were chosen to optimize mapping for quantification of transposable elements^55^.

For analysis of expression of protein coding genes, reads were mapped with maximum of 20 multimapping alignments allowed. Reads mapped to mesAur1 were counted over exon features annotated by Ensembl (release 99) using featureCounts v.2.0.0 (ref. ^56^):


featureCounts -a $ {FILE}.gtf -o $ {FILE}.counts.txt $ {FILE}.bam -T 12 -F GTF -M -O --fraction


For the paired-end libraries -p flag was added. For the stranded libraries, -s 2 flag was added. Statistical significance and fold changes in gene expression were computed in R (https://www.R-project.org/) using DESeq2 package^57^. Genes were considered to be significantly upregulated or downregulated if their corresponding adjusted *P* values were smaller than 0.01. Principal component analysis was computed on counts data that were transformed using the regularized logarithm (rlog) function.

For the heat map showing the expression of piRNA factors in testes and oocytes of four mammalian species (Fig. 1a), the following publicly available datasets were used: bovine oocyte GSE52415 (ref. ^58^), bovine testis PRJNA471564 (ref. ^59^), human oocyte GSE72379 (ref. ^60^), human testis GSE74896 (ref. ^61^), mouse oocyte GSE116771 (ref. ^62^), mouse testis GSE49417 (ref. ^63^), rat oocyte GSE137563 (ref. ^64^) and rat testis GSE53960 (ref. ^65^). Read mapping coverage was visualized in the UCSC Genome Browser by constructing bigWig tracks using the UCSC tools^66^.

#### Small-RNA-seq analysis of testes

Small-RNA-seq reads were trimmed in two rounds using bbduk.sh v.38.87 (https://jgi.doe.gov/data-and-tools/bbtools/). First, the NEXTflex adapter was trimmed from the right end:


bbduk.sh -Xmx20G threads=6 in=$ {FILE}.fastq.gz out=$ {FILE}.atrim.fastq.gz literal= TGGAATTCTCGGGTGCCAAGG stats=$ {FILE}.atrim.stats overwrite=t ktrim=r k=21 rcomp=f mink=10 hdist=1 minoverlap=8


Next, four random bases from both sides of the reads were trimmed:


bbduk.sh -Xmx20G threads=6 in=$ {FILE}.atrim.fastq.gz out=$ {FILE}.trimmed.fastq.gz stats=$ {FILE}.ftrim.stats overwrite=t forcetrimright2=4 forcetrimleft=4 minlength=18


Trimmed reads were mapped to the genomes using the following parameters:


STAR --readFilesIn $ {FILE}.fastq.gz --genomeDir $ {GENOME_INDEX} --runThreadN 12 --genomeLoad LoadAndRemove --limitBAMsortRAM 20000000000 --readFilesCommand unpigz –c --outFileNamePrefix $ {FILENAME} --outSAMtype BAM SortedByCoordinate --outReadsUnmapped Fastx --outFilterMismatchNmax 1 --outFilterMismatchNoverLmax 1 --outFilterMismatchNoverReadLmax 1 --outFilterMatchNmin 16 --outFilterMatchNminOverLread 0 --outFilterScoreMinOverLread 0 --outFilterMultimapNmax 5000 --winAnchorMultimapNmax 5000 --seedSearchStartLmax 30 --alignTranscriptsPerReadNmax 30000 --alignWindowsPerReadNmax 30000 --alignTranscriptsPerWindowNmax 300 --seedPerReadNmax 3000 --seedPerWindowNmax 300 --seedNoneLociPerWindow 1000 --outFilterMultimapScoreRange 0 --alignIntronMax 1 --alignSJDBoverhangMin 999999999999


#### Small-RNA-seq analysis of oocytes

Small-RNA-seq reads were trimmed using bbduk.sh v.38.87:


bbduk.sh -Xmx20G threads=6 in=$ {FILE}.fastq.gz out=$ {FILE}.atrim.fastq.gz literal= TGGAATTCTCGGGTGCCAAGG stats=$ {FILE}.atrim.stats overwrite=t ktrim=r k=21 rcomp=f mink=10 hdist=1 minoverlap=8


Libraries were then deduplicated from PCR duplicates. First, using custom scripts (available at https://github.com/fhorvat) both UMI sequences from the read (four random nucleotides from each end of the trimmed read) were added to the read header. UMI sequences were then removed from read sequence using Cutadapt v.2.10 (ref. ^67^):


cutadapt -u 4 -o ${FILE}.trim_1.fastq -j 6 ${FILE}.umi.fastqcutadapt -m 18 -u -4 -o ${FILE}.trim_2.fastq -j 6 ${FILE}.trim_1.fastqTrimmed reads were then mapped to the new golden hamster genome using STAR 2.7.3a:STAR --readFilesIn $ {FILE}.dedup.fastq.gz --genomeDir $ {GENOME_INDEX} --runThreadN 12 --genomeLoad LoadAndRemove --limitBAMsortRAM 20000000000 --readFilesCommand unpigz –c --outFileNamePrefix $ {FILENAME} --outSAMtype BAM SortedByCoordinate --outReadsUnmapped Fastx --outFilterMismatchNmax 1 --outFilterMismatchNoverLmax 1 --outFilterMismatchNoverReadLmax 1 --outFilterMatchNmin 16 --outFilterMatchNminOverLread 0 --outFilterScoreMinOverLread 0 --outFilterMultimapNmax 5000 --winAnchorMultimapNmax 5000 --seedSearchStartLmax 30 --alignTranscriptsPerReadNmax 30000 --alignWindowsPerReadNmax 30000 --alignTranscriptsPerWindowNmax 300 --seedPerReadNmax 3000 --seedPerWindowNmax 300 --seedNoneLociPerWindow 1000 --outFilterMultimapScoreRange 0 --alignIntronMax 1 --alignSJDBoverhangMin 999999999999


Next, UMI tools v.1.1.1 (ref. ^68^) was used on mapped .bam files to deduplicate them:


umi_tools dedup --method=directional --multimapping-detection-method=NH -I ${FILE}.trim_1.fastq --output-stats=${FILE}.dedup_stats --log=${FILE}.dedup_log.txt -S ${FILE}.dedup.bam.


Final .fastq files were generated from deduplicated mapped reads .bam files using samtools v.1.10 (ref. ^69^):


samtools fastq -@ 12 ${FILE}.dedup.bam > ${FILE}.dedup.fastq


Those deduplicated reads were then mapped to the genomes using same STAR 2.7.3a with same parameters as above.

#### Definition of hamster piRNA clusters in testes and their analysis

Small-RNA-seq analysis of whole testes was used to distinguish pre-pachytene pre-meiotic piRNAs and meiotic pachytene piRNAs as 9 d.p.p. and 13 d.p.p. testes contain only pre-meiotic spermatogonia, whereas spermatogenesis in pubertal hamsters reaches the pachytene stage at 21 d.p.p. (ref. ^28^). piRNA clusters in testes were defined using custom R scripts as follows:

1. The genome was divided into 1 kb windows and, for each window, alignments of 24–31-nucleotide reads were counted with fractional counts. Counts were normalized to the total number of 19–32-nucleotide reads in millions into RPM. RPMs were then normalized to the length of windows without counting gaps in assembly (N nucleotides) into RPKM values. Windows with RPKM < 1 were removed.
2. For pre-pachytene clusters, neighbouring tiles were merged into clusters if their log_2_-transformed fold changes of knockout/wild-type RPKMs at 9 d.p.p. and 13 d.p.p. were lower than −2. For pachytene clusters, neighbouring tiles were merged into clusters if their log_2_-transformed fold changes of knockout/wild-type RPKMs at 21 d.p.p. were lower than −2 and log_2_-transformed fold changes of knockout/wild-type RPKMs at 13 d.p.p. were higher than −2.
3. Next, clusters were merged into superclusters if they were at most 2 kb apart.
4. Clusters were manually curated and the final RPKM values were recalculated.

For further analysis, we selected loci with a piRNA density of greater than 10 RPM per kilobase for 9 d.p.p. and 13 d.p.p. pre-pachytene piRNAs and 100 RPM for 21 d.p.p. pachytene piRNAs (Extended Data Fig. 1a).

#### Definition of hamster piRNA clusters in oocytes and their analysis

For defining oocytic piRNA clusters, we took advantage of existing analysis of PIWIL1- and PIWIL3-associated small RNAs in the oocyte identified by Ishino et al.^11^. In brief, the genome was divided into 1 kb windows and, for each window, alignments of PIWIL1- and PIWIL3-associated reads were counted with fractional counts. Counts were normalized to the total number of reads in millions into RPM. Next, RPMs were normalized to the length of windows without counting gaps in assembly (N nucleotides) into RPKM values. Windows with RPKM < 1 were removed. Finally, clusters were merged into superclusters if they were at most 2 kb apart and expression of small-RNA-seq reads in oocyte samples was calculated using fractional counts.

Only clusters with a minimum of 10 RPMs in PIWIL1 or PIWIL3 IP samples and a minimum of 10 RPMs average for combined 18–20 and 24–32 reads (excluding the 21–23-nucleotide miRNA/siRNA-rich population) in WT oocyte samples were used for subsequent analysis.

#### Gene Ontology annotation analysis

Gene Ontology annotation analysis was performed using the clusterProfiler^70^ R package.

#### piRNA sequence logos

The sequence logos (Extended Data Fig. 1e) were calculated from the primary alignments only. First, only the reads with aligned first nucleotide were selected (all reads with a clipped 5′ end were removed). Reads mapped within the piRNA clusters were then selected. The 25–31-nucleotide-long reads were used for drawing the sequence logo^71^.

#### Annotation of transposable elements

Transposable elements in new golden hamster assembly were annotated using RepeatMasker (v.4.0.9)^72^ using the mouse repeats database as the closest available annotated organism. In summarizing the expression of transposable element groups, each read was counted only once using a custom R script.

#### Bisulfite sequencing

Raw bisulfite sequencing reads were trimmed using the following parameters:


bbduk.sh -Xmx20G threads=12 in1=$ {FILE}_1.txt.gz in2=$ {FILE}_2.txt.gz out1=$ {BASE}_1.trim.txt.gz out2=$ {BASE}_2.trim.txt.gz outs=$ {BASE}_s.trim.txt.gz stats=$ {BASE}.stats literal=AGATCGGAAGAGC overwrite=t ktrim=r k=12 rcomp=t mink=8 hdist=1 minoverlap=8 minlength=25 minlength=50 tbo


Trimmed reads were mapped to the genome using Bismark^73^:


bismark --non_directional --parallel 4 --unmapped --output_dir. --temp_dir.--genome_folder $ {BISMARK_INDEX } -1 $ {FILE}_1.trim.txt.gz -2 $ {FILE}_2.trim.txt.gz $ {FILE}_s.trim.txt.gz


Next, to remove alignments arising from excessive PCR amplification, alignments were deduplicated using the deduplicate_bismark script. Methylation information for individual cytosines was then extracted using bismark_methylation_extractor. For analysis of full-length intact consensus sequences, reads were first mapped to the genome. Next, reads mapping to individual full-length intact insertions were extracted and mapped again to full-length intact consensus sequences.

### Statistics and reproducibility

All statistical analyses were performed in R software environment (https://www.R-project.org/). For transcriptome analyses, the DESeq2 package was used, which internally uses two-sided Wald test and corrects *P* values for multiple comparisons using the Benjamini–Hochberg method. DESeq2 *P* < 0.01 was considered to be significant. The *χ*^2^ test from base R was used for analysis of the Mendelian distribution of genotypes after crossing heterozygotes and for comparison of germ cell numbers in seminiferous tubules. *P* < 0.05 was considered to be significant in all tests. For the box plots of the nucleotide exchange rate analyses, the median (box centre) and first and third quartiles (bounds of box) are shown, and the whiskers extend from the bounds to the values no lower (minimum) or higher (maximum) than 1.5 × interquartile range. RT–qPCR analysis of expression changes used the ΔΔ*C*_t_ method in the REST tool^53^. The error bars delineate the 95% confidence interval estimated by REST.

In general, experiments were performed with at least duplicate independent biological samples. The number of replicates was influenced by the limited availability of the biological material stemming from time-consuming and laborious hamster breeding. The sample sizes and numbers of replicates are provided in the figure captions and, for RNA-seq analyses, in Supplementary Table 10.

No statistical method was used to predetermine the sample sizes. Two RNA-seq libraries from hamster oocytes, which were outliers in PCA and had poor quality/low complexity (clearly because of a limited amount of starting material), were excluded from analysis. Furthermore, matings where fertilization did not occur (evidenced by the absence of pronucleus formation and a lack of the presence of paternal DNA in unfertilized eggs) were excluded from the preimplantation development analysis.

The experiments were not strictly randomized as they depended on a limited amount of available biological material. However, this also precluded any deliberate selection of samples. The investigators were not blinded to allocation during the experiments and outcome assessment because of integrated animal breeding, genotyping and phenotype analysis. All replications were successful.

### Reporting Summary

Further information on research design is available in the Nature Research Reporting Summary linked to this article.

## Online content

Any methods, additional references, Nature Research reporting summaries, source data, extended data, supplementary information, acknowledgements, peer review information; details of author contributions and competing interests; and statements of data and code availability are available at 10.1038/s41556-021-00746-2.

## Supplementary information

Supplementary Information Supplementary Text, References and Data 1–5.

Reporting Summary

Peer Review Information

Supplementary Tables Supplementary Table 1: 9 d.p.p. pre-pachytene piRNA clusters. Supplementary Table 2: 13 d.p.p. pre-pachytene piRNA clusters. Supplementary Table 3: 21 d.p.p. pachytene piRNA clusters. Supplementary Table 4: mating performance. Supplementary Table 5: DEGs in *Mov10l1*^–/–^ GV oocytes (DESeq2, *P* < 0.01). Supplementary Table 6: oocyte piRNA clusters. Supplementary Table 7: DEGs in *Mov10l1*^–/–^ 9 d.p.p. testes (DESeq2, *P* < 0.01). Supplementary Table 8: DEGs in *Mov10l1*^–/–^ new-born testes (DESeq2, P < 0.01). Supplementary Table 9: primers. Supplementary Table 10: list of RNA-seq libraries produced during this study. Raw data were deposited at the GEO (GSE164658).

## Data Availability

All data are available in the main text or the Supplementary Information. High-throughput sequencing data have been deposited in the Gene Expression Omnibus (GEO) under the accession code GSE164658. Previously published data that were reanalysed here are available under accession codes GSE5241556, PRJNA471564, GSE7237958, GSE7489659, GSE11677160, GSE4941761, GSE13756362 and GSE5396063. All other data supporting the findings of this study are available from the corresponding authors on reasonable request. Inquiries concerning the hamster piRNA pathway analysis should be directed to P.S. (svobodap@img.cas.cz), inquiries concerning hamster animal model and its genome manipulation (iGONAD) should be directed to A.O. (ogura@rtc.riken.go.jp). Source data are provided with this paper.

## Source data

Source Data Fig. 1 Statistical source data.
Source Data Fig. 1 Unprocessed western blots.
Source Data Fig. 2 Statistical source data.
Source Data Fig. 2 Unprocessed gels.
Source Data Fig. 3 Statistical source data.
Source Data Fig. 5 Statistical source data.
Source Data Fig. 6 Statistical source data.
Source Data Fig. 7 Statistical source data.
Source Data Extended Data Fig. 1 Statistical source data.
Source Data Extended Data Fig. 2 Statistical source data.
Source Data Extended Data Fig. 3 Unprocessed gels.
Source Data Extended Data Fig. 5 Statistical source data.
Source Data Extended Data Fig. 7 Statistical source data.

## Code availability

The code used for sequence data analysis is available at GitHub (https://github.com/fhorvat/bioinfo_repo/tree/master/papers/piRNA_2021).
