# Supplementary material for: Formation of spermatogonia and fertile oocytes in golden hamsters requires piRNAs
Source: Nat Cell Biol. 2021 Sep 6;23(9):992–1001. doi: 10.1038/s41556-021-00746-2 (PMC8437802; doi:10.1038/s41556-021-00746-2)
Supplement: Supplementary file 1 — Supplementary Text, References and Data 1–5. [file 41556_2021_746_MOESM1_ESM.pdf]

nature portfolio

https://doi.org/10.1038/s41556-021-00746-2

Supplementary information

Formation of spermatogonia and fertile oocytes in golden hamsters requires piRNAs

In the format provided by the authors and unedited

Supplementary Information

Formation of spermatogonia and fertile oocytes in hamsters requires piRNAs

Zuzana Loubalova[1†], Helena Fulka[1,†‡], Filip Horvat[1,2], Josef Pasulka[1], Radek Malik[1], Michiko Hirose[3], Atsuo Ogura[3,4*], Petr Svoboda[1*]

[1] Institute of Molecular Genetics of the Czech Academy of Sciences, Videnska 1083, 142 20 Prague 4, Czech Republic.
[2] Bioinformatics Group, Faculty of Science, University of Zagreb, 10000, Zagreb, Croatia.
[3] Bioresource Engineering Division, RIKEN BioResource Research Center, 305-0074 Ibaraki, Japan.
[4] Bioresource Engineering Laboratory, RIKEN Cluster for Pioneering Research, 351-0198 Saitama, Japan.

Table of Content

Supplementary Text
Supplementary References
Supplementary Data 1 ERVK LTR retrotransposon nucleotide exchange rates
Supplementary Data 2 Other LTR retrotransposon nucleotide exchange rates
Supplementary Data 3 MaLR LTR retrotransposon nucleotide exchange rates
Supplementary Data 4 Golden hamster IAP full-length intact insertions
Supplementary Data 5 Golden hamster LINE L1 full-length intact insertions

Supplementary text

Hamster postnatal testicular piRNA analysis

Consistent with mouse data[74], pachytene piRNAs dominated the small RNA population at 21 dpp, their length peaked at 30 nt (Extended Data Fig. 1), most of them originated from a limited (~100) number of typically intergenic loci, and were absent at 9 dpp and minimal at 13 dpp. For further analysis we used 116 clusters with >100 RPM (Supplementary Table 3). When ranked by the contribution to the piRNA pool at 21 dpp, the first 20 clusters contributed 53% of 24-31 nt small RNAs and were syntenic with piRNA-producing loci in mouse, bovine and human testes (Supplementary Table 3). The first 50 clusters produced majority (>90%) of 24-31 nt small RNAs in 21 dpp testes and >90% were syntenic with piRNA clusters in the mouse genome (Supplementary Table 3). Most of pachytene piRNAs started with U, a small fraction of pachytene piRNAs carried non-templated U additions at the 3' end (Extended Data Fig. 1e). 10A, the signature of the "ping-pong" mechanism, was weak if any in pachytene piRNAs (Extended Data Fig. 1e).

Hamster retrotransposon analysis

As one of the key roles of piRNAs is repression of retrotransposons, we determined the hamster retrotransposon complement in order to reveal potentially active retrotransposons, their expression, and abundance of sense and antisense piRNAs carrying their sequences. Although some information could be extracted from the published *Mesocricetus auratus* Mesaur1.0 and *Cricetulus griseus* (Chinese hamster) criGriChoV2 genomes, this analysis was fragmentary owing to the incompleteness of the golden hamster genome and sequence divergence of the Chinese hamster genome. For analysis of solo long-terminal repeats (LTRs) and other short retrotransposon insertions, Mesaur 1.0 could yield acceptable results[27] but for long autonomous retrotransposon analysis, it was inadequate. For example, Mesaur1.0 contains several larger fragments of IAP retrotransposon covering most of its internal sequence but not a single full-length element matching the published full-length IAP sequence[75]. L1 retrotransposon insert assemblies are even worse in Mesaur1.0. Although this could be partially remedied by using Chinese hamster genome data and raw sequencing data from Mesaur 1.0, the situation was problematic for rigorous analysis of golden hamster retrotransposons.
The issue was solved by Ishino et al. who re-sequenced and re-assembled the golden hamster genome to a quality, which enabled a rigorous analysis[11]. Subsequently, we annotated retrotransposons using the RepeatMasker and de-novo RepeatModeler. Good concordance between the two annotations

suggested that no element group was missed by RepeatMasker. We subsequently followed RepeatMasker annotation to maintain consistence with annotated murine TE groups.

L1 elements in the hamster genome

The most successful autonomous TEs invading mammalian genomes are L1 elements[32], which represents non-LTR Long Interspersed Element (LINE) class. Analysis of nucleotide exchange rates of L1 subfamilies in mouse and hamster genomes revealed divergent L1 evolution in mouse and hamster lineages (Extended Data Fig. 2d). Murine L1Md subfamilies, which gave rise to most murine full-length intact L1 elements[33], showed minimal nucleotide exchange rates. Relatively low nucleotide exchange rates were observed for additional murine L1 subfamilies (Extended Data Fig. 2d). In contrast, hamster L1 subfamilies had relatively high nucleotide exchange rates except for the Lx5 subfamily, which apparently gave rise to the youngest L1 pool in the golden hamster genome (Extended Data Fig. 2d). This notion was consistent with identification of 110 full-length intact insertions, 108 of which came from the Lx5 subfamily (Supplementary Data 5). Analysis of Chinese hamster (*Cricetulus griseus*) genome criGriChoV2 revealed 103 of full-length intact L1 insertions but most of them were annotated L1_1 and only 11 as Lx6 suggesting that a different closely-related L1 subfamilies expanded in both hamster species. The number of golden hamster full-length intact L1s is comparable to the 146 full-length intact L1s in the human genome but is much smaller than the 492 in the rat genome or the 2,811 in the mouse genome[33].

LTR elements in the hamster genome

Another main type of autonomous TEs are long terminal repeat (LTR) retrotransposons. There are three main mouse LTR retrotransposon classes (reviewed in[76]): ERV1 (Class I), ERVK (Class II), and ERVL (Class III), which comprise 0.7%, 3.1% , and 5.5% of the mouse genome, respectively. Analysis of LTR retrotransposons included inspection of nucleotide substitution rates of hundreds of different repeat subfamilies from ERV1, ERVK, ERVL classes (Supplementary Data 1-3), from which we selected for further analysis a representative set of LTR retrotransposons of various age and type (Extended Data Fig. 2a).

We observed distinct divergent evolution of LTR retrotransposons in golden hamster and mouse genomes since their last common ancestor characterized by presence of young ERVK and absence of young ERVL retrotransposons. The ERVK class appeared as the most recently expanded

retrotransposon class when considering nucleotide substitution rate in retrotransposons insertions (Supplementary Data 1 and Extended Data Fig. 2a).

The ERVK class includes Intracisternal A Particle (IAP) retrotransposon, the currently only active autonomous LTR retrotransposon in the mouse genome[77]. IAP retrotransposons evolved in hamsters and mice from an ancestral retrovirus[29,30]. A notable expansion during the hamster genome evolution was observed among ERVK family retrotransposons, exemplified by MYSERV and IAP elements (Supplementary Data 1 and Extended Data Fig. 2c). Within the IAP retrotransposon family, which exhibited the lowest substitution rate, IAPLTR3 and IAPLTR4 subfamilies of IAP sequences exhibited minimal nucleotide exchange rates (Extended Data Fig. 2c) suggesting very recent or ongoing retrotransposition. Indeed, we identified 110 full-length intact IAP insertions in the golden hamster genome, which were classified as IAPLTR3/4 (Supplementary Data 4) while the IAPE subgroup is active in mice[31]. Remarkably, IAP-derived piRNAs were the second most abundant TE-targeting piRNAs after MYSERV-derived piRNAs at 9 dpp (Fig. 1c and Extended Data Fig. 2a). Consistent with that, we observed high abundances of pre-pachytene and pachytene piRNAs antisense to full-length intact IAP (Fig. 1d).

The ERVL class is an assorted group of elements, which includes the Mouse Endogenous Retrovirus type-L (MuERV-L). MuERV-L amplified in the mouse genome recently but it no longer retrotransposes despite it is transcriptionally activated during the mouse zygotic genome activation[78,79]. Non-autonomous Mammalian apparent LTR Retrotransposons (MaLRs), which expanded in the mouse genome despite they lack any protein-coding capacity[80], are ERVL class, which is sometimes classified as a separate ERVL-MaLR group. Notably, there was no support for ongoing or recent expansion of autonomous ERVL elements in the hamster genome (Supplementary Data 2 and Extended Data Fig. 2a). This was consistent with higher substitution rates of MaLR family of non-autonomous LTR elements, whose mobility requires functional autonomous ERVL elements (Supplementary Data 3 and Extended Data Fig. 2b). This contrasts with evolution of the mouse genome, where two distinct amplification bursts of MuERV-L retrotransposon occurred around 10 and 2 MYA[78]. MuERV-L expansion in the mouse genome presumably mobilized MaLR and gave rise to large MTA and ORR1A subfamilies, which have low substitution rates in the mouse genome but are absent in the golden hamster genome[27].

Taken together, *M. auratus* and *M. musculus* genomes carry full-length intact copies of L1 and IAP retrotransposons but these retrotransposon populations represent independent retrotransposon expansion events. Full-length intact L1 load in the hamster genome is reminiscent of that in the human genome and ten times lower than that in the mouse genome. The hamster genome was not subjected to a recent ERVL class expansion, which is observed in the mouse genome but exhibits some recent mobilization of the ERV1 class elements MuLV and LTRIS (Extended Data Fig. 2a).

Supplementary References

74 Gan, H. *et al.* piRNA profiling during specific stages of mouse spermatogenesis. *RNA* **17**, 1191-1203, doi:10.1261/rna.2648411 (2011).

75 Ono, M., Toh, H., Miyata, T. & Awaya, T. Nucleotide sequence of the Syrian hamster intracisternal A-particle gene: close evolutionary relationship of type A particle gene to types B and D oncovirus genes. *Journal of virology* **55**, 387-394 (1985).

76 Mager, D. L. & Stoye, J. P. Mammalian Endogenous Retroviruses. *Microbiol Spectr* **3**, MDNA3-0009-2014, doi:10.1128/microbiolspec.MDNA3-0009-2014 (2015).

77 Kuff, E. L. & Lueders, K. K. The intracisternal A-particle gene family: structure and functional aspects. *Adv Cancer Res* **51**, 183-276 (1988).

78 Costas, J. Molecular characterization of the recent intragenomic spread of the murine endogenous retrovirus MuERV-L. *Journal of molecular evolution* **56**, 181-186, doi:10.1007/s00239-002-2392-3 (2003).

79 Kigami, D., Minami, N., Takayama, H. & Imai, H. MuERV-L is one of the earliest transcribed genes in mouse one-cell embryos. *Biol Reprod* **68**, 651-654 (2003).

80 Smit, A. F. Identification of a new, abundant superfamily of mammalian LTR-transposons. *Nucleic Acids Res* **21**, 1863-1872 (1993).

Supplementary Data 1 to 3

All boxplots in Supplementary Data 1-3 were constructed from 200 randomly selected insertion sequences (or all insertions if there was less than 200 of them). The internal line in the boxplot represents median and bounds of box the 1st and the 3rd quartile; whiskers extends to values no lower (minima) or higher (maxima) than 1.5 * IQR (interquartile range).

Supplementary Data 1 ERVK LTR retrotransposon nucleotide exchange rates

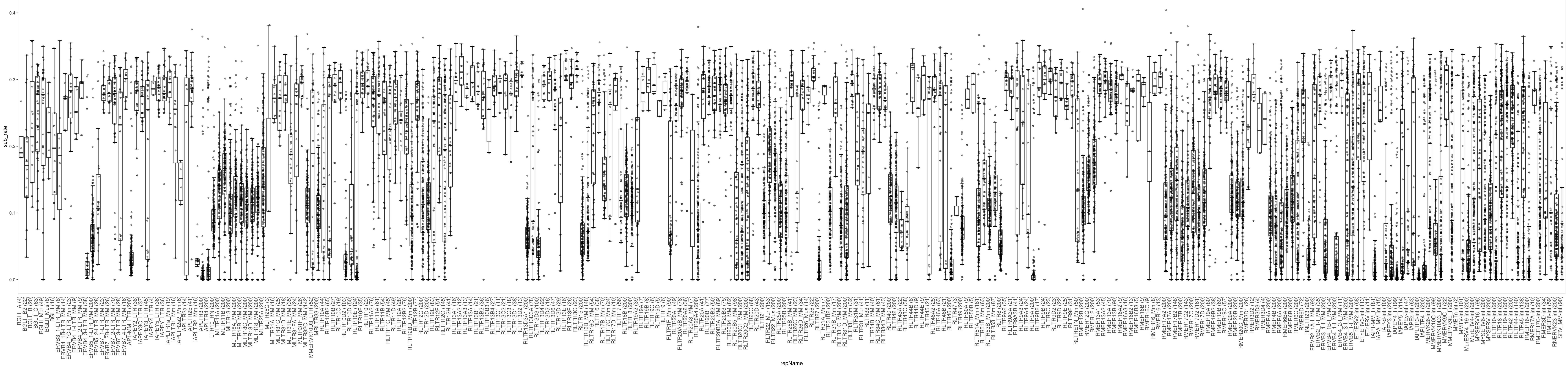

Supplementary Data 2 Other LTR retrotransposon nucleotide exchange rates

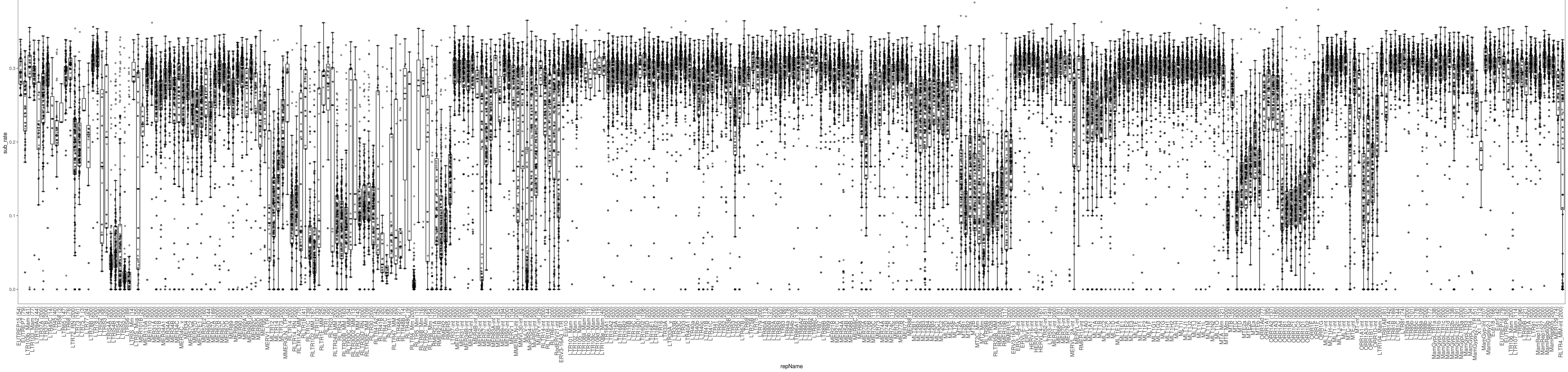

Supplementary Data 3 MaLR LTR retrotransposon nucleotide exchange rates

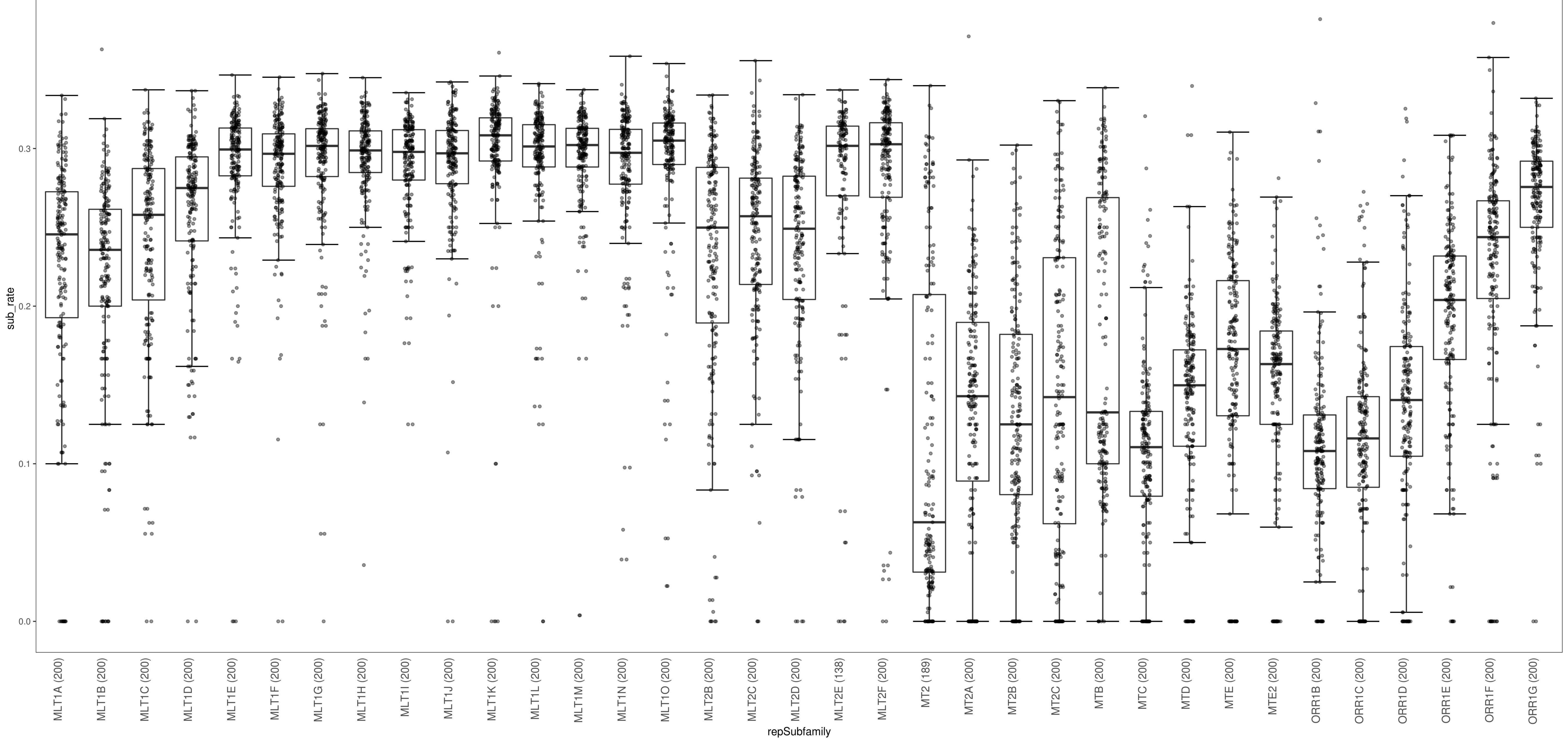

Supplementary Data 4 Golden hamster IAP full-length intact insertions

Full-length intact IAP retrotransposon inserts in FASTA format from hamster genome from [11].

>IAPLTR3.87762
[illegible]

>IAPLTR4_I.2701267
[illegible]

>IAPLTR4_I.59469
[illegible]

>IAPLTR3.125453
[illegible]

>IAPLTR3.2099368
[illegible]

[illegible]
TGCTGGCGAGACGGTAGCGCGGGACA

>IAPLTR3.4387567
[illegible]

>IAPLTR3.3712930
[illegible]
TCGTTCTTGCTGGCGAGACGGTAGCGCGGGACA

>IAPLTR4_I.1259142
[illegible]
GTATTAAAGCTTTACTGCAGAAGGATCCGAGTGTTCTGCGTCGTTTCTTGCTGGCGAGACGGTAGCGCGGGACA

>IAPLTR3.3199641
[illegible]

[illegible]
GATGCATTAAAGCTTTACTGCAGAAGGATCCGAGTGTTCTGCGTCGTTCTTGCTGGCGAGACGGTAGCGCGGGACA

>IAPLTR3.2073768
[illegible]
CTTGCTGGCGAGACGGTAGCGCGGGACA

>IAPLTR3.2099427
[illegible]
TACTGCAGAAGGATCTGAGTGTCCTGCGTCGTTCTTGCTGGCGAGACGGTAGCGTGGGACA

>IAPLTR3.455313
[illegible]
TTAAAGCTTTACTGCAGAAGGATCCGAGTGTTCTGCGTCGTTCTTGCTGGCGAGACGGTAGCGCGGGACA

>IAPLTR3.81900
[illegible]

[illegible]
GATGTATTAAAGCTTTACTGCAGAAGGATCCGAGTGTCCTGCGTCGTTCTTGCTGGCGAGACGGTAGCACGGGACA

>IAPLTR3.346684
[illegible]

>IAPLTR3.3357177
[illegible]

>IAPLTR3.1484621
[illegible]

>IAPLTR3.1792292
[illegible]

>IAPLTR3.1854531

[illegible]

>IAPLTR3.3809979

[illegible]

>IAPLTR3.3391277

[illegible]

>IAPLTR3.3879340

[illegible]

>IAPLTR3.819544

[illegible]

[illegible]

>IAPLTR3.3205730

[illegible]

>IAPLTR3.3829132

[illegible]

>IAPLTR3.3411338

[illegible]

>IAPLTR3.1279508

[illegible]

[illegible]

>IAPLTR3.3678913

[illegible]

>IAPLTR3.3692177

[illegible]

>IAPLTR3.3240402

[illegible]

>IAPLTR3.1150860

[illegible]

[illegible]

>IAPLTR3.1153750
[illegible]

>IAPLTR3.2780524
[illegible]

>IAPLTR3.3710829
[illegible]

>IAPLTR3.2234991
[illegible]

>IAPLTR3.2632982

[illegible]
GTGTTCTGCGTCGTTCTTGCTGGCGAGACGGTAGCGCGGGACA

>IAPLTR3.3077464
[illegible]

>IAPLTR3.308373
[illegible]

>IAPLTR3.3963398
[illegible]

>IAPLTR3.908452
[illegible]

CAGGTCAGTCCGGGATGTAAAAGGGAGGCTTCTGCCCCCTATTGAAGAACCCAAGGTACCCCAATCAAAAAACGGGTCACGGGGCCCATGGTCCCAGGGCCCTCAGAAATATGGGAACCAATTTCAGAAAAGCAATCAAGAGGAAGTAGAGGTTTCCGAGGAACCCCCGGAATGGACTTGCGTGCCGCCTCCGACTTCTTACTCATGCCCCAAATGAATGTTCAAC
CGGTCCCAGTCCATTCCCCTGGACCTTTGCCCCCTGCCACCATTGGGCTTATTCTGGGCCGTGGCTCCTTAACTTTACAGGGACTCATTATATATCCTGGGATTGTTGACCCGTATCATAAAGAGGAGATTCAAGTTCTCTGTTCTAACCCTCGGGGTGTATTTTCTATAAAGCAGGGAGATAGAATTGCACAACTGGTGTTGCTGCCCTCCCTCGGGGATGGAGA
AACCTACACTTTACAAAAAGGGCTATGGGATCTTCTGGAAGTGATTCAGCCTATCTGGCTATACCCCTAAATGAGAGACCTACAATAAAATTGTTAATTAATGGAAAAGAATTTGAAGGGATAATGGATACGGGAGCAGACAAAAGCATTATTTCACTCCATTGGTGGCCGAAGTCTTGGCCCACTGTTGTTTCATCTCATTCTCTACAAGGTCTTGGATACCAG
TCCTCTCCTGCCATCAGTGCCTCAGCCTTAACCTGGCGGAATGCTGAGGGCAAACAGGGATGTTTTACCCCCTATGTGTTGCCACTCCCTGTAAATTTATGGGGACGAGATGTGTTACAAGCCATGGGCATGACCCTAACTAATGAATACTCCCCCCAGGCATCAGCCATTATGGCAAAGATGGGGTATACAAAGGGTAGAGGCTTGGGTAGGCAAGAACAAGGCA
GAATAACACCCATTACGCAACACGGAAATCGGGGTAGAAAAGGACTGGGTTTTATTTAGGGGCCGTTGAGGCTTCACGACCCATACCATGGAAAACAGAGGAGCCGTTATGGGTCTCTCAATGGCCTCTATCCTCTGAAAAATTAGAGGCTGTCACAAGATTAGTGCAAGAACAGGAACGGCTGGGGCATCTAGAGCCTTCTACCTCCCCATGGAATACACCAATT
TTTGTTATTAAGAAGAAATCTGGGAAGTGGAGATTACTCCATGACCTGCGGGCCATTAACAATCAGATGCATCTTTTTGGCCCTGTTCAAAGAGGCCTTCCTTTGCTTTCTGCACTTCCTCAAGATTGGAAGCTTATTATTATAGATATTAAGGATTGTTTCTTCTCTATTCCACTTTACCCACGGGATAGACCAAGGTTTGCCTTCACTATCCCTTCTCTTAATC
ATATGGAACCAGACAAGAGATTTCAGTGGAAGGTACTGCCGCAAGGCATGGCCAATAGCCCAACAATATGTCAGCTGTATGTGCAGGAGGCTTTGGAGCCAATTAGGAAGCAATTTACATCTTTAATCGTTATCCATTATATGGACGATATTCTTATTTGTCACAAAGAGTTAGATGTTTTACAAAGGCTTTCCCCATGCTGGTGGCTGAATTAAAACAGTGGGG
ATTAGAGATTGCCTCAGAAAAGGTTCAAATTGCAGATACAGGCCTCTTTTTGGGCTCAATGATTACTCCCAAAAATATAGTTCCTCAGAAAATAGAAATTCGCAAAGACCACTTACAAACATTAAATGATTTTCAGAAACTTCTGGGAGATATAAATTGGTTGAGGCCCTTTTTAAAAATTCCATCTGCTGATCTAAAACCCCTCTTCGATCTCCTGGAGGGTGAG
CCTCATATCTCCTCACCCAGAAAATTTACCCCTGCCGCCCACCGAGCCTTACAGATGGTGGAAGAGGCCTTACAGGAAGCACAATTACAACGTATTGAGGAGTCACTACCTTTTAATCTGTGTGTTTTTAAGACAGCTCAATTGCCAACTGCAGTCCTGTGGCAACATGGACCATTACTTTGGATTCATCCAAATGCTTCCCCTGCTAAGATTATTGACTGGTACC
CTGATGCTGTTGCACAGCTCGCGCTTCGCGGAATAAAAGCAGCTGTTACACATTTTGGCAGGGATCCTGATTCCTTGATTGTGCCTTATACTGCTGCACAAGTTCAGACCTTGGCAGCCACATCTAGTGATTGGGCAGTTTTAGTTACTTCCTTTTCAGGACAAATTGATAATCATTTTCCAAAGCATCCAATTTTACAGTTTGCCCTAAACCAGGCTATAGTGTT
TCCACAAGTGACAGCTAAAGACCCACTCCCAGATGGGACTGTGGTGTACACTGATGGATCAAAAACTGGTATGGGAGCTTATGTGGTCAAGGATAGGGTTATATCTAAACAATATAATGAAACTTCACCTCAAGTTGTAGAATGTCTGATAGTACTGGAGGTTCTTGAGGCTTTTCCAGGACCCCTCAATATTGTATCAGATTCCTCTTATGTGGTTAATGCAGTT
AATCTCCTTGAGATAGCTGGAATAATAAGATCCTCCAGCAGAGTTGCTAACATCTTTCAAAAGATACAAGCTGCCTTGTTAAATAGGAGATTTCCTGTTTTCATTACCCATGTCCGAGCACATTCTGGACTCCCAGGTCCCATGTCCTTAGGGAATGACTTAGCGGACAAGGCTACAAAGCTGGTGGCCGCTGCCTTGTCTACCCATGCACAAGCAGCAAAAGAAT
TCCATAAGCGCTTTCATGTGACGGCTGAGACCTTGCGCCGTCGTTTTGCTCTATCCAGGAAAGAGGCTAGGGAAATTGTTACTCAATGCCAGAATTGCTGCGAATTCTTACCTACTCCCCACATGGGAATAAACCCACGTGGCATTAGACCATTGCAGATGTGGCAGATGGATGTTACTCATATTCCCTCTTTTGGAAGGCTTCAATATGTTCATGTTTCTGTTGA
CACCTGTTCTGGCGTAATGTTTGCCACACCCCTAACAGGGGAAAAGGCCTCATATGTGATTCAACATTGCCTTGAGGCCTGGAGTGCTTGGGGAAAACCCAGAATTTTAAAAACAGACAATGGACCAGCATATACCTCTCAAAAATTCCGACAGTTTTGTCGGCAAATGGATGTTACCCATCTAACTGGCCTACCTTATAACCCTCAAGGACAGGGCATTGTGGAA
CATGCCCATCGTACACTCAAATCATATCTGATTAAACAAAAGGGGAGTATTGAGGATGTTCTACCCTCGGTACCAAGAGTCGCTGTATCCATGGCACTTTTTACCCTTAATTTCTTGAACTCTGATGCCCAAGGCCACACTGCGGCCGATCGCCACAGTTTAGAGCCTGATAGGCCAAAAGAAATGGTAAAATGGAAAGATGTCTTGACTGATCTCTGGAAAGGCC
CGGATCCTATTCTTATAAGATCCAGGGGAGCTGTGTGTGTTTTTCCACAGGAGGAAGAAAACCCCCTGTGGATCCCTGAAAGACTCACTCGAAGAGCTCCTTCACAACTCCAGAATAAGAAGGATGGAGGCCAAGTGGGTGATGAAGACTCTCCTACTCGGGATGATGGTTCCTAGTATGATGGGAGAGTTACGGTGGGCCATCATGTCTACCTTCCCCCCTACCGA
TGCCAGTGATGCAGAATGCCCAGGTGTTCCCTAGATTTTTCACAACTAATAGAGAGTTGGGGCTTGCCTTTTTGCCTTTAGATCCTCAGATACAGAGTTTTCAGGATGAGAGAACCTTGTCATTAGTAGGAAGCTTGTGCTTTACTGTGATTTCTTCATTAAAGGATAATTTTATTCATATGGATCCAGAAAGATCTAATGCTAATCCTTTTGAATTAATGGAAGC
TGTACTGATCTTTCTCCTATGATAGTGAGAAGGGACAGATTACTTTTGATTGGAAAGGGGCATTAGGCAACAGTCTGAGTGAGGGACAGAGCTATGGATCATCTGATTGGTCCTCCTCCAATGTTAAGTATACCCCTTTTAAGAGAACTAATTATTCAGCTGCCCTTTATTTGGGTTGTGAGTAATGATACCTTAGGTAAGAATCAGAAAGAAATAAATTGCTCTG
CTTAGAATTGTTTTTATGCATTATGCTGGGATGCTATTAAGCACCCTTTTGCTTTGGTAATGCGCATGCCTCGATTTGTTCCAGTTCCTGTTGAGACGCCAAGTAATTTGAGTTTGTTTAGGGAAAAGCGAGATTTTTGGGATCTCTGCTATTATTGTGGGCCTGATAGCCACCGCCGCGGTTACTGCTTCGGTTACCGCCTCGGCCCTTGCACTTTCTGCCACTG
TGCAGACAACTCAAACCATTAATGAACTATAGGCCACTGTGACCATGGTCCTAGATAAACAGGCCACTGCCAATTCTCAGATACAAGGAGGCCTGATGTTGGTAAACCAACAGATTAATTTGGTTCAGGAACAATTGGATATATTGTGGCAGATGGCCCAATTGGGATGTGAACAAAAGTTGCCTGGCCTTTGTGTCACTTCAGTACAATATGAGAATTTTACAAG
AGCAGCTAACCTGTCAAAAGCTCTGTCTCAACATTTATTACAGAATTGGACTTTTGAGTTTGAGCAGACACTCCGTGAGTTGAGAATGGCGATCCTGCAAGTGAATTCGACGCATCTGGATCTTTCTCTGATGGAAGGCCTCTCCTCCTGGATTTCATCTGCATTTCTTATTTTAAGGAATGGGTGGGGGTAGGATCATTTAGCCTAGTTCTCTGCTGTGGAATA
GTGTTGATGCTTTGGATGGTGTGTAAGCTCAGAGCCCAAACAAAAGAGACAAAGTGGTGGTGGCACAAGCACTTGCTGCCTTAGAACACGGAGCCTCCCCTGACATTTGGTTAACCATGTTAAAGCAATAGGTCGCTGGCCTCCCAGCTCTTGCACCCCACGAGGCTAGTCTCATTGCACGGGATAGAGTGGGTAGGCTTCAGCAGCCCAAGGAGTTGCAAGGCTA
AGCACTGCACAGGAGAGGTCTGCGGTATAACGACTTTCTCCTGGGAGATAAGTCATCTTGCATGAAGGTTCTATGTCATGATCTCCTTCCCCCAGAAAAACGACATCGGACTGGTCAGGGCTCTCTCTGGGGATAAGACCCTGGATAGGAGCTTTGTACATGTTCCGAATTTGCACATGGGGATTTGACCTCTATCTCCACTCCAAAGTTGTGGGTGTCCTATTGC
TTCAAAAATAAATGAATAGGGGGAGATGTGAGGAGCCGCCCTCGCTATCGCTATTGCCGTTAGAAGATGGCGCTGACATCTGCTGTCAGTTGGAGTTAACCGTTAGAAGATGGCGCTGACATCTGCTGTCAGTTGGAGTTAACCGTTTAAGCTGTGCCTCTCCCGTGGCGTCATCTGGGGTGATGTGCAAACCACCAATCCCGGCTCTACACGTCTCACTCGGAGC
TCCTAGGCTTATATAAGGGGCTGGGTTTCTTAGCTTGGGGTCTCCCTCTAAGAAGCTGATCATCTATCTCTCAAGATGCATTAAAGCTTTACTGCAGAAGGATCCGAGTGTTCTGCGTCGTTCTTGCTGGCGAGACGGTAGCGCGGGACA

>IAPLTR3.1679467
GTGAGGAGCCGCCCTCGCTATCGCTATTGCCGTTAGAAGATGGCGCTGACATCAGCTGTCAGTTGGAGTTAACCGTTAGAAGATGGCGCTGACATCTGCTGTCAGTTGGAGTTAACCGTTTAAGCTGTGCCTCTCCCGTGGCGTCATCTGGGGTGATGTGCAAACCACCAATCCCGGCTCTACACGTCTCACTCGGAGCTCCTAGGCTTATATAAGGGGCTGGGTT
TCTTAGCTTGGGGTCTCCCTCTAAGAAGCTGATCATCTATCTCTCAAGATGCATTAAAGCTTTACTGCAGAAGGATCCGAGTGTTCTGCGTCGTTCTTGCTGGTGAGACGGTAGCGCGGGACATCTGGTGCCGAAACCCGGGAACTCTTCAACATCGCCGGCGCCGCGGGAGACCCCTCGGAAGACGGGGCGGATTCAGAACTGCAGGGACGTAAGTTCAGAGAGG
TATGCTTTATCCTGAATACCCCCTTTCTCGACTTTGGCCTTAGTTTGTCATCACCGTGGGACGGGTCAGTGGAGGCTTTGGTGCTAGTCCTATTTGTCCTTCTCCTTCATCGTGTCTGGTGCTGTCAGTCTGGTGCTGTCAGTCTGGTGCTGTCAGTCTGGTGCTGTCAGTCTGGTGCTGTCAGTCTGGTGCTGTCAGTCTGGTGCTGTCAGTCTGGTGCTGTCAG
TCTGGTGCTGTCAGTCTGGTAACGGAGTTGAGCGCCGTTTTTTAGATAAGCCGCTAGCGCGATGGGGTTGTCACAGTCAGTGGTTACTGCCCTACAGACTGTGCTAAAACAAAGAGATCTTAAAATTGCACCACGAACACTTCAAAATTTTGTGAAGGAGGTAGATAGAGTGGCACCATGGTATGCTTGTTCCGGGTCCCTCACTGTGGCCTCATGGAACAAGCTA
GGTAAAGAACTAGACAGGAAGCATGCAGAAGGAGATCTCCACTTAGGTACCAAAGCCATTTGGAAGCTAGTTAAAACTGCTTGGAGGATGAGGCCTGTCACCCTGCTATAATAGAAAGTCAGGGAACCTTAGAGGAAGTTCAGGATAGCATGTCAGAAACTGAACGGAGTGAGAGAATGGGAGCTCGCAAAAGGAAAGACATGTCTAAGAAAAAGGCCCTCCCC
AAGAAGTTAAGAAAGGGGGAGAGAAAGAAGGGAGTGATCATTCACCCCCTAACAAATCTAAGAAAAAGAAAAAGCCAGAATCTAGTCTATACCCTACAGTAGAGCTAGAAGCCTTGGAGCTAGATAATTCCGATTCTGACACTCTGTATTCTAGCGAAGAAGGTGGTCTAGAGGAGGAGGTGGCACGGTATGAGGAAGAAAGATATCACCCTGATAGACACCGGCC
ACTGAAAACAAAAATGAATGTGAGGCCACCGCCTATAAATCCGGCGGGCTCACGCCCCTCAGCGCCTCCGCAGTATGAGCTTCGGCTGAATAATGGTACTGACTCCTTTTTACCTTTAGAGGAATGGAGAAAAATACAAGTAGCTTTCCAGTATTCGAGAATGCAGAAGGCGGAAGAGTACATACCCCTGTAGATTACAATCAATCAAAGAATTGGCCGAATCC
GTTCGTAATTATGGGGTCAATGCTAACTTTACCACATTACAGGTGGAAAGGCTTGCTAATTTTGCCATGACTCCCACTGACTGGCAGACGACAGTGAAAGCAGTGCTTCCCAACATGGGACAATACATGGAATGGAAAGCTCTCTGGTATGATGCAGCCCAGGCACAAGCTAGAGTTAATGCCACGGCTGAAGATGAGAATCAGAGGCAGTGGACATTTGAAATGC
TGACAGGTCAGGGTCAACATGCCCTTAATCAAACCAACTACATCTGGGGTGTCTATGGTCAGGTGTCAGCCGCCGCTATTAAAGCATGGAAGGCCCTCACAAAAAGGGGAGAGTCCAGTGGACACCTTACAAAGATTGTCCAAGGACCCCAAGAGCCATTTTCAGACTTTGTGGCCAGAGTGACAGAGGCCGCCAGTCGCATCTTTGGTGATTCAGAACAAGCTAT
GCCCCTGATTGAGCAACTTGTCTATGAACAAGCAACCCAGGAATGTCGAGCGGCTATCGCCCCTCGCAAAAGCAAAGGTTTACAAGATTGGCTCAGAGTGTGCAGGGAGCTGGGGGGACCCCTAACCAATGCAGGACTAGCTGCCGCCATTTTACAAACGCACAGGCATAGAGATTTATCTAATCGGAAAGCTTGTTTTAATTGTGGGAGAATGGGCACCTTAAG
AAAGATTGTCAAGCCCCTGAAAGAACAAGAGAATCGAAGCTTTGCCATCGTTGTGGTAAAGGTTATCATAGGGCTAGCGAATGCAGGTCAGTCCGGGATGTAAAGGGAGGCTTCTGCCCCCTATTGAAGAACCCAAGGTACCCCAATCAAAAAACGGGTCACGGGGCCCATGGTCCCAGGGCCCTCAGAAATATGGGAACCAATTTCAGAAAAGCAATCAGAGG
AAGTAGAGGTTTCCGAGGAACCCCCGGAATGGACTTGCGTGCCGCCTCCGACTTCTTACTCATGCCCCAAATGAATGTTCAACCGGTCCCAGTCCATTCCCCTGGACCTTTGCCCCCTGCCACCATTGGGCTTATTCTGGGCCGTGGCTCCTTAACTTTACAGGGACTCATTATATATCCTGGAATTGTTGACCCGTATCATAAAGAGGAGATTCAAGTTCTCTGT
TCTAGCCCTCGGGGTGTATTTTCTATAAAGCAGGGAGATAGAATTGCACAACTGGTGTTGCTGCCCTCCCTCGGGGATGGAGAAACCTACACTTTACAAAAAAGGGCTATGGGATCTTCTGGAAGTGATTCAGCCTATCTGGCTATACCCCTAAATGAGAGACCTACAATAAAATTGTTAATTAATGGAAAAGAATTTGAAGGGATAATGGATACGGGAGCAGACA
AAAGCATTATTTCACTCCATTGGTGGCCGAAGTCTTGGCCCACTGTTGTTTCATCTCATTCTCTACAAGGTCTTGGATACCAGTCCTCTCCTGCCATCAGTGCCTCAGCCTTAACCTGGCGGAATGCTGAGGGCAAACAGGGATGTTTTACCCCCTATGTGTTGCCACTCCCTGTAAATTTATGGGGACGGATGTGTTACAAGCCATGGGCATGACCCTAACTAA
TGAATACTCCCCCCAGGCATCAGCCATTATGGCAAAGATGGGGTATACAAAGGGTAGAGGCTTGGGTAGGCAAGAACAAGGCAGAATAAAACCCATTATGCAACACGGAAATCGGGGTAGAAAAGGACTGGGTTTTATTTAGGGGCCGTTGAGGCTTCACGACCCATACCATGGAAAACAGAGGAGCCGTTATGGGTCTCTCAATGGCCTCTATCCTCTGAAAAAT
TAGAGGCTGTCACAAGATTAGTGCAAGAACAGGAACGGCTGGGGCATCTAGAGCCTTCTACCTCCCCATGGAATACACCAATTTTTGTTATTAAGAAGGAATCTGGGAAGTGGAGATTACTCCATGACCTGCGGGCCATTAGCAATCAGATGCATCTTTTTGGCCCTGTTCAAAGAGGCCTTCCTTTGCTTTCTGCACTTCCTCAAGATTGGAAGCTTATTATTAT
AGATATTAAGGATTGTTTCTTCTCTATTCCACTTTACCCACGGGATAGACCAAGGTTTGCCTTCACTATCCCTTCTCTTAATCATATGGAACCAGACAAGAGATTTCAGTGGAAGGTACTGCCACAAGGCATGGCCAATAGCCCAACAATATGTCAGCTGTATGTGCAGGAGGCTTTGGAGCCAATTAGGAAGCAATTTACATCTTTAATCGTTATCCATTATATG
GACGATATTCTTATTTGTCACAAAGAGTTAGATGTTTTACAAAGGCTTTCCCCATGCTGGTGGCTGAATTAAAACAGTGGGGATTAGAGATTGCCTCAGAAAAGGTTCAAATTGCAGATACAGGGCTCTTTTTGGGCTCAATGATTACTCCCAAAAATATAGTTCCTCAGAAAATAGAAATTCGCAAAGACCACTTACAAACATTAAATGATTTTCAGAAACTTC
TGGGAGATATAAATTGGTTGAGGCCCTTTTTAAAAATTCCATCTGCTGATCTAAAACCCCTCTTCGATCTCCTGGAGGGTGAGCCTCATATCTCCTCACCCAGAAAATTTACCCCTGCCGCCCACCGAGCCTTACAGATGGTGGAAGAGGCCTTACAGGAAGCACAATTACAACGTATTGAGGAGTCACTACCTTTTAATCTGTGTGTTTTTAAGACAGCTCAATT
GCCAACTGCAGTCCTGTGGCAACATGGACCATTACTTTGGATTCATCCAAATGCTTCCCCTGCTAAGATTATTGACTGGTACCCTGATGCTGTTGCACAGCTCGCGCTTCGCGGAATAAAAGCAGCTGTTACACATTTTGGCAGGGATCCTGATTCCTTGATTGTGCCTTATACTGCTGCACAAGTTCAGACCTTGGCAGCAACATCTAGTGATTGGGCAGTTTTA
GTTACTTCCTTTTCAGGACAAATTGATAATCATTTTCCAGAGCATCCAATTTTACAGTTTGCCCTAAACCAGGCTATAGTGTTTCCACAAGTGACAGCTAAAGACCCACTCCCAGATGGGACTGTGGTGTACACTGATGGATCAAAAACTGGTATGGGAGCTTATGTGGTCAAGGATAGGGTTATATCTAAACAATATAATGAAACTTCACCTCAAGTTGTAGAAT
GTCTGATAGTACTGGAGGTTCTTGAGGCTTTTCCAGGACCCCTCAATATTGTATCAGATTCCTCTTATGTGGTTAATGCAGTTAATCTCCTTGAGATAGCTGGAATAATAAGATCCTCCAGCAGAGTTGCTAACATCTTTCAAAAGATACAAGCTGCCTTGTTAAATAGGAGATTTCCTGTTTTCATTACCCATGTCCGAGCACATTCTGGACTCCCAGGTCCCAT
GTCCTTAGGGAATGACTTAGCGGACAAGGCTACAAAGCTGGTGGCCGCTGCCTTGTCTACCCATGCACAAGCAGCAAAAGAATTCTATAAGCGCTTTCATGTGACGGCTGAGACCTTGCGCCGTCGTTTTGCTCTATCCAGGAAAGAGGCTAGGGAAATTGTTACTCAATGCCAGAATTGCTGCGAATTCTTACCTACTCCCCACATGGGAATAAACCCACGTGGC
ATTAGACCATTGCAGATGTGGCAGATGGATGTTACTCATATTCCCTCTTTTAGAAGGCTTCAATATGTTCATGTTTCTGTTGACACCTGTTCTGGCGTAATGTTCGCCACACCCCTAACAGGGGAAAAGGCCTCATATGTGATTCAACATTGCCTTGAGGCCTGGAGTGCTTGGGGAAAACCCAGAATTTTAAAAACAGACAATGGACCAGCATATACCTCTCAAA
AATTCCGACAGTTTTGTCGGCAAATGGATGTTACCCATCTAACTGGCCTACCTTATAACCCTCAAGGACAGGGCATTGTGGAACGTGCCCATCGTACACTCAAATCATATCTGATTAAACAAAAGGGGAGTATTGAGGATGTTCTACCCTCGGTACCAAGAGTTGCTGTATCCATGGCACTTTTTACCCTTAATTTCTTGAACTCTGATGCCCAAGGCCACACTGC
GGCCGATCGCCACAGTTTAGAGCCTGATAGGCCAAAAGAAATTGTAAAATGGAAAGATGTGCTAACTAATGAATGGAAAGGCCCGGATCCTATTCTTATAAGATCCAGGGGAGCTGTGTGTGTTTTTCCACAGGAGGAAGAAAACCCCCTGTGGATCCCTGAAAGACTCACTCGAAGAGCTCCTTCACAACTCCAGAATAAGAAGGATGGAGGCCAAGTGGGTGAT
GAAGACTCTCCTACTCGGGATGATGGTTCCTAGTATGATGGGAGAGTTACGGTGGGCCATCATGTCTACCTTCCCCCCTACCGATGCCAGTGATGCAGAATGCCCAGGTGTTCCCTAGATTTTTCACAACTAATAGAGAGTTGGGGCTTGCCTTTTTGCCTTTAGATCCTCAGATACAGAGTTTTCAGGATGAGAGAACCTTGTCATTAGTAGGAAGCTTGTGCTTT
ACTGTGATTTCTTCATTAAAGGATAATTTTATTCATATGGATCCAGAAAGATCTAATGCTAATCCTTTTGAATTAATGGAAGCTGTACTGATCTTTCTCCTATGATAGTGAGAAGGGACAGATTACTTTTGATTGGAAAGGGGCATTAGGCAACAGTCTGAGTGAGGGACAGAGCTATGGATCATCTGATTGGTCCTCCTCCAATGTTAAGTATACCCCTTTTAAG
AGAACTAATTATTCAGCTGCCCTTTATTTGGGTTGTGAGTAATGATACCTTAGGTAAGAATCAGAAAGAAATAAATTGCTCTGCTTAGAATTGTTTTTATGCATTATGCTGGGATGCTATTAAGCACCCTTTTGCTTTGGTAATGCGCATGCCTCGATTTGTTCCAGTTCCTGTTGAGACGCCAAGTAATTTGAGTTTGTTTAGGGAAAAGCGAGATTTTTGGGAT
CTCTGCTATTATTGTGGGCCTGATAGCCACCGCCGCGGTTACTGCTTCGGTTACCGCCTCGGCCCTTGCACTTTCTGCCACTGTGCAGACAACTCAAACCATTAATGAACTATAGGCCACTGTGACCATGGTCCTAGATAAACAGGCCACTGCCAATTCTCAGATACAAGGAGGCCTGATGTTGGTAAACCAACAGATTAATTTGGTTCAGGAACAATTGGATATA
TTGTGGCAGATGGCCCAATTGGGATGTGAACAAAAGTTGCCTGGCCTTTGTGTCACTTCAGTACAATATGAGAATTTTACAAGAGCAGCTAACCTGTCACAAGCTCTGCCTCAACATTTATTACAGAATTGGACTTTTGAGTTTGAGCAGACACTCCGTGAGTTGAGAATGGCGATCCTGCAAGTGAATTCGACGCATCTGGATCTTTCTCTGATGGAAGGCCTCT
CCTCCTGGATTTCATCTGCATTTTCTTATTTTAAGGAATGGGTGGGGGTAGGATCATTTAGCCTAGTTCTCTGCTGTGGAATAGTGTTGATGCTTTGGATGGTGTGTAAGCTCAGAGCCCAAACAAAAGAGACAAAGTGGTGGTGGCACAAGCACTTGCTGCCTTAGAACACGGAGCCTCCCCTGACATTTGGTTAACCATGTTAAAGCAATAGGTCGCTGGCCTC
CCAACTCTTGCACCCCACGAGGCTAGTCTCATTGCACGGGATAGAGTGGGTAGGCTTCAGCAGCCCAAGGAGTTGCAAGGCTAAGCACTGCACAGGAGAGGTCTGCGGTATAACGACTTTCTCCTGGGAGATAAGTCATCTTGCATGAAGGTTCTATGTCATGATCTCCTTCCCCCAGAAAAACGACATCGGACTGGTCAGGGCTCTCTCTGGGGATAAGACCCTG
GAGAGGAGCTTTGTACATGTTCCAAATTTGCACATGGGGATTTGACCTCTATCTCCACTCCAAAGTTGTGGGTGTCCTATTGCTTCAAAAATAAATGAATAGGGGGAGATGTGAGGAGCCGCCCTCGCTATCGCTATTGCCGTTAGAAGATGGCGTTGACATCTGCTGTCAGTTGGAGTTAACCGTTAGAAGATGGCGCTGACATCTGCTGTCAGTTGGAGTTAAC
CGTTTAAGCTGTGCCTCTCCCGTGGCGTCATCTGGGGTGATGTGCAAACCACCAATCCCGGCTCTACACGTCTCACTCGGAGCTCCTAGGCTTATATAAGGGGCTGGGTTTCTTAGCTTGGGGTCTCCCTCTAAGAAGCTGATCATCTATCTCTCAAGATGCATTAAAGCTTTACTGCAGAAGGATCCGAGTGTTCTGCGTCGTTCTTGCTGGCGAGACGGTAGCG
CGGGACA

>IAPLTR3.922462
GTGAGGAGCCGCCCTCGCTATCGCTATTGCCGTTAGAAGATGGCGCTGACATCTGCTGTCAGTTGGAGTTAACCGTTAGAAGATGGCGCTGACATCTGCTGTCAGTTGGAGTTAACCGTTTAAACTGTGCCTCTCCCGTGACGTCATCTGGGGTGATGTGCAAACCACCAATCCCGGCTCTACACATCTCACTCGGAGCTCCTAGGCTTATATAAGGGGCTGGGTT
TCTTAGCTTGGGGTCTCCCTCTAAGAAGCTGATCATCTATCTCTCAAGATGCATTAAAGCTTTACTGCAGAAGGATCCGAGTGTTCTGCGTCGTTCTTGCTGGCGAGACGGTAGCGCGGGACATCTGGTGCCGAAACCCGGGAACTCTTCAACATCGCCGGCGCCGCGGGAGACCCCTCGGAAGACGGGGCGGATTCAGAACTGCAGGGACGTAAGTTCAGAGAGG
TATGCTTTATCCTGAACACCCCCTTTCTCGACTTTGGCCTTAGTTTGTCATCACCGTGGGACGGGTCAGTGGAGGCTTTGGTGCTAGTCCTGTTTGTCCTTCTCCTTCATCGTGGCTGGTGCTGACAGAACGGCGCTGTCCATCTGGCGCTGTCCGTCTGGCGCTGTCCATCTGGCGCTGTCCATCTGGCGCTGTCAGTCTGGTGCTGTCAG
TCTGGTGCTGTCAGTCTGGTGCTGTCAGTCTGGTAACGGAGTTGAGCGCGTTTTTTAGATAAGCTGTCAGCGCGATGGGGTCGTCACAGTCAGTGGTTACAGCCTTACAGACTGTGCTAAAACAAAGAGATCTTAAAATTGCACCACGAACACTTCAAAATTTTGTGAAGGAGGTAGATAGAGTGGCACCATGGTATGCTTGTTCCGGGTCCCTCACTGTGGCCTC
ATGGAACAAGCTAGGTAAAGAACTAGACAGGAAGCATGCAGAAGGAGATCTCTGCTTAGGTACCAAAGCCATTTGGAAGCTAGTTAAAACTGCTTGGAGGATGAGGCCTGTCACCCTGCTATAATAGAAAGTCAGGGAACCTTAGAGGAAGTTCAGGATAGCATGTCAGAAACTGAACGGAGTGAGAGAATGGGAGCTCGCAAAAGGAAAGACATGTCTAAGAAA
AAAGGCCCTCCCCAGGAAGTTAAGAAAGGGGGAGAGAAAGAAGGGAGTTATCATTCACCCCTTAACAAATCTAAGAAAAAGAAAAAGCCAGAATCTAGTCTATACCCTGCAGTAGAGCTAGAAGCCTTGGAGCTAGATAATTCTGATTCTGACACTCTGTATTCTAGCGAAGAAGGTGGTCTAGAGGAGGAGGTGGCACGGTATGAGGAAGAAAGATATCACCCTG
ATAGACACCGGCCACTGAAAACAAAAATGAATGTGAGGCCACCGCCTATAAATCCGGCGGGCTCACGCCCCTCAGCGCCTCCGCCATATGAGCTTCGGCTGAATACTGGTACTGACTCCTTTTTACCTTTAGAGGAACGGAGAAAAATACAAGTAGCTTTCCAGTATTCGAGAATGCGGAAGGCGGAAGAGTACATACCCCTGTAGATTACAATCAATCGAAGA
ATTGGCCGAATCCGTTCGTAATTATGGGGTCAATGCTAACTTTACCACATTACAGGTGGAAAGGCTTGCTAATTTTGCCATGACTCCCACTGACTGGCAGACGACAGTGAAAGCAGTGCTTCCCAACATGGGACAATACATGGAATGGAAGGCTCTCTGGTATGATGCAGCCCAGGCACAAGCTAGAGTTAATGCCACGGCTGAAGATGAGAATCAGAGGCAGTGG
ACATTTGAAATGCTGACAGGTCAGGGTCAACATGCCCTTAATCAAACCAACTACATCTGGGGTGTCTATGGTCAGGTGTCGGCCGCCGCTATTAAAGCATGGAAGGCCCTCACAAAAAGGGGAGAGTCCAGTGGACACCTTACAAAGATTGTCCAAGGACCCCAAGAGCCATTTTCAGACTTTGTGGCCAGAATGACAGAGGCCGCCAGTCGCATCTTTGGTGATT
CAGAACAAGCTATGCCCCTGATTGAGCAACTTGTCTATGAACAAGCAACCCAGGAATGTCGAGTGGCTATCGCCCCTCGCAAAAGCAAAGGTTTACAAGATTGGCTCAGAGTGTGCAGGGAGCTGGGGGGACCCCTAACCAATGCAGGACTAGCTGCCGCCATTTTACAAACGCACAGGTATAGAGATTTATCTAGTCGAAAAGCTTGTTTTAATTGTGGGAGAAT
GGGGCACCTTAAGAAAGATTGTCAAGCCCCTGAAAGAACAAGAGAATCGAAGCTTTGCCATCGTTGTGGTAAAGGTTATCATAGGGCTAGCGAATGCAGGTCAGTCCGGGATGTAAAGGGAGGCTTCTGCCCCCTATTGAAGAACCCAAGGTACCCCAATCAAAAAACGGGTCACGGGGCCCATGGTCCCAGGGCCCTCAGAAATATGGGAACCAATTTCAGAAA
AGCAATCAAGAGGAAGTAGAGGCTTCCGAGGAACCCCCGGAATGGACTTGCGTGCCGCCTCCGACTTCTTACTCATGCCCCAAATGAATGTTCAACCGGTCCCAGTCCATTCCCCTGGACCTTTGCCCCCTGCCACCATTGGGCTTATTCTGGGCCGTGGCTCCTTAACTTTACAGGGACTCATTATATATCCTGGAATTGTTGACCCGTATCATAAAGAGGAGAT
TCAAGTTCTCTGTTCTAGCCCTCGGGGTGTATTTTCTATAAAGCAGGGAGATAGAATTGCACAACTGGTGTTGCTGCCCTCCCTCGGGGATGGAGAAACCTACACTTTACAAAAAAGGGCTATGGGATCTTCTGGAAGTGATTCAGCCTATCTGGCTATACCCCTAAATGAGAGACCTACAATAAAATTGTTAATTAATGGAAAAGAATTTGAAGGGATAATGGAT
TCGGGAGCAGACAAAAGCATTATTTCACTCCATTGGTGGCCGAAGTCTTGGCCCACTGTTGTTTCATCTCATTCTCTACAAGGTCTTGGATACCAGTCCTCTCCTGCCATCAGTGCCTCAGCCTTAACCTGGCGGGATGCTGAAGGCAAACAGGGATGTTTTACCCCCTATGTGTTGCCACTCCCTGTAAATTTATGGGGACGAGATGTGTTACAAGCCATGGGCA
TGACCCTAACTAATGAATACTCCCCCCAGGCATCAGCCATTATGGCAAAGATGGGGTATACAAATGGTAGAGGCTTGGGTAGGCAAGAACAAGGCAGAATAAAACCCATTACACAACACGGAAATCGGGGTAGAAAAGGACTGGGTTTTATTTAGGGGCCGTTGAGGCTTCACGACCCATACCATGGAAAACAGAGGAGCCGTTATGGGTCTCTCAATGGCCTCTA
TCCTCTGAAAAATTAGAGGCTGTCACAAGATTAGTGCAAGAACAGGAACGGCTGGGGCATCTAGAGCCTTCTACCTCCCCATGGAATACACCAATTTTTGTTATTAAGAAGAAATCTGGGAAGTGGAGATTACTCCACGACCTGCGGGCCATTAACAATCAGATGCATCTTTTTGGCCCTGTTCAAAGAGGCCTTCCTTTGCTTTCTGCACTTCCTCAAGTTGGA
AGCTTGTTATTATAGATATTAAGGATTGTTTCTTCTCTATTCCACTTTACCCACGGGATAGACCAAGGTTTGCCTTCACTATCCCTTCTCTTAATCATATGGAACCAGACAAGAGATTTCAGTGGAAGGTACTGCCGCAAGGCATGGCCAATAGCCCAACAATATGTCAGCTGTATGTGCAGGAGGCTTTGGAGCCAATTAGGAAGCAATTTACATCTTTAATCGT
TATCCATTATATGGACGATATTCTTATTTGTCACAAAGAGTTAGATGTTTTACAAAGGCTTTCCCCATGCTGGTGGCTGAATTAAAACAGTGGGGATTAGAGATTGCCTCAGAAAAGGTTCAAATTGCAGATACAGGCCTCTTTTTGGGCTCAAGATTACTCCCAAAAATATAGTTCCTCAGAAAATAGAAATTCGCAAAGACCACTTACAAACATTAAATGAT
TTTCAGAAACTTCTGGGAGATATAAATTGGTTGAGGCCCTTTTTAAAAATTCCATCTGCTGATCTAAAACCCCTCTTCCATCTCCTGGAGGGTGAGCCTCATATCTCCTCACCCAGAAAATTTACCCCTGCCGCCCACCGAGCCTTACAGATGGTGGAAGAGGCCTTACAGGAAGCACAATTACAACGTATTGAGGAGTCACTACCTTTTAATCTGTGTGTTTTTA
AGACAGCTCAATTGCCAACTGCAGTCCTGTGGCAACATGGACCATTACTTTGGATTCATCCAAATGCTTCCCCTGCTAAGATTATTGACTGGTACCCTGATGCTGTTGCACAGCTCGCGCTTCGCGGAATAAAAGCAGCTGTTACACATTTTGGCAGGGATCCTGATTCCTTGATTGTGCCTTATACTGCTGCACAAGTTCAGACCTTGGCAGCCACATCTAGTGA
TTGGGCAGTTTTAGTTACTTCCTTTTCAGGACAAATTGATAATCATTTTCCAAAGCATCCAATTTTACAGTTTGCCCTAAACCAGGCTATAGTGTTTCCACAAGTGACAGCTAAAGACCCACTCCCAGATGGGACTGTGGTGTACACTGATGGATCAAAAACTGGTATGGGAGCTTATGTGGTCAAGGATAGGGTTATATCTAAACAATATAATGAAACTTCACCT
CAAGTTGTAGAATGTCTGATAGTACTGGAGGTTCTTGAGGCTTTTCCAGGACCCCTCAATATTGTATCAGATTCCTCTTATGTGGTTAATGCAGTTAATCTCCTTGAGATAGCTGGAATAATAAGATCCTCCAGCAGAGTTGCTAACATCTTTCAAAAGATACAAGCTGCCTTGTTAAATAGGAGATTTCCTGTTTTCATTACCCATGTCCGAGCACATTCTGGAC
TCCCAGGTCCCATGTCCTTAGGGAATGACTTAGCGGACAAGGCTACAAAGCTGGTGGCCGCTGCCTTGTCTACCCATGCACAAGCAGCAAAAGAATTCCATAAGCGCTTTCATGTGACGGCTGAGACCTTGCGCCGTCGTTTTGCTCTATCCAGGAAAGAGGCTAGGGAAATTGTTACTCAATGCCAGAATTGCTGCGAATTCTTACCTACTCCCCACATGGGAAT
AAACCCACGTGGCATTAGACCATTGCAGATGTGGCAGATGGATGTTACTCATATTCCCTCTTTTGGAAGGCTTCAATATGTTCATGTTTCTGTTGACACCTGTTCTGGCGTAATGTTTGCCACACCCCTAACAGGGGAAAAGGCCTCATATGTGATTCAACATTGCCTTGAGGCCTGGAGTGCTTGGGGAAAACCCAGAATTTTAAAAACAGACAATGGACCAGCA
TATACCTCTCAAAAATTCCGACAGTTTTGTCGGCAAATGGATGTTACCCATCTAACTGGCCTACCTTATAACCCTCAAGGACAGGGCATTGTGGAACGTGCCCATCGTACACTCAAATCATATCTGATTAAACAAAAGGGGAGTATTGAGGATGTTCTACCCTCGGTACCAAGAGTTGCTGTATCCATGGCACTTTTTACCCTTAATTTCTTGAACTCTGATGCCC
AAGGCCACACTGCGGCTGATCGCCACAGTTTAGAGCCTGATAGGCCAAAAGAAATGGTAAAATGGAAAGATGTCTTGACTGATCTCTGGAAAGGCCCGGATCCTATTCTTATAAGATCCAGGGGAGCTGTGTGTGTTTTTCCACAGGAGGAAGAAAACCCCCTGTGGATCCCTGAAAGACTCACTCAAAGAGCTCCTTCACAACTCCAGAATAAGAAGGATGGAGG
CCAAGTGGGTGATGAAGACTCTCCTACTCGGGATGATGGTTCCTAGTATGATGGGAGAGTTACGGTGGGCCATCATGTCTACCTTCCCCCCTACCGATGCCAGTGATGCAGAATGCCCAGGTGTTCCCTATATTTTTCACAACTAATAGAGAGTTGGGGCTTGCCTTTTTGCCTTTAGATCCTCAGATACAGAGTTTTCAGGATGAGAGAACCTTGTCATTAGTAGG
AAGCTTGTGCTTTACTGTGATTTCTTCATTAAAGGATAATTTTATTCATATGGATCCAGAAAGATCTAATGCTAATCCTTTTGAATTAATGGAAGCTGTACTGATCTTTCTCCTATGATAGTGAGAAGGGACAGATTACTTTTGATTGGAAAGGGGCATTAGGCAACAGTCTGAGTGAGGGACAGAGCTCTGGATCATCTGATTGGTCCTCCTCCAATGTTAAGTA
TACCCCTTTTAAGAGAACTAATTATTCAGCTGCCCTTTATTTGGGTTGTGAGTAATGATACCTTAGGTAAGAATCAGAAAGAAATAAATTGCTCTGCTTAGAATTGTTTTTATGCATTATGCTGGGATGCTATTAAGCACCCTTTTGCTTTGGTAATGCGCATGCCTCGATTTGTTCCAGTTCCTGTTGAGACGCCAAGTAATTTGAGTTTGTTTAGGGAAAAGCG
AGATTTTTGGGATCTCTGCTATTATTGTGGGCCTGATAGCCACCGCCGCGGTTACTGCTTCGGTTACCGCCTCGGCCCTTGCACTTTCCGCCACTGTGCAGACAACTCAAACCATTAATGAACTATAGGCCACTGTGACCATGGTCCTAGATAAACAGGCCACTGCCAATTCTCAGATACAAGGAGGCCTGATGTTGGTAAACCAACAGATTAATTTGGTTCAGGA
ACAATTGGATATATTGTGGCAGATGGCCCAATTGGGATGTGAACAAAAGTTGCCTGGCCTTTGTGTCACTTCAGTACAATATGAGAATTTTACAAGAGCAGCTAACCTGTCACAAGCTCTGTCTCAACATTTATTACAGAATTGGACTTTTGAGTTTGAGCAGACACTCCGTGAGTTGAGAATGGCGATCCTGCAAGTGAATTCGACGCATCTGGATCTTTCTCTG
ATGGAAGACCTCTCCCCCTGGATTTCATCTGCATTTTCTTATTTTAAGGAATGGGTGGGGGTAGGATCATTTAGCCTAGTTCTCTGCTGTGGAATAGTGTTGATGCTTTGGATGGTGTGTAAGCTCAGAGCCCAAACAAAAGAGACAAAGTGGTGGTGGCACAAGCACTTGCTGCCTTAGAACACGGAGCCTCCCCTGACATTTGGTTAACCATGTTAAAGCAATA
GGTCGCTGGCCTCCCAGCTCTTGCACCCCACGAGGCTAGTCTCATTGCACGGGATAGAGTGGGTAGGCTTCAGCAGCCCAAGGAGTTGCAAGGCTAAGCACTGCACAGGAGAGGTCTGCGGTATAACGACTTTCTCCTGGGAGATAAGTCATCTTGCATGAAGGTTCTATGTCATGATCTCCTTCCCCCAGAAAAACGACATCGGACTGGTCAGGGCTCTCTCTGG
GGATAAGACCCTGGAGAGGAGCTTTGTACATATTCCGAATTTGCACATGGGGATTTGACCTCTATCTCCACTCCAAAGTTGTGGGTGTCCTATTGCTTCAAAAATAAATGAATAGGGGGAGATGTGAGGAGCCGCCCTCGCTATCGCTATTGCCGTTAGAAGATGGCGCTGACATCTGCTGTCAGTTGGAGTTAACCGTTAGAAGATGGCGCTGACATCTGCTGTC
AGTTGGAGTTAACCGTTTAAACTGTGCCTCTCCCGTGACGTCATCTGGGGTGATGTGCAAACCACCAATCCCGGCTCTACACATCTCACTCGGAGCTCCTAGGCTTATATAAGGGGCTGGGTTTCTTAGCTTGGGGTCTCCCTCTAAGAAGCTGATCATCTATCTCTCAAGATGCATTAAAGCTTTACTGCAGAAGGATCCGAGTGTTCTGCGTCGTTCTTGCTGG
CGAGACGGTAGCGCGGGACA

>IAPLTR3.2282330
TGTGAGGAGCCGCCCTCGCTATCGCTATTGCCGTTAGAAGATGGCGCTGACATCCACTGTCAGTTGGAGTTAACTGTCGCTGTCAGTTGGAGTTAACTGTCGCTGTCAGTTGGAGTTAACTGTCGCTGTCAGTTAGAGTTAACTGTCGCTGTCAGTTGGAGTTAACCGTTTAAACTGTGCCTCTCCCGTGACGTCATCTGGGGTGATGTGCAAACCACCAATCCCG
GCTCTACACATCTCACTCGGAGCTCCTAGGCTTATATAAGGGGCTGGGTTTCTTAGCTTGGGGTCTCCCTCTAAGAAGCTGATCATCTATCTCTCAAGATGCATTAAAGCTTTACTGCAGAAGGATCCGAGTGTTCTGCGTCGTTCTTGCTGGCGAGACGGTAGCGCGGGACATCTGGTGCCGAAACCCGGGAACTCTTCAACATCGCCGGCGCCGCGGGAGACCC
CTCGGAAAACGGGGCGGATTCAGAACTGCAGGGACGTAGGTTCAGAGAGGTATGCTTTATCCTGAACAACCCTTTTCTCGACTTTGGCCTTAGTTTGTCATCACCGTGGGACGGGTCAGTGGAGGCTTTGGTGCTAGTCCTGTTTGTCCTTCTCCTTCATCGTGGCTGGTGCTGACAGAACGGCGCTGTCCGTCTGGCGCTGTCCGTCTGGCGCTGTCCATCTGGC
GCTGTCCATCTGGCGCTGTCCATCTGGCGCTGTCCATCTGGCGCTGTCAGTCTGGTGCTGTCAGTCTGGTGCTGTCAGTCTGGTGCTGTCAGTCTGGTAACGGAGTTGAGCGCGTTTTTTAGATAAGCTGTCAGCGCGATGGGGTCGTCACAGTCAGTGGTTACAGCCTTACAGACTGTGCTAAAACAAAGAGATCTTAAAATTGCACCACGAACACTTCAAAATT
TTGTGAAGGAGGTAGATAGAGTGGCACCATGGTATGCTTGTTCCGGGTCCCTCACTGTGGCCTCATGGAACAAGCTAGGTAAAGAACTAGACAGGAAGCATGCAGAAGGAGATCTCTGCTTAGGTACCAAAGCCATTTGGAAGCTAGTTAAAACTGCTTGGAGGATGAGGCCTGTCACCCTGCTATAATAGAAAGTCAGGGAACCTTAGAGGAAGTTCAGGATAG
CATGTCAGAAACTGAACGGAGTGAGAGAATGGGAGCTCGCAAAAGAAAAGACATGTCTAAGAAAAAGGCCCTCCCCAGGAAGTTAAGAAAGGGGGAGAGAAAGAAGGGAGTTATCATTCACCCCTTAACAAATCTAAGAAAAAGAAAAAGCCAGAATCTAGTCTATACCCTGCAGTAGAGCTAGAAGCCTTGGAGCTAGATAATTCTGATTCTGACACTCTGGAT
TCTAGCGAAGAAGGTGGTCTAGAGGAGGAGGTGGCACGGTATGAGGAAAAAAGATATCACCCTGATAGACACCGGCCACTGAAAACAAAAATGAATGTGAGGCCACCGCCTATAAATCCGGCGGGCTCACGCCCCTCAGCGCCTCCGCCATATGAGCTTCGGCTGAATACTGGTACTGACTCCTTTTTACCTTTAGAGGAACGGAGAAAAATACAAATGACGGTTTC
CAGTATTCGAGAATGTGGAAGGCGGAAGAGTACATACCCCCGTAGATTACAATCAAATCAAAGAATTGGCCGAATCCGTTCGTAATTATGGGGTCAATGCTAACTTTACCACATTACAGGTGGAAAGGCTTGCTAATTTTGCCATGACTCCCACTGACTGGCAAACGACAGTGAAAGCAGTGCTTCCCAACATGGGACAATACATGGAATGGAAGGCTCTCTGGTA
TGATGCAGCCCAGGCACAAGCTAGAGTTAATGCCACGGCTGAAGATGAGAATCAGAGGCAGTGGACATTTGAAATGCTGACAGGTCAGGGTCAACATGCCCTTAATCAAACCAACTACATCTGGGGCGTCTATGGTCAGGTGTCGGCCGCCGCTATTAAAGCATGGAAGGCCCTCACAAAAGGGGAGAGTCCAGTGGACACCTTACAAAGATTGTCCAAGGACCC
CAAGAGCCATTTTCAGACTTTGTGGCCAGAATGACAGAGGCCGCCAGTCGCATCTTTGGTGATTCAGAACAAGCTATGCCCCTGATTGAGCAACTTGTCTATGAACAAGCAACCCAGGAATGTCGAGCGGCTATCGCCCCTCGCAAAAGCAAAGGTTTACAAGATTGGCTCAGAGTGTGCAGGGAGCTGGGGGGACCCCTAACCAATGCAGGACTAGCTGCCGCCA
TTTTACAAACGCACAGGTATAGAGATTTATCTAATCGAAAAGCTTGTTTTAATTGTGGGAGAATGGGGCACCTTAAGAAAGATTGTCAAGCCCCTGAAAGAACAAGAGAATCGAAGCTTTGCTATCGTTGTGGTAAAGGTTATCATAGGGCTAGCGAATGCAGGTCAGTCCGGGATGTAAAGGGAGGCTTCTGCCCCCTATTGAAGAATCCAAGGTACCCCAATC
AAAAAACGGGTCACGGGGCCCATGGTCCCAGGGCCCTCAGAAATATGGGAACCAATTTCAGAAAAGCAATCAAGAGGAAGTAGAGGTTTCCGAGGAACCCCCGGAATGGACTTGCGTGCCGCCTCCGACTTCTTACTCATGCCCCAAATGAATGTTCAACCGGTCCCAGTCCATTCCCCTGGACCTTTGCCCCCTGCCACCATTGGGCTTATTCTGGGCCGTGGCT
CCTTAACTTTACAGGGACTCATTATATATCCTGGGATTGTTGACCCATATCATAAAGAGGAGATTCAAGTTCTCTGTTCTAGCCCTCGGGGTGTATTTTCTATAAAGCAGGGAGATAGAATTGCACAACTGGTGTTGCTGCCCTCCCTCGGGGATGGAGAAACCTACACTTTACAAAAAGGGCTATGGGATCTTCTGGAAGTGATTCAGCCTATCTGGCTATACC
CCTAAATGAGAGACCTACAATAAAATTGTTAATTAATGGAAAAGAATTTGAAGGGATAATGGATTCGGGAGCAGACAAAAGCATTATTTCACTCCATTGGTGGCCGAAGTCTTGGCCCACTGTTGTTTCATCTCATTCTCTACAAGGTCTTGGATACCAGTCCTCTCCTGCCATCAGTGCCTCAGCCTTAACCTGGCGGATGCTGAAGGCAAACAGGGATGTTT
ACCCCCTATGTGTTGCCACTCCCTGTAAATTTATGGGGACGAGATGTGTTACAAGCCATGGGCATGACCCTAACTAATGAATACTCCCCCCAGGCATCAGCCATTATGGCAAAGATGGGGTATACAAATGGTAGAGGCTTGGGTAGGCAAGAACAAGGCAGAATAAAACCCATTACACAACACGGAAATCGGGGTAGAAAAGGACTGGGTTTTATTTAGGGGCCGT
TGAGGCTTCACGACCCATACCATGGAAACAGAGGAGCCGTTATGGGTCTCTCAATGGCCTCTATCCTCTGAAAAATTAGAGGCTGTCACAAGATTAGTGCAAGAACAGGAACGGCTGGGGCATCTAGAGCCTTCTACCTCCCCATGGAATACACCAATTTTTGTTATTAAGAAGAAATCTGGGAAGTGGAGATTACTCCACGACCTGCGGGCCATTAACAATCAG
ATGCATCTTTTTGGCCCTGTTCAAAGAGGCCTTCCTTTGCTTTCTGCACTTCCTCAAGATTGGAAGCTTATTATTATAGATATTAAGGATTGTTTCTTCTCTATTCCACTTTACCCACGGGATAGACCAAGGTTTGCCTTCACTATCCCTTCTCTTAATCATATGGAACCAGACAAGAGATTTCAGTGGAAGGTACTGCCGCAAGGCATGGCCAATAGCCCAACAA
TATGTCAGCTATATGTGCAGGAGGCTTTGGAGCCAATTAGGAAGCAATTTACATCTTTAATCGTTATCCATTATATGGACGATATTCTTATTTGTCACAAAGAGTTAGATGTTTTACAAAGGCTTTCCCATGCTGGTGGCTGAATTAAAACAGTGGGGATTAGAGATTGCCTCAGAAAAGGTTCAAATTGCAGATACAGGCCTCTTTTTGGGCTCAAAGATTAC
TCCCAAAAATATAGTTCCTCAGAAAATAGAAATTCGCAAAGACCACTTACAAACATTAAATGATTTTCAGAAACTTCTGGGAGATATAAATTGGTTGAGGCCCTTTTTAAAAATTCCATCTGCTGATCTAAAACCCCTCTTCGATCTCCTGGAGGGTGAGCCTCATATCTCCTCACCCAGAAAATTTACCCCTGCCGCCCACCGAGCCTTACAGATGGTGGAAGAG
GCCTTACAGGAAGCACAATTACAACGAATTGAGGAGTCACTACCTTTTAATCTGTGTGTTTTTAAGACAGCTCAATTGCCAACTGCAGTCCTGTGGCAACATGGACCATTACTTTGGATTCATCCAAATGCTTCCCCTGCTAAGATTATTGACTGGTACCCTGATGCTGTTGCACAGCTCGCGCTTCGCGGAATAAAAGCAGCTGTTACACATTTTGGCAGGGATC
CTGATTCCTTGATTGTGCCTTATACTGCTGCACAAGTTCAGACCTTGGCAGCCACATCTAGTGATTGGGCAGTTTTAGTTACTTCCTTTTCAGGACAAATTGATAATCATTTTCCAAAGCATCCAATTTTACAGTTTGCCCTAAACCAGGCTATAGTGTTTCCACAAGTGACAGCTAAAGACCCACTCCCAGATGGGACTGTGGTGTACACTGATGGATCAAAAC
TGGTATGGGAGCTTATGTGGTCAAGGATAGGGTTATATCTAAACAATATAATGAAACTTCACCTCAAGTTGTAGAATGTCTGATAGTACTGGAGGTTCTTGAGGCTTTTCCAGGACCCCTCAATATTGTATCAGATTCCTCTTATGTGGTTAATGCAGTTAATCTCCTTGAGATAGCTGGAATAATAAGATCCTCCAGCAGAGTTGCTAACATCTTTCAGAAGATA
CAAGCTGCCTTGTTAAATAGGAGATTTCCTGTTTTCATTACCCATGTCCGAGCACATTCTGGACTCCCAGGTCCCATGTCCTTAGGGAATGACTTAGCGGACAAGGCTACAAAGCTGGTGGCCGCTGCCTTGTCTACCCATGCACAAGCAGCAAAAGAATTCCATAAGCGCTTTCATGTGACGGCTGAGACCTTGCGCCGTCGTTTTGCTCTATCCAGGAAAGAGG
CTAGGGAAATTGTTACTCAATGCCAGAATTGCTGCGAATTCTTACCTACTCCCCACATGGGAATAAACCCACGTGGCATTAGACCATTGCAGATGTGGCAGATGGATGTTACTCATATTCCCTCTTTTGGAAGGCTTCAATATGTTCATGTTTCTGTTGACACCTGTTCTGGCGTAATGTTTGCCACACCCCTAACAGGGGAAAAGGCCTCATATGTGATTCAACA
TTGCCTTGAGGCCTGGAGTGCTTGGGGAAAACCCAGAATTTTAAAAACAGACAATGGACCAGCATATACCTCTCAAAAATTCCGACAGTTTTGTCGGCAAATGGATGTTACCCATCTAACTGGCCTACCTTATAACCCTCAAGGACAGGGCATTGTGGAACGTGCCCATCGTACACTCAAATCATATCTGATTAAACAAAAGGGGAGTATTGAGGATGTTCTACCC
TCGGTACCAAGAGTTGCTGTATCCATGGCACTTTTTACCCTTAATTTCTTGAACTCTGATGCCCAAGGCCACACTGCGGCTGATCGCCACAGTTTAGAGCCTGATAGGCCAAAAGAAATGGTAAAATGGAAAGATGTCTTGACTGATCTCTGGAAAGGCCCGGATCCTATTCTTATAAGATCCAGGGGAGCTGTGTGTGTTTTTCCACAGGAGGAAGAAAACCCCC
TGTGGATCCCTGAAAGACTCACTCGAAGAGCTCCTTCACAACTCCAGAATAAGAAGGATGGAGGCCAAGTGGGTGATGAAGACTCTCCTACTCGGGATGATGGTTCCTAGTATGATGGGAGAGTTACGGTGGGCCATCATGTCTACCTTCCCCCCTACCGATGCCAGTGATGCAGAATGCCCAGGTGTTCCCTAGATTTTTCACAAATAATAGAGAGTTGGGGCTTG
CCTTTTTGCCTTTAGATCCTCAGATACAGAGTTTTCAGGATGAGAGAACCTTGTCATTAGTAGGAAGCTTGTGCTTTACTGTGATTTCTTCATTAAAGGATAATTTTATTCATATGGATCCAGAAAGATCTAATGCTAATCCTTTTGAATTAATGGAAGCTGTACTGATCTTTCTCCTATGATAGTGAGAAGGGACAGATTACTTTTGATTGGAAAGGGGCATTAG
GCAACAGTCTGAGTGAGGGACAGAGCTCTGGATCATCTGATTGGTCCTCCTCCAATGTTAAGTATACCCCTTTTAAGAGAACTAATTATTCAGCTGCCCTTTATTTGGGTTGTGAGCAATGATACCTTAGGTAAGAATCAGAAAGAAATAAATTGTTCTGCTTAGAATTGTTTTTATGCATTATGCTGGGATGCTATTAAGCACCCTTTTGCTTTGGTAATGCGCA
TGCCTCGATTTATCCAGCTCCTGTTGAGGCGCCAAGTAATTTGAGTTTGTTTAGGGAAAAGCGAGATTTTTGGGATCTCTGCTATTATTGTGGGCCTGATAGCCACCGCCGCGGTTACTGCTTCGGTTACCGCCTCGGCCCTTGCACTTTCTGCCACTGTGCAGACAACTCAAACCATTAATGAACTATAGGCCACTGTGACCATGGTCCTAGATAAACAGGCCA
CTGCCAATTCTCAGATACAAGGAGGCCTGATGTTGGTAAACCAACAGATTAATTTGGTTCAGGAACAATTGGATATATTGTGGCAGATGGCCAAATTGGGATGTGAACAAAGTTGCCTGGCCTTTGTGTCACTTAGTACAATATGAGAATTTTACAAGAGCAGCTAACCTGTCAAAAGCTCTGTCTCAACATTTATTACAGAATTGGACTTTTGAGTTTGAGCAGA
CACTCCGTGAGTTGAGAATGGCGATCCTGCAAGTGAATTCGACGCATCTGGATCTTTCTCTGATGGAAGACCTCTCCCCCTGGATTTCATCTGCATTTTCTTATTTTAAGGAATGGGTGGGGGTAGGATCATTTAGCCTAGTTCTCTGCTGTGGAATAGTGTTGATGCTTTGGATGGTGTGTACGCTCAGAGCCCAAACAAAGGAGAAAGTGGTGGTGGCACAAG
CACTTGCTGCCTTAGAACACGGAGCATCCCCTGACATTTGGTTAACCATGTTAAAGCAATAGGTCGCTGGCCTCCCAGCTCTTGCACCCCACGAGGCTAGTCTCATTGCACGGGATAGAGTGGGTAGGCTTCAGCAGCCCAAGGAGTTGCAAGGCTAAGCACTGCACAGGAGAGGTCTGCGGTATAACGACTTTCTCCTGGGAGATAAGTCATCTTGCATGAAGGT
TCTATGTCATGATCTCCTTCCCCCAGAAAAAGACATCGGACTGGTCAGGACTTCCTCTGGGGATAAGACCCTGGAGAGGAGCTTTGTACATGTTCCGAATTTGCACATGGGGATTTGACCTCTATCTCCACTCCAAAGTTGTGGGTGTCCTATTGCTTCAAAAATAAATGAATAGGGGGAGATGTGAGGAGCCGCCCTCGCTATCGCTATTGCCGTTAGAAGATG
GCGCTGACATCCACTGTCAGTTGGAGTTAACTGTCGCTGTCAGTTGGAGTTAACTGTCGCTGTCAGTTGGAGTTAACTGTCGCTGTCAGTTAGAGTTAACTGTCGCTGTCAGTTGGAGTTAACCGTTTAAACTGTGCCTCTCCCGTGACGTCATCTGGGGTGATGTGCAAACCACCAATCCCGGCTCTACACATCTCACTCGGAGCTCCTAGGCTTATATAAGGGG
CTGGGTTTCTTAGCTTGGGGTCTCCCTCTAAGAAGCTGATCATCTATCTCTCAAGATGCATTAAAGCTTTACTGCAGAAGGATCCGAGTGTTCTGCGTCGTTCTTGCTGGCGAGACGGTAGCGCGGGACA

>IAPLTR3.1975241
TGTGAGGAGCCGCCCTCGCTATCGCTATTGCCGTTAGAAGATGGCGCTGACATCCACTGTCAGTTGGAGTTAACTGTCGCTGTCAGTTGGAGTTAACTGTCGCTGTCAGTTGGAGTTAACCGTTTAAACTGTGCCTCTCCCGTGACGTCATCTGGGGTGATGTGCAAACCACCAATCCCGGCTCTACACATCTCACTCGGAGCTCCTAGGCTTATATAAGGGGCTG
GGTTTCTTAGCTTGGGGTCTCCCTCTAAGAAGCTGATCATCTATCTCTCAAGATGCATTAAAGCTTTACTGCAGAAGGATCCGAGTGTTCTGCGTCGTTCTTGCTGGCGAGACGGTAGCGCGGGACATCTGGTGCCGAAACCCGGGAACTCTTCAACATCGCCGGCGCCGCGGGAGACCCCTCGGAAGACGGGGCGGATTCAGAACTGCAGGGACGTAGGTTCAGA
GAGGTATGCTTTATCCTGAACAACCCTTTTCTCGACTTTGGCCTTAGTTTGTCATCACCGTGGGACGGGTCAGTGGAGGCTTTGGTGCTAGTCCTGTTTGTCCTTCTCCTTCATCGTGGCTGGTGCTGACAGAACGGCGCTGTCCGTCTGGCGCTGTCCGTCTGGCGCTGTCCATCTGGCGCTGTCAGTCTGGTGCTGTCAGTCTGGTGCTGTCAGTCTGGTGCTG
TCAGTCTGGTAACTGAGTTGAGCGCCGTTTTTTAGATAAGCCGCTAGCGCGATGGGGTTGTCACAGTCAGTGGTTACTGCCCTACAGACTGTGCTAAAACAAAGAGATCTTAAAATTGCACCACGAACACTTCAAAATTTTGTGAAGGAGGTAGATAGAGTGGCACCATGGTATGCTTGTTCCGGGTCCCTCACTGTGGCCTCATGGAACAAGCTAGGTAAAGAAC
TAGACAGGAAGCATGCAGAAGGAGATCTCCACTTAGGTACCAAAGCCATTTGGAAGCTAGTTAAAACTGCTTGGAGGATGAGGCCTGTCACCCTGCTATAATAGAAAGTCAGGGAACCTTAGAGGAAGTTCAGGATAGCATGTCAGAAACTGAACGGAGTGAGAGAATGGGAGCTCGCAAAAGAAAAGACATGTCTAAGAAAAAAGGCCCTCCCCAGGAGTTAA
GAAAGGGGGAGAGAAAGAAGGGAGTTATCATTCACCCCTTAACAAATCTAAGAAAAAGAAAAAGCCAGAATCTAGTCTATACCCTGCAGTAGAGCTAGAAGCCTTGGAGCTAGATAATTCTGATTCTGACACTCTGGATTCTAGCGAAGAAGGTGGTCTAGAGGAGGAGGTGGCACGGTATGAGGAAAAAAGATATCACCCTGATAGACACCGGCCACTGAAAACA
AAAATGAATGTGAGGCCACCGCCTATAAATCCGGCGGGCTCACGCCCCTCAGCGCCTCCGCAGTATGAGCTTCGGCTGAATACTGGTACTGACTCCTTTTTACCTTTAGAGGAACGGAGAAAAATACAAATGGCTTTCCAGTATTCGAGAATGTGGAAGGCGGAAGAGTACATACCCCCGTAGATTACAATCAAATTAAAGAATTGGCCGAATCCGTTCGTAATT
ATGGGGTCAATGCTAACTTTACCACATTACAGGTGGAAAGGCTTGCTAATTTTGCCATGACTCCCACTGACTGGCAGACGACAGTGAAAGCAGTGCTTCCCAACATGGGACAATACATGGAATGGAAGGCTCTCTGGTATGATGCAGCCCAGGCACAAGCTAGAGTTAATGCCACGGCTGAAGATGAGAATCAGAGGCAGTGGACATTTGAAATGCTGACAGGTCA
GGGTCAACATGCCCTTAATCAAACCAACTACATCTGGGGTGTCTATGGTCAGGTGTCGGCCGCCGCTATTAAAGCATGGAAGGCCCTCACAAAAAGGGGAGAGTCCAGTGGACACCTTACAAAGATTGTCCAAGGACCCCAAGAGCCATTTTCAGACTTTGTGGCCAGAATGACAGAGGCCGCCAGTCGCATCTTTGGTGATTCAGAACAAGCTATGCCCCTGATT
GAGCAACTTGTCTATGAACAAGCAACCCAGGAATGTCGAGCGGCTATCGCCCCTCGCAAAAGCAAAGGTTTACAAGATTGGCTCAGAGTGTGCAGGGAGCTGGGGGGACCCCTAACCAATGCAGGACTAGCTGCCGCCATTTTACAAACGCACAGGTATAGAGATTTATCTAGTCGAAAAGCTTGTTTTAATTGTGGGAGAATGGGGCACCTTAAGAAAGATTGTC
AAGCCCCTGAAAGAACAAGAGAATCGAAGCTTTGCCATCGTTGTGGTAAAGGTTATCATAGGGCTAGCGAATGCAGGTCAGTCCGGGATGTAAAAGGGAGGCTTCTGCCCCCTATTGAAGAATCCAAGGTACCCAATCAAAAAACGGGTCACGGGGCCCATGGTCCCAGGGCCCTCAGAAATATGGGAACCAATTTCAGAAAAGCAATCAAGAGGAAGTAGAGGT
TTCCGAGGAACCTCCGGAATGGACTTGCGTGCCGCCTCCGACTTCTTACTCATGCCCCAAATGAATGTTCAACCGGTCCCAGTCCATTCCCCTGGACCTTTGCCCCCTGCCACCATTGGGCTTATTCTGGGCCGTGGCTCCTTAACTTTACAGGGACTCATTATATATCCTGGGATTGTTGACCCATATCATAAAGAGGAGATTCAAGTTCTCTGTTCTAGCCCTC
GGGGTGTATTTTCTATAAAGCAGGGAGATAGAATTGCACAACTGGTGTTGCTGCCCTCCCTCGGGGATGGAGAAACCTACACTTTACAAAAAAGGGCTATGGGATCTTCTGGAAGTGATTCAGCCTATCTGGCTATACCCCTAAATGAGAGACCTACAATAAAATTGTTAATTAATGGAAAAGAATTTGAAGGGATAATGGATATGGGAGCAGACAAAAGCATTAT
TTCACTCCATTGGTGGCCGAAGTCTTGGCCCACTGTTGTTTCATCTCATTCTCTACAAGGTCTTGGATACCAGTCCTCTCCTGCCATCAGTGCCTCAGCCTTAACCTGGCGGAATGCTGAGGGCAAACAGGGATGTTTTACCCCCTATGTGTTGCCACTCCCTGTAAATTTATGGGGACGAGATGTGTTACAAGCCATGGGCATGACCCTAACTAATGAATACTCC
CCCCAGGCATCAGCCATTATGGCAAAGATGGGGTATACAAAGGGTAGAGGCTTGGGTAGGCAAGAACAAGGCAGAATAAAACCCATTACGCAACACGGAAATCGGAGTAGAAAAGGACTGGGTTTTATTTAGGGGCCGTTGAGGCTTCACGACCCATACCATGGAAAACAGAGGAGCCGTTATGGGTCTCTCAATGGCCTCTATCCTCTGAAAAATTAGAGGCTGT
CACAAGATTAGTGCAAGAACAGGAACGGCTGGGGCATCTAGAGCCTTCTACCTCCCCATGGAATACACCAATTTTTGTTATTAAGAAGAAATCTGGGAAGTGGAGATTACTCCACGACCTGCGGGCCATTAACAATCAGATGCATCTTTTTGGCCCTGTTCAAAGAGGCCTTCCTTTGCTTTCTGCACTTCCTCAAGATTGGAAGCTTATTATTATAGATATTAAG
GATTGTTTCTTCTCTATTCCACTTTACCCACGGGATAGACCAAGGTTTGCCTTCACTATCCCTTCTCTTAATCATATGGAACCAGACAAGAGATTTCAGTGGAAGGTACTGCCGCAAGGCATGGCCAATAGCCCAACAATATGTCAGCTATATGTGCAGGAGGCTTTGGAGCCAATTAGGAAGCAATTTACATCTTTAATCGTTATCCATTATATGGACGATATTC
TTATTTGTCACAAAGAGTTAGATGTTTTACAAAGGCTTTTCCCATGCTGGTGGCTGAATTAAAACAGTGGGGATTAGAGATTGCCTCAGAAAAGGTTCAAATTGCAGATACAGGCCTCTTTTTGGGCTCAAAGATTACTCCCAAAAATATAGTTCCTCAGAAAATAGAAATTCGCAAAGACCACTTACAAACATTAAATGATTTTCAGAAACTTCTGGGAGATAT
AAATTGGTTGAGGCCCTTTTTAAAAATTCCATCTGCTGATCTAAAACCCCTCTTCGATCTCCTGGAGGGTGAGCCTCATATCTCCTCACCCAGAAAATTTACCCCTGCCGCCCACCGAGCCTTACAGATGGTGGAAGAGGCCTTACAGGAAGCACAATTACAACGTATTGAGGAGTCACTACCTTTTAATCTGTGTGTTTTTAAGACAGCTCAATTGCCAACTGCA

[illegible]

>IAPLTR3.3305705

[illegible]

>IAPLTR3.3312096

[illegible]

>IAPLTR3.1225160

[illegible]

>IAPLTR3.2703412

[illegible]

TTAGGGAAAAGCGAGATTTTTGGGATCTCTGCTATTATTGTGGGCCTGATAGCCACCGCCGCGGTTACTGCTTCGGTTACCGCCTCGGCCCTTGCACTTTCTGCCACTGTGCAGACAACTCAAACCATTAATGAACTATAGGCCACTGTGACCATGGTCCTAGATAAACAGGCCACTGCCAATTCTCAGATACAAGGAGGCCTGATGTTGGTAAACCAACAGATTA
ATTTGGTTCAGGAACAATTGGATATATTGTGGCAGATGGCCAAATTGGGATGTGAACAAAGTTGCCTGGCCTTTGTGTCACTTAGTACAATATGAGAATTTTACAAGAGCAGCTAACCTGTCAAAAGCTCTGTCTCAACATTTATTACAGAATTGGACTTTTGAGTTTGAGCAGACACTCCGTGAGTTGAGAATGGCGATCCTGCAAGTGAATTCGACGCATCTGG
ATCTTTCTCTGATGGAAGACCTCTCCCCCTGGATTTCATCTGCATTTTCTTATTTTAAGGAATGGGTGGGGGTAGGATCATTTAGCCTAGTTCTCTGCTGTGGAATAGTGTTGATGCTTTGGATGGTGTGTACGCTCAGAGCCCAAACAAAAGGAGAAAGTGGTGGTGGCACAAGCACTTGCTGCCTTAGAACACGGAGCATCCCCTGACATTTGGTTAACCATGT
TAAAGCAATAGGTCGCTGGCCTCCCAGCTCTTGCACCCCACGAGGCTAGTCTCATTGCACGGGATAGAGTGGGTAGGCTTCAGCAGCCCAAGGAGTTGCAAGGCTAAGCACTGCACAGGAGAGGTCTGCGGTATAACGACTTTCTCCTGGGAGATAAGTCATCTTGCATGAAGGTTCTATGTCATGATCTCCTTCCCCCAGAAAAAAGACATCGGACTGGTCAGGA
CTTCCTCTGGGGATAAGACCCTGGAGAGGAGCTTTGTACATGTTCCGAATTTGCACATGGGGATTTGACCTCTATCTCCACTCCAAAGTTGTGGGTGTCCTATTGCTTCAAAAATAAATGAATAGGGGGAGATGTGAGGAGCCGCCCTCGCTATCGCTATTGCCGTTAGAAGATGGCGCTGACATCCACTGTCAGTTGGAGTTAACTGTCGCTGTCAGTTGGAGTT
AACTGTCGCTGTCAGTTGGAGTTAACTGTCGCTGTCAGTTGGAGTTAACCGTTTAAACTGTGCCTCTCCCGTGACGTCATCTGGGGTGATGTGCAAACCACCAATCCCGGCTCTACACATCTCACTCGGAGCTCCTAGGCTTATATAAGGGGCTGGGTTTCTTAGCTTGGGGTCTCCCTCTAAGAAGCTGATCATCTATCTCTCAAGATGCATTAAAGCTTTACTG
CAGAAGGATCCGAGTGTTCTGCGTCGTTCTTGCTGGCGAGACGGTAGCGCGGGACA

>IAPLTR3.2308497
TGTGAGGAGCCGCCCTCGCTATCGCTATTGCCGTTAGAAGATGGCGCTGACATCCACTGTCAGTTGGAGTTAACTGTCGCTGTCAGTTGGAGTTAACTGTCGCTGTCAGTTGGAGTTAACTGTCGCTGTCAGTTAGAGTTAACTGTCGCTGTCAGTTGGAGTTAACCGTTTAAACTGTGCCTCTCCCGTGACGTCATCTGGGGTGATGTGCAAACCACCAATCCCG
GCTCTACACATCTCACTCGGAGCTCCTAGGCTTATATAAGGGGCTGGGTTTCTTAGCTTGGGGTCTCCCTCTAAGAAGCTGATCATCTATCTCTCAAGATGCATTAAAGCTTTACTGCAGAAGGATCCGAGTGTTCTGCGTCGTTCTTGCTGGCGAGACGGTAGCGCGGGACATCTGGTGCCGAAACCCGGGAACTCTTCAACATCGCCGGCGCCGCGGGAGACCC
CTCGGAAGACGGGGCGGATTCAGAACTGCAGGGACATAGGTTCAGAGAGGTATGCTTTATCCTGAACAACCCTTTTCTCGACTTTGGCCTTAGTTTGTCATCACCGTGGGACGGGTCAGTGGAGGCTTTGGTGCTAGTCCTGTTTGTCCTTCTCCTTCATCGTGGCTGGTGCTGACAGAACGGCGCTGTCCGTCTGGCGCTGTCCGTCTGGCGCTGTCCGTCTGGC
GCTGTCCGTCTGGCGCTGTCCGTCTGGCGCTGTCCGTCTGGCGCTGTCCATCTGGCGCTGTCCATCTGGCGCTGTCAGTCTGGTGCTGTCAGTCTGGCGCTGTCAGTCTGGTGCTGTCAGTCTGGTGCTGTCAGTCTGGTGCTGTCAGTCTGGTAACGGAGTTGAGCGCGTTTTTAGATAAGCTGTCAGCGCGATGGGGTCGTCACAGTCAGTGGTTACAGCCTT
ACAGACTGTGCTAAAACAAAGAGATCTTAAAATTGCACCACGAACACTTCAAAATTTTGTGAAGGAGGTAGATAGAGTGGCACCATGGTATGCTTGTTCCGGGTCCCTCACTGTGGCCTCATGGAACAAGCTAGGTAAAGAACTAGACAGGAAGCATGCAGAAGGAGATCTCTGCTTAGGTACCAAAGCCATTTGGAAGCTAGTTAAAAACTGCTTGGAGGATGAG
GCCTGTCACCCTGCTATAATAGAAAGTCAGGGAACCTTAGAGGAAGTTCAGGATAGCATGTCAGAAACTGAACGGAGTGAGAGAATGGGAGCTCGCAAAAGAAAAGACATGTCTAAGAAAAAAGGCCCTCCCCAGGAAGTTAAGAAAGGGGGAGAGAAAGAAGGGAGTTATCATTCACCCCTTAACAAATCTAAGAAAAAGAAAAAGCCAGAATCTAGTCTATACC
CTGCAGTAGAGCTAGAAGCCTTGGAGCTAGATAATTCTGATTCTGACACTCTGGATTCTAGCGAAGAAGGTGGTCTAGAGGGGGAGGTGGCACGGTATGAGGAAAAAAGATATCACCCTGATAGACACCGGCCACTGAAAACAAAAATGAATGTGAGGCCACCGCCTATAAATCCGGCGGGCTCACGCCCCTCAGCGCCTCCGCCATATGAGCTTCGGCTGAATAC
TGGTACTGACTCCTTTTTACCTTTAGAGGAACGGAGAAAAATACAAATGGCTTTTCCAGTATTCGAGAATGTGGAAGGCGGAAGAGTACATACCCCCGTAGATTACAATCAAATTAAAGAATTGGCCGAATCCGTTCGTAATTATGGGGTCAATGCTAACTTTACCACATTACAGGTGGAAAGGCTTGCTAATTTTGCCATGACTCCCACTGACTGGCAAACGACA
GTGAAAGCAGTGCTTCCCAACATGGGACAATACATGGAATGGAAGGCTCTCTGGTATGATGCAGCCCAGGCACAAGCTAGAGTTAATGCCACGGCTGAAGATGAGAATCAGAGGCAGTGGACATTTGAAATGCTGACAGGTCAGGGTCAACATGCCCTTAATCAAACCAACTACATCTGGGGCGTCTATGGTCAGGTGTCGGCCGCCGCTATTAAAGCATGGAAGG
CCCTCACAAAAAGGGGAGAGTCCAGTGGACACCTTACAAAGATTGTCCAAGGACCCCAAGAGCCATTTTCAGACTTTGTGGCCAGAATGACAGAGGCCGCCAGTCGCATCTTTGGTGATTCAGAACAAGCTATGCCCCTGATTGAGCAACTTGTCTATGAACAAGCAACCCAGGAATGTCGAGCGGCTATCGCCCCTCGCAAAAGCAAAGGTTTACAAGATTGGCT
CAGAGTGTGCAGGGAGCTGGGGGGACCCCTAACCAATGCAGGACTAGCTGCCGCCATTTTACAAACGCACAGGTATAGAGATTTATCTAATCGAAAAGCTTGTTTTAATTGTGGGAGAATGGGGCACCTTAAGAAAGATTGTCAAGCCCCTGAAAGAACAAGAGAATCGAAGCTTTGCTATCGTTGTGGTAAAGGTTATCATAGGCTAGCGAATGCAGGTCAGTC
CGGGATGTAAAAGGGAGGCTTCTGCCCCCTATTGAAGAATCCAAGGTACCCCAATCAAAAAACGGGTCACGGGGCCCATGGTCCCAGGGCCCTCAGAAATATGGGAACCAATTTCAGAAAAGCAATCAAGAGGAAGTAGAGGTTTCCGAGGAACCTCCGGAATGGACTTGCGTGCCGCCTCCGACTTCTTACTCATGCCCCAAATGAATGTTCAACCGGTCCCAGT
CCATTCCCCTGGACCTTTGCCCCCTGCCACCATTGGGCTTATTCTGGGCCGTGGCTCCTTAACTTTACAGGGACTCATTATATATCCTGGGATTGTTGACCCATATCATAAAGAGGAGATTCAAGTTCTCTGTTCTAGCCCTCGGGGTGTATTTTCTATAAAGCAGGGAGATAGAATTGCACAACTGGTGTTGCTGCCCTCCCTCGGGGATGGAGAAACCTACACT
TTACAAAAAAGGGCTATGGGATCTTCTGGAAGTGATTCAGCCATACCCCTAAATGAGAGACCTACAATAAAATTGTTAATTAATGGAAAAGAATTTGAAGGGATAATGGATTCGGGAGCAGACAAAAGCATTATTTCACTCCATTGGTGGCCGAAGTCTTGGCCCACTGTTGTTTCATCTCATTCTCTACAAGGTCTTGGATACCAGTCCTCTCCTGCCATCAGTG
CCTCAGCCTTAACCTGGCGGGATGCTGAAGGCAAACAGGGATGTTTTACCCCCTATGTGTTGCCACTCCCTGTAAATTTATGGGGACGAGATGTGTTACAAGCCATGGGCATGACCCTAACTAATGAATACTCCCCCAGGCATCAGCCATTATGGCAAAGATGGGGTATACAAATGGTAGAGGCTTGGGTAGGCAAGAACAAGGCAGAATAAAACCCATTACACA
ACACGGAAATCGGGGTAGAAAAGGACTGGGTTTTATTTAGGGGCCGTTGAGGCTTCACGACCCATACCATGGAAAACAGAGGAGCCGTTATGGGTCTCTCAATGGCCTCTATCCTCTGAAAAATTAGAGGCTGTCACAAGATTAGTGCAAGAACAGGAACGGCTGGGGCATCTAGAGCCTTCTACCTCCCCATGGAATACACCAATTTTTGTTATTAAGAAGAAAT
CTGGGAAGTGGAGATTACTCCATGACCTGCGGGCCATTAACAATCAGATGCATCTTTTGGCCCTGTTCAAAGAGGCCTTCCTTTGCTTTCTGCACTTCCTCAAGATTGGAAGCTTATTATTATAGATATTAAGGATTGTTTCTTCTCTATTCCACTTTACCCACGGGATAGACCAAGGTTTGCCTTCACTATCCCTTCTCTTAATCATATGGAACCAGACAAGAG
ATTTCAGTGGAAGGTACTGCCGCAAGGCATGGCCAATAGCCCAACAATATGTCAGCTATATGTGCAGGAGGCTTTGGAGCCAATTAGGAAGCAATTTACATCTTTAATCGTTATCCATTATATGGACGATATTCTTATTTGTCACAAAGAGTTAGATGTTTTACAAAGGCTTTTCCCATGCTGGTGGCTGAATTAAAACAGTGGGGATTAGAGATTGCCTCAGAA
AAGGTTCAAATTGCAGATACAGGCCTCTTTTTGGGCTCAAAGATTACTCCCAAAAATATAGTTCCTCAGAAAATAGAAATTCGCAAAGACCACTTACAAACATTAAATGATTTTCAGAAACTTCTGGGAGATATAAATTGGTTGAGGCCCTTTTTAAAAATTCCATCTGCTGATCTAAAACCCCTCTTCGATCTCCTGGAGGGTGAGCCTCATATCTCCTCACCCA
GAAAATTTACCCCTGCCGCCCACCGAGCCTTACAGATGGTGGAAGAGGCCTTACAGGAAGCACAATTACAACGAATTGAGGAGTCACTACCTTTTAATCTGTGTGTTTTTAAGACAGCTCAATTGCCAACTGCAGTCCTGTGGCAACATGGACCATTACTTTGGATTCATCCAAATGCTTCCCCTGCTAAGATTATTGACTGGTACCCTGATGCTGTTGCACAGCT
CGCGCTTCGCGGAATAAAAGCAGCTGTTACACATTTTGGCAGGGATCCTGATTCCTTGATTGTGCCTTATACTGCTGCACAAGTTCAGACCTTGGCAGCCACATCTAGTGATTGGGCAGTTTTAGTTACTTCCTTTTCAGGACAAATTGATAATCATTTTCAAAGCATCCAATTTTACAGTTTGCCCTAAACCAGGCTATAGTGTTTCCACAAGTGACAGCTAAA
GACCCACTCCCAGATGGGACTGTGGTGTACACTGATGGATCAAAAACTGGTATGGGAGCTTATGTGGTCAAGGATAGGGTTATATCTAAACAATATAATGAAACTTCACCTCAAGTTGTAGAATGTCTGATAGTACTGGAGGTTCTTGAGGCTTTTCCAGGACCCCTCAATATTGTATCAGATTCCTCTTATGTGGTTAATGCAGTTAATCTCCTTGAGATAGCTG
GAATAATAAGATCCTCCAGCAGAGTTGCTAACATCTTTCAGAAGATACAAGCTGCCTTGTCAAATAGGAGATTTCCTGTTTTCATTACCCATGTCCGAGCACATTCTGGACTCCCAGGTCCCATGTCCTTAGGGAATGACTTAGCGGACAAGGCTACAAAGCTGGTGGCCGCTGCCTTGTCTACCCATGCACAAGCAGCAAAAGAATTCCATAAGCGCTTTCATGT
GACGGCTGAGACCTTGCGCCGTCGTTTTGCTCTATCCAGGAAAGAGGCTAGGGAAATTGTTACTCAATGCCAGAATTGCTGCGAATTCTTACCTACTCCCCACATGGGAATAAACCCACGTGGCATTAGACCATTGCAGATGTGGCAGATGGATGTTACTCATATTCCCTCTTTTGGAAGGCTTCAATATGTTCATGTTTCTGTTGACACCTGTTCTGGCGTAATG
TTTGCCACACCCCTAACAGGGGAAAAGGCCTCATATGTGATTCAACATTGCCTTGAGGCCTGGAGTGCTTGGGGAAAACCCAGAATTTTAAAAACAGACAATGGACCAGCATATACCTCTCAAAAATTCCGACAGTTTTGTCGGCAAATGGATGTTACCCATCTAACTGGCCTACCTTATAACCCTCAAGGACAGGGCATTGTGGAACGTGCCCATCGTATACTCA
AATCATATCTGATTAAACAAAAGGGGAGTATTGAGGATGTTCTACCCTCGGTACCAAGAGTTGCTGTATCCATGGCACTTTTTACCCTTAATTTCTTGAACTGTGATGCCCAAGGCCACACTGCGGCTGATCGCCACAGTTTAGAGCCTGATAGGCCAAAAGAAATGGTAAAATGGAAAGATGTCTTGACTGATCTCTGGAAAGGCCCGGATCCTATTCTTATAAG
ATCCAGGGGAGCTGTGTGTGTTTTTCCACAGGAGGAAGAAAACCCCCTGTGGATCCCTGAAAGACTCACTCGAAGAGCTCCTTCACAACTCCAGAATAAGAAGGATGGAGGCCAAGTGGGTGATGAAGACTCTCCTACTCGGGATGATGGTTCCTAGTATGATGGGAGAGTTACGGTGGGCCATCATGTCTACCTTCCCCCTACCGATGCCAGTGATGCAGAATGC
CCAGGTGTTCCCTAGATTTTTCACAAATAATAGAGAGTTGGGGCTTGCCTTTTTGCCTTTAGATCCTCAGATACAGAGTTTTCAGGATGAGAGAACCTTGTCATTAGTAGGAAGCTTGTGCTTTACTGTGATTTCTTCATTAAAGGATAATTTTATTCATATGGATCCAGAAAGATCTAATGCTAATCCTTTTGAATTAATGGAAGCTGTACTGATCTTTCTCCTA
TGATAGTGAGAAGGGACAGATTACTTTTGATTGGAAAGGGGCATTAGGCAACAGTCTGAGTGAGGGACAGAGCTCTGGATCATCTGATTGGTCCTCTCCAATGTTAAGTATACCCCTTTTAAGAGAACTAATTATTCAGCTGCCCTTTATTTGGGTTGTGAGTAATGATACCTTAGGTAAGAATCAGAAAGAAATAAATTGTTCTGCTTAGAATTGTTTTTATGC
ATTATGCTGGGATGCTATTAAGCACCCTTTTGCTTTGGTAATGCGCATGCCTCGATTTATTCCAGCTCCTGTTGAGGCGCCAAGTAATTTGAGTTTGTTTAGGGAAAAGCGAGATTTTTGGGATCTCTGCTATTATTGTGGGCCTGATAGCCACCGCCGCGGTTACTGCTTCGGTTACCGCCTCGGCCCTTGCACTTTCTGCCACTGTGCAGACAACTCAAACCAT
TAATGAACTATAGGCCACTGTGACCATGGTCCTAGATAAACAGGCCACTGCCAATTCTCAGATACAAGGAGGCCTGATGTTGGTAAACCAACAGATTAATTTGGTTCAGGAACAATTGGATATATTGTGGCAGATGGCCAAATTGGGATGTGAACAAAGTTGCCTGGCCTTTGTGTCACTTAGTACAATATGAGAATTTTACAAGAGCAGCTAACCTGTCAAAAGC
TCTGTCTCAACATTTATTACAGAATTGGACTTTTGAGTTTGAGCAGACACTCCGTGAGTTGAGAATGGCGATCCTGCAAGTGAATTCGACGCATCTGGATCTTTCTCTGATGGAAGACCTCTCCCCCTGGATTTCATCTGCATTTTCTTATTTTAAGGAATGGGTGGGGGTAGGATCATTTAGCCTAGTTCTCTGCTGTGGAATAGTGTTGATGCTTTGGATGGTG
TGTACGCTCAGAGCCCAAACAAAAGGAGAAAGTGGTGGTGGCACAAGCACTTGCTGCCTTAGAACACGGAGCATCCCCTGACATTTGGTTAACCATGTTAAAGCAATAGGTCGCTGGCCTCCCAGCTCTTGCACCCCACGAGGCTAGTCTCATTGCACGGGATAGAGTGGGTAGGCTTCAGCAGCCCAAGGAGTTGCAAGGCTAAGCACTGCACAGGAGAGGTCTG
CGGTATAACGACTTTCTCCTGGGAGATAAGTCATCTTGCATGAAGGTTCTATGTCATGATCTCCTTCCCCCAGAAAAAAGACATCGGACTGGTCAGGACTTCCTCTGGGGATAAGACCCTGGAGAGGAGCTTTGTACATGTTCCGAATTTGCACATGGGGATTTGACCTCTATCTCCACTCCAAAGTTGTGGGTGTCCTATTGCTTCAAAAATAAATGAATAGGGG
GAGATGTGAGGAGCCGCCCTCGCTATCGCTATTGCCGTTAGAAGATGGCGCTGACATCCACTGTCAGTTGGAGTTAACTGTCGCTGTCAGTTGGAGTTAACTGTCGCTGTCAGTTAGAGTTAACTGTCGCTGTCAGTTGGAGTTAACCGTTTAAACTGTGCCTCTCCCGTGACGTCATCTGGGGTGATGTGCAAACCACCAAT
CCCGGCTCTACACATCTCACTCGGAGCTCCTAGGCTTATATAAGGGGCTGGGTTTCTTAGCTTGGGGTCTCCCTCTAAGAAGCTGATCATCTATCTCTCAAGATGCATTAAAGCTTTACTGCAGAAGGATCCGAGTGTTCTGCGTCGTTCTTGCTGGCGAGACGGTAGCGCGGGACA

>IAPLTR3.373814
TGTGAGGAGCCGCCCTCGCTATCGCTATTGCCGTTAGAAGATGGCGCTGACATCCACTGTCAGTTGGAGTTAACTGTCGCTGTCAGTTGGAGTTAACTGTCGCTGTCAGTTGGAGTTAACCGTTTAAACTGTGCCTCTCCCGTGACGTCATCTGGGGTGATGTGCAAACCACCAATCCCGGCTCTACACATCTCACTCGGAGCTCCTAGGCTTATATAAGGGGCTG
GGTTTCTTAGCTTGGGGTCTCCCTCTAAGAAGCTGATCATCTATCTCTCAAGATGCATTAAAGCTTTACTGCAGAAGGATCCGAGTGTTCTGCGTCGTTCTTGCTGGCGAGACGGTAGCGCGGGACATCTGGTGCCGAAACCCGGGAACTCTTCAACATCGCCGGCGCCGCGGGAGACCCCTCGGAAAACGGGGCGGATTCAGAACTGCAGGGACGTAGGTTCAGA
GAGGTATGCTTTATCCTGAACAACCCTTTTCTCGACTTTGGCCTTAGTTTGTCATCACCGTGGGACGGGTCAGTGGAGGCTTTGGTGCTAGTCCTGTTTGTCCTTCTCCTTCATCGTGGCTGGTGCTGACAGAACGGCGCTGTCCGTCTGGCGCTGTCCGTCTGGCGCTGTCCGTCTGGCGCTGTCCGTCTGGCGCTGTCCGTCTGGCGCTGTCCGTCTGGCGCTG
TCCATCTGGCGCTGTCCATCTGGCGCTGTCAGTCTGGTGCTGTCAGTCTGGTGCTGTCAGTCTGGTGCTGTCAGTCTGGTAACGGAGTTGAGCGCGTTTTTAGATAAGCTGTCAGCGCGATGGGGTCGTCACAGTCAGTGGTTACAGCCTTACAGACTGTGCTAAAACAAAGAGATCTTAAAATTGCACCACGAACACTTCAAAATTTTGTGAAGGAGGTAGATA
GAGTGGCACCATGGTATGCTTGTTCCGGGTCCCTCACTGTGGCCTCATGGAACAAGCTAGGTAAAGAACTAGACAGGAAGCATGCAGAAGGAGATCTCTGCTTAGGTACCAAAGCCATTTGGAAGCTAGTTAAAAACTGCTTGGAGGATGAGGCCTGTCACCCTGCTATAATAGAAAGTCAGGGAACCTTAGAGGAAGTTCAGGATAGCATGTCAGAAACTGAACG
GAGTGAGAGAATGGGAGCTCGCAAAAGAAAAGACATGTCTAAGAAAAAAGGCCCTCCCCAGGAAGTTAAGAAAGGGGGAGAGAAAGAAGGGAGTTATCATTCACCCCTTAACAAATCTAAGAAAAAGAAAAAGCCAGAATCTAGTCTATACCCTGCAGTAGAGCTAGAAGCCTTGGAGCTAGATAATTCTGATTCTGACACTCTGGATTCTAGCGAAGAAGGTGGT
CTAGAGGAGGAGGTGGCACGGTATGAGGAAAAAAGATATCACCCTGATAGACACCGGCCACTGAAAACAAAAATGAATGTGAGGCCACCGCCTATAAATCCGGCGGGCTCACGCCCCTCAGCGCCTCCGCCATATGAGCTTCGGCTGAATACTGGTACTGACTCCTTTTTACCTTTAGAGGAACGGAGAAAAATACAAATGGCTTTTCCAGTATTCGAGAATGTGG
AAGGCGGAAGAGTACATACCCCCGTAGATTACAATCAAATTAAAGAATTGGCCGAATCCGTTCGTAATTATGGGGTCAATGCTAACTTTACCACATTACAGGTGGAAAGGCTTGCTAATTTTGCCATGACTCCCACTGACTGGCAAACGACAGTGAAAGCAGTGCTTCCCAACATGGGACAATACATGGAATGGAAGGCTCTCTGGTATGATGCAGCCCAGGCACA
AGCTAGAGTTAATGCCACGGCTGAAGATGAGAATCAGAGGCAGTGGACATTTGAAATGCTGACAGGTCAGGGTCAACATGCCCTTAATCAAACCAACTACATCTGGGGCGTCTATGGTCAGGTGTCGGCCGCCGCTATTAAAGCATGGAAGGCCCTCACAAAAAGGGGAGAGTCCAGTGGACACCTTACAAAGATTGTCCAAGGACCCCAAGAGCCATTTTCAGAC
TTTGTGGCCAGAATGACAGAGGCCGCCAGTCGCATCTTTGGTGATTCAGAACAAGCTATGCCCCTGATTGAGCAACTTGTCTATGAACAAGCAACCCAGGAATGTCGAGCGGCTATCGCCCCTCGCAAAAGCAAAGGTTTACAAGATTGGCTCAGAGTGTGCAGGGAGCTGGGGGGACCCCTAACCAATGCAGGACTAGCTGCCGCCATTTTACAAACGCACAGGT
ATAGAGATTTATCTAATCGAAAAGCTTGTTTTAATTGTGGGAGAATGGGGCACCTTAAGAAAGATTGTCAAGCCCCTGAAAGAACAAGAGAATCGAAGCTTTGCTATCATTGTGGTAAAGGTTATCATAGGGCTAGCGAATGCAGGTCAGTCCGGGATGTAAAAGGGAGGCTTCTGCCCCCTATTGAAGAATCCAAGGTACCCCAATCAAAAAACGGGTCACGGGG
CCCATGGTCCCAGGGCCCTCAGAAATATGGGAACCAATTTCAGAAAAGCAATCAAGAGGAAGTAGAGGTTTCCGAGGAACCTCCGGAATGGACTTGCGTGCCGCCTCCGACTTCTTACTCATGCCCCAAATGAATGTTCAACCGGTCCCAGTCCATTCCCCTGGACCTTTGCCCCCTGCCACCATTGGGCTTATTCTGGGCCGTGGCTCCTTAACTTTACAGGGAC
TCATTATATATCCTGGGATTGTTGACCCATATCATAAAGAGGAGATTCAAGTTCTCTGTTCTAGCCCTCGGGGTGTATTTTCTATAAAGCAGGGAGATAGAATTGCACAACTGGTGTTGCTGCCCTCCCTCGGGGATGGAGAAACCTACACTTTACAAAAAAGGGCTATGGGATCTTCTGGAAGTGATTCAGCCTATCTGGCTATACCCCTAAATGAGAGACCTAC
AATAAAATTGTTAATTAATGGAAAAGAATTTGAAGGGATAATGGATTCGGGAGCAGACAAAAGCATTATTTCACTCCATTGGTGGCCGAAGTCTTGGCCCACTGTTGTTTCATCTCATTCTCTACAAGGTCTTGGATACCAGTCCTCTCCTGCCATCAGTGCCTCAGCCTTAACCTGGCGGGATGCTGAAGGCAAACAGGGATGTTTTACCCCCTATGTGTTGCCA
CTCCCTGTAAATTTATGGGGACGAGATGTGTTACAAGCCATGGGCATGACCCTAACTAATGAATACTCCCCCAGGCATCAGCCATTATGGCAAAGATGGGGTATACAAATGGTAGAGGCTTGGGTAGGCAAGAACAAGGCAGAATAAAACCCATTACACAACACGGAAATCGGGGTAGAAAAGGACTGGGTTTTATTTAGGGGCCGTTGAGGCTTCACGACCCAT
ACCATGGAAAACAGAGGAGCCGTTATGGGTCTCTCAATGGCCTCTATCCTCTGAAAAATTAGAGGCTGTCACAAGATTAGTGCAAGAACAGGAACGGCTGGGGCATCTAGAGCCTTCTACCTCCCCATGGAATACACCAATTTTTGTTATTAAGAAGAAATCTGGGAAGTGGAGATTACTCCACGACCTGCGGGCCATTAACAATCAGATGCATCTTTTGGCCCT
GTTCAAAGAGGCCTTCCTTTGCTTTCTGCACTTCCTCAAGATTGGAAGCTTATTATTATAGATATTAAGGATTGTTTCTTCTCTATTCCACTTTACCCACGGGATAGACCAAGGTTTGCCTTCACTATCCCTTCTCTTAATCATATGGAACCAGACAAGAGATTTCAGTGGAAGGTACTGCCGCAAGGCATGGCCAATAGCCCAACAATATGTCAGCTATATGTGC
AGGAGGCTTTGGAGCCAATTAGGAAGCAATTTACATCTTTAATCGTTATCCATTATATGGACGATATTCTTATTTGTCACAAAGAGTTAGATGTTTTACAAAGGCTTTTCCCATGCTGGTGGCTGAATTAAAACAGTGGGGATTAGAGATTGCCTCAGAAAAGGTTCAAATTGCAGATACAGGGCTCTTTTTGGGCTCAAAGATTACTCCCAAAAATATAGTTCC
TCAGAAAATAGAAATTCGCAAAGACCACTTACAAACATTAAATGATTTTCAGAAACTTCTGGGAGATATAAATTGGTTGAGGCCCTTTTTAAAAATTCCATCTGCTGATCTAAAACCCCTCTTCGATCTCCTGGAGGGTGAGCCTCATATCTCCTCACCCAGAAAATTTACCCCTGCCGCCCACCGAGCCTTACAGATGGTGGAAGAGGCCTTACAGGAAGCACAA
TTACAACGAATTGAGGAGTCACTACCTTTTAATCTGTGTGTTTTTAAGACAGCTCAATTGCCAACTGCAGTCCTGTGGCAACATGGACCATTACTTTGGATTCATCCAAATGCTTCCCCTGCTAAGATTATTGACTGGTACCCTGATGCTGTTGCACAGCTCGCGCTTCGCGGAATAAAAGCAGCTGTTACACATTTTGGCAGGGATCCTGATTCCTTGATTGTGC
CTTATACTGCTGCACAAGTTCAGACCTTGGCAGCCACATCTAGTGATTGGGCAGTTTTAGTTACTTCCTTTTCAGGACAAATTGATAATCATTTTCAAAGCATCCAATTTTACAGTTTGCCCTAAACCAGGCTATAGTGTTTCCACAAGTGACAGCTAAAGACCCACTCCCAGATGGGACTGTGGTGTACACTGATGGATCAAAAACTGGTATGGGAGCTTATGT
GGTCAAGGATAGGGTTATATCTAAACAATATAATGAAACTTCACCTCAAGTTGTAGAATGTCTGATAGTACTGGAGGTTCTTGAGGCTTTTCCAGGACCCCTCAATATTGTATCAGATTCCTCTTATGTGGTTAATGCAGTTAATCTCCTTGAGATAGCTGGAATAATAAGATCCTCCAGCAGAGTTGCTAACATCTTTCAGAAGATACAAGCTGCCTTGTTAAAT
AGGAGATTTCCTGTTTTCATTACCCATGTCCGAGCACATTCTGGACTCCCAGGTCCCATGTCCTTAGGGAATGACTTAGCGGACAAGGCTACAAAGCTGGTGGCCGCTGCCTTGTCTACCCATGCACAAGCAGCAAAAGAATTCCATAAGCGCTTTCATGTGACGGCTGAGACCTTGCGCCGTCGTTTTGCTCTATCCAGGAAAGAGGCTAGGGAAATTGTTACTC
AATGCCAGAATTGCTGCGAATTCTTACCTACTCCCCACATGGGAATAAACCCACGTGGCATTAGACCATTGCAGATGTGGCAGATGGATGTTACTCATATTCCCTCTTTTGGAAGGCTTCAATATGTTCATGTTTCTGTTGACACCTGTTCTGGCGTAATGTTTGCCACACCCCTAACAGGGGAAAAGGCCTCATATGTGATTCAACATTGCCTTGAGGCCTGGAG
TGCTTGGGGAAAACCCAGAATTTTAAAAACAGACAATGGACCAGCATATACCTCTCAAAAATTCCGACAGTTTTGTCGGCAAATGGATGTTACCCATCTAACTGGCCTACCTTATAACCCTCAAGGACAGGGCATTGTGGAACGTGCCCATCGTACACTCAAATCATATCTGATTAAACAAAAGGGGAGTATTGAGGATGTTCTACCCTCGGTACCAAGAGTTGCT
GTATCCATGGCACTTTTTACCCTTAATTTCTTGAACTGTGATGCCCAAGGCCACACTGCGGCTGATCGCCACAGTTTAGAGCCTGATAGGCCAAAAGAAATGGTAAAATGGAAAGATGTCTTGACTGATCTCTGGAAAGGCCCGGATCCTATTCTTATAAGATCCAGGGGAGCTGTGTGTGTTTTTCCACAGGAGGAAGAAAACCCCCTGTGGATCCCTGAAAGAC
TCACTCGAAGAGCTCCTTCACAACTCCAGAATAAGAAGGATGGAGGCCAAGTGGGTGATGAAGACTCTCCTACTCGGGATGATGGTTCCTAGTATGATGGGAGAGTTACGGTGGGCCATCATGTCTACCTTCCCCCTACTGATGCCAGTGATGCAGAATGCCCAGGTGTTCCCTAGATTTTTCACAAATAATAGAGAGTTGGGGCTTGCCTTTTTGCCTTTAGATC
CTCAGATACAGAGTTTTCAGGATGAGAGAACCTTGTCATTAGTAGGAAGCTTGTGCTTTACTGTGATTTCTTCATTAAAGGATAATTTTATTCATATGGATCCAGAAAGATCTAATGCTAATCCTTTTGAATTAATGGAAGCTGTACTGATCTTTCTCCTATGATAGTGAGAAGGGACAGATTACTTTTGATTGGAAAGGGGCATTAGGCAACAGTCTGAGTGAGG
GACAGAGCTCTGGATCATCTGATTGGTCCTCTCCAATGTTAAGTATACCCCTTTTAAGAGAACTAATTATTCAGCTGCCCTTTATTTGGGTTGTGAGTAATGATACCTTAGGTAAGAATCAGAAAGAAATAAATTGTTCTGCTTAGAATTGTTTTTATGCATTATGCTGGGATGCTATTAAGCACCCTTTTGCTTTGGTAATGCGCATGCCTCGATTTATTCCAG
CTCCTGTTGAGGCGCCAAGTAATTTGAGTTTGTTTAGGGAAAAGCGAGATTTTTGGGATCTCTGCTATTATTGTGGGCCTGATAGCCACCGCCGCGGTTACTGCTTCGGTTACCGCCTCGGCCCTTGCACTTTCTGCCACTGTGCAGACAACTCAAACCATTAATGAACTATAGGCCACTGTGACCATGGTCCTAGATAAACAGGCCACTGCCAATTCTCAGATAC
AAGGAGGCCTGATGTTGGTAAACCAACAGATTAATTTGGTTCAGGAACAATTGGATATATTGTGGCAGATGGCCAAATTGGGATGTGAACAAAGTTGCCTGGCCTTTGTGTCACTTAGTACAATATGAGAATTTTACAAGAGCAGCTAACCTGTCAAAAGCTCTGTCTCAACATTTATTACAGAATTGGACTTTTGAGTTTGAGCAGACACTCCGTGAGTTGAGAA
TGGCGATCCTGCAAGTGAATTCGACGCATCTGGATCTTTCTCTGATGGAAGACCTCTCCCCCTGGATTTCATCTGCATTTTCTTATTTTAAGGAATGGGTGGGGGTAGGATCATTTAGCCTAGTTCTCTGCTGTGGAATAGTGTTGATGCTTTGGATGGTGTGTACGCTCAGAGCCCAAACAAAAGGAGAAAGTGGTGGTGGCACAAGCACTTGCTGCCTTAGAAC
ACGGAGCATCCCCTGACATTTGGTTAACCATGTTAAAGCAATAGGTCGCTGGCCTCCCAGCTCTTGCACCCCACGAGGCTAGTCTCATTGCACGGGATAGAGTGGGTAGGCTTCAGCAGCCCAAGGAGTTGCAAGGCTAAGCACTGCACAGGAGAGGTCTGCGGTATAACGACTTTCTCCTGGGAGATAAGTCATCTTGCATGAAGGTTCTATGTCATGATCTCCT
TCCCCCAGAAAAAAGACATCGGACTGGTCAGGACTTCCTCTGGGGATAAGACCCTGGAGAGGAGCTTTGTACATGTTCCGAATTTGCACATGGGGATTTGACCTCTATCTCCACTCCAAAGTTGTGGGTGTCCTATTGCTTCAAAAATAAATGAATAGGGGGAGATGTGAGGAGCCGCCCTCGCTATCGCTATTGCCGTTAGAAGATGGCGCTGACATCCACTGT
CAGTTGGAGTTAACTGTCGCTGTCAGTTGGAGTTAACTGTCGCTGTCAGTTGGAGTTAACCGTTTAAACTGTGCCTCTCCCGTGACGTCATCTGGGGTGATGTGCAAACCACCAATCCCGGCTCTACACATCTCACTCGGAGCTCCTAGGCTTATATAAGGGGCTGGGTTTCTTAGCTTGGGGTCTCCCTCTAAGAAGCTGATCATCTATCTCTCAAGATGCATTA
AAGCTTTACTGCAGAAGGATCCGAGTGTTCTGCGTCGTTCTTGCTGGCGAGACGGTAGCGCGGGACA

>IAPLTR3.1238823
TGTGAGGAGCCGCCCTCGCTATCGCTATTGCCGTTAGAAGATGGCGCTGACATCCACTGTCAGTTGGAGTTAACTGTCGCTGTCAGTTGGAGTTAACTGTCGCTGTCAGTTGGAGTTAACCGTTTAAACTGTGCCTCTCCCGTGACGTCATCTGGGGTGATGTGCAAACCACCAATCCCGGCTCTACACATCTCACTCGGAGCTCCTAGGCTTATATAAGGGGCTG
GGTTTCTTAGCTTGGGGTCTCCCTCTAAGAAGCTGATCATCTATCTCTCAAGATGCATTAAAGCTTTACTGCAGAAGGATCCGAGTGTTCTGCGTCGTTCTTGCTGGCGAGACGGTAGCGCGGGACATCTGGTGCCGAAACCCGGGAACTCTTCAACATCGCCGGCGCCGCGGGAGACCCCTCGGAAAACGGGGCGGATTCAGAACTGCAGGGACGTAGGTTCAGA
GAGGTATGCTTTATCCTGAACAACCCTTTTCTCGACTTTGGCCTTAGTTTGTCATCACCGTGGGACGGGTCAGTGGAGGCTTTGGTGCTAGTCCTGTTTGTCCTTCTCCTTCATCGTGGCTGGTGCTGACAGAACGGCGCTGTCCGTCTGGCGCTGTCCGTCTGGCGCTGTCCATCTGGCGCTGTCCATCTGGCGCTGTCAGTCTGGTGCTGTCAGTCTGGTGCTG
TCAGTCTGGTGCTGTCAGTCTGGTAACGGAGTTGAGCGCGTTTTTAGATAAGCTGTCAGCGCGATGGGGTCGTCACAGTCAGTGGTTACAGCCTTACAGACTGTGCTAAAACAAAGAGATCTTAAAATTGCACCACGAACACTTCAAAATTTTGTGAAGGAGGTAGATAGAGTGGCACCATGGTATGCTTGTTCCGGGTCCCTCACTGTGGCCTCATGGAACAAG
CTAGGTAAAGAACTAGACAGGAAGCATGCAGAAGGAGATCTCTGCTTAGGTACCAAAGCCATTTGGAAGCTAGTTAAAAACTGCTTGGAGGATGAGGCCTGTCACCCTGCTATAATAGAAAGTCAGGGAACCTTAGAGGAAGTTCAGGATAGCATGTCAGAAACTGAACGGAGTGAGAGAATGGGAGCTCGCAAAAGAAAAGACATGTCTAAGAAAAAAGGCCCTC
CCCAGGAAGTTAAGAAAGGGGGAGAGAAAGAAGGGAGTTATCATTCACCCCTTAACAAATCTAAGAAAAAGAAAAAGCCAGAATCTAGTCTATACCCTGCAGTAGAGCTAGAAGCCTTGGAGCTAGATAATTCTGATTCTGACACTCTGGATTCTAGCGAAGAAGGTGGTCTAGAGGGGGAGGTGGCACGGTATGAGGAAAAAAGATATCACCCTGATAGACACCG
GCCACTGAAAACAAAAATGAATGTGAGGCCACCGCCTATAAATCCGGCGGGCTCACGCCCCTCAGCGCCTCCGCCATATGAGCTTCGGCTGAATACTGGTACTGACTCCTTTTTACCTTTAGAGGAACGGAGAAAAATACAAATGGCTTTTCCAGTATTCGAGAATGTGGAAGGCGGAAGAGTACATACCCCCGTAGATTACAATCAAATTAAAGAATTGGCCGAA
TCCGTTCGTAATTATGGGGTCAATGCTAACTTTACCACATTACAGGTGGAAAGGCTTGCTAATTTTGCCATGACTCCCACTGACTGGCAAACGACAGTGAAAGCAGTGCTTCCCAACATGGGACAATACATGGAATGGAAGGCTCTCTGGTATGATGCAGCCCAGGCACAAGCTAGAGTTAATGCCACGGCTGAAGATGAGAATCAGAGGCAGTGGACATTTGAAA
TGCTGACAGGTCAGGGTCAACATGCCCTTAATCAAACCAACTACATCTGGGGCGTCTATGGTCAGGTGTCGGCCGCCGCTATTAAAGCATGGAAGGCCCTCACAAAAAGGGGAGAGTCCAGTGGACACCTTACAAAGATTGTCCAAGGACCCCAAGAGCCATTTTCAGACTTTGTGGCCAGAATGACAGAGGCCGCCAGTCGCATCTTTGGTGATTCAGAACAAGC
TATGCCCCTGATTGAGCAACTTGTCTATGAACAAGCAACCCAGGAATGTCGAGCGGCTATCGCCCCTCGCAAAAGCAAAGGTTTACAAGATTGGCTCAGAGTGTGCAGGGAGCTGGGGGGACCCCTAACCAATGCAGGACTAGCTGCCGCCATTTTACAAACGCACAGGTATAGAGATTTATCTAATCGAAAAGCTTGTTTTAATTGTGGGAGAATGGGGCACCTT
AAGAAAGATTGTCAAGCCCCTGAAAGAACAAGAGAATCGAAGCTTTGCTATCGTTGTGGTAAAGGTTATCATAGGGCTAGCGAATGCAGGTCAGTCCGGGATGTAAAAGGGAGGCTTCTGCCCCCTATTGAAGAATCCAAGGTACCCCAATCAAAAAACGGGTCACGGGGCCCATGGTCCCAGGGCCCTCAGAAATATGGGAACCAATTTCAGAAAAGCAATCAAG
AGGAAGTAGAGGTTTCCGAGGAACCTCCGGAATGGACTTGCGTGCCGCCTCCGACTTCTTACTCATGCCCCAAATGAATGTTCAACCGGTCCCAGTCCATTCCCCTGGACCTTTGCCCCCTGCCACCATTGGGCTTATTCTGGGCCGTGGCTCCTTAACTTTACAGGGACTCATTATATATCCTGGAATTGTTGACCCGTATCATAAAGAGGAGATTCAAGTTCTC
TGTTCTAGCCCTCGGGGTGTATTTTCTATAAAGCAGGGAGATAGAATTGCACAACTGGTGTTGCTGCCCTCCCTCGGGGATGGAGAAACCTACACTTTACAAAAAAGGGCTATGGGATCTTCTGGAAGTGATTCAGCCTATCTGGCTATACCCCTAAATGAGAGACCTACAATAAAATTGTTAATTAATGGAAAAGAATTTGAAGGGATAATGGATTCGGGAGCAG
ACAAAAGCATTATTTCACTCCATTGGTGGCCGAAGTCTTGGCCCACTGTTGTTTCATCTCATTCTCTACAAGGTCTTGGATACCAGTCCTCTCCTGCCATCAGTGCCTCAGCCTTAACCTGGCGGGATGCTGAAGGCAAACAGGGATGTTTTACCCCCTATGTGTTGCCACTCCCTGTAAATTTATGGGGACGAGATGTGTTACAAGCCATGGGCATGACCCTAAC
TAATGAATACTCCCCCAGGCATCAGCCATTATGGCAAAGATGGGGTATACAAATGGTAGAGGCTTGGGTAGGCAAGAACAAGGCAGAATAAAACCCATTACACAACACGGAAATCGGGGTAGAAAAGGACTGGGTTTTATTTAGGGGCCGTTGAGGCTTCACGACCCATACCATGGAAAACAGAGGAGCCGTTATGGGTCTCTCAATGGCCTCTATCCTCTGAAA
AATTAGAGGCTGTCACAAGATTAGTGCAAGAACAGGAACGGCTGGGGCATCTAGAGCCTTCTACCTCCCCATGGAATACACCAATTTTTGTTATTAAGAAGAAATCTGGGAAGTGGAGATTACTCCACGACCTGCGGGCCATTAACAATCAGATGCATCTTTTGGCCCTGTTCAAAGAGGCCTTCCTTTGCTTTCTGCACTTCCTCAAGATTGGAAGCTTATTAT
TATAGATATTAAGGATTGTTTCTTCTCTATTCCACTTTACCCACGGGATAGACCAAGGTTTGCCTTCACTATCCCTTCTCTTAATCATATGGAACCAGACAAGAGATTTCAGTGGAAGGTACTGCCGCAAGGCATGGCCAATAGCCCAACAATATGTCAGCTATATGTGCAGGAGGCTTTGGAGCCAATTAGGAAGCAATTTACATCTTTAATCGTTATCCATTAT
ATGGACGATATTCTTATTTGTCACAAAGAGTTAGATGTTTTACAAAGGCTTTTCCCATGCTGGTGGCTGAATTAAAACAGTGGGGATTAGAGATTGCCTCAGAAAAGGTTCAAATTGCAGATACAGGCCTCTTTTTGGGCTCAAAGATTACTCCCAAAAATATAGTTCCTCAGAAAATAGAAATTCGCAAAGACCACTTACAAACATTAAATGATTTTCAGAAAC
TTCTGGGAGATATAAATTGGTTGAGGCCCTTTTTAAAAATTCCATCTGCTGATCTAAAACCCCTCTTCGATCTCCTGGAGGGTGAGCCTCATATCTCCTCACCCAGAAAATTTACCCCTGCCGCCCACCGAGCCTTACAGATGGTGGAAGAGGCCTTACAGGAAGCACAATTACAACGAATTGAGGAGTCACTACCTTTTAATCTGTGTGTTTTTAAGACAGCTCA
ATTGCCAACTGCAGTCCTGTGGCAACATGGACCATTACTTTGGATTCATCCAAATGCTTCCCCTGCTAAGATTATTGACTGGTACCCTGATGCTGTTGCACAGCTCGCGCTTCGCGGAATAAAAGCAGCTGTTACACATTTTGGCAGGGATCCTGATTCCTTGATTGTGCCTTATACTGCTGCACAAGTTCAGACCTTGGCAGCCACATCTAGTGATTGGGCAGTT
TTAGTTACTTCCTTTTCAGGACAAATTGATAATCATTTTCAAAGCATCCAATTTTACAGTTTGCCCTAAACCAGGCTATAGTGTTTCCACAAGTGACAGCTAAAGACCCACTCCCAGATGGGACTGTGGTGTACACTGATGGATCAAAAACTGGTATGGGAGCTTATGTGGTCAAGGATAGGGTTATATCTAAACAATATAATGAAACTTCACCTCAAGTTGTAG
AATGTCTGATAGTACTGGAGGTTCTTGAGGCTTTTCCAGGACCCCTCAATATTGTATCAGATTCCTCTTATGTGGTTAATGCAGTTAATCTCCTTGAGATAGCTGGAATAATAAGATCCTCCAGCAGAGTTGCTAACATCTTTCAGAAGATACAAGCTGCCTTGTTAAATAAGAGATTTCCTGTTTTCATTACCCATGTCCGAGCACATTCTGGACTCCCAGGTCC
CATGTCCTTAGGGAATGACTTAGCGGACAAGGCTACAAAGCTGGTGGCCGCTGCCTTGTCTACCCATGCACAAGCAGCAAAAGAATTCCATAAGCGCTTTCATGTGACGGCTGAGACCTTGCGCCGTCGTTTTGCTCTATCCAGGAAAGAGGCTAGGGAAATTGTTACTCAATGCCAGAATTGCTGCGAATTCTTACCTACTCCCCACATGGGAATAAACCCACGT
GGCATTAGACCATTGCAGATGTGGCAGATGGATGTTACTCATATTCCCTCTTTTGGAAGGCTTCAATATGTTCATGTTTCTGTTGACACCTGTTCTGGCGTAATGTTTGCCACACCCCTAACAGGGGAAAAGGCCTCATATGTGATTCAACATTGCCTTGAGGCCTGGAGTGCTTGGGGAAAACCCAGAATTTTAAAAACAGACAATGGACCAGCATATACCTCTC
AAAAATTCCGACAGTTTTGTCGGCAAATGGATGTTACCCATCTAACTGGCCTACCTTATAACCCTCAAGGACAGGGCATTGTGGAACGTGCCCATCGTACACTCAAATCATATCTGATTAAACAAAAGGGGAGTATTGAGGATGTTCTACCCTCGGTACCAAGAGTTGCTGTATCCATGGCACTTTTTACCCTTAATTTCTTGAACTGTGATGCCCAAGGCCACAC
TGCGGCTGATCGCCACAGTTTAGAGCCTGATAGGCCAAAAGAAATGGTAAAATGGAAAGATGTCTTGACTGATCTCTGGAAAGGCCCGGATCCTATTCTTATAAGATCCAGGGGAGCTGTGTGTGTTTTTCCACAGGAGGAAGAAAACCCCCTGTGGATCCCTGAAAGACTCACTCGAAGAGCTCCTTCACAACTCCAGAATAAGAAGGATGGAGGCCAAGTGGGT
GATGAAGACTCTCCTACTCGGGATGATGGTTCCTAGTATGATGGGAGAGTTACGGTGGGCCATCATGTCTACCTTCCCCCTACCGATGCCAGTGATGCAGAATGCCCAGGTGTTCCCTAGATTTTTCACAAATAATAGAGAGTTGGGGCTTGCCTTTTTGCCTTTAGATCCTCAGATACAGAGTTTTCAGGATGAGAGAACCTTGTCATTAGTAGGAAGCTTGTGC
TTTACTGTGATTTCTTCATTAAAGGATAATTTTATTCATATGGATCCAGAAAGATCTAATGCTAATCCTTTTGAATTAATGGAAGCTGTACTGATCTTTCTCCTATGATAGTGAGAAGGGACAGATTACTTTTGATTGGAAAGGGGCATTAGGCAACAGTCTGAGTGAGGGACAGAGCTCTGGATCATCTGATTGGTCCTCTCCAATGTTAAGTATACCCCTTTT
AAGAGAACTAATTATTCAGCTGCCCTTTATTTGGGTTGTGAGTAATGATACCTTAGGTAAGAATCAGAAAGAAATAAATTGTTCTGCTTAGAATTGTTTTTATGCATTATGCTGGGATGCTATTAAGCACCCTTTTGCTTTGGTAATGCGCATGCCTCGATTTATTCCAGCTCCTGTTGAGGCGCCAAGTAATTTGAGTTTGTTTAGGGAAAAGCGAGATTTTTGG
GATCTCTGCTATTATTGTGGGCCTGATAGCCACCGCCGCGGTTACTGCTTCGGTTACCGCCTCGGCCCTTGCACTTTCTGCCACTGTGCAGACAACTCAAACCATTAATGAACTATAGGCCACTGTGACCATGGTCCTAGATAAACAGGCCACTGCCAATTCTCAGATACAAGGAGGCCTGATGTTGGTAAACCAACAGATTAATTTGGTTCAGGAACAATTGGAT
ATATTGTGGCAGATGGCCAAATTGGGATGTGAACAAAGTTGCCTGGCCTTTGTGTCACTTAGTACAATATGAGAATTTTACAAGAGCAGCTAACCTGTCAAAAGCTCTGTCTCAACATTTATTACAGAATTGGACTTTTGAGTTTGAGCAGACACTCCGTGAGTTGAGAATGGCGATCCTGCAAGTGAATTCGACGCATCTGGATCTTTCTCTGATGGAAGACCTC
TCCCCCTGGATTTCATCTGCATTTTCTTATTTTAAGGAATGGGTGGGGGTAGGATCATTTAGCCTAGTTCTCTGCTGTGGAATAGTGTTGATGCTTTGGATGGTGTGTACGCTCAGAGCCCAAACAAAAGGAGAAAGTGGTGGTGGCACAAGCACTTGCTGCCTTAGAACACGGAGCATCCCCTGACATTTGGTTAACCATGTTAAAGCAATAGGTCGCTGGCCTC
CCAGCTCTTGCACCCCACGAGGCTAGTCTCATTGCACGGGATAGAGTGGGTAGGCTTCAGCAGCCCAAGGAGTTGCAAGGCTAAGCACTGCACAGGAGAGGTCTGCGGTATAACGACTTTCTCCTGGGAGATAAGTCATCTTGCATGAAGGTTCTATGTCATGATCTCCTTCCCCCAGAAAAAAGACATCGGACTGGTCAGGACTTCCTCTGGGGATAAGACCCTG
GAGAGGAGCTTTGTACATGTTCCGAATTTGCACATGGGGATTTGACCTCTATCTCCACTCCAAAGTTGTGGGTGTCCTATTGCTTCAAAAATAAATGAATAGGGGGAGATGTGAGGAGCCGCCCTCGCTATCGCTATTGCCGTTAGAAGATGGCGCTGACATCCACTGTCAGTTGGAGTTAACTGTCGCTGTCAGTTGGAGTTAACTGTCGCTGTCAGTTGGAGTT
AACCGTTTAAACTGTGCCTCTCCCGTGACGTCATCTGGGGTGATGTGCAAACCACCAATCCCGGCTCTACACATCTCACTCGGAGCTCCTAGGCTTATATAAGGGGCTGGGTTTCTTAGCTTGGGGTCTCCCTCTAAGAAGCTGATCATCTATCTCTCAAGATGCATTAAAGCTTTACTGCAGAAGGATCCGAGTGTTCTGCGTCGTTCTTGCTGGCGAGACGGTA
GCGCGGGACA

>IAPLTR3.726418
TGTGAGGAGCCGCCCTCGCTATCGCTATTGCCATTAGAAGATGGCGCTGACATCCACTGTCAGTTGGAGTTAACTGTCGCTGTCAGTTGGAGTTAACCGTTTAAACTGTGCCTCTCCCGTGACGTCATCTGGGGTGATGTGCAAACCACCAATCCCGGCTCTACACATCTCACTCGGAGCTCCTAGGCTTATATAAGGGGCTGGGTTTCTTAGCTTGGGGTCTCCC
TCTAAGAAGCTGATCATCTATCTCTCAAGATGCATTAAAGCTTTACTGCAGAAGGATCCGAGTGTTCTGCGTCGTTCTTGCTGGCGAGACAGTAGCGCGGGACATCTGGTGCCGAAACCCGGGAACTCTTCAACATCGCCGGCGCCGCGGGAGACCCCTCGGAAAACGGGGCGGATTCAGAACTGCAGGGACGTAGGTTCAGAGAGGTATGCTTTATCCTGAACAA
CCCTTTTCTCGACTTTGGCCTTAGTTTGTCATCACCGTGGGACGGGTCAGTGGAGGCTTTGGTGCTAGTCCTGTTTGTCCTTCTCCTTCATCGTGGCTGGTGCTGACAGAACGGCGCTGTCCGTCTGGCGCTGTCCGTCTGGCGCTGTCCATCTGGCGCTGTCCGTCTGGCGCTGTCCGTCTGGCGCTGTCCATCTGGCGCTGTCCATCTGGCGCTGTCCATCTGG
CGCTGTCAGTCTGGTGCTGTCAGTCTGGTGCTGTCAGTCTGGTGCTGTCAGTCTGGTAACGGAGTTGAGCGCGTTTTTAGATAAGCTGTCAGCGCGATGGGGTCGTCACAGTCAGTGGTTACAGCCTTACAGACTGTGCTAAAACAAAGAGATCTTAAAATTGCACCACGAACACTTCAAAATTTTGTGAAGGAGGTAGATAGAGTGGCACCATGGTATGCTTGT
TCCGGGTCCCTCACTGTGGCCTCATGGAACAAGCTAGGTAAAGAACTAGACAGGAAGCATGCAGAAGGAGATCTCTGCTTAGGTACCAAAGCCATTTGGAAGCTAGTTAAAAACTGCTTGGAGGATGAGGCCTGTCACCCTGCTATAATAGAAAGTCAGGGAACCTTAGAGGAAGTTCAGGATAGCATGTCAGAAACTGAACGGAGTGAGAGAATGGGAGCTCGCA
AAAGAAAAGACATGTCTAAGAAAAAAGGCCCTCCCCAGGAAGTTAAGAAAGGGGGAGAGAAAGAAGGGAGTTATCATTCACCCCTTAACAAATCTAAGAAAAAGAAAAAGCCAGAATCTAGTCTATACCCTACAGTAGAGCTAGAAGCCTTGGAGCTAGATAATTCTGATTCTGACACTCTGGATTCTAGCGAAGAAGGTGGTCTAGAGGAGGAGGTGGCACGGTA
TGAGGAAAAAAGATATCACCCTGATAGACACCGGCCACTGAAAACAAAAATGAATGTGAGGCCACCGCCTATAAATCCGGCGGGCTCACGCCCCTCAGCGCCTCCGCCATATGAGCTTCGGCTGAATACTGGTACTGACTCCTTTTTACCTTTAGAGGAACGGAGAAAAATACAAATGGCTTTTCCAGTATTCGAGAATGTGGAAGGCGGAAGAGTACATACCCCC
GTAGATTACAATCAAATTAAAGAATTGGCCGAATCCGTTCGTAATTATGGGGTCAATGCTAACTTTACCACATTACAGGTGGAAAGGCTTGCTAATTTTGCCATGACTCCCACTGACTGGCAAACGACAGTGAAAGCAGTGCTTCCCAACATGGGACAATACATGGAATGGAAGGCTCTCTGGTATGATGCAGCCCAGGCACAAGCTAGAGTTAATGCCACGGCTG
AAGATGAGAATCAGAGGCAATGGACATTTGAAATGCTGACAGGTCAGGGTCAACATGCCCTTAATCAAACCAACTACATCTGGGGCGTCTATGGTCAGGTGTCGGCCGCCGCTATTAAAGCATGGAAGGCCCTCACAAAAAGGGGAGAGTCCAGTGGACACCTTACAAAGATTGTCCAAGGACCCCAAGAGCCATTTTCAGACTTTGTGGCCAGAATGACAGAGGC
CGCCAGTCGCATCTTTGGTGATTCAGAACAAGCTATGCCCCTGATTGAGCAACTTGTCTATGAACAAGCAACCCAGGAATGTCGAGCGGCTATCGCCCCTCGCAAAAGCAAAGGTTTACAAGATTGGCTCAGAGTGTGCAGGGAGCTGGGGGGACCCCTAACCAATGCAGGACTAGCTGCCGCCATTTTACAAACGCACAGGTATAGAGATTTATCTAATCGAAAA
GCTTGTTTTAATTGTGGGAGAATGGGGCACCTTAAGAAAGATTGTCAAGCCCCTGAAAGAACAAGAGAATCGAAGCTTTGCTATCGTTGTGGTAAAGGTTATCATAGGGCTAGCGAATGCAGGTCAGTCCGGGATGTAAAAGGGAGGCTTCTGCCCCCTATTGAAGAATCCAAGGTACCCCAATCAAAAAACGGGTCACGGGGCCCATGGTCCCAGGGCCCTCAGA
AATATGGGAACCAATTTCAGAAAAGCAATCAAGAGGAAGTAGAGGTTTCCGAGGAACCTCCGGAATGGACTTGCGTGCCGCCTCCGACTTCTTACTCATGCCCCAAATGAATGTTCAACCGGTCCCAGTCCATTCCCCTGGACCTTTGCCCCCTGCCACCATTGGGCTTATTCTGGGCCGTGGCTCCTTAACTTTACAGGGACTCATTATATATCCTGGAATTGTT
GACCCGTATCATAAAGAGGAGATTCAAGTTCTCTGTTCTAGCCCTCGGGATATATTTTCTATAAAGCAGGGAGATAGAATTGCACAACTGGTGTTGCTGCCCTCCCTTGGGGATGGAGAAACCTACACTTTACAAAAAAGGGCTATGGGATCTTCTGGAAATGATTCAGCCTATCTGGCTATACCCCTAAATGAGAGACCTACAATAAAATTATTAATTAATGGAA
AAGAATTTGAAGGGATAATGGATTCGGGAGCAGACAAAAGCATTATTTCACTCCATTGGTGGCCGAAGTCTTGGCCCACTGTTGTTTCATCTCATTCTCTACAAGGTCTTGGATACCAGTCCTCTCCTGCCATCAGTGCCTCAGCCTTAACCTGGCGGGATGCTGAAGGCAAACAGGGATGTTTTACCCCCTATGTGTTGCCACTCCCTGTAAATTTATGGGGACG
AGATGTGTTACAAGCCATGGGCATGACCCTAACTAATGAATACTCCCCCAGGCATCAGCCATTATGGCAAAGATGGGGTATACAAATGGTAGAGGCTTGGGTAGGCAAGAACAAGGCAGAATAAAACCCATTACACAACACGGAAATCGGGGTAGAAAAGGACTGGGTTTTATTTAGGGGCCGTTGAGGCTTCACGACCCATACCATGGAAAACAGAGGAGCCGT
TATGGGTCTCTCAATGGCCTCTATCCTCTGAAAAATTAGAGGCTGTCACAAGATTAGTGCAAGAACAGGAACGGCTGGGGCATCTAGAGCCTTCTACCTCCCCATGGAATACACCAATTTTTGTTATTAAGAAGAAATCTGGGAAGTGGAGATTACTCCACGACCTGCGGGCCATTAACAATCAGATGCATCTTTTGGCCCTGTTCAAAGAGGCCTTCCTTTGCT
TTCTGCACTTCCTCAAGATTGGAAGCTTATTATTATAGATATTAAGGATTGTTTCTTCTCTATTCCACTTTACCCACGGGATAGACCAAGGTTTGCCTTCACTATCCCTTCTCTTAATCATATGGAACCAGACAAGAGATTTCAGTGGAAGGTACTGCCGCAAGGCATGGCCAATAGCCCAACAATATGTCAGCTATATGTGCAGGAGGCTTTGGAGCCAATTAGG
AAGCAATTTACATCTTTAATCGTTATCCATTATATGGACGATATTCTTATTTGTCACAAAGAGTTAGATGTTTTACAAAGGCTTTCCCCATGCTGGTGGCTGAATTAAAACAGTGGGGATTAGAGATTGCCTCAGAAAAGGTTCAAATTGCAGATACAGGCCTCTTTTTGGGCTCAAAGATTACTCCCAAAAATATAGTTCCTCAGAAAATAGAAATTCGCAAAG
ACCACTTACAAACATTAAATGATTTTCAGAAACTTCTGGGAGATATAAATTGGTTGAGGCCCTTTTTAAAAATTCCATCTGCTGATCTAAAACCCCTCTTCGATCTCCTGGAGGGTGAGCCTCATATCTCCTCACCCAGAAAATTTACCCCTGCCGCCCACCGAGCCTTACAGATGGTGGAAGAGGCCTTACAGGAAGCACAATTACAACGAATTGAGGAGTCACT
ACCTTTTAATCTGTGTGTTTTTAAGACAGCTCAATTGCCAACTGCAGTCCTGTGGCAACATGGACCATTACTTTGGATTCATCCAAATGCTTCCCCTGCTAAGATTATTGACTGGTACCCTGATGCTGTTGCACAGCTCGCGCTTCGCGGAATAAAAGCAGCTGTTACACATTTTGGCAGGGATCCTGATTCCTTGATTGTGCCTTATACTGCTGCACAAGTTCAG
ACCTTGGCAGCCACATCTAGTGATTGGGCAGTTTTAGTTACTTCCTTTTCAGGACAAATTGATAATCATTTTCAAAGCATCCAATTTTACAGTTTGCCCTAAACCAGGCTATAGTGTTTCCACAAGTGACAGCTAAAGACCCACTCCCAGATGGGACTGTGGTGTACACTGATGGATCAAAAACTGGTATGGGAGCTTATGTGGTCAAGGATAGGGTTATATCTA
AACAATATAATGAAACTTCACCTCAAGTTGTAGAATGTCTGATAGTACTGGAGGTTCTTGAGGCTTTTCCAGGACCCCTCAATATTGTATCAGATTCCTCTTATGTGGTTAATGCAGTTAATCTCCTTGAGATAGCTGGAATAATAAGATCCTCCAGCAGAGTTGCTAACATCTTTCAGAAGATACAAGCTGCCTTGTTAAATAGGAGATTTCCTGTTTTCATTAC
CCATGTCCGAGCACATTCTGGACTCCCAGGTCCCATGTCCTTAGGGAATGACTTAGCGGACAAGGCTACAAAGCTGGTGGCCGCTGCCTTGTCTACCCATGCACAAGCAGCAAAAGAATTCCATAAGCGCTTTCATGTGACGGCTGAGACCTTGCGCCGTCGTTTTGCTCTATCCAGGAAAGAGGCTAGGGAAATTGTTACTCAATGCCAGAATTGCTGCGAATTC
TTACCTACTCCCCACATGGGAATAAACCCACGTGGCATTAGACCATTGCAGATGTGGCAGATGGATGTTACTCATATTCCCTCTTTTGGAAGGCTTCAATATGTTCATGTTTCTGTTGACACCTGTTCTGGCGTAATGTTTGCCACACCCCTAACAGGGGAAAAGGCCTCATATGTGATTCAACATTGCCTTGAGGCCTGGAGTGCTTGGGGAAAACCCAGAATTT
TAAAAACAGACAATGGACCAGCATATACCTCTCAAAAATTCCGACAGTTTTGTCGGCAAATGGATGTTACCCATCTAACTGGCCTACCTTATAACCCTCAAGGACAGGGCATTGTGGAACGTGCCCATCGTACACTCAAATCATATCTGATTAAACAAAAGGGGAGTATTGAGGATGTTCTACCCTCGGTACCAAGAGTTGCTGTATCCATGGCACTTTTTACCCT
TAATTTCTTGAACTGTGATGCCCAAGGCCACACTGCGGCTGATCGCCACAGTTTAGAGCCTGATAGGCCAAAAGAAATGGTAAAACGGAAAGATGTCTTGACTGATCTCTGGAAAGGCCCGGATCCTATTCTTATAAGATCCAGGGGAGCTGTGTGTGTTTTTCCACAGGAGGAAGAAAACCCCCTGTGGATCCCTGAAAGACTCACTCGAAGAGCTCCTTCACAA
CTCCAGAATAAGAAGGATGGAGGCCAAGTGGGTGATGAAGACTCTCCTACTCGGGATGATGGTTCCTAGTATGATGGGAGAGTTACGGTGGGCCATCATGTCTACCTTCCCCCTACCGATGCCAGTGATGCAGAATGCCCAGGTGTTCCCTAGATTTTTCACAAATAATAGAGAGTTGGGGCTTGCCTTTTTGCCTTTAGATCCTCAGATACAGAGTTTTCAGGAT
GAGAGAACCTTGTCATTAGTAGGAAGCTTGTGCTTTACTGTGATTTCTTCATTAAAGGATAATTTTATTCATATGGATCCAGAAAGATCTAATGCTAATCCTTTTGAATTAATGGAAGCTGTACTGATCTTTCTCCTATGATAGTGAGAAGGGACAGATTACTTTTGATTGGAAAGGGGCATTAGGCAACAGTCTGAGTGAGGGACAGAGCTCTGGATCATCTGAT
TGGTCCTCTCCAATGTTAAGTATACCCCTTTTAAGAGAACTAATTATTCAGCTGCCCTTTATTTGGGTTGTGAGTAATGATACCTTAGGTAAGAATCAGAAAGAAATAAATTGTTCTGCTTAGAATTGTTTTTATGCATTATGCTGGGATGCTATTAAGCACCCTTTTGCTTTGGTAATGCGCATGCCTCGATTTATTCCAGTTCCTTGAGACGCCAAGTAATTT
GAGTTTGTTTAGGGAAAAGCGAGATTTTTGGGATCTCTGCTATTATTGTGGGCCTGATAGCCACCGCCGCGGTTACTGCTTCGGTTACCGCCTCGGCCCTTGCACTTTCTGCCACTGTGCAGACAACTCAAACCATTAATGAACTATAGGCCACTGTGACCATGGTCCTAGATAAACAGGCCACTGCCAATTCTCAGATACAAGGAGGCCTGATGTTGGTAAACCA
ACAGATTAATTTGGTTCAGGAACAATTGGATATATTGTGGCAGATGGCCAAATTGGGATGTGAACAAAGTTGCCTGGCCTTTGTGTCACTTAGTACAATATGAGAATTTTACAAGAGCAGCTAACCTGTCAAAAGCTCTGTCTCAACATTTATTACAGAATTGGACTTTTGAGTTTGAGCAGACACTCCGTGAGTTGAGAATGGCGATCCTGCAAGTGAATTCGAC
GCATCTGGATCTTTCTCTGATGGAAGACCTCTCCCCCTGGATTTCATCTGCATTTTCTTATTTTAAGGAATGGGTGGGGGTAGGATCATTTAGCCTAGTTCTCTGCTGTGGAATAGTGTTGATGCTTTGGATGGTGTGTACGCTCAGAGCCCAAACAAAAGGAGAAAGTGGTGGTGGCACAAGCACTTGCTGCCTTAGAACACGGAGCATCCCCTGACATTTGGTT
AACCATGTTAAAGCAATAGGTCGCTGGCCTCCCAGCTCTTGCACCCCACGAGGCTAGTCTCATTGCACGGGATAGAGTGGGTAGGCTTCAGCAGCCCAAGGAGTTGCAAGGCTAAGCACTGCACAGGAGAGGTCTGCGGTATAACGACTTTCTCCTGGGAGATAAGTCATCTTGCATGAAGGTTCTATGTCATGATCTCCTTCCCCCAGAAAAAAGACATCGGACT
GGTCAGGACTTCCTCTGGGGATAAGACCCTGGAGAGGAGCTTTGTACATGTTCCGAATTTGCACATGGGGATTTGACCTCTATCTCCACTCCAAAGTTGTGGGTGTCCTATTGCTTCAAAAATAAATGAATAGGGGGAGATGTGAGGAGCCGCCCTCGCTATCGCTATTGCCGTTAGAAGATGGCGCTGACATCCACTGTCAGTTGGAGTTAACTGTCGCTGTCAG
TTGGAGTTAACCGTTTAAACTGTGCCTCTCCCGTGACGTCATCTGGGGTGATGTGCAAACCACCAATCCCGGCTCTACACATCTCACTCGGAGCTCCTAGGCTTATATAAGGGGCTGGGTTTCTTAGCTTGGGGTCTCCCTCTAAGAAGCTGATCATCTATCTCTCAAGATGCATTAAAGCTTTACTGCAGAAGGATCCGAGTGTTCTGCGTCGTTCTTGCTGGCG
AGACGGTAGCGCGGGACA

>IAPLTR3.2571193

[illegible]

>IAPLTR3.546568

[illegible]

>IAPLTR3.4039526

[illegible]

>IAPLTR3.3559589

[illegible]

>IAPLTR3.1754286

[illegible]

CAGAGGCCGCCAGTCGCATCTTTGGTGATTCAGAACAAGCTATGCCCCTGATTGAGCAACTTGTCTATGAACAAGCAACCCAGGAATGTCGAGCGGCTATCGCCCCTCGCAAAAGCAAAGGTTTACAAGATTGGCTCAGAGTGTGCAGGGAGCTGGGGGGACCCCTAACCAATGCAGGACTAGCTGCCGCCATTTTACAAACGCACAGGTATAGAGATTTATCTAA
TCGAAAAGCTTGTTTTAATTGTGGGAGAATGGGGCACCTTAAGAAAGATTGTCAAGCCCCTGAAAGAACAAGAGAATCGAAGCTTTGCTATCGTTGTGGTAAAGGTTATCATAGGGCTAGCGAATGCAGGTCAGTCCGGGATGTAAAAGGGAGGCTTCTGCCCCCTATTGAAGAATCCAAGGTACCCCAATCAAAAAACGGGTCACGGGGCCCATGGTCCCAGGGC
CCTCAGAAATATGGGAACCAATTTCAGAAAAGCAATCAAGAGGAAGTAGAGGTTTCCGAGGAACCTCCGGAATGGACTTGCGTGCCGCCTCCGACTTCTTACTCATGCCCCAAATGAATGTTCAACCGGTCCCAGTCCATTCCCCTGGACCTTTGCCCCCTGCCACCATTGGGCTTATTCTGGGCCGTGGCTCCTTAACTTTACAGGGACTCATTATATATCCTGG
GATTGTTGACCCATATCATAAAGAGGAGATTCAAGTTCTCTGTTCTAGCCCTCGGGGTGTATTTTCTATAAAGCAGGGAGATAGAATTGCACAACTGGTGTTGCTGCCCTCCCTCGGGGATGGAGAAACCTACACTTTACAAAAAAGGGCTATGGGATCTTCTGGAAGTGATTCAGCCTATCTGGCTATACCCCTAAATGAGAGACCTACAATAAAATTGTTAATT
AATGGAAAAGAATTTGAAGGGATAATGGATTCGGGAGCAGACAAAAGCATTATTTCACTCCATTGGTGGCCGAAGTCTTGGCCCACTGTTGTTTCATCTCATTCTCTACAAGGTCTTGGATACCAGTCCTCTCCTGCCATCAGTGCCTCAGCCTTAACCTGGCGGGATGCTGAAGGCAAACAGGGATGTTTTACCCCCTATGTGTTGCCACTCCCTGTAAATTTAT
GGGGACGAGATGTGTTACAAGCCATGGGCATGACCCTAACTAATGAATACTCCCCCAGGCATCAGCCATTATGGCAAAGATGGGGTATACAAATGGTAGAGGCTTGGGTAGGCAAGAACAAGGCAGAATAAAACCCATTACACAACACGGAAATCGGGGTAGAAAAGGACTGGGTTTTATTTAGGGGCCGTTGAGGCTTCACGACCCATACCATGGAAAACAGAG
GAGCCGTTATGGGTCTCTCAATGGCCTCTATCCTCTGAAAAATTAGAGGCTGTCACAAGATTAGTGCAAGAACAGGAACGGCTGGGGCATCTAGAGCCTTCTACCTCCCCATGGAATACACCAATTTTTGTTATTAAGAAGAAATCTGGGAAGTGGAGATTACTCCACGACCTGCGGGCCATTAACAATCAGATGCATCTTTTGGCCCTGTTCAAAGAGGCCTTC
CTTTGCTTTCTGCACTTCCTCAAGATTGGAAGCTTATTATTATAGATATTAAGGATTGTTTCTTCTCTATTCCACTTTACCCACGGGATAGACCAAGGTTTGCCTTCACTATCCCTTCTCTTAATCATATGGAACCAGACAAGAGATTTCAGTGGAAGGTACTGCCGCAAGGCATGGCCAATAGCCCAACAATATGTCAGCTATATGTGCAGGAGGCGTTGGAGCC
AATTAGGAAGCAATTTACATCTTTAATCGTTATCCATTATATGGACGATATTCTTATTTGTCACAAAGAGTTAGATGTTTTACAAAAGGCTTTTCCCATGCTGGTGGCTGAATTAAAACAGTGGGGATTAGAGATTGCCTCAGAAAAGGTTCAAATTGCAGATACAGGGCTCTTTTTGGGCTCAAAGATTACTCCCAAAAATATAGTTCCTCAGAAAATAGAAATT
CGCAAAGACCACTTACAAACATTAAATGATTTTCAGAAACTTCTGGGAGATATAAATTGGTTGAGGCCCTTTTTAAAAATTCCATCTGCTGATCTAAAACCCCTCTTCGATCTCCTGGAGGGTGAGCCTCATATCTCCTCACCCAGAAAATTTACCCCTGCCGCCCACCGAGCCTTACAGATGGTGGAAGAGGCCTTACAGGAAGCACAATTACAACGAATTGAGG
AGTCACTACCTTTTAATCTGTGTGTTTTTAAGACAGCTCAATTGCCAACTGCAGTCCTGTGGCAACATGGACCATTACTTTGGATTCATCCAAATGCTTCCCCTGCTAAGATTATTGACTGGTACCCTGATGCTGTTGCACAGCTCGCGCTTCGCGGAATAAAAGCAGCTGTTACACATTTTGGCAGGGATCCTGATTCCTTGATTGTGCCTTATACTGCTGCACA
AGTTCAGACCTTGGCAGCCACATCTAGTGATTGGGCAGTTTTAGTTACTTCCTTTTCAGGACAAATTGATAATCATTTTCCAAAGCATCCAATTTTACAGTTTGCCCTAAACCAGGCTATAGTGTTTCCACAAGTGACAGCTAAAGACCCACTCCCAGATGGGACTGTGGTGTACACTGATGGATCAAAAACTGGTATGGGAGCTTATGTGGTCAAGGATAGGGTT
ATATCTAAACAATATAATGAAACTTCACCTCAAGTTGTAGAATGTCTGATAGTACTGGAGGTTCTTGAGGCTTTTCCAGGACCCCTCAATATTGTATCAGATTCCTCTTATGTGGTTAATGCAGTTAATCTCCTTGAGATAGCTGGAATAATAAGATCCTCCAGCAGAGTTGCTAACATCTTTCAGAAGATACAAGCTGCCTTGTTAAATAGGAGATTTCCTGTTT
TCATTACCCATGTCCGAGCACATTCTGGACTCCCAGGTCCCATGTCCTTAGGGAATGACTTAGCGGACAAGGCTACAAAGCTGGTGGCCGCTGCCTTGTCTACCCATGCACAAGCAGCAAAAGAATTCCATAAGCGCTTTCATGTGACGGCTGAGACCTTGCGCCGTCGTTTTGCTCTATCCAGGAAAGAGGCTAGGGAAATTGTTACTCAATGCCAGAATTGCTG
CGAATTCTTACCTACTCCCCACATGGGAATAAACCCACGTGGCATTAGACCATTGCAGATGTGGCAGATGGATGTTACTCATATTCCCTCTTTTGGAAGGCTTCAATATGTTCATGTTTCTGTTGACACCTGTTCTGGCGTAATGTTTGCCACACCCCTAACAGGGGAAAAGGCCTCATATGTGATTCAACATTGCCTTGAGGCCTGGAGTGCTTGGGGAAAACCC
AGAATTTTAAAAACAGACAATGGACCAGCATATACCTCTCAAAAATTCCGACAGTTTTGTCGGCAAATGGATGTTACCCATCTAACTGGCCTACCTTATAACCCTCAAGGACAGGGCATTGTGGAACGTGCCCATCGTACACTCAAATCATATCTGATTAAACAAAAGGGGGAGTATTGAGGATGTTCTACCCTCGGTACCAAGAGTTGCTGTATCCATGGCACTTT
TTACCCTTAATTTCTTGAACTGTGATGCCCAAGGCCACACTGCGGCTGATCGCCACAGTTTAGAGCCTGATAGGCCAAAAGAAATGGTAAAATGGAAAGATGTCTTGACTGATCTCTGGAAAGGCCCGGATCCTATTCTTATAAGATCCAGGGGAGCTGTGTGTGTTTTTCCACAGGAGGAAGAAAACCCCCTGTGGATCCCTGAAAGACTCACTCGAAGAGCTCC
TTCACAACTCCAGAATAAGAAGGATGGAGGCCAAGTGGGTGATGAAGACTCTCCTACTCGGGATGATGGTTCCTAGTATGATGGGAGAGTTACGGTGGGCCATCATGTCTACCTTCCCCCTACCGATGCCAGTGATGCAGAATGCCCAGGTGTTCCCTAGATTTTTCACAAATAATAGAGAGTTGGGGCTTGCCTTTTTGCCTTTAGATCCTCAGATACAGAGTTT
TCAGGATGAGAGAACCTTGTCATTAGTAGGAAGCTTGTGCTTTACTGTGATTTCTTCATTAAAGGATAATTTTATTCATATGGATCCAGAAAGATCTAATGCTAATCCTTTTGAATTAATGGAAGCTGTACTGATCTTTCTCCTATGATAGTGAGAAGGGACAGATTACTTTTGATTGGAAAGGGGCATTAGGCAACAGTCTGAGTGAGGGACAGAGCTCTGGATC
ATCTGATTGGTCCTCCTCCAATGTTAAGTATACCCCTTTTAAGAGAACTAATTATTCAGCTGCCCTTTATTTGGGTTGTGAGTAATGATACCTTAGGTAAGAATCAGAAAGAAATAAATTGCTCTGCTTAGAATTGTTTTTATGCATTATGCTGGGATGCTATTAAGCACCCTTTTGCTTTGGTAATGCGCATGCCTCGATTTATTCCAGCTCCTGTTGAGGCGCC
AAGTAATTTGAGTTTGTTTAGGGAAAAGCGAGATTTTTGGGATCTCTGCTATTATTGTGGGCCTGATAGCCACCGCCGCGGTTACTGCTTCGGTTACCGCCTCGGCCCTTGCACTTTCTGCCACTGTGCAGACAACTCAAACCATTAATGAACTATAGGCCACTGTGACCATGGTCCTAGATAAACAGGCCACTGCCAATTCTCAGATACAAGGAGGCCTGATGTT
GGTAAACCAACAGATTAATTGGTTCAGGAACAATTGGATATATTGTGGCAGATGGCCAAATTGGGATGTGAACAAAGTTGCCTGGCCTTTGTGTCACTTAGTACAATATGAGAATTTTACAAGAGCAGCTAACCTGTCAAAAGCTCTGTCTCAACATTTATTACAGAATTGGACTTTTGAGTTTGAGCAGACACTCCGTGAGTTGAGAATGGCGATCCTGCAAGT
GAATTCGACGCATCTGGATCTTTCTCTGATGGAAGACCTCTCCCCCTGGATTTCATCTGCATTTTCTTATTTTAAGGAATGGGTGGGGGTAGGATCATTTAGCCTAGTTCTCTGCTGTGGAATAGTGTTGATGCTTTGGATGGTGTGTACGCTCAGAGCCCAAACAAAAGGAGAAAGTGGTGGTGGCACAAGCACTTGCTGCCTTAGAACACGGAGCATCCCCTGA
CATTTGGTTAACCATGTTAAAGCAATAGGTCGCTGGCCTCCCAGCTCTTGCACCCCACGAGGCTAGTCTCATTGCACGGGATAGAGTGGGTAGGCTTCAGCAGCCCAAGGAGTTGCAAGGCTAAGCACTGCACAGGAGAGGTCTGCGGTATAACGACTTTCTCCTGGGAGATAAGTCATCTTGCATGAAGGTTCTATGTCATGATCTCCTTCCCCCAGAAAAAAGA
CATCGGACTGGTCAGGACTTCCTCTGGGGATAAGACCCTGGAGAGGAGCTTTGTACATGTTCCGAATTTGCACATGGGGATTTGACCTCTATCTCCACTCCAAAGTTGTGGGTGTCCTATTGCTTCAAAAATAAATGAATAGGGGGAGATGTGAGGAGCCGCCCTCGCTATTGCCGTTAGAAGATGGCGCTGACATCCACTGTCAGTTGGAGTTAACTGTCGCTGT
CAGTTGGAGTTAACTGTCGCTGTCAGTTGGAGTTAACTGTCGCTGTCAGTTGGAGTTAACTGTCGCTGTCAGTTGGAGTTAACCGTTTAAACTGTGCCTCTCCCGTGACGTCATCTGGGGTGATGTGCAAACCACCAATCCCGGCTCTACACATCTCACTCGGAGCTCCTAGGCTTATATAAGGGGCTGGGTTTCTTAGCTTGGGGTCTCCCTCTAAGAAGCTGAT
CATCTATCTCTCAAGATGCATTAAAGCTTTACTGCAGAAGGATCCGAGTGTTCTGCGTCGTTCTTGCTGGCGAGACGGTAGCGCGGGACA

>IAPLTR3.199311
TGTGAGGAGCCGCCCTCGCTATCGCTATTGCCGTTAGAAGATGGCGCTGACATCCACTGTCAGTTGGAGTTAACTGTCGCTGTCAGTTGGAGTTAACTGTCGCTGTCAGTTGGAGTTAACTGTCGCTGTCAGTTGGAGTTAACCGTTTAAACTGTGCCTCTCCCGTGACGTCATCTGGGGTGATGTGCAAACCACCAATCCCGGCTCTACACATCTCACTCGGAGC
TCCTAGGCTTATATAAGGGGCTGGGTTTCTTAGCTTGGGGTCTCCCTCTAAGAAGCTGATCATCTATCTCTCAAGATGCATTAAAGCTTTACTGCAGAAGGATCCGAGTGTTCTGCGTCGTTCTTGCTGGCGAGACGGTAGCGCGGGACATCTGGTGCCGAAACCCGGGAACTCTTCAACATCGCCGGCGCCGCGGGAGACCCCTCGGAAAACGGGGCGGATTCAG
AACTGCAGGGACGTAGGTTCAGAGAGGTATGCTTTATCCTGAACAACCCTTTTCTCGACTTTGGCCTTAGTTTGTCATCACCGTGGGACGGGTCAGTGGAGGCTTTGGTGCTAGTCCTGTTTGTCCTTCTCCTTCATCGTGGCTGGTGCTGACAGAACGGCGCTGTCCATCTGGCGCTGTCCATCTGGCGCTGTCCATCTGGCGCTGTCCATCTGGCGCTGTCAGT
CTGGCGCTGTCAGTCTGGTGCTGTCAGTCTGGTGCTGTCAGTCTGGTGCTGTCAGTCTGGTAACGGAGTTGAGCGCGTTTTTTAGATAAGCTGTCAGCGCGATGGGGTCGTCACAGTCAGTGGTTACAGCCTTACAGACTGTGCTAAAACAAAGAGATCTTAAAATTGCACCACGAACACTTCAAAATTTTGTGAAGGAGGTAGATAGAGTGGCACCATGGTATGC
TTGTTCCGGGTCCCTCACTGTGGCCTCATGGAACAAGCTAGGTAAAGAACTAGACAGGAAGCATCAGAGGAGATCTCTGCTTAGGTACCAAAGCCATTTGGAAGCTAGTTAAAAACTGCTTGGAGGATGAGGCCTGTCACCCTGCTATAATAGAAAGTCAGGGAACCTTAGAGGAAGTTCAGGATAGCATGTCAGAAACTGAACGGAGTGAGAGAATGGGAGCT
CGCAAAGAAAAGACATGTCTAAGAAAAAGGCCTCCCCAGGAAGTTAAGAAAGGGGGAGAGAAAGAAGGGAGTTATCATTCACCCCTTAACAAATCTAAGAAAAAGAAAAAGCCAGAATCTAGTCTATACCCTGCAGTAGAGCTAGAAGCCTTGGAGCTAGATAATTTTGATTCTGACACTCTGGATTCTAGCGAAGAAGGTGGTCTAGAGGAGGAGGTGGCAC
GGTATGAGGAAAAAAGATATCACCCTGATAGACACCGGCCACTGAAAACAAAAATGAATGTGAGGCCACCGCCTATAAATCCGGCGGGCTCACGCCCCTCAGCGCCTCCGCCATATGAGCTTCGGCTGAATACTGGTACTGACTCCTTTTTACCTTTAGAGGAACGGAGAAAAATACAAATGGCTTTTCCAGTATTCGAGAATGTGGAAGGCGGAAGAGTACATAC
CCCCGTAGATTACAATCAAATTAAAGAATTGGCCGAATCCGTTCGTAATTATGGGGTCAATGCTAACTTTACCACATTACAGGTGGAAAGGCTTGCTAATTTTGCCATGACTCCCACTGACTGGCAAACGACAGTGAAAGCAGTGCTTCCCAACATGGGACAATACATGGAATGGAAGGCTCTCTGGTATGATGCAGCCCAGGCACAAGCTAGAGTTAATGCCACG
GCTGAAGATGAGAATCAGAGGCAGTGGACATTTGAAATGCTGACAGGTCAGGGTCAACATGCCCTTAATCAAACCAACTACATCTGGGGCGTCTATGGTCAGGTGTCGGCCGCCGCTATTAAAGCATGGAAGGCCCTCACAAAAAGGGGAGAGTCCAGTGGACACCTTACAAAGATTGTCCAAGGACCCCAAGAGCCATTTTCAGACTTTGTGGCCAGAATGACAG
AGGCCGCCAGTCGCATCTTTGGTGATTCAGAACAAGCTATGCCCCTGATTGAGCAACTTGTCTATGAACAAGCAACCCAGGAATGTCGAGCGGCTATCGCCCCTCGCAAAAGCAAAGGTTTACAAGATTGGCTCAGAGTGTGCAGGGAGCTGGGGGGACCCCTAACCAATGCAGGACTAGCTGCCGCCATTTTACAAACGCACAGGTATAGAGATTTATCTAATCG
AAAAGCTTGTTTTAATTGTGGGAGAATGGGGCACCTTAAGAAAGATTGTCAAGCCCCTGAAAGAACAAGAGAATCGAAGCTTTGCTATCGTTGTGGTAAAGGTTATCATAGGGCTAGCGAATGCAGGTCAGTCCGGGATGTAAAAGGGAGGCTTCTGCCCCCTATTGAAGAATCCAAGGTACCCCAATCAAAAAACGGGTCACGGGGCCCATGGTCCCAGGGCCCT
CAGAAATATGGGAACCAATTTCAGAAAAGCAATCAAGAGGAAGTAGAGGTTTCCGAGGAACCTCCGGAATGGACTTGCGTGCCGCCTCCGACTTCTTACTCATGCCCCAAATGAATGTTCAACCGGTCCCAGTCCATTCCCCTGGACCTTTGCCCCCTGCCACCATTGGGCTTATTCTGGGCCGTGGCTCCTTAACTTTACAGGGACTCATTATATATCCTGGAAT
TGTTGACCCATATCATAAAGAGGAGATTCAAGTTCTCTGTTCTAGCCCTCGGGGTGTATTTTCTATAAAGCAGGGAGATAGAATTGCACAACTGGTGTTGCTGCCCTCCCTCGGGGATGGAGAAACCTACACTTTACAAAAAAGGGCTATGGGATCTTCTGGAAGTGATTCAGCCTATCTGGCTATACCCCTAAATGAGAGACCTACAATAAAATTGTTAATTAAT
GGAAAAGAATTTGAAGGGATAATGGATTCGGGAGCAGACAAAAGCATTATTTCACTCCATTGGTGGCCGAAGTCTTGGCCCACTGTTGTTTCATCTCATTCTCTACAAGGTCTTGGATACCAGTCCTCTCCTGCCATCAGTGCCTCAGCCTTAACCTGGCGGGATGCTGAAGGCAAACAGGGATGTTTTACCCCCTATGTGTTGCCACTCCCTGTAAATTTATGGG
GACGAGATGTGTTACAAGCCATGGGCATGACCCTAACTAATGAATACTCCCCCAGGCATCAGCCATTATGGCAAAGATGGGGTATACAAATGGTAGAGGCTTGGGTAGGCAAGAACAAGGCAGAATAAAACCCATTACACAACACGGAAATCGGGGTAGAAAAGGACTGGGTTTTATTTAGGGGCCGTTGAGGCTTCACGACCCATACCATGGAAAACAGAGGAG
CCGTTATGGGTCTCTCAATGGCCTCTATCCTCTGAAAAATTAGAGGCTGTCACAAGATTAGTGCAAGAACAGGAACGGCTGGGGCATCTAGAGCCTTCTACCTCCCCATGGAATACACCAATTTTTGTTATTAAGAAGAAATCTGGGAAGTGGAGATTACTCCACGACCTGCGGGCCATTAACAATCAGATGCATCTTTTGGCCCTGTTCAAAGAGGCCTTCCTT
TGCTTTCTGCACTTCCTCAAGATTGGAAGCTTATTATTATAGATATTAAGGATTGTTTCTTCTCTATTCCACTTTACCCACGGGATAGACCAAGGTTTGCCTTCACTATCCCTTCTCTTAATCATATGGAACCAGACAAGAGATTTCAGTGGAAGGTACTGCCGCAAGGCATGGCCAATAGCCCAACAATATGTCAGCTATATGTGCAGGAGGCTTTGGAGCCAAT
TAGGAAGCAATTTACATCTTTAATCGTTATCCATTATATGGACGATATTCTTATTTGTCACAAAGAGTTAGATGTTTTACAAAAGGCTTTTCCCATGCTGGTGGCTGAATTAAAACAGTGGGGATTAGAGATTGCCTCAGAAAAGGTTCAAATTGCAGATACAGGCCTCTTTTTGGGCTCAATGATTACTCCCAAAAATATAGTTCCTCAGAAAATAGAAATTCGC
AAAGACCACTTACAAACATTAAATGATTTTCAGAAACTTCTGGGAGATATAAATTGGTTGAGGCCCTTTTTAAAAATTCCATCTGCTGATCTAAAACCCCTCTTCGATCTCCTGGAGGGTGAGCCTCATATCTCCTCACCCAGAAAATTTACCCCTGCCGCCCACCGAGCCTTACAGATGGTGGAAGAGGCCTTACAGGAAGCACAATTACAACGAATTGAGGAGT
CACTACCTTTTAATCTGTGTGTTTTTAAGACAGCTCAATTGCCAACTGCAGTCCTGTGGCAACATGGACCATTACTTTGGATTCATCCAAATGCTTCCCCTGCTAAGATTATTGACTGGTACCCTGATGCTGTTGCACAGCTCGCGCTTCGCGGAATAAAAGCAGCTGTTACACATTTTGGCAGGGATCCTGATTCCTTGATTGTGCCTTATACTGCTGCACAAGT
TCAGACCTTGGCAGCCACATCTAGTGATTGGGCAGTTTTAGTTACTTCCTTTTCAGGACAAATTGATAATCATTTTCCAAAGCATCCAATTTTACAGTTTGCCCTAAACCAGGCTATAGTGTTTCCACAAGTGACAGCTAAAGACCCACTCCCAGATGGGACTGTGGTGTACACTGATGGATCAAAAACTGGTATGGGAGCTTATGTGGTCAAGGATAGGGTTATA
TCTAAACAATATAATGAAACTTCACCTCAAGTTGTAGAATGTCTGATAGTACTGGAGGTTCTTGAGGCTTTTCCAGGACCCCTCAATATTGTATCAGATTCCTCTTATGTGGTTAATGCAGTTAATCTCCTTGAGATAGCTGGAATAATAAGATCCTCCAGCAGAGTTGCTAACATCTTTCAGAAGATACAAGCTGCCTTGTTAAATAGGAGATTTCCTGTTTTCA
TTACCCATGTCCGAGCACATTCTGGACTCCCAGGTCCCATGTCCTTAGGGAATGACTTAGCGGACAAGGCTACAAAGCTGGTGGCCGCTGCCTTGTCTACCCATGCACAAGCAGCAAAAGAATTCCATAAGCGCTTTCATGTGACGGCTGAGACCTTGCGCCGTCGTTTTGCTCTATCCAGGAAAGAGGCTAGGGAAATTGTTACTCAATGCCAGAATTGCTGCGA
ATTCTTACCTACTCCCCACATGGGAATAAACCCACGTGGCATTAGACCATTGCAGATGTGGCAGATGGATGTTACTCATATTCCCTCTTTTGGAAGGCTTCAATATGTTCATGTTTCTGTTGACACCTGTTCTGGCGTAATGTTTGCCACACCCCTAACAGGGGAAAAGGCCTCATATGTGATTCAACATTGCCTTGAGGCCTGGAGTGCTTGGGGAAAACCCAGA
ATTTTAAAAACAGACAATGGACCAGCATATACCTCTCAAAAATTCCGACAGTTTTGTCGGCAAATGGATGTTACCCATCTAACTGGCCTACCTTATAACCCTCAAGGACAGGGCATTGTGGAACGTGCCCATCGTACACTCAAATCATATCTGATTAAACAAAAGGGGGAGTATTGAGGATGTTCTACCCTCGGTACCAAGAGTTGCTGTATCCATGGCACTTTTTA
CCCTTAATTTCTTGAACTGTGATGCCCAAGGCCACACTGCGGCTGATCGCCACAGTTTAGAGCCTGATAGGCCAAAAGAAATGGTAAAATGGAAAGATGTCTTGACTGATCTCTGGAAAGGCCCGGATCCTATTCTTATAAGATCCAGGGGAGCTGTGTGTGTTTTTCCACAGGAGGAAGAAAACCCCCTGTGGATCCCTGAAAGACTCACTCGAAGAGCTCCTTC
ACAACTCCAGAATAAGAAGGATGGAGGCCAAGTGGGTGATGAAGACTCTCCTACTCGGGATGATGGTTCCTAGTATGATGGGAGAGTTACGGTGGGCCATCATGTCTACCTTCCCCCTACCGATGCCAGTGATGCAGAATGCCCAGGTGTTCCCTAGATTTTTCACAAATAATAGAGAGTTGGGGCTTGCCTTTTTGCCTTTAGATCCTCAGATACAGAGTTTTCA
GGATGAGAGAACCTTGTCATTAGTAGGAAGCTTGTGCTTTACTGTGATTTCTTCATTAAAGGATAATTTTATTCATATGGATCCAGAAAGATCTAATGCTAATCCTTTTGAATTAATGGAAGCTGTACTGATCTTTCTCCTATGATAGTGAGAAGGGACAGATTACTTTTGATTGGAAAGGGGCATTAGGCAACAGTCTGAGTGAGGGACAGAGCTCTGGATCATC
TGATTGGTCCTCCTCCAATGTTAAGTATACCCCTTTTAAGAGAACTAATTATTCAGCTGCCCTTTATTTGGGTTGTGAGTAATGATACCTTAGGTAAGAATCAGAAAGAAATAAATTGTTCTGCTTAGAATTGTTTTTATGCATTATGCTGGGATGCTATTAAGCACCCTTTTGCTTTGGTAATGCGCATGCCTCGATTTATTCCAGCTCCTGTTGAGGCGCCAAG
TAATTTGAGTTTGTTTAGGGAAAAGCGAGATTTTTGGGATCTCTGCTATTATTGTGGGCCTGATAGCCACCGCCGCGGTTACTGCTTCGGTTACCGCCTCGGCCCTTGCACTTTCTGCCACTGTGCAGACAACTCAAACCATTAATGAACTATAGGCCACTGTGACCATGGTCCTAGATAAACAGGCCACTGCCAATTCTCAGATACAAGGAGGCCTGATGTTGGT
AAACCAACAGATTAATTGGTTCAGGAACAATTGGATATATTGTGGCAGATGGCCAAATTGGGATGTGAACAAAGTTGCCTGGCCTTTGTGTCACTTAGTACAATATGAGAATTTTACAAGAGCAGCTAACCTGTCAAAAGCTCTGTCTCAACATTTATTACAGAATTGGACTTTTGAGTTTGAGCAGACACTCCGTGAGTTGAGAATGGCGATCCTGCAAGTGAA
TTCGACGCATCTGGATCTTTCTCTGATGGAAGACCTCTCCCCCTGGATTTCATCTGCATTTTCTTATTTTAAGGAATGGGTGGGGGTAGGATCATTTAGCCTAGTTCTCTGCTGTGGAATAGTGTTGATGCTTTGGATGGTGTGTACGCTCAGAGCCCAAACAAAAGGAGAAAGTGGTGGTGGCACAAGCACTTGCTGCCTTAGAACACGGAGCATCCCCTGACAT
TTGGTTAACCATGTTAAAGCAATAGGTCGCTGGCCTCCCAGCTCTTGCACCCCACGAGGCTAGTCTCATTGCACGGGATAGAGTGGGTAGGCTTCAGCAGCCCAAGGAGTTGCAAGGCTAAGCACTGCACAGGAGAGGTCTGCGGTATAACGACTTTCTCCTGGGAGATAAGTCATCTTGCATGAAGGTTCTATGTCATGATCTCCTTCCCCCAGAAAAAAGACAT
CGGACTGGTCAGGACTTCCTCTGGGGATAAGACCCTGGAGAGGAGCTTTGTACATGTTCCGAATTTGCACATGGGGATTTGACCTCTATCTCCACTCCAAAGTTGTGGGTGTCCTATTGCTTCAAAAATAAATGAATAGGGGGAGATGTGAGGAGCCGCCCTCGCTATCGCTATTGCCGTTAGAAGATGGCGCTGACATCCACTGTCAGTTGGAGTTAACTGTCGC
TGTCAGTTGGAGTTAACTGTCGCTGTCAGTTGGAGTTAACTGTCGCTGTCAGTTGGAGTTAACCGTTTAAACTGTGCCTCTCCCGTGACGTCATCTGGGGTGATGTGCAAACCACCAATCCCGGCTCTACACATCTCACTCGGAGCTCCTAGGCTTATATAAGGGGCTGGGTTTCTTAGCTTGGGGTCTCCCTCTAAGAAGCTGATCATCTATCTCTCAAGATGCA
TTAAAGCTTTACTGCAGAAGGATCCGAGTGTTCTGCGTCGTTCTTGCTGGCGAGACGGTAGCGCGGGACA

>IAPLTR3.1805841
TGTGAGGAGCCGCCCTCGCTATCGCTATTGCCGTTAGAAGATGGCGCTGACATCCACTGTCAGTTGGAGTTAACTGTCGCTGTCAGTTGGAGTTAACTGTCGCTGTCAGTTGGAGTTAACTGTCGCTGTCAGTTAGAGTTAACTGTCGCTGTCAGTTGGAGTTAACCGTTTAAACTGTGCCTCTCCCGTGACGTCATCTGGGGTGATGTGCAAACCACCAATCCCG
GCTCTACACATCTCACTCGGAGCTCCTAGGCTTATATAAGGGGCTGGGTTTCTTAGCTTGGGGTCTCCCTCTAAGAAGCTGATCATCTATCTCTCAAGATGCATTAAAGCTTTACTGCAGAAGGATCCGAGTGTTCTGCGTCGTTCTTGCTGGCGAGACGGTAGCGCGGGACATCTGGTGCCGAAACCCGGGAACTCTTCAACATCGCCGGCGCCGCGGGAGACCC
CTCGGAAAACGGGGCGGATTCAGAACTGCAGGGACGTAGGTTCAGAGAGGTATGCTTTATCCTGAACAACCCTTTTCTCGACTTTGGCCTTAGTTTGTCATCACCGTGGGACGGGTCAGTGGAGGCTTTGGTGCTAGTCCTGTTTGTCCTTCTCCTTCATCGTGGCTGGTGCTGACAGAACGGCGCTGTCCGTCTGGCGCTGTCCATCTGGCGCTGTCCATCTGGC
GCTGTCCATCTGGCGCTGTCAGTCTGGTGCTGTCAGTCTGGTGCTGTCAGTCTGGTGCTGTCAGTCTGGTAACGGAGTTGAGCGCGTTTTTTAGATAAGCTGTCAGCGCGATGGGGTCGTCACAGTCAGTGGTTACAGCCTTACAGACTGTGCTAAAACAAAGAGATCTTAAAATTGCACCACGAACACTTCAAAATTTTGTGAAGGAGGTAGATAGAGTGGCACC
ATGGTATGCTTGTTCCGGGTCCCTCACTGTGGCCTCATGGAACAAGCTAGGTAAAGAACTAGACAGGAAGCATCAGAGGAGATCTCTGCTTAGGTACCAAAGCCATTTGGAAGCTAGTTAAAAACTGCTTGGAGGATGAGGCCTGTCACCCTGCTATAATAGAAAGTCAGGGAACCTTAGAGGAAGTTCAGGATAGCATGTCAGAAACTGAACGGAGTGAGAGA
ATGGGAGCTCGCAAAGAAAAGACATGTCTAAGAAAAAGGCCTCCCCAGGAAGTTAAGAAAGGGGGAGAGAAAGAAGGGAGTTATCATTCACCCCTTAACAAATCTAAGAAAAAGAAAAAGCCAGAATCTAGTCTATACCCTGCAGTAGAGCTAGAAGCCTTGGAGCTAGATAATTCTGATTCTGACACTCTGGATTCTAGCGAAGAAGGTGGTCTAGAGGAGG
AGGTGGCACGGTATGAGGAAAAAAGATATCACCCTGATAGACACCGGCCACTGAAAACAAAAATGAATGTGAGGCCACCGCCTATAAATCCGGCGGGCTCACGCCCCTCAGCGCCTCCGCCATATGAGCTTCGGCTGAATACTGGTACTGACTCCTTTTTGCCTTTAGAGGAACGGAGAAAAATACAAATGGCTTTTCCAGTATTCGAGAATGTGGAAGGCGGAAG
AGTACATACCCCCGTAGATTACAATCAAATTAAAGAATTGGCCGAATCCGTTCGTAATTATGGGGTCAATGCTAACTTTACCACATTACAGGTGGAAAGGCTTGCTAATTTTGCCATGACTCCCACTGACTGGCAAACGACAGTGAAAGCAGTGCTTCCCAACATGGGACAATACATGGAATGGAAGGCTCTCTGGTATGATGCAGCCCAGGCACAAGCTAGAGTT
AATGCCACGGCTGAAGATGAGAATCAGAGGCAGTGGACATTTGAAATGCTGACAGGTCAGGGTCAACATGCCCTTAATCAAACCAACTACATCTGGGGTGTCTATGGTCAGGTGTCGGCCGCCGCTATTAAAGCATGGAAGGCCCTCACAAAAAGGGGAGAGTCCAGTGGACACCTTACAAAGATTGTCCAAGGACCCCAAGAGCCATTTTCAGACTTTGTGGCCA
GAATGACAGAGGCCGCCAGTCGCATCTTTGGTGATTCAGAACAAGCTATGCCCCTGATTGAGCAACTTGTCTATGAACAAGCAACCCAGGAATGTCGAGCGGCTATCGCCCCTCGCAAAAGCAAAGGTTTACAAGATTGGCTCAGAGTGTGCAGGGAGCTGGGGGGACCCCTAACCAATGCAGGACTAGCTGCCGCCATTTTACAAACGCACAGGTATAGAGATTT
ATCTAATCGAAAAGCTTGTTTTAATTGTGGGAGAATGGGGCACCTTAAGAAAGATTGTCAAGCCCCTGAAAGAACAAGAGAATCGAAGCTTTGCTATCGTTGTGGTAAAGGTTATCATAGGGCTAGCGAATGCAGGTCAGTCCGGGATGTAAAAGGGAGGCTTCTGCCCCCTATTGAAGAATCCAAGGTACCCCAATCAAAAAACGGGTCACGGGGCCCGTGGTCC
CAGGGCCCTCAGAAATATGGGAACCAATTTCAGAAAAGCAATCAAGAGGAAGTAGAGGTTTCCGAGGAACCTCCGGAATGGACTTGCGTGCCGCCTCCGACTTCTTACTCATGCCCCAAATGAATGTTCAACCGGTCCCAGTCCATTCCCCTGGACCTTTGCCCCCTGCCACCATTGGGCTTATTCTGGGCCGTGGCTCCTTAACTTTACAGGGACTCATTATATA
TCCTGGGATTGTTGACCCATATCATAAAGAGGAGATTCAAGTTCTCTGTTCTAGCCCTCGGGGTGTATTTTCTATAAAGCAGGGAGATAGAATTGCACAACTGGTGTTGCTGCCCTCCCTCGGGGATGGAGAAACCTACACTTTACAAAAAAGGGCTATGGGATCTTCTGGAAGTGATTCAGCCTATCTGGCTATACCCCTAAATGAGAGACCTACAATAAAATTG
TTAATTAATGGAAAAGAATTTGAAGGGATAATGGATTCGGGAGCAGACAAAAGCATTATTTCACTCCATTGGTGGCCGAAGTCTTGGCCCACTGTTGTTTCATCTCATTCTCTACAAGGTCTTGGATACCAGTCCTCTCCTGCCATCAGTGCCTCAGCCTTAACCTGGCGGGATGCTGAAGGCAAACAGGGATGTTTTACCCCCTATGTGTTGCCACTCCCTGTAA
ATTTATGGGGACGAGATGTGTTACAAGCCATGGGCATGACCCTAACTAATGAATACTCCCCCAGGCATCAGCCATTATGGCAAAGATGGGGTATACAAATGGTAGAGGCTTGGGTAGGCAAGAACAAGGCAGAATAAAACCCATTACACAACACGGAAATCGGGGTAGAAAAGGACTGGGTTTTATTTAGGGGCCGTTGAGGCTTCACGACCCATACCATGGAAA
ACAGAGGAGCCGTTATGGGTCTCTCAATGGCCTCTATCCTCTGAAAAATTAGAGGCTGTCACAAGATTAGTGCAAGAACAGGAACGGCTGGGGCATCTAGAGCCTTCTACCTCCCCATGGAATACACCAATTTTTGTTATTAAGAAGAAATCTGGGAAGTGGAGATTACTCCACGACCTGCGGGCCATTAACAATCAGATGCATCTTTTGGCCCTGTTCAAAGAG
GCCTTCCTTTGCTTTCTGCACTTCCTCAAGATTGGAAGCTTATTATTATAGATATTAAGGATTGTTTCTTCTCTATTCCACTTTACCCACGGGATAGACCAAGGTTTGCCTTCACTATCCCTTCTCTTAATCATATGGAACCAGACAAGAGATTTCAGTGGAAGGTATTGCCGCAAGGCATGGCCAATAGCCCAACAATATGTCAGCTATATGTGCAGGAGGCTTT
GGAGCCAATTAGGAAGCAATTTACATCTTTAATCGTTATCCATTATATGGACGATATTCTTATTTGTCACAAAGAGTTAGATGTTTTACAAAAGGCTTTTCCCATGCTGGTGGCTGAATTAAAACAGTGGGGATTAGAGATTGCCTCAGAAAAGGTTCAAATTGCAGATACAGGCCTCTTTTTGGGCTCAATGATTACTCCCAAAAATATAGTTCCTCAGAAAATA
GAAATTCGCAAAGACCACTTACAAACATTAAATGATTTTCAGAAACTTCTGGGAGATATAAATTGGTTGAGGCCCTTTTTAAAAATTCCATCTGCTGATCTAAAACCCCTCTTCGATCTCCTGGAGGGTGAGCCTCATATCTCCTCACCCAGAAAATTTACCCCTGCCGCCCACCGAGCCTTACAGATGGTGGAAGAGGCCTTACAGGAAGCACAATTACAACGAA
TTGAGGAGTCACTACCTTTTAATCTGTGTGTTTTTAAGACAGCTCAATTGCCAACTGCAGTCCTGTGGCAACATGGACCATTACTTTGGATTCATCCAAATGCTTCCCCTGCTAAGATTATTGACTGGTACCCTGATGCTGTTGCACAGCTCGCGCTTCGCGGAATAAAAGCAGCTGTTACACATTTTGGCAGGGATCCTGATTCCTTGATTGTGCCTTATACTGC
TGCACAAGTTCAGACCTTGGCAGCCACATCTAGTGATTGGGCAGTTTTAGTTACTTCCTTTTCAGGACAAATTGATAATCATTTTCCAAAGCATCCAATTTTACAGTTTGCCCTAAACCAGGCTATAGTGTTTCCACAAGTGACAGCTAAAGACCCACTCCCAGATGGGACTGTGGTGTACACTGATGGATCAAAAACTGGTATGGGAGCTTATGTGGTCAAGGAT
AGGGTTATATCTAAACAATATAATGAAACTTCACCTCAAGTTGTAGAATGTCTGATAGTACTGGAGGTTCTTGAGGCTTTTCCAGGACCCCTCAATATTGTATCAGATTCCTCTTATGTGGTTAATGCAGTTAATCTCCTTGAGATAGCTGGAATAATAAGATCCTCCAGCAGAGTTGCTAACATCTTTCAGAAGATACAAGCTGCCTTGTTAAATAGGAGATTTC
CTGTTTTCATTACCCATGTCCGAGCACATTCTGGACTCCCAGGTCCCATGTCCTTAGGGAATGACTTAGCGGACAAGGCTACAAAGCTGGTGGCCGCTGCCTTGTCTACCCATGCACAAGCAGCAAAAGAATTCCATAAGCGCTTTCATGTGACGGCTGAGACCTTGCGCCGTCGTTTTGCTCTATCCAGGAAAGAGGCTAGGGAAATTGTTACTCAATGCCAGAA
TTGCTGCGAATTCTTACCTACTCCCCACATGGGAATAAACCCACGTGGCATTAGACCATTGCAGATGTGGCAGATGGATGTTACTCATATTCCCTCTTTTGGAAGGCTTCAATATGTTCATGTTTCTGTTGACACCTGTTCTGGCGTAATGTTTGCCACACCCCTAACAGGGGAAAAGGCCTCATATGTGATTCAACATTGCCTTGAGGCCTGGAGTGCTTGGGGA
AAACCCAGAATTTTAAAAACAGACAATGGACCAGCATATACCTCTCAAAAATTCCGACAGTTTTGTCGGCAAATGGATGTTACCCATCTAACTGGCCTACCTTATAACCCTCAAGGACAGGGCATTGTGGAACGTGCCCATCGTACACTCAAATCATATCTGATTAAACAAAAGGGGGAGTATTGAGGATGTTCTACCCTCGGTACCAAGAGTTGCTGTATCCATGG
CACTTTTTACCCTTAATTTCTTGAACTGTGATGCCCAAGGCCACACTGCGGCTGATCGCCACAGTTTAGAGCCTGATAGGCCAAAAGAATTTGTAAAATGGAAAGATGTGCTAACTAATGAATGGAAAGGCCCGGATCCTATTCTTATAAGATCCAGGGGAGCTGTGTGTGTTTTTCCACAGGAGGAAGAAAACCCCCTGTGGATCCCTGAAAGACTCACTCGAAG
AGCTCCTTCACAACTCCAGAATAAGAAGGATGGAGGCCAAGTGGGTGATGAAGACTCTCCTACTCGGGATGATGGTTCCTAGTATGATGGGAGAGTTACGGTGGGCCATCATGTCTACCTTCCCCCTACCGATGCCAGTGATGCAGAATGCCCAGGTGTTCCCTAGATTTTTCACAACTAATAGAGAGTTGGGGCTTGCCTTTTTGCCTTTAGATCCTCAGATACA
GAGTTTTCAGGATGAGAGAACCTTGTCATTAGTAGGAAGCTTATGCTTTACTGTGATTTCTTCATTAAAGGATAATTTTATTCATATGGATCCAGAAAGATCTAATGCTAATCCTTTTGAATTAATGGAAGCTGTACTGATCTTTCTCCTATGATAGTGAGAAGGGACAGATTACTTTTGATTGGAAAGGGGCATTAGGCAACAGTCTGAGTGAGGGACAGAGCTC
TGGATCATCTGATTGGTCCTCCTCCAATGTTAAGTATACCCCTTTTAAGAGAACTAATTATTCAGCTGCCCTTTATTTGGGTTGTGAGTAATGATACCTTAGGTAAGAATCAGAAAGAAATAAATTGTTCTGCTTAGAATTGTTTTTATGCATTATGCTGGGATGCTATTAAGCACCCTTTTGCTTTGGTAATGCGCATGCCTCGATTTATTCCAGCTCCTGTTGA
GGCGCCAAGTAATTTGAGTTTGTTTAGGGAAAAGCGAGATTTTTGGGATCTCTGCTATTATTGTGGGCCTGATAGCCACCGCCGCGGTTACCGCCTCGGCCCTTGCACTTTCTGCCACTGTGCAGACAACTCAAACCATTAATGAACTATAGGCCACTGTGACCATGGTCCTAGATAAACAGGCCACTGCCAATTCTCAGATACAAGGAGGCCTGATGTTGGTAAA
CCAACAGATTAATTGGTTCAGGAACAATTGGATATATTGTGGCAGATGGCCAAATTGGGATGTGAACAAAGTTGCCTGGCCTTTGTGTCACTTAGTACAATATGAGAATTTTACAAGAGCAGCTAACCTGTCAAAAGCTCTGTCTCAACATTTATTACAGAATTGGACTTTTGAGTTTGAGCAGACACTCCGTGAGTTGAGAATGGCGATCCTGCAAGTGAATTC
GACGCATCTGGATCTTTCTCTGATGGAAGACCTCTCCCCCTGGATTTCATCTGCATTTTCTTATTTTAAGGAATGGGTGGGGGTAGGATCATTTAGCCTAGTTCTCTGCTGTGGAATAGTGTTGATGCTTTGGATGGTGTGTACGCTCAGAGCCCAAACAAAAGGAGAAAGTGGTGGTGGCACAAGCACTTGCTGCCTTAGAACACGGAGCATCCCCTGACATTTG
GTTAACCATGTTAAAGCAATAGGTCGCTGGCCTCCCAGCTCTTGCACCCCACGAGGCTAGTCTCATTGCACGGGATAGAGTGGGTAGGCTTCAGCAGCCCAAGGAGTTGCAAGGCTAAGCACTGCACAGGAGAGGTCTGCGGTATAACGACTTTCTCCTGGGAGATAAGTCATCTTGCATGAAGGTTCTATGTCATGATCTCCTTCCCCCAGAAAAAAGACATCAG
ACTGGTCAGGACTTCCTCTGGGGATAAGACCCTGGAGAGGAGCTTTGTACATGTTCCGAATTTGCACATGGGGATTTGACCTCTATCTCCACTCCAAAGTTGTGGGTGTCCTATTGCTTCAAAAATAAATGAATAGGGGGAGATGTGAGGAGCCGCCCTCGCTATCGCTATTGCCGTTAGAAGATGGCGCTGACATCCACTGTCAGTTGGAGTTAACTGTCGCTGT
CAGTTGGAGTTAACTGTCGCTGTCAGTTGGAGTTAACTGTCGCTGTCAGTTAGAGTTAACTGTCGCTGTCAGTTGGAGTTAACCGTTTAAACTGTGCCTCTCCCGTGACGTCATCTGGGGTGATGTGCAAACCACCAATCCCGGCTCTACACATCTCACTCGGAGCTCCTAGGCTTATATAAGGGGCTGGGTTTCTTAGCTTGGGGTCTCCCTCTAAGAAGCTGAT
CATCTATCTCTCAAGATGCATTAAAGCTTTACTGCAGAAGGATCCGAGTGTTCTGCGTCGTTCTTGCTGGCGAGACGGTAGCGCGGGACA

>IAPLTR3.1812487
TGTGAGGAGCCGCCCTCGCTATCGCTATTGCCGTTAGAAGATGGCGCTGACATCCACTGTCAGTTGGAGTTAACTGTCGCTGTCAGTTGGAGTTAACCGTTTAAACTGTGCCTCTCCCGTGACGTCATCTGGGGTGATGTGCAAACCACCAATCCCGGCTCTACACATCTCACTCGGAGCTCCTAGGCTTATATAAGGGGCTGGGTTTCTTAGCTTGGGGTCTCCC
TCTAAGAAGCTGATCATCTATCTCTCAAGATGCATTAAAGCTTTACTGCAGAAGGATCCGAGTGTTCTGCGTCGTTCTTGCTGGCGAGACGGTAGCGCGGGACATCTGGTGCCGAAACCCGGGAACTCTTCAACATCGCCGGCGCCGCGGGAGACCCCTCGGAAAACGGGGCGGATTCAGAACTGCAGGGACGTAGGTTCAGAGAGGTATGCTTTATCCTGAACAA
CCCTTTTCTCGACTTTGGCCTTAGTTTGTCATCACCGTGGGACGGGTCAGTGGAGGCTTTGGTGCTAGTCCTGTTTGTCCTTCTCCTTCATCGTGGCTGGTGCTGACAGAACGGCGCTGTCCGTCTGGCGCTGTCCATCTGGCGCTGTCCGTCTGGCGCTGTCCGTCTGGCGCTGTCCATCTGGCGCTGTCCATCTGGCGCTGTCAGTCTGGTGCTGTCAGTCTGG
TGCTGTCAGTCTGGTGCTGTCAGTCTGGTAACGGAGTTGAGCGCGTTTTTTAGATAAGCTGTCAGCGCGATGGGGTCGTCACAGTCAGTGGTTACAGCCTTACAGACTGTGCTAAAACAAAGAGATCTTAAAATTGCACCACGAACACTTCAAAATTTTGTGAAGGAGGTAGATAGAGTGGCACCATGGTATGCTTGTTCCGGGTCCCTCACTGTGGCCTCATGGA
ACAAGCTAGGTAAAGAACTAGACAGGAAGCATACAGAGGAGATCTCTGCTTAGGTACCAAAGCCATTTGGAAGCTAGTTAAAAACTGCTTGGAGGATGAGGCCTGTCACCCTGCTATAATAGAAAGTCAGGGAACCTTAGAGGAAGTTCAGGATAGCATGTCAGAAACTGAACGGAGTGAGAGAATGGGAGCTTGCAAAAGAAAAGACATGTCTAAGAAAAAGG
CCCTCCCCAGGAAGTTAAGAAAGGGGGAGAGAAAGAAGGGAGTTATCATTCACCCCTTAACAAATCTAAGAAAAAGAAAAAGCCAGAATCTAGTCTATACCCTGCAGTAGAGCTAGAAGCCTTGGAGCTAGATAATTCTGATTCTGACACTCTGGATTCTAGCGAAGAAGGTGGTCTAGAGGAGGAGGTGGCACGGTATGAGGAAAAAAGATATCACCCTGATAGA
CACCGGCCACTGAAAACAAAAATGAATGTGAGGCCACCGCCTATAAATCCGGCGGGCTCACGCCCCTCAGCGCCTCCGCCATATGAGCTTCGGCTGAATACTGGTACTGACTCCTTTTTACCTTTAGAGGAACGGAGAAAAATACAAATGGCTTTTCCAGTATTCGAGAATGTGGAAGGCGGAAGAGTACATACCCCCGTAGATTACAATCAAATTAAAGAATTGG
CCGAATCCGTTCGTAATTATGGGGTCAATGCTAACTTTACCACATTACAGGTGGAAAGGCTTGCTAATTTTGCCATGACTCCCACTGACTGGCAAACGACAGTGAAAGCAGTGCTTCCCAACATGGGACAATACATGGAATGGAAGGCTCTCTGGTATGATGCAGCCCAGGCACAAGCTAGAGTTAATGCCACGGCTGAAGATGAGAATCAGAGGCAGTGGACATT
TGAAATGCTGACAGGTCAGGGTCAACATGCCCTTAATCAAACCAACTACATCTGGGGCGTCTATGGTCAGGTGTCGGCCGCCGCTATTAAAGCATGGAAGGCCCTCACAAAAAGGGGAGAGTCCAGTGGACACCTTACAAAGATTGTCCAAGGACCCCAAGAGCCATTTTCAGACTTTGTGGCCAGAATGACAGAGGCCGCCAGTCGCATCTTTGGTGATTCAGAA
CAAGCTATGCCCCTGATTGAGCAACTTGTCTATGAACAAGCAACCCAGGAATGTCGAGCGGCTATCGCCCCTCGCAAAAGCAAAGGTTTACAAGATTGGCTCAGAGTGTGCAGGGAGCTGGGGGGACCCCTAACCAATGCAGGACTAGCTGCCGCCATTTTACAAACGCACAGGTATAGAGATTTATCTAATCGAAAAGCTTGTTTTAATTGTGGGAGAATGGGGC
ACCTTAAGAAAGATTGTCAAGCCCCTGAAAGAACAAGAGAATCGAAGCTTTGCTATCGTTGTGGTAAAGGTTATCATAGGGCTAGCGAATGCAGGTCAGTCCGGGATGTAAAAGGGAGGCTTCTGCCCCCTATTGAAGAATCCAAGGTACCCCAATCAAAAAACGGGTCACGGGGCCCATGGTCCCAGGGCCCTCAGAAATATGGGAACCAATTTCAGAAAAGCAA
TCAAGAGGAAGTAGAGGTTTCCGAGGAACCTCCGGAATGGACTTGCGTGCCGCCTCCGACTTCTTACTCATGCCCCAAATGAATGTTCAACCGGTCCCAGTCCATTCCCCTGGACCTTTGCCCCCTGCCACCATTGGGCTTATTCTGGGCCGTGGCTCCTTAACTTTACAGGGACTCATTATATATCCTGGGATTGTTGACCCATATCATAAAGAGGAGATTCAAG
TTCTCTGTTCTAGCCCTCGGGGTGTATTTTCTATAAAGCAGGGAGATAGAATTGCACAACTGGTGTTGCTGCCCTCCCTCGGGGATGGAGAAACCTACACTTTACAAAAAAGGGCTATGGGATCTTCTGGAAGTGATTCAGCCTATCTGGCTATACCCCTAAATGAGAGACCTACAATAAAATTGTTAATTAATGGAAAAGAATTTGAAGGGATAATGGATTCGGG
AGCAGACAAAAGCATTATTTCACTCCATTGGTGGCCGAAGTCTTGGCCCACTGTTGTTTCATCTCATTCTCTACAAGGTCTTGGATACCAGTCCTCTCCTGCCATCAGTGCCTCAGCCTTAACCTGGCGGGATGCTGAAGGCAAACAGGGATGTTTTACCCCCTATGTGTTGCCACTCCCTGTAAATTTATGGGGACGAGATGTGTTACAAGCCATGGGCATGACC
CTAACTAATGAATACTCCCCCAGGCATCAGCCATTATGGCAAAGATGGGGTATACAAATGGTAGAGGCTTGGGTAGGCAAGAACAAGGCAGAATAAAACCCATTACACAACACGGAAATCGGGGTAGAAAAGGACTGGGTTTTATTTAGGGGCCGTTGAGGCTTCACGACCCATACCATGGAAAACAGAGGAGCCGTTATGGGTCTCTCAATGGCCTCTATCCTC
TGAAAAATTAGAGGCTGTCACAAGATTAGTGCAAGAACAGGAACGGCTGGGGCATCTAGAGCCTTCTACCTCCCCATGGAATACACCAATTTTTGTTATTAAGAAGAAATCTGGGAAGTGGAGATTACTCCACGACCTGCGGGCCATTAACAATCAGATGCATCTTTTGGCCCTGTTCAAAGAGGCCTTCCTTTGCTTTCTGCACTTCCTCAAGATTGGAAGCTT
ATTATTATAGATATTAAGGATTGTTTCTTCTCTATTCCACTTTACCCACGGGATAGACCAAGGTTTGCCTTCACTATCCCTTCTCTTAATCATATGGAACCAGACAAGAGATTTCAGTGGAAGGTACTGCCGCAAGGCATGGCCAATAGCCCAACAATATGTCAGCTATATGTGCAGGAGGCTTTGGAGCCAATTAGGAAGCAATTTACATCTTTAATCGTTATCC
ATTATATGGACGATATTCTTATTTGTCACAAAGAGTTAGATGTTTTACAAAAGGCTTTTCCCATGCTGGTGGCTGAATTAAAACAGTGGGGATTAGAGATTGCCTCAGAAAAGGTTCAAATTGCAGATACAGGCCTCTTTTTGGGCTCAAAGATTACTCCCAAAAATATAGTTCCTCAGAAAATAGAAATTCGCAAAGACCACTTACAAACATTAAATGATTTTCA
GAAACTTCTGGGAGATATAAATTGGTTGAGGCCCTTTTTAAAAATTCCATCTGCTGATCTAAAACCCCTCTTCGATCTCCTGGAGGGTGAGCCTCATATCTCCTCACCCAGAAAATTTACCCCTGCCGCCCACCGAGCCTTACAGATGGTGGAAGAGGCCTTACAGGAAGCACAATTACAACGAATTGAGGAGTCACTACCTTTTAATCTGTGTGTTTTTAAGACA
GCTCAATTGCCAACTGCAGTCCTGTGGCAACATGGACCATTACTTTGGATTCATCCAAATGCTTCCCCTGCTAAGATTATTGACTGGTACCCTGATGCTGTTGCACAGCTCGCGCTTCGCGGAATAAAAGCAGCTGTTACACATTTTGGCAGGGATCCTGATTCCTTGATTGTGCCTTATACTGCTGCACAAGTTCAGACCTTGGCAGCCACATCTAGTGATTGGG
CAGTTTTAGTTACTTCCTTTTCAGGACAAATTGATAATCATTTTCCAAAGCATCCAATTTTACAGTTTGCCCTAAACCAGGCTATAGTGTTTCCACAAGTGACAGCTAAAGACCCACTCCCAGATGGGACTGTGGTGTACACTGATGGATCAAAAACTGGTATGGGAGCTTATGTGGTCAAGGATAGGGTTATATCTAAACAATATAATGAAACTTCACCTCAAGT
TGTAGAATGTCTGATAGTACTGGAGGTTCTTGAGGCTTTTCCAGGACCCCTCAATATTGTATCAGATTCCTCTTATGTGGTTAATGCAGTTAATCTCCTTGAGATAGCTGGAATAATAAGATCCTCCAGCAGAGTTGCTAACATCTTTCAGAAGATACAAGCTGCCTTGTTAAATAGGAGATTTCCTGTTTTCATTACCCATGTCCGAGCACATTCTGGACTCCCA
GGTCCCATGTCCTTAGGGAATGACTTAGCGGACAAGGCTACAAAGCTGGTGGCCGCTGCCTTGTCTACCCATGCACAAGCAGCAAAAGAATTCCATAAGCGCTTTCATGTGACGGCTGAGACCTTGCGCCGTCGTTTTGCTCTATCCAGGAAAGAGGCTAGGGAAATTGTTACTCAATGCCAGAATTGCTGCGAATTCTTACCTACTCCCCACATGGGAATAAACC
CACGTGGCATTAGACCATTGCAGATGTGGCAGATGGATGTTACTCATATTCCCTCTTTTGGAAGGCTTCAATATGTTCATGTTTCTGTTGACACCTGTTCTGGCGTAATGTTTGCCACACCCCTAACAGGGGAAAAGGCCTCATATGTGATTCAACATTGCCTTGAGGCCTGGAGTGCTTGGGGAAAACCCAGAATTTTAAAAACAGACAATGGACCAGCATATAC
CTCTCAAAAATTCCGACAGTTTTGTCGGCAAATGGATGTTACCCATCTAACTGGCCTACCTTATAACCCTCAAGGACAGGGCATTGTGGAACGTGCCCATCGTACACTCAAATCATATCTGATTAAACAAAAGGGGGAGTATTGAGGATGTTCTACCCTCGGTACCAAGAGTTGCTGTATCCATGGCACTTTTTACCCTTAATTTCTTGAACTGTGATGCCCAAGGC
CACACTGCGGCTGATCGCCACAGTTTAGAGCCTGATAGGCCAAAAGAAATGGTAAAATGGAAAGATGTCTTGACTGATCTCTGGAAAGGCCCGGATCCTATTCTTATAAGATCCAGGGGAGCTGTGTGTGTTTTTCCACAGGAGGAAGAAAACCCCCTGTGGATCCCTGAAAGACTCACTCGAAGAGCTCCTTCACAACTCCAGAATAAGAAGGATGGAGGCCAAG
TGGGTGATGAAGACTCTCCTACTCGGGATGATGGTTCCTAGTATGATGGGAGAGTTACGGTGGGCCATCATGTCTACCTTTCCCCCTACCGATGCCAGTGATGCAGAATGCCCAGGTGTTCCCTAGATTTTTCACAAATAATAGAGAGTTGGGGCTTGCCTTTTTGCCTTTAGATCCTCAGATACAGAGTTTTCAGGATGAGAGAACCTTGTCATTAGTAGGAAGC
TTGTGCTTTACTGTGATTTCTTCATTAAAGGATAATTTTATTCATATGGATCCAGAAAGATCTAATGCTAATCCTTTTGAATTAATGGAAGCTGTACTGATCTTTCTCCTATGATAGTGAGAAGGGACAGATTACTTTTGATTGGAAAGGGGCATTAGGCAACAGTCTGAGTGAGGGACAGAGCTCTGGATCATCTGATTGGTCCTCCTCCAATGTTAAGTATACC
CCTTTTAAGAGAACTAATTATTCAGCTGCCCTTTATTTGGGTTGTGAGTAATGATACCTTAGGTAAGAATCAGAAAGAAATAAATTGTTCTGCTTAGAATTGTTTTTATGCATTATGCTGGGATGCTATTAAGCACCCTTTTGCTTTGGTAATGCGCATGCCTCGATTTATTCCAGCTCCTGTTGAGGCGCCAAGTAATTTGAGTTTGTTTAGGGAAAAGCGAGAT
TTTTGGGATCTCTGCTATTATTGTGGGCCTGATAGCCACCGCCGCGGTTACTGCTTCGGTTACCGCCTCGGCCCTTGCACTTTCTGCCACTGTGCAGACAACTCAAACCATTAATGAACTATAGGCCACTGTGACCATGGTCCTAGATAAACAGGCCACTGCCAATTCTCAGATACAAGGAGGCCTGATGTTGGTAAACCAACAGATTAATTGGTTCAGGAACAA
TTGGATATATTGTGGCAGATGGCCAAATTGGGATGTGAACAAAGTTGCCTGGCCTTTGTGTCACTTAGTACAATATGAGAATTTTACAAGAGCAGCTAACCTGTCAAAAGCTCTGTCTCAACATTTATTACAGAATTGGACTTTTGAGTTTGAGCAGACACTCCGTGAGTTGAGAATGGCGATCCTGCAAGTGAATTCGACGCATCTGGATCTTTCTCTGATGGAA
GACCTCTCCCCCTGGATTTCATCTGCATTTTCTTATTTTAAGGAATGGGTGGGGGTAGGATCATTTAGCCTAGTTCTCTGCTGTGGAATAGTGTTGATGCTTTGGATGGTGTGTACGCTCAGAGCCCAAACAAAAGGAGAAAGTGGTGGTGGCACAAGCACTTGCTGCCTTAGAACACGGAGCATCCCCTGACATTTGGTTAACCATGTTAAAGCAATAGGTCGCT
GGCCTCCCAGCTCTTGCACCCCACGAGGCTAGTCTCATTGCACGGGATAGAGTGGGTAGGCTTCAGCAGCCCAAGGAGTTGCAAGGCTAAGCACTGCACAGGAGAGGTCTGCGGTATAACGACTTTCTCCTGGGAGATAAGTCATCTTGCATGAAGGTTCTATGTCATGATCTCCTTCCCCCAGAAAAAAGACATCGGACTGGTCAGGACTTCCTCTGGGGATAAG
ACCCTGGAGAGGAGCTTTGTACATGTTCCGAATTTGCACATGGGGATTTGACCTCTATCTCCACTCCAAAGTTGTGGGTGTCCTATTGCTTCAAAAATAAATGAATAGGGGGAGATGTGAGGAGCCGCCCTCGCTATCGCTATTGCCGTTAGAAGATGGCGCTGACATCCACTGTCAGTTGGAGTTAACTGTCGCTGTCAGTTGGAGTTAACCGTTTAAACTGTGC
CTCTCCCGTGACGTCATCTGGGGTGATGTGCAAACCACCAATCCCGGCTCTACACATCTCACTCGGAGCTCCTAGGCTTATATAAGGGGCTGGGTTTCTTAGCTTGGGGTCTCCCTCTAAGAAGCTGATCATCTATCTCTCAAGATGCATTAAAGCTTTACTGCAGAAGGATCCGAGTGTTCTGCGTCGTTCTTGCTGGCGAGACGGTAGCGCGGGACA

>IAPLTR3.1073540
TGTGAGGAGCCGCCCTCGCTATCGCTATTGCCGTTAGAAGATGGCGCTGACATCCACTGTCAGTTAGAGTTAACTGTCGCTGTCAGTTGGAGTTAACCGTTTAAACTGTGCCTCTCCCGTGACGTCATCTGGGGTGATGTGCAAACCACCAATCCCGGCTCTACACATCTCACTCGGAGCTCCTAGGCTTATATAAGGGGCTGGGTTTCTTAGCTTGGGGTCTCCC
TCTAAGAAGCTGATCATCTATCTCTCAAGATGCATTAAAGCTTTACTGCAGAAGGATCCGAGTGTTCTGCGTCGTTCTTGCTGGCGAGACGGTAGCGCGGGACATCTGGTGCCGAAACCCGGGATCTCTTCAACATCGCCGGCGCCGCGGGAGACCCCTCGGAAGACGGGGCGGATTCAGAACTGCAGGGACGTAGGTTCAGAGAGGTATGCTTTATCCTGAACAA
CCCTTTTCTCGACTTTGGCCTTAGTTTGTCATCACCGTGGGACGGGTCAGTGGAGGCTTTGGTGCTAGTCCTGTTTGTCCTTCTCCTTCATCGTGGCTGGTGCTGACAGAACGGCGCTGTCCATCTGGCGCTGTCCGTCTGGCGCTGTCCATCTGGCGCTGTCCATCTGGCGCTGTCCGTCTGGCGCTGTCAGTCTGGTGCTGTCAGTCTGGTGCTGTCAGTCTGG
TGCTGTCAGTCTGGTAACGGAGTTGAGCGCGTTTTTTAGATAAGCTGTCAGCGCGATGGGGTCGTCACAGTCAGTGGTTACAGCCTTACAGACTGTGCTAAAACAAAGAGATCTTAAAATTGCACCACGAACACTTCAAAATTTTATGAAGGAGGTAGATAGAGTGGCACCATGGTATGCTTGTTCCGGGTCCCTCACTGTGGCCTCATGGAACAAGCTAGGTAAA
GAACTAGACAGGAAGCATCAGAGGAGATCTCTGCTTAGGTACCAAAGCCATTTGGAAGCTAGTTAAAAACTGCTTGGAGGATGAGGCCTGTCACCCTGCTATAATAGAAAGTCAGGGAACCTTAGAGGAAGTTCAGGATAGCATGTCAGAAACTGAACGGAGTGAGAGAATGGGAGCTCGCAAAAGAAAAGACATGTCTAAGAAAAAGGCCCTCCCCAGGAAG
TTAAGAAAGGGGGAGAGAAAGAAGGGAGTTATCATTCACCCCTTAACAAATCTAAGAAAAAGAAAAAGCCAGAATCTAGTCTATACCCTGCAGTAGAGCTAGAAGCCTTGGAGCTAGATAATTCTGATTCTGACACTCTGGATTCTAGCGAAGAAGGTGGTCTAGAGGAGGAGGTGGCACGGTATGAGGAAAAAAGATATCACCCTGATAGACACCGGCCACTGAA
AACAAAAATGAATGTGAGGCCACCGCCTATAAATCCGGCGGGCTCACGCCCCTCAGCGCCTCCGCCATATGAGCTTCGGCTGAATACTGGTACTGACTCCTTTTTACCTTTAGAGGAACGGAGAAAAATACAAATGGCTTTTCCAGTATTCGAGAATGTGGAAGGCGGAAGAGTACATACCCCCGTAGATTACAATCAAATTAAAGAATTGGCCGAATCCGTTCGT
AATTATGGGGTCAATGCTAACTTTACCACATTACAGGTGGAAAGGCTTGCTAATTTTGCCATGACTCCCACTGACTGGCAAACGACAGTGAAAGCAGTGCTTCCCAACATGGGACAATACATGGAATGGAAGGCTCTCTGGTATGATGCAGCCCAGGCACAAGCTAGAGTTAATGCCACGGCTGAAGATGAGAATCAGAGGCAGTGGACATTTGAAATGCTGACAG
GTCAGGGTCAACATGCCCTTAATCAAACCAACTACATCTGGGGCGTCTATGGTCAGGTGTCGGCCGCCGCTATTAAAGCATGGAAGGCCCTCACAAAAAGGGGAGAGTCCAGTGGACACCTTACAAAGATTGTCCAAGGACCCCAAGAGCCATTTTCAGACTTTGTGGCCAGAATGACAGAGGCCGCCAGTCGCATCTTTGGTGATTCAGAACAAGCTATGCCCCT
GATTGAGCAACTTGTCTATGAACAAGCAACCCAGGAATGTCGAGCGGCTATCGCCCCTCGCAAAAGCAAAGGTTTACAAGATTGGCTCAGAGTGTGCAGGGAGCTGGGGGGACCCCTAACCAATGCAGGACTAGCTGCCGCCATTTTACAAACACACAGGCGTAGAGATTTATCTAGTCGAAAAGCTTGTTTTAATTGTGGGAGAATGGGGCACCTTAAGAAAGAT
TGTCAAGCCCCTGAAAGAACAAGAGAATCGAAGCTTTGCCATCGTTGTGGTAAAGGTTATCATAGGGCTAGCGAATGCAGGTCAGTCCGGGATGTAAAAGGGAGGCTTCTGCCCCCTATTGAAGAATCCAAGGTACCCCAATCAAAAAACGGGTCACGGGGCCCATGGTCCCAGGGCCCTCAGAAATATGGGAACCAATTTCAGAAAAGCAATCAAGAGGAAGTAG
AGGTTTCCGAGGAACCTCCGGAATGGACTTGCGTGCCGCCTCCGACTTCTTACTCATGCCCCAAATGAATGTTCAACCGGTCCCAGTCCATTCCCCTGGACCTTTGCCCCCTGCCACCATTGGGCTTATTCTGGGCCGTGGCTCCTTAACTTTACAGGGACTCATTATATATCCTGGGATTGTTGACCCATATCATAAAGAGGAGATTCAAGTTCTCTGTTCTAGC
CCTCGGGGTGTATTTTCTATAAAGCAGGGAGATAGAATTGCACAACTGGTGTTGCTGCCCTCCCTCGGGGATGGAGAAACCTACACTTTACAAAAAAGGGCTATGGGATCTTCTGGAAGTGATTCAGCCTATCTGGCTATACCCCTAAATGAGAGACCTACAATAAAATTGTTAATTAATGGAAAAGAATTTGAAGGGATAATGGATTCGGGAGCAGACAAAAGCA
TTATTTCACTCCATTGGTGGCCGAAGTCTTGGCCCACTGTTGTTTCATCTCATTCTCTACAAGGTCTTGGATACCAGTCCTCTCCTGCCATCAGTGCCTCAGCCTTAACCTGGCGGGATGCTGAAGGCAAACAGGGATGTTTTACCCCCTATGTGTTGCCACTCCCTGTAAATTTATGGGGACGAGATGTGTTACAAGCCATGGGCATGACCCTAACTAATGAATA
CTCCCCCAGGCATCAGCCATTATGGCAAAGATGGGGTATACAAATGGTAGAGGCTTGGGTAGGCAAGAACAAGGCAGAATAAAACCCATTACACAACACGGAAATCGGGGTAGAAAAGGACTGGGTTTTATTTAGGGGCCGTTGAGGCTTCACGACCCATACCATGGAAAACAGAGGAGCCGTTATGGGTCTCTCAATGGCCTCTATCCTCTGAAAAATTAGAGG
CTGTCACAAGATTAGTGCAAGAACAGGAACGGCTGGGGCATCTAGAGCCTTCTACCTCCCCATGGAATACACCAATTTTTGTTATTAAGAAGAAATCTGGGAAGTGGAGATTACTCCACGACCTGCGGGCCATTAACAATCAGATGCATCTTTTGGCCCTGTTCAAAGAGGCCTTCCTTTGCTTTCTGCACTTCCTCAAGATTGGAAGCTTATTATTATAGATAT
TAAGGATTGTTTCTTCTCTATTCCACTTTACCCACGGGATAGACCAAGGTTTGCCTTCACTATCCCTTCTCTTAATCATATGGAACCAGACAAGAGATTTCAGTGGAAGGTACTGCCGCAAGGCATGGCCAATAGCCCAACAATATGTCAGCTATATGTGCAGGAGGCTTTGGAGCCAATTAGGAAGCAATTTACATCTTTAATCGTTATCCATTATATGGACGAT
ATTCTTATTTGTCACAAAGAGTTAGATGTTTTACAAAAGGCTTTTCCCATGCTGGTGGCTGAATTAAAACAGTGGGGATTAGAGATTGCCTCAGAAAAGGTTCAAATTGCAGATACAGGGCTCTTTTTGGGCTCAAAGATTACTCCCAAAAATATAGTTCCTCAGAAAATAGAAATTCGCAAAGACCACTTACAAACATTAAATGATTTTCAGAAACTTCTGGGAG

ATATAAATTGGTTGAGGCCCTTTTTAAAAATTCCATCTGCTGATCTAAAACCCCTCTTCGATCTCCTGGAGGGTGAGCCTCATATCTCCTCACCCAGAAAATTTACCCCTGCCGCCCACCGAGCCTTACAGATGGTGGAAGAGGCCTTACAGGAAGCACAATTACAACGAATTGAGGAGTCACTACCTTTTAATCTGTGTGTTTTAAGACAGCTCAATTGCCAAC
TGCAGTCCTGTGGCAACATGGACCATTACTTTGGATTCATCCAAATGCTTCCCCTGCTAAGATTATTGACTGGTACCCTGATGCTGTTGCACAGCTCGCGCTTCGCGGAATAAAAGCAGCTGTTACACATTTTGGCAGGGATCCTGATTCCTTGATTGTGCCTTATACTGCTGCACAAGTTCAGACCTTGGCAGCCACATCTAGTGATTGGGCAGTTTTAGTTACT
TCCTTTTCAGGACAAATTGATAATCATTTTCCAAAGCATCCAATTTTACAGTTTGCCCTAAACCAGGCTATAGTGTTTCCACAAGTGACAGCTAAAGACCCACTCCCAGATGGGACTGTGGTGTACACTGATGGATCAAAAACTGGTATGGGAGCTTATGTGGTCAAGGATAGGGTTATATCTAAACAATATAATGAAACTTCACCTCAAGTTGTAGAATGTCTGA
TAGTACTGGAGGTTCTTGAGGCTTTTCCAGGACCCCTCAATATTGTATCAGATTCCTCTTATGTGGTTAATGCAGTTAATCTCCTTGAGATAGCTGGAATAATAAGATCCTCCAGCAGAGTTGCTAACATCTTTCAGAAGATACAAGCTGCCTTGTTAAATAGGAGATTTCCTGTTTTCATTACCCATGTCCGAGCACATTCTGGACTCCCAGGTCCCATGTCCTT
AGGGAATGACTTAGCGGACAAGGCTACAAAGCTGGTGGCCGCTGCCTTGTCTACCCATGCACAAGCAGCAAAAGAATTCCATAAGCGCTTTCATGTGACGGCTGAGACCTTGCGCCGTCGTTTTGCTCTATCCAGGAAAGAGGCTAGGGAAATTGTTACTCAATGCCAGAATTGCTGCGAATTCTTACCTACTCCCCACATGGGAATAAACCCACGTGGCATTAGA
CCATTGCAGATGTGGCAGATGGATGTTACTCATATTCCCTCTTTTGGAAGGCTTCAATATGTTCATGTTTCTGTTGACACCTGTTCTGGCGTAATGTTTGCCACACCCCTAACAGGGGAAAAGGCCTCATATGTGATTCAACATTGCCTTGAGGCCTGGAGTGCTTGGGGAAAACCCAGAATTTTAAAAACAGACAATGGACCAGCATATACCTCTCAAAAATTCC
GACAGTTTTGTCGGCAAATGGATGTTACCCATCTAACTGGCCTACCTTATAACCCTCAAGGACAGGGCATTGTGGAACGTGCCCATCGTACACTCAAATCATATCTGATTAAACAAAAGGGGAGTATTGAGGATGTTCTACCCTCGGTACCAAGAGTTGCTGTATCCATGGCACTTTTTACCCTAATTTCTTGAACTGTGATGCCCAAGGCCACACTGCGGCTGA
TCGCCACAGTTTAGAGCCTGATAGGCCAAAAGAAATGGTAAAATGGAAAGATGTCTTGACTGATCTCTGGAAAGGCCCGGATCCTATTCTTATAAGATCCAGGGGAGCTGTGTGTGTTTTTCCACAGGAGGAAGAAAACCCCCTGTGGATCCCTGAAAGACTCACTCGAAGAGCTCCTTCACAACTCCAGAATAAGAAGGATGGAGGCCAAGTGGGTGATGAAGAC
TCTCCTACTCGGGATGATGGTTCCTAGTATGATGGGAGAGTTACGGTGGGCCATCATGTCTACCTTCCCCCTACCGATGCCAGTGATGCAGAATGCCCAGGTGTTCCCTAGATTTTTCACAAATAATAGAGAGTTGGGGCTTGCCTTTTTGCCTTTAGATCCTCAGATACAGAGTTTTCAGGATGAGAGAACCTTGTCATTAGTAGGAAGCTTGTGCTTTACTGTG
ATTTCTTCATTAAAGGATAATTTTATTCATATGGATCCAGAAAGATCTAATGCTAATCCTTTTGAATTAATGGAAGCTGTACTGATCTTTCTCCTATGATAGTGAGAAGGGACAGATTACTTTTGATTGGAAAGGGGCATTAGGCAACAGTCTGAGTGAGGGACAGAGCTCTGGATCATCTGATTGGTCCTCTCCAATGTTAAGTATACCCCTTTTAAGAGAACT
AATTATTCAGCTGCCCTTTATTTGGGTTGTGAGTAATGATACCTTAGGTAAGAATCAGAAAGAAATAAATTGTTCTGCTTAGAATTGTTTTTATGCATTATGCTGGGATGCTATTAAGCACCCTTTTGCTTTGGTAATGCGCATGCCTCGATTTATTCCAGCTCCTGTTGAGGCGCCAAGTAATTTGAGTTTGTTTAGGGAAAAGCGAGATTTTTGGGATCTCTGC
TATTATTGTGGGCCTGATAGCCACCGCCGCGGTTACTGCTTCGGTTACCGCCTCGGCCCTTGCACTTTCTGCCACTGTGCAGACAACTCAAACCATTAATGAACTATAGGCCACTGTGACCATGGTCCTAGATAAACAGGCCACTGCCAATTCTCAGATACAAGGAGGCCTGATGTTGGTAAACCAACAGATTAATTTGGTTCAGGAACAATTGGATATATTGTGG
CAGATGGCCAAATTGGGATGTGAACAAAGTTGCCTGGCCTTTGTGTCACTTAGTACAATATGAGAATTTTACAAGAGCAGCTAACCTGTCAAAAGCTCTGTCTCAACATTTATTACAGAATTGGACTTTTGAGTTTGAGCAGACACTCCGTGAGTTGAGAATGGCGATCCTGCAAGTGAATTCGACGCATCTGGATCTTTCTCTGATGGAAGACCTCTCCCCCTGG
ATTTCATCTGCATTTTCTTATTTTAAGGAATGGGTGGGGGTAGGATCATTTAGCCTAGTTCTCTGCTGTGGAATAGTGTTGATGCTTTGGATGGTGTGTACGCTCAGAGCCCAAACAAAAGGAGAAAGTGGTGGTGGCACAAGCACTTGCTGCCTTAGAACACGGAGCATCCCCTGACATTTGGTTAACCATGTTAAAGCAATAGGTCGCTGGCCTCCCAGCTCTT
GCACCCCACGAGGCTAGTCTCATTGCACGGGATAGAGTGGGTAGGCTTCAGCAGCCCAAGGAGTTGCAAGGCTAAGCACTGCACAGGAGAGGTCTGCGGTATAACGACTTTCTCCTGGGAGATAAGTCATCTTGCATGAAGGTTCTATGTCATGATCTCCTTCCCCCAGAAAAAAGACATCGGACTGGTCAGGACTTCCTCTGGGGATAAGACCCTGGAGAGGAGC
TTTGTACATGTTCCGAATTTGCACATGGGGATTTGACCTCTATCTCCACTCCAAAGTTGTGGGTGTCCTATTGCTTCAAAAATAAATGAATAGGGGGAGATGTGAGGAGCCGCCCTCGCTATCGCTATTGCCGTTAGAAGATGGCGCTGACATCCACTGTCAGTTAGAGTTAACTGTCGCTGTCAGTTGGAGTTAACCGTTTAAACTGTGCCTCTCCCGTGACGTC
ATCTGGGGTGATGTGCAAACCACCAATCCCGGCTCTACACATCTCACTCGGAGCTCCTAGGCTTATATAAGGGGCTGGGTTTCTTAGCTTGGGGTCTCCCTCTAAGAAGCTGATCATCTATCTCTCAAGATGCATTAAAGCTTTACTGCAGAAGGATCCGAGTGTTCTGCGTCGTTCTTGCTGGCGAGACGGTAGCGCGGGACA

>IAPLTR3.3654607
TGTGAGGAGCCGCCCTCGCTATCGCTATTGCCGTTAGAAGATGGCGCTGACATCCACTGTCAGTTGGAGTTAACTGTCGCTGTCAGTTGGAGTTAACTGTCGCTGTCAGTTGGAGTTAACCGTTTAAACTGTGCCTCTCCCGTGACGTCATCTGGGGTGATGTGCAAACCACCAATCCCGGCTCTACACATCTCACTCGGAGCTCCTAGGCTTATATAAGGGGCTG
GGTTTCTTAGCTTGGGGTCTCCCTCTAAGAAGCTGATCATCTATCTCTCAAGATGCATTAAAGCTTTACTGCAGAAGGATCCGAGTGTTCTGCGTCGTTCTTGCTGGCGAGACGGTAGCGCGGGACATCTGGTGCCGAAACCCGGGAACTCTTCAACATCGCCGGCGCCGCGGGAGACCCCTCGGAAAACGGGGCGGATTCAGAACTGCAGGGACGTAGGTTCAGA
GAGGTATGCTTTATCCTGAACAACCCTTTTCTCGACTTTGGCCTTAGTTTGTCATCACCGTGGGACGGGTCAGTGGAGGCTTTGGTGCTAGTCCTGTTTGTCCTTCTCCTTCATCGTGGCTGGTGCTGACAGAACGGCGCTGTCCGTCTGGCGCTGTCCGTCTGGCGCTGTCCATCTGGCGCTGTCCATCTGGCGCTGTCCATCTGGCGCTGTCAGTCTGGTGCTG
TCAGTCTGGTGCTGTCAGTCTGGTGCTGTCAGTCTGGTAACGGAGTTGAGCGCGTTTTTAGATAAGCTGTCAGCGCGATGGGGTCGTCACAGTCAGTGGTTACAGCCTTACAGACTGTGCTAAAACAAAGAGATCTTAAAATTGCACCACGAACACTTCAAAATTTTGTGAAGGAGGTAGATAGAGTGGCACCATGGTATGCTTGTTCCGGGTCCCTCACTGTGG
CCTCATGGAACAAGCTAGGTAAAGAACTAGACAGGAAGCATGCAGAAGGAGATCTCTGCTTAGGTACCAAAGCCATTTGGAAGCTAGTTAAAAACTGCTTGGAGGATGAGGCCTGTCACCCTGCTATAATAGAAAGTCAGGGAACCTTAGAGGAAGTTCAGGATAGCATGTCAGAAACTGAACGGAGTGAGAGAATGGGAGCTCGCAAAAGAAAAGACATGTCTAA
GAAAAAAGGCCCTCCCCAGGAAGTTAAGAAGGGGGAGAGAAAGAAGGGAGTTATCATTCACCCCTTAACAAATCTAAGAAAAAGAAAAAGCCAGAATCTAGTCTATACCCTGCAGTAGAGCTAGAAGCCTTGGAGCTAGATAATTCTGATTCTGACACTCTGGATTCTAGCGAAGAAGGTGGTCTAGAGGAGGAGGTGGCACGGTATGAGGAAAAAAGATATCAC
CCTGATAGACACCGGCCACTGAAAACAAAAATGAATGTGAGGCCACCGCCTATAAATCCGGCGGGCTCACGCCCCTCAGCGCCTCCGCCATATGAGCTTCGGCTGAATACTGGTACTGACTCCTTTTTACCTTTAGAGGAACGGAGAAAAATACAAATGGCTTTTCCAGTATTCGAGAATGTGGAAGGCGGAAGAGTACATACCCCCGTAGATTACAATCAAATTA
AAGAATTGGCCGAATCCATTCGTAATTATGGGGTCAATGCTAACTTTACCACATTACAGGTGGAAAGGCTTGCTAATTTTGCCATGACTCCCACTGACTGGCAGACGACAGTGAAAGCAGTGCTTCCCAACATGGGACAATACATGGAATGGAAGGCGCTCTGGTATGATGCAGCCCAGGCACAAGCTAGAGTTAATGCCACGGCTGAAGATGAGAATCAGAGGCA
GTGGACATTTGAAATGCTGACAGGTCAGGGTCAACATGCCCTTAATCAAACCAACTACATCTGGGGCGACTATGGTCAGGTGTCGGCCGCCGCTATTAAAGCATGGAAGGCCCTCACAAAAAGGGGAGAGTCCAGTGGACACCTTACAAAGATTGTCCAAGGACCCCAAGAGCCATTTTCAGACTTTGTGGCCAGAATGACAGAGGCCGCCAGTCGCATCTTTGGT
GATTCAGAACAAGCTATGCCCCTGATTGAGCAACTTGTCTATGAACAAGCAACCCAGGAATGTCGAGCGGCTATCGCCCCTCGCAAAAGCAAGGTTTACAAGATTGGCTCAGAGTGTGCAGGGAGCTGGGGGGACCCCTAACCAATGCAGGACTAGCTGCCGCCATTTTACAAACGCACAGGTATAGAGATTTATCTAATCGAAAAGCTTGTTTTAATTGTGGGA
GAATGGGGCACCTTAAGAAAGATTGTCAAGCCCCTGAAAGAACAAGAGAATCGAAGCTTGGCCATCGTTGTGGTAAAGGTTATCATAGGGCTAGCGAATGCAGGTCAGTCCGGGATGTAAAAGGGAGGCTTCTGCCCCCTATTGAAGAATCCAAGGTACCCCAATCAAAAAACGGGTCACGGGGCCCATGGTCCCAGGCCCCTCAGAAATATGGGAACCAATTTCA
GAAAAGCAATCAAGAGGAAGTAGAGGTTTCCGAGGAACCTCCGGAATGGACTTGCGTGCCGCCTCCGACTTCTTACTCATGCCCCAAATGAATGTTCAACCGGTCCCAGTCCATTCCCCTGGACCTTTGCCCCCTGCCACCATTGGGCTTATTCTGGGCCGTGGCTCCTTAACTTTACAGGGACTCATTATATATCCTGGGATTGTTGACCCATATCATAAAGAGG
AGATTCAAGTTCTCTGTTCTAGCCCTCGGGGTGTATTTTCTATAAAGCAGGGAGATAGAATTGCACAACTGGTGTTGCTGCCCTCCCTCGGGGATGGAGAAACCTACACTTTACAAAAAGGGCTATGGGATCTTCTGGAAGTGATTCAGCCTATCTGGCTATACCCCTAAATGAGAGACCTACAATAAAATTGTTAATTAATGGAAAAGAATTTGAAGGGATAAT
GGATTCGGGAGCAGACAAAAGCATTATTTCACTCCATTGGTGGCCGAAGTCTTGGCCCACTGTTGTTTCATCTCATTCTCTACAAGGTCTTGGATACCAGTCCTCTCCTGCCATCAGTGCCTCAGCCTTAACCTGGCGGGATGCTGAAGGCAAACAGGGATGTTTTACCCCCTATGTGTTGCCACTCCCTGTAAATTTATGGGGACGAGATGTGTTACAAGCCATG
GGCATGACCCTAACTAATGAATACTCCCCCAGGCATCAGCCATTATGGCAAAGATGGGGTATACAAATGGTAGAGGCTTGGGTAGGCAAGAACAAGGCAGAATAAAACCCATTACACAACACGGAAATCGGGGTAGAAAAGGACTGGGTTTTATTTAGGGGCCGTTGAGGCTTCACGACCCATACCATGGAAAACAGAGGAGCCGTTATGGGTCTCTCAATGGCC
TCTATCCTCTGAAAAATTAGAGGCTGTCACAAGATTAGTGCAAGAACAGGAACGGCTGGGGCATCTAGAGCCTTCTACCTCCCCATGGAATACACCAATTTTTGTTATTAAAAAGAAATCTGGGAAGTGGAGATTACTCCACGACCTGCGGGCCATTAACAATCAGATGCATCTTTTTGGCCCTGTTCAAAGAGGCTTCCTTTGCTTTCTGCACTTCCTCAAGAT
TGGAAGCTTATTATTATAGATATTAAGGATTGTTTCTTCTCTATTCCACTTTACCCACGGGATAGACCAAGGTTTGCCTTCACTATCCCTTCTCTTAATCATATGGAACCAGACAAGAGATTTCAGTGGAAGGTACTGCCGCAAGGCATGGCCAATAGCCCAACAATATGTCAGCTATATGTGCAGGAGGCTTTGGAGCCAATTAGGAAGCAATTTACATCTTTAA
TCGTTATCCATTATATGGACGATATTCTATTTGTCACAAAGAGTTAGATGTTTACAAAAGGCTTTTCCCATGCTGGTGGCTGAATTAAAACAGTGGGGATTAGAGATTGCCTCAGAAAAGGTTCAAATTGCAGATACAGGGCTCTTTTTGGGCTCAAAGATTACTCCCAAAAATATAGTTCCTCAGAAAATAGAAATTCGCAAAGACCACTTACAAACATTAAA
TGATTTTCAGAAACTTCTGGGAGATATAAATTGGTTGAGGCCCTTTTTAAAAATTCCATCTGCTGATCTAAAACCCCTCTTCGATCTCCTGGAGGGTGAGCCTCATATCTCCTCACCCAGAAAATTTACCCCTGCCGCCCACCGAGCCTTACAGATGGTGGAAGAGGCCTTACAGGAAGCACAATTACAACGAATTGAGGAGTCACTACCTTTTAATCTGTGTGTT
TTTAAGACAGCTCAATTGCCAACTGCAGTCCTGTGGCAACATGGACCATTACTTTGGATTCATCCAAATGCTTCCCCTGCTAAGATTATTGACTGGTACCCTGATGCTGTTGCACAGCTCGCGCTTCGCGGAATAAAAGCAGCTGTTACACATTTTGGCAGGGATCCTGATTCCTTGATTGTGCCTTATACTGCTGCACAAGTTCAGACCTTGGCAGCCACATCTA
GTGATTGGGCAGTTTTAGTTACTTCCTTTTCAGGACAAATTGATAATCATTTTCAAAGCATCCAATTTTACAGTTTGCCCTAAACCAGGCTATAGTGTTTCCACAAGTGACAGCTAAAGACCCACTCCCAGATGGGACTGTGGTGTACACTGATGGATCAAAAACTGGTATGGGAGCTTATGTGGTCAAGGATAGGGTTATATCTAAACAATATAATGAAACTTC
ACCTCAAGTTGTAGAATGTCTGATAGTACTGGAGGTTCTTGAGGCTTTTCAGGACCCCTCAATATTGTATCAGATTCCTCTTATGTGGTTAATGCAGTTAATCTCCTTGAGATAGCTGGAATAATAAGATCCTCCAGCAGAGTTGCTAACATCTTTCAGAAGATACAAGCTGCCTTGTTAAATAGGAGATTTCCTGTTTTCATTACCCATGTCCGAGCACATTCT
GGACTCCCAGGTCCCATGTCCTTAGGGAATGACTTAGCGGACAAGGCTACAAAGCTGGTGGCCGCTGCCTTGTCTACCCATGCACAAGCAGCAAAAGAATTCCATAAGCGCTTTCATGTGACGGCTGAGACCTTGCGCCGTCGTTTTGCTCTATCCAGGAAAGAGGCTAGGGAAATTGTTACTCAATGCCAGAATTGCTGCGAATTCTTACCTACTCCCCACATGG
GAATAAACCCACGTGGCATTAGACCATTGCAGATGTGGCAGATGGATGTTACTCATATTCCCTCTTTTGGAAGGCTTCAATATGTTCATGTTTCTGTTGACACCTGTTCTGGCGTAATGTTTGCCACACCCCTAACAGGGGAAAAGGCCTCATATGTGATTCAACATTGCCTTGAGGCCTGGAGTGCTTGGGGAAAACCCAGAATTTTAAAAACAGACAATGGACC
AGCATATACCTCTCAAAAATTCCGACAGTTTTGTCGGCAAATGGATGTTACCCATCTAACTGGCCTACCTTATAACCCTCAAGGACAGGGCATTGTGGAACGTGCCCATCGTACACTCAAATCATATCTGATTAAACAAAAGGGGAGTATTGAGGATGTTCTACCCTCGGTACCAAGAGTTGCTGTATCCATGGCACTTTTTACCCTAATTTCTTGAACTGTGAT
GCCCAAGGCCACACTGCGGCTGATCGCCACAGTTTAGAGCCTGATAGGCCAAAAGAAATGGTAAAATGGAAAGATGTCTTGACTGATCTCTGGAAAGGCCCGGATCCTATTCTTATAAGATCCAGGGGAGCTGTGTGTGTTTTTCCACAGGAGGAAGAAAACCCCCTGTGGATCCCTGAAAGACTCACTCGAAGAGCTCCTTCACAACTCCAGAATAAGAAGGATG
GAGGCCAAGTGGGTGATGAAGACTCTCCTACTCGGGATGATGGTTCCTAGTATGATGGGAGAGTTACGGTGGGCCATCATGTCTACCTTCCCCCTACCGATGCCAGTGATGCAGAATGCCCAGGTGTTCCCTAGATTTTTCACAACTAATAGAGAGTTGGGGCTTGCCTTTTTGCCTTTAGATCCTCAGATACAGAGTTTTCAGGATGAGAGAACCTTGTCATTAG
TAGGAAGCTTGTGCTTTACTGTGATTTCTTCATTAAAGGATAATTTTATTCATATGGATCCAGAAAGATCTAATGCTAATCCTTTTGAATTAATGGAAGCTGTACTGATCTTTCTCCTATGATAGTGAGAAGGGACAGATGATACCTTAGGTAAGAATCAGAAAGAAATAAATTGTTCTGCTTAGAATTGTTTTTATGCATTATGCTGGGATGCTATTAAGCACCC
TTTTGCTTTGGTAATGCGCATGCCTCGATTTATTCCAGCTCCTGTTGAGGCGCCAAGTAATTTGAGTTTGTTTAGGGAAAAGCGAGATTTTTGGGATCTCTGCTATTATTGTGGGCCTGATAGCCACCGCCGCGGTTACTGCTTCGGTTACCGCCTCGGCCCTTGCACTTTCTGCCACTGTGCAGACAACTCAAACCATTAATGAACTATAGGCCACTGTGACCAT
GGTCCTAGATAAACAGGCCACTGCCAATTCTCAGATACAAGGAGGCCTGATGTTGGTAAACCAACAGATTAATTTGGTTCAGGAACAATTGGATATATTGTGGCAGATGGCCAAATTGGGATGTGAACAAAGTTGCCTGGCCTTTGTGTCACTTAGTACAATATGAGAATTTTACAAGAGCAGCTAACCTGTCAAAAGCTCTGTCTCAACATTTATTACAGAATTG
GACTTTTGAGTTTGAGCAGACACTCCGTGAGTTGAGAATGGCGATCCTGCAAGTGAATTCGACGCATCTGGATCTTTCTCTGATGGAAGACCTCTCCCCCTGGATTTCATCTGCATTTTCTTATTTTAAGGAATGGGTGGGGGTAGGATCATTTAGCCTAGTTCTCTGCTGTGGAATAGTGTTGATGCTTTGGATGGTGTGTACGCTCAGAGCCCAAACAAAAGGA
GAAAGTGGTGGTGGCACAAGCACTTGCTGCCTTAGAACACGGAGCATCCCCTGACATTTGGTTAACCATGTTAAAGCAATAGGTCGCTGGCCTCCCAGCTCTTGCACCCCACGAGGCTAGTCTCATTGCACGGGATAGAGTGGGTAGGCTTCAGCAGCCCAAGGAGTTGCAAGGCTAAGCACTGCACAGGAGAGGTCTGCGGTATAACGACTTTCTCCTGGGAGAT
AAGTCATCTTGCATGAAGGTTCTATGTCATGATCTCCTTCCCCCAGAAAAAAGACATCGGACTGGTCAGGACTTCCTCTGGGGATAAGACCCTGGAGAGGAGCTTTGTACATGTTCCGAATTTGCACATGGGGATTTGACCTCTATCTCCACTCCAAAGTTGTGGGTGTCCTATTGCTTCAAAAATAAATGAATAGGGGGAGATGTGAGGAGCCGCCCTCGCTATC
GCTATTGCCGTTAGAAGATGGCGCTGACATCCACTGTCAGTTGGAGTTAACTGTCGCTGTCAGTTGGAGTTAACTGTCGCTGTCAGTTGGAGTTAACCGTTTAAACTGTGCCTCTCCCGTGACGTCATCTGGGGTGATGTGCAAACCACCAATCCCGGCTCTACACATCTCACTCGGAGCTCCTAGGCTTATATAAGGGGCTGGGTTTCTTAGCTTGGGGTCTCCC
TCTAAGAAGCTGATCATCTATCTCTCAAGATGCATTAAAGCTTTACTGCAGAAGGATCCGAGTGTTCTGCGTCGTTCTTGCTGGCGAGACGGTAGCGCGGGACA

>IAPLTR3.619164
TGTGAGGAGCCGCCCTCGCTATCGCTATTGCCGTTAGAAGATGGCGCTGACATCCACTGTCAGTTGGAGTTAACTGTCGCTGTCAGTTGGAGTTAACTGTCGCTGTCAGTTGGAGTTAACTGTCGCTGTCAGTTGGAGTTAACCGTTTAAACTGTGCCTCTCCCGTGACGTCATCTGGGGTGATGTGCAAACCACCAATCCCGGCTCTACACATCTCACTCGGAGC
TCCTAGGCTTATATAAGGGGCTGGGTTTCTTAGCTTGGGGTCTCCCTCTAAGAAGCTGATCATCTATCTCTCAAGATGCATTAAAGCTTTACTGCAGAAGGATCCGAGTGTTCTGCGTCGTTCTTGCTGGCGAGACGGTAGCGCGGGACATCTGGTGCCGAAACCCGGGAACTCTTCAACATCGCCGGCGCCGCGGGAGACCCCTCGGAAAACGGGGCGGATTCAG
AACTGCAGGGACGTAGGTTCAGAGAGGTATGCTTTATCCTGAACAACCCTTTTCTCGACTTTGGCCTTAGTTTGTCATCACCGTGGGACGGGTCAGTGGAGGCTTTGGTGCTAGTCCTGTTTGTCCTTCTCCTTCATCGTGGCTGGTGCTGACAGAACGGCGCTGTCCGTCTGGCGCTGTCCATCTGGCGCTGTCCATCTGGCGCTGTCAGTCTGGCGCTGTCAGT
CTGGTGCTGTCAGTCTGGTGCTGTCAGTCTGGTAACGGAGTTGAGCGCGTTTTTAGATAAGCTGTCAGCGCGATGGGGTCGTCACAGTCAGTGGTTACAGCCTTACAGACTGTGCTAAAACAAAGAGATCTTAAAATTGCACCACGAACACTTCAAAATTTTGTGAAGGAGGTAGATAGAGTGGCACCATGGTATGCTTGTTCCGGGTCCCTCACTGTGGCCTCA
TGGAACAAGCTAGGTAAAGAACTAGACAGGAAGCATGCAGAAGGAGATCTCCACTTAGGTACCAAAGCCATTTGGAAGCTAGTTAAAAACTGCTTGGAGGATGAGGCCTGTCACCCTGCTATAATAGAAAGTCAGGGAACCTTAGAGGAAGTTCAGGATAGCATGTCAGAAACTGAACGGAGTGAGAGAATGGGAGCTCGCAAAAGAAAAGACATGTCTAAGAAAA
AAGGCCCTCCCCAGGAAGTTAAGAAGGGGGAGAGAAAGAAGGGAGTTATCATTCACCCCTTAACAAATCTAAGAAAAAGAAAAAGCCAGAATCTAGTCTATACCCTGCAGTAGAGCTAGAAGCCTTGGAGCTAGATAATTCTGATTCTGACACTCTGGATTCTAGCGAAGAAGGTGGTCTAGAGGGGGAGGTGGCACGGTATGAGGAAAAAAGATATCACCCTGA
TAGACACCGGCCACTGAAAACAAAAATGAATGTGAGGCCACCGCCTATAAATCCGGCGGGCTCACGCCCCTCAGCGCCTCCGCCATATGAGCTTCGGCTGAATACTGGTACTGACTCCTTTTTACCTTTAGAGGAACGGAGAAAAATACAAATGGCTTTTCCAGTATTCGAGAATGTGGAAGGCGGAAGAGTACATACCCCCGTAGATTACAATCAAATTAAAGAA
TTGGCCGAATCCGTTCGTAATTATGGGGTCAATGCTAACTTTACCACATTACAGGTGGAAAGGCTTGCTAATTTTGCCATGACTCCCACTGACTGGCAAACGACAGTGAAAGCAGTGCTTCCCAACATGGGACAATACATGGAATGGAAGGCGCTCTGGTATGATGCAGCCCAGGCACAAGCTAGAGTTAATGCCACGGCTGAAGATGAGAATCAGAGGCAGTGGA
CATTTGAAATGCTGACAGGTCAGGGTCAACATGCCCTTAATCAAACCAACTACATCTGGGGCGTCTATGGTCAGGTGTCGGCCGCCGCTATTAAAGCATGGAAGGCCCTCACAAAAAGGGGAGAGTCCAGTGGACACCTTACAAAGATTGTCCAAGGACCCCAAGAGCCATTTTCAGACTTTGTGGCCAGAATGACAGAGGCCGCCAGTCGCATCTTTGGTGATTC
AGAACAAGCTATGCCCCTGATTGAGCAACTTGTCTATGAACAAGCAACCCAGGAATGTCGAGCGGCTATCGCCCCTCGAAAAAGCAAGGTTTACAAGATTGGCTCAGAGTGTGCAGGGAGCTGGGGGGACCCCTAACCAATGCAGGACTAGCTGCCGCCATTTTACAAACGCACAGGTATAGAGATTTATCTAATCGAAAAGCTTGTTTTAATTGTGGGAGAATG
GGGCACCTTAAGAAAGATTGTCAAGCCCCTGAAAGAACAAGAGAATCGAAGCTTTGCTATCGTTGTGGTAAAGGTTATCATAGGGCTAGCGAATGCAGGTCAGTCCGGGATGTAAAAGGGAGGCTTCTGCCCCCTATTGAAGAATCCAAGGTACCCCAATCAAAAAACGGGTCACGGGGCCCATGGTCCCAGGCCCCTCAGAAATATGGGAACCAATTTCAGAAAA
GCAATCAAGAGGAAGTAGAGGTTTCCGAGGAACCTCCGGAATGGACTTGCGTGCCGCCTCCGACTTCTTACTCATGCCCCAAATGAATGTTCAACCGGTCCCAGTCCATTCCCCTGGACCTTTGCCCCCTGCCACCATTGGGCTTATTCTGGGCCGTGGCTCCTTAACTTTACAGGGACTCATTATATATCCTGGGATTGTTGACCCATATCATAAAGAGGAGATT
CAAGTTCTCTGTTCTAGCCCTCGGGGTGTATTTTCTATAAAGCAGGGAGATAGAATTGCACAACTGGTGTTGCTGCCCTCCCTCGGGGATGGAGAAACCTACACTTTACAAAAAGGGCTATGGGATCTTCTGGAAGTGATTCAGCCTATCTGGCTATACCCCTAAATGAGAGACCTACAATAAAATTGTTAATTAATGGAAAAGAATTTGAAGGGATAATGGATT
CGGGAGCAGACAAAAGCATTATTTCACTCCATTGGTGGCCGAAGTCTTGGCCCACTGTTGTTTCATCTCATTCTCTACAAGGTCTTGGATACCAGTCCTCTCCTGCCATCAGTGCCTCAGCCTTAACCTGGCGGGATGCTGAAGGCAAACAGGGATGTTTTACCCCCTATGTGTTGCCACTCCCTGTAAATTTATGGGAACGAGATGTGTTACAAGCCATGGGCAT
GACCCTAACTAATGAATACTCCCCCAGGCATCAGCCATTATGGCAAAGATGGGGTATACAAATGGTAGAGGCTTGGGTAGGCAAGAACAAGGCAGAATAAAACCCATTACACAACACGGAAATCGGGGTAGAAAAGGACTGGGTTTTATTTAGGGGCCGTTGAGGCTTCACGACCCATACCATGGAAAACAGAGGAGCCGTTATGGGTCTCTCAATGGCCTCTAT
CCTCTGAAAAATTAGAGGCTGTCACAAGATTAGTGCAAGAACAGGAACGGCTGGGGCATCTAGAGCCTTCTACCTCCCCATGGAATACACCAATTTTTGTTATTAAGAAGAAATCTGGGAAGTGGAGATTACTCCACGACCTGCGGGCCATTAACAATCAGATGCATCTTTTTGGCCCTGTTCAAAGAGGCCTTCCTTTGCTTTCTGCACTTCCTCAAGATTGGAA
GCTTATTATTATAGATATTAAGGATTGTTTCTTCTCTATTCCACTTTACCCACGGGATAGACCAAGGTTTGCCTTCACTATCCCTTCTCTTAATCATATGGAACCAGACAAGAGATTTCAGTGGAAGGTACTGCCGCAAGGCATGGCCAATAGCCCAACAATATGTCAGCTATATGTGCAGGAGGCTTTGGAGCCAATTAGGAAGCAATTTACATCTTTAATCGTT
ATCCATTATATGGACGATATTCTATTTGTCACAAAGAGTTAGATGTTTACAAAAGGCTTTTCCCATGCTGGTGGCTGAATTAAAACAGTGGGGATTAGAGATTGCCTCAGAAAAGGTTCAAATTGCAGATACAGGCCTCTTTTTGGGCTCAATGATTACTCCCAAAAATATAGTTCCTCAGAAAATAGAAATTCGCAAAGACCACTTACAAACATTAAATGATT
TTCAGAAACTTCTGGGAGATATAAATTGGTTGAGGCCCTTTTTAAAAATTCCATCTGCTGATCTAAAACCCCTCTTCGATCTCCTGGAGGGTGAGCCTCATATCTCCTCACCCAGAAAATTTACCCCTGCCGCCCACCGAGCCTTACAGATGGTGGAAGAGGCCTTACAGGAAGCACAATTACAACGAATTGAGGAGTCACTACCTTTTAATCTGTGTGTTTTTAA
GACAGCTCAATTGCCAACTGCAGTCCTGTGGCAACATGGACCATTACTTTGGATTCATCCAAATGCTTCCCCTGCTAAGATTATTGACTGGTACCCTGATGCTGTTGCACAGCTCGCGCTTCGCGGAATAAAAGCAGCTGTTACACATTTTGGCAGGGATCCTGATTCCTTGATTGTGCCTTATACTGCTGCACAAGTTCAGACCTTGGCAGCCACATCTAGTGAT
TGGGCAGTTTTAGTTACTTCCTTTTCAGGACAAATTGATAATCATTTTCAAAGCATCCAATTTTACAGTTTGCCCTAAACCAGGCTATAGTGTTTCCACAAGTGACAGCTAAAGACCCACTCCCAGATGGGACTGTGGTGTACACTGATGGATCAAAAACTGGTATGGGAGCTTATGTGGTCAAGGATAGGGTTATATCTAAACAATATAATGAAACTTCACCTC
AAGTTGTAGAATGTCTGATAGTACTGGAGGTTCTTGAGGCTTTTCAGGACCCCTCAATATTGTATCAGATTCCTCTTATGTGGTTAATGCAGTTAATCTCCTTGAGATAGCTGGAATAATAAGATCCTCCAGCAGAGTTGCTAACATCTTTCAGAAGATACAAGCTGCCTTGTTAAATAGGAGATTTCCTGTTTTCATTACCCATGTCCGAGCACATTCTGGACT
CCCAGGTCCCATGTCCTTAGGGAATGACTTAGCGGACAAGGCTACAAAGCTGGTGGCCGCTGCCTTGTCTACCCATGCACAAGCAGCAAAAGAATTCCATAAGCGCTTTCATGTGACGGCTGAGACCTTGCGCCGTCGTTTTGCTCTATCCAGGAAAGAGGCTAGGGAAATTGTTACTCAATGCCAGAATTGCTGCGAATTCTTACCTACTCCCCACATGGGAATA
AACCCACGTGGCATTAGACCATTGCAGATGTGGCAGATGGATGTTACTCATATTCCCTCTTTTGGAAGGCTTCAATATGTTCATGTTTCTGTTGACACCTGTTCTGGCGTAATGTTTGCCACACCCCTAACAGGGGAAAAGGCCTCATATGTGATTCAACATTGCCTTGAGGCCTGGAGTGCTTGGGGAAAACCCAGAATTTTAAAAACAGACAATGGACCAGCAT
ATACCTCTCAAAAATTCCGACAGTTTTGTCGGCAAATGGATGTTACCCATCTAACTGGCCTACCTTATAACCCTCAAGGACAGGGCATTGTGGAACGTGCCCATCGTACACTCAAATCATATCTGATTAAACAAAAGGGGAGTATTGAGGATGTTCTACCCTCGGTACCAAGAGTTGCTGTATCCATGGCACTTTTTACCCTAATTTCTTGAACTGTGATGCCCA
AGGCCACACTGCGGCTGATCGCCACAGTTTAGAGCCTGATAGGCCAAAAGAAATGGTAAAATGGAAAGATGTCTTGACTGATCTCTGGAAAGGCCCGGATCCTATTCTTATAAGATCCAGGGGAGCTGTGTGTGTTTTTCCACAGGAGGAAGAAAACCCCCTGTGGATCCCTGAAAGACTCACTCGAAGAGCTCCTTCACAACTCCAGAATAAGAAGGATGGAGGC
CAAGTGGGTGATGAAGACTCTCCTACTCGGGATGATGGTTCCTAGTATGATGGGAGAGTTACGGTGGGCCATCATGTCTACCTTCCCCCTACCGATGCCAGTGATGCAGAATGCCCAGGTGTTCCCTAGATTTTTCACAAATAATAGAGAGTTGGGGCTTGCCTTTTTGCCTTTAGATCCTCAGATACAGAGTTTTCAGGATGAGAGAACCTTGTCATTAGTAGGA
AGCTTGTGCTTTACTGTGATTTCTTCATTAAAGGATAATTTTATTCATATGGATCCAGAAAGATCTAATGCTAATCCTTTTGAATTAATGGAAGCTGTACTGATCTTTCTCCTATGATAGTGAGAAGGGACAGATTACTTTTGATTGGAAAGGGGCATTAGGCAACAGTCTGAGTGAGGGACAGAGCTCTGGATCATCTGATTGGTCCTCTCCAATGTTAAGTAT
ACCCCTTTTAAGAGAACTAATTATTCAGCTGCCCTTTATTTGGGTTGTGAGTAATGATACCTTAGGTAAGAATCAGAAAGAAATAAATTGTTCTGCTTAGAATTGTTTTTATGCATTATGCTGGGATGCTATTAAGCACCCTTTTGCTTTGGTAATGCGCATGCCTCGATTTATTCCAGCTCCTGTTGAGGCGCCAAGTAATTTGAGTTTGTTTAGGGAAAAGCGA
GATTTTTGGGATCTCTGCTATTATTGTGGGCCTGATAGCCACCGCCGCGGTTACTGCTTCGGTTACCGCCTCGGCCCTTGCACTTTCTGCCACTGTGCAGACAACTCAAACCATTAATGAACTATAGGCCACTGTGACCATGGTCCTAGATAAACAGGCCACTGCCAATTCTCAGATACAAGGAGGCCTGATGTTGGTAAACCAACAGATTAATTTGGTTCAGGAA
CAATTGGATATATTGTGGCAGATGGCCAAATTGGGATGTGAACAAAGTTGCCTGGCCTTTGTGTCACTTAGTACAATATGAGAATTTTACAAGAGCAGCTAACCTGTCAAAAGCTCTGTCTCAACATTTATTACAGAATTGGACTTTTGAGTTTGAGCAGACACTCCGTGAGTTGAGAATGGCGATCCTGCAAGTGAATTCGACGCATCTGGATCTTTCTCTGATG
GAAGACCTCTCCCCCTGGATTTCATCTGCATTTTCTTATTTTAAGGAATGGGTGGGGGTAGGATCATTTAGCCTAGTTCTCTGCTGTGGAATAGTGTTGATGCTTTGGATGGTGTGTACGCTCAGAGCCCAAACAAAAGGAGAAAGTGGTGGTGGCACAAGCACTTGCTGCCTTAGAACACGGAGCATCCCCTGACATTTGGTTAACCATGTTAAAGCAATAGGTC
GCTGGCCTCCCAGCTCTTGCACCCCACGAGGCTAGTCTCATTGCACGGGATAGAGTGGGTAGGCTTCAGCAGCCCAAGGAGTTGCAAGGCTAAGCACTGCACAGGAGAGGTCTGCGGTATAACGACTTTCTCCTGGGAGATAAGTCATCTTGCATGAAGGTTCTATGTCATGATCTCCTTCCCCCAGAAAAAAGACATCGGACTGGTCAGGACTTCCTCTGGGGAT
AAGACCCTGGAGAGGAGCTTTGTACATGTTCCGAATTTGCACATGGGGATTTGACCTCTATCTCCACTCCAAAGTTGTGGGTGTCCTATTGCTTCAAAAATAAATGAATAGGGGGAGATGTGAGGAGCCGCCCTCGCTATCGCTATTGCCGTTAGAAGATGGCGCTGACATCCACTGTCAGTTGGAGTTAACTGTCGCTGTCAGTTGGAGTTAACTGTCGCTGTCA
GTTGGAGTTAACTGTCGCTGTCAGTTGGAGTTAACCGTTTAAACTGTGCCTCTCCCGTGACGTCATCTGGGGTGATGTGCAAACCACCAATCCCGGCTCTACACATCTCACTCGGAGCTCCTAGGCTTATATAAGGGGCTGGGTTTCTTAGCTTGGGGTCTCCCTCTAAGAAGCTGATCATCTATCTCTCAAGATGCATTAAAGCTTTACTGCAGAAGGATCCGAG
TGTTCTGCGTCGTTCTTGCTGGCGAGACGGTAGCGCGGGACA

>IAPLTR3.3848539
TGTGAGGAGCCGCCCTCGCTATCGCTATTGCCGTTAGAAGATGGCGCTGACATCTGCTGTCAGTTGGAGTTAACCGTTTAAGCTGTGCCTCTCCGTGGCGTCATCTGGGGTGATGTGCAAACCACCAATCCCGGCTCTACACGTCTCACTCGGAGCTCCTAGGCTTATATAAGGGGCTGGGTTTCTTAGCTTGGGGTCTCCCTCTAAGAAGCTGATCATCTATCTC
TCAAGATGCATTAAAGCTTTACTGCAGAAGGATCCGAGTGTTCTGCGTCGTTCTTGCTGGCGAGACGGTAGCGCGGGACATCTGGTGCCGAAACCCGGGAACTCTTCAACATCGCCGGCGCCGCGGGAGACCCCTCGGAAGACGGGGCGGATTCAGAACTGCAGGGACGTAAGTTCAGAGAGGTATGCTTTATCCTGAACACCCCCTTTCTCGACTTTGGCCTTAG
TTTGTCATCACCGTGGGACGGGCTCAGTGGAGGCTTTGGTGCTAGTCCTGTTTGTCCTTCTCCTTCATCGTGGCTGGTGCTGACAGAACGGTGCTGTCAGTCTGGTGCTGTCAGTCTGGTGCTGTCAGTCTGGTGCTGTCAGTCTGGTGCTGTCAGTCTGGTAACGGAGTTGAGCGCCGTTTTTTAGATAAGCCGCCAGCGCGATGGGGTTGTCACAGTCAGTGGTT
ACTGCCCTACAGACTGTGCTAAAACAAAGAGATCTTAAAATTGCACCACGAACACTTCAAAATTTTGTGAAGGAGGTAGATAGAGTGGCACCATGGTATGCTAGTTCCGGGTCCCTCACTGTGGCCTCATGGAACAAGCTAGGTAAAGAACTAGACAGGAAGCATGCAGAAGGAGATCTCCACTTAGGTACCAAAGCCATTTGGAAGCTAGTTAAAAACTGCTTGG
AGGATGAGGCCTGTCACCCTGCTATAATAGAAAGTCAGGGAACCTTAGAGGAAGTTCAGGATAGCATGTCAGAAACTGAACGGAGTGAGAGAATGGGAGCTCGCAAAAGGAAAGACATGTCTAAGAAAAAAAGGCCCTCCCCAAGAAGTTAAGAAGGGGGAGAGAGAAGGGAGTGATCATTCACCCCCTAACAAATCTAAGAAAAAGAAAAAGCCAGAAGCTAG
TCTATACCCTACAGTAGAGCTAGAAGCCTTGGAGCTAGATAATTCTGATTCTGACACTCTGGATTCTAGCGAAGAAGGTGGTCTAGAGGAGGAGGTGGCACGGTATGAGGAAGAAAGATATCACCCTGATAGACACCGGCCACTGAAAACAAAAATGAATGTGAGGCCACCGCCTATAAATCCGGCGGGCTCACGCCCCTCAGCGCCTCCGCAGTATGAGCTTCGG
CTGAAAACTGGTACTGACTCCTTTTTACCTTTAGAGGAACGGAGAAAAATACAAATGGCTTTTCCAGTATTCGAGAATGCGGAAGGCGGAAGAGTACATACCCCTGTAGATTACAATCAAATCAAAGAATTGGCCGAATCCATTCGTAATTATGGGGTCAATGCTAACTTTACCACATTACAGGTGGAAAGGCTTGCTAATTTTGCCATGACTCCCACTGACTGGC
AGACGACAGTGAAAGCAGTGCTTCCCAACATGGGACAATACATGGAATGGAAGGCTCTCTGGTATGATGCAGCCCAGGCACAAGCTAGAGTTAATGCCACGGCTGAAGATGAGAATCAGAGGCAGTGGACATTTGAAATGCTGACAGGTCAGGGTCAACATGCCCTTAATCAAACCAACTACATCTGGGGTGTCTATGGTCAGGTGTCGGCCGCCGCTATTAAAGC
ATGGAAGGCCCTCACAAAAAGGGGAGAGTGCAGTGGACACCTTACAAAGATTGTCCAAGGACCCCAAGAGCCATTTTCAGACTTTGTGGCCAGAATGACAGAGGCCGCCAGTCGCATCTTTGGTGATTCAGAACAAGCTATGCCCCTGATTGAGCAACTTGTCTATGAACAAGCAACCCAGGAATGTCGAGCGGCTATCGCCCCTCGCAAAAGCAAGGTTTACAA
GATTGGCTCAGAGTGTGCAGGGAGCTGGGGGGACCCCTAACCAATGCAGGACTAGCTGCCGCCATTTTACAAACGCACAGGTATAGAGATTTATCTAATCGAAAAGCTTGTTTTAATTGTGGGAGAATGGGGCACCTTAAGAAAGATTGTCAAGCCCCTGAAAGAACAAGAGAATCGAAGCTTTGCCATCGTTGTGGTAAAGGTTATCATAGGGCTAGCGAATGCA
GGTCAGTCCGGGATGTAAAAGGGAGGCTTCTGCCCCCTATTGAAGAACCCAAGGTACCCCAATCAAAAAACGGGTCACGGGGCCCATGGTCCCAGGCCCCTCAGAAATATGGGAACCAATTTCAGAAAAGCAATCAAGAGGAAGTAGAGGCTTCCGAGGAACCCCCGGAATGGACTTGCGTGCCGCCTCCGACTTCTTACTCATGCCCCAAATGAATGTTCAACCG
GTCCCAGTCCATTCCCCTGGACCTTTGCCCCCTGCCACCATTGGGCTTATTCTGGGCCGTGGCTCCTTAACTTTACAGGGACTCATTATATATCCTGGAATTGTTGACCCGTATCATAAAGAGGAGATTCAAGTTCTCTGTTCTAGCCCTCGGGGTGTATTTTCTATAAAGCAGGGAGATAGAATTGCACAACTGGTGTTGCTGCCCTCCCTCGGGGATGGAGAAA
CCTACACTTTACAAAAAGGGCTATGGGATCTTCTGGAAGTGATTCAGCCTATCTGGCTATACCCCTAAATGAGAGACCTACAATAAAATGGTTAATTAATGGAAAAGAATTTGAAGGGATAATGGATACGGGAGCAGACAAAAGCATTATTTCACTCCATTGGTGGCCGAAGTCTTGGCCCACTGTTGTTTCATCTCATTCTCTACAAGGTCTTGGATACCAGTC
CTCTCCTGCCATCAGTGCCTCAGCCTTAACCTGGCGGGATGCTGAGGGCAAACAGGGATGTTTTACCCCCTATGTGTTGCCACTCCCTGTAAATTTATGGGGACGAGATGTGTTACAAGCCATGGGCATGACCCTAACTAATGAATACTCCCCCAGGCATCAGCCATTATGGCAAAGATGGGGTATACAAACGGTAGAGGCTTGGGTAGGCAAGAACAAGGCAGA
ATAAAACCTATTACGCAACACGGAAATCGGGGTAGAAAAGGACTGGGTTTTATTTAGGGGCCGTTGAGGCTTCACGACCCATACCATGGAAAACAGAGGAGCCGTTATGGGTCTCTCAATGGCCTCTATCCTCTGAAAAATTAGAGGCTGTCACAAGATTAGTGCAAGAACAGGAACGGCTGGGGCATCTAGAGCCTTCTACCTCCCCATGGAATACACCAATTTT
TGTTATTAAGAAGAAATCTGGGAAGTGGAGATTACTCCACGACCTGCGGGCCATTAACAATCAGATGCATCTTTTTGGCCCTGTTCAAAGAGGCCTTCCTTTGCTTTCTGCACTTCCTCAAGATTGGAAGCTTATTATTATAGATATTAAGGATTGTTTCTTCTCTATTCCACTTTACCCACGGGATAGACCAAGGTTTGCCTTCACTATCCCTTCTCTTAATCAT
ATGGAACCAGACAAGAGATTTCAGTGGAAGGTACTGCCGCAAGGCATGGCCAATAGCCCAACAATATGTCAGCTGTATGTGCAGGAGGCTTTGGAGCCAATTAGGAAGCAATTTACATCTTTAATCGTTATCCATTATATGGACGATATTCTTATTTGTCACAAAGAGTTAGATGTTTTACAAAAGGCTTTCCCCATGCTGGTGGCTGAATTAAAACAGTGGGGAT
TAGAGATTGCCTCAGAAAAGGTTCAAATTGCAGATACAGGCCTCTTTTTGGGCTCAATGATTACTCCCAAAAATATAGTTCCTCAGAAAATAGAAATTCGCAAAGACCACTTACAAACATTAAATGATTTTCAGAAACTTCTGGGAGATATAAATTGGTTGAGGCCCTTTTTAAAAATTCCATCTGCTGATCTAAAACCCCTCTTCGATCTCCTGGAGGGTGAGCC
TCATATCTCCTCACCCAGAAAATTTACCCCTGCCGCCCACCGAGCCTTACAGATGGTGGAAGAGGCCTTACAGGAAGCACAATTACAACGTATTGAGGAGTCACTACCTTTTAATCTGTGTGTTTTTAAGACAGCTCAATTGCCAACTGCAGTCCTGTGGCAACATGGACCATTACTTTGGATTCATCCAAATGCTTCCCCTGCTAAGATTATTGACTGGTACCCT
GATGCTGTTGCACAGCTCGCGGTTCGCAGAATAAAAGCAGCTGTTACACATTTTGGCAGGGATCCTGATTCCTTGATTGTGCCTTATACTGCTGCACAAGTTCAGACCTTGGCAGCCACATCTAGTGATTGGGCAGTTTTAGTTACTTCCTTTTCAGGACAAATTGATAATCATTTTCAAAGCATCCAATTTTACAGTTTGCCCTAAACCAGGCTATAGTGTTTC
CACAAGTGACAGCTAAAGACCCACTCCCAGATGGGACTGTGGTGTACACTGATGGATCAAAAACTGGTATGGGAGCTTATGTGGTCAAGGATAGGGTTATATCTAAACAATATAAGGAACTTCACCTCAAGTTGTAGAATGTCTGATAGTACTGGAGGTTCTTGAGGCTTTTCCAGGACCCCTCAATATTGTATCAGATTCCTCTTATGTGGTTAATGCAGTTAA
TCTCCTTGAGATAGCTGGAATAATAAGATCCTCCAGCAGAGTTGCTAACATCTTTCAAAAGATACAAGCTTCCTTGTTAAATAGGAGATTTCCTGTTTTCATTACCCATGTCCGAGCACATTCTGGACTCCCAGGTCCCATGTCCTTAGGGAATGACTTAGCGGACAATGCTACAAAGCTGGTGGCCGCTGCCTTGTCTACCCATGCACAAGCAGTAAAAGAATTC
CATAAGCGCTTTCATGTGACGGCTGAGACCTTGCGCCGTCGTTTTGCTCTATCCAGGAAAGAGGCTAGGGAAATTGTTACTCAATGCCAGAATTGCTGCGAATTCTTACCTACTCCCCACATGGGAATAAACCCACGTGGCATTAGACCATTGCAGATGTGGCAGATGGATGTTACTCATATTCCCTCTTTTGGAGGCTTCAATATGTTCATGTTTCTGTTGACA
CCTGTTCTGGCGTAATGTTCGCCACACCCCTAACAGGGGAAAAGGCCTCATATGTGATTCAACATTGCCTTGAGGCCTGGAGTGCTTGGGGAAAACCCAGAATTTTAAAAACAGACAATGGACCAGCATATACCTCTCAAAAATTCCGACAGTTTTGTCGGCAAATGGATGTTACCCATCTAACTGGCCTACCTTATAACCCTCAAGGACAGGGCATTGTGGAACG
TGCCCATCGTACACTCAAATCATATCTGATTAAACAAAAGGGGAGTATTGAGGATGTTCTACCCTCGGTACCAAGAGTTGCTGTATCCATGGCACTTTTTACCCTAATTTCTTGAACTCTGATGCCCAAGGCCACACTGCGGCCGATCGCCACAGTTTAGAGCCTGATAGGCCAAAAGAAATGGTAAAATGGAAAGATGTCTTGACTGATCTCTGGAAAGGCCCG
GATCCTATTCTTATAAGATCCAGGGGAGCTGTGTGTGTTTTTCCACAGGAGGAAGAAAATCCCCTGTGGATCCCTGAAAGACTCACTCGAAGAGCTCCTTCACAACTCCAGAATAAGAAGGATGGAGGCCAAGTGGGTGATGAAGACTCTCCTACTCGGGATGATGGTTCCTAGTATGATGGGAGAGTTACGGTGGGCCATCATGTCTACCTTCCCCCTACCGATG
CCAGTGATGCATAATGCCCAGGTGTTCCCTAGATTTTTCACAACTAATAGAGAGTTGGGGCTTGCCTTTTTGCCTTTAGATCCTCAGATACAGAGTTTTCAGGATGAGAGAACCTTGTCATTAGTAGGAAGCTTGTGCTTTACTGTGATTTCTTCATTAAAGGATAATTTTATTCATATGGATCCAGAAAGATCTAATGCTAATCCTTTTGAATTAATGGAAGCTG
TACTGATCTTTCTCCTATGATAGTGAGAAGGGACAGATTACTTTTGATTGGAAAGGGGCATTAGGCAACAGTCTGAGTGAGGGACAGAGCTATGGATCATCTGATTGGTCCTCTCCAATGTTAAGTATACCCCTTTTAAGAGAACTAATTATTCAGCTGCCCTTTATTTGGGTTGTGAGTAATGATACCTTAGGTAAGAATCAGAAAGAAATAAATTGCTCTGCT
TAGAATTGTTTTTATGCATTATGCTGGGATGCTATTAAGCACCCTTTTGCTTTGGTAATGCGCATGCCTCGATTTGTTCCAGTTCCTGTTGAGACGCCAAGTAATTTGAGTTTGTTTAGGGAAAAGCGAGATTTTTGGGATCTCTGCTATTATTGTGGGCCTGATAGCCACCGCCGCGGTTACTGCTTCGGTTACCGCCTCGGCCCTTGCACTTTCTGCCACTGTG
CAGACAACTCAAACCATTAATGAACTATAGGCCACTGTGACCATGGTCCTAGATAAACAGGCCACTGCCAATTCTCAGATACAAGGAGGCCTGATGTTGGTAAACCAACAGATTAATTTGGTTCAGGAACAATTGGATATATTGTGGCAGATGGCCCAATTGGGATGTGAACAAAGTTGCCTGGCCTTTGTGTCACTTCAGTACAATATGAGAATTTTACAAGAG
CAGCTAACCTGTCACAAGCTCTGTCTCAACATTTATTACAGAATTGGACTTTTGAGTTTGAGCAGACACTCCGTGAGTTGAGAGTGGCGATCCTGCAAGTGAATTCGACGCGTCTGGATCTTTCTCTGATGGAAGACCTCTCCTCCTGGATTTCATCTGCATTTTCTTATTTTAAGGAATGGGTGGGGGTAGGATCATTTAGCCTAGTTCTCTGCTGTGGAATAGT
GTTGATGCTTTGGATGGTGTGTAAGCTCAGAGCCCAAACAAAAGAGACAAAGTGGTGGTGGCACAAGCACTTGCTGCCTTAGAACACGGAGCCTCCCCTGACATTTGGTTAACCATGTTAAAGCAATAGGTCGCTGGCCTCCCAGCTCTTGCACCCCACGAGGCTAGTCTCATTGCATGGGATAGAGTGGGTAGGCTTCAGCAGCCCAAGGAGTTGCAAGGCTAAG
CACTGCACAGGAGAGGTCTGCGGTATAACGACTTTCTCCTGGGAGATAAGTCATCTTGCATGAAGGTTCTATGTCATGATCTCCTTCCCCCAGAAAAACGACATCGGACTGGTCATGGCTCCCTCTGGGGATAAGACCCTGGAGAGGAGCTTTGTACATGTTCCAAATTTGCACATGGGGATTTGACCTCTATCTCCACTCCAAAGTTGTGGGTGTCCTATTGCTT
CAAAAATAAATGAATAGGGGGAGATGTGAGGAGCCGCCCTCGCTATCGCTATTGCCGTTAGAAGATGGCGCTGACATCTGCTGTCAGTTGGAGTTAACCGTTTAAGCTGTGCCTCTCCCGTGGCGTCATCTGGGGTGATGTGCAAACCACCAATCCCGGCTCTACACGTCTCACTCGGAGCTCCTAGGCTTATATAAGGGGCTGGGTTTCTTAGCTTGGGGTCTCC
CTCTAAGAAGCTGATCATCTATCTCTCAAGATGCATTAAAGCTTTACTGCAGAAGGATCCGAGTGTTCTGAGTCGTTCTTGCTGGCGAGACGGTAGCGCGGGACA

>IAPLTR3.1908891
TGTGAGGAGCCGCCCTCGCTATCGCTATTGCCGTTAGAAGATGGCGCTGACATCTGCTGTCAGTTGGAGTTAACCGTTAGAAGATGGCGCTGACATCTGCTGTCAGTTGGAGTTAACCGTTTAAGCTGTGCCTCTCCCGTGGCGTCATCTGGGGTGATGTGCAAACCACCAATCCTGGCTCTACACGTCTCACTCGGAACTCCTAGGCTTATATAAGGGGCTGGGT
TTCTTAGCTTGGGGTCTCCCTCTAAGAAGCTGATCATCTATCTCTCAAGATGCATTAAAGCTTTACTGCAGAAGGATCCGAGTGTTCTGCGTCGTTCTTGCTGGCGAGACGGTAGCGCGGGACATCTGGTGCCGAAACCTGGGAACTCTTCAACATCACCGGCGCCGCGGGAGACCCCTCGGAAGACGGGGCGGATTCAGAACTGCAGGGACGTAAGTTCAGAGAG
GTATGCTTTATCCTGAATACCCCCTTTCTCGACTTTGGCCTTAGTTTGTCATCACCGTGGGACGGGTCAGTGGAGGCTTTGGTGCTAGTCCTGTTTGTCCTTCTCCTTCATCGTGGCTGGTGCTGACAGAACGGTGCTGTCAGTCTGGTGCTGTCAGTCTGGTGCTGTCAGTCTGGTGCTGTCAGTCTGGTGCTGTCAGTCTGGTAACGGAGTTGAGCGCCGTTTT
TTAGATAAGCCGCTAGCGCGATGGGGTTGTCACAGTCAGTGGTTACTGCCCTACAGACTGTGCCAAAACAAAGAGATCTTAAAATTGCACCACGAACACTTCAAAATTTTGTGAAGGAGGTAGATAGAGTGGCACCATGGTATGCTTGTTCCGGGTCCCTCACTGTGGCCTCATGGAACAAGCTAGGTAAAGAACTAGACAGGAAGCATGCAGAAGGAGATCTCCA
CTTAGGTACCAAAGCCATTTGGAAGCTAGTTAAAAACTGCTTGGAGGATGAGGCCTGTCACCCTGCTATAATAGAAAGTCAGGGAACCTTAGAGGAAGTTCAGGATAGCATGTCAGAAACTGAACGGAGTGAGAGAATGGGAGCTCGCAAAAGGAAAGACATGTCTAAGAAAAAAGGCCCTCCCCAAGAGTTAAGAAGGGGGAGAGAAAGAAGGGAGTGATCAT
TCACCCCCTAACAAATCTAAGAAAAAGAAAAAGCCAGAATCTAGTCTATACCCTACAGTAGATCTAGAAGCCTTGGAGCTAGATAATTCTGATTCTGACACTCTGTATTCTAGCGAAGAAGGTGGTCTAGAGGAGGAGGTGGCACGGTATGAGGAAGAAAGATATCACCCTGATAGACACCGGCCACTGAAAACAAAAATGAATGTGAGGCCACCGCCTATAAATC
CGGCGGGCTCACGCCCCTCGGCGCCTCCGCAGTATGAGCTTCGGCTGAATACTGGTACTGACTCCTTTTTACCTTTAGAGGAACGGAGAAAAATACAAGTAGCTTTTCCAGTATTTGAGAATGTGGAAGGCGGAAGAGTACATACTCCCGTAGATTACAATCAAATCAAAGAATTGGCCGAATCCGTTCGTAATTATGGGTCAATGCTAACTTTACCACATTACA
GGTGGAAAGGCTTGCTAATTTTGCCATGACTCCCACTGACTGGCAGACGACAGTGAAAGCAGTGCTTCCCAACATGGGACAATACATGGAATGGAAGGCTCTCTGGTATGATGCAGCCCAGGCGCAAGCTAGAGTTAATGCCACAGCTGAAGATGAGAATCAGAGGCAGTGGACATTTGAAATGCTGACAGGTCAGGGTCAACATGCCCTTAATCAAACCAACTAC
ATCTGGGGTGTCTATGGTCAGGTGTCAGCCGCCACTATTAAACCATGGAAGGCCCTCACAAAAAGGGGAGAGTCCAGTGGACACCTTACAAAGATTGTCCAAGGACCCCAAGAGCCACTTTCAGACTTTGTGGCCAGAATGACAGAGGCCGCCAGTCGCATCTTTGGTGATTCAGAACAAGCTATGCCCCTGATTGAGCAACTTGTCTATGAACAAGCAACCCAGG
AATGTCGAGCGGCTATCGCCCCTCGCAAAAGCAAGGTTTACAAGATTGGCTCAGAGTGTGCAGGGAGCTGGGGGGACCCCTAACCAATGCAGGACTAGCTGCCGCCATTTTACAAACGCACAGGCGTAGAGATTTATCTAGTCGAAAAGCTTGTTTTAATTGTGGGAGAATGGGGCACCTTAAGAAAGATTGTCAAGCCCCTGAAAGAACAAGAGAATCGAAGCT
TTGCCATCGTTGTGGTAAAGGTTATCATAGGGCTAGCGAATGCAGGTCAGTCCGGGATGTAAAAGGGAGGCTTCTGCCCCCTATTGAAGAACCCAAGGTACCCCAATCAAAAAACGGGTCACGGGGCCCATGGTCCCAGGGCCCTCAGAAATATGGGAACCAATTTCAGAAAAGCAATCAAGAGGAAGTAGAGGTTTCCGAGGAACCCCCGGAATGGACTTGCGTG
CCGCCTCCGACTTCTTACTCATGCCCCAAATGAATGTTCAACCGGTCCCAGTCCATTCCCCTGGACCTTTGCCCCCTGCCACCATTGGGCTTATTCTGGGCCGTGGCTCCTTAACTTTACAGGGACTCATTATATATCCTGGAATTGTTGACCCGTATCATAAAGAGGAGATTCAAGTTCTCTGTTCTAGCCCTCGGGGTGTATTTTCTATAAAGCAGGGAGATAG
AATTGCACAACTGGTGTTGCTGCCCTCCCTCGGGGATGGAGAAACCTACACTTTACAAAAAGGGCTATGGGATCTTCTGGAAGTGATTCAGCCTATCTGGCTATACCCCTAAATGAGAGACCTACAATAAAATTGTTAATTAATGGAAAAGAATTTGAAGGGATAATGGATACGGGAGCAGACAAAAGCATTATTTCACTCCATTGGTGGCCGAAGTCTTGGCCC
ACTGTTGTTTCATCTCATTCTCTACAAGGTCTTGGATACCAGTCCTCTCCTGCCATCAGTGCCTCAGCCTTAACCTGGCGGAATGCTGAGGGCAAACAGGGATGTTTTACCCCCTATGTGTTGCCACTCCCTGTAAATTTATGGGGACGGGATGTGTTACAAGCCATGGGCATGACCCTAACTAATGAATACTCCCCCAGGCATCAGCCATTATGGCAAAGATGG
GGTATACAAATGGTAGAGGCTTGGGTAGGCAAGAACAAGGCAGAATAAAACCCATTACGCAACACGGAAATCGGGGTAGAAAAGGACTGGGTTTTATTTAGGGGCCGTTGAGGCTTCACGACCCATACCGTGGAAAACAGAGGAGCCGTTATGGGTCTCTCAATGGCCTCTATCCTCTGAAAAATTAGAGGCTGTCACAAGATTAGTGCAAGAACAGGAACGGCTG
GGGCATCTAGAGCCTTCTACCTCCCCATGGAATACACCAATTTTTGTTATTAAGAAGAAATCTGGGAAGTGGAGATTACTCCATGACCTGCGGGCCATTAACAATCAGATGCATCTTTTTGGCCCTGTTCAAAGAGGCCTTCCTTTGCTTTCTGCACTTCCTCAAGATTGGAAGCTTATTATTATAGATATTAAGGATTGTTTCTTCTCTATTCCACTTTACCCAC
GGGATAGACCAAGGTTTGCCTTCACTATCCCTTCTCTTAATCATATGGAACCAGACAAGAGATTTCAGTGGAAGGTACTGCCGCAAGGCATGGCCAATAGCCCAACAATATGTCAGCTGTATGTGCAGGAGGCTTTGGAGCCAATTAGGAAGCAATTTACATCTTTAATCATGATCCATTATATGGACGATATTCTTATTTGTCACAAAGAGTTAGATGTTTTACA
AAAGGCTTTCCCCATGCTGGTGGCTGAATTAAAACAGTGGGGATTAGAGATTGCCTCAGAAAAGATTCAAATTGCAGATACAGGCCTCTTTTTGGGCTCAATGATTACTCCCAAAAATATAGTTCCTCAGAAAATAGAAATTCGCAAAGACCACTTACAAACATTAAATGATTTTCAGAAACTTCTGGGAGATATAAATTGGTTGAGGCCCTTTTTAAAAATTCCA
TCTGCTGATCTAAAACCCCTCTTCGATCTCCTGGAGGGTGAGCCTCATATCTCCTCACCCAGAAAATTTACCCCTGCCGCCCACCGAGCCTTACAGATGGTGGAAGAGGCCTTACAGGAAGCACAATTACAACGCATTGAGGAGTCACTACCTTTTAATCTGTGTGTTTTTAAGACAGCTCAATTGCCAACTGCAGTCCTGTGGCAACATGGACCATTACTTTGGA
TTCATCCAAATGCTTCCCCTGCTAAGATTATTGACTGGTACCCTGATGCTGTTGCACAGCTCGCGCTTCGCGGAATAAAAGCAGCTGTTACACATTTTGGCAGGGATCCTGATTCCTTGATTGTGCCTTATACTGCTGCACAAGTTCAGACCTTGGCAGCCACATCTAGTGATTGGGCAGTTTTAGTTACTTCCTTTTCAGGACAAATTGATAATCATTTTCCAAA
GCATCCAATTTTACAGTTTGCCCTAAACCAGGCTATAGTGTTTCCACAAGTGACAGCTAAAGACCCACTCCCAGATGGGACTGTGGTGTACACTGATGGATCAAAAACTGGTATGGGAGCTTATGTGGTCAAGGATAGGGTTATATCTAAACAATATAATGAAACTTCACCTCAAGTTGTAGAATGTCTGATAGTACTGGAGGTTCTTGAGGCTTTTCCAGGACCC
CTCAATATTGTATCAGATTCCTCTTATGTGGTTAATGCAGTTAATCTCCTTGAGATAGCTGGAATAATAAGATCCTCCAGCAGAGTTGCTAACATCTTTCAAAAGATACAAGCTGCCTTGTTAAATAGGAGATTTCCTGTTTTCATTACCCATGTCCGAGCACATTCTGGACTCCCAGGTCCCATGTCCTTAGGGAATGACTTAGCAGACAAGGCTACAAAGCTGG
TGGCCGCTGCCTTGTCTACCCATGCACAAGCAGCAAAAGAATTCCATAAGCGCTTTCATGTGACGGCTGAGACCTTGCGCCGTCGTTTTGCTCTATCCAGGAAAGAGGCTAGGGAAATTGTTACTCAATGCCAGAATTGCTGCGAATTCTTACCTACTCCCCACATGGGAATAAACCCACGTGGCATTAGACCATTGCAGATGTGGCAGATGGATGTTACTCATAT
TCCCTCTTTTGGAAGGCTTCAATATGTTCATGTTTCTGTTGACACCTGTTCTGGCGTAATGTTTGCCACACCCCTAACAGGGGAAAAGGCCTCATATGTGATTCAACATTGCCTTGAGGCCTGGAGTGCTTGGGGAAAACCCAGAATTTTAAAAACAGACAATGGACCAGCATATACCTCTCAAAAATTCCGACAGTTTTGTCGGCAAATGGATGTTACCCATCTA
ACTGGCCTACCTTATAACCCTCAAGGACAGGGCATTGTGGAACGTGCCCATCGTACACTCAAATCATATCTGATTAAACAAAAGGGGAGTATTGAGGATGTTCAACCCTCGGTACCAAGAGTTGCTGTATCCATGGCACTTTTTACCCTAATTTCTTGAACTCTGATGCCCAAGGCCACACTGTGGCCGATCGCCACAGTTTAGAGCCTGATAGGCCAAAAGAAA
TGGTAAAATGGAAAGATGTCTTGACTGATCTCTGGAAAGGCCCGGATCCTATTCTTATAAGATCCAGGGGAGCTGTGTGTGTTTTTCCACAGGAGGAAGAAAACCCCCTGTGGATCCCTGAAAGACTCACTCGAAGAGCTCCTTCACAACTCCAGAATAAGAAGGATGGAGGCCAAGTGGGTGATGAAGACTCTCCTACTCGGGATGATGGTTCCTAGTATGATGG
GAGAGTTACGGTGGGCCATCATGTCTACCTTCCCCCTACCGATGCCAGTGATGCAGAATGCCCAGGTGTTCCCTAGATTTTTCACAACTAATAGAGAGTTGGGGCTTGCCTTTTTGCCTTTAGATCCTCAGATACAGAGTTTTCAGGATGAGAGAACCTTGTCATTAGTAGGAAGCTTGTGCTTTACTGTGATTTCTTCATTAAAGGATAATTTTATTCATATGGA
TCCAGAAAGATCTAATGCTAATCCTTTTGAATTAATGGAAGCTGTACTGATCTTTCTCCTATGATAGTGAGAAGGGACAGATTACTTTTGATTGGAAAGGGGCATTAGGCAACAGTCTGAGTGAGGGACAGAGCTATGGATCATCTGATTGGTCCTCTCCAATGTTAAGTATACCCCTTTTAAGAGAACTAATTATTCAGCTGCCCTTTATTTGGGTTGTGAGTA
ATGATACCTTAGGTAAGAATCAGAAAGAAATAAATTGCTCTGCTTAGAATTGTTTTTATGCATTATGCTGGGATGCTATTAAGCACCCTTTTGCTTTGGTAATGCGCATGCCTCGATTTGTTCCAGTTCCTGTTGAGACGCCAAGTAATTTGAGTTTGTTTAGGGAAAAGCGAGATTTTTGGGATCTCTGCTATTATTGTGGGCCTGATAGCCACCGCCGCGGTTA

[illegible]

>IAPLTR3.3248119

[illegible]

>IAPLTR3.2630427

[illegible]

>IAPLTR3.3489724

[illegible]

>IAPLTR3.512706

[illegible]

>IAPLTR3.703937

[illegible]

[illegible]

>IAPLTR3.371049

[illegible]

GTAGCGCGGGACA

>IAPLTR3.4318228

[illegible]

>IAPLTR3.3363794

[illegible]

>IAPLTR3.4395110

[illegible]

[illegible]

>IAPLTR4_I.3817896

[illegible]

>IAPLTR4_I.3041411

[illegible]

>IAPLTR4_I.3794325

[illegible]

>IAPLTR4_I.193090

[illegible]

[illegible]

>IAPLTR4_I.1570666

[illegible]

>IAPLTR4_I.3054716

[illegible]

>IAPLTR4_I.3301530

[illegible]

>IAPLTR4_I.1720958

[illegible]

>IAPLTR4_I.144295

TGTGAGGAGCCGCCCTCACTATCGCTATCACCATTAGAAGATGGCGCTGACATCTGCTGTCAGTTGGAGTTTACCGTTTAAGCTGTGCCTCACCCGTCATCTGGGGTGATGAGCAAACCACCAATCCCGGCTCTACACATCTCACTCGGAGCTCCTAGGCTTATATAAGGGGCTGGGTTTCTTAGCTTGGGGTCTCCCTCTCAGAAGCTGATCATCTATCTCTCAA
GATGTATTAAAGCTTTACTGCAGAAGGATCCGAGTGTCCTGCGTCGTTCTTGCTGGTGAGACGGTAGCGCGGGACATCTGGTGCCGAAACCCGGGAACTCTTCAACATCGCCGGCGCTGCGGGAGACCCCTCGGAAGACGGGGCGGATTCAGAACTGCAGGGACGTAAGTTCAGAGAGGTATGCTTTATCCTGAACACCCCTTTTCTCGACTTTGGCTTAGTTTG
TCATCACCGTGGGACGGGTCAGTGGAGGCTTTGGTGCTAGTCCTGTTTGTCCTTCTTCATCGTGGCTGGTACTGACAGAACGGTGCTGTCAGTCTGGTAAGTCGAGCGCCGTTTTTAGATAAGCCGCTAGCGCGATGGGATTGTCACAGTCAGTGGTTACTGCCCTACAGACTGTGCTAAAACAAAGAGATCTCAAAATTGCACCACGAACACTTCAAAATTTTG
TGAAGGAGGTAGATAGAGTGGCACCATGGTATGCTTATTCCGGGTCCCTCACTGTGGCCTCATGGAACAAGCTAGGTAAAGAACTAGACAGGAAGCATGCAGAAGGAGATCTCCACTTAGGTACCAAAGCCATTTGGAAGCTAGTTAAAAACTGCTTGGAGGATGAGGCCTGTCACCCTGCTATAGTAGAAAGTCAGGGAACCTTAGAGGAAGTTCAGGATAGCAT
GTCAGAAACTGAACGGAGTGAGAGAATGGGAGCTCGCAAAAGGAAAGACATGTCTAAGAAAAAAGGCCCTCCCCAAGAAGTTAAGAAAGGGGGAGAGAAAGAAGGGAGTGATCATTCACCCCCTAACAAATCTAAGAAAAAGAAAAAGCCAGAATCTAGGCTATACCCTACGGTAGAGCTAGAAGCCTTGGAGCTAGATAATTCCGATTCTGACACTCTGGATTCT
AGCGAAGAAGGTGGTCTAGAGGAGGAGGTGGCATGGTATGAGGAAGAAAGATATCACCCTGATAGACACCGGCCGCTGAAAACAAAAATGAATGTGAGGCCACCACCTATAAATCCGGCGGGCTCACGCCCCTCGGCGCCTCCGCAGTATGAGCTTCGGCTGAATACTGGTACTGACTCCTTTTTACCTTTAGAGGAACGGAGAAAAATACAAATGGCTTTTCCAG
TATTCGAGAATGTGGAAGGCGGAAGAGTACATACTCCCGTAGATTACAATCAAATCAAAGAATTGGCCGAATCCGTTCGTAATTATGGGGTCAATGCTAACTTTACCACAATACAGGTAGAAAGGCTTGCTAATTTTGCCATGACTCCCACTGACTGGCAGACGACAGTGAAAGCAGTGCTTCCCAACATGGGACAATACATGGAATGGAAGGCTCTCTGGTATGA
TGCAGCCCAGGCGCAAGCTAGAGTTAATGCCACGGCTGAAGATGAGAATCAGAGGCAGTGGACATTTGAAATGCTGACAGGTCAGGGTCAACATGCCCTTAATCAAACCAACTACATCTGGGGCGTCTATGGTCAGGTGTCGGCCGCCGCTATTAAAGCATGGAAGGCCCTCACAAAAAGGGGAGAATCCAGTGGACACCTTACAAAGATTGTCCAAGGACCCCAA
GAGCCATTTTCAGACTTTGTGGCCAGAATGACCGAGGCCGCCGGTCGCATCTTTGGTGATTCAGAACAAGCTATGCCCCTGATTGAGCAACTTGTCTATGAACAAGCAACCCAGGAATGTCAAGCGGCTATCGCCCCTCGAAAAAGCAAAGGTTTACAAGATTGGCTCAGAGTGTGCAGGGAGCTGGGGGGACCCCTAACCAATGCAGGACTAGCTGCCGCCATTT
TACAAATGCACAGGCGTGGAGATTTATCTAGTCGAAAAGCTTGTTTTAATTGTGGGAAAATGGGGCACCTTAAGAAAGATTGTCAATCCCCTGAAAGAACAAGAGAACTGAAGCTTTGCCGTCGTTGTGGTAAAGGTTATCATAGGGCTAGTGAATGCAGGTCAGTCCGGGACATGAAAGGGAGGCTTCTGCCCCCTATTGAAGAACCCAAGGTACCCCAATCAAA
AAACGGGTCACGGGGCCCATGGTCCCAGGGCCCTCAGAAATATGGGAACCGATTTCAGAAAAGCAATCAAGAGGAAGTAGAGGCTTCCGAGGAACCCCCGGAATGGACTTGCGTGCCGCTTCCGACTTCTTACTCATGCCCCAAATGAATGTTCAACCGGTCCCAGTCCATTCCCCTGGACCTTTGCCCCCTGCCACCATTGGGCTTATTCTGGGCCGTGGCTCCT
TAACTTTACAGGGACTCATTATATATCCTGGGATTGTTGACCCGTATCATAAAGAGGAGATTCAAGTTCTCTGTTCTAGCCCTCGGGGTGTATTTTCTATAAAGCAGGGAGATAGAATTGCACAACTGGTGTTGCTGCCCTCCCTCGGGGATGGAGAAACCTACACTTTACAAAAAAGGGCTATGGGATCTTCTGGAAGTGATTCAGCCTATCTGGCTATACCCCT
AAATGAGAGACCTACAATAAAATTGTTAATTAATGGAAAAGAATTTGAAGGGATAATGGATACGGGAGCAGACAAAAGCATTATTTCACTCCATTGGTGGCCGAAGTCTTGGCCCACTGTTGTTTCATCTCATTCTCTACAAGGTCTTGGATACCAGTCCTCTCCTGCCATCAGTGCCTCAGCCTTAACCTGGCGGAACGCTGAGGGCAAACAGGGATGTTTTACC
CCCTATGTGTTGCCACTCCCTGTAAATTTATGGGGACGGGATGTGTTACAAGCCATGGGCATGACCCTAACTAATGAATACTCCCCCCAGGCATCAGCCATTATGGCAAAGATGGGGTATACAAAGGGTAGAGGCTTGGGTAGGCAAGAACAAGGCAGAATAAAACCCATTACGCAACATGGAAATCGGGGTAGAAAAGGACTGGGTTTTATTTAGGGGCCGTTGA
GGCTTCACGACCCATACCGTGGAAAACAGAGGAGCCGTTATGGGTCTCTCAATGGCCTCTATCCTCTGAAAAATTAGAGGCTGTCACAAGATTAGTGCAAGAACAGGAACGGCTGGGGCATCTAGAGCCTTCTACCTCCCCATGGAATACACCAATTTTTGTTATTAAGAAGAAATCTGGGAAGTGGAGATTACTCCATGACCTGCGGGCCATTAACAATCAGATG
CATCTTTTTGGCCCTGTTCAAAGAGGACTTCCTTTGCTTTCTGCACTCCCTCAAGATTGGAAGCTTATTATTATAGATATTAAGGATTGTTTCTTCTCTATTCCACTTTACCCACGGGATAGACTAAGGTTTGCCTTCACTATCCCTTCTCTTAATCATATGGAACCAGACAAGAGATTTCAGTGGAAGGTACTGCCGCAAGGCATGGCCAATAGCCCAACAATAT
GTCAGCTGTATGTGCAGGAGGCTTTGGAGCCAATTAGGAAGCAATTTACATCTTTAATCATGATCCATTATATGGACGATATTCTTATTTGTCACAAAGAGTTAGATGTTTTACAAAAGGCTTTTCCCATGCTGGTGGCTGAATTAAAACAGTGGGGATTAGAGATTGCCTCAGAAAAGGTTCAAATTGCAGATACAGGGCTCTTTTTGGGCTCAATGATTACTCC
CAAAAATATAGTTCCTCAGAAAATAGAAATTCGCAAAGACCACTTACAAACATTAAATGATTTTCAGAAACTTCTGGGAGATATAAATTGGTTGAGGCCCTTTTTAAAAATTCCATCTGCTGATCTAAAACCCCTCTTCGATCTCCTGGAGGGTGAGCCTCATATCTCCTCACCCAGAAAATTTACCCCTGCTGCCCACCGAGCCTCACAGATGGTGGAAGAGGCC
TTACAGGAAGCACAATTACAACGTATTGAGGAGTCACTACCTTTTAATCTGTGTGCTTTTAAGACAGCTCAATTGCCAACTGCAGTCCTGTGGCAACATGGACCATTACTTTGGATTCATCCAAATGCTTCCCCTGCTAAGATTATTGACTGGTACCCTGATGCTGTTGCACAGCTCGCGCTTCGTGGAATAAAAGCAGCTGTTACGCATTTTGGCAGGGATCCTG
ATTCCTTGATTGTGCCTTATATTGCTGCACAAGTTCAGACCTTGGCAGCCACATCTAGTGATTGGGCAGTTTTAGTTACTTCCTTTTCAGGACAAATTGATAATCATTTTCCAAAGCATCCAATTTTACAGTTTGCCCTAAACCAGGCTATAGTGTTTCCACAAGTGACAGCTAAAGACCCACTCCCAGATGGGACTGTGGTGTACACTGATGGATCGAAAACTGG
TATGGGAGCTTATGTGGTCAAGGATAGGGTTATATCTAAACAATATAACGAAACTTCACCTCAAGTTGTAGAATGTCTGATAGTACTGGAGGTTCTTGAGGCTTTTCCAGGACCCCTTAATATTGTATCAGATTCCTCTTATGTGGTTAATGCAGTTAATCTCCTTGAGATAGCTGGAATAATAAGATCCTCCAGCAGAGTTGCTAACATCTTACAGAAGATACAA
GCTGCCTTGTTAAATAGGAGATTTCCTGTTTTCATTACCCATGTCCGAGCACATTCTGGACTCCCAGGTCCCATGTCCTTAGGGAATGACTTAGCGGACAAGGCTACAAAGCTGGTGGCCGCTGCCTTGTCTACCCACACACAAGCAGCAAAAGAATTCCATCAGCGCTTTCATGTGACGGCTGAGACCTTGCGCCGTCGTTTTGCTCTATCCAGGAAAGAGGCTA
GGGAAATTGTTACTCAATGCCAGAATTGCTGTGAATTCTTACCTGCTCCCCACGTGGGAATAAACCCACGTGGCATTAGACCATTGCAGATGTGGCAGATGGATGTTACTCATATTCCCTCTTTTAGAAGGCTTCAATATGTTCATGTTTCTGTTGACACCTGTTCTGGCGTAATGTTTGCCACGCCCCTAACAGGGGAAAAGGCCTCATATGTGATTCAACATTG
CCTTGAGGCCTGGAGTGCTTGGGGAAACCCCAGAATTTTAAAAACAGACAATGGACCAGCATTTACCTCTCAAAAATTCCGACAGTTTTGTCGGCAAATGGATGTTACCCATCTAACTGGCCTACCTTATAACCCTCAAGGACAGGGCATTGTGGAACGTGCCCATCGTACACTCAAATCATATCTGATTAAACAAAAGGGGAGTATTGAGGATGTTCTACCCTCG
GTACCAAGAGTTGCTGTGTCCATGGCACTTTTTACCCTTAATTTCTTGAACTCTGATGCCCAAGGCCACACTGCGGCCGATCGCCACAGTTTAGAGCCTGATAGGCCAAAAGAATTTGTAAAATGGAAAGATGTGCTAACTAATGAATGGAAAGGCCCGGATCCTATTCTAATAAGATCCAGGGGAGCTGTGTGTGTTTTTCCACAGGAGGAAGAAAACCCCCTGT
GGATCCCTGAAAGACTCACTCGAAGAGCTCCTTCACAACTCCAGAATAAGAAGGATGGAGGCCAAGTGGGTGATGAAGACTCTCCTACTCGGGATGATGGTTCCTAGTATGATGGGAGAGTTACGGTGGGCCATCATGTCTACCTTCCCCCTACCGATGCCAGTGATGCATAATGCCCAGGAGTTCCCTAGATTTTTCACAACTAATAGAGAGTTGGGGCTTGCCT
TTTTGCCTTTAGATCCTCAGATACAGAGTTTTCAGGATGAGAGAACCTTGTCATTGGTAGGAAGCTTGTGCTTTACTGTGATTTCTTCATTAAAGGATAATTTTATTCATATGGATCCAGAAAGATCTAATGCTAATCCTTTTGAATTAATGGAAGCTGTACTGATCTTTCTCCTATGGCAATGATTGCTGGAGGATATAGTGAGAAGGGACAGATTACTTTTGAT
TGGAAAGGGGCATTAGGCAACAGTCTGAGTGAGGGACAGAGCTCTGGATCATCTGATTGGTCCTCTCCAATGTTAAGTATACCCCTTTTAAGAGAACTAATTATTCAGCTGCTCCAATTTGTGTTTGGCTGCCCTTTATTTGGGTTGTGAGTAATGATACCTTAGGTAAGAATCAGAAAGAAATAAATTGCTCTGCTTAGAATTGTTTTTATGCATTATGCTGGA
ATGCTATTAAGCACCCTTTTGCTTTGGTAATGCGCATGCCTCGATTTGTTCCAGTTCCTGTTGAGGTGCCAAGTAGTTTGAGTTTGTTTAGGGAAAAGCGAGATTTTTGGGATCTCTGCTATTATTGTGGGCCTGATAGCTGCCGCCGCGGTTACTACTTCGGTTACCGCCTCGGCCCTTGCACTTTCCGCCACTGTGCAGACAACTCAAACCATTAATGAACTAT
CGGCCACTGTGACCATGGCCCTAGATAAACAGGCCACTGCCAATTCTCAGATACAAGGAGGCCTGATGTTGGTAAACCAACAGATTAATTTGGTTCAGGACCAATTGGATATATTGTGGCAGATGGCCCAATTGGGATGTGAACAAAAGTTGCCTGGCCTTTGCGTCACTTCAGTACAATATGAGAATTTTACAAGAGCAGCTAACCTGTCAAAAGCTCTGTCTCA
ACATTTATTACAGAATTGGACTTTTGAGTTTGAGCAGACACTCCGTGAGTTGAGAGTGGCGATCCTGCAAGTGAATTCCACGCGTCTGGATCTTTCTCTGATGGAAGGCCTCTCCTCCTGGATTTCATCTGCATTTTCTTATTTTAAGGAATGGGTGGGGGTGGGATCATTTAGCCTAGTTCTCTGCTGTGGAATAGTGTTGATGCTTTGGATGGTGTGTAAGCTC
AGAGCCCAAACAAAAGAGACAAAGTGATGGTGGCACAAGCACTTGCTGCCTTAGAACACGGAGCCTCCCCTGACATTTGGTTAACCATGTTAAAGCAATAGGTCGCTGGCCTCCCGGCTCTTGCACCCCACGAGGCTAGTCTCATTGCACGGGATAGAGTGGGTAGGCTTCAGCAGCCCAAGGAGTTGCAAGGCTAAGCACTGCACAGGAGAGGTCTGCGGTATAA
CGACTTTCTCCTGGGAGATAAGTCATCTTGCATGAAGGTTCTATGTCATGATCTCCTTCCCCCAGAAAAACGACATCGGGACTGGTCAGGGCTCCCTCTGGGGATAAGACCCTGGAGAGGAGCTTTGTACATGTTCCAAATTTGCACATGGGGATTTGACCTCTATCTCCACTCCAAAGTTGTGGGTGTCCTATGGCTTCAAAAATAAATGAATAGGGGGAGATGT
GAGGAGCCGCCCTCACTATCGCTATCACCATTAGAAGATGGCGCTGACATCTGCTGTCAGTTGGAGTTTACCGTTTAAGCTGTGCCTCACCCGTCATCTGGGGTGATGAGCAAACCACCAATCCCGGCTCTACACGTCTCACTCGGAGCTCCTAGGCTTATATAAGGGGCTGGGTTTCTTAGCTTGGGGTCTCCCTCTCAGAAGCTGATCATCTATCTCTCAAGAT
GTATTAAAGCTTTACTGCAGAAGGATCCGAGTGTCCTGCGTCGTTCTTGCTGGCAAGACGGTAGCGCGGGACA

>IAPLTR4_I.562405
TGTGAGGAGCCGCCCTCACTATCGCTATCGCCATTAGAAGATGGCGCTGACATCTGCTGTCAGTTGGAGTTTACCGTTTAAGCTGTGCCTCACCCGTCATCTGGGGTGATGAGCAAACCACCAATCCCGGCTCTACACGTCTCACTCGGAGCTCCTAGGCTTATATAAGGGGCTGGGTTTCTTAGCTTGGGGTCTCCCTCTCAGAAGCTGATCATCTATCTCTCAA
GATGCATTAAAGCTTTACTGCAGAAGGATCCGAGTGTCCTGCGTCGTTCTTGCTGGCGAGATGGTAGCGCGGGACATCTGGTGCCGAAACCCGGGAACTCTTCAACATCACTGGCGCCGCGGGAGACCCCTCGGAAGACGGGGCAGATTCAGAACTGCAGGGACGTAAGTTCAGAGAGGTATGCTTTATCCTGAACACCCCTTTTCTCGACTTTGGCCTTAGTTTG
TCATCACCGTGGGACGGGTCAGTGGAGGCTTTGGTGCTAGTCCTGTTTGTCCTTCTTCATCGTGGCTGGTACTGACAGAACGGTGCTGTCAGTCTGGTAAGTCGAGCGCCGTTTTTAGATAAGCCGCTAGCGCGATGGGGTTGTCACAGTCAGTGGTTACTGCCCTACAGACTGTGCTAAAACAAAGAGATCTCAAAATTGCACCACGAACACTTCAAAATT
TTGTGAAGGAGGTAGATAGAGTGGCACCATGGTATGCTTGTTCCGGGTCCCTCACTGTGGCCTCATGGAACAAGCTAGGTAAAGAACTAGACAGGAAGCATGCAGAAGGAGATCTCCACTTAGGTACCAAAGCCATTTGGAAGCTAGTTAAAAACTGCTTGGAGGATGAGGCCTGTCACCCTGCTATAGTAGAAAGTCAGGGAACCTTAGAGGAAGTTCAGGATAG
CATGTCAGAAACTGAACGGAGTGAGAGAATGGGAGCTCGCAAAAGGAAAGACATGTCTAAGAAAAAAGGCCCTCCCCAAGAAGTTAAGAAAGGGGGAGAGAAAGAAGGGAGTGATCATTCACCCCCTAACAAATCTAAGAAAAAGAAAAAGCCAGAATCTAGGCTATACCCTACGGTAGAGCTAGAAGCCTTGGAGCTAGATAATTCCGATTCTGACACTCTGGAT
TCTAGCGAAGAAGGTGGTCTAGAGGAGGAGGTGGCACGGTATGAGGAAGAAAGATATCACCCTGATAGACACCGGCCGCTGAAAACAAAAATGAATGTGAGGCCACCACCTATAAATCCGGCGGGCTCATGCCCCTCGGCGCCTCCGCAGTATGAGCTTCGGCTGAATACTGGTACTGACTCCTTTTTACCTTTAGAGGAACGGAGAAAAATACAAATGGCTTTTC
CAGTATTCGAGAATGTGGAAGGCAGAAGAGTACATACTCCCGTAGATTACAATCAAATCAAAGAATTGGCCGAATCCGTTCGTAATTATGGGGTCAATGCTAACTTTACCACAATACAGGTGGAAAGGCTTGCTAATTTTGCCATGACTCCCACTGACTGGCAGACGACAGTGAAAGCAGTGCTTCCCAACATGGGACAATACATGGAATGGAAGGCTCTCTGGTA
TGATGCAGCCCAGGCGCAAGCTAGAGTTAATGCCACAGCTGAAGATGAGAATCAGAGGCAGTGGACATTTGAAATGCTGACAGGTCAGGGTCAACATGCCCTTAATCAAACCAACTACATCTGGGGCGTCTATGGTCAGGTGTCGGCCGCCGCTATTAAAGCATGGAAGGCCCTCACAAAAAGGGGAGAATCCAGTGGACACCTTACAAAGATTGTCCAAGGACCC
CAAGAGCCATTTTCAGACTTTGTAGCCAGAATGACCGAGGCCGCCGTTCGCATCTTTGGTGATTCAGAACAAGCTATGCCCCTGATTGAGCAACTTGTCTATGAACAAGCAACCCAGGAATGTCGAGTGGCTATCGCCCCTCGCAAAAGCAAAGGTTTACAAGATTGGCTCAGAGTGTGCAGGGAGCTGGGGGGACCCCTAACCAATGCAGGACTAGCTGCCGCCA
TTTTACAAACGCACAGGCGTAGAGATTTATCTAGTCGAAAAGTTTGTTTTAATTGTGGGAAAATGGGGCACCTTAAGAAAGATTGTCAATCCCCTGAAAGAACAAGAGAACTGGAGCTTTGCCGTCATTGTGGTAAAGGTTATCATAGGGCTAGTGAATGCAGGTCAGTCCGGGATATGAAAGGGAGGCTTCTGCCCCCTATTGAAGAACCCAAGGTACCCCAATC
AAAAAACGGGTCACGGGGCCCATGGTCCCAGGGCCCTCAGAAATATGGGAACCGATTTCAGAAAAGCAATCAAGAGGAAGTAGAGGCTTCCGAGGAACCCCCGGAATGGACTTGCGTGCCGCTTCCGACTTCTTACTCATGCCCCAAATGAATGTTCAACCGGTCCCAGTCCATTCCCCTGGACCTTTGCCCCCTGCCACCATTGGGCTTATTCTGGGCCGTGGCT
CCTTAACTTTACAGGGACTCATTATATATCCTGGGATTGTTGACCCGTATCATAAAGAGGAGATTCAAGTTCTCTGTTCTAGCCCTCGGGGTATATTTTCTATAAAGCAGGGAGATAGAATTGCACAACTGGTGTTGCTGCCCTCCCTCGGGGATGGAGAAACCTACACTTTACAAAAAAGGGCTATGGGATCTTCTGGAAGTGATTCAGCCTATCTGGCTATACC
CCTAAATGAGAGACCTACAATAAAATTGTTAATTAATGGAAAAGAATTTGAAGGGATAATGGATACGGGAGCAGACAAAAGCATTATTTCACTCCATTGGTGGCCGAAGTCTTGGCCCACTGTTGTTTCATCTCATTCTCTACAAGGTCTTGGATACCAGTCCTCTCTTGCCATCAGTGTCTCAGCCTTAACCTGGCGGAACGCTGAGGGCAAACAGGGATGTTTT
ACCCCCTATGTGTTGCCACTCCCTGTAAATTTATGGGGATGGGATGTGTTACAAACCATGGGCATGACCCTAACTAATGAATACTCCCCCAGGCATCAGCCATTGTGGCAAAGATGGGGTATACAAAGGGTAGAGGCTTGGGTAGGCAAGAACAAGGCAGAATAAAACCCATTACACAACATGGAAATCGGGGTAGAAAAGGACTGGGTTTTATTTAGGGGCCGT
TGAGGCTTCACGACCCATACCGTGGAAAACAGAGGAGCCGTTATGGGTCTCTCAATGGCCTCTATCCTCTGAAAAATTAGAGGCTGTCACAAGATTAGTGCAAGAACAGGAACGGCTGGGGCATCTAGAGCCTTCTACCTCCCCATGGAATACACCAATTTTTGTTATTAAGAAGAAATCTGGGAAGTGGAGATTACTCCATGACCTGCGGGCCATTAACAATCAG
ATGCATCTTTTTGGCCCTGTTCAAAGAGGACTTCCTTTGCTTTCTGCACTCCCTCAAGATTGGAAGCTTATTATTATAGATATTAAGGATTGTTTCTTCTCTATTCCACTTTACCCACGGGATAGACCAAGGTTTGCCTTCACTATCCCTTCTCTTAATCATATGGAACCAGACAAGAGATTTCAGTGGAAGGTACTGCCGCAAGGCATGGCCAATAGCCCAACAA
TATGTCAGCTGTATGTGCAGGAGGCTTTGGAGCCAATTAGGAAGCAATTTACATCTTTAATCATGATCCATTATATGGACGATATTCTTATTTGTCACAAAGAGTTAGATGTTTTACAAAAGGCTTTTCCCATGCTGGTGGCTGAATTAAAACAGTGGGGATTAGAGACTGCCTCAGAAAAGGTTCAAATTGCAGATACAGGCCTCTTTTTGGGCTCAATGATTAC
TCCCAAAAATATAGTTCCTCAGAAAATAGAAATTCGCAAAGACCACTTACAAACATTAAATGATTTTCAGAAACTTCTGGGAGATATAAATTGGTTGAGGCCCTTTTTAAAAATTCCATCTGCTGATCTAAAACCCCTCTTCGATCTCCTGGAGGGTGAGCCTCATATCTCCTCACCCAGAAAATTTACCCCTGCCGCCCACCGAGCCTTACAGATGGTGGAAGAG
GCCTTACAGGAAGCACAATTACAACGTATTGAGGAGTCACTACCTTTTAATCTGTGTGTTTTTAAGACAGCTCAATTGCCAACTGCAGTCCTGTGGCAACATGGACCATTACTTTGGATTCATCCAAATGCTTCCCCTGCTAAGATTATTGACTGGTACCCTGATGCTGTTGCACAGCTCGCGCTTCGAGGAATAAAAGCAGCTGTTACGCATTTTGGCAGGGATC
CTGATTCCTTGATTGTGCCTTATACTGCTGCACAAGTTCAGACCTTGGCAGCCACATCTAGTGATTGGGCAGTTTTAGTTACTTCCTTTTCAGGACAAATTGATAATCATTTTCCAAAGCATCCAATTTTACAGTTTGCCCTAAACCAGGCTATAGTGTTTCCACAAGTGACAGCTAAAGACCCACTCCCAGATGGGACTGTGGTGTACACTGATGGATCGAAAAC
TGGTATGGGAGCTTATGTGGTCAAGGATAGGGTTATATCTAAACAATATAATGAAACTTCACCTCAAGTTGTAGAATGTCTGATAGTACTGGAGGTTCTTGAGGCTTTTCCAGGACCCCTCAATATTGTATCAGATTCCTCTTATGTGGTTAATGCAGTTAATCTCCTTGAGATAGCTGGAATAATAAGATCCTCCAGCAGAGTTGCTAACATCTTTCAGAAGATA
CAAGCTGCCTTGTTAAATAGGAGATTTCCTGTTTTCATTACCCATGTCCGAGCACATTCTGGACTCCCAGGTCCCATGTCCTTAGGGAATGACTTAGCGGACAAGGCTACAAAGCTGGTGGCCGCTGCCTTGTCTACCCACACACAAGCAGCAAAAGAATTCCATCAGCGCTTTCATGTGACGGCTGAGACCTTGCGCCGTCGTTTTGCTCTATCCAGGAAAGAGG
CTAGGGAAATTGTTACTCAATGCCAGAATTGCTGCGAATTCTTACCTGCTCCCCACGTGGGAATAAACCCACATGGCATTAGACCATTGCAGATGTGGCAGATGGATGTTACTCATATTCCCTCTTTTGGAAGGCTTCAATATGTTCATGTTTCTGTTGACACCTGTTCTGGCGTAATGTTTGTCACACCCCTAACAGGGGAAAAGGCCTCATATGTGATTCAACA
TTGCCTTGAGGCCTGGAGTGCTTGGGGAAAACCCAGAATTTTAAAAACAGACAATGGACCAGCATATACCTCTCAAAAATTCCGACAGTTTTGTCGGCAAATGGATGTTACCCATCTAACTGGCCTACCTTATAACCCTCAAGGACAGGGCATTGTGGAACGTGCCCATCGTACACTCAAATCATATCTGATTAAACAAAAGAGGAGTATTGAGGATGTTCTACCC
TCGGTACCAAGAGTTGCTGTGTCCATGGCACTTTTTACCCTTAATTTCTTGAACTCTGATGCCCAAGGCCACACTGCGGCCGATCGCCACAGTTTAGAGCCTGATAGGCCAAAAGAATTTGTAAAATGGAAAGATATGCTAACTAATGAATGGAAAGGCCCGGATCCTATTCTAATAAGATCCAGGGGAGCTGTGTGTGTTTTTCCACAGGAGGAAGAAAACCCCC
TGTGGATCCCTGAAAGACTCACTCGAAGAGCTCCTTCACAACTCCAGAATAAGAAGGATGGAGGCCAAGTGGGTGATGAAGACTCTCCTACTCGGGATGATGGTTCCTAGTATGATGGGAGAGTTACGGTGGGCCATCATGTCTACCTTCCCCCTACCGATGCCAGTGATGCATAATGCCCAGGAGTTCCCTAGATTTTTCACAACTAATAGAGAGTTGGGGCTTG
CCTTTTTGCCTTTAGATCCTCAGATACAGAGTTTTCAGGATGAGAGAACCTTGTCATTGGTAGGAAGCTTGTGCTTTACTGTGATTTCTTCATTAAAGGATAATTATATTCATATGGATCCAGAAAGATCTAATGCTAATCCTTTTGAATTAATGGAAGCTGTACTGATCTTTCTCCTATGGCAATGATTGCTGGAGGATATAGTGAGAAGGGACAGATTACTTTT
GATTGGAAAGGGGCATTAAGCAACAGTCTGAGTGAGGGACAGAGCTCTGGATCATCTGATTGGTCCTCTCCAATGTTAAGTATACCCCTTTTAAGAGAACTAATTATTCAGCTGCTCCAATTTGTGTTTGGCTGCCCTTTATTTGGGTTGTGAGTAATGATACCTTAGGTAAGAATCAGAAAGAAATAAATTGCTCTGCTTAGAATTGTTTTTATGCATTATGCT
GGGATGCTATTAAGCACCCTTTTGCTTTGGTAATGCGCATGCCTCGATTTGTTCCAGTTCCTGTTGAGGTGCCAAGTAGTTTGAGTTTGTTTAGGGAAAAGCGAGATTTTTGGGATCTCTGCTATTATTGTGGGCCTGATAGCTGCCGCCGCGGTTACTGCTTCGGTTACCGCCTCGGCCCTTGCACTTTCCGCCACTGTGCAGACAACTCAAACCATTAATGAAC
TATCGGCCACTGTGACCATGGCCCTAGATAAACAGGCCACTGCCAATTCTCAGATACAAGGAGGCCTGATGTTGGTAAACCAACAGATTAATTTGGTTCAGGAACAATTGGATATATTGTGGCAGATGGCCCAATTGGGATGTGAACAAAAGTTGCCTGGCCTTTGTGTCACTTCAGTACAATATGAGAATTTTACAAGAGCAGCTAACCTGTCAAAAGCTCTGTC
TCAACATTTATTACAGAATTGGACTTTTGAGTTTGAGCAGACACTCCGTGAGTTGAGAGTGGCGATCCTGCAAGTGAATTCGACGTGTCTGGATCTTTCTCTGATGGAAGGCCTCTCCTCTTGGATTTCATCTGCATTTTCTTATTTTAAGGAATGGGAGGGGGTGGGATCATTTAGCCTAGTTCTCTGCTGTGGAATAGTGTTGATGCTTTGGATGGTGTGTAAG
CTCAGAGCCCAAACAAAAGAGACAAAGTGATGGTGGCACAAGCACTTGCTGCCTTAGAACACGGAGCCTCCCCTGACATTTGGTTAACCATGTTAAAGCAATAGGTCGCTGGCCTCCCGGCTCTTGCACCCCATGAGGCTAGTCTCATTGCACGGGATAGAGTGGGTAGGCTTCAGCAGCCCAAGGAGTTGCAAGGCTAAGCACTGCACAGGAGAGGTCTGCGGTA
TAACGACTTTCTCCTGGGAGATAAGTCATCTTGCATGAAGGTTCTATGTCATGATCTCCTTCCCCCAGAAAAACGACATCAGGACTGGTCAGGGCTCCCTCTGGGGATAAGACCCTGGAGAGGAGCTTTGTACATGTTCCAAATTTGCACATGGGGATTTGACCTCTATCTCCACTCCAAAGTTGTGGGTGTCCTATGGCTTCAAAAATAAATGAATAGGGGGAGA
TGTGAGGAGCCGCCCTCACTATCGCTATCGCCATTAGAAGATGGCGCTGACATCTGCTGTCAGTTGGAGTTTACCGTTTAAGCTGTGCCTCACCCGTCATCTGGGGTGATGAGCAAACCACCAATCCCGGCTCTACCCGTCTCACTCGGAGCTCCTAGGCTTATATAAGGGGCTGGGTTTCTTAGCTTGGGGTCTCCCTCTCAGAAGCTGATCATCTATCTCTCAA
GATGCATTAAAGCTTTACTGCAGAAGGATCCGAGTGTCCTGCGTCGTTCTTGCTGGCGAGACGGTAGCGCGGGACA

>IAPLTR4_I.1474216
TGTGAGGAGCCGCCCTCATTATCGCTATCGCCATTAGAAGATGGCGCTGACATCTGCTGTCAGTTGGAGTTTACCGTTTAAGCTGTGCCTCACCCGTCATCTGGGGTGATGAGCAAACCACCAATCCCGGCTCTACACGTCTCACTCGGAGCTCCTAGGCTTATATAAGGGGCTGGGTTTCTTAGCTTGGGGTCTCCCTCTCAGAAGCTGATCATCTATCTCTCAA
GATGTATTAAAGCTTTACTGCAGAAGGATCCGAGTGTCCTGCGTCGTTCTTGCTGGCGAGACGGTAGCGCGGGACATCTGGTGCCGAAACCCGGGAACTCTTCAACATCGCCGGCGCCGCGGGAGACCCCTCGGAAGACGGGGCGGATTCAGAACTGCAGGGACGTAAGTTCAGAGAGGTATGCTTTATCCTGAACACCCCTTTTCTCAACTTTGGCCTTAGTTTG
TCATCACCGTGGGACGGGTCAGTGGAGGCTTTGGTGCTAGTCCTGTTTGTCCTTCTTCATCGTGGCTGGTACTGACAGAACGGTGCTGTCAGTCTGGTAAGTCGAGCGCCGTTTTTAGATAAGCCGCTAGCGCGATGGGGTTGTCACAGTCAGTGGTTACTGCCCTACAGACTGTGCTAAAACAAAGAGATCTCAAAATTGCACCACGAACACTTCAAAATTTTG
TGAAGGAGGTAGATAGAGTGGCACCATGGTATGCTTGTTCCGGGTCCCTCACTGTGGCCTCATGGAACAAGCTAGGTAAAGAACTAGACAGGAAGCATGCAGAAGGAGATCTCCACTTAGGTACCAAAGCCATTTGGAAGCTAGTTAAAAACTGCTTGGAGGATGAGGCCTGTCACGCTGCTATAGTAGAAAGTCAGGGAACCTTAGAGGAAGTTCAGGATAGCAT
GTCAGAAACTGAACGGAGTGAGAGAATGGGAGCTCGCAAAAGGAAAGACATGTCTAAGAAAAAAGGCCCTCCCCAAGAAGTTAAGAAAGGGGGAGAGAAAGAAGGGAGTGATCATTCACCCCCTAACAAATCTAAGAAAAAGAAAAAGCCAGAATCTAGGCTATACCCTACGGTAGAGCTAGAAGCCTTGGAGCTAGATAATTCCGATTCTGACACTCTGGATTCT
AGCGAAGAAGGTGGTCTAGAGGAGGAGGTGGCACGGTATGAGGAAGAAAGATATCACCCTGATAGACACCGGCCGCTGAAAACAAAAATGAATGTGAGGCCACCACCTATAAATCCGGCGGGCTCACGCCCCTCGGCGCCTCCGCAGTATGAGCTTCGGCTGAATACTGGTACTGACTCCTTTTTACCTTTAGAGGAACGGAGAAAAATACAAATGGCTTTTCCAG
TATTCGAGAATGTGGAAGGCAGAAGAGTACATACTCCCGTAGATTACAATCAAATCAAAGAATTGGCCGAATCCGTTCGTAATTATGGGGTCAATGCTAACTTTACCACAATACAGGTAGAAAGGCTTGCTAATTTTGCCATGACTCCCACTGACTGGCAGACGACAGTGAAAGCAGTGCTTCCCAACATGGGACAATACATGGAATGGAAGGCTCTCTGGTATGA
TGCAGCCCAGGCGCAAGCTAGAGTTAATGCCACAGCTGAAGATGAGAATCAGAGGCAGTGGACATTTGAAATGCTGACAGGTCAGGGTCAACATGCCCTTAATCAAACCAACTACATCTGGGGCATCTATGGTCAGGTGTCAGCCGCCGCTATTAAAGCATGGAAGGCCCTCACAAAAAGGGGAGAATCCAGTGGACACCTTACAAAGATTGTCCAAGGACCCCAA
GAGCCATTTTCAGACTTTGTGGCCAGAATGACCGAGGCCGCCGGTCGCATCTTTGGTGATTCAGAACAAGCTATGCCCCTGATTGAGCAACTTGTCTATGAACAAGCAACCCAGGAATGTCGAGCGGCTATCGCCCCTCGCAAAAGCAAAGGTTTACAAGATTGGCTCAGAGTGTGCAGGGAGCTGGGGGGACCCCTAACCAATGCAGGACTAGCTGCCGCCATTT
TACAAACGCACAGGCGTAGAGATTTATCTAGTCGAAAAGCTTGTTTTAATTGTGGGAAAATGGGGCACCTTAAGAAAGATTGTCAATCCCCTGAAAGAACAAGAGAACTGAAGCTTTGCCATCGTTGTGGTAAAGGTTATCATAGGGCTAGTGAGTGCAGGTCAGTCCGGGATATGAAAGGGAGGCTTCTGCCCCCTATTGAAGAACCCAAGGTACCCCAATCAAA
AAACGGGTCACGGGGCCCATGGTCCCAGGGCCCTCAGAAATATGGGAACCGATTTCAGAAAAGCAATCAAGAGGAAGTAGAGGCTTCCGAGGAACCCCCGGAATGGACTTGCGTGCCGCTTCCGACTTCTTACTCATGCCCCAAATGAATGTTCAACCGGTCCCAGTCCATTCCCCTGGACCTTTGCCCCCTGCCACCATTGGGCTTATTCTGGGCCGTGGCTCCT
TAACTTTACAGGGACTCATTATATATCCTGGGATTGTTGACCCGTATCATAAAGAGGAGATTCAAGTTCTCTGTTCTAGCCCTCGGGGTGTATTTTCTATAAAGCAGGGAGATAGAATTGCACAACTGGTGTTGCTGCCCTCCCTCGGGGATGGAGAAACCTACACTTTACAAAAAAGGGCTATGGGATCTTCTGGAAGTGATTCAGCCTATCTGGCTATACCCCT
AAATGAGAGACCTACAATAAAATTGTTAATTAATGGAAAAGAATTTGAAGGGATAATGGATACGGGAGCAGACAAAAGCATTATTTCACTCCATTGGTGGCCGAAGTCTTGGCCCACTGTTGTTTCATCTCATTCTCTACAAGGTCTTGGATACCAGTCCTCTCCTGCCATCAGTGCCTCAGCCTTAACCTGGCGGAACGCTGAGGGCAAACAGGGATGTTTTACC
CCCTATGTGTTGCCACTCCCTGTAAATTTATGGGGACGGGATGTGTTACAAGCCATGGGCATGACCCTAACTAATGAATACTCCCCCCAGGCATCAGCCATTATGGTAAAGATGGGGTATACAAAGGGTAGAGGCTTGGGTAGGCAAGAACAAGGCAGAATAAAACCCATTACACAACATGGAAATCGGGGTAGAAAAGGACTGGGTTTTATTTAGGGGCCGTTGA
GGCTTCACGACCCATACCGTGGAAAACAGAGGAGCCGTTATGGGTCTCTCAATGGCCTCTATCCTCTGAAAAATTAGAGGCTGTCACAAGATTAGTGCAAGAACAGGAACGGCTGGGGCATCTAGAGCCTTCTACCTCCCCATGGAATACACCAATTTTTGTTATTAAGAAGAAATCTGGGAAGTGGAGATTACTCCATGACCTGCGGGCCATTAACAATCAGATG
CATCTTTTTGGCCCTGTTCAAAGAGGACTTCCTTTGCTTTCTGCACTCCCTCAAGATTGGAAGCTTATTATTATAGATATTAAGGATTGTTTCTTCTCTATTCCACTTTACCCACGGGATAGACCAAGGTTTGCCTTCACTATCCCTTCTCTTAATCATATGGACCAGACAAGAGATTTCAGTGGAAGGTACTGCCGCAAGGCATGGCCAATAGCCCAACAATAT
GTCAGCTGTATGTGCAGGAGGCTTTGGAGCCAATTAGGAAGCAATTTACATCTTTAATCATGATCCATTATATGGACGATATTCTTATTTGTCACAAAGAGTTAGATGTTTTACAAAAGGCTTTTCCCATGCTGGTGGCTGAATTAAAACAGTGGGGATTAGAGATTGCCTCAGAAAAGGTTCAAATTGCAGATACAGGGCTCTTTTTGGGCTCAATGATTACTCC
CAAAAATATAGTTCCTCAGAAAATAGAAATTCGCAAAGACCACTTACAAACATTAAATGATTTTCAGAAACTTCTGGGAGATATAAATTGGTTGAGGCCCTTTTTAAAAATTCCATCTGCTGATCTAAAACCCCTCTTCAATCTCCTGGAGGGTGAGCCTCATATCTCCTCACCCAGAAAATTTACCCCTGCTGCCCACCGAGCCTTACAGATGGTGGAAGAGGCC
TTACAGGAAGCACAATTACAACGTATTGAGGAGTCACTACCTTTTAATCTGTGTGTTTTTAAGACAGCTCAATTGCCAACTGCAGTCCTGTGGCAACATGGACCATTACTTTGGATTCATCCAAATGCTTCCCCTGCTAAGATTATTGACTGGTACCCTGATGCTGTTGCACAGCTCGCGCTTCGCGGAATAAAAGCAGCTGTTACGCATTTTGGCAGGGATCCTG
ATTCCTTGATTGTGCCTTATACTGCTGCACAAGTTCAGACCTTGGCAGCCACATCTAGTGATTGGGCAGTTTTAGTTACTTCCTTTTCAGGACAAATTGATAATCATTTTCCAAAGCATCCAATTTTACAGTTTGCCCTAAACCAGGCTATAGTGTTTCCACAAGTGACAGCTAAAGACCCACTCCCAGATGGGACTGTGGTGTACACTGATGGATCGAAAACTGG
TATGGGAGCTTATGTGGTCAAGGATAGGGCTATATCTAAACAATATAATGAAACTTCACCTCAAGTTGTAGAATGTCTGATAGTACTGGAGGTTCTTGAGGCTTTTCCAGGACCCCTCAATATTGTATCAGATTCCTCTTATGTGGTTAATGCAGTTAATCTCCTTGAGATAGCTGGAATAATAAGATCCTCCAGCAGAGTTGCTAACATCTTTCAGAAGATACAA
GCTGCCTTGTTAAATAGGAGATTTCCTGTTTTCATTACCCATGTCCGAGCACATTCTGGACTCCCAGGTCCCATGTCCTTAGGGAATGACTTAGCGGACAAGGCTACAAAGCTGGTGGCCGCTGCCTTGTCTACCCACACACAAGCAGCAAAAGAATTCCATCAGCGCTTTCATGTGACGGCTGAGACCTTGCGCCGTCGTTTTGCTCTATCCAGGAAAGAGGCTA
GGGAAATTGTTACTCAATGCCAGAATTGCTGCAAATTCTTACCTGCTCCCCACGTGGGAATAAACCCACGTGGCATTAGACCATTGCAGATGTGGCAGATGGATGTTACTCATATTCCCTCTTTTGGAAGGCTTCAATATGTTCATGTTTCTGTTGACACCTGTTCTGGCGTAATGTTTGCCACACCCCTAACAGGGGAAAAGGCCTCATATGTGATTCAACATTG
CCTTGAGGCCTGGAGTGCTTGGGGAAAACCCAGAATTTTAAAAACAGACAATGGACCAGCATATACCTCTCAAAAATTCCGACAGTTTTGTCGGCAAATGGATGTTACCCATCTAACTGGCCTACCTTATAACCCTCAAGGACAGGGCATTGTGGAACGTGCCCATCGTACACTCAAATCATATCTGATTAAACAAAAGGGGAGTATTGAGGATGTTCTACCCTCG
GTACCAAGAGTTGCTGTGTCCATGGCACTTTTTACCCTTAATTTCTTGAACTCTGATGCCCAAGGCCACACTGGGGCCGATCGCCACAGTTTAGAGCCTGATAGGCCAAAAGAATTTGTAAAATGGAAAGATGTGCTAACTAATGAATGGAAAGGCCCGGATCCTATTCTAATAAGATCCAGGGGAGCTGTGTGTGTTTTTCCACAGGAGGAAGAAAACCCCCTGT
GGATCCCTGAAAGACTCACTCGAAGAGCTCATTCACAACTCCAGAATAAGAAGGATGGAGGCCAAGTGGGTGATGAAGACTCTCCTACTCGGGATGATGGTTCCTAGTATGATGGGAGAGTTACGGTGGGCCATCATGTCTACCTTCCCCCTACCGATGCCAGTGATGCATAATGCCCAGGAGTTCCCTAGATTTTTCACAACTAATAGAGAGTTGGGGCTTGCCT
TTTTGCCTTTAGATCCTCAGATACAGAGTTTTCAGGATGAGAGAACCTTGTCATTGGTAGGAAGCTTGTGCTTTACTGTGATTTCTTCATTAAAGGATAATTTTATTCATATGGATCCAGAAAGATCTAATGCTAATCCTTTTGAATTAATGGAAGCTGTACTGATCTTTCTCCTATGGCAATGATTGCTGGAGGATATAGTGAGAAGGGACAGATTACTTTTGAT
TGGAAAGGGGCATTAGGCAACAGTCTGAGTGAGGGACAGAGCTCTGGATCATCTGATTGGTCCTCTCCAATGTTAAGTATACCCCTTTTAAGAGAACTAATTATTCAGCTGCTCCAATTTGTGTTTGGCTGCCCTTTATTTGGGTTGTGAGTAATGATACCTTAGGTAAGAATCAGAAAGAAATAAATTGCTCTGCTTAGAATTGTTTTTATGCATTATGCTGGG
ATGCTATTAAGCACCCTTTTGCTTTGGTAATGCGCATGCCTCGATTTGTTCCAGTTCCTGTTGAGGTGCCAAGTAGTTTGAGTTTGTTTAGGGAAAAGCGAGATTTTTGGGATCTCTGCTATTATTGTGGGCCTGATAGCTGCCGCCGCGGTTACTGCTTCGGTTACCGCCTCGGCCCTTGCACTTTCCGCCACTGTGCAGACAACTCAAACCATTAATGAACTAT
CAGCCACTGTGACCATGGCCCTAGATAAACAGGCCACTGCCAATTCTCAGATACAAGGAGGCCTGATGTTGGTAAACCAACAGATTAATTTGGTTCAGGAACAATTGGATATATTGTGGCAGATGGCCCAATTGGGATGTGAACAAAAGTTGCCTGGCCTTTGTGTCACTTCAGTACAATATGAGAATTTTACAAGAGCAGCTAACCTGTCAAAAGCTCTGTCTCA
ACATTTATTACAGAATTGGACTTTTGAGTTTGAGCAGACACTCCGTGAGTTGAGAGTGGCGATCCTGCAAGTGAATTCCACGCGTCTGGATCTTTCTCTGATGGAAGGCCTCTCCTCCTGGATTTCATCTGCATTTTCTTATTTTAAGGAATGGGTGGGGGTGGGATCATTTAGCCTAGTTGTCTGCTGTGGAATAGTGTTGATGCTTTGGATGGTGTGTAAGCTC
AGAGCCCAAACAAAAGAGACAAAGTGATGGTGGCACAAGCACTTGCTGCCTTAGAACACGGAGCCTCCCCTGACATTTGGTTAACCATGTTAAAGCAATAGGTCGCTGGCCTCCTGGCTCTTGCACCCCACGAGGCTAGTCTCATTGCACGGGATAGAGTGGGTAGGCTTCAGCAGCCCAAGGAGTTGCAAGGCTAAGCACTGCACAGGAGAGGTCTGCGGTATAA
CGACTTTCTCCTGGGAGATAAGTCATCTTGCATGAAGGTTCTATGTCATGATCTCCTTCCCCCAGAAAAACGACATCGGGACTGGTCAGGGCTCCCTCTGGGGATAAGACCCTGGAGAGGAGCTTTGTACATGTTCCAAATTTGCACATGGGGATTTGACCTCTATCTCCACTCCAAAGTTGTGGGTGTCCTATGGCTTCAAAAATAAATGAATAGGGGGAGATGT
GAGGAGCCGCCCTCATTATCGCTATCGCCATTAGAAGATGGCGCTGACATCTGCTGTCAGTTGGAGTTTACCGTTTAAGCTGTGCCTCACCCGTCATCTGGGGTGATGAGCAAACCACCAATCCCGGCTCTACACATCTCACTCGGAGCTCCTAGGCTTATATAAGGGGCTGGGTTTCTTAGCTTGGGGTCTCCCTCTCAGAAGCTGATCATCTATCTCTCAAGAT
GTATTAAAGCTTTACTGCAGAAGGATCCGAGTGTCCTGCGTCGTTCTTGCTGGCGAGACGGTAGCGCGGGACA

>IAPLTR3.41335
TGTGAGGAGCCGCCCTCGCTATCGCTATTGCCGTTAGAAGATGGCGCTGACATCTGCTGTCAGTTGGAGTTAACCGTTAGAAGATGGCGCTGACATCTGCTGTCAGTTGGAGTTAACCGTTTAAGCTGTGCCTCTCCCGTGGCGTCATCTGGGGTGATGTGCAAACCACCAATCCCGGCTCTACACGTCTCACTCGGAGCTCCTAGGCTTATATAAGGGGCTGGGT
TTCTTAGCTTGGGGTCTCCCTCTAAGAAGCTGATCATCTATCTCTCAAGATGCATTAAAGCTTTACTGCAGAAGGATCCGAGTGTTCTGCGTCGTTCTTGCTGGCGAGACGGTAGCGCGGGACATCTGGTGCCGAAACCCGGGAACTCTTCAACATCGCCGGCGCCGTGGGAGACCCCTCGGAAGACGGGGCGGATTCAGAACTGCAGGGACGTAAGTTCAGAGAG
GTATGCTTTATCCTGAACACCCCTTTCTCGACTTTGGCCTTAGTTTGTCATCACCGTGGGACGGGTCAGTGGAGGCTTTGGTGCTAGTCCTGTTTGTCCTTCTCCTTCATCGTGGCTGGTGCTGACAGAACGGTGCTGTCAGTCTGGTGCTGTCAGTCTGGTGCTGTCAGTCTGGTGCTGTCAGTCTGGTGCTGTCAGTCTGGTGCTGTCAGTCTGGTAACGGAG
TTGAGCGCCGTTTTTAGATAAGCCGCTAGCGCGATGGGGTTGTCACAGTCAGTGGTTACTGCCCTACAGACTGTGCTAAAACAAAGAGATCTTAAAATTGCACCACGAACACTTCAAAATTTTGTGAAGGAGGTAGATAGAGTGGCACCATGGTATGCTTGTTCCGGGTCCCTCACTGTGGCCTCATGGAACAAGCTAGGTAAAGAACTAGACAGGAAGCATGCA
GAAGGAGATCTCCACTTAGGTACCAAAGCCATTTGGAAGCTAGTTAAAAACTGCTTGGAGGATGAGGCCTGTCACCCTGCTATAATAGAAAGTCAGGGAACCTTAGAGGAAGTTCAGGATAGCATGTCAGAAACTGAACGGAGTGAGAGAATGGGAGCTCGCAAAAGGAAAGACATGTCTAAGAAAAAAGGCCCTCCCCAAGAAGTTAAGAAAGGGGGAGAGAAAG
AAGGGAGTGATCATTCACCCCCTAACAAATCTAAGAAAAAGAAAAAGCCAGAATCTAGTCTATACCCTACAGTAGAGCTAGAAGCCTTGGAGCTAGATAATTCTGATTCTGACACTCTGTATTCTAGCGAAGAAGGTGGTCTAGAGGAGGAGGTGGCACGGTATGAGGAAGAAAGATATCACCCTGATAGACACCGGCCACTGAAAACAAAAATGAATGTGAGGCCC
ACCGCCTATAAATCCGGCGGGCTCACGCCCCTCAGCGCCTCCGCAGTATGAGCTTCGGCTGAATACTGGTACTGACTCCTTTTTACCTTTAGAGGAACGGAGAAAAATACAAGTAGCTTTTCCAGTATTCGAGAATGCGGAAGGCGGAAGAGTACATACCCCTGTAGATTACAATCAAATCGAAGAATTGGCCGAATCCGTTCGTAATTATGGGGTCAATGCTAAC
TTTACCACATTACAGGTGGAAAGGCTTGCTAATTTTGCCATGACTCCCACTGACTGGCAGACGACAGTGAAAGCAGTGCTTCCCAACATGGGACAATACATGGAATGGAAGGCTCTCTGGTATGATGCAGCCCAGGCACAAGCTAGAGTTAATGCCACGGCTGAAGATGAGAATCAGAGGCAGTGGACATTTGAAATGCTGACAGGTCAGGGTCAACATGCCCTTA
ATCAAACCAACTACATCTGGGGTGTCTATGGTCAGGTGTCGGCCGCCGCTATTAAAGCATGGAAGGCCCTCACAAAAAGGGGAGAGTCCAGTGGACACCTTACAAAGATTGTCCAAGGACCCCAAGAGCCATTTTCAGACTTTGTGGCCAGAATGACAGAGGCCGCCAGTCGCATCTTTGGTGATTCAGAACAAGCTATGCCCCTGATTGAGCAACTTGTCTATGA
ACAAGCAACCCAGGAATGTCGAGCGGCTATCGCCCCTCGCAAAAGCAAAGGTTTACAAGATTGGCTCAGAGTGTGCAGGGAGCTGGGGGGACCCCTAACCAATGCAGGACTAGCTGCCGCCATTTTACAAACGCACAGGTATAGAGATTTATCTAGTCGAAAAGCTTGTTTTAATTGTGGGAGAATGGGGCACCTTAAGAAAGATTGTCAAGCCCCTGAAAGAACA
AGAGAATCGAAGCTTTGCCATCGTTGTGGTAAAGGTTATCATAGGGCTAGCGAATGCAGGTCAGTCCGGGATGTAAAAGGGAGGCTTCTGCCCCTTATTGAAGAACCCAAGGTACCCCAATCAAAAAACGGGTCACGGGGCCCATGGTCCCAGGGCCCTTATAAATATGGGAACCGATTTCAGAAAAGCAATCAAGAGGAAGTAGAGGCTTCCGAGGAACCCCCGG
AATGGACTTGCGTGCCGCCTCCGACTTCTTACTCATGCCCCAAATGAATGTTCAACCGGTCCCAGTCCATTCCCCTGGACCTTTGCCCCCTGCCACCATTGGGCTTATTCTGGGCCGTGGCTCCTTAACTTTACAGGGACTCATTATATATCCTGGAATTGTTGACCCGTATCATAAAGAGGAGATTCAAGTTCTCTGTTCTAGCCCTCGGGTGTATTTTCTATAA
AGCAGGGAGATAGAATTGCACAACTGGTGTTGCTGCCCTCCCTCGGGGATGGAGAAACCTACACTTTACAAAAAAGGGCTATGGGATCTTCTGGAAGTGATTCAGCCTATCTGGCTATACCCCTAAATGAGAGACCTACAATAAAATTGTTAATTAATGGAAAAGAATTTGAAGGGATAATGGATACGGGAGCAGACAAAAGCATTATTTCACTCCATTGGTGGCC
GAAGTCTTGGCCCACTGTTGTTTCATCTCATTCTCTACAAGGTCTTGGATACCAGTCCTCTCCTGCCATCAGTGCCTCAGCCTTAACCTGGCAGAATGCTGAGGGCAAACAGGGATGTTTTACCCCCTATGTGTTGCCACTCCCTGTAAATTTATGGGGACGGGATGTGTTACAAGCCATGGGCATGACCCTAACTAATGAATACTCCCCCCAGGCATCAGCCATT
ATGGCAAAGATGGGGTATACAAAGGGTAGAGGCTTGGGTAGGCAAGAACAAGGCAGAATAAAACCCATTACGCAACACGGAAATCGGGGTAGAAAAGGACTGGGTTTTATTTAGGGGCCGTTGAGGCTTCACGACCCATACCATGGAAAACAGAGGAGCCGTTATGGGTCTCTCAATGGCCTTTATCCTCTGAAAAATTAGAGGCTGTCACAAGATTAGTGCAAGA
ACAGGAACGGCTGGGGCATCTAGAGCCTTCTACCTCCCCATGGAATACACCAATTTTTGTTATTAAGAAGAAATCTGGGAAGTGGAGATTACTCCATGACCTGCGGGCCATTAACAATCAGATGCATCTTTTTGGCCCTGTTCAAAGAGGCCTTCCTTTGCTTTCTGCACTTCCTCAAGTTTGGAAGCTTGTTATTATAGATATTAAGGATTGTTTCTTCTCTATT
CCACTTTACCCACGGGATAGACCAAGGTTTGCCTTCACTATCCCTTCTCTTAATCATATGGAACCAGACAAGAGATTTCAGTGGAAGGTACTGCCGCAAGGCATGGCCAATAGCCCAACAATATGTCAGCTGTATGTGCAGGAGGCTTTGGAGCCAATTAGGAAGCAATTTACATCTTTAATCGTTATCCATTATATGGACGATATTCTTATTTGTCACAAAGAGT
TAGATGTTTTACAAAAGGCTTTCCCCATGCTGGTGGCTGAATTAAAACAGTGGGGATTAGAGATTGCCTCAGAAAAGGTTCAAATTGCAGATACAGGCCTCTTTTTGGGCTCAATGATTACTCCCAAAAATATAGTTCCTCAGAAAATAGAAATTCGCAAAGACCACTTACAAACATTAAATGATTTTCAGAAACTTCTGGGAGATATAAATTGGTTGAGGCCCTT
TTTAAAAATTCCATCTGCTGATCTAAAACCCCTCTTCGATCTCCTGGAGGGTGAGCCTCATATCTCCTCACCCAGAAAATTTACCCCTGCCGCCCACCGAGCCTTACAGATGGTGGAAGAGGCCTTACAGGAAGCACAATTACAACGTATTGAGGAGTCACTACCTTTTAATCTGTGTGTTTTTAAGACAGCTCAATTGCCAACTGCAGTCCTGTGGCAACATGGA
CCATTACTTTGGATTCATCCAAATGCTTCCCCTGCTAAGATTGACTGGTACCCTGATGCTGTTGCACAGCTCGCGCTTCGCGGAATAAAAGCAGCTGTTACACATTTTGGCAGGGATCCTGATTCCTTGATTGTGCCTTATACTGCTGCACAAGTTCAGACCTTGGCAGCCACATCTAGTGATTGGGCAGTTTTAGTTACTTCCTTTTCAGGACAAATTGATAATC
ATTTTCCAAAGCATCCAATTTTACAGTTTGCCCTAAACCAGGCTATAGTGTTTCCACAAGTGACAGCTAAAGACCCACTCCCAGATGGGACTGTGGTGTACACTGATGGATCAAAAACTGGTATGGGAGCTTATGTGGTCAAGGATAGGGTTATATCTAAACAATATAATGAAACTTCACCTCAAGTTGTAGAATGTCTGATAGTACTGGAGGTTCTTGAGGCTTT
TCCAGGACCCCTCAATATTGTATCAGATTCCTCTTATGTGGTTAATGCAGTTAATCTCCTTGAGATAGCTGGAATAATAAGATCCTCCAGCAGAGATGCTAACATCTTTCAAAAGATACAAGCTGCCTTGTTAAATAGGAGATTTCCTGTTTTCATTACCCATGTCCGAGCACATTCTGGACTCCCAGGTCCCATGTCCTTAGGGAATGACTTAGCGGACAAGGCT
ACAAAGCTGGTGGCCGCTGCCTTGTCTACCCATGCACAAGCAGCAAAAGAATTCCATAAGCGCTTTCATGTGACGGCTGAGACCTTGCGCCGTCGTTTTGCTCTATCCAGGAAAGAGGCTAGGGAAATTGTTACTCAATGCCAGAATTGCTGCGAATTCTTACCTACTCCCCACATGGGAATAAACCCACGTGGCGTTAGACCATTGCAGATGTGGCAGATGGATG
TTACTCATATTCCCTCTTTTGGAAGGCTTCAATATGTTCATGTTTCTGTTGACACCTGTTCTGGCGTAATGTTCGCCACACCCCTAACAGGGGAAAAGGCCTCATATGTGATTCAACATTGCCTTGAGGCCTGGAGTGCTTGGGGAAAACCCAGAATTTTAAAAACAGACAATGGACCAGCATATACCTCTCAAAAATTCCGACAGTTTTGTCGGCAAATGGATGT
TACCCATCTAACTGGCCTACCTTATAACCCTCAAGGACAGGGCATCGTGGAACGTGCCCATCGTACACTCAAATCATATCTGATTAAACAAAAGGGGAGTATTGAGGATGTTCTACCCTCGGTACCAAGAGTTGCTGTATCCATGGCACTTTTTACCCTTAATTTCTTGAACTCTGATGCCCAAGGCCACACTGCGGCCGATCGCCACAGTTTAGAGCCTGATAGG
CCAAAAGAATGGTAAAATGGAAAGATGTCTTGACTGATCTCTGGAAAGGCCCGGATCCTATTCTTATAAGATCCAGGGGAGCTGTGTGTGTTTTTCCACAGGAGGAAGAAAACCCCCTGTGGATCCCTGAAAGACTCACTCGAAGAGCTCCTTCACAACTCCAGAATAAGAAGGATGGAGGCCAAGTGGGTGATGAAGACTCTCCTACTCGGGATGATGGTTCCT
AGTATGATGGGAGAGTTACGGTGGGCCATCATGTCTACCTTCCCCCTACCGATGCCAGTGATGCAGAATGCCCAGGTGTTCCCTATATTTTTCACAACTAATAGAGAGTTGGGGCTTGCCTTTTTGCCTTTAGATCCTCAGATACAGAGTTTTCAGGATGAGAGAACCTTGTCATTAGTAGGAAGCTTGTGCTTTACTGTGATTTCTTCATTAAAGGATAATTTTA
TTCATATGGATCCAGAAAGATCTAATGCTAATCCTTTTGAATTAATGGAAGCTGTACTGATCTTTCTCCCTATGATAGTGAGAAGGGACAGATTACTTTTGATTGGAAAGGGGCATTAGGCAACAGTCTGAGTGAGGGACAGAGCTCTGGATCATCTGATTGGTCCTCCTCCAATGTTAAGTATACCCCTTTTAAGAGAACTAATTATTCAGCTGCCCTTTATTTGG
GTTGTGAGTAATGATACCTTAGGTAAGAATCAGAAAGAAATAAATTGCTCTGCTTAGAATTGTTTTTATGCATTATGCTGGGATGCTATTAAGCACCCTTTTGCTTTGGTAATGCGCATGCCTCGATTTGTTCCAGTTCCTGTTGAGACGCCAAGTAATTTGAGTTTGTTTAGGGAAAAGCGAGATTTTTGGGATCTCTGCTATTATTGTGGGCCTGATAGCCACC
GCCGCGGTTACTGCTTCGGTTACCGCCTCGGCCCTTGCACTTTCCGCCACTGTGCAGACAACTCAAACCATTAATGAACTATAGGCCACTGTGACCATGGTCCTAGATAAACAGGCCACTGCCAATTCTCAGATACAAGGAGGCCTGATGTTGGTAAACCAACAGATTAATTTGGTTCAGGAACAATTGGATATATTGTGGCAGATGGCCCAATTGGGATGTGAAC
AAAAGTTGCCTGGCCTTTGTGTCACTTCAGTACAATATGAGAATTTTACAAGAGCAGCTAACCTGTCACAAGCTCTGTCTCAACATTTATTACAGAATTGGACTTTTGAGTTTGAGCAGACACTCCGTGAGTTGAGAATGGCGATCCTGCAAGTGAATTCGACGCATCTGGATCTTTCTCTGATGGAAGGCCTCTCCTCCTGGATTTCATCTGCATTTTCTTATTT
TAAGGAATGGGTGGGGGTAGGATCATTTAGCCTAGTTCTCTGCTGTGGAATAGTGTTGATGCTTTGGATGGTGTGTAAGCTCAGAGCCCAAACAAAAGAGACAAAGTGGTGGTGGCACAAGCACTTGCTGCCTTAGAACACGGAGCCTCCCCTGACATTTGGTTAACCATGTTAAAGCAATAGGTCGCTGGCCTCCCAGCTCTTGCACCCCACGAGGCTAGTCTCA
TTGCACGGGATAGAGTGGGTAGGCTTCAGCAGCCCAAGGAGTTGCAAGGCTAAGCACTGCACAGGAGAGGTCTGCGGTATAACGACTTTCTCCTGGGAGATAAGTCATCTTGCATGAAGGTTCTATGTCATGATCTCCTTCCCCCAGAAAAACGACATCGGACTGGTCAGGGCTCTCTCTGGGGATAAGACCCTGGAGAGGAGCTTTGTACATGTTCCGAATTTGC
ACATGGGGATTTGACCTCTATCTCCACTCCAAAGTTGTGGGTGTCCTATTGCTTCAAAAATAAATGAATAGGGGGAGATGTGAGGAGCCGCCCTCGCTATCGCTATTGCCGTTAGAAGATGGCGCTGACATCTGCTGTCAGTTGGAGTTAACCGTTAGAAGATGGCGCTGACATCTGCTGTCAGTTGGAGTTAACCGTTTAAGCTGTGCCTCTCCCGTGGCGTCAT
CTGGGGTGATGTGCAAACCACCAATCCCGGCTCTACACGTCTCACTCGGAGCTCCTAGGCTTATATAAGGGGCTGGGTTTCTTAGCTTGGGGTCTCCCTCTAAGAAGCTGATCATCTATCTCTCAAGATGCATTAAAGCTTTACTGCAGAAGGATCCGAGTGTTCTGCGTCGTTCTTGCTGGCGAGACGGTAGCGCGGGACA

>IAPLTR4_I.1125009
TGTGAGGAGCCGCCCTCGCTATCGCTATCACCATTAGAAGATGGCGCTGACATCCGCTGTCAGTTGGAGTTCACCGTTTAAGCTGTGCCTCTCCCGTGGCGTCATCTGGGGTGATGAGCAAACCACCAATCCCGGCTCTACACGTCTCACTCGGAGCTCCTAGGCTTATATAAGGGGCTGGGTTTCTTAGCTTGGGGTCTCCCTCTCAGAAGCTGATCATCTATCT
CTCAAGATGCATTAAAGCTTTACTGCAGAAGGATCCGAGTGTCCTGCGTCGTTCTTGCTGGCGAGACGGTAGCGCGGGACATCTGGTGCCGAAACCCGGGAACTCTTCAACATCGCCGGCACCGCAGGAGACCCCTCGGAAGACGGGGTGGATTCAGAACTGCAGGGACGTAAGTTCAGAGAGGTATGCTTGATCCTGAACACCCCTTTTCTCGACTTTGGCCTTA
GTTTGTCATCACCGTGGGACGGGTCAGTGGAGGCTTTGGTGCTAGTCCTGTTTGTCCTTCTTCATTGTGGCTGGTGCTGACAGAACGGTGCTGTCAGTCTGGTAAAGGAGTCGAGCGCCATTTTTAGATAAGCCGCCAGCGCGATGGGGTTGTCACAGTCAGTGGTTACTGCCCTACAGACTGTGCTAAAACAAAGAGATCTCAAAATTGCACCACGAACAC
TTCAAAATTTTGTGAAGGAGGTAGATAGAGTGGCACCATGGTATGCTTGTTCCGGGTCCCTCACTGTGGCCTCATGGAACAAGCTAGGTAAAGAACTAGACAGGAAGCATGCAGAAGGAGATCTCCGCTTAGGTACCAAAGCCATTTGGAAGCTAGTTAAAAACTGCTTGGAGGATGAGGCCTGTCACCCTGCTATAGTAGAAAGTCAGGGAACCTTAGAGGAAGT
TCAGGATAGCATGTCAGAAACTGAACGGAGTGAGAGAATGGGAGCTCGCAAAAGGAAAGACATGTCTAAGAAAAAAGGCCCTCCCCAAGAAGTTAAGAAAGGGGGAGAGAAAGAAGGGAGTGATCATTCACCCCCTAACAAATCTAAGAAAAAGAAAAAGCCAGAATCTAGCGTATACCCTACGGTAGAGCTAGAAGCCTTGGAGCTAGATAATTCCGATTCTGAC
ACTCTGGATTCTAGCGAAGAAGGTGGTCTAGAGGAGGAGGTGGCACAGTATGAGGAAGAAAGGTATCACCCTGATAGACACCGGCTGCTGAAAACAAAAATGAATGTGAGGCCACCGCCTATAAATCCGGCGGGCTCACGCCCCTCGGCGCCTCCGCAGTATGAGCTTCGGCTGAATACTGGTACTGACTCCTTTTTACCTTTAGAGGAACGGAGAAAAATACAAA
TGGCTTTTCCAGTATTCGAGAATGCGGAAGGCGGAAGAGTACATACCCCGTAGATTACAATCAAATCAAAGAATTGGCCGAATCCGTTCGTAATTATGGGGTCAATGCTAACTTTACCACAATACAGGTGGAAAGGCTTGCTAATTTTGCCATGACTCCCACTGACTGGCAGATGACAGTGAAAGCAGTGCTTCCCAACATGGGACAATACATGGAATGGAAGGC
TCTCTGGTATGATGCAGCCCAGGCGCAAGCTAGAGTTAATGCCACAGCTGAAGATGAGAATCAGAGGCAGTGGACATTTGAAATGCTGACAAGTCAGGGTCAACATGCCCTTAATCAAACCAACTACATCTGGGGCGTCTATGGTCAGGTGTCGGCCGCCGCTATTAAAGCATGGAAGGCCCTCACAAAAAGGGGAGAATCCAGTGGACACCTTACAAAGATTGTC
CAAGGACCCCAAGAGCCATTTTCAGACTTTGTGGCCAGAATGACCGAGGCTGCCGGTCGCATCTTTGGTGATTCAGAACAAGCTATGCCCCTGATTGAGCAACTTGTCTATGAACAAGCAACCCAGGAATGTCGAGCGGCTATCGCCCCTCGCAAAAGCAAAGGTTTACAAGATTGGCTCAGAGTGTGCAGGGAGCTGGGGGGACCCCTAACCAATGCAGGACTAG
CTGCCGCCATTTTACAAACGCACAGGCGTAGAGATTTATCTAATCGAAAAGCTTGTTTTAATTGTGGGAAAATGGGGCACCTTAAGAAAGATTGTCAAGCCCCTGAAAGAACAAGAGAACTGGAGCTTTGCCGTCATTGTGGTAAAGGTTATCATAGGGCTAGTGAATGCAGGTCAGTCCGGGATATGAAAGGGAGGCTTCTGCCCCCTATTGAAGAACCCAAGGT
ACCCCAATCAAAAAACGGGTCACGGGGCCCATGGTCCCAGGGCCCTCAGAAATATGGGAACCAATTTCAGAAAAGCAATCAAGAGGAAGTAGAGGAACCCCCGGAATGGACTTGCGTGCCGCCTCCGACTTCTTACTCATGCCCCAAATGAATGTTCAACCAGTCCCAGTCCATTCCCCTGGACCTTTGCCCCCTGCCACCATTGGGCTTATTCTGGGCCGTGGCT
CCTTAACTTTACAGGGACTCATTATATATCCTGGGATTGTTGACCCGTATCATAAAGAGGAGAGTCAAGTTCTCTGTTCTAGCCCTCGGGGTGTATTTTCTATAAAGCAGGGAGATAGAATTGCACAACTGGTGTTGCTGCCCTCCCTCGGGGATGGAGAAACCTACACTTTACAAAAAAGGGCTATGGAATCTTCTGGAAGTGATTCAGCCTATCTGGCTATACC

[illegible]

>IAPLTR3.1125224

[illegible]

>IAPLTR4_I.1022916

[illegible]

>IAPLTR4_I.1289680

[illegible]

>IAPLTR3.3823185

[illegible]

ACCAGCATATACCTCTCAAAAATTCCGACAGTTTTGTCGGCAAATGGATGTTACCCATCTAACTGGCCTACCTTATAACCCTCAAGGACAGGGCATTGTGGAACGTGCCCATCGTACACTCAAATCATATCTGATTAAACAAAAGGGGAGTATTGAGGATGTTCTACCCTCGGTACCAAGAGTTGCTGTATCCATGGCACTTTTTACCCTTAATTTCTTGAACTCT
GATGCCCAAGGCCACACTGCGGCCGATCGCCACAGTTTAGAGCCTGATAGGCCAAAAGAAATGGTAAAATGGAAAGATGTCTTGACTGATCTCTGGAAAGGCCCGGATCCTATTCTTATAAGATCCAGGGGAGCTGTGTGTGTTTTTCCACAGGAGGAAGAAAACCCCCTGTGGATCCCTGAAAGACTCACTTGAAGAGCTCCTTCACAACTCCAGAATAAGAAGG
ATGGAGGCCAAGTGGGTGATGAAGACTCTCCTACTCGGGATGATGGTTCCTAGTATGATGGGAGAGTTACGGTGGGCCATCATGTCTACCTTCCCCCTACCGATGCCAGTGATGCAGAATGCCCAGGTGTTCCCTAGATTTTTCACAACTAATAGAGAGTTGGGGCTTGCCTTTTTGCCTTTAGATCCTCAGATACAGAGTTTTCAGGATGAGAGAACCTTGTCAT
TAGTAGGAAGCTTGTGCTTTACTGTGATTTCTTCATTAAAGGATAATTTTATTCATATGGATCCAGAAAGATCTAATGCTAATCCTTTTGAATTAATGGAAGCTGTACTGATCTTTCTCCTATGATAGTGAGAAGGGACAGATTACTTTTGATTGGAAAGGGGCATTAGGCAACAGTCTGAGTGAGGGACAGAGCTATGGATCATCTGATTGGTCCTCCTCCAATG
TTAAGTATACCCCTTTTAAGAGAACTAATTATTCAGCTGCCCTTTATTTGGGTTGTGAGTAATGATACCTTAGGTAAGAATCAGAAAGAAATAAATTGCTCTGCTTAGAATTGTTTTTATGCATTATGCTGGGATGCTATTAAGCACCCTTTTGCTTTGGTAATGCGCATGCCTCGATTTGTTCCAGTTCCTGTTGAGACGCCAAGTAATTTGAGTTTGTTTAGGG
AAAAGCGAGATTTTTGGGATCTCTGCTATTATTGTGGGCCTGATAGCCACCGCCGCGGTTACTGCTTCAGTTACCGCCTCGGCCCTTGCACTTTCTGCCACTGTGCAGACAACTCAAACCATTAATGAACTATAGGCCACTGTGACCATGGTCCTAGATAAACAGGCCACTGCCAATTCTCAGATACAAGGAGGCCTGATGTTGGTAAACCAACAGATTAATTTGG
TTCAGGAACAATTGGATATATTGTGGCAGATGGCCCAATTGGGATGTGAACAAAAGTTGCCTGGCCTTTGTGTCACTTCAGTACAATATGAGAATTTTACAAGAGCAGCTAACCTGTCACAAGCTCTGTCTCAACATTTATTACAGAATTGGACTTTTGAGTTTGAGCAGACACTCCGTGAGTTGAGAATGGCGATCCTGCAAGTGAATTCGACGCATCTGGATCT
TTCTCTGATGGAAGGCCTCTCCTCCTGGATTTCATCTGCATTTTCTTATTTTAAGGAATGGGTGGGGGTAGGATCATTTAGCCTAGTTCTCTGCTGTGGAATAGTGTTGATGCTTTGGATGGTGTGTAAGCTCAGAGCCCAAACAAAAGAGACAAAGTGGTGGTGGCACAAGCACTTGCTGCCTTAGAACACGGAGCCTTCCCTGACATTTGGTTAACCATGTTAA
AGCAATAGGTCGCTGGCCTCCCGGCTCTTGCACCCCACGAGGCTAGTCTCATTGCACGGGATAGAGTGGGTAGGCTTCAGCAGCCCAAGGAGTTGCAAGGCTAAGCACTGCACAGGAGAGGTCTGCGGTATAACGACTTTCTCCTGGGAGATAAGTCATCTTGCATGAAGGTTCTATGTCATGATCTCCTTCCCCCAGAAAAACGACATCGGACTGGTCAGGGCTC
TCTCTGGGGATAAGACCCTGGAGAGGAGCTTTGTACATGTTCCGAATTTGCACATGGGGATTTGACCTCTATCTCCACTCCAAAGTTGTGGGTGTCCTATTGCTTCAAAAATAAATGAATAGGGGGAGATGTGAGGAGCCGCCCTCGCTATCGCTATTGCCGTTAGAAGATGGCGCTGACATCTGCTGTCAGTTGGAGTTAACCGTTAGAAGATGGCGCTGACATC
TGCTGTCAGTTGGAGTTAACCGTTTAAGCTGTGCCTCTCCCGTGGCGTCATCTGGGGTGATGTGCAAACCACCAATCCCGGCTCTACACGTCTCACTCGGAGCTCCTAGGCTTATATAAGGGGCTGGGTTTCTTAGCTTGGGGTCTCCCTCTAAGAAGCTGATCATCTATCTCTCAAGATGCATTAAAGCTTTACTGCAGAAGGATCCGAGTGTTCTGCGTCGTTC
TTGCTGGCGAGACAGTAGCGCGG

>IAPLTR3.3906348
TGTGAGGAGCCGCCCTCGCTATCGCTATTGCCGTTAGAAGATGGCGCTGACATCTGCTGTCAGTTGGAGTTAACCGTTAGAAGATGGCGCTGACATCTGCTGTCAGTTGGAGTTAACCGTTTAAGCTGTGCCTCTCCCGTGGCGTCATCTGGGGTGATGTGCAAACCACCAATCCCGGCTCTACACGTCTCACTCGGAGCTCCTAGGCTTATATAAGGGGCTGGGT
TTCTTAGTTTGGGGTCTCCCTCTAAGAAGCTGATCATCTATCTCTCAAGATGCATTAAAGCTTTACTGCAGAAGGATCCGAGTGTTCTGCGTCGGCTGGCGAGACGGTAGCGCGGGACATCTGGTGCCGAAACCCGGGAACTCTTCAACATCGCCGGCGCCGCGGGAGACCCCTCGGAAGACGGGGCGGATTCAGAACTGCAGGGACGTAAGTTCAGAGAGGTATG
CTTTATCCTGAATACCCCCTTTCTCGACTTTGGCCTTAGTTTGTCATCACCGTGGGACGGGTCAGTGGAGGCTTTGGTGCTAGTCCTGTTTGTCCTTCTCCTTCATCGTGGCTGGTGCTGACAGAACGGTGCTGTCAGTCTGGTGCTGTCAGTCTGGTAACGGAGTTGAGCGCCGTTTTTTAGATAAGCCGCTAGCGCGATGGGGTTGTCACAGTCAGTGGTTACT
GCCCTACAGACTGTGCTAAAACAAAGAGATCTTAAAATTGCACCACGAACACTTCAAAATTTTGTGAAGGAGGTAGATAGAGTGGCACCATGGTATGCTTGTTCCGGGTCCCTCACTGTGGCCTCATGGAACAAGCTAGGTAAAGAACTAGACAGGAAGCATGCAGAAGGAGATCTCCACTTAGGTACCAAAGCCATTTGGAAGCTAGTTAAAAACTGCTTGGAGG
ATGAGGCCTGTCACCCTGCTATAATAGAAAGTCAGGGAACCTTAGAGGAAGTTCAGGATAGCATGTCAGAAACTGAACGGAGTGAGAGAATGGGAGCTCGCAAAAGGAAAGACATGTCTAAGAAAAAAGGCCCTCCCCAAGAAGTTAAGAAAGGGGGAGAGAGAGAAGGGAGTGATCATTCACCCCCTAACAAATCTAAGAAAAAGAAAAAGCCAGAATCTAGTCT
ATACCCTACAGTAGAGCTAGAAGCCTTGGAGCTAGATAATTCTGATTCTGACACTCTGTATTCTAGCGAAGAAGGTGGTCTAGAGGAGGAGGTGGCACGGTATGAGGAAGAAAGATATCACCCTGATAGACACCGGCCACTGAAAACAAAAATGAATGTGAGACCACCACCTATAAATCCGGCGGGCTCACGCCCCTCAGCGCCTCCGCAGTATGAGCTTCGGCTG
AATACTGGTACTGACTCCTTTTTACCTTTAGAGGAACGGAGAAAAATACAAGTAGCTTTACCAGTATTCGAGAATGTGGAAGGCGGAAGAGTACATACCCCTGTAGATTACAATCAAATCAAAGAATTGGCCGAATCCGTTCGTAATTATGGGGTCAATGCTAACTTTACCACATTACAGGTGGAAAGGCTTGCTAATTTTGCCATGACTCCCACTGACTGGCAGA
CGACAGTGAAAGCAGTGCTTCCCAACATGGGACAATACATGGAATGGAAGGCTCTCTGGTATGATGCAGCCCAGGCACAAGCTAGAGTTAATGCCACGGCTGAAGATGAGAATCAGAGGCAGTGGACATTTGAAATGCTGACAGGTCAGGGTCAACATGCCCTTAATCAAACCAACTACATCTGGGGTGTCTATGGTCAGGTGTCGGCCGCCGCTATTAAAGCATG
GAAGGCCCTCACAAAAAGGGGAGAGTCCAGTGGACACCTTACAAAGATTGTCCAAGGACCCCAAGAGCCATTTTCAGACTTTGTGGCCAGAATAACAGAGGCCGCCAGTCGCATCTTTGGTGATTCAGAACAAGCTATGCCCCTGATTGAGCAACTTGTCTATGAACAAGCAACCCAGGAATGTCGAGCGGCTATCGCCCCTCGCAAAAGCAAAGGTTTACAAGAT
TGGCTCAGAGTGTGCAGGGAGCTGGGGGGACCCCTAACCAATGCAGGACTAGCTGCCGCCATTTTACAAACGCACAGGCGTAGAGATTTATCTAGTCGAAAAGCTTGTTTTAATTGTGGGAGAATGGGGCACCTTAAGAAAGATTGTCAAGCCCCTGAAAGAACAAGAGAATCGAAGCTTTGCCATCGTTGTGGTAAAGGTTATCATAGGGCTAGCGAATGCAGGT
CAGTCCGGGATGTAAAAGGGAGGCTTCTGCCCCCTATTGAAGAACCCAAGGTACCCCAATCAAAAAACGGGTCACGGGGCCCGTGGTCCCAGGGCCCTCAGAAATATGGGAACCAATTTCAGAAAAGCAATCAAGAGGAAGTAGAGGTTTCCGAGGAACCCCCGGAATGGACTTGCGTGCTGCCTCCGACTTCTTACTCATGCCCCAAATGAATGTTCAACCGGTC
CCAGTCCATTCCCCTGGACCTTTGCCCCCTGCCACCATTGGGCTTATTCTGGGCCGTGGCTCCTTAACTTTACAGGGACTCATTATATATCCTGGAATTGTTGACCCGTATCATAAAGAGGAGATTCAAGTTCTCTGTTCTAGCCCTCGGGGTGTATTTTCTATAAAGCAGGGAGATAGAATTGCACAACTGGTGTTGCTGCCCTCCCTCGGGGATGGAGAAACCT
ACACTTTACAAAAAAGGGCTATGGGATCTTCTGGAAGTGATTCAGCCTATCTGGCTATACCCCTAAATGAGAGACCTACAATAAAATTGTTAATTAATGGAAAAGAATTTGAAGGGATAATGGATACGGGAGCAGACAAAAGCATTATTTCACTCCATTGGTGGCCGAAGTCTTGGCCCACTGTTGTTTCATCTCATTCTCTACAAGGTCTTGGATACCAGTTCTC
TCCTGCCATCAGTGCCTCAGCCTTAACCTGGTGGAACGCTGAGGGCAAACAGGGATGTTTTACCCCCTATGTGTTGCCACTCCCTGTAAATTTATGGGGACGGGATGTGTTACAAGCCATGGGCATGACCCTAACTAATAAATACTCCCCCCAGGCATCAGCCATTATGGCAAAGATGGGGTATACAAAGGGTAGAGGCTTGGGTAGGCAAGAACAAGGCAGAATA
AAACCCATTACGCAACACGGAAATCGGGGTAGAAAAGGACTGGGTTTTATTTAGGGGCCGTTGAGGCTTCACGACCCATACCGTGGAAAACAGAGGAGCCGTTATGGGTCTCTCAATGGCCTCTATCCTCTGAAAAATTAGAGGCTGTCACAAGATTAGTGCAAGAACAGGAACGGCTGGGGCATCTAGAGCCTTCTACCTCCCCATGGAATACACCAATTTTTGT
TATTAAGAAGAAATCTGGGAAGTGGAGATTACTCCATGACCTGCGGGCCATTAACAATCAGATGCATCTTTTTGGCCCTGTTCAAAGAGGCCTTCCTTTGCTTTCTGCACTTCCTCAAGATTGGAAGCTTATTATTATAGATATTAAGGATTGTTTCTTCTCTATTCCACTTTACCCACGGGATAGACCAAGGTTTGCCTTCACTATCCCTTCTCTTAATCATATG
GGACCAGACAAGAGATTTCAGTGGAAGGTACTGCCGCAAGGCATGGCCAATAGCCCAACAATATGTCAGCTGTATGTGCAGGAGGCTTTGGAGCCAATTAGGATGCAATTTACATCTTTAATCGTTATCCATTATATGGACGATATTCTTATTTGTCACAAAGAGTTAGATGTTTTACAAAAGGCTTTCCCCATGCTGGTGGCTGAATTAAAACAGTGGGGATTAG
AGATTGCCTCAGAAAAGGTTCAAATTGCAGATACAGGCCTCTTTTTGGGCTCAATGATTACTCCCAAAAATATAGTTCCTCAGAAAATAGAAATTCGCAAAGACCACTTACAAACATTAAATGATTTTCAGAAACTTCTGGGAGATATAAATTGGTTGAGGCCCTTTTTAAAAATTCCATCTGCTGATCTAAAACCCCTCTTCGATCTCCTGGAGGGTGAGCCTCA
TATCTCCTCACCCAGAAAATTTACCCCTGCCGCCCACCGAGCCTTACAGATGGTGGAAGAGGCCTTACAGGAAGCACAATTACAACGTATTGAGGAGTCACTACCTTTTAATCTGTGTGTTTTTAAGACAGCTCAATTGCCAACTGCAGTCCTGTGGCAACATGGACCATTACTTTGGATTCATCCAAATGCTTCCCCTGCTAAGATTATTAACTGGTACCCTGAT
GCTGTTGCACAGCTCGCGCTTCGCGGAATAAAAGCAGCTGTTACGCATTTTGGCAGGGATCCTGATTCCTTGATTGTGCCTTATACTGCTGCACAAGTTCAGACCTTGGCAGCCACATCTAGTGATTGGGCAGTTTTAGTTACTTCCTTTTCAGGACAAATTGATAATCATTTTCCAAAGCATCCAATTTTACAGTTTGCCCTAAACCAGGCTATAGTGTTTCCAC
AAGTGACAGCTAAAGACCCACTCCCAGATGGGACTGTGGTGTACACTGATGGATCAAAAACTGGTATGGGAGCTTATGTGGTCAAGGATAGGGTTATATCTAAACAATATAATGAAACTTCACCTCAAGTTGTAGAATGTCTGATAGTACTGGAGGTTCTTGAGGCTTTTCCAGGACCCCTCAATATTGTATCAGATTCTTCTTATGTGGTTAATGCAGTTAATCT
CCTTGAGATAGCTGGAATAATAAGATCCTCCAGCAGAGTTGCTAACATCTTTCAAAAGATACAAGCTGCCTTGTTAAATAGAAGATTTCCTGTTTTCATTACCCATGTCCGAGCACATTCTGGACTCCCAGGTCCCATGTCCTTAGGGAATGACTTAGCAGACAAGGCTACAAAGCTGGTGGCCGCTGCCTTGTCTACCCATGCACAAGCAGCAAAAGAATTCCAT
AAGCGCTTTCATGTGACGGCTGAGACCTTGCGCCGTCGTTTTGCTCTATCCAGGAAAGAGGCTAGGGAAATTGTTACTCAATGCCAGAATTGCTGCGAATTCTTACCTACTCCCCACATGGGAATAAACCCACGTGGCATTAGACCATTGCAGATGTGGCAGATGGATGTTACTCATATTCCCTCTTTTGGAAGGCTTCAATATGTTCATGTTTCTGTTGACACCT
GTTCTGGCGTAATGTTCGCCACACCCCTAACAGGGGAAAAGGCCTCATATGTGATTCAACATTGCCTTGAGGCCTGGAGTGCTTGGGGAAAACCCAGAATTTTAAAAACAGACAATGGACCAGCATATACCTCTCAAAAATTCCGACAGTTTTGTCGGCAAATGGATGTTACCCATCTAACTGGCCTACCTTATAACCCTCAAGGACAGGGCATTGTGGAACGTGC
CCATCGTACACTCAAATCATATCTGATTAAACAAAAGGGGAGTATTGAGGATGTTCTACCCTCGGTACCAAGAGTTGCTGTATCCATGGCACTTTTTACCCTTAATTTCTTGAACTCTGATGCCCAAGGCCACACTGCGGCCGATCGCCACAGTTTAGAGCCTGATAGGCCAAAAGAAATGGTAAAATGGAAAGATGTCTTGACTGATCTCTGGAAAGGCCCGGAT
CCTATTCTTATAAGATCCAGGGGAGCTGTGTGTGTTTTTCCACAGGAGGAAGAAAACCCCCTGTGGATCCCTGAAAGACTCACTTGAAGAGCTCCTTCACAACTCCAGAATAAGAAGGATGGAGGCCAAGTGGGTGATGAAGACTCTCCTACTCGGGATGATGGTTCCTAGTATGATGGGAGAGTTACGGTGGGCCATCATGTCTACCTTCCCCCTACCGATGCCA
GTGATGCAGAATGCCCAGGTGTTCCCTAGATTTTTCACAACTAATAGAGAGTTGGGGCTTGCCTTTTTGCCTTTAGATCCTCAGATACAGAGTTTTCAGGATGAGAGAACCTTGTCATTAGTAGGAAGCTTGTGCTTTACTGTGATTTCTTCATTAAAGGATAATTTTATTCATATGGATCCAGAAAGATCTAATGCTAATCCTTTTGAATTAATGGAAGCTGTAC
TGATCTTTCTCCTATGATAGTGAGAAGGGACAGATTACTTTTGATTGGAAAGGGGCATTAGGCAACAGTCTGAGTGAGGGACAGAGCTATGGATCATCTGATTGGTCCTCCTCCAATGTTAAGTAGAACTAATTATTCAGCTGCCCTTTATTTGGGTTGTGAGTAATGATACCTTAGGTAAGAATCAGAAAGAAATAAATTGCTCTGCTTAGAATTGTTTTTATGC
ATTATGCTGGGATGCTATTAAGCACCCTTTTGCTTTGGTAATGCGCATGCCTCGATTTGTTCCAGTTCCTGTTGAGACGCCAAGTAATTTGAGTTTGTTTAGGGAAAAGCGAGATTTTTGGGATCTCTGCTATTATTGTGGGCCTGATAGCCACCGCCGCGGTTACTGCTTCGGTTACCGCCTCGGCCCTTGCACTTTCTGCCACTGTGCAGACAACTCAAACCAT
TAATGAACTATAGGCCACTGTGACCATGGTCCTAGATAAACAGGCCACTGCCAATTCTCAGATACAAGGAGGCCTGATGTTGGTAAACCAACAGATTAATTTGGTTCAGGAACAATTGGATATATTGTGGCAGATGGCCCAATTGGGATGTGAACAAAAGTTGCCTGGCCTTTGTGTCACTTCAGTACAATATGAGAATTTTACAAGAGCAGCTAACCTGTCAAAA
GCTCTGTCTCAACATTTATTACAGAATTGGACTTTTGAGTTTGAGCAGACCCTCCGTGAGTTGAGAATGGCGATCCTGCAAGTGAATTCGACGCATCTGGATCTTTCTCTGATGGAAGGCCTCTCCTCCTGGATTTCATCTGCATTTTCTTATTTTAAGGAATGGGTGGGGGTAGGATCATTTAGCCTAGTTCTCTGCTGTGGAATAGTGTTGATGCTTTGGATGG
TGTGTAAGCTCAGAGCCCAAACAAAAGAGACAAAGTGGTGGTGGCACAAGCACTTGCTGCCTTAGAACACGGAGCCTCCCCTGACATTTGGTTAACCATGTTAAAGCAATAGGTCGCTGGCCTCCCGGCTCTTGCACCCCACGAGGCTAGTCTCATTGCACGGGATAGAGTGGGTAGGCTTCAGCAGCCCAAGGAGTTGCAAGGCTAAGCACTGCACAGGAGAGGT
CTGCGGTATAACGACTTTCTCCTGGGAGATAAGTCATCTTGCATGAAGGTTCTATGTCATGATCTCCTTCCCCCAGAAAAACGACATCGGACTGGTCAGGGCTCTCTCTGGGGATAAGACCCTGGAGAGGAGCTTTGTACATGTTCCGAATTTGCACATGGGGATTTGACCTCTATCTCCACTCCAAAGTTGTGCGTGTCCTATTGCTTCAAAAATAAATGAATAG
GGGGAGATGTGAGGAGCCGCCCTCGCTATCGCTATTGCCGTTAGAAGATGGCGCTGACATCTGCTGTCAGTTGGAGTTAACCGTTAGAAGATGGCGCTGACATCTGCTGTCAGTTGGAGTTAACCGTTTAAGCTGTGCCTCTCCCGTGGCGTCATCTGGGGTGATGTGCAAACCACCAATCCCGGCTCTACACGTCTCACTCGGAGCTCCTAGGCTTATATAAGGG
GCTGGGTTTCTTAGTTTGGGGTCTCCCTCTAAGAAGCTGATCATCTATCTCTCAAGATGCATTAAAGCTTTACTGCAGAAGGATCCGAGTGTTCTGCGTCGTTCTTGCTGGCGAGACGGTAGCGCGGGACA

>IAPLTR4_I.3748249
TGTGAGGAGCCGCCCTCGCTATCGCTATCGCCATTAGAAGATGGCGCTGACATCTGCTGTCAGTTGGAGTTTACCGTTTAAGCTGTGCCTCACCCGTCATCTGGGGTGATGAGCAAACCACCAATCCCGGCTCTACACGTCTCACTCGGAGCTCCTAGGCTTATATAAGGGGCTGGGTTTCTTAGCTTGGGGTCTCCCTTTCAGAAGCTGATCATCTATCTCTCAA
GATGTATTAAAGCTTTACTGCAGAAGGATCCGAGTGTCCTGTGTCGTTCTTGCTGGCAAGACGGTAGCGCGGGACATCTGGTGCCGAAACCCGGGAACTCTTCAACATCGCCGGCGCCGCGGGAGACCCCTTGGAAGACGGGGCGGATTCAGAACTGCAGGGACGTAAGTTCAGAGAGGTATGCTTTATCCTGAACACCCCTTTTCTCGACTTTGGCCTTAGTTTG
TCATCACAGTGGGACGGGTCAGTGGAGGCTTTGGTGCTAGTCCTGTTTGTCCTTCTTCATCGTGGCTGGTGCTGACAGAACGGTGCTGTCAGTCTGGTAAAGGAGTCGAGCGCTGTTTTTTAGATAAGCCGCCAGCGCGATGGGATTGTCACAGTCAGTGGTTACTGCCCTACAGACTGTGCTAAAACAAAGAGATCTCAAAATTGCACCACGAACACTTCAAAAT
TTTGTGAAGGAGGTAGATAGAGTGGCACCATGGTATGCTTGTTCCGGGTCCCTCACTGTGGCCTCATGGAACAAGCTAGGTAAAGAACTAGACAGGAAGCATGCAGAAGGAGATCTCCGCTTAGGTACCAAAGCCATTTGGAAGCTAGTTAAAAACTGCTTGGAGGATGAGGCCTGTCACCCTGCTATAGTAGAAAGTCAGGGAACCTTAGAGGAAGTTCAGGATA
GCATGTCAGAAACTGAATGGAGTGAGAGAATGGGAGCTCGCAAAAGGAAAGACATGTCTAAGAAAAAAGGCCCTCCCCAAGAAGTTAAGAAAGGGGGAGAGAAAGAAGGGAGTGATCATTCACCCCCTAACAAATCTAAGAAAAAGAAAAAGCCAGAATCTAGCCTATACCCTACGGTACAGCTAGAAGCCTTGGAGCTAGATAATTCCGATTCTGACACTCTGGA
TTCTAGCGAAGAAGGTGGTCTAGAGGAGGAGGTGGCACGGTATGAGGAAGAAAGATATCACCCTGATAGACACCGGCCGCTGAAAACAAAAATGAATGTGAGGCCACCGCCTATAAATCCAGCGGGCTCACGCCCCTCGGCGCCTCCGCAGTATGAGCTTCGGCTGAATACTGGTACTGACTCCTTTTTACCTTTAGAGGAACGGAGAAAAATACAAATGGCTTTT
CCAGTATTCGAGAATGTGGAAGGCGGAAGAGTACATACCCCCGTAGATTACAATCAAATCAAAGAATTGGCTGAATCCGTTCGTAATTATGGGGTCAATGCTAACTTTACCACATTACAGGTGGAAAGGCTTGCTAATTTTGCCATGACTCCCACTGACTGGCAGACGACAGTGAAAGCAGTGCTTCCCAACATGGGACAATACATGGAATGGAAGGCTCTCTGGT
ATGATGCAGCCCAGGCGCAAGCTAGAGTTAATGCCACAGCTGAAGATGAGAATCAGAGGCAGTGGACATTTGAAATGCTGACAGGTCAGGGTCAACATGCCCTTAATCAAACCAACTACATCTGGGGCGTCTATGGTCAGGTGTCGGCCGCCGCTATTAAAGCATGGAAGGCCCTCACAAAAAGGGGAGAATCCAGTGGACACCTTACAAAGATTGTCCAAGGACC
CCAAGAGCCATTTTCAGACTTTGTGGCCAGAATGACCGAGGCCGCCGGTCGCATCTTTGGTGATTCAGAACAAGCTATGCCCCTGATTGAGCAACTTGTCTATGAACAAGCAACCCAGGAATGTCGAGCGGCTATCGCCCCTCGCAAAAGCAAAGGTTTACTAGATTGGCTCAGAGTGTGCAGGGAGCTGGGGGGACCCCTAACCAATGCAGGACTAGCTGCCGCC
ATTTTACAAACGCACAGGTGTAGAGATTTATCTAGTCGAAAAGCTTGTTTTAATTGTGGGAAAATGGGGCACCTTAAAAAAGATTGTCAAGCCCCTGAAAGAACAAGAGAACTGGAGCTTTGCCGTCGTTGTGGTAAAGGTTATCATAGGGCTAGTGAATGCAGGTCAGTCCGGGATATGAAAGGGAGGCTTCTGCCCCCTATTGAAGAACCCAAGGTACCCCAAT
CAAAAAACGGGTCATGGGGCCCATGGTCCCAGGGCCCTCAGAAATATGGGAACCAATTTCAGAAAAGCAATCAAGAGGAAGTAGAGGCTTCCGAGGAACCCCCGGAATGGACTTGCATGCCGCCTCCGACTTCTTACTCATGCCCCAAATGAATGTTCAACCGGTCCCAGTCCATTCCCCTGGACCTTTGCCCCCTGCCACCATTGGGCTTATTCTGGGCCGTGGC
TCCTTAACTTTACAGGGTCTCATTATATATCCTGGGATTGTTGACCCGTATCATAAAGATGAGATTCAAGTTCTCTGTTCTAGCCCTCGGGGTGTATTTTCTATAAAGCAGGGAGATAGAATTGCACAACTGGTGTTGCTGCCCTCCCTTGGGGATGGAGAAACCTACACTTTACAAAAAGGGCTATGGGATCTTCTGGAAGTGATTCAGCCTATCTGGCTATAC
CCCTAAATGAGAGACCTACAATAAAATTGTTAATTAATGGAAAAGAATTTGAAGGGATAATGGATACGGGAGCAGACAAAAGCATTATTTCACTCCATTGGTGGCCGAAGTCTTGGCCCACTGTTGTTTCATCTCATTCTCTACAAGGTCTTGGATACCAGTCCTCTCCTGCCATCAGTGCCTCAGCCTTAACCTGGCGGAACGCTGAGGGCAAACAGGGATTTTT
TACCCCCTATGTGTTGCCACTCCCTGTAAATTTATGGGGACGGGATGTGTTACAAGCCATGGGCATGACCCTAACTAATGAATACTCCCCCCAGGCATCAGCCATTATGGCAAAGATGGGGTATACAAAGGGTAGAGGCTTGGGTAGGCAAAAACAAGGCAGAATAAAACCCATTACACAACATGGAAATCGGGGTAGAAAAGGACTGGGTTTTATTTAGGGGCCG
TTGAGGCTTCATGACCCATACCATGGAAAACAGAGGAGCCGTTATGGGTCTCTCAATGGCCTCTATCCTCTGAAAAATTAGAGGCTGTCACAAGATTAGTGCAAGAACAGGAATGGCTGGGGCATCTAGAGCCTTCTACCTCCCCATGGAATACACCAATTTTTGTTATTAAGAAGAAATCTGGGAAGTGGAGATTACTCCACGACCTGCGGGCCATTAACAATCA
GATGCATCTTTTTGGCCCTGTTCAAAGAGGACTTCCTTTGCTTTCTGCACTTCCTCAAGATTGGAAGCTTATTATTATAGATATTAAGGATTGTTTCTTCTCTATTCCACTTTACCCACGGGATAGACCAAGGTTTGCCTTCATTATCCCTTCTCTTAATCATATGGAACCAGACAAGAGATTTCAGTGGAAGGTACTGCCACAAGGCATGGCCAATAGCCCAACA
ATATGTCAGCTGTATGTGCAGGAGGCTTTGGAGCCAATTAGGAAGCAATTTACATCTTTAATCATGATCCATTATATGGACGATATTCTTATTTGTCACAAAGAGTTAGATGTTTTACAAAAGGCTTTTCCCATGCTGGTGGCTGAATTAAAACAGTGGGGATTAGAGATTACCTCAGAAAAGGTTCAAATTACAGATACAGGCCTCTTTTTGGGCTCAATGATTA
CTCCCAAAAATATAGTTCCTCAGAAAATAGAAATTCGCAAAGACCACTTACAAACATTAAATGATTTTCAGAAACTTCTGGGAGATATAAATTGGTTGAGGCCCTTTTTAAAAATTCCATCTGCTGATCTACAACCCCTCTTCGATCTCTTGGAGGGTGAGCCTCATATCTCCTCACCCAGAAAATTTACCCCTGCCGCCCACCGAGCCTTACAGATGGTGGAAGA
GGCCTTACAGGAAGCACAATTACAACGTATTGAGGAGTCACTACCTTTTAATCTGTGTGTTTTTAAGACAGCTCAATTGCCAACTGCAGTTCTGTGGCAACATGGACCATTACTTTGGATTCATCCAAAAGCTTCCCCTGCTAAGATTATTGACTGGTACCCTGATGCTGTTGCACAGCTCGCGCTTCGCGGAATAAAAGCAGCTGTTACGCATTTTGGCAGGGAT
CCTGATTCCTTGATTGTGCCTTATACTGCTGCACAAGTTCAGACCTTGGCAGCCACATCTAGTGATTGGGCAGTTTTAGTTACTTCCTTTTCAGGACAAATTGATAATCATTTTCCAAAACATCCAATTTTACAGTTTGCCCTAAACCAGGCTATAGTGTTTCCACAAGTGACAGCTAAAGACCCACTCCCAGATGGGACTGTGGTGTACACTGATGGATCAAAAA
CTGGTATGGGAGCTTATGTGGTCAAGGATAGGGTTATATCTAAACAATATAATGAAACTTCACCTCAAGTTGTAGAATGTCTGATAGTACTGGAGGTTCTTGAGGCTTTTCCAGGACCCCTCAATATTGTATCAGATTCCTCTTATGTGGTTAATACAGTTAATCTCCTTGAGATAGCTGGAATAATAAGATTCTCCAGCAGAGTTGCTAACATCTTTCAGAAGAT
ACAAGCTGCCTTGTTAAATAGGAGATTTCCTGTTTTCATTACCCATGTCCGAACACATTCTGGACTCCCAGGTCCCATGTCCTTAGGGAATGACTTAGCGGACAAGTCTACAAAGCTGGTGGCCGCTGCCTTGTCTACCCACGCACAAGCAGCAAAAGAGTTCCATCAGCGCTTTCATGTGACGGCTGAGACCTTGCGCCGTCGTTTTGCTCTATCTAGGAAAGAG
GCTAGGGAAATTGTTACTCAATGCCAGAATTGCTGCGAATTCTTACCTGCTCCCCACGTGGGAATAAACCCACGTGGCATTAGACCATTGCAGATGTGGCAGATGGATGTTACTCATATTCCCTCTTTTGGAAGGCTTCAATATGTTCATGTTTCTGTTGACACCTGTTCTGGCGTAATGTTTGCCACGCCCCTAACAGGGGAAAAGGCCTCATATGTGATTCAAC
ATTGCCTTGAGGCCTGGAGTGCTTGGGGAAAACCCAGAATTTTAAAAACAGACAATGGACCAGCATATACCTCTCAAAAATTCCGACAGTTTTGTCGGCAAATGGATGTTACCCATCTAACTGGCCTACCTTATAACCCTCAAGGACAGGGCATCGTGGAATGTGCCCATCGTACACTCAAATCATATCTGATTAAACAAAAGGGGAGTATTGAGGATGTTCTACC
CTCGGTACCAAGAGTTGCTGTGTCCATGGCAGTTTTTACCCTTAATTTCTTGAACTCTGATGCCCAAGGGCACACTGCGGCCGATCGCCACAGTTTAGAGCCTGATAGGCCAAAAGAAATGGTAAAATGGAAAGATGTCTTGACTGATCTCTGGAAAGGCCCGGATCCTATTCTTATAAGATCCAGGGGAGCTGTGTGTTTTTCCACAGGAGGAAGAAAACCCCCT
GTGGATCCCTGAAAGACTCACTCGAAGAGCTCCTTCACAACTCCAGAATAAGAAGGATGGAGGCCAAGTGGGTGATGAAGACTCTCCTACTTGGGATGATGGTTCCTAGTATGATGGGAGAGTTACGGTGGGCCATCATGTCTACCTTCCCCCTACCGATGCCAGTGATGCATAATGCCCAGGAGTTCCCTAGATTTTTCACAACTAATAGAGAGTTGGGGCTTGC
CTTTTTGCCTTTAGATCCTCAGATACAGAGTTTTCAGGATGAGAGAACCTTGTCATTGGTAGGAAACTTGTGCTTTACTGTGATTTCTTCATTAAAGGATAATTTACTCATATGGATCCAGAAAGATCTAATGCTAATCCTTTTGAATTAATGGAAGCTGTACTGATCTTTCTCCTATGGCAATGATTGCTGGAGGATATAGTGAGAAGGGACAGATTACTTTTG
ATTGGAAAGGGGCATTAGGCAACAGTCTGAGTGAGGGACAGAGCTCTGGATCATCTGATTGGTCCTCCTCCAATGTTAAGTATACCCCTTTTAAGAGAACTAATTATTCAGCTGCTCCAATTTGTGTTTGGCTGCCCTTTATTTGGGTTGTGAGTAATGATACCTTAGGTAAGAATCAGAAATAAATTGCTCTGCTTAGAATTGTTTTTATGCATTATGCTGGGAT
GCTATTAAGCACCCTTTTGCTTTGGTAATGCGCATGCCTCGATTTGTTCCAGTTCCTGTTGAGGTGCCAAGTAGTTTGAGTTTGTTTAGGGAAAAGCAAGATTTTTGGGATCTTTGCTATTATTGTGGGCCTGATAGCCACTGCCGCGGTTACTGCTTCGGTTACCGCCTTGGCCCTTGCACTTTCCGCCACTGTGCAGACAAATCAAACCATTAATGAACTATCG
GCCACTGTGACCATGGCCCTAGATAAACAGGCCACTGCCAATTCTCAGATACAAGGAGGCCTGATGTTGGTAAACCAACAGATTAATTTGGTTCAAGAACAATTGGATATATTGTGGCAGATGGCCCAATTGGAATGTGAACAAAAGTTGCCTGGCCTTTGTGTCACTTCAGTACAATATGAGAATTTTACAAGAGCAGCTAACCTGTCAAAAGCTCTGTCTCAAC
ATTTATTACAGAATTGGACTTTTGAGTTTGAGTAGACACTCCGTGAGTTGAGAGTGGCGATCCTGCAAGTGAATTCGACGCATCTGGATCTTTCTCTGATGGAAGGCCTCTCCTCCTGGATTTCATCTGCATTTTCTTATTTTAAGGAATGGGTGGGGGTGGGATCATTTAGCCTAGTTCTCTGCTGTGGAATAGTGTTGATGCTTTGGATGGTGTGTAAGCTCAG
AGCCCAAACAAAAGAGACAAAGTGATGGTGGCACAAGCACTTGCTGCCTCAGAACACGGAGCCTCCCCTGACATTTGGTTAACCATGTTAAAGCAATAGGTCGCTGGCCTCCCGGCTCTTGCACCCCACGAGGCTAGTCTCATTGCACGGGATAGAGTGGGTAGGCTTCAGCAGCCCAAGGAGTTGCAAGGCTAAGCACTGCACAGGAGAGGTCTCCTGGGAGATA
AGTCATCTTGCATGAAGGTTCTATGTCATGATCTCCTTCCCCCAGAAAAACGACATCGGACTGGTCAGGGCTCCCTCTGGGGATAAGACCCTGGAGAGGAGCTTTGTACATGTTCCAAATTTGCACATGGGGATTTGACCTCTATCTCCACTCCAAAGTTGTGGGTGTCCTATGGCTTCAAAAATAAATGAATAGGGGGAGATGTGAGGAGCCGCCCTCGCTATC
GCTATCGCCATTAGAAGATGGCGCTGACATCTGCTGTCAGTTGGAGTTTACCGTTTAAGCTGTGCCTCACCCGTCATCTGGGGTGATGAGCAAACCACCAATCCCGGCTCTACACGTCTCACTCGGAGCTCCTAGGCTTATATAAGGGGCTGGGTTTCTTAGCTTGGGGTCTCCCTTTCAGAAGCTGATCATCTATCTCTCAAGATGTATTAAAGCTTTACTGCAG
AAGGATCCGAGTGTCCTGCGTCGTTCTTGCTGGCGAGACGGTAGCGTGGGACA

>IAPLTR3.1202126
TGTGAGGAGCCGCCCTCGCTATCGCTATTGCCGTTAGAAGATGGCGCTGACATCTGCTGTCAGTTGGAGTTAACCGTTAGAAGATGGCGCTGACATCTGCTGTCAGTTGGAGTTAACCGTTTAAGCTGTGCCTCTCCCGTGGCGTCATCTGGGGTGATGTGCAAACCACCAATCCCGGCTCTACACGTCTCACTCGGAGCTCCTAGGCTTATATAAGGGGCTGGGT
TTCTTAGCTTGGGGTCTCCCTCTAAGAAGCTGATCATCTATCTCTCAAGATGCATTAAAGCTTTACTGCAGAAGGATCCGAGTGTTCTGCGTCGTTCTTGCTGGCGAGATGGTAGCGCGGGACATCTGGTGCCGAAACCCGGGAACTCTTCAACATCGCCGGCGCCGCGGGAGACCCCTCGGAAGACGGGGCGGATTCAGAACTGCAGGGACGTAAGTTCAGAGAG
GTATGCTTTATCCTGAACACCCCTTTTCTAGACTTTGGCCTTAGTTTGTCATCACCGTGGGACGGGTCAGTGGAGGCTTTGGTGCTAGTCCTGTTTGTCCTTCTCCTTCATCGTGGCTGGTGCTGACAGAACGGTGCTGTCAGTCTGGTGCTGTCAGTCTGGTGCTGTCAGTCTGGTGCTGTCAGTCTGGTAACAGAGTTGAGCGCCGTTTTTTAGATAAGCCGCT
AGCGCGATGGGGTTGTCACAGTCAGTGGTTACTGCCCTACAGACTGTGCTAAAACAAAGAGATCTTAAAATTGCACCACGAACACTTCAAAATTTTGTGAAGGAGGTAGATAGAGTGGCACCATGGTATGCTTGTTCCGGGTCCCTCACTGTGGCCTCATGGAACAAGCTAGGTAAAGAACTAGACAGGAAGCATGCAGAAGGAGATCTCCACTTAGGTACCAAAG
CCATTTGGAAGCTAGTTAAAAACTGCTTGGAGGATGAGGCCTGTCACCCTGCTATAATAGAAAGTCAGGGAACCTTAGAGGAAGTTCAGGATAGCATGTCAGAAACTGAACGGAGTGAGAGAATGGGAGCTCGCAAAAGGAAAGACATGTCTAAGAAAAAAGGCCCTCCCCAAGAAGTTAAGAAAGGGGGAGAGAAAGAAGGGAGTGATCATTCACCCCCTAACAA
ATCTAAGAAAAAGAAAAAGCCAGAATCTAGTCTATACCCTACAGTAGAGCTAGAAGCCTTGGAGCTAGATAATTCTGATTCTGACACTCTGTATTCTAGCGAAGAAGGTGGTCTAGAGGAGGAGGTGGCACGGTATGAGGAAGAAAGATATCACCCTGATAGACACCGGCCACTGAAAACAAAAATGAATGTGAGACCACCACCTATAAATCCGGCGGGCTCACGC
CCCTCAGCGCCTCCGCAGTATGAGCTTCGGCTGAATACTGGTACTGACTCCTTTTTACCTTTAGAGGAACGGAGAAAAATACAAGTAGCTTTTCCAGTATTCGAGAATGCGGAGGGCGGAAGAGTACATACCCCTGTAGATTACAATCAAATCAAAGAATTGGCCGAATCCGTTCGTAATTATGGGGTCAATGCTAACTTTACCACATTACAGGTGGAAAGGCTTG
CTAATTTTGCCATGACTCCCACTGACTGGCAGACGACAGTGAAAGCAGTGCTTCCCAACATGGGACAATACATGGAATGGAAGGCTCTCTGGTATGATGCAGCCCAGGCACAAGCTAGAGTTAATGCCACGGCTGAAGATGAGAATCAGAGGCAGTGGACATTTGAAATGCTGACAGGTCAGGGTCAACATGCCCTTAATCAAACCAACTACATCTGGGGTGTCTA
TGGTCAGGTGTCGGCCGCCGCTATTAAAGCATGGAAGGCCCTCACAAAAAGGGGAGAGTCCAGTGGACACCTTACAAAGATTGTCCAAGGACCCCAAGAGCCATTTTCAGACTTTGTGGCTAGAATGACAGAGGCCGCCAGTCGCATCTTTGGTGATTCAGAACAAGCTATGCCCCTGATTGAGCAACTTGTCTATGAACAAGCAACCCAGGAATGTCGAGCGGCT
ATCGCCCCTCGCAAAAGCAAAGGTTTACAAGATTGGCTCAGAGTGTGCAGGGAGCTGGGGGGACCCCTAACCAATGCAGGACTAGCTGCCGCCATTTTACAAACGCACAGGTATAGAGATTTATCTAGTCGAAAAGCTTGTTTTAATTGTGGGAAAATGGGGCACCTTAAGAAAGATTGTCAAGCCCCTGAAAGAACAAGAGAATCGAAGCTTTGCCATCGTTGTG
GTAAAGGTTATCATAGGGCTAGCGAATGCAGGTCAGTCCGGGATGTAAAAGGGAGGCTTCTGCCCCCTATTGAAGAACCCAATGTACCCCAATCAAAAAACGGGTCACGGGGCCCATGGTCCCAGGGCCCTCAGAAATATGGGAACCAATTTCAGAAAAGCAATCAAGAGGAAGTAGAGGCTTCCGAGGAACCCCCGGAATGGGCTTGCGTGCCGCCTCCGACTTC
TTACTCATGCCCCAAATGAATGTTCAACCGGTCCCAGTCCATTCCCCTGGACCTTTGCCCCCTGCCACCATTGGGCTTATTCTGGGCCGTGGCTCCTTAACTTTACAGGGACTCATTATATATCCTGGAATTGTTGACCCGTATCATAAAGAGGAGATTCAAGTTCTCTGTTCTAGCCCTCGGGGTGTATTTTCTATAAAGCAGGGAGATAGAATTGCACAACTGG
TGTTGCTGCCCTCCCTCGGGGATGGAGAAACCTACACTTTACAAAAAAGGGCTATGGGATCTTCTGGAAGTGATTCAGCCTATCTGGCTATACCCCTAAATGAGAGACCTACAATAAAATTGTTAATTAATGGAAAAGAATTTGAAGGGATAATGGATACGGGAGCAGACAAAAGCATTTTTCACTCCATTGGTGGCCGAAGTCTTGGCCCACTGTTGTTTCATCT
CATTCTCTACAAGGTCTTGGATACCAGTCCTCTCCTGCCATCAGTGCCTCAGCCTTAACCTGGCGGAATGCTGAGGGCAAACAGGGATGTTTTACCCCCTATGTGTTGCCACTCCCTGTAAATTTATGGGGACGGGATGTGTTACAAGCCATGGGCATGACCCTAACTAAAGAATACTCCCCCCAGGCATCAGCCATTATGGCAAAGATGTGGTATACAAACGGTA
GAGGCTTGGGTAGGCAAGAACAAGGCAGAATAACACCCATTACACAACACGGAAATCGGGGTAGAAAAGGACTGGGTTTTATTTAGGGGCCGTTGAGGCTTCACGACCCATACCATGGAAAACAGAGGAGCCGTTATGGGTCTCTCAATGGCCTCTATCCTCTGAAAAATTAGAGGCTGTCACAAGATTAGTGCAAGAACAGGAACGGCTGGGGCATCTAGAGCCT
TCTACCTCCCCATGGAATACACCAATTTTTGTTATTAAGAAGAAATCTGGGAAGTGGAGATTACTCCATGACCTGCGGGCCATTAACAATCAGATGCATCTTTTTGGCCCTGTTCAAAGAGGCCTTCCTTTGCTTTCTGCACTTCCTCAAGATTGGAAGCTTATTATTATAGATATTAAGGATTGTTTCTTCTCTATTCCACTTTACCCACGGGATAGACCAAGGT
TTGCCTTCACTATCCCTTCTCTTAATCATATGGAACCAGACAAGAGATTTCAGTGGAAGGTACTGCCGCAAGGCATGGCCAATAGCCCAACAATATGTCAGCTGTATGTGCAGGAGGCTTTGGAGCCAATTAGGAAGCAATTTACATCTTTAATCGTTATCCATTATATGGACGATATTCTTATTTGTCACAAAGAGTTAGATGTTTTACAAAAGGCTTTCCCCAT
GCTGGTGGCTGAATTAAAACAGTGGGGATTAGAGATTGCCTCAGAAAAGGTTCAAATTGCAGATACAGGCCTCTTTTTGGGCTCAATGATTACTCCCAAAAATATAGTTCCTCAGAAAATAGAAATTCGCAAAGACCACTTACAAACATTAAATGATTTTCAGAAACTTCTGGGAGATATAAATTGGTTGAGGCCCTTTTTAAAAATTCCATCTGCTGATCTAAAA
CCCCTCTTCGATCTCCTGGAGGGTGAGCCTCATATCTCCTCACCCAGAAAATTTACCCCTGCCGCCCACCGAGCCTTACAGATGGTGGAAGAGGCCTTACAGGAAGCACAATTACAACGTATTGAGGAGTCACTACCTTTTAATCTGTGTGTTTTTAAGACAGCTCAATTGCCAATTGCAGTCCTGTGGCAACATGGACCATTACTTTGGATTCATCCAAATGCTT
CCCCTGCTAAGATTATTCACTGGTACCCTGATGCTGTTGCACAGCTCGCGCTTCGCGGAATAAAAGCAGCTGTTACACATTTTGGCAGGGATCCTGATTCCTTGATTGTGCCTTATACTGCTGCACAAGTTCAGACCTTGGCAGCCACATCTAGTGATTGGGCAGTTTTAATTACTTCCTTTTCAGGACAAATTGATAATCATTTTCCAAAGCATCCAATTTTACA
GTTTGCCCTAAACCAGGCTATAGTGTTTCCACAAGTGACAGCTAAAGACCCACTCCCAGATGGGACTGTGGTGTACACTGATGGATCAAAAACTGGTATGGGAGCTTATGTGGTCAAGGATAGGGTTATATCTAAACAATATAATGAAACTTCACCTCAAGTTGTAGAATGTCTGATAGTACTGGAGGTTCTTGAGGCTTTTCCAGGACCCCTCAATATTGTATCA
GATTCCTCTTATGTGGTTAATGCAGTTAATCTCCTTGAGATAGCTGGAATAATAAGATCCTCCAGCAGAGTTGCTAACATCTTTCAAAAGATACAAGCTGCCTTGTTAAATAGGAGATTTCCTGTTTTCATTACCCATGTCCGAGCACATTCTGGACTCCCAGGTCCCATGTCCTTAGGGAATGACTTAGCGGACAAGGCTACAAAGCTGGTGGCCGCTGCCTTGT
CTACCCATGCACAAGCAGCAAAAGAATTCCATAAGCGCTTTCATGTGACGGCTGAGACCTTGCGCCGTCGTTTTGCTCTATCCAGGAAAGAGGCTAGGGAAATTGTTACTCAATGCCAGAATTGCTGCAAATTCTTACCTACTCCCCACATGGGAATAAACCCACGTGGCATTAGACCACTGCAGATGTGGCAGATGGATGTTACTCACATTCCCTCTTTTGGAAG
GCTTCAATATGTTCATGTTTCTGTTGACACCTGTTCTGGCGTAATGTTCGCCACACCCCTAACAGGGGAAAAGGCCTCATATGTGATTCAACATTGCCTTGAGGCCTGGAGTGCTTGGGGAAAACCCAGAATTTTAAAAACAGACAATGGACCAGCATATACCTCTCAAAAATTCCGACAGTTTTGTCGGCAAATGGATGTTACCCATCTAACTGGCCTACCTTAT
AACCCTCAAGGACAGGGCATTGTGGAACGTGCCCATCGTACACTCAAATCATATCTGATTAAACAAAAGGGGAGTATTGAGGATGTTCTACCCTCGGTACCAAGAGTTGCTGTATCCATGGCACTTTTTACCCTTAATTTCTTGAACTCTGATGCCCAAGGCCACACTGCGGCCGATCGCCACAGTTTAGAGCCTGATAGGCCAAAAGAAATGGCAAAATGGAAAG
ATGTCTTGACTGATCTCTGGAAAGGCCCGGATCCTATTCTTATAAGATCCAGGGGAGCTGTGTGTGTTTTTCCATAGGAGGAAGAAAACCCCCTGTGGATCCCTGAAAGACTCACTCGAAGAGCTCCTTCACAACTCCAGAATAAGAAGGATGGAGGCCAAGTGGGTGATGAAGACTCTCCTACTCGGGATGATGGTTCCTAGTATGATGGGAGAGTTACGGTGGG
CCATCATGTCTACCTTCCCCCTACCGATGCCAGTGATGCAGAATGCCCAGGTGTTCCCTAGATTTTTCACAACTAATAGAGAGTTGGGGCTTGCCTTTTTGCCTTTAGATCCTCAGATACAGAGTTTTCAGGATGAGAGAACCTTGTCATTGGTAGGAAGCTTGTGCTTTACTGTGATTTCTTCATTAAAGGATAATTTTATTCATATGGATCCAGAAAGATCTAA
TGCTAATCCTTTTGAATTAATGGAAGCTGTACTGATCTTTCTCCTATGATAGTGAGAAGGGACAGATTACTTTTGATTGGAAAGGGGCATTAGGCAACAGTCTGAGTGAGGGACAGAGCTATGGATCATCTGATTGGTCCTCCTCCAATGTTAAGTATACCCCTTTTAAGAGAACTAATTATTCAGCTGCCCTTTATTTGGGTTGTGAGTAATGATACCTTAGGTA
AGAATCAGAAAGAAATAAATTGCTCTGCTTAGAATTGTTTTTATGCATTATGCTGGGATGCTATTAAGCACCCTTTTGCTTTGGTAATGCGCATGCCTCGATTTGTTCCAGTTCCTGTTGAGACGCCAAGTAATTTGAGTTTGTTTAGGGAAAAGCGAGATTTTTGGGATCTCTGCTATTATTGTGGGCCTGATAGCCACCGCCGCGGTTACTGCTTCGGTTACCG
CCTCGGCCCTTGCACTTTCTGCCACTGTGCAGACAACTCAAACCATTAATGAACTATAGGCCACTGTGACCATGGTCCTAGATAAACAGGCCACTGCCAATTCTCAGATACAAGGAGGCCTGATGTTGGTAAACCAACAGATTAATTTGGTTCAGGAACAATTGGATATATTGTGGCAGATGGCCCAATTGGGATGTGAACAAAAGTTGCCTGGCCTTTGTGTCAC
TTCAGTACAATATGAGAATTTTACAAGAGCAGCTAACCTGTCACAAGCTCTGTCTCAACATTTATTACAGAATTGGACTTTTGAGTTTGAGCAGACACTCCGTGAGTTGAGAATGGCGATCCTGCAAGTGAATTCGACGCATCTGGATCTTTCTCTGATGGAAGGCCTCTCCTCCTGGATTTCATCTGCATTTTCTTATTTTAAGGAATGGGTGGGGGTAGGATCA
TTTAGCCTAGTTCTCTGCTGTGGAATAGTGTTGATGCTTTGGATGGTGTGTAAGCTCAGAGCCCAAACAAAAGAGACAAAGTGGTGGTGGCACAAGCACTTGCTGCCTTAGAACACGGAGCCTCCCCTGACATTTGGTTAACCATGTTAAAGCAATAGGTCGCTGGCCTCCCAGCTCTTGCACCCCACGAGGCTAGTCTCATTGCACGGGATAGAGTGGGTAGGCT
TCAGCAGCCCAAGGAGTTGCAAGGCTAAGCACTGCACAGGAGAGGTCTGCGGTATAACGACTTTCTCCTGGGAGATAAGTCATCTTGCATGAAGGTTCTATGTCATGATCTCCTTCCCCCAGAAAAACGACATCGGACTGGTCAGGGCTCTCTCTGGGGATAAGACCCTGGAGAGGAGCTTTGTACATGTTCCGAATTTGCACATGGGGATTTGACCTCTATCTCC
ACTCCAAAGTTGTGGGTGTCCTATTGCTTCAAAAATAAATGAATAGGGGGAGATGTGAGGAGCCGCCCTCGCTATCGCTATTGCCGTTAGAAGATGGCGCTGACATCTGCTGTCAGTTGGAGTTAACCGTTAGAAGATGGCGCTGACATCTGCTGTCAGTTGGAGTTAACCGTTTAAGCTGTGCCTCTCCCGTGGCGTCATCTGGGGTGATGTGCAAACCACCAAT
CCCGGCTCTACACGTCTCACTCGGAGCTCCTAGGCTTATATAAGGGGCTGGGTTTCTTAGCTTGGGGTCTCCCTCTAAGAAGCTGATCATCTATCTCTCAAGATGCATTAAAGCTTTACTGCAGAAGGATCCGAGTGTTCTGCGTCGTTCTTGCTGGCGAGATGGTAGCGCGGGACA

>IAPLTR4_I.2679650
TGTGAGGAGCCGCCCTCGCTATCGCTATTGCCGTTAGAAGATGGCGCTGACATCTGCTGTCAGTTGGAGTTAACCGTTTAAGCTGTGCCTCTCCCGTGGCGTCATCTGGGGTGATGTGCAAACCACCAATCCCGGCTCTACACGTCTCACTCGGAGCTCCTAGGCTTATATAAGGGGCTGGGTTTCTTAGCTTGGGGTCTCCCTCTAAGAAGCTGATCATCTATCT
CTCAAGATGCATTAAAGCTTTACTGCAGAAGGATCCGAGTGTTCTGCGTCGTTCTTGCTGGCGAGACGGTAGCGCGGGACATCTGGTGCCGAAACCCGGGAACTCTTCAACATCGCCGGCGCCGCGGGAGACCCCTCGGAAGACGGGGCGGATTCAGAACTGCAGGGACGTAAGTTCAGAGAGGTATGCTTTATCCTGAATACCCCCTTTCTCGACTTTGGCCTTA
GTTTGTCATCACCGTGGGACGGGTCAGTGGAGGCTTTGGTGCTAGTCCTGTTTGTCCTTCTCCTTCATCGTGGCTGGTGCTGACAGAACGGTGCTGTCAGTCTGGTGCTGTCAGTCTGGTAACGGAGTTGAGCGCCGTTTTTTAGATAAGCCGCTAGCGCGATGGGGTTGTCACAGTCAGTGGTTACTGCCCTACAGACTGTGCTAAAACAAAGAGATCTTAAAAT
TGCACCACGAACACTTCAAAATTTTGTGAAGGAGGTAGATAGAGTGGCACCATGGTATGCTTGTTCCGGGTCCCTCACTGTGGCCTCATGGAACAAGCTAGGTAAAGAACTAGACAGGAAGCATGCAGAAGGAGATCTCCACTTAGGTACCAAAGCCATTTGGAAGCTAGTTAAAAACTGCTTGGAGGATGAGGCCTGTCACCCTGCTATAATAGAAAGTCAGGGA
ACCTTAGAGGAAGTTCAGGATAGCATGTCAGAAACTGAACGGAGTGAGAGAATGGGAGCTCGCAAAAGGAAAGACATGTCTAAGAAAAAAGGCCCTCCCCAAGAAGTTAAGAAAGGGGGAGAGAGAGAAGGGAGTGATCATTCACCCCCTAACAAATCTAAGAAAAAGAAAAAGCCAGAATCTAGTCTATACCCTACAGTAGAGCTAGAAGCCTTGGAGCTAGATA
ATTCTGATTCTGACACTCTGTATTCTAGCGAAGAAGGTGGTCTAGAGGAGGAGGTGGCACGGTATGAGGAAGAAAGATATCACCCTGATAGACACCGGCCACTGAAAACAAAAATGAATGTGAGGCCACCGCCTATAAATCCGGCGGGCTCACGCCCCTCAGCGCCTCCGCAGTATGAGCTTCGGCTGAATACTGGTACTGACTCCTTTTTACCTTTAGAGGAACG
GAGAAAAATACAAGTAGCTTTTCCAGTATTCGAGAATGCGGAAGGCGGAAGAGTACATACCCCTGTAGATTACAATCAAATCAAAGAATTGGCCGAATCCGTTTGTAATTATGGGGTCAATGCTAACTTTACCACATTACAGGTGGAAAGGCTTGCTAATTTTGCCATGACTCCCACTGACTGGCAGACGACAGTGAAAGCAGTGCTTCCCAACATGGGACAATAC
ATGGAATGGAAGGCTCTCTGGTATGATGCAGCCCAGGCACAAGCTAGAGTTAATGCCACAGCTGAAGATGAGAATCAGAGGCAGTGGACATTTGAAATGCTGACAGGTCAGGGTCAACATGCCTTTAATCAAACCAACTACATCTGGGGTGTCTATGGTCAGGTGTCGGCCGCCGCTATTAAAGCATGGAAGGCCCTCACAAAAAGGGGAGAATCCAGTGGACACC
TTACAAAGATTGTCCAAGGACCCCAAGAGCCATTTTCAGACTTTGTGGCCAGAATGACAGAGGCCGCCAGTCGCATCTTTGGTGATTCAGAACAAGCTATGCCCCTGATTGAGCAACTTGTCTATGAACAAGCAACCCAGGAATGTCGAGCGGCTATCGCCCCTCGCAAAAGCAAAGGTTTACAAGATTGGCTCAGAGTGTGCAGGGAGCTGGGGGAACCCATAAC
CAATGCAGGACTAGCTGCCGCCATTTTACAAACGCACAGGTATAGAGATTTGTCTAATCGAAAAGCTTGTTTTAATTGGGGGAGAATGGGGCATCTTAAGAAAGATTGTCAAGCCCCTGAAAGAACAAGAGAATCGAAGCTTTGCCGTCGTTGTGGTAAAGGTTATCATAGGGCTAGCGAATGCAGGTCAGTCCGGGATGTAAAAGGGAGGCTTCTGCCCCCTATT
GAAGAACCCAAGGTACCCCAATCAAAAAACGGGTCACGGGGCCCATGGTCCCAGGGCCCTCAGAAATATGGGAACCAATTTCAGAAAAGCAATCAAGAGGAAGTAGAGGTTTCCGAGGAACCCCCGGAATGGACTTGCGTGCCGCCTCCGACTTCTTACTCATGCCCCAAATGAATGTTCAACCGGTCCCAGTCCATTCCCCTGGACCTTTGCCCCCTGCCACCAT
TGGGCTTATTCTGGGCCGTGGCTCCTTAACTTTACAGGGACTCATTATATATCCTGGAATTGTTGACCCGTATCATAAAGAGGAGATTCAAGTTCTCTGTTCTAGCCCTCGGGGTGTATTTTCTATAAAGCAGGGAGATAGAATTGCACAACTGGTGTTGCTGCCCTCCCTCGGGGATGGAGAAACCTACACTTTACAAAAAAGGGCTATGGGATCTTCTGGAAGT
GATTCAGCCTATCTGGCTATACCCCTAAATGAGAGACCTACAATAAAATTGTTAATTAATGGAAAAGAATTTGAAGGGATAATGGATACGGGAGCAGACAAAAGCATTATTTCACTCCATTGGTGGCCGAAGTCTTGGCCCACTGTTGTTTCATCTCATTCTCTACAAGGTCTTGGATACCAGTCCTCTCCTGCCATCAGTGCCTCAGCCTTAACCTGGCGGAATG
CTGAGGACAAACAGGGATGTTTTACCCCCTATGTGTTGCCACTCCCTGTAAATTTATGGGGACGGGATGTGTTACAAGCCATGGGCATGACCCTAACTAATGAATACTCCCCCCAGGCATCAGCCATTATGGCAAAGATGGGGTATACAAACGGTAGAGGCTTGGGTAGGCAAGAACAAGGCAGAATAAAACCCATTACGCAACATGGAAATCAGGGTAGAAAAGG
ACTGGGTTTTATTTAGGGGCCGTTGAGGCTTCACGACCCATACCGTGGAAAACAGAGGAGCCGTTATGGGTCTCTCAATGGCCTCTATCCTCTGAAAAATTAGAGGCTGTCACAAGATTAGTGCAAGAACAGGAACGGCTGGGGCATCTAGAGCCTTCTACCTCCCCATGGAATACACCAATTTTTGTTATTAAGAAGAAATCTGGGAAGTGGAGATTACTCCACG
ACCTGCGGGCCATTAACAATCAGATGCATCTTTTTGGCCCTGTTCAAAGAGGCCTTCCTTTGCTTTCTGCACTTCCTCAAGATTGGAAGCTTATTATTATAGATATTAAGGATTGTTTCTTCTCTATTCCACTTTACCCACGGGATAGACCAAGGTTTGCCTTCACTATCCCTTCTCTTAATCATATGGAACCAGACAAGAGATTTCAGTGGAAGGTACTGCCGCA
AGGCATGGCCAATAGCCCAACAATATGTCAGCTGTATGTGCAGGAGGCTTTGGAGCCAATTAGGAAGCAATTTACATCTTTAATCGTTATCCATTATATGGACGATATTCTTATTTGTCACAAAGAGTTAGATGTTTTACAAAAGGCTTTTCCCATGCTGGTGGCTGAATTAAAACAGTGGGGATTAGAGATTGCCTCAGAAAAGGTTCAAATTGCAGATACAGGG
CTCTTTTTGGGCTCAATGATTACTCCCAAAAATATAGTTCCTCAGAAAATAGAAATTCGCAAAGACCACTTACAAACATTAAATGATTTTCAGAAACTTCTGGGAGATATAAATTGGTTGAGGCCCTTTTTAAAAATTCCATCTGCTGATCTAAAACCCCTCTTCGATCTCCTGGAGGGTGAGCCTCATATCTCCTCACCCAGAAAATTTACCCCTGCCGCCCACC
GAGCCTTACAGATGGTGGAAGAGGCCTTACAGGAAGCACAATTACAACGTATTGAGGAGTCACTACCTTTTAATCTGTGTGTTTTTAAGACAGCTCAATTGCCAACTGCAGTCCTGTGGCAACATGGACCATTACTTTGGATTCATCCAAATGCTTCCCCTGCTAAGATTATTGACTGGTACCCTGATGCTGTTGCACAGCTCGCGCTTCGCGGAATAAAAGCAGC
TGTTACACATTTTGGCAGGGATCCTGATTCCTTGATTGTGCCTTATACTGCTGCACAAGTTCAGACCTTGGCAGCCACATCTAGTGATTGGGCAGTTTTAGTTACTTCCTTTTCAGGACAAATTGATAATCATTTTCCAAAGCATCCAATTTTACAGTTTGCCCTAAACCAGGCTATAGTGTTTCCACAAGTGACAGCTAAAGACCCACTCCCAGATGGGACTGTG
GTGTACACTGATGGATCGAAAACTGGTATGGGAGCTTATGTGGTCAAGGATAGGGTTATATCTAAACAATATAATGAAACTTCACCTCAAGTTGTAGAATGTCTGATAGTACTGGAGGTTCTTGAGGCTTTTCAGGACCCCTCAATATTGTATCAGATTCCTCTTATGTGGTTAATGCAGTTAATCTCCTTGAGATAGCTGGAATAATAAGATCCTCCAGCAGAG
TTGCTAACATCTTTCAGAAGATACAAGCTGCCTTGTTAAATAGGAGATTTCCTGTTTTCATTACCCATGTCCGAGCACATTCTGGACTCCCAGGTCCCATGTCCTTAGGGAATGACTTAGCGGACAAGGCTACAAAGCTGGTGGCCGCTGCCTTGTCTACCCATGCACAAGCAGCAAAAGAATTCCATAAGCGCTTTCATGTGACGGCTGAGACCTTGCGCCGTCG
[illegible]
CTCTCAAGATGCATTAAAGCTTTACTGCAGAAGGATCCGAGTGTTCTGCGTCGTTCTTGCTGGCGAGACGGTAGC

>IAPLTR3.2897495
TGTGAGGAGCCGCCCTCGCTATCGCTATTGCCGTTAGAAGATGGCGCTGACATCTGCTGTCAGTTGGAGTTAACCGTTAGAAGATGGCGCTGACATCTGCTGTCAGTTGGAGTTAACCGTTTAAGCTGTGCCTCTCCCGTGGCGTCATCTGGGGTGATGTGCAAACCACCAATCCCGGCTCTACACGTCTCACTCGGAGCTCCTAGGCTTATATAAGGGGCTGGGT
TTCTTAGCTTGGGGTCTCCCTCTAAGAAGCTGATCATCTATCTCTCAAGATGCATTAAAGCTTTACTGCAGAAGGATCCGAGTGTTCTGCGTCGTTCTTGCTGGCGAGACGGTAGCGCGGGACATCTGGTGCCGAAACCCGGGAACTCTTCAACATCGCCGGCGCCGCGGGAGACCCCTCGGAAGACGGGGCGGATTCAGAACTGCAGGGACGTAAGTTCAGAGAG
GTATGCTTTATCCTGAATACCCCCTTTCTCGACTTTGGCCTTAGTTTGTCATCACCGTGGGACGGGTCAGTGGAGGCTTTGGTGCTAGTCCTGTTTGTCCTTCTCCTTCATCGTGGCTGGTGCTGACAGAACGGTGCTGTCAGTCTGGTGCTGTCAGTCTGGTGCTGTCAGTCTGGTGCTGTCAGTCTGGTAACGGAGTTGAGCGCCGTTTTTAGATAAGCCGCC
AGCGCGATGGGGTTGTCACAGTCAGTGGTTACTGCCCTACAGACTGTGCTAAAACAAAGAGATCTCAAAATTGCACCACGAACACTTCAAAATTTTGTGAAGGATGTAGATAGAGTGGCACCATGGTATGCTTGTTCCGGGTCCCTCACTGTGGCCTCATGGAACAAGCTAGGTAAAGAACTAGACAGGAAGCATGCAGAAGGAGATCTCCACTTAGGTACCAAAG
CCATTTGGAAGCTAGTTAAAAACTGCTTGGAGGATGAGGCCTGTCACCCTGCTATAATAGAAAGTCAGGGAACCTTAGAGGAAGTTCAGGATAGCATGTCAGAAACTGAACGGAGTGAGAGAATGGGAGCTCGCAAAAGGAAAGACATGTCTAAGAAAAAAGGCCCTCCCCAAGAAGTTAAGAAAGGGGGAGAGAAAGAAGGGAGTGATCATTCACCCCCTAACAA
ATCTAAGAAAAAGAAAAAGCCAGAATCTAGTCTATACCCTACAGTAGAGCTAGAAGCCTTGGAGCTAGATAATTCTGATTCTGACACTCTGTATTCTAGCGAAGAAGGTGGTCTAGAGGAGGAGGTGGCACGGTATGAGGAAGAAAGATATCACCCTGATAGACACCGGCCACTGAAAACAAAAATGAATGTGAGGCCACCACGCCTATAAATCCGGCGGGCTCACGC
CCCTCAGCGCCTCCGCAGTATGAGCTTCGGCTGAATAATGGTACTGACTCCTTTTTACCTTTAGAGGAACGGAGAAAAATACAAGTAGCTTTTCCAGTATTCGAGAATGCGGAAGGCGGAAGAGTACATACCCCTGTAGATTACAATCAAATCAAAGAATTGGCCGAATCCGTTCGTAATTATGGGGTCAATGCTAACTTTACCACATTACAGGTGGAAAGGCTTG
CTAATTTTGCCATGACTCCCACTGACTGGCAGACGACAGTGAAAGCAGTGCTTCCCAACATGGGACAATACATGGAATGGAAAGCTCTCTGGTATGATGCAGCCCAGGCGCAAGCTAGAGTTAATGCCACGGCTGAAGATGAGAATCAGAGGCAGTGGACATTTGACATGCTGACAGGTCAGGGTCAACATGCCCTTAATCAAACCAACTACATCTGGGGTGTCTA
TGGTCAGGTGTCGGCCGCCGCTATTAAAGCATGGAAGGCCCTCACAAAAAGGGGAGAGTCCAGTGGACACCTTACAAAGATTGTCCAAGGACCCCAAGAACCATTTTCAGACTTTGTGGCCAGAATGACAGAGGCCGCCAGTCGCATCTTTGGTGATTCAGAACAAGCTATGCCCCTGATTGAGCAACTTGTCTATGAACAAGCAACCCAGGAATGTCGAGCGGCT
ATCGCCCCTCGCAAAAGCAAAGGTTTACAAGATTGGCTCAGAGTGTGCAGGGAGCTGGGGGGACCCCTAACCAATGCAGGACTAGCTGCTGCCATTTTACAAACGCACAGGTATAGAGATTTATCTAATCGAAAAGCTTGTTTTAATTGTGGGAGAATGGGGCACCGTAAGAAAGATTGTCAAGCCCCTGAAAGAACAAGAGAATCGAAGCTTTGCCATCGTTGTG
GTAAAGGTTATCATAGGGCTAGCGAATGCAGGTCAGTCCGGGATGTAAAAGGGAGTCTTCTGCCCCCTATTGAAGAACCCAAGGTACCCCAATCAAAAAACGGGTCACGGGGCCCGTGGTCCCAGGGCCCTCAGAAATATGGGAACAAATTTCAGAAAAGCAATCAAGAGGAAGTAGAGGTTTCCGAGGAACCCCCGGAATGGACTTGCGTGCCGCCTCCGACTTC
TTACTCATGCCCCAAATGAATGTTCAACCGGTCCCAGTCCATTCCCCTGGACCTTTGCCCCCTGCCACCATTGGAATTATTCTGGGCCGTGGCTCCTTAACTTTACATTGACTCATTATATATCCTGGGATTTTTGACCCGTATCATAAAGAGGAGATTCAAGTTCTCTGTTCTAGCCCTCGGGGTGTATTTTCTATAAAGCAGGGAGATAGAATTGCACAACTGG
TGTTGCTGCCCTCCCTCGGGGATGGAGAAACCTACACTTTACAAAAAAGGGCTATGGGATCTTCTGGAAGTGATTCAGCCTATCTGGCTATACCCCTAAATGAGAGACCTACAATAAAATTGTTAATTAATGGAAAAGAATTTGAAGGGATAATGGATACGGGAGCAGACAAAAGCATTATTTCACTCCATTGGTGGCCGAAGTCTTGGCCCACTGTTGTTTCATC
TCATTCTCTACAAGGTCTTGGATACCAGTCCTCTCCTGCCATCAGTGACTCAGCCTTAACCTGGCGGAATGCTGAGGGCAAACAGGGATGTTTTACCCCCTATGTGTTGCCACTCCCTGTAAATTTATGGGGACGGGATGTGTTACAAGCCATGGGCATGACCCTAACTAATGAATACTCCCCCCAGGCATCAGCCATTATGGCAAAGATGGGGTATACAAATGGT
AGAGGCTTGGGTAGGCAAGAACAAGGCAGAATAAAACCCATTACGCAACACGGAAATCGGGGTAGAAAAGGACTGGGTTTTATTTAGGGGCCGTTGAGGCTTCACAACCCATACCATGGAAAACAGAGGAGCCGTTATGGGTCTCTCAATGGCCTCTATCCTCTGAAAAATTAGAGGCTGTCACAAGATTAGTGCAAGAACAGGAACGGCTGGGGCATCTAGAGCC
TTCTACCTCCCCATGGAATACACCAATTTTTGTTATTAAGAGAAATCTGGGAAGTGGAGATTACTCCATGACCTGCGGGCCATTAACAATCAGATGCATCTTTTTGGCCCTGTTCAAAGAGGCCTTCTTTGCTTTCTGCACTTCCTCAAGATTGGAAGCTTATTATTATAGATATTAAGGATTGTTTCTTCTCTATTCCACTTTACCCACGGGATAGACCAAGGT
TTGCCTTCACTATCCCTTCTCTTAATCATATGGAACCAGACAAGAGATTTCAGTGGAAGGTACTGCCGCAAGGCATGGCCAATAGCCCAACAATATGTCAGCTGTATGTGCAGGAGGCTTTGGAGCCAATTAGGAAGCAATTTACATCTTTAATCGTTATCCATTATATGGACGATATTCTTATTTGTCACAAAGAGTTAGATGTTTACAAAAGGCTTTTTCCAT
GCTGGTGGCTGAATTAAAACAGTGGGGATTAGAGATTGCCTCAGAAAAGGTTCAAATTGCAGATACAGGCCTCTTTTTGGGCTCAATGATTACTCCCAAAAATATAGTTCCTCAGAAAATAGAAATTCGCAAAGACCACTTACAAACATTAAATGATTTTCAGAAACTTCTGGGAGATATAAATTGGTTGAGGCCCTTTTTAAAAATTCCATCTGCTGATCTAAAA
CCCCTCTTCGATCTCCTGGAGGGTGAGCCTCATATCTCCTCACCCAGAAAATTTACCCCTGCCGCCCATCGAGCCTTACAGATGGTGGAAGAGGCCTTACAGGAAGCACAATTACAACGTATTGAAGAGTCACTACCTTTTAATCTGTGTGTTTTAAGACAGCTCAATTGCCAACTGCAGTCCTGTGGCAACATGGACCATTACTTTGGATTCATCCAAATGCTT
CCCCTGCTAAGATTATTGACTGGTACCCTGATGCTGTTGCACAGCTCGCGCTTCGCGGAATAAAAGCAGCTGTTACACATTTTGGCAGGGATCCTGATTCCTTGATTGTGCCTTATACTGCTGCACAAGTTCAGACCTTGGCAGCCACATCTAGTGATTGGGCAGTTTTAGTTACTTCCTTTTCAGGACAAATTGATAATCATTTTCCAAAGCATCCAATTTTACA
GTTTGCCCTAAACCAGGCTATAGTGTTTCCACAAGTGACAGCTAAAGACCCACTCCCAAATGGGACTGTGGTGTACACTGATGGATCAAAAACTGGTATGGGAGCTTATGTGGTCAAGGATAGGGTTATATCTAAACAATATAATGAAACTTCACCTCAAGTTGTAGAATGTCTGATAGTACTGGAGGTTCTTGAGGCTTTTCCAGGACCCCTCAATATTGTATCA
GATTCCTCTTATGTGGTTAATGCAGTTAATCTCCTTGAGATAGCTGGAATAATAAGATCCTCCAGCAGAGTTGCTAACATCTTTCAGAAGATACAAGCTGCCTTGTTAAATAGGAGATTTCCTGTTTTCATTACCCATGTCCGAGCACATTCTGGACTCCCAGGTCCCATGTCCTTAGGGAATGACTTAGCGGACAAGGCTACAAAGCTGGTGGCCGCTGCCTTGT
CTACCCATGCACAAGCAGCAAAAGAATTCCATAAGCGCTTTCATGTGACGGCTGAGCCTTGCGCCGTCGTTTTGCTCTATCCAGGAAAGAGGCTAGGGAAATTGTTACTCAATGCCAGAATTGCTGCGAATTCTTACCTACTCCGCACATGGGAATAAACCCACGTGGCATTAGACCATTGCAGATGTGGCAGATGGATATTACTCATATTCCCTCTTTTGGAAG
GCTTCAATATGTTCATGTTTCTGTTGACACCTGTTCTGGCATAATGTTCGCCACACCCCTAACAGGGGAAAAGGCTTCATATGTGATTCAACATTGCCTTGAGGCCTGGAGTGCTTGGGGAAAACCCAGAATTTTAAAAACAGACAATGGACCAGCATATACCTCTCAAAAATTCCGACAGTTTTGTCGGCAAATGGATGTTACCCATCTAACTGGCCTACCTTAT
AACCCTCAAGGACAGGGCATTGTGGAACGTGCCCATCGTACACTCAAATCATATCTGATTAAACAAAAGGGGAGTATTGAGATTGAGGATGTTCTACCCTCGGTACCAAGAGTTGCTGTGTCCATGGCACTTTTTACCCTTAATTTCTTGAACTCTGATGCCCAAGGCCACACTGCGGCCGATCGCCACAGTTTAGAGCCTGATAGGCCAAAAGAAATGGTAAAAT
GGAAAGATGTCTTGACTGATCTCTGGAAAGGCCCGGATCCTATTCTTATAAGATCCAGGGGAGCTGTGTGTGTTTTTCCACAGGAGGAAGAAAACCCCCTGTGGATCCCTGAAAGACTCACTCGAAGAGCTCCTTCACAACTCCAGAATAAGAAGGATGGAGGCCAAGTGGGTGATGAAGACTCTCCTACTCGGGATGATGGTTCCTAGTATGATGGGAGAGTTAC
GGTGGGCCATCATGTCTACCTTCCCCCTACTGATGCCAGTGATGCATAATGCCCAGGTATTCCCTAGATTTTTCACAACTAATAGAGAGTTGGGGCTTGCCTTTTTGCCTTTAGATCCTCAGATACAGAGTTTTCAGGATGAGAGATCCTTGTCATTAGTAGGAAGCTTGTGCTTTACTGTGATTTCTTCATTAAAGGATAATTTTATTCATATGGATCCAGAAAG
ATCTAATGCTAATCCTTTTGAATTAATGGAAGCTGTACTGATCTTTCTCCTATGATAGTGAGAAGGGACAGATTACTTTTGATTGGAAAGGGGCATTAGGCAACAGTCTGAGTGAGGGACAGAGCTATGGATCATCTGATTGGTCCTCCTCCAATGTTAAGTATACCCGTTTTAAGAGAACTAATTATTCAGCTGCTCCAATTTGTGTTTGGCTGCCCTTTATTTG
GGTTGTGAGTAATGATACCTTAGGTAAGAATCAGAAAGAAATAAATTGCTCTGCTTAGAATTGTTTTTATGCATTATGCTGGGATGCTATTAAGCACCCTTTTGCTTTGGTAATGCGCATGCCTCGATTTGTTCCAGTTCCTGTTGAGACGCCAAGTAATTTGAGTTTGTTTAGGGAAAAGCGAGATTTTTGGGATCTCTGCTATTATTGTGGGCCTGATAGCCAC
CGCTGCGGTTACTGCTTCGGTTACCGCCTCGGCCCTTGCACTTTCTGCCACTGTGCAGACAACTCAAACCATTAATGAACTATAGGCCACTGTGACCATGGCCCTAGATAAACAGGCCACTGCCAATTCTCAGATACAAGGAGGCCTGATGTTGGTAAACCAACAGATTAATTTGGTTCAGGAACAATTGGATATATTGTGGCAGATGGCCCAATTGGGATGTGAA
CAAAAGTTGCCTGGCCTTTGTGTCACTTCAGTACAATATGAGAATTTTACAAGAGCAGCTAACCTGTCACAAGCTCTGTCTCAACATTTATTACAGAATTGGACTTTTGAGTTTGAGCAGACACTCCGTGAGTTGAGAATGGCGATCCTGCAAGTGAATTCGACGCATCTGGATCTTTCTCTGATGGAAGGCCTCTCCTCCCTGGATTTCATCTGCATTTTCTATT
TTAAGGAATGGGTGCGGGTAGGATCATTTAGCCTAGTTCTCTGCTGTGGAATAGTGTTGATGCTTTGGATGGTGTGTAAGCTCAGAGCCCAAACAAAAGAGACAAAGTGGTGGTGGCACAAGCACTTGCTGCCTTAGAACACGGAGCCTCCCCTGACATTTGGTTAACCATGTTAAAGCAATAGGTCGCTGGCCTCCCGGCTCTTGCACCCCATGAGGCTAGTCTC
ATTGCACGGGATAGAGTGGGTAGGCTTCAGCAGCCCAAGGAGTTGCAAGGCTAAGCACTGCACAGGAGAGGTCTGCGGTATAACGACTTTCTCCTGGGAGATAAGTCATCTTGCATGAAGGTTCTATGTCATGATCTCCTTCCCCCAGAAAAACGACATCGGACTGGTCAGGGCTCTCTCTGGGGATAAGACCCTGGAGAGGAGCTTTGTACATGTTCCGAATTTG
CACATGGGGATTTGACCTCTATCTCCACTCCAAAGTTGTGGGTGTCCTATTGCTTCAAAAATAAATGAATAGGGGGAGATTTGAGGAGCCGCCCTCGCTATCGCTATTGCTGTTAGAAGATGGCGCTGACATCTGCTGTCAGTTGGAGTTAACCGTTAGAAGATGGCGCTGACATCTGCTGTCAGTTGGAGTTTACCGTTTAAGCTGTGCCTCTCCCGTGGCGTCA
TCTGGGGTGATGTGCAAACCACCAATCCCGGCTCTACACGTCTCACTCGGAGCTCCTAGGCTTATATAAGGGGCTGGGTTTCTTAGCTTGGGGTCTCCCTCTAAGAAGCTGATCATCTATCTCTCAAGATGCATTAAAGCTTTACTGCAGAAGGATCCGAGTGTTCTGCGTCGTTCTTGCTGGCGAGACGGTAGCGCGGGACA

>IAPLTR3.2686032
TGTGAGGAGCCGCCCTCGCTATCGCTATTGCCGTTAGAAGATGGCGCTGACATCTGCTGTCAGTTGGAGTTAACCGTTAGAAGATGGCGCTGACATCTGCTGTCAGTTGGAGTTAACCGTTTAAGCTGTGCCTCTCCCGTGGCGTCATCTGGGGTGATGTGCAAACCACCAATCCCGGCTCTACACGTCTCACTCGGAGCTCCTAGGCTTATATAAGGGGCTGGGT
TTCTTAGCTTGGGGTCTCCCTCTAAGAAGCTGATCATCTATCTCTCAAGATGCATTAAAGCTTTACTGCAGAAGGATCCGAGTGTTCTGCGTCGTTCTTGCTGGCGAGACGGTAGCGCGGGACATCTGGTGCCGAAACCCGGGAACTCTTCAACATCGCCGGCGCCATGGGAGACCCCTCGGAAGACGGGGCGGATTCAGAACTGCAGGGACGTAAGTTCAGAGAG
GTATGCTTTATCCTGAACACCCCTTTCTCGACTTTGGCCTTAGTTTGTCATCACCGTGGGACGGGTCAGTGGAGGCTTTGGTGCTAGTCCTGTTTGTCCTTCTCCTTCATCGTGGCTGGTGCTGACAGAACGGTGCTGTCAGTCTGGTGCTGTCAGTCTGGTGCTGTCAGTCTGGTGCTGTCAGTCTGGTGCTGTCAGTCTGGTGCTGTCAGTCTGGTGCTGTCA
GTCTGGTGCTGTCAGTCTGGTGCTGTCAGTCTGGTAACGGAGTTGAGCGCCGTTTTTAGATAAGCCGCTAGCACGATGGGGTTGTCACAGTCAGTGGTTACTGCCCTACAGACTGTGCTAAAACAAAGAGATCTCAAAATTGCACCACGAACACTTCAAAATTTTGTGAAGGAGGTAGATAGAGTGGCACCATGGTATGCTTGTTCCGGGTCCCTCACTGTGGCC
TCATGGAACAAGCTAGGTAAAGAACTAGACAGGAAGCATGCAGAAGGAGATCTCCACTTAGGTACCAAAGCCATTTGGAAGCTAGTTAAAAGCTGCTTGGAGGATGAGGCCTGTCACCCTGCTATAATAGAAAGTCAGGGAACCTTAGAGGAAGTTCAGGATAGCATGTCAGAAACTGAACGGAGTGAGAGAATGGGAGCTCGCAAAAGGAAAGACATGTCTAAGA
AAAAAGGCCCTCCCCAAGAAGTTAAGAAAGGGGGAGAGAGAGAAGGGAGTGATCATTCACCCCCTAACAAATCTAAGAAAAAGAAAAAGCCAGAATCTAGTCTATACCCTACAGTAGAGCTAGAAGCCTTGGAGCTAGATAATTCTGATTCTGACACTCTGTATTCTAGCGAAGAAGGTGGTCTAGAGGAGGAGGTGGCACGGTATGAGGAAGAAAGATATCACCC
TGATAGACACCGGCCACTGAAAACAAAAATGAATGTGAGGCCACCACCTATAAATCCGGCGGGCTCACGCCCTCAGCGCCTCCGCAGTATGAGCTTCGGCTGAATACTGGTACTGACTCCTTTTTACCTTTAGAGGAACGGAGAAAAATACAAGTAGCTTTTCCAGTATTCGAGAATGCGGAAGGCGGAAGAGTACATACCCCTGTAGATTACAATCAAATCAAA
GAATTGGCCGAATCCGTTCGTAATTATGGGGTCAATGCTAACTTTACCACATTACAGGTGGAAAGGCTTGCTAATTTTGCCATGACTCCCACTGACTGGCAGACGACAGTGAAAGCAGTGCTTCCCAACATGGGACAATACATGGAATGGAAGGCTCTCTGGTATGATGCAGCCCAGGCACAAGCTAGAGTTAATGCCACGGCTGAAGATGAGAATCAGAGGCAGT
GGACATTTGAAATGCTGACAGGTCAGGGTCAACATGCCCTTAATCAAACCAACTACATCTGGGGTGTCTATGGTCAGGTGTCGGCCGCCGCTATTAAAGCATGGAAGGCCCTCACAAAAAGGGGAGAGTCCAGTGGGCACCTTACAAAGATTGTCCAAGGACCCCAAGAGCCATTTTCAGACTTTGTGGCCAGAATGACAGAGGCCGCCAGTCGCATCTTTGGTGA
TTCAGAACAAGCTATGCCCCTGATTGAGCAACTTGTCTATGAACAAGCAACCCAGGAATGTCGAGCGGCTATCGCCCCTCGCAAAAGCAAAGGTTTACAAGATTGGCTCAGAGTGTGCAGGGAGCTGGGGGGACCCCTAACCAATGCAGGACTAGCTGCCGCCATTTTACAAACGCACAGGTATAGAGATTTATCTAGTCGAAAAGCTTGTTTTAATTGTGGGAGA
ATGGGGCACCTTAAGAAAGATTGTCAAGCCCCTGAAAGAACAAGAGAATCGAAGCTTTGCCATCGTTGTGGTAAAGGTTATCATAGGGCTAGCGAATGCAGGTCAGTCCGGGATGTAAAAGGGAGGCTTCTGCCCCCTATTGAAGAACCCAAGGTACCCCAATCAAAAAACGGGTCACGGGGCCCGTGGTCCCAGGGCCCTCAGAAATATGGGAACCAATTTCAGA
AAAGCAATCAAGAGGAAGTAGAGGCTTCCGAGGAACCCCCGGAATGGACTTGCGTGCCGCCTCCGACTTCTTACTCATGCCCCAAATGAATGTTCAACCGGTCCCAGTCCATTCCCCTGGACCTTTGCCCCCTGCCACCATTGGGCTTATTCTGGGCCGTGGCTCCTTAACTTTACAGGGACTCATTATATATCCTGGAATTGTTGACCCGTATCATAAAGAGGAG
ATTCAAGTTCTCTGTTCTAGCCCTCGGGGTGTATTTTCTATAAAGCAGGGAGATAGAATTGCACAACTGGTGTTGCTGCCCTCCCTCGGGGATGGAGAAACCTACACTTTACAAAAAAGGGCTATGGGATCTTCTGGAAGTGATTCAGCCTATCTGGCTATACCCCTAAATGAGAGACCTACAATAAAATTGTTAATTAATGGAAAAGAATTTGAAGGGATAATGG
ATACGGGAGCAGACAAAAGCATTATTTCACTCCATTGGTGGCCGAAGTCTTGGCCCACTGTTGTTTCATCTCATTCTCTACAAGGTCTTGGATACCAGTCCTCTCCTGCCATCAGTGCCTCAGCCTTAACCTGGCGGAATGCTGAGGGCAAACAGGGATGTTTTACCCCCTATGTGTTGCCACTCCCTGCAAATTTATGGGGACGGGATGTGTTACAAGCCATGGG
CATGACCCTAACTAATGAATACTCCCCCAGGCATCAGCCATTATGGCAAAGATGGGGTATACAAAGGGTAGAGGCTTGGGTAGGCAAGAACAAGGCAGAATAAAACCCATTACGCAACACGGAAATCGGGGTAGAAAAGGACTGGGTTTTATTTAGGGGCCGTTGAGGCTTCACGACCCATACCATGGAAAACAGAGGAGCCGTTATGGGTCTCTCAATGGCCTC
TATCCTCTGAAAAATTAGAGGCTGTCACAAGATTAGTGCAAGAACAGGAACGGCTGGGGCATCTAGAGCCTTCTACCTCCCCATGGAATACACCAATTTTTGTTATTAAGAAGAAATCTGGGAAGTGGAGATTACTCCATGACCTGCGGGCCATTAACAATCAGATGCATCTTTTTGGCCCTGTTCAAAGAGGCCTTCCTTTGCTTTCTGCACTCCCTCAAGATTG
GAAGCTTATTATTATAGCTTCTCTATTCCACTTTACCCACGGGATAGACCAAGGTTTGCCTTCACTATCCCTTCTCTTAATCATATGGAACCAGACAAGAGATTTCAGTGGAAGGTACTGCCGCAAGGCATGGCCAATAGCCCAACAATATGTCAGCTGTATGTGCAGGAGGCTTTGGAGCCAATTAGGAAGCAATTTACATCTTTAATCGTTATCCATTATATGG
ACGATATTCTTATTTGTCACAAAGAGTTAGATGTTTACAAAAGGCTTTCCCCATGCTGGTGGCTGAATTAAAACAGTGGGGATTAGAGATTGCCTCAGAAAAGGTTCAAATTGCAGATACAGGCCTCTTTTTGGGCTCAATGATTACTCCCAAAAATATAGTTCCTCAGAAAATAGAAATTCGCAAAGACCACTTACAAACATTAAATGATTTTCAGAAACTTCT
GGGAGATATAAATTGGTTGAGGCCCTTTTTAAAAATTCCATCTGCTGATCTAAAACCCCTCTTCGATCTCCTGGAGGGTGAGCCTCATATCTCCTCACCCAGAAAATTTACCCCTGCCGCCCACCGAGCCTTACAGATGGTGGAAGAGGCCTTACAGGAAGCACAATTACAACGTATTGAGGAGTCACTACCTTTTAATCTGTGTGTTTTTAAGACAGCTCAATTG
CCAACTGCAGTCCTGTGGCAATATGGACCATTACTTTGGATTCATCCAAATGCTTCCCCTGCTAAGATTATTGACTGGTACCCTGATGCTGTTGCACAGCTCGCGCTTCGCGGAATAAAAGCAGCTGTTACACATTTTGGCAGGGATCCTGATTCCTTGATTGTGCCTTATACTGCTGCACAAGTTCAGACCTTGGCAGCCACATCTAGTGATTGGGCAGTTTTAG
TTACTTCCTTTTCAGGACAAATTGATAATCATTTTCCAAAGCATCCAATTTTACAGTTTGCCCTAAACCAGGCTATAGTGTTTCCACAAGTGACAGCTAAAGACCCACTCCCAGATGGGACTGTGGTGTACACTGATGGATCAAAAACTGGTATGGGAGCTTATGTGGTCAAGGATAGGGTTATATCTAAACAATATAATGAAACTTCACCTCAAGTTGTAGAATG
TCTGATAGTACTGGAGGTTCTTGAGGCTTTTCCAGGACCCCTCAATATTGTATCAGATTCCTCTTATGTGGTTAATGCAGTTAATCTCCTTGAGATAGCTGGAATAATAAGATCCTCCAGCAGAGTTGCTAACATCTTTCAAAAGATACAAGCTGCCTTGTTAAATAGGAGATTTCCTGTTTTCATTACCCATGTCCGAGCACATTCTGGACTCCCAGGTCCCATG
TCCTTAGGGAATGACTTAGCGGACAAGGCTACAAAGCTGGTGGCCGCTGCCTTGTCTACCCATGCACAAGCAGCAAAAGAATTCCATAAGCGCTTTCATGTGACGGCTGAGACCTTGCGCCGTCGTTTTGCTCTATCCAGGAAAGAGGCTAGGGAAATTGTTACTCAATGCCAGAATTGCTGCGAATTCTTACCTACTCCCCACATGGGAATAAACCCACGTGGCA
TTAGACCATTGCAGATGTGGCAGATGGATGTTACTCATATTCCCTCTTTTGGAAGGCTTCAATATGTTCATGTTTCTGTTGACACCTGTTCTGGCGTAATGTTCGCCACGCCCCTAACAGGGGAAAAGGCCTCATATGTGATTCAACATTGCCTTGAGGCCTGGAGTGCTTGGGGAAAACCCAGAATTTTAAAAACAGACAATGGACCAGCATATACCTCTCAAAA
ATTCCGACAGTTTTGTCGGCAAATGGATGTTACCCATCTAACTGGCCTACCTTATAACCCTCAAGGACAGGGCATTGTGGAACGTGCCCATCGTACACTCAAATCATATCTGATTAAACAAAAGGGGAGTATTGAGGATGTTCTACCCTCGGTACCAAGAGTTGCTGTATCCATGGCACTTTTTACCCTTAATTTCTTGAACTCTGATGCCCAAGGCCACACTGCG
GCCGATCGCCACAGTTTAGAGCCTGATAGGCCAAAAGAAATGGTAAAATGGAAAGATGTCTTGACTGATCTCTGGAAAGGCCCGGATCCTATTCTTATAAGATCCAGGGGAGCTGTGTGTGTTTTTCCACAGGAGGAAGAAAACCCCCTGTGGATCCCTGAAAGACTCACTCGAAGAGCTCCTTCACAACTCCAGAATAAGAAGGATGGAGGCCAAGTGGGTGATG
AAGACTCTCCTACTCGGGATGATGGTTCCTAGTATGCCAGTGATGCAGAATGCCCAGGTGTTCCCTAGATTTTTCACAACTAATAGAGAGTTGGGGCTTGCCTTTTTGCCTTTAGATCCTCAGATACAGAGTTTTCAGGACTTTTGATTGGAAAGGGGCATTAGGCAACAGTCTGAGTGAGGGACAGAGCTATGGATCATCTGATTGGTCCTCCTCCAATGTTAAG
TAGAACTAATTATTCAGCTGCCCTTTATTTGGGTTGTGAGTAATGATACCTTAGGTAAGAATCAGAAAGAAATAAATTGCTCTGCTTAGAATTGTTTTTATGCATTATGCTGGGATGCTATTAAGCACCCTTTTGCTTTGGTAATGCGCATGCCTCGATTTGTTCCAGTTCCTGTTGAGACGCCAAGTAATTTGAGTTTGTTTAGGGAAAAGCGAGATTTTTGGGA
TCTCTGCTATTATTGTGGGCCTGATAGCCACCGCCGCGGTTACTGCTTCGGTTACCGCCTCGGCCCTTGCACTTTCTGCCACTGTGCAGACAACTCAAACCATTAATGAACTATCGGCCACTGTGACCATGGTCCTAGATAAACAGGCCACTGCCAATTCTCAGATACAAGGAGGCCTGATGTTGGTAAACCAACAGATTAATTTGGTTCAGGAACAATTGGATAT
ATTGTGGCAGATGGCCCAATTGGGATGTGAACAAAAGTTGCCTGGCCTTTGTGTCACTTCAGTACAATATGAGAATTTTACAAGAGCAGCTAACCTGTCACAAGCTCTGTCTCAACATTTATTACAGAATTGGACTTTTGAGTTTGAGCAGACACTCCGTGAGTTGAGAATGGCGATCCTGCAAGTGAATTCGACGCATCTGGATCTTTCTCTGATGGAAGGCCTC
TCCTCCTGGATTTCATCTGCATTTTCTATTTTAAGGAATGGGTGGGGGTAGGATCATTTAGCCTAGTTCTCTGCTGTGGAATAGTGTCGATGCTTTGGATGGTGTGTAAGCTCAGAGCCCAAACAAAAGAGACAAAGTGGTGGTGGCACAAGCACTTGCTGCCTTAGAACACGGAGCCTCCCCTGACATTTGGTTAACCATGTTAAAGCAATAGGTCGCTGGCCT
CCCAGCTCTTGCACCCCACGAGGCTAGTCTCATTGCACGGGATAGAGTGAGTAGGCTTCAGCAGCCCAAGGAGTTGCAAGGCTAAGCACTGCACAGGAGAGGTCTGCGGTATAACGACTTTCTCCTGGGAGATAAGTCATCTTGCATGAAGGTTCTATGTCATGATCTCCTTCCCCCAGAAAAACGACATCGGACTGGTCAGGGCTCTCTCTGGGGATAAGACCCT
GGAGAGGAGCTTTGTACATGTTCCGAATTTGCACATGGGGATTTGACCTCTATCTCCACTCCAAAGTTGTGGGTGTCCTATTGCTTCAAAAATAAATGAATAGGGGGAGATGTGAGGAGCCGCCCTCGCTATCGCTATTGCCGTTAGAAGATGGCGCTGACATCTGCTGTCAGTTGGAGTTAACCGTTAGAAGATGGCGCTGACATCTGCTGTCAGTTGGAGTTAA
CCGTTTAAGCTGTGCCTCTCCCGTGGCGTCATCTGGGGTGATGTGCAAACCACCAATCCCGGCTCTACACGTCTCACTCGGAGCTCCTAGGCTTATATAAGGGGCTGGGTTTCTTAGCTTGGGGTCTCCCTCTAAGAAGCTGATCATCTATCTCTCAAGATGCATTAAAGCTTTACTGCAGAAGGATCCGAGTGTTCTGCGTCGTTCTTGCTGGCGAGACGGTAGC
GCGGGACA

>IAPLTR3.774563
TGTGAGGAGCCGCCCTCGCTATCGCTATTGCCGTTAGAAGATGGCGCTGACATCTGCTGTCAGTTGGAGTTAACCGTTAGAAGATGGCGCTGACATCTGCTGTCAGTTGGAGTTAACCGTTTAAGCTGTGCCTCTCCCATGGCGTCATCTGGGGTGATGTGCAAACCACCAATCCCGGCTCTACACGTCTCACTCGGAGCTCCTAGGCTTATATAAGGGGCTGGGT
TTCTTAGCTTGGGGTCTCCCTCTAAGAAGCTGATCATCTATCTCTCAAGATGCATTAAAGCTTTACTGCAGAAGGATCCGAGTGTTCTGCGTCGTTCTTGCTGGCGAGACGGTAGCGCGGGACATCTGGTGCCGAAACCCGGGAACTCTTCAACATCGCCGGCGCTGCGGGAGACCCCTCGGAAGACGGGGCGGATTCAGAACTGCAGGGACGTAAGTTCAGAGAG
GTATGCTTTATCCTGAACACCCCCTTTCTCGACTTTGGCCTTAGTTTGTCATCACCGTGGGACGGGTCAGTGGAGGCTTTGGTGCTAGTCCTGTTTGTCCTTCTCCTTCATCGTGGCTGGTGCTGACAGAACGGTGCTGTCAGTCTGGTGCTGTCAGTCTGGTGCTGTCAGTCTGGTGCTGTCAGTCTGGTGCTGTCAGTCTGGTAACGGAGTTGAGCGCCGTTTT
TTAGATAAGCCGCTAGCGCGATGGGGTTGTCACAGTCAGTGGTTACTGCCCTACAGACTGTGCTAAAACAAAGAGATCTCAAAATTGCACCACGAACACTTCAAAATTTGTGAAGGAGGTAGATAGAGTGGCACCATGGTATGCTTGTTCCGGGTCCCTCACTGTGGCCTCATGGAACAAGCTAGGTAAAGAACTAGACAGGAAGCATGCAGAAGGAGATCTCCA
CTTAGGTACCAAAGCCATTTGGAAGCTAGTTAAAAACTGCTTGGAGGATGAGGCCTGTCACCCTGCTATAATAGAAAGTCAGGGAACCTTAGAGGAAGTTCAGGATAGCATGTCAGAAACTGAACGGAGTGAGAGAATGGGAGCTCGCAAAAGGAAAGACATGTCTAAGAAAAAAGGCCCTCCCCAAGAAGTTAAGAAAGGGGGAGAGAAAGAAGGGAGTGATCAT
TCACCCCCTAACAAATCTAAGAAAAAGAAAAAGCCAGAATCTAGTCTATACCCTACAGTAGAGCTAGAAGCCTTGGAGCTAGATAATTCCGATTCTGACACTCTGTATTCTAGCGAAGAAGGTGGTCTAGAGGAGGAGGTGGCACGGTATGAGGAAGAAAGATATCACCCTGATAGACACCGGCCACTGAAAACAAAAATGAATGTGAGACCACCGCCTATAAATC
CGGCGGGCTCACGCCCTCAGCGCCTCCGCAGTATGAGCTTCGGCTGAATAATGGTACTGACTCCTTTTTACCTTTAGAGGAACGGAGAAAAATACAAGTAGCTTTTCCAGTATTCGAGAATGCGGAAGGCGGAAGAGTACATACCCCTGTAGATTACAATCAAATCAAAGAATTGGCCGAATCCGTTCGTAATTATGGGGTCAATGCTAACTTTACCACATTACA
GGTGGAAAGGCTTGCTAATTTTGCCATGACTCCCACTGACTGGCAGACGACAGTGAAAGCAGTGCTTCCCAACATGGGACAATACATGGAATGGAAGGCTCTCTGGTATGATGCAGCCCAGGCACAAGCTAGAGTTAATGCCATGGCTGAAGATGAGAATCAGAGGCAGTGGACATTTGAAATGCTGACAGGTCAGGGTCAACATGCCCTTAATCAAACCAACTAC
ATCTGGGGTGTCTATGGTCAGGTGTCGGCCGCCGCTATTAAAGCATGGAAGGCCCTCACAAAAAGGGGAGAGTCCAGTGGACACCTTACAAAGATTGTCCAAGGACCCCAAGAGCCATTTTCAGACTTTGTGGCCAGAATGACAGAGGCCGCCAGTCGCATCTTTGGTGATTCAGAACAAGCTATGCCCCTGATTGAGCAACTTGTCTATGAACAAGCAACCCAGG
AATGTCGAGCGGCTATCGCCCCTCGCAAAAGCAAAGGTTTACAAGATTGGCTCAGAGTGTGCAGGGAGCTGGGGGGACCCCTAACCAATGCAGGACTAGCTGCCGCCATTTTACAAACGCACAGGTATAGAGATTTATCTAGTCGAAAAGCTTGTTTTAATTGTGGGAGAATGGGGCACCTTAAGAAAGATTGTCAAGCCCCTGAAAGAACAAGAGAATCGAAGCT
TTGCCATCGTTGTGGTAAAGGTTATCATAGGGCTAGCGAATGCAGGTCAGTCCGGGATGTAAAAGGGAGGCTTCTGCCCCCTATTGAAGAACCCAAGGTACCCCAATCAAAAAACGGGTCACGGGGCCCATGGTCCCAGGGCCCTCAGAAATATGGGAACCAATTTCAGAAAAGCAATCAAGAGGAAGTAGAGGCTTCCGAGGAACCCCCGGAATGGACTTGCGTG
CCGCCTCCGACTTCTTACTCATGCCCCAAATGAATGTTCAACCGGTCCCAGTCCATTCCCCTGGACCTTTGCCCCCTGCCACCATTGGGCTTATTCTGGGCCGTGGCTCCTTAACTTTACAGGGACTCATTATATATCCTGGAATTGTTGACCCGTATCATAAAGAGGAGATTCAAGTTCTCTGTTCTAGCCCTCGGGGTGTATTTTCTATAAAGCAGGGAGATAG
AATTGCACAACTGGTGTTGCTGCCCTCCCTCGGGGATGGAGAAACCTACACTTTACAAAAAAGGGCTATGGGATCTTCTGGAAGTGATTCAGCCTATCTGGCTATACCCCTAAATGAGAGACCTACAATAAAATTGTTAATTAATGGAAAAGAATTTGAAGGGATAATGGATACGGGAGCAGACAAAAGCATTATTTCACTCCATTGGTGGCCGAAGTCTTGGCCC
ACTGTTGTTTCATCTCATTCTCTACAAGGTCTTGGATACCAGTCCTCTCCTGCCATCAGTGCCTCAGCCTTAACCTGGCGGAATGCTGAGGGCAAACAGGGATGTTTTACCCCCTATGTGTTGCCACTCCCTGTAAATTTATGGGGACGGGATGTGTTACAAGCCATGGGCATGACCCTAACTAATGAATACTCCCCCCAGGCATCAGCCATTATGGCAAAGATGG
GGTATACAAACGGTAGAGGCTTGGGTAGGCAAGAACAAGGCAGAATAAAACCCATTACACAACACGGAAATCGGGGTAGAAAAGGACTGGGTTTTATTTAGGGGCCGTTGAGGCTTCACGACCCATACCATGGAAAACAGAGGAGCCGTTATGGGTCTCTCAATGGCCTCTATCCTCTGAAAAATTAGAGGCTGTCACAAGATTAGTGCAAGAACAGGAACGGCTG
GGGCATCTAGAGCCTTCTACCTCCCCATGGAATACACCAATTTTTGTTATTAAGAAGAAATCTGGGAAGTGGAGATTACTCCATGACCTGCGGGCCATTAACAATCAGATGCATCTTTTTGGCCCTGTTCAAAGAGGCCTTCCTTTGCTTTCTGCACTTCCTCAAGATTGGAAGCTTATTATTATAGATATTAAGGATTGTTTCTTCTCTATTCCACTTTACCCAC
GGGATAGACCAATGGTTTGCCTTCACTATCCCTTCTCTTAATCATATGGAACCAGACAAGAGATTTCAGTGGAAGGTACTGCCGCAAGGCATGGCCAATAGCCCAACAATATGTCAGCTGTATGTGCAGGAGGCTTTGGAGCCAATTAGGAAGCAATTTACATCTTTAATCGTTATCCATTATATGGACGATATTCTTATTTGTCACAAAGAGTTAGATGTTTTAC
AAAAGGCTTTCCCCATGCTGGTGGCTGAATTAAAACAGTGGGGATTAGAGATTGCCTCAGAAAAGGTTCAAATTGCAGATACAGGCCTCTTTTTGGGCTCAATGATTACTCCCAAAAATATAGTTCCTCAGAAAATAGAAATTCGCAAAGACCACTTACAAACATTAAATGATTTTCAGAAACTTCTGGGAGATATAAATTGGTTGAGGCCCTTTTTAAAAATTCC
ATCTGCTGATCTAAAACCACTCTTCGATCTCCTGGAGGGTGAGCCTCATATCTCCTCACCCAGAAAATTTACCCCTGCCGCCCACCGAGCCTTACAGATGGTGGAAGAGGCCTTACAGGAAGCACAATTACAACGTATTGAGGAGTCACTACCTTTTAATCTGTGTGTTTTAAGACAGCTCAATTGCCAACTGCAGTCCTGTGGCAACATGGACCATTACTTTGG
ATTCATCCAAATGCTTCCCCTGCTAAGATTATTGACTGGTACCCTGATGCTGTTGCACAGCTCGCGCTTCGCGGAATAAAAGCAGCTGTTACACATTTTGGCAGGGATCCTGATTCCTTGATTGTGCCTTATACTGCTGCACAAGTTCAGACCTTGGCAGCCACATCTAGTGATTGGGCAGTTTTAGTTACTTCCTTTTCAGGACAAATTGATAATCATTTTCCAA
AGCATCCAATTTTACAGTTTGCCCTAAACCAGGCTATAGTGTTTCCACAAGTGACAGCTAAAGACCCACTCCCAGATGGGACTGTGGTGTACACTGATGGATCAAAAACTGGTATGGGAGCTTATGTGGTCAAGGATAGGGTTATATCTAAAAAATATAATGAAACTTCACCTCAAGTTGTAGAATGTCTGATAGTACTGGAGGTTCTTGAGGCTTTTCCAGGACC
CCTCAATATTGTATCAGATTCCTCTTATGTGGTTAATGCAGTTAATCTCCTTGAGATAGCTGGAATAATAAGATCCTCCAGCAGAGTTGCTAACATCTTTCAAAAGATACAAGCTGCCTTGTTAAATAGGAGATTTCCTGTTTTCATTACCCATGTCCGAGCACATTCTGGACTCCCAGGTCCCATGTCCTTAGGGAATGACTTAGCGGACAAGGCTACAAAGCTG
GTGGCCGCTGCCTTGTCTACCCATGCACAAGCAGCAAAAGAATTCCATAAGCGCTTTCATGTGACGGCTGAGACCTTGCGCCGTCGTTTTGCTCTATCCAGGAAAGAGGCTAGGGAAATTGTTACTCAATGCCAGAATTGCTGCGAATTCTTACCTACTCCCCACATGGGAATAAACCCACGTGGCATTAGACCATTGCAGATGTGGCAGATGGATGTTACTCATA
TTCCCTCTTTTGGAAGGCTTCAATATGTTCATGTTTCTGTTGACACCTGTTCTGGCGTAATGTTCGCCACACCCCTAACAGGGGAAAAGGCCTCATATGTGATTCAACATTGCCTTGAGGCCTGGAGTGCTTGGGGAAAACCCAGAATTTTAAAAACAGACAATGGACCAGCATATACCTCTCAAAAATTCCGACAGTTTTGTCGGCAAATGGATGTTACCCATCT
AACTGGCCTACCTTATAACCCTCAAGGACAGGGCATTGTGGAACGTGCCCATCGTACACTCAAATCATATCTGATTAAACAAAAGGGGAGTATTGAGGATGTTCTACCCTCGGTACCAAGAGTTGCTGTATCCATGGCACTTTTTACCCTTAATTTCTTGAACTCTGATGCCCAAGGCCACACTGCGGCCGATCGCCACAGTTTAGAGCCTGATAGGCCAAAAGAA
ATGGTAAAATGGAAAGATGTGCTAACTAATGAATGGAAAGGCCCGGATCCTATTCTAATAAGATCCAGGGGAGCTGTGTGTGTTTTTCCACAGGAGGAAGAAAACCCCCTGTGGATCCCTGAAAGACTCACTCGAAGAGCTCCTTCACAACTCCAGAATAAGAAGGATGGAGGCCAAGTGGGTGATGAAGACTCTCCTACTCGGGATGATGGTTCCTAGTATGCCA
GTGATGCAGAATGCCCAGGTGTTCCCTAGATTTTTCACAACTAATAGAGAGTTGGGGTTTGCCTTTTTGCCTTTAGATCCTCAGATACAGAGTTTTCAGGACTTTTGATTGGAAAGGGGCATTAGGCAACAGTCTGAGTGAGGGACAGAGCTATGGATCATCTGATTGGTCCTCCTCCAATGTTAAGTATACCCCTTTTAAGAGAACTAATTATTCAGCTGCCCTT
TATTTGGGTTGTGAGTAATGATACCTTAGGTAAGAATCAGAAAGAAATAAATTGCTCTGCTTAGAATTGTTTTTATGCATTATGCTGGGATGCTATTAAGCACCCTTTTGCTTTGGTAATGCGCATGCCTCGATTTGTTCCAGTTCCTGTTGAGACGCCAAGTAATTTGAGTTTGTTTAGGGAAAAGCGAGATTTTTGGGATCTCTGCTATTATTGTGGGCCTGATA
GCCACCGCCGCGGTTACTGCTTCGGTTACCGCCTCGGCCCTTGCACTTTCTGCCACTGTGCAGACAACTCAAACCATTAATGAACTATAGGCCACTGTGACCATGGTCCTAGATAAACAGGCCACTGCCAATTCTCAGATACAAGGAGGCCTGATGTTGGTAAACCAACAGATTAATTTGGTTCAGGAACAATTGGATATATTGTGGCAGATGGCCCAATTGGGAT
GTGAACAAAAGTTGCCTGGCCTTTGTGTCACTTCAGTACAATATGAGAATTTTACAAGAGCAGCTAACCTGTCACAAGCTCTGTCTCAACATTTATTACAGAATTGGACTTTTGAGTTTGAGCAGACACTCCGTGAGTTGAGAATGGCGATCCTGCAAGTGAATTCCACGCGTCTGGATCTTTCTCTGATGGAAGGCCTCTCCTCCCTGGATTTCATCTGCATTTTC
TTATTTTAAGGAATGGGTGGGGGTAGGATCATTTAGCCTAGTTCTCTGCTGTGGAATAGTGTTGATGCTTTGGATGGTGTGTAAGCTCAGATCCCAAACAAAAGAGACAAAGTGGTGGTGGCACAAGCACTTGCTGCCTTAGAACACGGAGCCTCCCCTGACATTTGGTTAACCATGTTAAAGCAATAGGTCGCTGGCCTCCCAGCTCTTGCACCCCACGAGGCTA
GTCTCATTGCACGGGATAGAGTGGGTAGGCTTCAGCAGCCCAAGGAGTTGCAAGGCTAAGCACTGCACAGGAGAGGTCTGCGGTATAACGACTTTCTCCTGGGAGATAAGTCATCTTGCATGAAGGTTCTATGTCATGATCTCCTTCCCCCAGAAAAACGACATCGGACTGGTCAGGGCTCTCTCTGGGGATAAGACCCTGGAGAGGAGCTTTGTACATGTTCCGA
ATTTGCACATGGGGATTTGACCTCTATCTCCACTCCAAAGTTGTGGGTGTCCTATTGCTTCAAAAATAAATGAATAGGGGGAGATGTGAGGAGCCGCCCTCGCTATCGCTATTGCCGTTAGAAGATGGCGCTGACATCTGCTGTCAGTTGGAGTTAACCGTTAGAAGATGGCGCTGACATCTGCTGTCAGTTGGAGTTAACCGTTTAAGCTGTGCCTCTCCCATGG
CGTCATCTGGGGTGATGTGCAAACCACCAATCCCGGCTCTACACGTCTCACTCAGAGCTCCTAGGCTTATATAAGGGGCTGGGTTTCTTAGCTTGGGGTCTCCCTCTAAGAAGCTGATCATCTATCTCTCAAGATGCATTAAAGCTTTACTGCAGAAGGATCCGAGTGTTCTGCGTCGTTCTTGCTGGCGAGACGGTAGCGCGGGACA

>IAPLTR4_I.2254616
TGTGAGGAGCCGCCCTTGCTATCGCTATCGCCATTAGAAGATGGCGCTGACATCTGCTGTCAGTTGGAGTTTACCATTTAAGCTGTGCCTCACCCGTCATCTGGGGTGATGAGCAAACCACCAATCCCAGCTCTACACATCTCACTCGGAGCTCCTAGGCTTATATAAGGGGCTGGGTTTCTTAGCTTGGGGTCTCCCTTTCAGAAGCTGATCATCTATCTCTCAA
GATGTATTAAAGCTTTACTGCAGAAGGATCCGAGTGTCCTGCGTCGTTCTTGCTGGCGAGACGGTAGCGCGGGACATCTGGTGCCGAAACCCGGGAACTCTTCAACATCGCTGGCGCCGCGGGAGACCCGGCGGATTCAGAACTGCAGGGACGTAAGTTCAGAGAGGTATGCTTTATCCTGAACACCCCTTTTCTCGACTTTGGCCTTAGTTTGTCATCACCGTGG
GAGTCAGTGGAGGCTTTGGTGCTAGTCCTGTTTGTCCTTCTTCTTCATCATGGCTGGTGCTGACAGAACGGTGCTGTCAGTCTGGTAAAGGAGTCGAGCGCCGTTCTTTAGATAAGCCGCCAGCGCGATGGGGTTGTCACAGTCAGTGGTTACTGCCCTACAGACTGTGCTAAAACAAAGAGATCTCAAAATTGCACCACGAACACTTCAAAATTTTGTGAAGGAG
GTAGATAGAGTGGCACCATGGTATGCTTGTTCCGGGTCCCTCACTGTGGCCTCATGGAACAAGCTAGGTAAAGAACTAGACAGGAAGCATGCAGAAGGAGATCTCCGCTTAGGTACCAAAGCCATTTGGAAGCTAGTTAAAAACTGCTTGGAGGATGAGGCCCGTCACCCTGCTATAGTAGAAAGTCAGGGAACCTTAGAGGAAGTTCAGGATAGCATGTCAGAAA
CTGAACGGAGTGAGAGAATGGGAGCTCGCAAAAGGAAAGACATGTCTAAGAGAAAGGCCCTCCCCAAGAAGTTAAGAAAGGGGGAGAGAAAGAAGGGAGTGATCATTCACCCCCTAACAAATCTAAGAAAAAGAAAAAGCCAGAATCTAGCCTATACCCTACGGTAGAGCTAGAAGCCTTGGAGCTAGATAATTCCGATTCTGACACTCTGGATTCTAGCGAAGA
AGGTGGTCTAGAGGAGGAGGTGGCACGGTATGAGGAAGAAAGATATCACCCTGATAGACACCGGCCGCTGAAAACAAAAATGAATGTGAGGCCACCGCCTATAAATCCGGCGGGCTCACGCCCCTCGGCGCCTCCGCAGTATGAGCTTCGGCTGAATACTGGTACTGACTCCTTTTTACCTTTAGAGGAACGGAGAAAAATACAAATGGCTTTTCCAGTATTCGAG
AATGCGGAAGGCGGAAGAGTACATACCCCCGTAGATTACAATCAAATCAAAGAATTGGCCGAATCCGTTCGTAATTATGGGGTCAATGCTAACTTTACCACAATACAGGTGGAAAGGCTTGCTAATTTTGCCATGACTCCCACTAACTGGCAGACAACAGTGAAAGCAGTGCTTCCCAATATGGGACAATACATGGAATGGAAGGCTTTCTGGTATGATGCAGCCC
AGGCGCAAGCTAGAGTTAATGCCACGGCTGAAGATGAGAATCAGAGGCAGTGGACATTTGAAATGCTGACAGGTCAGGGTCAACATGCCCTTAATCAAACCAACTACATCTGGGGCGTCTATGGTCAGGTGTCGGCCGCTGCTATTAAAGCATGGAAGGCCCTCACAAAAAGGGGAGAACCCAGTGGACACCTTACAAAGATTGTCCAAGGACCCCAAGAGCCATT
TTCAGACTTTGTGGCCAGAATGACCGAGGCCGCCGGTCGCATCTTTGGTGATTCAGAACAAGCTATGCCCCTGATTGAGCAACTTGTCTATGAACAAGCAACCCAGGAATGTCGAGCGGCTATCGCCCCTCGCAAAAGCAAAGGTTTACAAGATTGGCTCAGAGTGTGCAGGGAGCTGGGGGACCCCTAACCAATGCAGGACTAGCTGCCGCCATTTTACAAACG
CACAGGCGTAGAGATTTATCTAGTCGAAAAGCTTGTTTTAATTGTGGGAAAATGGGGCACCTTAAGAAAGATTGTCAATCCCCTGAAAGAACAAGAGAACTGGAGCTTTGCCATCGTTGTGGTAAAGGTTATCATAGGGCTAGTGAATGCAGGTCAGTCCGGGATATGAAAGGGAGGCTTCTGCCCCCTATTGAAGAACCCAAGGTACCCCAATCAAAAAACGGGT
CACGGGGCCCATGGTCCCAGGGCCCTCAGAAATATGGGAACCGATTTCAGAAAAGCAATCAAGAGGAAGTAGAGGCTTCCGAGGAACCCCCGGAATGGACTTGCGTGCCGCCTCCGACTTCTTACTCATGCCCCAAATGAATGTTCAACCGGTCCCAGTCCATTCCCCTGGACCTTTGCCCCTCTGCCACCATTGGGCTTATTCTGGGCCGTGGCTCCTTAACTTTA
CAGGGTCTCATTATATATCCTGGGATTGTTGACCCGTATCATAAAGAGGAGATTCAAGTTCTCTGTTCTAGCCCTCGGGGTGTATTTTTATAAAGCAGGGAGATAGAATTGCACAACTGGTGTTGCTGCCCTCCCTCGGGGATGGAGAAACCTACACTTTACAAAAAAGGGCTATGGGATCTTCTGGAAGTGATTCAGCCTATCTGGCTATACCCCTAAATGAGA
GACCTACAATAAAATTGTTAATTAATGGAAAAGAATTTGAAGGGATAATGGATACGGGAGCAGACAAAAGTATTATTTCACTCCATTGGTGGCCGAAGTCTTGGCCCACTGTTGTTTCATCTCATTCTCTACAAGGTCTTGGATACCAGTCCTCTCCTGTCATCAGTGCCTCAGCCTTAACCTGGCGGAACGCTGAGGGCAAACAGGGATGTTTTACCCCCTATGT
GTTGCCACTCCCTGTAAATTTATGGGGACGGGATGTGTTACAAACCATGGGCATGACCCTAACTAATGAATACTCCCCCCAGGCATCAGCCATTATGGCAAAGATGGGGTATACAAAGGGTAGAGGCTTGGGTAGGCAAGAATAAGGCAGACTAAAACCCATTACGCAACATGGAAATCGGGGTAGAAAAGGACTGGGTTTTATTTAGGGGCCGTTGAGGCTTCAC
GACCCATACCGTGGAAAACAGAGGAGCCGTTATGGGTCTCTCAATGGCCTCTATCCTCTGAAAAATTAGAGGCTGTCACAAGATTAGTGCAAGAACAGGAACGGCTGGGGCATCTAGAGCCTTCTACCTCCCCATGGGATACACCAATTCTTGTCATTAAGAAGAAATCTGGGAAGTGGAGATTACTCCATGACCTGCGGGCCAATTAACAATCAGATGCATCTTTT
TGGCCCTGTTCAAAGAGGACTTCCTTTGCTTTCTGCACTTCCTCAAGATTAGAAGCTTATTATTATAGATATTAAGGATTGTTTCTTCTCTATTCCACTTTACCCACGGGATAGACCAAGGTTTGCCTTCACTATCCCTTCTCTTAATCATATGGAACCAGACAAGAGATTTCAGTGGAAGGTACTGCCGCAAGGCATGGCCAATAGCCCAACAATATGTCAGCTG
TATGTGCAGGAGGCTTTGGAGCCAATTAGGAAGCAATGTACATCTTTAATCATGATCCATTATATGGACGATATTCTTATTTGTCACAAAGAGTTAGATGTTTTACAAAGGCTTTTCCCATGCTGGTGGCAGAATTAAAACAGTGGGGATTAGAGATTGCCTCAGAAAAGGTTCAAATTGCAGATACAGGCCTCTTTTTGGGCTCAATGATTACTCCCAAAAATA
TAGTTCCTCAGAAAATAGAAATTCGCAAAGACCACTTACAAACATTAAATGATTTTCAGAAACTTCTGGGAGATATAAATTGGTTGAGGCCCTTTTTAAAAATTCCATCTGCTGATCTAAAATCCCTCTTCAATCTCCTGGAGGGTGAGCCTCATATCTCCTCACCCAGAAAATTTACCCCTGCCGCCCACCGAGCCTTACAGATGGTGGAAGAGGCCTTACAGGA
AGCACAATTACAACGTATTGAGGAGTCACTACCTTTTAATCTGTGTGTTTTTAAGACAGCTCAATTGCCAACTGCAGTCCTGTGGCAACATGGACCATTACTTTGGATTCATCCAAATGCTTCCCCTGCTAAGATTATTGACTGGTACCCTGATGCTGTTGCACAGCTCACGCTTCGCGGAATAAAAGCAGCTGTTACGCATTTTGGCAGGGATCCTGATTCCTTG
ATTGTGCCTTATACTGCTGCACAAGTTCAGACCTTGGCAGCCACATCTAGTGATTGGGCAGTTTTAGTTACTTCCTTTTCAGGACAAATTGATAATCATTTTCCGAAGCATCCAATTTTACAGTTTGCCCTAAACCAGGCTATAGTGTTTCCACAAGTGACAGCTAAAGACCCACTCCCAGATGGGACTGTGGTGTACACTGATGGATCAAAAACTGGTATGGGAG
CTTATGTGGTCAAGGATAGGGTTATATCTAAACAATATAATGAAACTTCACCTCAAGTTGTAGAATGTCTGATAGTACTGGAGGTTCTTGAGGCTTTTCCAGGACCCCTCAATATTGTATCAGATTCCTCTTATGTGGTTAATGCAGTTAATCTCCTTGAGATAGCTGGAATAATAAGATCCTCCAGCAGAGTTGCTAACATCTTTCAGAAGATACAAGCTGCCTT
GTTAAATAGGAGATTTCCTGTTTTCATTACCCATGTCCGAGCACATTCTGGACTCCCAGGTCCCATGTCCTTAGGGAATGACTTAGCGGGCAAGGCTACAAAGCTGGTGGCCACTGCCTTGTCTACCCACGCACAAGCAGCAAAAGAGTTCCATCAGCGCTTTCATGTGACGGCTGAGACCTTGCGCTGTCGTTTTGCTCTATCCAGGAAAGAGGCTAGGGAAATT
GTTACTCAATGCCAGAATTGCTGCGAATTCTTACCTGCTCCCCACATGGGAATAAACCCACGTGGCATTAGACCATTGCAGATGTGGCAGATGGATGTTACTCATATTCCCTCTTTTAGAAGGCTTCAATATGTTCATGTTTCTGTTGACACCTGTTCTGGCGTAATGTTCGCCACGCCCCTAACAGGGGAAAAGGCCTCATATGTGATTCAACATTGCCTTGAGG
CCTGGAGTGCTTGGGGAAAACCCAGAATTTTAAAAACAGACAATGGACCAGCATATACCTCTCAAAAATTCCGACAGTTTTGTCGGCAAATGGATGTTACCCATCTAACTGGCCTACCTTATAACCCTCAAGGACAGGGCATTGTGGAACGTGCCCATCGTACACTCAGATCATATCTGATTAAACAAAAGAGGAGTATTGAGGATGTTCTACCCTCGGTACCAAG
AGTTGCTGTGTCCATGGCACTTTTTACCCTTAATTTCTTGAACTCTGATGCCCAAGGCCACACTGCGGCCGATCGCCACAGTTTAGAGCCTGATAGGCCAAAAGAAATGGTAAAATGGAAAGATGTCTTGACTGATCTCTGGAAAGGCCCCGATCCTATTCTTATAAGATCCAGGGGAGCTGTGTGTGTTTTTCCACAGGAGGAAGAAAACCCCCTGTGGATCCCT
GAAAGACTCACTCGAAGAGCTCCTTCACAACTCCAGAATAAGAAGGATGGAGGCCAAGTGGGTGATGAAGACTCTCCTACTCAGGATGATGGTTCCTAGTATGATGGGAGAGTTACGGTGGGCCATCATGTCTACCTTCCCCCTACCGATGCCAGTGATGCATAATGCCCAGGAGTTCCCTAGATTTTTCACAACTAATAGAGAGTTGGGGCTTGCCTTTTTGCCT
TTAGATCCTCAGATACAGAGTTTTCAGGATGAGAGAACCTTGTCATTGGTAGGAAGCTTGTGCTTTACTGTGATTTCTTCATTAAAGGATAATTTTATTCATATGGATCCAGAAAGATCTAATGCTAATCCTTTTGAATTAATGGAAGCTGTACTGATCTTTCTCCTATGGCAATGATTGCTGGAGGATATAGTGAGAAGGGACAGATTACTTTTGATTGGAAAGG
GGCATTAGGCAACAGTCTGAGTGAGGGACAGAGCTCTGGATCATCTGATTGGTCCTCCTCCAATGTTAAGTATACCCCTTTTAAGAGAACTAATTATTCAGCTGCTCCAATTTGTGTTTGGCTGCCCTTTATTTGGGTTGTGAGTAATGATACCTTAGGTAAGAATCAGAAAGAAATAAATTGCTCTGCTAGAATTGTTTTTATGCATTATGCTGGGATGCTATTA
AGCACCCTTTTGCTTTGGTAATGCACATGCCTCGATTTGTTCCAGTTCCTGTTGAGGTGCCAAGTAGTTTGAGTTTGTTTAGGGAAAAGCGAGATTTTTGGGATCTCTGCTATTATTGTGGGCCTGATAGCTGCCACTGTGGTTACTGCTTCGGTTACCGCCTCGGCCCTTGCACTTTCCGCCACTGTGCAGACAACTCATACCATTAATGAACTATCGGCCACTG
TGACCATGGCCCTAGATAAACAGGCCACTGCCAATTCTCAGATACAAGGAGGCCTGATGTTGGTAAACCAACAGATTAATTTGGTTCAGGAACAATTGGATATATTGTGGCAGATGGCCCAATTGGGATGTGAACAAAAGTTGCCTGGCCTTTGTGTTCACTTCAGTACAATATGAGAATTTTACAAGAGCAGCTAACCTGTCAAAAGCTCTGTCTCAACATTTAT
TACAGAATTGGACTTTTGAGTTTGAGCAGACACTCCGTGAGTTGAGAGTGGCGATCCTGCAAGTGAATTCGACGCGTCTGGATCTTTCTCTGATGGAAGGCCTCTCCTCCTGGATTTCATCTGCATTTTCTTATTTTAAGGAATGGGTGGGGGTGGGATCATTTAGCCTAGTTCTCTGCTGTGGAATAGTGTTGATGCTTTGGATGGTGTGTAAGCTCAGAGCCCA
AACAAAAGAGACAAAGTGATGGTGGCACAAGCACTTGCTGCCTTAGAACACGGAGCCTCCCCTGACATTTGGTTAACCATGTTAAAGCAATAGGTCGCTGGCCTCCCGGCTCTTGCACCCCACGAGGCTAGTCTCATTGCACGGGATAGAGTGGGTAGGCTTCAGCAGCCCAAGGAGTTGCAAGGCTAAGCACTGCACAGGAGAGGTCTGCGGTATAACGACTTTC
TCCTGGGAGATAAGTCATCTTGCATGAAGGTTCTATGTCATGATCTCCTTCCCCCAGAAAAAACGACATAGGGACTGGTCAGGGCTCCCTCTGGGGATAAGACCCTGGAGAGGAGCTTTGTACATGTTCCAAATTTGCACATGGGGATTTGACCTCTATCTCCACTCCAAAGTTGTGGGTGTCCTATGGCTTCAAAAATAAATGAATAGGGGGAGATGTGAGGAGC
CGCCCTCGCTATCGCTATCGCCATTAGAAGATGGCGCTGACATCTGCTGTCAGTTGGAGTTTACCGTTTAAGCTGTGCCTCACCCGTCATCTGGGGTGATGAGCAAACCACCAATCCCGGCTCTACACGTCTCACTCGGAGCTCCTAGGCTTATATAAGGGGCTGGGTTTCTTAGCTTGGGGTCTCCCTTTCAGAAGCTGATCATCTATCTCTCAAGATGTATTAA
AGCTTTACTGCAGAAGGATCCGAGTGTCCTGCGTCGTTCTTGCTGGCGAGACGGTAGCGCAGGACA

>IAPLTR4_I.3877500
TGTGAGGAGCCGCCCTCGCTATCGCTATTGCCGTTAGAAGATGGCGCTGACATCTGCTGTCAGTTGGAGTTAACCGTTAGAAGATGGCGCTGACATCTGCTGTCAGTTGGAGTTAACCGTTTAAGCTGTGCCTCTCCCGTGGCGTCATCTGGGGTGATGTGCAAACCACCAATCCCGGCTCTACACGTCTCACTCGGAGCTCCTAGGCTTATATAAGGGGCTGGGT
TTCTTAGCTTGGGGTCTCCCTCTAAGAAGCTGATCATCTATCTCTCAAGATGCATTAAAGCTTTACTGCAGAAGGATCCGAGTGTTCTGCGTCGTTCTTGCTGGCGAGACGGTAGCGCGGGACATCTGGTGCCGAAACCCGGGAACTCTTCAACATCGCCGGCGCCGCGGGAGACCCCTCGGAAGACGGGGCGGATTCAGAACTGCAGGGACGTAAGTTCAGAGAG
GTATGCTTTATCCTGAATACCCCTTTCTCGACTTTGGCCTTAGTTTGTCATCACGGTGGGACGGGTCAGTGGAGGCTTTGGTGCTAGTCCTGTTTGTCCTTCTCCTTCATCGTGGCTGGTGCTGACAGAACGGTGCTGTCAGTCTGGTGCTGTCAGTCTGGTAACGGAGTTGAGCGCCGTTTTTTAGATAAGCCGCTAGCGTGATGGGGTTGTCACAGTCAGTGG
TTACTGCCCTACAGACTGTGCTAAAACAAAGAGATCTTAAAATTGCACCACGAACACTTCAAATTTTGTGAAGGAGGTAGATAGAGTGGCACCATGGTATGCTTGTTCCGGGTCCCTCACTGTGGCCTCATGGAACAAGCTAGGTAAAGAACTAGACAGGAAGCATGCAGAAGGAGATCTCCACTTAGGTACCAAAGCCATTTGGAAGCTAGTTAAAAACTGCTT
GGAGGATGAGGCCTGTCACCCTGCTATAATAGAAAGTCAGGGAACCTTAGAGGAAGTTCAGGATAGCATGTCAGAAACTGAACGGAGTGAGAGAATGGGAGCTCGCAAAAGGAAAGACATGTCTAAGAAAAAAGGCCCTCCCCAAGAAGTTAAGAAAGGGGGAGAGAAAGAAGGGAGTGATCATTCACCCCCTAACAAATCTAAGAAAAAGAAAAAGCCAGAATCT
AGTCTATACCCTACAGTAGAGCTAGAAGCCTTGGAGCTAGATAATTCCGATTCTGACACTCTGTATTCTAGCGAAGAAGGTGGTCTAGAGGAGGAGGTGGCACGGTATGAGGAAGAAAGATATCACCCTGATAGACACCGGCCACTGAAAACAAAAATGAATGTGAGGCCACTGCCTATAAATCCGGCGGGCTCACGCCCCTCAGCGCCTCCGCAGTATGAGCTTC
GGCTGAATACTGGTACTGACTCCTTTTTACCTTTAGAGGAACGGAGAAAAATACAAGTAGCTTTTCCAGTATTCGAGAATGCGGAAGGCGGAAGAGTACATACCCCTGTAGATTACAATCAAATCAAAGAATTGGCCGAATCCGTTCGTAATTATGGGGTCAATGCTAACTTTACCACATTACAGGTAGAAAGGCTTGCTAATTTTGCCATGACTCCCACTGACTG
GCAGACGACAGTGAAAGCAGTGCTTCCCAACATGGGACAATACATGGAATGGAAGGCTCTCTGGTATGATGCAGCCCAGGCACAAGCTAGAGTTAATGCCACGGCTGAAGATGAGAATCAGAGGCAGTGGACATTTGAAATGCTGACAGGTCAGGGTCAACATGCCCTTAATCAAACCAACTACATCTGGGGTGTCTATGGTCAGGTGTCGGCCGCCGCTATTAAA
GCATGGAAGGCCCTCACAAAAAGGGGAGAGTCCAGTGGACACCTTACAAAGATTGTCCAAGGACCCCAAGAGCCATTTTCAGACTTTGTGGCCAGAATGACAGAGGCCGCCAGTCGCATCTTTGGTGATTCAGAACAAGCTATGCCCCTGATTGAGCAACTTGTCTATGAACAAGCAACCCAGGAATGTCGAGCGGCTATCGCCCCTCGCAAAAGCAAAGGTTTAC
AAGATTGGCTCAGAGTGTGCAGGGAGCTGGGGGGACCCCTAACCAATGCAGGACTAGCTGTCGCCATTTTACAAACGCACAGGTATAGAGATTTATCTAGTCGAAAAGCTTGTTTTAATTGTGGGAGAATGGGGCACCTTAAGAAAGATTGTCAAGCCCCTGAAAGAACAAGAGAATCGAAGCTTTGCCATCGTTGTGGTAAAGGTTATCATAGGGCTAGCGAATG
CAGGTCAGTCCTGGGATGTAAAAGGGAGGCTTCTGCCCCCTATTGAAGAACCCAAGGTACCCCAATCAAAAAACGGGTCACGGGGCCCATGGTCCCAGGGCCCTCAGAAATATGGGAACCAATTTCAGAAAAGCAATCAAGAGGAAGTAGAGGCTTCCGAGGAACCCCCGGAATGGACTTGCGTGCCGCCTCCGACTTCTTACTCATGCCCCAAATGAATGTTCAA

[illegible]

>IAPLTR3.2622933

[illegible]

>IAPLTR4_I.1400139

[illegible]

>IAPLTR3.198286

[illegible]

>IAPLTR4_I.688626

[illegible]

CTTATAACCCTCAAGGACAGGGCATTGTGGAACGTGCCCATCGTACACTCAAATCATATCTGATTAAA[illegible]
GAAAGATGTCTTGACTGATCTCTGGAAAGGCCCGGATCCTATTCTTATAAGATCCAGGGGAGCTGTGT[illegible]
TGCCCAGGTGTTCCCTAGATTTTTCACAACTAATAGAGAGTTGGGGCTTGCCTTTTTGCCTTTAGATC[illegible]
CTATGATAGTGAGAAGGGACAGATTACTTTTGATTGGAAAGGGGCATTAGGCAACAGTCTGAGTGAGG[illegible]
TGCATTATGCTGGGATGCTATTAAGCACCATTTTGCTTTGGTAATGCGCATGCCTCGATTTGTTCCAG[illegible]
CATTAATGAACTATAGGCCACTGTGACCATGGTCCTAGATAAACAGGCCACTGCCAATTCTCAGATAC[illegible]
CAAGCTCTGTCTCAACATTTATTACAGAATTGGACTTTTGAGTTTGAGCAGACACTCCGTGAGTTGAG[illegible]
TGGTGTGTAAGCTCAGAGCCCAAACAAAAGAGACAAAGTGGTGGTGGCACAAGCACTTGCTGCCTTAG[illegible]
GGTCTGCGGTATAACGACTTTCTCCTGGGAGATAAGTCATCTTGCATGAAGGTTCTATGTCATGATCT[illegible]
TAGGGGGAGATGTGAGGAGCCGCCCTCGCTATCGCTATTGCCGTTAGAAGATGGCGCTGACATCTGCT[illegible]
GGGGCTGGGTTTCTTAGCTTGGGGTCTCCCTCTAAGAAGCTGATCATCTATCTCTCAAGATGCATTAA[illegible]

>IAPLTR3.3873252
[illegible]

>IAPLTR3.2210086
[illegible]

>IAPLTR3.3783140
[illegible]

>IAPLTR3.1258193
[illegible]

>IAPLTR4_I.3878839

[illegible]

>IAPLTR3.2742047

[illegible]

>IAPLTR3.1815955

[illegible]

Supplementary Data 5 Golden hamster LINE L1 full-length intact insertions

Full-length intact LINE L1 retrotransposon inserts in FASTA format from hamster genome from [11].

>Lx5.190629
[illegible]

>Lx5.198123
[illegible]

>Lx5.204379
[illegible]

>Lx5.204611
[illegible]

CTGAGCTGAACTCAAGAAAGCCAGTATCAGAGAGGGAGGACTGATGATCAAAGGGATTAAGACTGGGCTGGTTGAACCCC[illegible]
CCCATCCCAAGTATACACACGGTCTCTGGACTCCCATAGAACAATGTGAGGTGCACTCTCACCCTGAACTCTTGGGGAAGG[illegible]
GACTTGTGAAGCAACCTTGTTTCTTATCTAAATTTAAAATGGAAAATAAAAAAAATATTAATATAATAAAAAAAAA

>Lx5.42264
GTAAGACTAATCACCTCTCTATGTATTAAGGCTGGAAATGTCATGGAGTATGAGGAGTTGGGACTCAAAAGCCTGCAAAAG[illegible]
AATTAGTAGATTCTGTTTTTTGGTTTTGTTTTGTTTTGTTTGTTTGGTTTGGTTTGTTTGTTTTTTTTTGGCTGTACATT[illegible]
GTCACAGGAGATGGTCAGTTCACTCTACATATCTGCTATTGAGAGAAGATTAAGATGAGATCTTCTTTGTGGATTCCTGGA[illegible]
AGGACACTCAGATTTCCCTGGGAAGGGGAAATAGAAGAAATTTTGTGAGTAGACTGAGAACAAGTAGGGAAAGGAACACAA[illegible]
ATAAATCAAATAGATGGGAAGTTGGGAAAAGATCTGGGATGAGATGGGGAAGGGGAAACCATGATCAGAATATATTACAAG[illegible]
ACTGTGCCTCCCCTACAGGCAAGAGCCTAAGCATCCCAGAGTCCTGGTCTTCTTTTCCAGCAAAGGAACTAGATAAACCAG[illegible]
TCAAAGAACAGGTTATAATAATTCTCCTCAAATTATTCCATGCAACAGAAGCAGAAGGGTCTTTGCTAAACTCTTTTTATG[illegible]
[illegible]

>Lx5.57421
[illegible]

>Lx5.63450
[illegible]

>Lx5.78727
[illegible]

>Lx5.113353
[illegible]

>Lx5.115443
[illegible]

>Lx5.338131
[illegible]

>Lx5.344047
[illegible]

>Lx5.378646
[illegible]

[illegible]

>Lx5.424807
[illegible]

>Lx5.426247
[illegible]

>Lx5.486837
[illegible]

>Lx5.519861
[illegible]

[illegible]

>Lx5.530661

[illegible]

>Lx5.635261

[illegible]

>Lx5.668595

[illegible]

>Lx5.688284

[illegible]

[illegible]

>Lx5.692227

[illegible]

>Lx5.726789

[illegible]

>Lx5.747346

[illegible]

>Lx5.759982

[illegible]

[illegible]

>Lx5.1033771

[illegible]

>Lx5.833451

[illegible]

>Lx5.883434

[illegible]

>Lx5.902128

[illegible]

[illegible]

>Lx5.959873

[illegible]

>Lx8.965716

[illegible]

>Lx8.1188930

[illegible]

>Lx5.1114484

[illegible]

[illegible]

>Lx5.1245366

[illegible]

>Lx5.1488358

[illegible]

>Lx5.1545527

[illegible]

>Lx5.1303944

[illegible]

[illegible]

>Lx5.1317163
[illegible]

>Lx5.1319435
[illegible]

>Lx5.1332782
[illegible]

>Lx5.1340016
[illegible]

AGAGAGTTTTCAATAGAGGAGTCTAAAATGGCTGAGAGGCACTTGAGAAAGTGCTCAACATCCTTAGCCATCCGGGAAATGCAAATCAAAGCAACTCTGAGATACCATCTCACTCCTGTCAGAATG[illegible]
TTTGGAAAGCAGTATGGAGATTCCTCAGAAAAATGGGAATAAACCTACCACAAGACCCAGCAACTGCACTCTTAGGCATATATCCAAAAGATGCCCATTCATATAACAAAGATGTCTGTTCCACTA[illegible]
GGAGTACTATTCAGCAGAAAAAAATAATGGAATCATGAGATTCGCAGGCAAATGGATGACACTAGAAGAAACCATCCTGAGTGAGGTAACCCAGTCACAAAAAGAGAAACATGGCATGTACTCTCT[illegible]
CTGAGCTGAACTCAAGAAAGCCAGTATCAGAGAGGGAGGACTGATGATCAAAGGGATTAAGACTGGGCTGGTTGAACCCCCAGAAACAGCTGACCAGACAAAGGTTAACTCTTGAATCCCAGGCAT[illegible]
CCCATCCCAAGTATACACACGGTCTCTGGACTCCCATAGAACAATGTGAGGTGCACTCTCACCCTGAACTCTTGGGGAAGGGTCTAGGCCCTGCCCTATATGAAATGACAGACTATGGACACCCCC[illegible]
GACTTGTGAAGCAACCTTGTTTCTTATCTAAATTTAAAATGGAAAATAAAAAAAATATTAATATAATAAAAAAAAAAAAAAAAAAAAAAAA

>Lx5.1343686
GAGGAAGAGGAGAAGGAGGAGGAGGAGGAGGAGGAGGAGGAGGAGGAGGAGGAGGAGGAGGAGGAGAAAGACCCTGGGACTACACATGTGGCCATGCAGAGGGGGTGGAAAGAGACCACA[illegible]
AAACACAAAATGCATCATTTGTATTTAAACACAAGACAAAACTTGATCCAGTTATTTATATGTATATTATATCCAGCCAATGAAATCAGACAAAAGTTTACTCAAATTAGCATTTCTGAATCCTAG[illegible]
AAAAGACATTAAGATATGATTCTCTTTACTGCCTCACTAAACAGAGGGAACAAACCATTATCAAACTTTTGCATGTCAGCAACAGAAATGCTTCAATGGATCCTTGAGGTGATGATGTGTAAGGGG[illegible]
ACACTTGGGGTATAATGCAATTAATTACAGGAAGAAACCTGCAATGTGTTATTTTAGCCATAAGAGACAGAATGTTCCACTTTTAAAGAATTTTAAGTAATTTAATTAATATTTTTATTTTCGATA[illegible]
TAAAAATGACAATTCATTCAATGTATTTTTAAAATTGTTTTGAACCGTGGTGTACAAAATGTTATACTAAGCTATGACCCATAGTCTATTAAATATATAGGAGAATTCAAGGTTGATTTCCAAATT[illegible]
AACTGTATGTATAATAAAACATAATAAGAAATCTACCATGCTTAAAATTATGATAACGCAGTTAAAATTCATTGAATTTTTATATATTACTACTTAAATTTTATTCTCCAAAGAGTTAAGGTCATT[illegible]
GAGTTATTCTTTAAGGAAATATGTATACTAATAAGAGGTATTTCAATACTGTTGATTTTAGAAAGTGTGTTTTTAACAATTAAGAAAGCCTAATTTCGTCCAGGAAAGGAGAGGAGGCTGGGAGTA[illegible]
[illegible]

>Lx5.1351049
[illegible]

>Lx5.1385961
[illegible]

>Lx5.1811153
[illegible]

[illegible]
GACTTGTGAAGCAACCTTGTTTCTTATCTAAATTTAAAATGGAAAATAAAAAAATATTAATAAATAAAAAAAAAAAAAAAA

>Lx5.1601977
[illegible]

>Lx5.1622571
[illegible]

>Lx5.1647466
[illegible]

>Lx5.1754206
[illegible]

>Lx5.1883302

[illegible]

>Lx5.1891196
[illegible]

>Lx5.1921904
[illegible]

>Lx5.1967146
[illegible]

>Lx5.1973028
[illegible]

[illegible]

>Lx5.1974466

[illegible]

>Lx5.2028686

[illegible]

>Lx5b.2113870

[illegible]

>Lx5.2117329

[illegible]

[illegible]

>Lx5.2146413
[illegible]

>Lx5.2175746
[illegible]

>Lx5.2180868
[illegible]

>Lx5.2212469
[illegible]

[illegible]

>Lx5.2222377

[illegible]

>Lx5.2222557

[illegible]

>Lx5.2242883

[illegible]

>Lx5.2259369

[illegible]

[illegible]

>Lx5.2267807

[illegible]

>Lx5.2565466

[illegible]

>Lx5.2577522

[illegible]

>Lx5.2605591

[illegible]

[illegible]

>Lx5.2636012

[illegible]

>Lx5.2656634

[illegible]

>Lx5.2700315

[illegible]

>Lx5.2901944

[illegible]

[illegible]

>Lx5.2758396

[illegible]

>Lx5.2810116

[illegible]

>Lx5.2837211

[illegible]

>Lx5.2848103

[illegible]

[illegible]
ACTGACTTGTGAAGCAACCTTGTTTCTTATCTAAATTTAAAATGGAAAATAAAAAAAATATTAATATAAAAAAAAAAAAAGAAAAAAAAAA

>Lx5.2851779
[illegible]

>Lx5.2853898
[illegible]

>Lx5.2983767
[illegible]

>Lx5.2996099
[illegible]

CTGAGCTGAACTCAAGAAAGCCAGTATCAGAGAGGGAGGACTGATGATCAAAGGGATTAAGACTGGGCTGGTTGAACCCCCAGAAACAGCTGACCAGACAAAGGTTAACTCTTGAATCCCAGGCAT[illegible]
CCCATCCCAAGTATACACACGGTCTCTGGACTCCCATAGAACAATGTGAGGTGCACTCTCACCCTGAACTCTTGGGGAAGGGTCTAGGCCCTGCCCTATATGAAATGACAGACTATGGACACCCCC[illegible]
TACTGACTTGTGAAGCAACCTTGTTTCTTATCTAAATTTAAAATGGAAAATAAAAAAAATATTAATATAAAAAAAAAAATAAAAATAAAAA

>Lx5.3041833
AATACACAACTAGTAAAAATCCTAGTTCCAAAAGGGAGAAGTAGAGACACAGCAAGAAGAGACTGAACCAAAGCAAGACTGAATCACACAGGAGAGCAAACATCAAATCCTACCCCTGCATGTCCA[illegible]
AGTAGACTCTGTTCTATGAGTGAAGCTTTCCTCTACAGATCTGTCATTAGGATTGGGATGGGGATACGGCTCCAGGCTGCTCCCAGCAGCTGAAGGATTTCTTTCTAGAGACAAAACGTGGTTTT[illegible]
GAGATTTTAGAAGATTGGTAGGAGAGTCCTTATAATGAGAACAATCTGTATACTGTTTCTTGTGGTTCAGGAGTAGGATAGCCTATTCATCAGCCATTACATAAGTCTCTGCTGAGAAAGCAAAGA[illegible]
ATTGAGGGCATCAGAAACTCTGCTCCTGCCGCCTCCTGTGGTGCCTAGCCTTGGTTTTCTACATTCTCCACCCAGTAAAAGCAGGAGTCTTGGTTTTGCCTCAGCAAAGAATTTCAGAATGAGCCA[illegible]
CCAAGAGTGTGTGTGGCTTCTCAAAGGAGAGTCAGCGTTCAAGAGTGTGTTATTATTTCTCAAGAAAGAGTAGTGTCGAAACAGGCCAGCTTCCCGGCCTGAGTTCCAGAGCCTGCAGATCGCCTG[illegible]
GAGCTCCCAATCCCGGCAGGAGCATCAGGTAAACAGGCCAGCTTCCAGGCCTGTGTTCCAGAGCCTGCAGATCGCCTGGGAACTGCACAGAGCTCCCAATCCCGGCAGGAGCATCAGGTAAACAGG[illegible]
GCCTGTGTTCCAGAGCCTGCAGATCACCTGGGAACTGCACAGAGCTCCCAATCCCGGCGGGAGCATCAGGTAAACAGGCCAGCTTCCCGGCCTGTGTTCCAGAGCCTGCAGATCGCCTGGGAACTG[illegible]
[illegible]

>Lx5.3047083
[illegible]

>Lx5.3047588
[illegible]

>Lx5.3095567
[illegible]

>Lx5.3106010

[illegible]

>Lx5.3120881

[illegible]

>Lx5.3124274

[illegible]

>Lx5.3198273

[illegible]

>Lx5.3348503

[illegible]

AGCTGTCCCTCTGGCATCATCAGGTAAACAGGCCAGCTTCCCGGCCTGAGTTCCAGAGCCTGCAGATCGCCTGGGAACTGCACAGAGCTCCCAATCCAGGCGGGAGCATCAGGTAAACAGGCCAGCTTCCCGGCCTGTGTTCCAGAGCCTGCAGATCGCCTGGGAACTGCACAGAGCTCCCAATCCCGGCGGGAGCAAAGCACCCCAGACTCCAGATCTTCTCTCC
CACCAACGATTCCAGGAAAGGAGAGGAGGCTGGGAGTACAGGGAAGTAGCTGTCCCTCTGGCATCATCAGGTAAACAGGCCAGCTTCCTGGCCTGAGTTCCAGAGCCTGCAGATCGCCTGGGAACTGCACAGAGCTCCCAATCCCGGCGGGAGCATCAGGTAAACAGGCCAGCTTCCCGGCCTGTGTTCCAGAGCCTGCAGATCGCCTGGGAACTGCACAGAGCTC
CCAATCCCGGCGGGAGCATCAGGTAAACAGGCCAGCTTCCCGGCCTGTGTTCCAGAGCCTGCAGATCGCCTGGGAACTGCACAGAGCTCCCAATCCCGGCGGGAGCATCAGGTAAACAGGCCAGCTTCCCGGCCTGTGTTCCAGACCCTGCAGATCGCCTGGGAACTGCACAGAGCTCCCAATCCCGGCGGGAGCAAAGCACCCCAGACTCCAGATCTTCTCTCCC
ACCAACGATTCCAGGAAAGGAGAGGAGGCTGGGAGTACAGGGAAGTAGCTGTCCCTCTGGCATCATCAGGTAAACAGGCCAGCTTCCTGGCCTGAGTTCCAGAGCCTGCAGATCGCCTGGGAACTGCACAGAGCTCCCAATCCCGGCGGGAGCATCAGGTAAACAGGCCAGCTTCCCGGCCTGTGTTCCAGAGCCTGCAGATCACCTGGGAACTGCACAGAGCTCC
CAATCCCGGCGGGAGCATCAGGTAAACAGGCCAGCTTCCCGGCCTGTGTTCCAGAGCCTGCAGATCGCCTGGGAACTGCACAGAGCTCCCAATCCCGGCGGGAGCATCAGGTAAACAGGCCAGCTTCCTGGCCTGTGTTCCAGACCCTGCAGATCGCCTGGGAACTGCACAGAGCTCCCAATACCGGCGGGAGCAAAGCACCCCAGACTCCAGATCTTCTCTCCCA
CCAACGATTCCAGGAAAGGAGAGGAGGCTGGGAGTACAGGGAAGTAGCTGTCCCTCTGGCATCATCAGGTAAACAGGCCAGCTTCCCAGCCTGAGTTCCAGAGCCTGCAGATCGCCTGGGAACTGCACAGAGCTCCCAATCCCGGCGGGAGCCAAGCACCCCAGACTCCAGATCTTCTCTCCCACCAAAGAGACCAGGAAACTACAGGAACCAGGGAGACCAGGGG
AGTAGCTGTTCCGGATCAGGTACAACAGGACCCAGCATAGGGTCCAGGGGACCAGGTAGGAGGCTAAGCGCCCCAGATTCCTGTTCTTCTTCTCTACCCACGGACCAGCATTTGGACAAGGCATAGAAGAACCACTAGATTACTGGACTTACTAGCACCCACCAAAAGGGAACTCTGGTCCCCAGACCAGGAAGGGGCTCTTTCTGCACCCACTAAAGAAGAGAT
GGGAAAGAGACAATACAAAAACACAGTCAGCAACAGAAAAACAAATATGACACCGCCAGATTCTAGAGACTCCACACCAGCAAGACCCCAATATTCAAACGCGAAGGAAGCAGAAGAGAACGATCTTAAAAGCACTTTCATGAAAATGATAGAGGCCCTAAAAGAGGATATTGGAAAATCCCTCAAAGAAATGGAAGAAAAAACAAACCAAAAAATGCAAGAAATG
GAAGAAAAAACAAACCAAAAAATGCAAGAATTGGAAGAAAAAACAAACCAAAAACGGCACGAAATGGAAGAAGAAATGAAGCAAAAAATGCAAGAAATGGAAGAAAAAGCAAAGCAAAAAATGCAAGAATTAGAAGAAGAAACATACCAAATAAGGCAGGAAATGGAAGAAGCAACAAAGCAAAAAATGCAAGAAATGGTAGAAAAAACAAAGCAAAAAATGAAAA
AAATGGAGGAAGAAACAAACCAAAAAAGGCAGGAAAGGGAAGAAGAAACAAAGCACAAAATCCAAGAAATGGAACAAAAAGCAAACCAAAAAATGCAAGAAATGGAAGAAAGAACAAACCAAAAAATTCATAAAAGGCAACAAACGGACGAACAGATAAACAGAAAATTGCAAGAAGCAATGCAAATGCTGCAAGGGTTGAAAACTGAAATAGAGACACTAAAAAA
AACACAATCTGAAGAAAGGCAGAAAATAGAAAAGCTGGACAAACAGTCAGGCACTACCAATACAAGCATTACCAACAGAATAGAAGAGATGGAAGAGAGAATCTCAAGTGTTGAAGATACATTAGGAGAAATGGATTTATCAACCAAAGAAAACCTTAAATCCAACAAATCCTTAACACAAAACATCCAGGAATTATGGGACACCATGAAAAGACCAAACCTAAGA
ATAATAGGTATAGAAGAAGGAGAAGAAATCCACCTCAAAGGGGCAGAAGGCATATTCAACAAAATCATAGAAGAAAACTTTCCCAACCTTAAGAAAGACATGCCAATGAAGGTACAAGAAGCCTACAGAACACCAAATAGAGTGGACCACAAAAAAAAATCCCCTCGCCACATAATAATCAAGACCCCAAACATACAGAATAAAGAAAGAATATTAAGAGCAGCTA
AGGAAAAAGGTCAAGTAACATATAAAGGCAGACCTATCAGAATTACACCTGATTTCTCCATGGAAACTCTGAAAGCCAGAAGGTCCTGGATAGATATTCTACCTACACTAAGAGACCATGGATGCCAGCCCAGACTACTATACCCGGCAAAGCTCTCAATCACTATAGACGGAGAAAACAAGATATTCCATGACAAAACTAGATTCAACCAATATGTGTCCACTAA
CCCAGCCCTACAGAAAATTTTGGAAGGAAAACTCCAACCCAAGGAAGCTAACTACAACCAGAAAAACATAAGCATTAGAGAATCCCTCTCTACCAACAGCCAAAAGAACAAAGGGGCGAAATTCCACCCATAATTCCACCAACAACAACCATCGCAAATCGAAGAAGGATGAGCAATCAATGGTCATTAATATCCCTCAATATTAATGGCCTTAACTCACCCATAA
AAAGACACAGACTAGCAGAATGGATTCGAAGACAGAACCCTTCCTTCTGCTGCATACAAGAAACACACCTCACCCTCAAAGACAGACATTACTTGAGAGTTAAAGGTTGGGAAAAAATTTTTCAATCAAATGGACCCAAGAAACAAGCAGGGGTAGCAATCCTAATATCTAACAAACTAGACTTTAAAATAAAATCAATCAAAAGAGATGAAGGAGGTCATTACAT
ACTCATCACAGGTGAAACCCACCAAGACGAAGTGTCTATCCTGAACATCTATGCCCCCAATACAAAGGCATCCACATTCGTTAAAGAAACATTATTAGAACTTAAATCTCATCTAAAGCCCCACACAGTTATAGTGGGAGATTTCAACACACCACTCTCACCACTAGACAAGAATACCAGACAGGAACTCAACAAAGAAGCAAAGGGTCTAATAGAAGTAATGACC
CAACTGGGATTAACAGATATCTATAGAACATTTCATCCACATAAAAAAGAATATACCTTCTTCTCACCACCACATGGAACCTTCTCAAAATCGACCACATACTTGGCAACATATCAAACCTCAGCAGCTACAAAAAAATTGGAATAACTCCATGCATCTTATCAGACCACCATGCCTTAAAATTAGAATTCAACAACAACACAAAGTGCAGAAAACCTACAAACT
CATGGAAATTGAATAATGCTCAACTGCACCATCCCTGGGTCAAAGAGGAAATAAAAAAAGAAATTAAAGACTTCCTCGAACTCAATGAGAATGAAGACACAACATACCCAAACCTATGGGACACTTTGAAAGCAGTGCTACGGGGGAAGTTCATAGCACTAAGTGCTCACATGAAGAAACAGGAAAACAGTTATACCAGAGAATTAACATCACAGCTAAAAGCTCT
AGAACACAAAGAAGCAAACTCCCCCAGGAGGAGTAGACGCCAGGAAATAATCAAATTGAGGGCTGAAATCAACAAAGTAGAAACCAGGAAAACAATACAAAGAATCAATGCAACGAAGAGTTGGTTCTTTGAGAAAATCAACAAGATAGACAAACCTTTGTCCAAACTTACCAAACAGCAGAGAGTGAACACGCAAATTAACAAAATCAGAAATGAAAAGGGGGAC
ATAACAACAGACTCTGAGGAAATCCAGAGAATCATTAGGTCATACTTTGAAAACCTGTACTCCACAAAATTCGAAATTTAAAGGAAATGGATGACTTCCTGGACAGCTATCATTTGCCAAAACTAAATCAAGAACAGATAAGCAATTTAAATAGACCTATAACCCCTATGGAAATAGAAGCTGTCATCAAAAGTCTCCCAACCAAAAAAAGTCCAGGTCCAGATG
GCCTCAGTGCAGAATTCTACCACAAATTCAAAGAACAGCTTATACCAATTCTTCTCAAAGTGTTCCACACAATAGAAGCAGAAGGGTCTTTGCCAAACTCTTTCTATGAGGCTTCAATAACCTTGATCCCCAAGCCACACAAAGATACAACCAAGAAAGAGAACTTCAGACCAATATCCCTCATGAACATTGATGCAAAAATACTCAATAAAATACTGGCAAATCG
GATCCAAGAACACATCAAAACAATCATTCACCACGATCAAGTAGGTTTCATCCCAGGGATGCAGGGATGGTTCAACATACGAAAATCCATCAACGTAATCCACCATATAAACAAGCTGAAAAACAAAAATCACATGATCATCTCACTAGATGCTGAAAAAGCCTTTGACAAAATCCAACATCCCTTCATGATAAAGGTCTTGGAGAGATCTGGGATAACAGGACA
TACTTAAACATAATAAAAGCCATATACAGTAAACCAACAGCCAACATCAAACTAAATGGACAGAAACTCAACGCAATTCCTCTAAAATCAGGAACAAAACAAGGCTGTCCACTCTCTCCATACCTTTTCAACATGGTACTTGAAGTTCTAGCTATAGCCATACGACAACAAAGGGGGATCAAGGGGATACAAATTGGAAGGAAGAAGTCAAACTTTCACTGTTTG
CAGACGATATGATAGTTTATATCAGTGACCCAAAGAACTCTACCAGGGAACTCTTACAGCTGATTAACACCTTCAGCAAAGTGGCAGGTTACAAGATCAACTCAAGAAAGTCAGTAGCCCTACTATATACAGATGATAAAAGGGCTGAAAGAGAAATCAGAGAAACATCACCCTTTACAATTGCCACAAACAACATAAAATACCTTGGGGTAACGCTAACCAAACA
AGTGAAAGACCTGTATCGTAAGAACTTTGAATCTTTAAAGAAAGAAATTAAAGAAGCCCTCAGAAAATGGAAAGATCTCCCATGCTCATGGATAGGTAGGATCAATGTAGTAAAAATGGCAATCCTGCCAAAAGCAATCTACAGATTCAATGCTATCCCCATTAAAATCCCAGCACAATTCTTCACAGACCTTGAAAGAACAATAATCAATTTTGTATGGAAAAAC
AAAAGACCCAGGATAGCCAAAACAACCCTGTTCAACAAGGCAACGTCCGGAGGCATCAATATCCCTGACCTCAAGCTCTATTATAGAGCTATAGTCCTGAAAACAGCTTGGTATTGGCACAAAAATAGACAGGCAGACCAATGGAATCGAATTGAAAATCCGGGTATTAACCCACACACGTACGAACATCTGATCTTCGACAAAGAAGCTAAAAATATACAATGGA
ATAAAGAAAGCATCTTCAACAAATGGTGCTGGCATAATTGGAGACTGGCATGTAGAAAAATGCGGATCGATCCATGTTTATCGCCATGCACAAAACTTAAGTCCAAATGGATCAAAGACCTCAACATAAATCCAGCCACACTGAACCTCTTAGAAGAGAAAGTGGGTGGTACCCTTGAAAGAATTGGTACAGGAGACAGCTTCCTGAACATAACACCAGTAGCACA
GACAGTGAGCTCAACAATTAATAAATGGGACCTACTGAAACTGAGAAGCTTCTGTAAGGCAAAGGACACAGTCAGCAAGACAAAAAGGCAGCCCACAGAATGGGAAAAAATATTCACTAATCCCACATCTGACAGAGGGTTGATCTCCAAAATTTACAAAGAACTCAAGAAGCAAGTCTCCAAAACAGCAAAGGACCCAATTAAGAAGTGGGCTGCAGAACTAAAT
AGAGAGTTTTCAATAGAGGAGTCTAAAATGGCTGAGAGGCACTTGAGAAAGTGCTCAACATCCTTAGCCATCCGGGAAATGCAAATCAAAGCAACTCTGAGATACCATCTCACTCCTGTCAGAATGGCTAAAATCAAAAACACCAATGACAATTTATGCTGGAGAGGATGTGGTGAAAGGGGGACACTCCTCCATTGCTGGTGGGAGTGCCAACTGGTTCAGCCAC
TTTGGAAAGCAGTATGGAGATTCCTCAGAAAATGGGAATAAACCTACCACAAGACCCAGCAACTGCACTCTTAGGCATATATCCAAAGATGCCCATTCATATAACAAAGATGTCTGTTCCACTATGTTCATAGCAGCTTTATTTGTAATAGCCAGAACTTGGAAGCAACCTAGATGTCCCTCACCCGAAGAATGGATAGAGAAAATGTGGTACATCTACACAAT
GGAGTACTATTCAGCAGAAAAAAATAATGGAATCATGAGATTCGCAGGCAAATGGATGACACTAGAAGAAACCATCCTGAGTGAGGTAACCCAGTCACAAAAAGAGAAACATGGCATGTACTCTCTCATATATGGATTTTAGACATAGATCTGGGGAGTGCCAGCCTACAATCCTATTGCCAGAGAGTCTGGGAAACAGAACTTCATTTACTGGCTGGTTAAGTA
CTGAGCTGAACTCAAGAAAGCCAGTATCAGAGAGGGAGGACTGATGATCAAAGGGATTAAGACTGGGCTGGTTGAACCCCCAGAAACAGCTGACCAGACAAAGGTTAACTCTTGAATCCCAGGCATCAGCCTGGGAATCCAGCATGGGACTGATCAAGACCCCCTGAATCTGGGTGTCAGTGAGGAGGCCTAGGTAATCTATTGAGCCTCGGACAGCAGATCAGTA
CCCATCCCAAGTATACACACGGTCTCTGGACTCCCATAGAACAATGTGAGGTGCACTCTCACCCTGAACTCTTGGGGAAGGGTCTAGGCCCTGCCCTATATGAAATGACAGACTATGGACACCCCCATGGAGGGCCTCACCCTCCCTGGGGGAATGAGGGGATAACGGGGGGGGGGGATGGGGGGAGGCACGGGAGGAGGGGAGGGAAAGGGAGAGGGACCTCG
TACTGACTTGTGAAGCAACCTTGTTTCTTATCTAAATTTAAAATGGAAAATAAAAAAATATTAATAAAAAAAAAAAAAAAAAAGAAAA

>Lx5.3349044
TCGTAACTACTAATCTATGTATTTCTACACAGCCTTATCTTACCAGAGAATGGCTGGTGCATCCTGTCTTCCTGATTTATACAGGGCGTCTCTTCACAGCCTCTCTTTCTGCTTTCCTCTCTCCAGAGTTCTCCTGGTCTCCTAGCCCTGCCTATCTTCCTGCCTTTCCTGGCCAGTCAGTGTTTTATTGATCAACCACTAAGAGAAGGAAAGCATCTGCCATCAA
AAAAATACTGTTTCCGATCATCAGGTAAACGGGCCAGCTTCCAGGCCTGAGTTCCAGAGCCTGCAGATCGCCTGGGAACTGCACAGAGCTCCCAATCCCAGCGGGAGCATCAGGTAAACAGGCCAGCTTCCCGGCCTGTGTTCCAGAGCCTGCGGATCGCCTGGGAACTGCACAGAGCTCCCAATCCCGGCAGGAGCATCAGGTAAACAGGCCAGCTTCCCGGCCT
GTGTTCCAGAGCCTGCAGATCGCCTGGGAACTGCACAGAGCTCCCAATCCCGGCGGGAGCAAAGCACCCCAGACTCCAGATCTTCTCTCCCACCAACGATTCCAGGAAAGGAGAGGAGGCTGGGAGTACAGGGAGGTGGCTGTCCCTCTGGCATCTTCAGGTAAACAGGCCAGCTTCCCGGCCTGAGTTCCGGGGCCTGCAGATTGCCTGGGAACTGCACAGAGCT
CCCAATCCCGGCGGGAGCGTCGGGTAACAGGCCAGCTTCCCGGCCTGTGTTCCGGAGCCTGCAGATCGCCTGGGAGCTGCACAGGGCTCCCAATCCCGGCGGGAGCATCAGGTAAACAGGCCAGCTTCCTGGCCTGTGTTCCAGAGCCTGCAGATCGCCTGGGAACTGCACAGAGCTCCCAATACCGGCGGGAGCAAAGCACCCCAGACTCCAGGTCTTCTCTCC
CACCAACGATTCCAGGAAAGGAGAGGAGGCTGGGAGTGCAGGGGGGTGGCTGTCCCTCTGGCATCATCAGGTAAACGGGCCAGCTTCCAGGCCTGAGTTCCAGAGCCTGCAGATCGCCTGGGAACTGCACAGAGCTCCCAATCCCAGCGGGAGCATCAGGTAAACAGGCCAGCTTCCCGGCCTGTGTTCCAGAGCCTGCGGATCGCCTGGGAACTGCACAGAGCTC
CCAATCCCGGCAGGAGCATCAGGTAAGCAGGCCAGCTTCCCGGCCTGTGTTCCAGAGCCTGCAGATCGCCTGGGAACTGCACAGAGCTCCCAATCCCGGCGGGAGCATCAGGTAAACAGGACAGCTTCCCGGCCTGTGTTCCAGAGCCTGCAGATCGCCTGGGAACTGCACAGAGCTCCCAATCCCGGCGGGAGCAAAGCACCCCAGACTCCAGATCTTCTCTCCC
ACCAACGATTCCAGGAAAGGAGAGGAGGCTGGGAGTACAGGGAGGTGGCTGTCCCTCTGGCATCGTCAGGTAAACAGGCCAGCTTCCCGGCCTGAGTTCCGGGGCCTGCAGATCGCCTGGGAACTGCACAGAGCTCCCAATCCCGGCGGGAGCATCAGGTAAACAGGCCAGCTTCCCAGCCTGTGTTCCAGAGCCTGCAGATCGCCTGGGAACTGCACAGAGCTCC
CAATCCAGGCGGGAGCGTCGGGTGAACAGGCCAGCTTCCCGGCCTGTGTTCCGGAGCCTGCAGATCGCCTGGGAGCTGCACAGGGCTCCCAATCCCGGCGGGAGCATCAGGTAAACAGGCCAGCTTCCTGGCCTGTGTTCCGGAGCCTGCAGATCGCCTGGGAACTGCACAGAGCTCCCAATACCGGCGGGAGCAAAGCACCCCAGACTCCAGGTCTTCTCTCCCA
CCAACGATTCCAGGAAAGGGGAGGAGGCTGGGAGTGCAGGGGGGTGGCTGTCCCTCTGGCATCATCAGGTAAACGGGCCAGCTTCCAGGCCTGAGTTCCAGAGCCTGCAGATCGCCTGGGGACTGCGCAGAGCTCCCAGTCCCTGCGGGAGCCAGGCACCCCAGACTCCAGATCTTCTCTCCCACCAAAGAGACCAGGAAACTACAGGAACCAGGGAGACCAGGGG
AGTAGCTGTTCCGGATCAGGTACAACAGGACCCAGCATAGGGTCCAGGGGACCAGGTAGGAGGCTAAGCGCCCCAGATTCCTGTTCTTCTTCTCTACCCACGGACCAGCATTTGGACAAGGCATAGAAGAACCACCAGATTACTGGACTTACTAGCACCCACCAAAAGGGAACTCTGGTCCCCAGACCAGGAAGGGGCTCTTTCTGCACCCACTAAAGAAGAGAT
GGGAAAGAGACAATACAAAAACACAGTCAGCAACAGAAAAACAAATATGACACCGCCAGATTCTAGAGACTCCACACCAGCAAGACCCCAATATTCCAACGCGAAGGAAGCAGAAGAGAACGATCTTAAAAGCACTTTCATGAAAATGATAGAGGCCCTAAAAGAGGATATTGGAAAATCCCTCAAAGAAATGGAAGAAAAAACAAACCAAAAAATGCAAGAAATG
GAAGAAAAAACAAACCAAAAAATGCAAGAATTGGAAGAAAAAACAAACCAAAAACGGCACGAAATGGAAGAAGAAATGAAGCAAAAAATGCAAGAAATGGAAGAAAAAGCAAAGCAAAAAATGCAAGAATTAGAAGAAGAAACATACCAAATAAGGCAGGAAATGGAAGAAGCAACAAAGCAAAAAATGCAAGAAATGGTAGAAAAAACAAAGCAAAAAATGAAAA
AAATGGAGGAAGAAACAAACCAAAAAAGGCAGGAAAGGGAAGAAGAAACAAAGCACAAAATCCAAGAAATGGAACAAAAAGCAAACCAAAAAATGCAAGAAATGGAAGAAAGAACAAACCAAAAAATTCATAAAAGGCAACAAACGGACGAACAGATAAACAGAAAATTGCAAGAAGCAATGCAAATGCTGCAAGGGTTGAAAACTGAAATAGAGACACTAAAAAA
AACACAATCTGAAGAAAGGCAGAAAATAGAAAAGCTGGACAAACAGTCAGGCACTACCAATACAAGCATTACCAACAGAATAGAAGAGATGGAAGAGAGAATCTCAAGTGTTGAAGATACATTAGGAGAAATGGATTTATCAACCAAAGAAAACCTTAAATCCAACAAATCCTTAACACAAAACATCCAGGAATTATGGGACACCATGAAAAGACCAAACCTAAGA
ATAATAGGTATAGAAGAAGGAGAAGAAATCCACCTCAAAGGGGCAGAAGGCATATTCAACAAAATCATAGAAGAAAACTTTCCCAACCTTAAGAAAGACATGCCAATGAAGGTACAAGAAGCCTACAGAACACCAAATAGAGTGGACCACAAAAAAAAATCCCCTCGCCACATAATAATCAAGACCCCAAACATACAGAATAAAGAAAGAATATTAAGAGCAGCTA
AGGAAAAAGGTCAAGTAACATATAAAGGCAGACCTATCAGAATTACACCTGATTTCTCCATGGAAACTCTGAAAGCCAGAAGGTCCTGGATAGATATTCTACCTACACTAAGAGACCATGGATGCCAGCCCAGACTACTATACCCGGCAAAGCTCTCAATCACTATAGACGGAGAAAACAAGATATTCCATGACAAAACTAGATTCAACCAATATGTGTCCACTAA
CCCAGCCCTACAGAAAATTTTGGAAGGAAAACTCCAACCCAAGGAAGCTAACTACAACCAGAAAAACATAAGCATTAGAGAATCCCTCTCTACCAACAGCCAAAAGAACAAAGGGGCGAAATTCCACCCATAATTCCACCAACAACAACCATCGCAAATCGAAGAAGGATGAGCAATCAATGGTCATTAATATCCCTCAATATTAATGGCCTTAACTCACCCATAA
AAAGACACAGACTAGCAGAATGGATTCGAAGACAGAACCCTTCCTTCTGCTGCATACAAGAAACACACCTCACCCTCAAAGACAGACATTACTTGAGAGTTAAAGGTTGGGAAAAAATTTTTCAATCAAATGGACCCAAGAAACAAGCAGGGGTAGCAATCCTAATATCTAACAAACTAGACTTTAAAATAAAATCAATCAAAAGAGATGAAGGAGGTCATTACAT
ACTCATCACAGGTGAAACCCACCAAGACGAAGTGTCTATCCTGAACATCTATGCCCCCAATACAAAGGCATCCACATTCGTTAAAGAAACATTATTAGAACTTAAATCTCATTTAAAGCCCCACACAGTTATAGTGGGAGATTTCAACACACCACTCTCACCACTAGACAAGAATACCAGACAGGAACTCAACAAAGAAGCAAAGGGTCTAATAGAAGTAATGACC
CAACTGGGATTAACAGATATCTATAGAACATTTCATCCACATAAAAAAGAATATACCTTCTTCTCACCACCACATGGAACCTTCTCAAAATCGACCACATACTTGGCAACATATCAAACCTCAGCAGCTACAAAAAAATTGGAATAACTCCATGCATCTTATCAGACCACCATGCCTTAAAATTAGAATTCAACAACAACACAAATTGCAGAAAACCTACAAACT
CATGGAAATTGAATAATGCTCAACTGCACCATCCCTGGGTCAAAGAGGAAATAAAAAAAGAAATTAAAGACTTCCTCGAACTCAATGAGAATGAAGACACAACATACCCAAACCTATGGGACACTTTGAAAGCAGTGCTATGGGGGAAGTTCATAGCACTAAGTGCTCACATGAAGAAACAGGAAAACAGTTATACCAGAGAATTAACATCACAGCTAAAAGCTCT
AGAACACAAAGAAGCAAACTCCCCCAGGAGGAGTAGACGCCAGGAAATAATCAAATTGAGGGCTGAAATCAACAAAGTAGAAACCAGGAAAACAATACAAAGAATCAATGCAACGAAGAGTTGGTTCTTTGAGAAAATCAACAAGATAGACAAACCTTTGTCCAAACTTACCAAACAGCAGAGAATGAACACGCAAATTAACAAAATCAGAAATGAAAAGGGGGAC
ATAACAACAGACTCTGAGGAAATCCAGAGAATCATTAGGTCATACTTTGAAAACCTGTACTCCACAAAATTCGAAATTTAAAGGAAATGGATGACTTCCTGGACAGCTATCATTTGCCAAAACTAAATCAAGAACAGATAAGCAATTTAAATAGACCTATAACCCCTATGGAAATAGAAGCTGTCATCAAAAGTCTCCCAACCAAAAAAAGTCCAGGTCCAGATG
GCCTCAGTGCAGAATTCTACCACAAATTCAAAGAACAGCTTATACCAATTCTTCTCAAAGTGTTCCACACAATAGAAGCAGAAGGGTCTTTGCCAAACTCTTTCTATGAGGCTTCAATAACCTTGATCCCCAAGCCACACAAAGATACAACCAAGAAAGAGAACTTCAGACCAATATCCCTCATGAACATTGATGCAAAAATACTCAATAAAATACTGGCAAATCG
GATCCAAGAACACATCAAAACAATCATTCACCACGATCAAGTAGGTTTCATCCCAGGGATGCAGGGATGGTTCAACATACGAAAATCCATCAACGTAATCCACCATATAAACAAGCTGAAAAACAAAAATCACATGATCATCTCACTAGATGCTGAAAAAGCCTTTGACAAAATCCAACATCCCTTCATGATAAAGGTCTTGGAGAGATCTGGGATAACAGGACA
TACTTAAACATAATAAAAGCCATATACAGTAAACCAACAGCCAACATCAAACTAAATGGACAGAAACTCAACGCAATTCCTCTAAAATCAGGAACAAAACAAGGCTGTCCACTCTCTCCATACCTTTTCAACATGGTACTTGAAGTTCTAGCTATAGCCATACGACAACAAAGGGGGATCAAGGGGATACAAATTGGAAGGAAGAAGTCAAACTTTCACTGTTTG
CAGACGATATGATAGTTTATATCAGTGACCCAAAGAACTCTACCAGGGAACTCTTACAGCTGATTAACACCTTCAGCAAAGTGGCAGGTTACAAGATCAACTCAAGAAAGTCAGTAGCCCTACTATATACAGATGATAAAAGGGCTGAAAGAGAAATCAGAGAAACATCACCCTTTACAATTGCCACAAACAACATAAAATACCTTGGGGTAACGCTAACCAAACA
AGTGAAAGACCTGTATCGTAAGAACTTTGAATCTTTAAAGAAAGAAATTAAAGAAGCCCTCAGAAAATGGAAAGATCTCCCATGCTCTTGGATAGGTAGGATCAATGTAGTAAAAATGGCAATCCTGCCAAAAGCAATCTACAGATTCAATGCTATCCCCATTAAAATCCCAGCACAATTCTTCACAGACCTTGAAAGAACAATAATCAATTTTGTATGGAAAAAC
AAAAGACCCAGGATAGCCAAAACAACCCTGTTCAACAAGGCAACGTCCGGAGGCATCACTATCCCTGACCTCAAGCTCTATTATAGAGCTATAGTCCTGAAAACAGCTTGGTATTGGCACAAAAATAGACAGGCAGACCAATGGAATCGAATTGAAAATCCGGGTATTAACCCACACACGTACGAACATCTGATCTTCGACAAAGAAGCTAAAAATATACAATGGA
ATAAAGAAAGCATCTTCAACAAATGGTGCTGGCATAATTGGAGACTGGCATGTAGAAAAATGCGGATCGATCCATGTTTATCGCCATGCACAAAACTTAAGTCCAAATGGATCAAAGACCTCAACATAAATCCAGCCACACTGAACCTCTTAGAAGAGAAAGTGGGTGGTACCCTTGAAAGAATTGGTACAGGAGACAGCTTCCTGAACATAACACCAGTAGCACA
GACAGTGAGATCAACAATTAATAAATGGGACCTACTGAAACTGAGAAGCTTCTGTAAGGCAAAGGACACAGTCAGCAAGACAAAAAGGCAGCCCACAGAATGGGAAAAAATATTCACTAATCCCACATCTGACAGAGGGTTGATCTCCAAAATTTACAAAGAACTCAAGAAGCAAGTCTCCAAAACAGCAAAGGACCCAATTAAGAAGTGGGCTGCAGAACTAAAT
AGAGAGTTTTCAATAGAGGAGTCTAAAATGGCTGAGAGGCACTTGAGAAAGTGCTCAACATCCTTAGCCATCCGGGAAATGCAAATCAAAGCAACTCTGAGATACCATCTCACTCCTGTCAGAATGGCTAAAATCAAAAACACCAATGACAATTTATGCTGGAGAGGATGTGGTGAAAGGGGGACACTCCTCCATTGCTGGTGGGAGTGCCAACTGGTTCAGCCAC
TTTGGAAAGCAGTATGGAGATTCCTCAGAAAATGGGAATAAACCTACCACAAGACCCAGCAACTGCACTCTTAGGCATATATCCAAAGATGCCCATTCATATAACAAAGATGTCTGTTCCACTATGTTCATAGCAGCTTTATTTGTAATAGCCAGAACTTGGAAGCAACCTAGATGTCCCTCACCCGAAGAATGGATAGAGAAAATGTGGTACATCTACACAAT
GGAGTACTATTCAGCAGAAAAAAATAATGGAATCATGAGATTCGCAGGCAAATGGATGACACTAGAAGAAACCATCCTGAGTGAGGTAACCCAGTCACAAAAAGAGAAACATGGCATGTACTCTCTCATATATGGATTTTAGACATAGATCTGGGGAGTGCCAGCCTACAATCCTATTGCCAGAGAGTCTGGGAAACAGAACTTCATTTACTGGCTGGTTAAGTA
CCGAGCTGAACTCAAGAAAGCCAGTATCAGAGAGGGAGGACTGATGATCAAAGGGATTAAGACTGGGCTGGTTGAACCCCCAGAAACAGCTGACCAGACAAAGGTTAACTCTTGAATCCCAGGCATCAGCCTGGGAATCCAGCATGGGACTGATCAAGACCCCCTGAATCTGGGTGTCAGTGAGGAGGCCTAGGTAATCTATTGAGCCTTGGACAGCAGATCAGTA
CCCATCCCAAGTATACACACGGTCTCTGGACTCCCATAGAACAATGTGAGGTGCACTCTCACCCTGAACTCTTGGGGAAGGGTCTAGGCCCTGCCCTATATGAAATGACAGACTATGGACACCCCCATGGAGGGCCTCACCCTCCCTGGGGGAATGAGGAGATAACGGGGGGGGGATGGGGGGAGGGCACGGGAGGAGGGGAGGGAAAGGGAGAGGGACCTCGTAC
TGACTTGTGAAGCAACCTTGTTTCTTATCTAAATTTAAAATGGAATAATAAAAAAATATTAATGAAAAAAAAAA

>Lx5.3195790
CGACAAAGCCAGATTTAACAATACGTATCCTTGATTCCAGCTCTACAGAAACTCCAGGAAGGAAAACTTAGATCCAAGGAAGATAACTACACTCACAAAGACACAGGCAATATATAATCCCACTTTACATAACACCAAAAGAAAAAGGGAGGAGAAAGCACACAAAATAACTTCACCACCAATAAATCAAGCACACACAAGATTCATCAATCAATGGTCATTGATA
TCCCTCAATGTCAATGGCCTCAACTTGCCTATAAAAAGACAAAGACTAGCAGAATGGATACGAAGACAGAACCATTCCTTCTGCTGCATACAAGAAACACATCTCAACATCACAGACAGACGGTATCTCAGGGTAAAGGGTTTGGAAAAGATTTTCCAATCAAAGGGACCCAAGAAACAAGCAGGTGTAGCAATCCTAATATCTAACAAACTAGACTTCAAATTAA
AATCAATCAAAAGAGATGAAGGACATTACATATTCATCAAAGGAAAAGCCCATCAAGATGAAGTCTCAATTTTAAACATCCATGCCCCAATTACAAAGGCACCTACATTTGTGAAAGAAACATTACTAAAGCTCAAATTGCACATAAAATCACAAACACTAATAGTGGGAGACTTCAACACTCCGCTCTCACCACTAGAGAGTTCCTCCAGACAGAAACTTAACAA
AGAAACAAAGGAACTAATAGAAGTTATTACCCACCTGGGTTTAACAGACATCTATAGAACATTCCACCCAAACACAAAGGGATAAACCTTCTCGGTGCCACATGGAACCTTCTCTAAAATAGGAAACATAGCAAATCTCCACAGAAACAAAAAAGTGGAATAACCCCCTGTATCCTCTCAGACCACCATGCATTAAAATTAGAATTCAACAATAACACAATTCGCA
GAAACCCCACAAACTCATGGAAACTGGACAATGCTCAATTGCACCATCCCTGGGTAAAGGACGAAATAAAAGAAGAAATTAAAGTCTTCCTGGAATTCAATGAGAATGAAGACACAACATATGCAAGCCTATGGGACACTTTGAAAGCAGTGCTGAGGAAAATTCATAGCACTAAGTGCCCACATGAAGAAACTGGAGAACAGTCATACTAGAGAATTAATGGC
ACAGCTGAACGTTCTAGAACAAAAGAAGCAAACTCACCCAGGAGGAACAGACTCCAGGAAATAATCAAATTGAGGGCTGAAATCAATCAAGTAGAAACAAGGAGAACACTGAGAAGAATCAATGAAACAAAGAGTTGGTTCTTTGAGAAAATCAACAAAATAGACAAACCTCTGTCCAAACTAACAAAAAGACACAGCGAGAGCATGCTAATTAACAAAATCAGA
AATGAAAACGGGGACATAACAATGGACTCTGAGGAAATCCAGAGAATCATCAGGGCATACTTTGAAAATCTGTACTCCACAAAATTCGAAACTATAAAGGAAATGGACAATTTCCTGGATAGATATCACTTGCCAAAACTAAATCAGAGCAGATTAAACAATCTAAACAGACCTATAACCCTTAATGAAATAGAGCAGTCATAAAGAAGGCTGGGAGCACAGGG
AAGTAGCTGTCCCTCTGGCATCATCAGGTAAACAGGCCAGCTTCCCGGCCTGTGTTCCAGAGCCTGCAGATCGCCTGGGAACTGCACAGAGCTCCCAATCCCGGCGGGAGCATCAGGTAAATAGGCCAGCTTCCCAGCCTGTGTTCCAGAGCCTGCAGATCGCCTGGGAACTGCACAGAGCTCCCAATCCCGGCGGGAGCATCAGGTAAATAGGCCAGCTTCCCAG
CCTGTGTTCCAGAGCCTGCAGATCGCCTGGGAACTGCACAGAGCTCCCAATCCCGGCGGGAGCATCAGGTAAACAGGCCAGCTTCCCAGCCTGTGTTCCAGAGCCTGCAGATCGCCTGGGAACTGCACAGAGCTCCCAATCCCGGCGGGAGCCAAGCACCCCAGACTCCAGATCTTCTCTCCCACCAAAGAGACCAGGAAACTAGAGGAGCCAGGGAGACCAGGGG
AGTAGCAGTTCCGGATCAGGTACAACAGGACCCAGCATAGGGTCCAGGGGACCAGGTAGGAGGCTAAGCGCCCCAGATTCCAGTTCTTCTCTACCCACGGACCAGCATTTGGACAAGGCATAGAAGAACCACCAGATTACTGGACTTACTAGCACCCACCAAAAGGGAACTCTGGTCCCCAGACCAGGAAGGGGCTCTTTCTGCACCCACTAGAAGAAGAGATGGG
AAAGAGACAATACAAAAACACAGTCAGCAACAGAAAAACAAATATGACACCGCCAGATTCTAGAGACTCCACACCAGCAAGACCCCAATATTCCAACGCGAAGGAAGCAGAAGAGAACGACCTTAAAAGCACTTTCATGAAAATGATAGAGGCCCTAAAAGAGGATATTGGAAAATCCCTCAAAGAAATGGAAGAAAAAACAAACCAAAAAATGCAAGAAATGGAA
GAAAAAACAAACCAAAAAATGCAAGAATTGGAAGAAAAAACAAACCAAAAAAGGCACGAAATGGAAGAAGAAATGACGCAAAAAATGCAAGAAATGGAAGAAAAAGCAAAGCAAAAAATGCAAGAATTAGAAGAAGAAACATACCAAATAAGGCAGGAAATGGAAGAAGAAACAAAGCAAAAAATGCAAGAAATGGTAGAAAAAACAAAGCAAAAAATGAAAAAAA
TGGAGGAAGAAACAAACCAAAAAAGGCAGGAAATGGAAGAAGAAACAAAGCACAAAATCCAAGAAATGGAACAAAAAGCAAACCAAAAAATGCAAGAAATGGAAGAAAGAACAAACCAAAAAATGCATAAAAGGCAACAAACGGACAAACAGATAAAAAGAAAATTGCAAGAAGCAATGCAAACGCTGCAAGGGTTGAAAACTGAAATAGAGTCACTAAAAAAAAC
ACAATCTGAAGAAAGGCAGAAAATAGAAAAGCTGGACAAACAGTCAGGCACTACCAATACAAGCATTACCAACAGAATAGAAGAGATGGAAGAGAGAATCTCAAGTGTTGAAGATACATTAGGAGAAATGGATTTATCAACCAAAGAAAACATTAAATCCAACAAATCCTTAACACAAAACATCCAGGAATTATGGGACACCATGAAAAGACCAAACCTAAGAATA
ATAGGTATAGAAGAAGGAGAAGAAATCCACCTCAAAGGGGCAGAAGGCATATTCAACAAAATCATAGAAGAACACTTTCCCAACCTTAAGAAAGACATGCCAATGAAGGTACAAGAAGCCTACAGAACACCAAATAGAGTGGACCACAAAAAAAAATCCCCTCGCCACATAATAATCAAGACCCCAAACATACAGAATAAAGAAAGAATATTAAGAGCAGCTAAGG
AAAAAGGTCAAGTAACATATAAAGGCAGACCTATCAGAATTACACCTGATTTCTCCATGGAAACTCTGAAAGCCAGAAGGTCCTGGATAGATATTCTACCTACACTAAGAGACCATGGATGCCAGCCCAGACTACTATACCCGGCAAAGCTCTCAATCACTATAGACGGAGAAAACAAGATATTCCATGACAAAACTAGATTCAACGAATATGTGTCCACTAACCC
AGCCCTACAGAAAATTTTGGAGGAAAACTCCAACCCAAGGAAGCTAACTACAACCAGAAAAACATAAGCATTAGAGACTCCCTCTCTACCAACAGCCAAAAGAACAAAGGGGTGAAATTCCACACATAATTCCACCAACAACAACCATCGCAAATCGAAGAAGGATGAGCAATCAATGGTCATTAATATCCCTCAATATTAACAGCCTTAACTCACCCATAAAAA
GACACAGACTAGCAGAATGGATTCGAAGACAGAACCCTTCCTTCTGCTGCATACAAGAAACACACCTCACCCTCAAAGACAGACATTACTTGAGAGTTAAAGGTTGGGAAAAAATTTTTCAATCAAATGGACCCAAGAAACAAGCAGGGGTAGCAATCCTAATATCTAACAAACTAGACTTTAAAATAAAATCAATCAAAAGAGATGAAGGAGGTCATTACATACT
CATCACAGGTGAAACCCACCAAGATGAAGTGTCTATCCTGAACATCTATGCCCCCAATACAAAGGCATCCACATTCATTAAAGAAACATTATTAGAACTTAAATCTCATCTAAAGCCCCACACAGTTATAGTGGGAAATTTCAACACACCACTCTCACCACTAGACAAGAATACCAGACAGAAACTCAACAAAGAAGCAAAGGGTCTAATAGAAGTAATGACCCAA
CTGGGATTAACAGATATCTATAGAACATTTCATCCACATAAAAAAGAATATACCTTCTTCTCACCACCACATGGAACCTTCTCAAAATCGACCACATACTTGGCAACATATCAAACCTCAGCAGCTACAAAAAAATTGGAATAACCCCATGCATCTTATCAGACCACCATGCCTTAAAATTAGAATTCAACAACAACACAAACTGCAGAAAACCTACAAACTCAT
GGAAATTGAATAATGCTCAACTGCACCATCCCTGGGTCAAAGAGGAAATAAAAAAAGAAATTAAAGACTTCCTCGAATTCAATGAGAATGAAGACACAACATACCCAAACCTATGGGACACTTTGAAAGCAGTGCTACGGGGGAAGTTCATAGCACTAAGTGCTCACATGAAGAAACAGGAAAACAGTTATACCAGAGAATTAACATCACAGCTAAAAGCTCTAGA
ACACAAAGAAGCAAACTCCCCCAGGAGGAGTAGACGCCAGGAAATAATCAAATTGAGGGCTGAAATCAACAAAGTAGAAACCAGGAAAACAATACAAAGAATCAATGCAACGAAGAGTTGGTTCTTTGAGAAAATCAACAAGATAGACAAACCTTTGTCCAAACTTACCAAACAGCAGAGAGTGAACACGCAAATTAACAAAATCAGAAATGAAAAGGGGGACATA
ACAACAGACTCTGAGGAAATCCAGAGAATCATTAGGTCATACTTTGAAAACCTGTACTCCACAAAATTCGAAATTTAAGGAAATGGATGACTTCCTGGACAGCTATCATTTGCCAAAACTAAATCAGAACAGATAAGCAATTTAAATAGACCTATAACCCCTATGGAAATAGAAGCTGTCATCAAAAGTCTCCCAACCAAAAAAAGTCCAGGTCCAGATGGCC
TCACTGCAGAATTCTACCAGAAATTCAAAGAACAGCTTACACCAATTCTTCTCAAAGTGTTCCACACAATAGAAGCAGAAGGGTCTTTGCCAAACTCTTTCTATGAGGCTTCAATACCTTGATCCCCAAGCCACACAAAGATACAACCAGAAAGAGAACTTCAGACCAATATCCCTCATGAACATTGATGCAAAAATACTCAATAAAATACTGGCAAATCGGAT
CCAAGAACACATCAAAACAATCATTCACCACGATCAAGAGGGTTTCATCCCAGGGATGCAGGGATGGTTCAACATACGAAAATCCATCAACATAATCCACCATATAAACAAGCTGAAAAACAAAAATCACATGGTCATCTCACTAGATGCTGAAAAAGCCTTTGACAAAATCCAACACCCCTTCATGATAAAGGTCTTGGAGAGATCTGGGATAACAGGACATAC
TTAAACATAATAAAAGCCATATACAGTAAACCAACAGCCAACATCAAACTAAATGGGCAGAAACTCAACGCAATTCCTCTAAAATCAGGAACAAAACAAGGCTGTCCACTCTCTCCATACCTTTTCAACATGGTACTTGAAGTTCTAGCTATAGCCATACGACAACAAAGGGGGATCAAGGGGATACAAATTGGAAGGAAGAAGTCAAACTTTCACTGTTTGCAG
ACGATATGATAGTTTATATCAATGACCCAAAGAACTCTACCAGGGAACTCTTACAGCTGATTAACACCTTCAGCAAAGTGGCAGGTTACAAGATCAACTCAAGAAAGTCAGTAGCCCTACTATATACAGATGATAAAAGGGCTGAAAGAGAAATCAGAGAAACATCACCCTTTACAATTGCCACAAACAACATAAAATACCTTGGGGTAACGCTAACCAAACAAGT
GAAAGACCTGTATCGTAAGAACTTTGAATCTTTAAAGAAAGAAATTAAAGAAGCCCTCAGAAAATGGAAAGATCTCCCATGCTCTTGGATAGGTAGGATCAATGTAGTAAAAATGGCAATCCTGCCAAAAGCAATCTACAGATTCAATGCTATCCCCATTAAAATCCCAGCACAATTCTTCACAGACCTTGAAAGAACAATAATCAATTTTGTATGGAAAAACAAA
AGACCCAGGATAGCCAAAACAACCCTGTTCAACAAGGCAACGTCCGGAGGCATCACTATCCCTGACCTCAAGCTCTATTATAGAGCTATAGTCCTGAAAACAGCTTGGTATTGGCACAAAAATAGACAGGCAGACCAATGGAATCGAATTGAAAATCCGGGTATTAACCCACACACGTACGAACATCTGATCTTTGACAAAGAAGCTAAAAATATACAATGGAATA
AAGAAAGCATCTTCAACAAATGGTGCTGGCATAATTGGAGACTGGCATGTAGAAAAATGCGGATCGATCCATGTTTATCGCCATGCACAAAACTTAAGTCCAAATGGATCAAAGACCTCAACATAAATCCAGCCACACTGAACCTCTTAGAAGAGAAAGTGGGTGGTACCCTTGAACGAATTGGTACAGGAGACAGCTTCCTGAACATAACTCCAGTAGCACAGAC
AGTGAGATCAACAATTAATAAATGGGACCTACTGAAACTGAGAAGCTTCTGTAAGGCAAAGGACACAGTCAGCAAGACAAAAAGGCAGCCCACAGAATGGGAAAAAATATTCACTAATCCCACATCTGACAGAGGGTTGATCTCCAAAATTTACAAAGAACTCAAGAAGCAAGTCTCCAAAACAGCAAAGGACCCAATTAAGAAGTGGGCTGCAGAACTAAATAGA
GAGTTTTCAATAGAGGAGTCTAAAATGGCTGAGAGGCACTTGAGAAAGTGCTCAACATCCTTAGCCATCCGGGAAATGCAAATCAAAGCAACTCTGAGATACCATCTCACTCCTGTCAGAATGGCTAAAATCAAAAACACCAATGACAATTTATGCTGGAGAGGATGTGGTGAAAGGGGGACACTCCTCCATTGCTGGTGGGAGTGCCAACTGGTTCAGCCACTTT
GGAAAGCAGTATGGAGATTCCTCAGAAAATGGGAATAAACCTACCACAAGACCCAGCAACTGCACTCTTAGGCATATATCCAAAGATGCCCATTCATATAACAAAGATGTCTGTTCCACTATGTTCATAGCAGCTTTATTTGTAATAGCCAGAACTTGGAAGCAACCTAGATGTCCCTCACCCGAAGAATGGATAGAGAAAATGTGGTACATCTACACAATGGA
GTACTATTCAGCAGAAAAAAATAATGGAATCATGAGATTCGCAGGCAAATGGATGACACTAGAAGAAACCATCCTGAGTGAGGTAACCCAGTCACAAAAAGAGAAACATGGCATGTACTCTCTCATATATGGATTTTAGACATAGATCTGGGGAGTGCCAGCCTACAATCCCATTGCCAGAGAGTCTGGGAAACAGAACTTCATTTACTGGCTGGTTAAGTACTG
AGCTGAACTCAAGAAAGCCAGTATCAGAGAGGGAGGACTGATGATCAAAGGGATTAAGACTGGGCTGGTTGAACCCCCAGAAACAGCTGACCAGACAAAGGTTAACTCTTGAATCCCAGGCATCAGCCTGGGAATCCAGCATGGGACTGATCAAGACCCCCTAAATCTGGGTGTCAGTGAGGAGGCCTAGGGAATCTATTGAGCCTTGGACAGCAGATCTGTACCC
ATCCCAAGTATACACACGGTGTCTGGACTCCCATAGAACAATGTGAGGTGCACTCTCACCCGAACTCTTGGGGAAGGTCTAGGCCCTGCCCTATATGAAATGACAGACTATGGACACCCCCATGGAGGGCCTCACCCTCCCTGGGGGAATGAGGGGATAACGGGGGGGATGGGGGGAGGGCACGGGAGGAGGGGAGGGAAAGGGAGAGGGACCTCGTACTGACTTG
TGAAGCAACCTTGTTTCTTATCTAAATTTAAAATGGAAAATAAAAAAATATTAATAAAAAAAGACAAAAAAA

>Lx5b.3358061
TGAGTTCCAGAGCCTGCAGATCGCCTGGGAACTGCACAGAGCTCCCAATCCCGGCGGGAGCATCAGGTAAACAGGCCAGCTTCCCGGCCTGTGTTCCAGAGCCTGCAGATCGCCTGGGAACTGCACAGAGCTCCCAATCCCGGCGGGAGCATCAGGTAAACAGGCCAGCTTCCCGGCCTGTGTTCCAGAGCCTGCAGATCGCCTGGGAACTGCACAGAGCTCCCAA
TCCCGGCGGGAGCAAAGCACCCCAGACTCCAGATCTTCTCTCCCACCAACGATTCCAGGAAAGGAGAGGAGGCTGGGAGTACAGGGAAGTAGCTGTCCCTCTGGCATCATCAGGTAAACAGGCCAGCTTCCCGGCCTGAGTTCCAGAGCCTGCAGATCACCTGGGAACTGCACAGAGCTCCCAATCCCGGCGGGAGCATCAGGTAAACAGGCCAGCTTCCCGGCCT
GTGTTCCAGACCCTGCAGATCGCCTGGGAACTGCACAGAGCTCCCAATCCCGGCGGGAGCAAAGCACCCCAGACTCCAGATCTTCTCTCCCACCAACGATTCCAGGAAAGGAGAGGAGGCTGGGAGTACAGGGAAGTAGCTGTCCCTCTGGCATCATCAGGTAAACAGGCCAGCTTCCCGGCCTGAGTTCCAGAGCCTGCAGATCGCCTGGGAACTGCACAGAGCT
CCCAATCCCGGCGGGAGCATCAGGTAAACAGGCCAGCTTCCCGGCCTGTGTTCCAGAGCCTGCAGATCACCTGGGAACTGCACAGAGCTCCCAATCCCGGCGGCAGCATCAGGTAAACAGGCCAGCTTCCCGGCCTGTGTTCCAGAGCCTGCAGATCGCCTGGGAACTGCACAGAGCTCCCAATCCCGGCGGGAGCATCAGGTAAACAGGCCAGCTTCCTGGCCTG
TGTTCCAGACCCTGCAGATCGCCTGGGAACTGCACAGAGCTCCCAATACCGGCGGGAGCAAAGCACCCCAGACTCCAGATCTTCTCTCCCACCAACGATTCCAGGAAAGGAGAGGAGGCTGGGAGTACAGGGAAGTAGCTGTCCCTCTGGCATCATCAGGTAAACAGGCCAGCTTCCCGGCCTGAGTTCCAGAGCCTGCAGATCGCCTGGGAACTGCACAGAGCTC
CCAATCCCGGCGGCAGCATCAGGTAAACAGGCCAGCTTCCCGGCCTGTGTTCCAGAGCCTGCAGATCGCCTGGGAACTGCACAGAGCTCCCAATCCCGGCGGGAGCATCAGGTAAACAGGCCAGCTTCCCGGCCTGTGTTCCAGACCCTGCAGATCGCCTGGGAACTGCACAGAGCTCCCAATCCCGGCGGGAGCAAAGCACCCCAGACTCCAGATCTTCTCTCCC
ACCAACGATTCCAGGAAAGGAGAGGAGGCTGGGAGTACAGGGAAGTAGCTGTCCCTCTGGCATCATCAGGTAAACAGGCCAGCTTCCCGGCCTGAGTTCCAGAGCCTGCAGATCGCCTGGGAACTGCACAGAGCTCCCAATCCCGGCGGGAGCATCAGGTAAACAGGCCAGCTTCCCGGCCTGTGTTCCAGAGCCTGCAGATCGCCTGGGAACTGCACAGAGCTCC
CAATCCCGGCGGGAGCATCAGGTAAACAGGCCAGCTTCCAGGCCTGTGTTCCAGAGCCTGCAGATCGCCTGGGAACTGCACAGAGCTCCCAATCCCGGCGGGAGCATCAGGTAAACAGGCCAGCTTCCTGGCCTGTGTTCCAGACCCTGCAGATCGCCTGGGAACTGCACAGAGCTCCCAATACCGGCGGGAGCAAAGCACCCCAGACTCCAGATCTTCTCTCCCA
CCAACGATTCCAGGAAAGGAGAGGAGGCTGGGAGTACAGGGAAGTAGCTGTCCCTCTGGCATCATCAGGTAAACAGGCCAGCTTCCCAGCCTGAGTTCCAGAGCCTGCAGATCGCCTGGGAACTGCACAGAGCTCCCAATCCCGGCGGGAGCCAAGCACCCCAGACTCCAGATCTTCTCTCCCACCAAAGAGACCAGGAAACTACAGGAACCAGGGAGACCAGGGG
AGTAGCTGTTCCGGATCAGGTACAACAGGACCCAGCATAGGGTCCAGGGGACCAGGTAGGAGGCTAAGCGCCCCAGATTCCTGTTCTTCTTCTCTACCCACGGACCAGCATTTGGACAAGGCATAGAAGAACCACCAGATTACTGGACTTACTAGCACCCACCAAAAGGGAACTCTGGTCCCCAGACCAGGAAGGGGCTCTTTCTGCACCCACTAAAGAAGAGAT
GGGAAAGAGACAATACAAAAACACAGTCAGCAACAGAAAAACAAATATGACACCGCCAGATTCTAGAGACTCCACACCAGCAAGACCCCAATATTCAAATGCGAAGGAAGCAGAAGAGAACGATCTTAAAAGCACTTTCATGAAAATGATAGAGGCCCTAAAAGAGGATATTGGAAAATCCCTCAAAGAAATGGAAGAAAAAACAAACCAAAAAATGCAAGAAATG
GAAGAAAAAACAAACCAAAAAATGCAAGAATTGGAAGAAAAAACAAACCAAAAACGGCACGAAATGGAAGAAGAAATGAAGCAAAAAATGCAAGAAATGGAAGAAAAAGCAAAGCAAAAAATGCAAGAATTAGAAGAAGAAACATACCAAATAAGGCAGGAAATGGAAGAAGCAACAAAGCAAAAAATGCAAGAAATGGTAGAAAAAACAAAGCAAAAAATGAAAA
AAATGGAGGAAGAAACAAACCACAAAAGGCAGGAAAGGGAAGAAGAAACAAAGCACAAAATCCAAGAAATGGAACAAAAAGCAAACCAAAAAATGCAAGAAATGGAAGAAAGAACAAACCAAAAAATTCATAAAAGGCAACAAACGGACGAACAGATAAACAGAAAATTGCAAGAAGCAATGCAAATGCTGCAAGGGTTGAAAACTGAAATAGAGACACTAAAAAA
AACACAATCTGAAGAAAGGCAGAAAATAGAAAAGCTGGACAAACAGTCAGGCACTACCAATACAAGCATTACCAACAGAATAGAAGAGATGGAAGAGAGAATCTCAAGTGTTGAAGATACATTAGGAGAAATGGATTTATCAACCAAAGAAAACCTTAAATCCAACAAATCCTTAACACAAAACATCCAGGAATTATGGGACACCATGAAAAGACCAAACCTAAGA
ATAATAGGTATAGAAGAAGGAGAAGAAATCCACCTCAAAGGGGCAGAAGGCATATTCAACAAAATCATAGAAGAAAACTTTCCCAACCTTAAGAAAGACATGCCAATGAAGGTACAAGAAGCCTACAGAACACCAAATAGAGTGGACCACAAAAAAAAATCCCCTCGCCACATAATAATCAAGACCCCAAACATACAGAATAAAGAAAGAATATTAAGAGCAGCTA
AGGAAAAAGGTCAAGTAACATATAAAGGCAGACCTATCAGAATTACACCTGATTTCTCCATGGAAACTCTGAAAGCCAGAAGGTCCTGGATAGATATTCTACCTACACTAAGAGACCATGGATGCCAGCCCAGACTACTATACCCGGCAAAGCTCTCAATCACTATAGACGGAGAAAACAAGATATTCCATGACAAAACTAGATTCAACCAATATGTGTCCACTAA
CCCAGCCCTACAGAAAATTTTGGAAGGAAAACTCCAACCCAAGGAAGCTAACTACAACCAGAAAAACATAAGCATTAGAGAATCCCTCTCTACCAACAGCCAAAAGAACAAAGGGGCGAAATTCCACCCATAATTCCACCAACAACAACCATCGCAAATCGAAGAAGGATGAGCAATCAATGGTCATTAATATCCCTCAATATTAATGGCCTTAACTCACCCATAA
AAAGACACAGACTAGCAGAATGGATTCGAAGACAGAACCCTTCCTTCTGCTGCATACAAGAAACACACCTCACCCTCAAAGACAGACATTACTTGAGAGTTAAAGGTTGGGAAAAAATTTTTCAATCAAATGGACCCAAGAAACAAGCAGGGGTAGCAATCCTAATATCTAACAAACTAGACTTTAAAATAAAATCAATCAAAAGAGATGAAGGAGGTCATTACAT
ACTCATCACAGGTGAAACCCACCAAGACGAAGTGTCTATCCTGAACATCTATGCCCCCAATACAAAGGCATCCACATTCGTTAAAGAAACATTATTAGAACTTAAATCTCATCTAAAGCCCCACACAGTTATAGTGGGAGATTTCAACACACCACTCTCACCACTAGACAAGAATACCAGACAGGAACTCAACAAAGAAGCAAAGGGTCTAATAGAAGTAATGACC
CAACTGGGATTAACAGATATCTATAGAACATTTCATCCACATAAAAAAGAATATACCTTCTTCTCACCACCACATGGAACCTTCTCAAAATCGACCACATACTTGGCAACATATCAAACCTCAGCAGCTACAAAAAAATTGGAATAACTCCATGCATCTTATCAGACCACCATGCCTTAAAATTAGAATTCAACAACAACACAAAGTGCAGAAAACCTACAAACT
CATGGAAATTGAATAATGCTCAACTGCACCATCCCTGGGTCAAAGAGGAAATAAAAAAAGAAATTAAAGACTTCCTCGAACTCAATGAGAATGAAGACACAACATACCCAAACCTATGGGACACTTTGAAAGCAGTGCTACGGGGGAAGTTCATAGCACTAAGTGCTCACATGAAGAAACAGGAAAACAGTTATACCAGAGAATTAACATCACAGCTAAAAGCTCT
AGAACACAAAGAAGCAAACTCCCCCAGGAGGAGTAGACGCCAGGAAATAATCAAATTGAGGGCTGAAATCAACAAAGTAGAAACCAGGAAAACAATACAAAGAATCAATGCAACGAAGAGTTGGTTCTTTGAGAAAATCAACAAGATAGACAAACCTTTGTCCAAACTTACCAAACAGCAGAGAGTGAACACGCAAATTAACAAAATCAGAAATGAAAAGGGGGAC
ATAACAACAGACTCTGAGGAAATCCAGAGAATCATTAGGTCATACTTTGAAAACCTGTACTCCACAAAATTCGAAATTTAAAGGAAATGGATGACTTCCTGGACAGCTATCATTTGCCAAAACTAAATCAAGAACAGATAAGCAATTTAAATAGACCTATAACCCCTATGGAAATAGAAGCTGTCATCAAAAGTCTCCCAACCAAAAAAAGTCCAGGTCCAGATG
GCCTCAGTGCAGAATTCTACCACAAATTCAAAGAACAGCTTATACCAATTCTTCTCAAAGTGTTCCACACAATAGAAGCAGAAGGGTCTTTGCCAAACTCTTTCTATGAGGCTTCAATAACCTTGATCCCCAAGCCACACAAAGATACAACCAAGAAAGAGAACTTCAGACCAATATCCCTCATGAACATTGATGCAAAAATACTCAATAAAATACTGGCAAATCG
GATCCAAGAACACATCAAAACAATCATTCACCACGATCAAGTAGGTTTCATCCCAGGGATGCAGGGATGGTTCAACATACGAAAATCCATCAACGTAATCCACCATATAAACAAGCTGAAAAACAAAAATCACATGATCATCTCACTAGATGCTGAAAAAGCCTTTGACAAAATCCAACATCCCTTCATGATAAAGGTCTTGGAGAGATCTGGGATAACAGGACA
TACTTAAACATAATAAAAGCCATATACAGTAAACCAACAGCCAACATCAAACTAAATGGACAGAAACTCAACGCAATTCCTCTAAAATCAGGAACAAAACAAGGCTGTCCACTCTCTCCATACCTTTTCAACATGGTACTTGAAGTTCTAGCTATAGCCATACGACAACAAAGGGAGATCAAGGGGATACAAATTGGAAGGAAGAAGTCAAACTTTCACTGTTTG
CAGACGATATGATAGTTTATATCAGTGACCCAAAGAACTCTACCAGGGAACTCTTACAGCTGATTAACACCTTCAGCAAAGTGGCAGGTTACAAGATCAACTCAAGAAAGTCAGTAGCCCTACTATATACAGATGATAAAAGGGCTGAAAGAGAAATCAGAGAAACATCACCCTTTACAATTGCCACAAACAACATAAAATACCTTGGGGTAACGCTAACCAAACA
AGCGAAAGACCTGTATCGTAAGAACTTTGAATCTTTAAAGAAAGAAATTAAAGAAGCCCTCAGAAAATGGAAAGATCTCCCATGCTCTTGGATAGGTAGGATCAATGTAGTAAAAATGGCAATCCTGCCAAAAGCAATTTACAGATTCAATGCTATCCCCATTAAAATCCCAGCACAATTCTTCACAGACCTTGAAAGAACAATAATCAATTTTGTATGGAAAAAC
AAAAGACCCAGGATAGCCAAAACAACCCTGTTCAACAAGGCAACGTCCGGAGGCATCAATATCCCTGACCTCAAGCTCTATTATAGAGCTATAGTCCTGAAAACAGCTTGGTATTGGCACAAAAATAGACAGGCAGACCAATGGAATCGAATTGAAAATCCGGGTATTAACCCACACACGTACGAACATCTGATCTTCGACAAAGAAGCTAAAAATATACAATGGA
ATAAAGAAAGCATCTTCAACAAATGGTGCTGGCATAATTGGAGACTGGCATGTAGAAAAATGCGGATCGATCCATGTTTATCGCCATGCACAAAACTTAAGTCCAAATGGATCAAAGACCTCAACATAAATCCAGCCACACTGAACCTCTTAGAAGAGAAAGTGGGTGGTACCCTTGAAAGAATTGGTACAGGAGACAGCTTCCTGAACATAACACCAGTAGCACA
GACAGTGAGCTCAACAATTAATAAATGGGACCTACTGAAACTGAGAAGCTTCTGTAAGGCAAAGGACACAGTCAGCAAGACAAAAAGGCAGCCCACAGAATGGGAAAAAATATTCACTAATCCCACATCTGACAGAGGGTTGATCTCCAAAATTTACAAAGAACTCAAGAAGCAAGTCTCCAAAACAGCAAAGGACCCAATTAAGAAGTGGGCTGCAGAACTGAAT
AGAGAGTTTTCAATAGAGGAGTCTAAAATGGCTGAGAGGCACTTGAGAAAGTGCTCAACATCCTTAGCCATCCGGGAAATGCAAATCAAAGCAACTCTGAGATACCATCTCACTCCTGTCAGAATGGCTAAAATCAAAAACACCAATGACAATTTATGCTGGAGAGGATGTGGTGAAAGGGGGACACTCCTCCATTGCTGGTGGGAGTGCCAACTGGTTCAGCCAC
TTTGGAAAGCAGTATGGAGATTCCTCAGAAAATGGGAATAAACCTACCACAAGACCCAGCAACTGCACTCTTAGGCATATATCCAAATGATGCCCATTCATATAACAAAGATGTCTGTTCCACTATGTTCATAGCAGCTTTATTTGTAATAGCCAGAACTTGGAAGCAACCTAGATGTCCCTCACCCGAAGAATGGATAGAGAAAATGTGGTACATCTACACAAT
GGAGTACTATTCAGCAGAAAAAAATAATGGAATCATGAGATTCGCAGGCAAATGGATGACACTAGAAGAAACCATCCTGAGTGAGGTAACCCAGTCACAAAAAGAGAAACATGGCATGTACTCTCTCATATATGGATTTTAGACATAGATCTGGGGAGTGCCAGCCTACAATCCTATTGCCAGAGAGTCTGGGAAACAGAACTTCATTTACTGGCTGGTTAAGTA
CTGAGCTGAACTCAAGAAAGCCAGTATCAGAGAGGGAGGACTGATGATCAAAGGGATTAAGACTGGGCTGGTTGAACCCCCAGAAACAGCTGACCAGACAAAGGTTAACTCTTGAATCCCAGGCATCAGCCTGGGAATCCAGCATGGGACTGATCAAGACCCCCTGAATCTGGGTGTCAGTGAGGAGGCCTAGGTAATCTATTGAGCCTTGGACAGCAGATCAGTA
CCCATCCCAAGTATACACACGGTCTCTGGACTCCCATAGAACAAAGTGAGGTGCACTCTCACCCTGAACTCTTGGGGAAGGGTCTAGGCCCTGCCCTATATGAAATGACAGACTATGGACACCCCCATGGAGGGCCTCACCCTCCCTGGGGGAATGAGGGGATAACGGGGGGGGGATGGGGGGAGGGCACGGGAGGAGGGGAGGGAAAGGGAGAGGGACCTCGTAC
TGACTTGTGAAGCAACCTTGTTTCTTATCTAAATTTAAAATGGAAAATAAAAAAATATTAATAAAAAAAAAAAAAAAAAAAAAAAAAAAAA

>Lx5.3511191
CATTTACCCAGACCTCCTACCTTATTTAAATCAAGTTCTTATGTTTAAAAAGATTCAACTGTTTTCCCCATCTTAAGATTTCACTGTCTGAGCCGGGCGTTGGTGGCGCACGCCTTTAATCCCAGCACTCGGGAGGCAGAGGCAGGCGGATCTCTGTGAGTTCGAGGCCAGCCTGGTCTACAAAGTGAGTTCCAGGACAGCCTCCAAAGCTACAGAGAAACCCTGT
CTCAAAAAACCAAAAAAAAAAAAAAGATTTCACTGTCTGTTAAAGAGACTTTTTATTCAGTATAATTTGGGTGAAAGGGATATAATTCTATAGTCTCTGTGGCGGTATGAGTTTGGCTGGGGGTGGGGCCCCTGGGAAGAACGCTGGGAAGTGGCTTTCTGGGCTCCTGGAAGCAGAGGAGGAAGATGAGCTCTACCAGACTACTTATGGGGCTTTCACAGAGGAA
TCTGGAGATGATGAGTATCAGGGAGACCAGTCTGATACAGAGGATGAGGTAGACTCTGACTTCGACATTGATGAAGGAGAGGAACCATCCAATGATGGAGAAGCAGAAGCGCCGAGAAGGAAGCGCAGAGTTGTCACCAAAGCCTATAAGGAGCCTCTAAAGAGCTTGAGGCCTCGAAAAGTCAGCACCCCAGCAAGTAGCTCTCAGAAGGCCAAAGAGGAAAAGG
CACTGCTGCCCCTGGAGCTACAGGATGACGGCTCTGACAGTCAGAAGTCTATGCATCAATCTACAGCTGAGCACACACGACAAACATTCCTTCAGTTCAGGAGTGGCAAGGACAGTCCAGGAGGTGAAAGGGTCCCCATTGCGAGCGACTGCTAATCCAGGAAGAGCTGCTCAGGGAGGCCAAGATCACAGAGAACTCAATTTATATTCACTGGAGATCTATGAA
AGGCTGGAGGCTGACAAAAGCAGGTTCACAAGAAGCGCAAGTGCCCTGGGCCAGTAATCACCGACTATTTGGTGACAGTGCCACTTGTTGGGAAGCCTGATCCCAAGGAGGAGAATGTGGACGTGGAAGGACTCGATCCTACTCCTACAGCTTCTGCACTGACTGCACATGCTGGGTCTGGACCTGCTGTCCCTCCTGCTCACGCACCTTCATCACATTTAGTGA
TGATGCGACATTTCAAGAGTGGTTTCCCAGGCCAGCTTCCTGGCCTGAGTTCCAGAGCCTGCAGATCGCCTGGGAACTGCACAGAGCTCCCAATCCCGGCAGGAGCATCAGGTAAACAGGCCAGCTTCCCGGCCTGTGTTCCAGAGCCTGCAGATCGCCTGGGAACTGCACAGAGCTCCCAATCCCGGCGGGAGCAAAGCACCCCAGACTCCAGATCTTCTCTCCC
ACCAACGATTCCAGGAAAGGAGAGGAGGCTGGGAGTACAGGGAAGTAGCTGTCCCTCTGGCATCATCAGGTAAACAGGCCAGCTTCCCGGCCTGAGTTCCAGAGCCTGCAGATCGCCTGGGAACTGCACAGAGCTCCCAATCCCGGCGGGAGCATCAGGTAAACAGGCCAGCTTCCCGGCCTGTGTTCCAGAGCCTGCAGATCACCTGGGAACTGCACAGAGCTCC

[illegible]

>Lx5.3689235

[illegible]

>Lx5.3767184

[illegible]

>Lx5.3769569

[illegible]

>Lx5.3790148

[illegible]

[illegible]

>Lx5.3827142
[illegible]

>Lx5.3814448
[illegible]

>Lx5.4078717
[illegible]

>Lx5.3874673
[illegible]

[illegible]

>Lx5.4029461
[illegible]

>Lx5.4032961
[illegible]

>Lx5.4062234
[illegible]

>Lx5.4365783
[illegible]

CAACTGGGATTAACAGATATCTATAGAACATTTCATCCACATAAAAAAGAATATACCTTCTTCTCACCACCACATGGAACCTTCTCAAAAATCGACCACATACTTGGCAACATATCAAACCTCAGCAGCTACAAAAAAATTGGAATAACTCCATGCATCTTATCAGACCACCATGCCTTAAAATTAGAATTCAACAACAACACAAATTGCAGAAAACCTACAAACT
CATGGAAATTGAATAATGCTCAACTGCACCATCCCTGGGTCAAAGAGGAAATAAAAAAAGAAATTAAAGACTTCCTCGAACTCAATGAGAATGAAGACACAACATACCCAAACCTATGGGACACTTTGAAAGCAGTGCTACGGGGGAAGTTCATAGCACTAAGTGCTCACATGAAGAAACAGGAAAACAGTTATACCAGAGAATTAACATCACAGCTAAAAGCTCT
AGAACACAAAGAAGCAAACTCCCCCAGGAGGAGTAGACGCCAGGAAATAATCAAATTGAGGGCTGAAATCAACAAAGTAGAAACCAGGAAAACAATACAAAGAATCAATGCAACGAAGAGTTGGTTCTTTGAGAAAATCAACAAGATAGACAAACCTTTGTCCAAACTTACCAAACAGCAGAGAATGAACACGCAAATTAACAAAATCAGAAATGAAAAGGGGGAC
ATAACAACAGACTCTGAGGAAATCCAGAGAATCATTAGGTCATACTTTGAAAACCTGTACTCCACAAAATTCGAAAATTTAAAGGAAATGGATGACTTCCTGGACAGCTATCATTTGCCAAAACTAAATCAAGAACAGATAAGCAATTTAAATAGACCTATAACCCCTATGGAAATAGAAGCTGTCATCAAAAGTCTCCCAACCAAAAAAAGTCCAGGTCCAGATG
GCCTCAGTGCAGAATTCTACCACAAATTCAAAGAACAGCTTATACCAATTCTTCTCAAAGTGTTCCACACAATAGAAGCAGAAGGGTCTTTGCCAAACTCTTTCTATGAGGCTTCAATAACCTTGATCCCCAAGCCACACAAAGATACAACTAAGAAAGAGAACTTCAGACCAATATCCCTCATGAACATTGATGCAAAAATACTCAATAAAATACTGGCAAATCG
GATCCAAGAACACATCAAAACAATCATTCACCACGATCAAGTAGGTTTCATCCCAGGGATGCAGGGATGGTTCAACATACGAAAATCCATCAACGTAATCCACCATATAAACAAGCTGAAAAACAAAAATCACATGATCATCTCACTAGATGCTGAAAAAGCCTTTGACAAAATCCAACATCCCTTCATGATAAAGGTCTTGGAGAGATCTGGGATAACAGGGACA
TACTTAAACATAATAAAAGCCATATACAGTAAACCAACAGCCAACATCAAACTAAATGGACAGAAACTCAACGCAATTCCTCTAAAATCAGGAACAAAACAAGGCTGTCCACTCTCTCCATACCTTTTCAACATGGTACTTGAAGTTCTAGCTATAGCCATACGACAACAAAGGGGGATCAAGGGGATACAAATTGGGAAGGAAGAAGTCAAACTTTCACTGTTTG
CAGACGATATGATAGTTTATATCAGTGACCCAAAGAACTCTACCAGGGAACTCTTACAGCTGATTAACACCTTCAGCAAAGTGGCAGGTTACAAGATCAACTCAAGAAAGTCAGTAGCCCTACTATATACAGATGATAAAAGGGCTGAAAGAGAAATCAGAGAAACATCACCCTTTACAATTGCCACAAACAACATAAAATACCTTGGGGTAACGCTAACCAAACA
AGTGAAAGACCTGTATCGTAAGAACTTTGAATCTTTAAAGAAAGAAATTAAAGAAGCCCTCAGAAAATGGAAAGATCTCCCATGCTCTTGGATAGGTAGGATCAATGTAGTAAAAATGGCAATCCTGCCAAAAGCAATCTACAGATTCAATGCTATCCCCATTAAAATCCCAGCACAATTCTTCACAGACCTTGAAAGAACAATAATCAATTTTGTATGGAAAAAC
AAAAGACCCAGGATAGCCAAAACAACCCTGTTCAACAAGGCAACGTCCGGAGGCATCACTATCCCTGACCTCAAGCTCTATTATAGAGCTATAGTCCTGAAAACAGCTTGGTATTGGCACAAAAATAGACAGGCAGACCAATGGAATCGAATTGAAAATCCGGGTATTAACCCACACACGTACGAACATCTGATCTTCGACAAAGAAGCTAAAAATATACAATGGA
ATAAAGAAAGCATCTTCAACAAATGGTGCTGGCATAATTGGAGACTGGCATGTAGAAAAATGCGGATCGATCCATGTTTATCGCCATGCACAAAACTTAAGTCCAAATGGATCAAAGACCTCAACATAAATCCAGCCACACTGAACCTCTTAGAAGAGAAAGTGGGTGGTACCCTTGAAAGAATTGGTACAGGAGACAGCTTCCTGAACATAACACCAGTAGCACA
GACAGTGAGATCAACAATTAATAAATGGGACCTACTGAAACTGAGAAGCTTCTGTAAGGCAAAGGACACAGTCAGCAAGACAAAAAGGCAGCCCACAGAATGGGAAAAAATATTCACTAATCCCACATCTGACAGAGGGTTGATCTCCAAAATTTACAAAGAACTCAAGAAGCAAGTCTCCAAAACAGCAAAGGACCCAATTAAGAAGTGGGCTGCAGAACTAAAT
AGAGAGTTTTCAATAGAGGAGTCTAAAATGGCTGAGAGGCACTTGAGAAAGTGCTCAACATCCTTAGCCATCCGGGAAATGCAAATCAAAGCAACTCTGAGATACCATCTCACTCCTGTCAGAATGGCTAAAATCAAAAACACCAATGACAATTTATGCTGGAGAGGATGTGGTGAAAGGGGGACACTCCTCCATTGCTGGTGGGAGTGCCAACTGGTTCAGCCAC
TTTGGAAAGCAGTATGGAGATTCCTCAGAAAAATGGGAATAAACCTACCACAAGACCCAGCAACTGCACTCTTAGGCATATATCCAAAGATGCCCATTCATATAACAAAGATGTCTGTTCCACTATGTTCATAGCAGCTTTATTTGTAATAGCCAGAACTTGGAAGCAACCTAGATGTCCCTCACCCGAAGAATGGATAGAGAAAATGTGGTACATCTACACAAT
GGAGTACTATTCAGCAGAAAAAAATAATGGAATCATGAGATTCGCAGGCAAATGGATGACACTAGAAGAAACCATCCTGAGTGAGGTAACCCAGTCACAAAAAGAGAAACATGGCATGTACTCTCTCATATATGGATTTTAGACATAGATCTGGGGAGTGCCAGCCTACAATCCTATTGCCAGAGAGTCTGGGAAACAGAAACTTCATTTACTGGCTGGTTAAGTA
CCGAGCTGAACTCAAGAAAGCCAGTATCAGAGAGGGAGGACTGATGATCAAAGGGATTAAGACTGGGCTGGTTGAACCCCCAGAAACAGCTGACCAGACAAAGGTTAACTCTTGAATCCCAGGCATCAGCCTGGGAATCCAGCATGGGACTGATCAAGACCCCCTGAATCTGGGTGTCAGTGAGGAGGCCTAGGTAATCTATTGAGCCTTGGACAGCAGATCAGTA
CCCATCCCAAGTATACACACGGTCTCTGGACTCCCATAGAACAATGTGAGGTGCACTCTCACCCTGAACTCTTGGGGAAGGGTCTAGGCCCTGCCCTATATGAAATGACAGACTATGGACACCCCCATGGAGGGCCTCACCCTCCCTGGGGGAATGAGGGGATAACGGGGGGGGGATGGGGGGAGGGCACGGGAGGAGGGGAGGGAAAGGGAGAGGGACCTCGTAC
TGACTTGTGAAGCAACCTTGTTTCTTATCTAAATTTAAAATGGAATAATAAAAAAATATTAATGAAAAAAAAAAA

>Lx5.4132629
AACGATTCCAGGAAAGGAGAGGAGGCTGGGAGTACAGGGAAGTAGCTGTCCCTCTGGCATCATCAGGTAAACAGGCCAGCTTCCTGGCCTGAGTTCCAGAGCCTGCAGATCGCCTGGGAACTGCACAGAGCTCCCAATCCCGGCGGGAGCATCAGGTAAACAGGCCAGCTTCCCGGCCTGTGTTCCAGAGCCTGCAGATCGCCTGGGAACTGCACAGAGCTCCCAA
TCCCGGCGGGAGCAAAGCACCCCAGACTCCAGATCTTCTCTCCCACCAACGATTCCAGGAAAGGAGAGGAGGCTGGGAGTACAGGGAAGTAGCTGTCCCTCTGGCATCATCAGGTAAACAGGCCAGCTTCCTGGCCTGAGTTCCAGAGCCTGCAGATCGCCTGGGAACTGCACAGAGCTCCCAATCCCGGCGGGAGCATCAGGTAAACAGGCCAGCTTCCCGGCCT
GTGTTCCAGAGCCTGCAGATCGCCTGGGAACTGCACAGAGCTCCCAATCCCGGCGGGAGCATCAGGTAAACAGGCCAGCTTCCCGGCCTGTGTTCCAGACCCTGCAGATCGCCTGGGAACTGCACAGAGCTCCCAATCCCGGCGGGAGCAAAGCACCCCAGACTCCAGATCTTCTCTCCCACCAACGATTCCAGGAAAGGAGAGGAGGTTGGGAGTACAGGGAAGT
AGCTGTCCCTCTGGCATCATCAGGTAAACAGGCCAGCTTCCCGGCCTGAGTTCCAGAGCCTGCAGATCGCCTGGGAACTGCACAGAGCTCCCAATCCCGGCGGGAGCATCAGGTAAACAGGCCAGCTTCCCGGCCTGTGTTCCAGAGCCTGCAGATCGCCTGGGAACTGCACAGAGCTCCCAATCCCGGCGGGAGCAAAGCACCCCAGACTCCAGATCTTCTCTCC
CACCAACGATTCCAGGAAAGGAGAGGAGGCTGGGAGTACAGGGAAGTAGCTGTCCCTCTGGCATCATCAGGTAAACAGGCCAGCTTCCTGGCCTGAGTTCCAGAGCCTGCAGATCGCCTGGGAACTGCACAGAGCTCCCAATCCCGGCGGGAGCATCAGGTAAACAGGCCAGCTTCCCGGCCTGTGTTCCAGAGCCTGCAGATCGCCTGGGAACTGCACAGAGCTC
CCAATCCCGGCGGGAGCATCAGGTAAACAGGCCAGCTTCCCGGCCTGTGTTCCAGAGCCTGCAGATCGCCTGGGAACTGCACAGAGCTCCCAATCCCGGCGGGAGCATCAGGTAAACAGGCCAGCTTCCCGGCCTGTGTTCCAGACCCTGCAGATCGCCTGGGAACTGCACAGAGCTCCCAATCCCGGCGGGAGCAAAGCACCCCAGACTCCAGATCTTCTCTCCC
ACCAACGATTCCAGGAAAGGAGAGGAGGCTGGGAGTACAGGGAAGTAGCTGTCCCTCTGGCATCATCAGGTAAACAGGCCAGCTTCCCGGCCTGAGTTCCAGAGCCTGCAGATCGCCTGGGAACTGCACAGAGCTCCCAATCCCGGCGAGAGCATCAGGTAAACAGGCCAGCTTCCCGGCCTGTGTTCCAGAGCCTGCAGATCACCTGGGAACTGCACAGAGCTCC
CAATCCCGGCGGGAGCATCAGGTAAACAGGCCAGCTTCCCGGCCTGTGTTCCAGAGCCTGCAGATCGCCTGGGAACTGCACAGAGCTCCCAATCCCGGCGGGAGCATCAGGTAAACAGGCCAGCTTCCTGGCCTGTGTTCCAGACCCTGCAGATCGCCTGGGAACTGCACAGAGCTCCCAATACCGGCGGGAGCAAAGCACCCCAGACTCCAGATCTTCTCTCCCA
CCAACGATTCCAGGAAAGGAGAGGAGGCTGGGAGTACAGGGAAGTAGCTGTCCCTCTGGCATCATCAGGTAAACAGGCCAGCTTCCCAGCCTGAGTTCCAGAGCCTGCAGATCGCCTGGGAACTGCACAGAGCTCCCAATCCCGGCGGGAGCCAAGCACCCCAGACTCCAGATCTTCTCTCCCACCAAAGAGACCAGGAAACTACAGGAACCAGGGAGACCAGGGG
AGTAGCTGTTCCGGATCAGGTACAACAGGACCCAGCATAGGGTCCAGGGGACCAGGTAGGAGGCTAAGCGCCCCAGATTCCTGTTCTTCTTCTCTACCCACGGACCAGCATTTGGACAAGGCATAGAAGAACCACCAGATTACTGGACTTACTAGCACCCACCAAAAGGGAACTCTGGTCCCCAGACCAGGAAGGGGCTCTTTCTGCACCCACTAAAAGAAGAGAT
GGGAAAGAGACAATACAAAAACACAGTCAGCAACAGAAAAACAAATATGACACCGCCAGATTCTAGAGACTCCACACCAGCAAGACCCCAATATTCAAACGCGAAGGAAGCAGAAGAGAACGATCTTAAAAGCACTTTCATGAAAATGATAGAGGCCCTAAAAGAGGATATTGGAAAATCCCTCAAAGAAATGGAAGAAAAAACAAACCAAAAAATGCAAGAAATG
GAAGAAAAAACAAACCAAAAAATGCAAGAATTGGAAGAAAAAACAAACCAAAAACGGCACGAAATGGAAGAAGAAATGAAGCAAAAAATGCAAGAAATGGAAGAAAAAGCAAAGCAAAAAATGCAAGAATTAGAAGAAGAAACATACCAAATAAGGCAGGAAATGGAAGAAGCAACAAAGCAAAAAATGCAAGAAATGGTAGAAAAAACAAAGCAAAAAATGAAAA
AAATGGAGGAAGAAACAAACCAAAAAAGGCAGGAAAGGGAAGAAGAAACAAAGCACAAAATCCAAGAAATGGAACAAAAAGCAAACCAAAAAAATGCAAGAAATGGAAGAAAGAACAAACCAAAAAAATTCATAAAAGGCAACAAACGGACGAACAGATAAACAGAAAATTGCAAGAAGCAATGCAAATGCTGCAAGGGTTGAAAACTGAAATAGAGACACTAAAAAA
AACACAATCTGAAGAAAGGCAGAAAATAGAAAAGCTGGACAAACAGTCAGGCACTACCAATACAAGCATTACCAACAGAATAGAAGAGATGGAAGAGAGAATCTCAAGTGTTGAAGATACATTAGGAGAAATGGATTTATCAACCAAAGAAAACCTTAAATCCAACAAATCCTTAACACAAAACATCCAGGAATTATGGGACACCATGAAAAGACCAAACCTAAGA
ATAATAGGTATAGAAGAAGGAGAAGAAATCCACCTCAAAGGGGCAGAAGGCATATTCAACAAAATCATAGAAGAAAACTTTCCCAACCTTAAGAAAGACATGCCAATGAAGGTACAAGAAGCCTACAGAACACCAAATAGAGTGGACCACAAAAAAAAATCCCCTCGCCACATAATAATCAAGACCCCAAACATACAGAATAAAGAAAGAATATTAAGAGCAGCTA
AGGAAAAAGGTCAAGTAACATATAAAGGCAGACCTATCAGAATTACACCTGATTTCTCCATGGAAACTCTGAAAGCCAGAAGGTCCTGGATAGATATTCTACCTACACTAAGAGACCATGGATGCCAGCCCAGACTACTATACCCGGCAAAGCTCTCAATCACTATAGACGGAGAAAACAAGATATTCCATGACAAAACTAGATTCAACCAATATGTGTCCACTAA
CCCAGCCCTACAGAAAATTTTGGAAGGAAAACTCCAACCCAAGGAAGCTAACTACAACCAGAAAAACATAAGCATTAGAGAATCCCTCTCTACCAACAGCCAAAAGAACAAAGGGGCGAAATTCCACCCATAATTCCACCAACAACAACCATCGCAAATCGAAGAAGGATGAGCAATCAATGGTCATTAATATCCCTCAATATTAATGGCCTTAACTCACCCATAA
AAAGACACAGACTAGCAGAATGGATTCGAAGACAGAACCCTTCCTTCTGCTGCATACAAGAAACACACCTCACCCTCAAAGACAGACATTACTTGAGAGTTAAAGGTTGGGAAAAAATTTTTCAATCAAATGGACCCAAGAAACAAGCAGGGGTAGCAATCCTAATATCTAACAAACTAGACTTTAAAATAAAATCAATCAAAAGAGATGAAGGAGGTCATTACAT
ACTCATCACAGGTGAAACCCACCAAGAGGAAGTGTCTATCCTGAACATCTATGCCCCCAATACAAAGGCATCCACATTCGTTAAAGAAACATTATTAGAACTTAAATCTCATCTAAAGCCCCACACAGTTATAGTGGGAGATTTCAACACACCACTCTCACCACTAGACAAGAATACCAGACAGGAACTCAACAAAGAAGCAAAGGGTCTAATAGAAGTAATGACC
CAACTGGGATTAACAGATATCTATAGAACATTTCATCCACATAAAAAAGAATATACCTTCTTCTCACCACCACATGGAACCTTCTCAAAAATCGACCACATACTTGGCAACATATCAAACCTCAGCAGCTACAAAAAAATTGGAATAACTCCATGCATCTTATCAGACCACCATGCCTTAAAATTAGAATTCAACAACAACACAAAGTGCAGAAAACCTACAAACT
CATGGAAATTGAATAATGCTCAACTGCACCATCCCTGGGTCAAAGAGGAAATAAAAAAAGAAATTAAAGACTTCCTCGAACTCAATGAGAATGAAGACACAACATACCCAAACCTATGGGACACTTTGAAAGCAGTGCTACGGGGGAAGTTCATAGCACTAAGTGCTCACATGAAGAAACAGGAAAACAGTTATACCAGAGAATTAACATCACAGCTAAAAGCTCT
AGAACACAAAGAAGCAAACTCCCCCAGGAGGAGTAGACGCCAAGAAATAATCAAATTGAGGGCTGAAATCAACAAAGTAGAAACCAGGAAAACAATACAAAGAATCAATGCAACGAAGAGTTGGTTCTTTGAGAAAATCAACAAGATAGACAAACCTTTGTCCAAACTTACCAAACAGCAGAGAGTGAACACGCAAATTAACAAAATCAGAAATGAAAAGGGGGAC
ATAACAACAGACTCTGAGGAAATCCAGAGAATCATTAGGTCATACTTTGAAAACCTGTACTCCACAAAATTCGAAAATTTAAAGGAAATGGATGACTTCCTGGACAGCTATCATTTGCCAAAACTAAATCAAGAACAGATAAGCAATTTAAATAGACCTATAACCCCTATGGAAATAGAAGCTGTCATCAAAAGTCTCCCAACCAAAAAAAGTCCAGGTCCAGATG
GCCTCAGTGCAGAATTCTACCACAAATTCAAAGAACAGCTTATACCAATTCTTCTCAAAGTGTTCCACACAATAGAAGCAGAAGGGTCTTTGCCAAACTCTTTCTATGAGGCTTCAATAACCTTGATCCCCAAGCCACACAAAGATACAACCAAGAAAGAGAACTTCAGACCAATATCCCTCATGAACATTGATGCAAAAATACTCAATAAAATACTGGCAAATCG
GATCCAAGAACACATCAAAACAATCATTCACCACGATCAAGTAGGTTTCATCCCAGGGATGCAGGGATGGTTCAACATACGAAAATCCATCAACGTAATCCACCATATAAACAAGCTGAAAAACAAAAATCACATGATCATCTCACTAGATGCTGAAAAAGCCTTTGACAAAATCCAACATCCCTTCATGATAAAGGTCTTGGAGAGATCTGGGATAACAGGGACA
TACTTAAACATAATAAAAGCCATATACAGTAAACCAACAGCCAACATCAAACTAAATGGACAGAAACTCAACGCAATTCCTCTAAAATCAGGAACAAAACAAGGCTGTCCACTCTCTCCATACCTTTTCAACATGGTACTTGAAGTTCTAGCTATAGCCATACGACAACAAAGGGGGATCAAGGGGATACAAATTGGGAAGGAAGAAGTCAAACTTTCACTGTTTG
CAGACGATATGATAGTTTATATCAGTGACCCAAAGAACTCTACCAGGGAACTCTTACAGCTGATTAACACCTTCAGCAAAGTGGCAGGTTACAAGATCAACTCAAGAAAGTCAGTAGCCCTACTATATACAGATGATAAAAGGGCTGAAAGAGAAATCAGAGAAACATCACCCTTTACAATTGCCACAAACAACATAAAATACCTTGGGGTAACGCTAACCAAACA
AGTGAAAGACCTGTATCGTAAGAACTTTGAATCTTTAAAGAAAGAAATTAAAGAAGCCCTCAGAAAATGGAAAGATCTCCCATGCTCATGGATAGGTAGGATCAATGTAGTAAAAATGGCAATCCTGCCAAAAGCAATCTACAGATTCAATGCTATCCCCATTAAAATCCCAGCACAGTTCTTCACAGACCTTGAAAGAACAATAATCAATTTTGTATGGAAAAAC
AAAAGACCCAGGATAGCCAAAACAACCCTGTTCAACAAGGCAACGTCCGGAGGCATCAATATCCCTGACCTCAAGCTCTATTATAGAGCTATAGTCCTGAAAACAGCTTGGTATTGGCACAAAAATAGACAGGCAGACCAATGGAATCGAATTGAAAATCCGGGTATTAACCCACACACGTACGAACATCTGATCTTCGACAAAGAAGCTAAAAATATACAATGGA
ATAAAGAAAGCATCTTCAACAAATGGTGCTGGCATAATTGGAGACTGGCATGTAGAAAAATGCGGATTGATCCATGTTTATCGCCATGCACAAAACTTAAGTCCAAATGGATCAAAGACCTCAACATAAATCCAGCCACACTGAACCTCTTAGAAGAGAAAGTGGGTGGTACCCTTGAAAGAATTGGTACAGGAGACAGCTTCCTGAACATAACACCAGTAGCACA
GACAGTGAGCTCAACAATTAATAAATGGGACCTACTGAAACTGAGAAGCTTCTGTAAGGCAAAGGACACAGTCAGCAAGACAAAAAGGCAGCCCACAGAATGGGAAAAAATATTCACTAATCCCACATCTGACAGAGGGTTGATCTCCAAAATTTACAAAGAACTCAAGAAGCAAGTCTCCAAAACAGCAAAGGACCCAATTAAGAAGTGGGCTGCAGAACTAAAT
AGAGAGTTTTCAATAGAGGAGTCTAAAATGGCTGAGAGGCACTTGAGAAAGTGCTCAACATCCTTAGCCATCCGGGAAATGCAAATCAAAGCAACTCTGAGATACCATCTCACTCCTGTCAGAATGGCTAAAATCAAAAACACCAATGACAATTTATGCTGGAGAGGATGTGGTGAAAGGGGGACACTCCTCCATTGCTGGTGGGAGTGCCAACTGGTTCAGCCAC
TTTGGAAAGCAGTATGGAGATTCCTCAGAAAAATGGGAATAAACCTACCACAAGACCCAGCAACTGCACTCTTAGGCATATATCCAAAGATGCCCATTCATATAACAAAGATGTCTGTTCCACTATGTTCATAGCAGCTTTATTTGTAATAGCCAGAACTTGGAAGCAACCTAGATGTCCCTCACCCGAAGAATGGATAGAGAAAATGTGGTACATCTACACAAT
GGAGTACTATTCAGCAGAAAAAAATAATGGAATCATGAGATTCGCAGGCAAATGGATGACACTAGAAGAAACCATCCTGAGTGAGGTAACCCAGTCACAAAAAGAGAAACATGGCATGTACTCTCTCATATATGGATTTTAGACATAGATCTGGGGAGTGCCAGCCTACAATCCTATTGCCAGAGAGTCTGGGAAACAGAAACTTCATTTACTGGCTGGTTAAGTA
CTGAGCTGAACTCAAGAAAGCCAGTATCAGAGAGGGAGGACTGATGATCAAAGGGATTAAGACTGGGCTGGTTGAACCCCCAGAAACAGCTGACCAGACAAAGGTTAACTCTTGAATCCCAGGCATCAGCCTGGGAATCCAGCATGGGACTGATCAAGACCCCCTGAATCTGGGTGTCAGTGAGGAGGCCTAGGTAATCTATTGAGCCTTGGACAGCAGATCAGTA
CCCATCCCAAGTATACACACGGTCTCTGGACTCCCATAGAACAATGTGAGGTGCACTCTCACCCTGAACTCTTGGGGAAGGGTCTAGGCCCTGCCCTATATGAAATGACAGACTATGGACACCCCCATGGAGGGCCTCACCCTCCCTGGGGGAATGAGGGGATAACGGGGGGGGGATGGGGGGAGGGCACGGGAGGAGGGGAGGGAAAGGGAGAGGGACCTCGTACT
GACTTGTGAAGCAACCTTGTTTCTTATCTAAATTTAAAATGGAAAATAAAAAAATATTAATAAAAAAAAAAAAAAAAAAAAAAAAAAAAAA

>Lx5.4266891
ATGGTCATGGTTGCGGTGTCTCTTTATAACAATAAAACCCTAAGACAGGATCTAATATGAAAGCAGAACTGAGACTTACTGGGGTGTGAGTGGGAATAGGGAGAAGACCACAGGGACCTTGGGAATGAATGAACTCAAGGTACATCATACACTTTAAGGAAAGTACTGGAAAATCTACCACTGTGATCCATGACTACACACCAATAAAACTATATTGAAATTTATA
ATGCTTTGAGAATAGCTACACTAATTTTTAAAGGTTAGCATGTAATGCATTGGCCTTCGGATGGTCTTTTCACACTTTGCTTTGGCTAAGGCTCGCACCTCCCTGCCCCCACCTCTTCCTTCTGGTCCACTTCCTCCCACATGCATTTTATTACCTTCCTTTCCATCCTTTAAGCTATCTTTTTTTTGCCTCTCATGATCTCATACTCCTTCCCGCAAAGCCCACA
TATATACAGATTAAAACCTAGGCTTAGTATGTAAGAAAGTTCAGTAGTCTTTCTGACACAGGCTGACTTCACGTTACATAATAATTTTCAGCTCCACACATTTCCCTGAAAATGTCCTGAATTTATTCTTCCTTATAGTGGAACTAATTCCCATTGTGGATATGTACCACATTTTTCCTTTGTATTTTTGAGACAGGGCTTTTCTATATATCCCTAGATGGCCTGT
AACTTGTCCTGTATAAGGATGCCCTCAAACTCACAGAGATCCAACTTCTGAGTGCTGGGACAAGGCATCTGCCTCCACACTTGGCTCCTCCTCATAATCCTCTCCTTCCTCTTCCTCCTCTTCTTCTTTCTCTTTTTGTTCTTCTTGTTCTTCTTCTAAAGCCATTCACCTAGAAGTAGATGTAGGCTAGAGTACCAATCAACAAGGATGAACAAAAGTCTCTCC
AGTAGGATCTAGAATACTTTGGGTAAAAGAAAAAACTCTGAGAATGCCTTTATCAAATCAGCTCACTGTTTGGGTGCCTTGTCTCTCAAATGTGCACATCCCTGCCTTTGAACTGTATTTTGAAGTCATGTCAATGATCTAAAACTCAAGTTTTTCTTTAAGAGAATTGAAAGAGCGGGTAAAAGGTGTAATAACACCTCTATACCAGAGAGGCTTTTCAGTGCAA
TGAAATGTTGCATGGGATGATATTTAAATGCTTACATGTTTCTTAATGGAATCATAACAATAATTAGTGATTTAATTTAATTCACTGTGATTCCTGCAAGAAGAATTTCTAGCACACTGTGAGCATTCACCTTACACACTCCTGTTTCATCCCCTGATAGTCCTCTTTCTAACTTCACTGTTTGATGGTGAGGGTGACAGCTTCATGCAGCCAGCTACCGATCAAT
GGCAGTATTAAAATAATGGTGTTGGAGGCTGGGAGTACAGGGAGGTGGCTGTCCCTCTGGCATCATCAGGTAAACAGGCCAGCTTCCCGGCCTGAGTTCCGGGGCCTGCAGATCGCCTGGGAACTGCACAGAGCTCCCAATCCCGGCGGGAGCATCAGGTAAACAGGCCAGCTTCCCGGCCTGTGTTCCAGAGCCTGCAGATCGCCTGGGAACTGCACAGAGCTCC
CAATCCGGGCGGGAGCGTCGGGTGAACAGGCCAGCTTCCTGGCCTGTGTTCCGGAGCCTGCAGATCGCCTGGGAGCTGCACAGGGCTCCCAATCCCGGCGGGAGCATCAGGTAAACAGGCCAGCTTCCTGGCCTGTGTCCCGGAGCCTGCAGATCGCCTGGGAACTGCACAGAGCTCCCAATACCGGCGGGAGCAAAGCACCCCAGACTCCAGGTCTTCTCTCCCA
CCAACGATTCCAGGAAAGGAGAGGAGGCTGGGAGTGCAGGGGGGTGGCTGTCCCTCTGGCATCATCAGGTAAACGGGCCAGCTTCCAGGCCTGAGTTCCAGAGCCTGCAGATCGCCTGGGGACTGCGCAGAGCTCCCAGTCCCGGCGGGAGCCAGGCACCCCAGACTCCAGATCTTCTCTCCCACCAAAGAGACCAGGAAACTACAGGAACCAGGGAGACCAGGGG
AGTAGCTGTTCCGGATCAGGTACAACAGGACCCAGCATAGGGTCCAGGGGACCAGGTAGGAGGCTAAGCGCCCCAGATTCCTGTTCTTCTTCTCTACCCACGGACCAGCATTTGGACAAGGCATAGAAGAACCACCAGATTACTGGACTTACTAGCACCCACCAAAAGGGAACTCTGGTCCCCAGACCAGGAAGGGGCTCTTTCTGCACCCACTAAAAGAAGAGAT
GGGAAAGAGACAATACAAAAACACAGTCAGCAACAGAAAAACAAATATGACACCGCCAGATTCTAGAGACTCCACACCAGCAAGACCCCAATATTCAAACGCGAAGGAAGCAGAAGAGAACGATCTTAAAAGCACTTTCATGAAAATGATAGAGGCCCTAAAAGAGGATATTGGAAAATCCCTCAAAGAAATGGAAGAAAAAACAAACCAAAAAATGCAAGAAATG
GAAGAAAAAACAAACCAAAAAATGCAAGAATTGGAAGAAAAAACAAACCAAAAACGGCACGAAATGGAAGAAGAAATGAAGCAAAAAATGCAAGAAATGGAAGAAAAAGCAAAGCAAAAAATGCAAGAATTAGAAGAAGAAACATACCAAATAAGGCAGGAAATGGAAGAAGCAACAAAGCAAAAAATGCAAGAAATGGTAGAAAAAACAAAGCAAAAAATGAAAA
AAATGGAGGAAGAAACAAACCAAAAAAGGCAGGAAAGGGAAGAAGAAACAAAGCACAAAATCCAAGAAATGGAACAAAAAGCAAACCAAAAAAATGCAAGAAATGGAAGAAAGAACAAACCAAAAAAATTCATAAAAGGCAACAAACAGACGAACAGATAAACAGAAAATTGCAAGAAGCAATGCAAATGCTGCAAGGGTTGAAAACTGAAATAGAGACACTAAAAAA
AACACAATCTGAAGAAAGGCAGAAAATAGAAAAGCTGGACAAACAGTCAGGCACTACCAATACAAGCATTACCAACAGAATAGAAGAGATGGAAGAGAGAATCTCAAGTGTTGAAGATACATTAGGAGAAATGGATTTATCAACCAAAGAAAACCTTAAATCCAACAAATCCTTAACACAAAACATCCAGGAATTATGGGACACCATGAAAAGACCAAACCTAAGA
ATAATAGGTATAGAAGAAGGAGAAGAAATCCACCTCAAAGGGGCAGAAGGCATATTCAACAAAATCATAGAAGAAAACTTTCCCAACCTTAAGAAAGACATGCCAATGAAGGTACAAGAAGCCTACAGAACACCAAATAGAGTGGACCACAAAAAAAAATCCCCTCGCCACATAATAATCAAGACCCCAAACATACAGAATAAAGAAAGAATATTAAGAGCAGCTA
AGGAAAAAGGTCAAGTAACATATAAAGGCAGACCTATCAGAATTACACCTGATTTCTCCATGGAAACTCTGAAAGCCAGAAGGTCCTGGATAGATATTCTACCTACACTAAGAGACCATGGATGCCAGCCCAGACTACTATACCCGGCAAAGCTCTCAATCACTATAGACGGAGAAAACAAGATATTCCATGACAAAACTAGATTCAACCAATATGTGTCCACTAA
CCCAGCCCTACAGAAAATTTTGGAAGGAAAACTCCAACCCAAGGAAGCTAACTACAACCAGAAAAACATAAGCATTAGAGAATCCCTCTCTACCAACAGCCAAAAGAACAAAGGGGCGAAATTCCACCCATAATTCCACCAACAACAACCATCGCAAATCGAAGAAGGATGAGCAATCAATGGTCATTAATATCCCTCAATATTAATGGCCTTAACTCACCCATAA
AAAGACACAGACTAGCAGAATGGATTCGAAGACAGAACCCTTCCTTCTGCTGCATACAAGAAACACACCTCACCCTCAAAGACAGACATTACTTGAGAGTTAAAGGTTGGGAAAAAATTTTTCAATCAAATGGACCCAAGAAACAAGCAGGGGTAGCAATCCTAATATCTAACAAACTAGACTTTAAAATAAAATCAATCAAAAGAGATGAAGGAGGTCATTACAT
ACTCATCACAGGTGAAACCCACCAAGACGAAGTGTCTATCCTGAACATCTATGCCCCCAATACAAAGGCATCCACATTCGTTAAAGAAACATTATTAGAACTTAAATCTCATCTAAAGCCCCACACAGTTATAGTGGGAGATTTCAACACACCACTCTCACCACTAGACAAGAATACCAGACAGGAACTCAACAAAGAAGCAAAGGGTCTAATAGAAGTAATGACC
CAACTGGGATTAACAGATATCTATAGAACATTTCATCCACATAAAAAAGAATATACCTTCTTCTCACCACCACATGGAACCTTCTCAAAAATCGACCACATACTTGGCAACATATCAAACCTCAGCAGCTACAAAAAAATTGGAATAACTCCATGCATCTTATCAGACCACCATGCCTTAAAATTAGAATTCAACAACAGCACAAATTGCAGAAAACCTACAAACT
CATGGAAATTGAATAATGCTCAACTGCACCATCCCTGGGTCAAAGAGGAAATAAAAAAAGAAATTAAAGACTTCCTCGAACTCAATGAGAATGAAGACACAACATACCCAAACCTATGGGACACTTTGAAAGCAGTGCTACGGGGGAAGTTCATAGCACTAAGTGCTCACATGAAGAAACAGGAAAACAGTTATACCAGAGAATTAACATCACAGCTAAAAGCTCT
AGAACACAAAGAAGCAAACTCCCCCAGGAGGAGTAGACGCCAGGAAATAATCAAATTGAGGGCTGAAATCAACAAAGTAGAAACCAGGAAAACAATACAAAGAATCAATGCAACGAAGAGTTGGTTCTTTGAGAAAATCAACAAGATAGACAAACCTTTGTCCAAACTTACCAAACAGCAGAGAATGAACACGCAAATTAACAAAATCAGAAATGAAAAGGGGGAC
ATAACAACAGACTCTGAGGAAATCCAGAGAATCATTAGGTCATACTTTGAAAACCTGTACTCCACAAAATTCGAAAATTTAAAGGAAATGGATGACTTCCTGGACAGCTATCATTTGCCAAAACTAAATCAAGAACAGATAAGCAATTTAAATAGACCTATAACCCCTATGGAAATAGAAGCTGTCATCAAAAGTCTCCCAACCAAAAAAAGTCCAGGTCCAGATG
GCCTCAGTGCAGAATTCTACCACAAATTCAAAGAACAGCTTATACCAATTCTTCTCAAAGTGTTCCACACAATAGAAGCAGAAGGGTCTTTGCCAAACTCTTTCTATGAGGCTTCAATAACCTTGATCCCCAAGCCACACAAAGATACAACCAAGAAAGAGAACTTCAGACCAATATCCCTCATGAACATTGATGCAAAAATACTCAATAAAATACTGGCAAATCG
GATCCAAGAACACATCAAAACAATCATTCACCACGATCAAGTAGGTTTCATCCCAGGGATGCAGGGATGGTTCAACATACGAAAATCCATCAACGTAATCCACCATATAAACAAGCTGAAAAACAAAAATCACATGATCATCTCACTAGATGCTGAAAAAGCCTTTGACAAAATCCAACATCCCTTCATGATAAAGGTCTTGGAGAGATCTGGGATAACAGGGACA
TACTTAAACATAATAAAAGCCATATACAGTAAACCAACAGCCAACATCAAACTAAATGGACAGAAACTCAACGCAATTCCTCTAAAATCAGGAACAAAACAAGGCTGTCCACTCTCTCCATACCTTTTCAACATGGTACTTGAAGTTCTAGCTATAGCCATACGACAACAAAGGGGGATCAAGGGGATACAAATTGGGAAGGAAGAAGTCAAACTTTCACTGTTTG
CAGACGATATGATAGTTTATATCAGTGACCCAAAGAACTCTACCAGGGAACTCTTACAGCTGATTAACACCTTCAGCAAAGTGGCAGGTTACAAGATCAACTCAAGAAAGTCAGTAGCCCTACTATATACAGATGATAAAAGGGCAGAAAGAGAAATCAGAGAAACATCACCCTTTACAATTGCCACAAACAACATAAAATACCTTGGGGTAACGCTAACCAAACA
AGTGAAAGACCTGTATCGTAAGAACTTTGAATCTTTAAAGAAAGAAATTAAAGAAGCCCTCAGAAAATGGAAAGATCTCCCATGCTCTTGGATAGGTAGGATCAATGTAGTAAAAATGGCAATCCTGCCAAAAGCAATCTACAGATTCAATGCTATCCCCATTAAAATCCCAGCACAATTCTTCACAGACCTTGAAAGAACAATAATCAATTTTGTATGGAAAAAC
AAAAGACCCAGGATAGCCAAAACAACCCTGTTCAACAAGGCAACGTCCGGAGGCATCACTATCCCTGACCTCAAGCTCTATTATAGAGCTATAGTCCTGAAAACAGCTTGGTATTGGCACAAAAATAGACAGGCAGACCAATGGAATCGAATTGAAAATCCGGGTATTAACCCACACACGTACGAACATCTGATCTTTGACAAAGAAGCTAAAAATATACAATGGA
ATAAAGAAAGCATCTTCAACAAATGGTGCTGGCATAATTGGAGACTGGCATGTAGAAAAATGCGGATCGATCCATGTTTATCGCCATGCACAAAACTTAAGTCCAAATGGATCAAAGACCTCAACATAAATCCAGCCACACTGAACCTCTTAGAAGAGAAAGTGGGTGGTACCCTTGAAAGAATTGGTACAGGAGACAGCTTCCTGAACATAACACCAGTAGCACA
GACAGTGAGATCAACAATTAATAAATGGGACCTACTGAAACTGAGAAGCTTCTGTAAGGCAAAGGACACAGTCAGCAAGACAAAAAGGCAGCCCACAGAATGGGAAAAAATATTCACTAATCCCACATCTGACAGAGGGTTGATCTCCAAAATTTACAAAGAACTCAAGAAGCAAGTCTCCAAAACAGCAAAGGACCCAATTAAGAAGTGGGCTGCAGAACTAAAT
AGAGAGTTTTCAATAGAGGAGTCTAAAATGGCTGAGAGGCACTTGAGAAAGTGCTCAACATCCTTAGCCATCCGGGAAATGCAAATCAAAGCAACTCTGAGATACCATCTCACTCCTGTCAGAATGGCTAAAATCAAAAACACCAATGACAATTTATGCTGGAGAGGATGTGGTGAAAGGGGGACACTCCTCCATTGCTGGTGGGAGTGCCAACTGGTTCAGCCAC
TTTGGAAAGCAGTATGGAGATTCCTCAGAAAAATGGGAATAAACCTACCACAAGACCCAGCAACTGCACTCTTAGGCATATATCCAAAGATGCCCATTCATATAACAAAGATGTCTGTTCCACTATGTTCATAGCAGCTTTATTTGTAATAGCCAGAACTTGGAAGCAACCTAGATGTCCCTCACCCGAAGAATGGATAGAGAAAATGTGGTACATCTACACAAT
GGAGTACTATTCAGCAGAAAAAAATAATGGAATCATGAGATTCGCAGGCAAATGGATGACACTAGAAGAAACCATCCTGAGTGAGGTAACCCAGTCACAAAAAGAGAAACATGGCATGTACTCTCTCATATATGGATTTTAGACATAGATCTGGGGAGTGCCAGCCTACAATCCTATTGCCAGAGAGTCTGGGAAACAGAAACTTCATTTACTGGCTGGTTAAGTA
CCGAGCTGAACTCAAGAAAGCCAGTATCAGAGAGGGAGGACTGATGATCAAAGGGATTAAGACTGGGCTGGTTGAACCCCCAGAAACAGCTGACCAGACAAAGGTTAACTCTTGAATCCCAGGCATCAGCCTGGGAATCCAGCATGGGACTGATCAAGACCCCCTGAATCTGGGTGTCAGTGAGGAGGCCTAGGTAATCTATTGAGCCTTGGACAGCAGATCAGTA
CCCATCCCAAGTATACACACGGTCTCTGGACTCCCATAGAACAATGTGAGGTGCACTCTCACCCTGAACTCTTGGGGAAGGGTCTAGGCCCTGCCCTATATGAAATGACAGACTATGGACACCCCCATGGAGGGCCTCACCCTCCCTGGGGGAATGAGGGGATAACGGGGGGGGGGATGGGGGGAGGGCACGGGAGGAGGGGAGGGAAAGGGAGAGGGACCTCGTA
CTGACTTGTGAAGCAACCTTGTTTCTTATCTAAATTTAAAATGGAATAATAAAAAAATATTAAAAAAAACAAAAAAAAAAAAAAAAAAAAAA

>Lx5.4346834
AATCACAATTTGGTCAACCTAAATTAACTGAATTCTGGATGTTTTAAGCTTTCCCTTTTCTTGTCTGTAAAACTCCATGCCCTTCTTAGGGACAGGTGGGCTATATAAATTAAAAGCATGCTGCATTCCTACAGCAATGTCTGGCAGCTTGTAAACTCGCCATCAGTGTTGGTACTGCAGTGTCTCCCTGCAGATGCCAGCTCAAGGTCAGAGAACTAGAAGTGGA
GATGTGTTCTGGCCAAACCCACAGTCTCCAGTAAGTTGTGCGGGCATTACATGTGGCTCTGTGGCCATTAGCCTCTTGAGCATATGCTAAGCCAGCCTGAACCGTGTCTGAGCTGCGTGGCCCCAAGCAAGCTACCTGACCTTACTGTGTGTCATTCTTCCTTTGCATGCTGGGTAATTTCTTCCCTGTGTGGGTACTAATGGTGTGAGAGAGAGTGTGTGTGCAA
AGCGCTCCACTCAGAAGTCAGTAGATGGAGCCTGCTCAGCCTCTGTCACTCCACCACTCCACTGCTACCTGGCCGGGTGTGACCTTGAACAATTGTTGGGAAGGTATCCAGTGTCCTGTTGTGCATTACCAACATACGGCTTCAAGAGGCGGATTGGCACATGTAACTGCCTCAGACTGAAGAAACTGCCTCCTACCTTCCCTGAGAGTCTAGAGTTAGCCACCAC
CACCCATCCCCACATGCCCCAACACAAAAGATGAAAATGAAGGGTAGACTTCCCAGACTGGAAACCCATTTCTCACTTTAAGACCCCAGAGTGGTTCACCAATCATGGTCTCTGCACACTGGCATCTTGGCTGCCTGCTCAGGAAACCCCACTCCTTGAGAGGTTGTGTGTGTGTGTGTGTGTGTGTGTGTGTGTGTGTGTGTGTGCGCCTGTTTTCTGCAGGGTC
ACAGCACACACACACACACACACACACATCTTCCTTCCTTCCTTAGCCCCTCCCCCTTTGTAAGTTATAGTTTCCTCACCACTCAGGAAGACTAAATCAGGTGTGTTTGGAGAATTATGCAGTTGTCACTGCTACAGCTAGCAGCATAAAGTGGCTTAGCATTTTCATGAATATATACTCAACATAGAATAAAAGTGAAAGGAGGAGGCTGGGAGTACAGGGAAGTA
GCTGTCCCTCTGGCATCATCAGGTAAACAGGCCAGCTTCCCGGCCTGAGTTCCAGAGCCTGCAGATCGCCTGGGAACTGCACAGAGCTCCCAATCCCGGCGGGAGCATCAGGTAAACAGGCCAGCTTCCCGGCCTGTGTTCCAGAGCCTGCAGATCTCCTGAGAACTGCACAGAGCTCCCAATCCCGGCGGGAGCAAAGCACCCCAGACTCCAGATCTTCTCTCCC
ACCAACGATTCCAGGAAAGGAGAGGAGGCTGGGAGTACAGGGAAGTAGCTGTCCCTCTGGCATCATCAGGTAAACAGGCCAGCTTCCCGGCCTGAGTTCCAGAGCCTGCAGATCGCCTGGGAACTGCACAGAGCTCCCAATCCCGGCGGGAGCATCAGGTAAACAGGCCAGCTTCCCGGCCTGTGTTCCAGAGCCTGCAGATCACCTGGGAACTGCACAGAGCTCC
CAATCCCGGCGGGAGCATCAGGTAAACAGGCCAGCTTCCCGGCCTGTGTTCCAGAGCCTGCAGATCGCCTGGGAACTGCACAGAGCTCCCAATCCCGGAGGGAGCATCAGGTAAACAGGCCAGCTTCCCGGCCTGTGTTCCAGAGCCTGCAGATCGCCTGGGAACTGCACAGAGCTCCCAATACCGGCGGGAGCAAAGCACCCCAGACTCCAGATCTTCTCTCCCA
CCAACGATTCCAGGAAAGGAGAGGAGGCTGGGAGTACAGGGAAGTAGCTGTCCCTCTGGCATCATCAGGTAAACAGGCCAGCTTCCCAGCCTGAGTTCCAGAGCCTGCAGATCGCCTGGGAACTGCACAGAGCTCCCAATCCCGGCGGGAGCCAAGCACCCCAGACTCCAGATCTTCTCTCCCACCAAAGAGACCAGGAAACTACAGGAACCAGGGAGACCAGGGG
AGTAGCTGTTCCGGATCAGGTACAACAGGACCCAGCATAGGGTCCAGGGGACCAGGTAGGAGGCTAAGCGCCCCAGATTCCTGTTCTTCTTCTCTACCCACGGACCAGCATTTGGACAAGGCATAGAAGAACCACCAGATTACTGGACTTACTAGCACCCACCAAAAGGGAACTCTGGTCCCCAGACCAGGAAGGGGCTCTTTCTGCACCCACTAAAAGAAGAGAT
GGGAAAGAGACAATACAAAAACACAGTCAGCAACAGAAAAACAAATATGACACCGCCAGATTCTAGAGACTCCACACCAGCAAGACCCCAATATTCAAACGCGAAGGAAGCAGAAGAGAACGATCTTAAAAGCACTTTCATGAAAATGATAGAGGCCCTAAAAGAGGATATTGGAAAATCCCTCAAACAAATGGAAGAAAAAACAAACCAAAAAATGCAAGAAATG
GAAGAAAAAACAAACCAAAAAATGCAAGAATTGGAAGAAAAAACAAACCAAAAACGGCACGAAATGGAAGAAGAAATGAAGCAAAAAATGCAAGAAATGGAAGAAAAAGCAAAGCAAAAAATGCAAGAATTAGAAGAAGAAACATACCAAATAAGGCAGGAAATGGAAGAAGCAACAAAGCAAAAAATGCAAGAAATGGTAGAAAAAACAAAGCAAAAAATAAAAA
AAATGGAGGAAGAAACAAACCAAAAAAGGCAGGAAAGGGAAGAAGAAACAAAGCACAAAATCCAAGAAATGGAACAAAAAGCAAACCAAAAAAATGCAAGAAATGGAAGAAAGAACAAACCAAAAAAATTCATAAAAGGCAACAAACGGACGAACAGATAAACAGAAAATTGCAAGAAGCAATGCAAATGCTGCAAGGGTTGAAAACTGAAATAGAGACACTAAAAAA
AACACAATCTGAAGAAAGGCAGAAAATAGAAAAGCTGGACAAACAGTCAGGCACTACCAATACAAGCATTACCAACAGAATAGAAGAGATGGAAGAGAGAATCTCAAGTGTTGAAGATACATTAGGAGAAATGGATTTATCAACCAAAGAAAACCTTAAATCCAACAAATCCTTAACACAAAACATCCAGGAATTATGGGACACCATGAAAAGACCAAACATAAGA
ATAATAGGTATAGAAGAAGGAGAAGAAATCCACCTCAAAGGGGCAGAAGGCATATTCAACAAAATCATAGAAGAAAACTTTCCCAACCTTAAGAAAGACATGCCAATGAAGGTACAAGAAGCCTACAGAACACCAAATAGAGTGGACCACAAAAAAAAATCCCCTCGCCACATAATAATCAAGACCCCAAACATACAGAATAAAGAAAGAATATTAAGAGCAGCTA
AGGAAAAAGGTCAAGTAACATATAAAGGCAGACCTATCAGAATTACACCTGATTTCTCCATGGAAACTCTGAAAGCCAGAAGGTCCTGGATAGATATTCTACCTACACTAAGAGACCATGGATGCCAGCCCAGACTACTATACCCGGCAAAGCTCTCAATCACTATAGACGGAGAAAACAAGATATTCCATGACAAAACTAGATTCAACCAATATGTGTCCACTAA
CCCAGCCCTACAGAAAATTTTGGAAGGAAAACTCCAACCCAAGGAAGCTAACTACAACCAGAAAAACATAAGCATTAGAGAATCCCTCTCTACCAACAGCCAAAAGAACAAAGGGGCGAAATTCCACCCATAATTCCACCAACAACAACCATCGCAAATCGAAGAAGGATGAGCAATCAATGGTCATTAATATCCCTCAATATTAATGGCCTTAACTCACCCATAA
AAAGACACAGACTAGCAGAATGGATTCGAAGACAGAACCCTTCCTTCTGCTGCATACAAGAAACACACCTCACCCTCAAAGACAGACATTACTTGAGAGTTAAAGGTTGGGAAAAAATTTTTCAATCAAATGGACCCAAGAAACAAGCAGGGGTAGCAATCCTAATATCTAACAAACTAGACTTTAAAATAAAATCAATCAAAAGAGATGAAGGAGGTCATTACAT
ACTCATCACAGGTGAAACCCACCAAGACGAAGTGTCTATCCTGAACATCTATGCCCCCAATACAAAGGCATCCACATTCGTTAAAGAAACATTATTAGAACTTAAATCTCATCTAAAGCCCCACACAGTTATAGTGGGAGATTTCAACACACCACTCTCACCACTAGACAAGAATACCAGACAGGAACTCAACAAAGAAGCAAAGGGTCTAATAGAAGTAATGACC
CAACTGGGATTAACAGATATCTATAGAACATTTCATCCACATAAAAATGAATATACCTTCTTCTCACCACCACATGGAACCTTCTCAAAAATCGACCACATACTTGGCAACATATCAAACCTCAGCAGCTACAAAAAAATTGGAATAACTCCATGCATCTTATCAGACCACCATGCCTTAAAATTAGAATTCAACAACAACACAAAGTGCAGAAAACCTACAAACT
CATGGAAATTGAATAATGCTCAACTGCACCATCCCTGGGTCAAAGAGGAAATAAAAAAAGAAATTAAAGACTTCCTCGAACTCAATGAGAATGAAGACACAACATACCCAAACCTATGGGACACTTTGAAAGCAGTGCTACGGGGGAAGTTCATAGCACTAAGTGCTCACATGAAGAAACAGGAAAACAGTTATACCAGAGAATTAACATCACAGCTAAAAGCTCT
AGAACACAAAGAAGCAAACTCCCCCAGGAGGAGTAGACGCCAGGAAATAATCAAATTGAGGGCTGAAATCAACAAAGTAGAAACCAGGAAAACAATACAAAGAATCAATGCAACGAAGAGTTGGTTCTTTGAGAAAATCAACAAGATAGACAAACCTTTGTCCAAACTTACCAAACAGCAGAGAATGAACACGCAAATTAACAAAATCAGAAATGAAAAGGGGGAC
ATAACAACAGACTCTGAGGAAATCCAGAGAATCATTAGGTCATACTTTGAAAACCTGTACTCCACAAAATTTGAAAATTTAAAGGAAATGGATGACTTCCTGGACAGCTATCATTTGCCAAAACTAAATCAAGAACAGATAAGCAATTTAAATAGACCTATAACCCCTATGGAAATAGAAGCTGTCATCAAAAGTCTCCCAACCAAAAAAAGTCCAGGTCCAGATG
GCCTCAGTGCAGAATTCTACCAGAAATTCAAAGAACAGCTTATACCAATTCTTCTCAAAGTGTTCCACACAATAGAAGCAGAAGGGTCTTTGCCAAACTCTTTCTATGAGGCTTCAATAACCTTGATCCCCAAGCCACACAAAGATACAACCAAGAAAGAGAACTTCAGACCAATATCCCTCATGAACATTGATGCAAAAATACTCAATAAAATACTGGCAAATCG
GATCCAAGAACACATCAAAACAATCATTCACCACGATCAAGTAGGTTTCATCCCAGGGATGCAGGGATGGTTCAACATACGAAAATCCATCAACGTAATCCACCATATAAACAAGCTGAAAAACAAAAATCACATGATCATCTCACTAGATGCTGAAAAAGCCTTTGACAAAATCCAACATCCCTTCATGATAAAGGTCTTGGAGAGATCTGGGATAACAGGGACA
TACTTAAACATAATAAAAGCCATATACAGTAAACCAACAGCCAACATCAAACTAAATGGACAGAAACTCAACGCAATTCCTCTAAAATCAGGAACAAAACAAGGCTGTCCACTCTCTCCATACCTTTTCAACATGGTACTTGAAGTTCTAGCTATAGCCATACGACAACAAAGGGGGATCAAGGGGATACAAATTGGGAAGGAAGAAGTCAAACTTTCACTGTTTG
CAGACGATATGATAGTTTATATCAGTGACCCAAAGAACTCTACCAGGGAACTCTTACAGCTGATTAACACCTTCAGCAAAGTGGCAGGTTACAAGATCAACTCAAGAAAGTCAGTAGCCCTACTATATACAGATGATAAAAGGGCTGAAAGAGAAATCAGAGAAACATCACCCTTTACAATTGCCACAAACAACATAAAATACCTTGGGGTAACGCTAACCAAACA
AGTGAAAGACCTGTATCGTAAGAACTTTGAATCTTTAAAGAAAGAAATTAAAGAAGCCCTCAGAAAATGGAAAGATCTCCCATGCTCTTGGATAGGTAGGATCAATGTAGTAAAAATGGCAATCCTGCCAAAAGCAATCTACAGATTCAATGCTATCCCCATTAAAATCCCAGCACAATTCTTCACAGACCTTGAAAGAACAATAATCAATTTTGTATGGAAAAAC
AAAAGACCCAGGATAGCCAAAACAACCCTGTTCAACAAGGCAACGTCCGGAGGCATCACTATCCCTGACCTCAAGCTCTATTATAGAGCTATAGTCCTGAAAACAGCTTGGTATTGGCACAAAAATAGACAGGCAGACCAATGGAATCGAATTGAAAATCCGGGTATTAACCCACACACGTACGAACATCTGATCTTCGACAAAGAAGCTAAAAATATACAATGGA
ATAAAGAAAGCATCTTCAACAAATGGTGCTGGCATAATTGGAGACTGGCATGTAGAAAAATGCGGATCGATCCATGTTTATCGCCATGCACAAAACTTAAGTCCAAATGGATCAAAGACCTCAACATAAATCCAGCCACACTGAACCTCTTAGAAGAGAAAGTGGGTGGTACCCTTGAAAGAATTGGTACAGGAGACAGCTTCCTGAACATAACACCAGTAGCACA
GACAGTGAGATCAACAATTAATAAATGGGACCTACTGAAACTGAGAAGCTTCTGTAAGGCAAAGGACACAGTCAGCAAGACAAAAAGGCAGCCCACAGAATGGGAAAAAATATTCACTAATCCCACATCTGACAGAGGGTTGATCTCCAAAATTTACAAAGAACTCAAGAAGCAAGTCTCCAAAACAGCAAAGGACCCAATTAAGAAGTGGGCTGCAGAACTAAAT
AGAGAGTTTTCAATAGAGGAGTCTAAAATGGCTGAGAGGCACTTGAGAAAGTGCTCAACATCCTTAGCCATCCGGGAAATGCAAATCAAAGCAACTCTGAGATACCATCTCACTCCTGTCAGAATGGCTAAAATCAAAAACACCAATGACAATTTATGCTGGAGAGGATGTGGTGAAAGGGGGACACTCCTCCATTGCTGGTGGGAGTGCCAACTGGTTCAGCCAC
TTTGGAAAGCAGTATGGAGATTCCTCAGAAAAATGGGAATAAACCTACCACAAGACCCAGCAACTGCACTCTTAGGCATATATCCAAAGATGCCCATTCATATAACAAAGATGTCTGTTCCACTATGTTCATAGCAGCTTTATTTGTAATAGCCAGAACTTGGAAGCAACCTAGATGTCCCTCACCCGAAGAATGGATAGAGAAAATGTGGTACATCTACACAAT
GGAGTACTATTCAGCAGAAAAAAATAATGGAATCATGAGATTCGCAGGCAAATGGATGACACTAGAAGAAACCATCCTGAGTGAGGTAACCCAGTCACAAAAAGAGAAACATGGCATGTACTCTCTCATATATGGATTTTAGACATAGATCTGGGGAGTGCCAGCCTACAATCCTATTGCCAGAGAGTCTGGGAAACAGAAACTTCATTTACTGGCTGGTTAAGTA
CTGAGCTGAACTCAAGAAAGCCAGTATCAGAGAGGGAGGACTGATGATCAAAGGGATTAAGACTGGGCTGGTTGAACCCCCAGAAACAGCTGACCAGACAAAGGTTAACTCTTGAATCCCAGGCATCAGCCTGGGAATCCAGCATGGGACTGATCAAGACCCCCTGAATCTGGGTGTCAGTGAGGAGGCCTAGGTAATCTATTGAGCCTTGGACAGCAGATCAGTA
CCCATCCCAAGTATACACACGGTCTCTGGACTCCCATAGAACAATGTGAGGTGCACTCTCACCCTGAACTCTTGGGGAAGGGTCTAGGCCCTGCCCTATATGAAATGACAGACTATGGACACCCCCATGGAGGGCCTCACCCTCCCTGGGGGAATGAGGGGATAACGGGGGGGGGATGGGGGGAGGGCACGGGAGGAGGGGAGGGAAAGGGAGAGGGACCTCGTACT
GACTTGTGAAGCAACCTTGTTTCTTATCTAAATTTAAAATGGAAAATAAAAAAATATTATATAAAAAAAAATAAATAATAAAAA
