# Supplementary material for: Formation of spermatogonia and fertile oocytes in golden hamsters requires piRNAs
Source: Nat Cell Biol. 2021 Sep 6;23(9):992–1001. doi: 10.1038/s41556-021-00746-2 (PMC8437802; doi:10.1038/s41556-021-00746-2)
Supplement: Supplementary file 2 — Reporting Summary [file 41556_2021_746_MOESM2_ESM.pdf]

nature research

Corresponding author(s): Petr Svoboda

Last updated by author(s): Jul 25, 2021

Reporting Summary

Nature Research wishes to improve the reproducibility of the work that we publish. This form provides structure for consistency and transparency in reporting. For further information on Nature Research policies, see our Editorial Policies and the Editorial Policy Checklist.

Statistics

For all statistical analyses, confirm that the following items are present in the figure legend, table legend, main text, or Methods section.

n/a	Confirmed	
☐	☒	The exact sample size (*n*) for each experimental group/condition, given as a discrete number and unit of measurement
☒	☐	A statement on whether measurements were taken from distinct samples or whether the same sample was measured repeatedly
☐	☒	The statistical test(s) used AND whether they are one- or two-sided<br>*Only common tests should be described solely by name; describe more complex techniques in the Methods section.*
☒	☐	A description of all covariates tested
☒	☐	A description of any assumptions or corrections, such as tests of normality and adjustment for multiple comparisons
☐	☒	A full description of the statistical parameters including central tendency (e.g. means) or other basic estimates (e.g. regression coefficient) AND variation (e.g. standard deviation) or associated estimates of uncertainty (e.g. confidence intervals)
☐	☒	For null hypothesis testing, the test statistic (e.g. *F*, *t*, *r*) with confidence intervals, effect sizes, degrees of freedom and *P* value noted<br>*Give P values as exact values whenever suitable.*
☒	☐	For Bayesian analysis, information on the choice of priors and Markov chain Monte Carlo settings
☒	☐	For hierarchical and complex designs, identification of the appropriate level for tests and full reporting of outcomes
☒	☐	Estimates of effect sizes (e.g. Cohen's *d*, Pearson's *r*), indicating how they were calculated

Our web collection on statistics for biologists contains articles on many of the points above.

Software and code

Policy information about availability of computer code

Data collection

Microscopy images were obtained using Leica DM6000 and SP8 firmware.

Data analysis

Microscopy images were visualized and formatted in LAS AF LITE 3.3 (Leica), and quantitatively analyzed using the Imaris 9.6 software (Bitplane AG).

Detailed description of software used for sequence data analyses, including original references, is provided in the subsection Bioinformatic analyses in the Methods section. Briefly, the following existing packages, algorithms, and scripts were used: STAR 2.7.3a aligner, featureCounts v2.0.0, DESeq2 package, UCSC tools, bbduk.sh 38.87, Cutadapt 2.10, UMI tools 1.1.1., clusterProfiler, RepeatMasker 4.0.9, Bismark (deduplicate_bismark, bismark_methylation_extractor), and samtools 1.10.

The code used for bioinformatic data analysis is available here: https://github.com/fhorvat/bioinfo_repo/tree/master/papers/piRNA_2021.

For manuscripts utilizing custom algorithms or software that are central to the research but not yet described in published literature, software must be made available to editors and reviewers. We strongly encourage code deposition in a community repository (e.g. GitHub). See the Nature Research guidelines for submitting code & software for further information.

Data

Policy information about availability of data

All manuscripts must include a data availability statement. This statement should provide the following information, where applicable:

- Accession codes, unique identifiers, or web links for publicly available datasets
- A list of figures that have associated raw data
- A description of any restrictions on data availability

All data are available in the main text or the supplementary materials. High-throughput sequencing data access to original results in the manuscript: GSE164658. Previously published data that were re-analyzed: GSE5241556, PRJNA47156457, GSE7237958, GSE7489659, GSE11677160, GSE4941761, GSE13756362, and GSE5396063.

Field-specific reporting

Please select the one below that is the best fit for your research. If you are not sure, read the appropriate sections before making your selection.

☒ Life sciences ☐ Behavioural & social sciences ☐ Ecological, evolutionary & environmental sciences

For a reference copy of the document with all sections, see nature.com/documents/nr-reporting-summary-flat.pdf

Life sciences study design

All studies must disclose on these points even when the disclosure is negative.

Sample size	No sample size was calculated, the number of replicates was determined by availability of the limited material. All results were replicated at least twice or done in duplicates, except of the bisulfite sequencing of the genome, which was done once on a pool of oocytes because of the limited amount of material. Numbers of replicates in trancriptome analyses are apparent from the Supplementary Table 10.
Data exclusions	We excluded two RNA sequencing libraries produced from hamster oocyte transcriptome analysis. These two libraries were outliers in PCA and had poor quality/low complexity because of the limited amount of the starting material. In addition, we excluded from the preimplantation development analysis those matings where fertilization did not occur (evidenced by the absence of pronucleus formation and lack of paternal DNA presence in unfertilized eggs).
Replication	Key experiments were independently replicated using at least two biological replicates. Numbers of replicates are indicated in methods, figure legends or description of sequencing libraries. All attempts at replication were successful. Furthermore, post-zygotic sterile phenotype of Mov10l1 knock-out was independently replicated in a co-submitted manuscript from Haruhiko Siomi's group (Hasuwa et al. Production of functional oocytes requires maternally expressed PIWI genes and piRNAs in golden hamsters, 2021; https://doi.org/10.1101/2021.01.27.428354) and in a preprint from Jianmin Li's lab (Zhang H. et al., piRNA pathway is essential for generating functional oocytes in golden hamster; https://doi.org/10.1101/2021.03.21.434510).
Randomization	Planned sampling randomization was not implemented in this study because the project relied on genetically modified hamsters where the specific aspects of their breeding and supply of animals made limited sample availability. Accordingly, animals with required genotypes were used for experiments as they were produced, i.e. samples were not strictly randomized but were not deliberately chosen either.
Blinding	Blinding was not implemented. First, mutant phenotypes were expected to be of qualitative nature and their analysis should not be subconsciously biased. Particularly blinding sterile phenotype analyses where mutants have atrophic testes and obvious histological defects would not bring any benefit. Second, limited human resources did not allow separating management of the animal colony, mating & genotyping, and sample preparation from data acquisition and analysis.

Reporting for specific materials, systems and methods

We require information from authors about some types of materials, experimental systems and methods used in many studies. Here, indicate whether each material, system or method listed is relevant to your study. If you are not sure if a list item applies to your research, read the appropriate section before selecting a response.

Materials & experimental systems

n/a	Involved in the study	
☐	☒	Antibodies
☒	☐	Eukaryotic cell lines
☒	☐	Palaeontology and archaeology
☐	☒	Animals and other organisms
☒	☐	Human research participants
☒	☐	Clinical data
☒	☐	Dual use research of concern

Methods

n/a	Involved in the study	
☒	☐	ChIP-seq
☒	☐	Flow cytometry
☒	☐	MRI-based neuroimaging

Antibodies

Antibodies used	Antibody use including antibody origin (western blotting and immunofluorescence) is described in detail in methods. Here is just the list of used antibodies:<br><br>primary antibodies:<br>anti-DDX4 (Abcam, #ab27591 and #ab13840)<br>anti-ɣH2AX (Milipore, #05-636)<br>anti-H3K9me3 (Upstate (Merck-Millipore) #07-442)<br>anti-IAP GAG (non-commercial, a gift from B.R. Cullen, Duke University)<br>anti-LINE1 ORF1p (non-commercial, a gift from Dónal O´Carroll, University of Edinburgh)<br>anti-MOV10L1 (non-commercial, a gift from P. Jeremy Wang, University of Pennsylvania)<br>anti-SCP3 (Abcam, #ab976672)<br>anti-Tubulin (Sigma, #T6074)<br>anti-WT1 (Novus Biologicals, #NB110-60011)<br>anti-ZBTB16 (Atlas antibodies, #HPA001499)<br><br>secondary antibodies:<br>anti-Rabbit-HRP (Thermo Fisher Scientific, # G21234)<br>anti-mouse-HRP (Thermo Fisher Scientific, # G21040)<br>anti-mouse conjugated with Alexa 488 (Thermo Fisher, # A21202)<br>anti-mouse conjugated with Alexa 594 (Thermo Fisher, # A-21203)<br>anti-rabbit conjugated with Alexa 488 (Thermo Fisher, # A-21206)<br>anti-rabbit conjugated with Alexa 594 (Thermo Fisher, # A21207)
Validation	mouse anti-DDX4 (Abcam, cat# ab27591)<br>validation reference: https://www.abcam.com/ddx4--mvh-antibody-mabcam27591-ab27591.html<br><br>rabbit anti-DDX4 (Abcam, cat# ab13840)<br>validation reference: https://www.abcam.com/ddx4--mvh-antibody-ab13840.html<br><br>mouse anti-ɣH2AX (Milipore, cat# 05-636)<br>validation reference: https://www.merckmillipore.com/CZ/cs/product/Anti-phospho-Histone-H2A.X-Ser139-Antibody-clone-JBW301,MM_NF-05-636?ReferrerURL=https%3A%2F%2Fwww.google.com%2F<br><br>rabbit anti-H3K9-me3 (cat# 07-442)<br>validation reference: https://www.merckmillipore.com/CZ/cs/product/Anti-trimethyl-Histone-H3-Lys9-Antibody,MM_NF-07-442?ReferrerURL=https%3A%2F%2Fwww.google.com%2F&bd=1<br><br>rabbit anti-IAP GAG (gift from B.R. Cullen, Duke University Medical Center)<br>validation reference: doi: 10.1038/s41594-018-0058-0<br><br>rabbit anti-LINE1 ORF1p (gift from Dónal O´Carroll, University of Edinburgh)<br>validation reference: DOI:https://doi.org/10.1016/j.molcel.2013.04.026<br><br>rabbit anti-MOV10L1 (gift from P. Jeremy Wang, University of Pennsylvania)<br>validation reference: https://doi.org/10.1073/pnas.1003953107<br><br>mouse anti-SYCP3 (Abcam, cat# ab97672)<br>validation reference: https://www.abcam.com/scp3-antibody-cor-10g117-ab97672.html<br><br>mouse anti-Tubulin (Sigma cat# T6074)<br>validation reference: https://www.sigmaaldrich.com/catalog/product/sigma/t6074?lang=en&region=CZ<br><br>mouse anti-Tubulin (Abcam, cat# ab7750)<br>validation reference: https://www.abcam.com/alpha-tubulin-antibody-tu-01-ab7750.html<br><br>mouse anti-WT1 (Novus Biologicals, cat# NB110-60011)<br>validation reference: https://www.novusbio.com/products/wt1-antibody-6f-h2_nb110-60011<br><br>rabbit anti-ZBTB16 (Atlas antibodies, cat# HPA001499)<br>validation reference: https://www.atlasantibodies.com/products/antibodies/primary-antibodies/triple-a-polyclonals/zbtb16-antibody-hpa001499/

Animals and other organisms

Policy information about studies involving animals; ARRIVE guidelines recommended for reporting animal research

Laboratory animals	Golden (Syrian) hamsters Mesocricetus auratus, males 0 to 102 weeks and females 10 to 52 weeks old.
Wild animals	The study did not involve wild animals

Field-collected samples	The study did not involve samples collected in the field
Ethics oversight	Animal experiments were approved by the Animal Experimentation Committee at the RIKEN Tsukuba Institute (T2019-J004) and the Institutional Animal Use and Care Committee at the Institute of Molecular Genetics of the Czech Academy of Sciences (approvals no. 42/2016 and 70/2018).

Note that full information on the approval of the study protocol must also be provided in the manuscript.
