# Supplementary figures and images for: Formation of spermatogonia and fertile oocytes in golden hamsters requires piRNAs
Source: Nat Cell Biol. 2021 Sep 6;23(9):992–1001. doi: 10.1038/s41556-021-00746-2 (PMC8437802; doi:10.1038/s41556-021-00746-2)

Figure 1g

Figure 1g

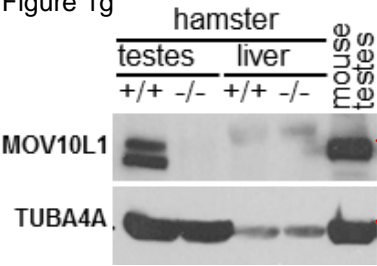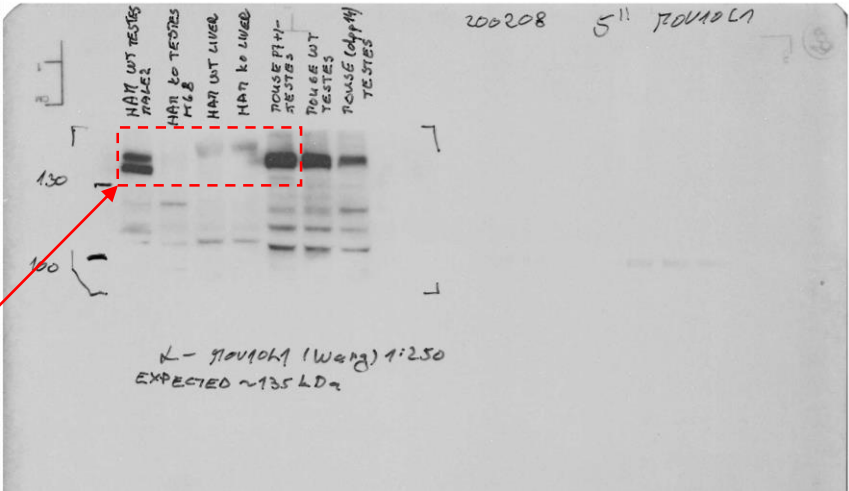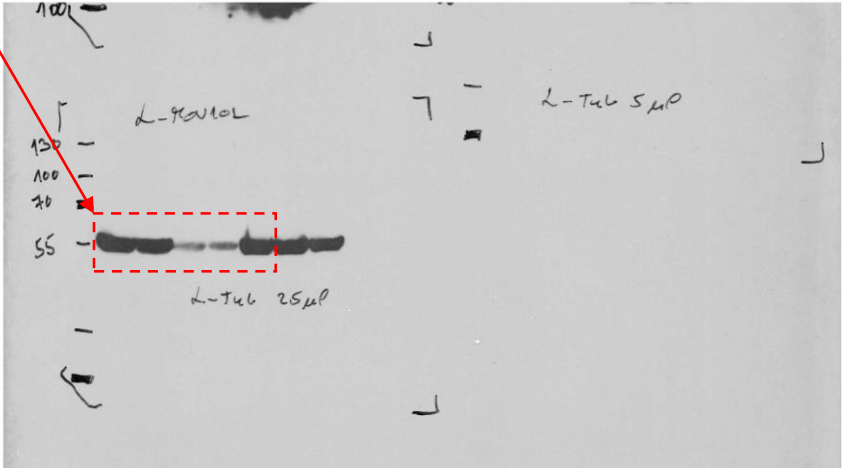

Supplement: Source Data Fig. 1 — Unprocessed western blots. [file 41556_2021_746_MOESM6_ESM.pdf]

Extended Figure 3a

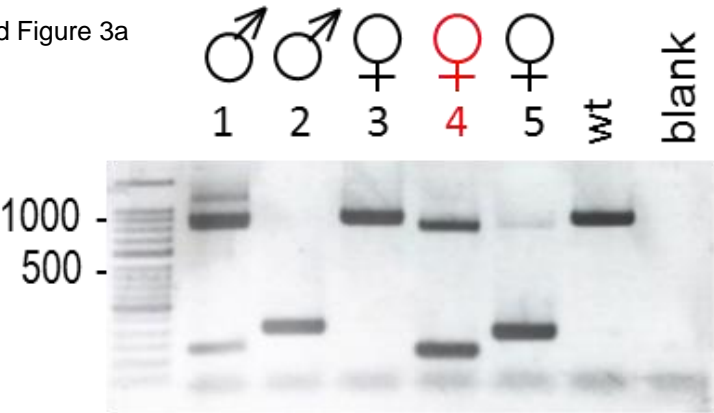

*inverted grayscale was used  
for better band visibility*

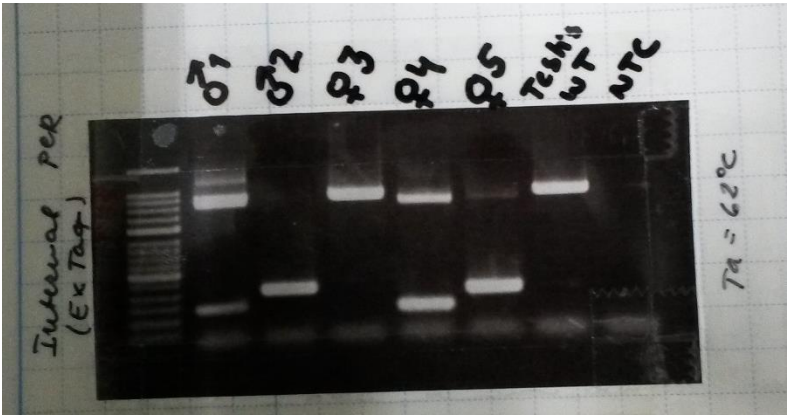

Supplement: Source Data Extended Data Fig. 3 — Unprocessed gels. [file 41556_2021_746_MOESM15_ESM.pdf]
