# Supplementary material for: Formation of spermatogonia and fertile oocytes in golden hamsters requires piRNAs
Source: Nat Cell Biol. 2021 Sep 6;23(9):992–1001. doi: 10.1038/s41556-021-00746-2 (PMC8437802; doi:10.1038/s41556-021-00746-2)
Supplement: Source Data Fig. 2 — Unprocessed gels. [file 41556_2021_746_MOESM8_ESM.pdf]

Figure 2g

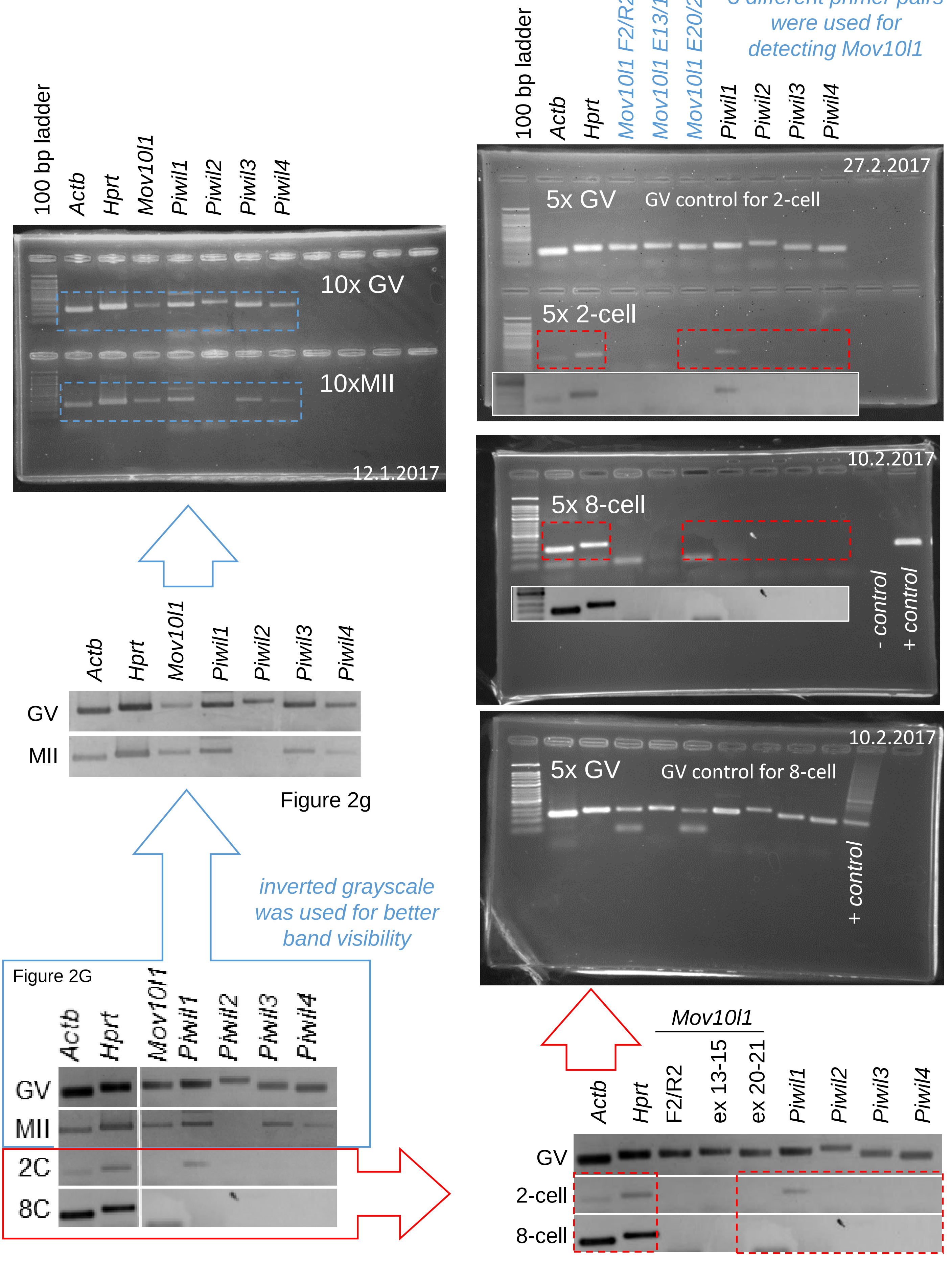

3 different primer pairs were used for detecting Mov10l1
100 bp ladder
Actb
Hprt
Mov10l1 F2/R2
Mov10l1 E13/15
Mov10l1 E20/21
Piwil1
Piwil2
Piwil3
Piwil4
27.2.2017
5x GV
GV control for 2-cell
5x 2-cell
10x GV
10xMII
12.1.2017
Mov10l1
10.2.2017
5x 8-cell
- control
+ control
5x GV
GV control for 8-cell
GV
MII
Figure 2g
inverted grayscale was used for better band visibility
Figure 2G
2C
8C
F2/R2
ex 13-15
ex 20-21
2-cell
8-cell
