# Supplementary material for: Formation of spermatogonia and fertile oocytes in golden hamsters requires piRNAs
Source: Nat Cell Biol. 2021 Sep 6;23(9):992–1001. doi: 10.1038/s41556-021-00746-2 (PMC8437802; doi:10.1038/s41556-021-00746-2)
Supplement: Supplementary file 3 — Peer Review Information [file 41556_2021_746_MOESM3_ESM.pdf]

natureresearch

Peer Review Information

Journal: Nature Cell Biology
Manuscript Title: Formation of hamster's spermatogonia and fertile oocytes requires piRNAs
Corresponding author name(s): Atsuo Ogura, Petr Svoboda

Reviewer Comments & Decisions:

Decision Letter, initial version:

Date: 16th April 21 09:47:55
Last Sent: 16th April 21 09:47:55
From: jie.wang@nature.com
To: svobodap@img.cas.cz
CC: ncb@springernature.com
Subject: Decision on Nature Cell Biology submission NCB-S44937-T
Message: *Please delete the link to your author homepage if you wish to forward this email to co-authors.

Dear Professor Svoboda,

Your manuscript, "Formation of hamster's spermatogonia and fertile oocytes requires piRNAs", has now been seen by 2 referees, who are experts in piRNA and germ cells (referee 1) and piRNA (referee 3). As you will see from their comments (attached below) they find this work of potential interest, but have raised substantial concerns, which in our view would need to be addressed with considerable revisions before we can consider publication in Nature Cell Biology. We have also engaged an additional piRNA expert (referee 2). Unfortunately, this referee hasn't submitted the report yet. We will forward the report if/when we receive it.

Nature Cell Biology editors discuss the referee reports in detail within the editorial team, including the chief editor, to identify key referee points that should be addressed with priority, and requests that are overruled as being beyond the scope of the current study. To guide the scope of the revisions, I have listed these points below. We are committed to providing a fair and constructive peer-review process, so please feel free to contact me if you would like to discuss any of the referee comments further.

In particular, it would be essential to:

a) profile small RNAs in in MOV10L1 mutants, as noted by referee 3:

Small RNAs are not sequenced in MOV10L1 mutants. Small RNAs are sequenced in wild type testes at 9, 13, and 21 dpp, but surprisingly not in MOV10L1 mutant. It is assumed that piRNAs are absent in hamster MOV10L1 mutants, because it is required for piRNA production in mouse. Direct experimental evidence is necessary to demonstrate this. Small RNAs need to be sequenced in MOV10L1-/- oocytes and MOV10L1-/- 9 dpp testes to see if MOV10L1 is essential for piRNA production in golden hamster. Such data will strengthen this study. Without it, this opportunity is missed.

b) provide further evidence to demonstrate that post-zygotic Mov10l1–/– phenotype is a maternal effect, as noted by referee 1:

2) Line 144: the mere fact that Mov10l1 and Piwil are maternally expressed does not show that the phenotype is a maternal effect. When is zygotic transcription initiated in hamster? If that only starts later, than that fact would show it is a maternal effect. If it starts before 2 cell, the authors need to show absence of zygotic expression to claim they describe a maternal effect. To be fair, it is a rather minor point, and I agree that the effect very likely is maternal, but it needs to be treated correctly. Also, only RNA is examined. It would be good if the authors could also demonstrate protein expression in this context.

c) reconcile the discrepancy between the current study and the study by Siomi and colleagues regarding whether deficiency in the piRNA pathway leads to meiosis defect, as noted by referee 1:

1) Line 142: Hasuwa et al. (the companion paper) describes an MII defect. Even though this defect is minor, the two manuscripts should be concise in citing each other. In fact, given that the mov10l1 mutant can be expected to be more severe than the piwil3 mutant, this discrepancy is remarkable and should be resolved. Is this a real difference between the two mutants, is a phenotype missed in the mov10l1 mutant, or is the phenotype in piwil3 mutants not really there? Did the authors check spindle sizes in mov10l1 mutant oocytes?

d) All other referee concerns pertaining to strengthening existing data, providing controls, methodological details, clarifications and textual changes, should also be addressed.

e) Finally please pay close attention to our guidelines on statistical and methodological reporting (listed below) as failure to do so may delay the reconsideration of the revised manuscript. In particular please provide:

- a Supplementary Figure including unprocessed images of all gels/blots in the form of a multi-page pdf file. Please ensure that blots/gels are labeled and the sections presented in the figures are clearly indicated.

- a Supplementary Table including all numerical source data in Excel format, with data for different figures provided as different sheets within a single Excel file. The file should include source data giving rise to graphical representations and statistical descriptions in the paper and for all instances where the figures present representative experiments of multiple independent repeats, the source data of all repeats should be provided.

We would be happy to consider a revised manuscript that would satisfactorily address these points, unless a similar paper is published elsewhere, or is accepted for publication in Nature Cell Biology in the meantime.

When revising the manuscript please:

- ensure that it conforms to our format instructions and publication policies (see below and https://www.nature.com/nature/for-authors).

- provide a point-by-point rebuttal to the full referee reports verbatim, as provided at the end of this letter.

- provide the completed Reporting Summary (found here https://www.nature.com/documents/nr-reporting-summary.pdf). This is essential for reconsideration of the manuscript will be available to editors and referees in the event of peer review. For more information see http://www.nature.com/authors/policies/availability.html or contact me.

When submitting the revised version of your manuscript, please pay close attention to our href="https://www.nature.com/nature-research/editorial-policies/image-integrity">Digital Image Integrity Guidelines. and to the following points below:

-- that unprocessed scans are clearly labelled and match the gels and western blots presented in figures.
-- that control panels for gels and western blots are appropriately described as loading on sample processing controls
-- all images in the paper are checked for duplication of panels and for splicing of gel lanes.

Finally, please ensure that you retain unprocessed data and metadata files after publication, ideally archiving data in perpetuity, as these may be requested during the peer review and production process or after publication if any issues arise.

Nature Cell Biology is committed to improving transparency in authorship. As part of our efforts in this direction, we are now requesting that all authors identified as 'corresponding author' on published papers create and link their Open Researcher and Contributor Identifier (ORCID) with their account on the Manuscript Tracking System (MTS), prior to acceptance. ORCID helps the scientific community achieve unambiguous attribution of all scholarly contributions. You can create and link your ORCID from the home page of the MTS by clicking on 'Modify my Springer Nature account'. For more information please visit please visit www.springernature.com/orcid.

This journal strongly supports public availability of data. Please place the data used in your paper into a public data repository, or alternatively, present the data as Supplementary Information. If data can only be shared on request, please explain why in your Data Availability Statement, and also in the correspondence with your editor. Please note that for some data types, deposition in a public repository is mandatory - more information on our data deposition policies and available repositories appears below.

Please submit the revised manuscript files and the point-by-point rebuttal to the referee comments using this link:

[REDACTED]

*This url links to your confidential home page and associated information about manuscripts you may have submitted or be reviewing for us. If you wish to forward this email to co-authors, please delete the link to your homepage.

We would like to receive a revised submission within six months.

We hope that you will find our referees' comments, and editorial guidance helpful. Please do not hesitate to contact me if there is anything you would like to discuss.

Best wishes,

Jie Wang

Jie Wang, PhD

Senior Editor
Nature Cell Biology

Tel: +44 (0) 207 843 4924
email: jie.wang@nature.com

Reviewers' Comments:

Reviewer #1:
Remarks to the Author:
Loubalova et al. describe the phenotypes the golden hamster, following loss of the helicase Mov10l1, a known player in the mammalian piRNA pathway. This animal likely is a better model for human piRNA biology than the mouse, as the latter have a rather atypical upregulation of Dicer-mediated RNAi in the female germline, something that is absent in most other mammals. In addition, previous work had shown that a specific Piwi protein, Piwil3 is specifically expressed in bovine oocytes, followed by studies in human oocytes that confirmed this finding, and expanded it by demonstrating Hiwi3 binds piRNAs. The manuscript by Loubalova et al. is very important, as it demonstrates the relevance of these female piRNAs and Piwi proteins for female fertility. This is a very important conclusion, as it provides the final piece of evidence that piRNAs are most certainly relevant in female reproduction. In addition to this insight, this manuscript delves into the hamster TE content, demonstrates nicely that also in males piRNA mediated silencing displays differences when compared with mice. A specific TE is pinpointed as the likely culprit behind the earlier spermatogenesis defect in hamster compared to mouse: MYSERV. I found this part of the manuscript particularly clear and interesting.
The authors conclude that the piRNA pathway is highly adaptive to novel TEs, and that phenotypes associated with piRNA loss will be affected by the specific biology of the dominant TE in the studied animal. Even though the reasons behind the female infertility of mov10l1 mutant hamsters remains unclear, I believe this manuscript is a very strong candidate for NCB, and I strongly support its publication therein, when the authors have addressed the following issues:

1) Line 142: Hasuwa et al. (the companion paper) describes an MII defect. Even though this defect is minor, the two manuscripts should be concise in citing each other. In fact, given that the mov10l1 mutant can be expected to be more severe than the piwil3 mutant, this discrepancy is remarkable and should be resolved. Is this a real difference between the two mutants, is a phenotype missed in the mov10l1 mutant, or is the phenotype in piwil3 mutants not really there? Did the authors check spindle sizes in mov10l1 mutant oocytes?
2) Line 144: the mere fact that Mov10l1 and Piwil are maternally expressed does not show that the phenotype is a maternal effect. When is zygotic transcription initiated in hamster? If that only starts

later, than that fact would show it is a maternal effect. If it starts before 2 cell, the authors need to show absence of zygotic expression to claim they describe a maternal effect. To be fair, it is a rather minor point, and I agree that the effect very likely is maternal, but it needs to be treated correctly. Also, only RNA is examined. It would be good if the authors could also demonstrate protein expression in this context.
3) Lines160-162: the authors conclude that IAPs are only partially released in mov10l1 mutant oocytes, based on a 2-3 fold higher activation of these elements in mouse mili mutants. I do not believe one can make that comparison. It may well be that full activation of IAPs in hamster results in lower expression than in mice. A claim to only partial release would require the demonstration of residual repression, and not only a comparison to a value from another species. Especially when the difference is only 2-3 fold (and not several orders of magnitude).
4) Line 165: what exactly was BS sequenced? And what was the average coverage. Only a short statement on the latter is given: "covered only a fraction of the hamster genome …". This seems to indicate that the average coverage is in fact below 1, which would prevent any detection of DNA methylation changes, except maybe global loss over extensive loci. If this is the case, I do not believe that this analysis is very informative. In any case, results as depicted in 2H and S11 do not seem to be warranted, as they must rely on the piling up of multi-mapping reads on a consensus sequence of a TE. Either more extensive sequencing will need to be done, or validation with cheaper amplicon BS sequencing needs to be done.
5) Figure 3E, but also other IF panels, need better visualization. The defect described in Line 200, referring to 3E, is not clear in the provided images (at least not to me). Higher magnification and better annotation are required.
6) Line 225: the small effects are being explained by arguing that genes may also be expressed in the somatic cells. This should be tested with smFISH for a few genes.

Minor points:
7) Line 64: It should be made clear that previous studies showed Piwil3 in present specifically in oocytes (Roovers et al. 2015 and Yang et al. 2019), already suggesting a major difference between piRNA biology in mouse oocytes versus other mammals.
8) "…as for them THERE was…"
9) Line 117: Figure S4D does not provide data that supports the statement. S4D is about substitution rates in L1s, while the text talks about absolute numbers of elements in the genomes of various species.
10) Line 152: the writing confused me. It occurred to me that the difference that is pointed out is that Hasuwa et al. look at MII stage while Loubalova et al. look at an earlier, pre-ovulation stage. If that is spelled out in this sentence that would help a lot.
11) Line 158: a 'space' is missing before "RNA-seq"
12) Lines 230-250: the authors should not over-interpret the results. Causality between TE effects and phenotypes are not demonstrated. Wording should be toned down a bit.

Reviewer #2:
None

Reviewer #3:
Remarks to the Author:
The manuscript “Formation of hamster’s spermatogonia and fertile oocytes requires piRNAs” submitted by Loubalova et al. reported that loss of MOV10L1 helicase, an essential protein in piRNA biogenesis, generates novel phenotypes in male and female hamsters compared with mice. The authors chose golden hamster as a model to study the essential roles of piRNAs in spermatogenesis and oogenesis because of its similarity with human. Golden hamster has four distinct PIWI proteins, including PIWIL1, PIWIL2, PIWIL3 and PIWIL4. Unlike mice, mutant male hamsters have impaired establishment of spermatogonia. More strikingly, the female mutant hamsters are infertile while female mutant mice are fertile. Overall, this study has logical and nice design and the phenotypes are convincing. The significance is two-fold. First, this is the first study of the function of MOV10L1 in a non-mouse mammalian model. Second, mouse MOV10L1/PIWI mutants do not display female fertility defects. This study shows that MOV10L1 in golden hamster is important for female fertility. This finding is novel and may be relevant to human. However, the mechanisms of the sterile phenotypes for both males and females are not clear, which tempered the significance of the study.

Major concerns:
1) Small RNAs are not sequenced in MOV10L1 mutants. Small RNAs are sequenced in wild type testes at 9, 13, and 21 dpp, but surprisingly not in MOV10L1 mutant. It is assumed that piRNAs are absent in hamster MOV10L1 mutants, because it is required for piRNA production in mouse. Direct experimental evidence is necessary to demonstrate this. Small RNAs need to be sequenced in MOV10L1-/- oocytes and MOV10L1-/- 9 dpp testes to see if MOV10L1 is essential for piRNA production in golden hamster. Such data will strengthen this study. Without it, this opportunity is missed.

2) In Abstract, the statement “Our results not only refute the notion that in mammals the piRNA pathway is important only in males” is not accurate. Line 177: “…and proves wrong the notion that in mammals the piRNA pathway is important only in males.” This notion is not widely held in the field. On the contrary, it is widely accepted that mouse oocyte has evolved the oocyte-specific dicer isoform and the siRNA pathway to suppress TEs and thus does not need the piRNA pathway. It is widely believed that mouse is an exception rather than the rule. These sentences need to be deleted or revised. Indeed, this study and a PIWI study in preprint are the first to show the importance of piRNA pathways in mammalian female fertility.

3) RNA-seq of 9 dpp testes in Fig 4A. The RNA-seq results was very likely complicated by the different cellular composition between MOV10L1-/- and WT. The KO testes likely contained fewer germ cells than wild type. This is a common problem for RNA-seq analysis using whole testes. This caveat needs to be acknowledged.

4) Lines 147-154: The authors found that compared with MOV10L1+/+ and MOV10L1+/– profiling, only 13 genes increased significantly in MOV10L1–/– oocytes, and concluded that “even small transcriptome changes in MOV10L1–/– oocytes still could contribute to the observed developmental incompetence”. However, if I understand Fig. S9C correctly, 25 genes were upregulated in MOV10L1+/– compared with MOV10L1+/+, and presumably MOV10L1+/– females are fertile. Therefore, it is not convincing to make the conclusion that the minor transcriptome change in the mutant causes the infertile phenotype in the mutant females.

5) The mutant oocytes were able to ovulate and mutant MII eggs can be fertilized but fail to support development after fertilization. It would be more informative to know at which stage the embryos cease developing.

Minor concerns:
1) Some figure legends and text descriptions are too simple to understand, and it might be due to the page or word limit. Specifically, in Lines 100-101, it was said that “MYSERV and IAP-derived piRNAs were the most abundant piRNAs targeting a transposable element (Fig. 1C and S4A)”, however, clearly we can see ORR1 has higher abundance than IAP in Fig. 1C. Another example is that the authors used Fig. S4D to indicate that the number of L1 elements are comparable to human genome but smaller than rat genome, however, I did not see the direct information in Fig. S4D or its figure legend.

2) Lines 120: How were young retrotransposons defined? I believe the result is true since it has the similar conclusion with a preprint paper published by Hasuwa et al, 2021. But more detailed information is necessary for clarity.

3) Lines 241-242: It would be more convincing to perform real-time PCR to confirm the upregulation of MYSERV retrotransposons in the mutant testes.

GUIDELINES FOR MANUSCRIPT SUBMISSION TO NATURE CELL BIOLOGY

READABILITY OF MANUSCRIPTS – Nature Cell Biology is read by cell biologists from diverse backgrounds, many of whom are not native English speakers. Authors should aim to communicate their findings clearly, explaining technical jargon that might be unfamiliar to non-specialists, and avoiding non-standard abbreviations. Titles and abstracts should concisely communicate the main findings of the study, and the background, rationale, results and conclusions should be clearly explained in the manuscript in a manner accessible to a broad cell biology audience. Nature Cell Biology uses British spelling.

MANUSCRIPT FORMAT – please follow the guidelines listed in our Guide to Authors regarding manuscript formats at Nature Cell Biology.

TITLE – should be no more than 100 characters including spaces, without punctuation and avoiding technical terms, abbreviations, and active verbs..

AUTHOR NAMES – should be given in full.

AUTHOR AFFILIATIONS – should be denoted with numerical superscripts (not symbols) preceding the names. Full addresses should be included, with US states in full and providing zip/post codes. The corresponding author is denoted by: "Correspondence should be addressed to [initials]."

ABSTRACT AND MAIN TEXT – please follow the guidelines that are specific to the format of your manuscript, as listed in our Guide to Authors (http://www.nature.com/ncb/pdf/ncb_gta.pdf) Briefly, Nature Cell Biology Articles, Resources and Technical Reports have 3500 words, including a 150 word abstract, and the main text is subdivided in Introduction, Results, and Discussion sections. Nature Cell Biology Letters have up to 2500 words, including a 180 word introductory paragraph (abstract), and the text is not subdivided in sections.

ACKNOWLEDGEMENTS – should be kept brief. Professional titles and affiliations are unnecessary. Grant numbers can be listed.

AUTHOR CONTRIBUTIONS – must be included after the Acknowledgements, detailing the contributions of each author to the paper (e.g. experimental work, project planning, data analysis etc.). Each author should be listed by his/her initials.

FINANCIAL AND NON-FINANCIAL COMPETING INTERESTS – the authors must include one of three declarations: (1) that they have no financial and non-financial competing interests; (2) that they have financial and non-financial competing interests; or (3) that they decline to respond, after the Author Contributions section. This statement will be published with the article, and in cases where financial and non-financial competing interests are declared, these will be itemized in a web supplement to the article. For further details please see https://www.nature.com/licenceforms/nrg/competing-interests.pdf.

REFERENCES – are limited to a total of 70 for Articles, Resources, Technical Reports; and 40 for Letters. This includes references in the main text and Methods combined. References must be numbered sequentially as they appear in the main text, tables and figure legends and Methods and must follow the precise style of Nature Cell Biology references. References only cited in the Methods should be numbered consecutively following the last reference cited in the main text. References only associated with Supplementary Information (e.g. in supplementary legends) do not count toward the total reference limit and do not need to be cited in numerical continuity with references in the main text. Only published papers can be cited, and each publication cited should be included in the numbered reference list, which should include the manuscript titles. Footnotes are not permitted.

METHODS – Nature Cell Biology publishes methods online. The methods section should be provided as a separate Word document, which will be copyedited and appended to the manuscript PDF, and incorporated within the HTML format of the paper.

Methods should be written concisely, but should contain all elements necessary to allow interpretation and replication of the results. As a guideline, Methods sections typically do not exceed 3,000 words. The Methods should be divided into subsections listing reagents and techniques. When citing previous methods, accurate references should be provided and any alterations should be noted. Information must be provided about: antibody dilutions, company names, catalogue numbers and clone numbers for monoclonal antibodies; sequences of RNAi and cDNA probes/primers or company names and catalogue numbers if reagents are commercial; cell line names, sources and information on cell line identity and authentication. Animal studies and experiments involving human subjects must be reported in detail, identifying the committees approving the protocols. For studies involving human subjects/samples, a statement must be included confirming that informed consent was obtained. Statistical analyses and information on the reproducibility of experimental results should be provided in a section titled “Statistics and Reproducibility”.

All Nature Cell Biology manuscripts submitted on or after March 21 2016 must include a Data availability statement as a separate section after Methods but before references, under the heading "Data Availability”. . For Springer Nature policies on data availability see http://www.nature.com/authors/policies/availability.html; for more information on this particular

policy see http://www.nature.com/authors/policies/data/data-availability-statements-data-citations.pdf. The Data availability statement should include:

• Accession codes for primary datasets (generated during the study under consideration and designated as "primary accessions") and secondary datasets (published datasets reanalysed during the study under consideration, designated as "referenced accessions"). For primary accessions data should be made public to coincide with publication of the manuscript. A list of data types for which submission to community-endorsed public repositories is mandated (including sequence, structure, microarray, deep sequencing data) can be found here http://www.nature.com/authors/policies/availability.html#data.

• Unique identifiers (accession codes, DOIs or other unique persistent identifier) and hyperlinks for datasets deposited in an approved repository, but for which data deposition is not mandated (see here for details http://www.nature.com/sdata/data-policies/repositories).

• At a minimum, please include a statement confirming that all relevant data are available from the authors, and/or are included with the manuscript (e.g. as source data or supplementary information), listing which data are included (e.g. by figure panels and data types) and mentioning any restrictions on availability.

• If a dataset has a Digital Object Identifier (DOI) as its unique identifier, we strongly encourage including this in the Reference list and citing the dataset in the Methods.

We recommend that you upload the step-by-step protocols used in this manuscript to the Protocol Exchange. More details can found at www.nature.com/protocolexchange/about.

DISPLAY ITEMS – main display items are limited to 6-8 main figures and/or main tables for Articles, Resources, Technical Reports; and 5 main figures and/or main tables for Letters. For Supplementary Information see below.

FIGURES – Colour figure publication costs $600 for the first, and $300 for each subsequent colour figure. All panels of a multi-panel figure must be logically connected and arranged as they would appear in the final version. Unnecessary figures and figure panels should be avoided (e.g. data presented in small tables could be stated briefly in the text instead).

All imaging data should be accompanied by scale bars, which should be defined in the legend.
Cropped images of gels/blots are acceptable, but need to be accompanied by size markers, and to retain visible background signal within the linear range (i.e. should not be saturated). The boundaries of panels with low background have to be demarked with black lines. Splicing of panels should only be considered

if unavoidable, and must be clearly marked on the figure, and noted in the legend with a statement on whether the samples were obtained and processed simultaneously. Quantitative comparisons between samples on different gels/blots are discouraged; if this is unavoidable, it should only be performed for samples derived from the same experiment with gels/blots were processed in parallel, which needs to be stated in the legend.

Figures should be provided at approximately the size that they are to be printed at (single column is 86 mm, double column is 170 mm) and should not exceed an A4 page (8.5 x 11"). Reduction to the scale that will be used on the page is not necessary, but multi-panel figures should be sized so that the whole figure can be reduced by the same amount at the smallest size at which essential details in each panel are visible. In the interest of our colour-blind readers we ask that you avoid using red and green for contrast in figures. Replacing red with magenta and green with turquoise are two possible colour-safe alternatives. Lines with widths of less than 1 point should be avoided. Sans serif typefaces, such as Helvetica (preferred) or Arial should be used. All text that forms part of a figure should be rewritable and removable.

We accept files from the following graphics packages in either PC or Macintosh format:

- For line art, graphs, charts and schematics we prefer Adobe Illustrator (.AI), Encapsulated PostScript (.EPS) or Portable Document Format (.PDF). Files should be saved or exported as such directly from the application in which they were made, to allow us to restyle them according to our journal house style.

- We accept PowerPoint (.PPT) files if they are fully editable. However, please refrain from adding PowerPoint graphical effects to objects, as this results in them outputting poor quality raster art. Text used for PowerPoint figures should be Helvetica (preferred) or Arial.

- We do not recommend using Adobe Photoshop for designing figures, but we can accept Photoshop generated (.PSD or .TIFF) files only if each element included in the figure (text, labels, pictures, graphs, arrows and scale bars) are on separate layers. All text should be editable in 'type layers' and line-art such as graphs and other simple schematics should be preserved and embedded within 'vector smart objects' - not flattened raster/bitmap graphics.

- Some programs can generate Postscript by 'printing to file' (found in the Print dialogue). If using an application not listed above, save the file in PostScript format or email our Art Editor, Allen Beattie for advice (a.beattie@nature.com).

Regardless of format, all figures must be vector graphic compatible files, not supplied in a flattened raster/bitmap graphics format, but should be fully editable, allowing us to highlight/copy/paste all text and move individual parts of the figures (i.e. arrows, lines, x and y axes, graphs, tick marks, scale bars

etc.). The only parts of the figure that should be in pixel raster/bitmap format are photographic images or 3D rendered graphics/complex technical illustrations.

All placed images (i.e. a photo incorporated into a figure) should be on a separate layer and independent from any superimposed scale bars or text. Individual photographic images must be a minimum of 300+ DPI (at actual size) or kept constant from the original picture acquisition and not decreased in resolution post image acquisition. All colour artwork should be RGB format.

FIGURE LEGENDS – must not exceed 350 words for each figure to allow fit on a single printed NCB page together with the figure. They must include a brief title for the whole figure, and short descriptions of each panel with definitions of the symbols used, but without detailing methodology.

TABLES – main tables should be provided as individual Word files, together with a brief title and legend. For supplementary tables see below.

SUPPLEMENTARY INFORMATION – Supplementary information is material directly relevant to the conclusion of a paper, but which cannot be included in the printed version in order to keep the manuscript concise and accessible to the general reader. Supplementary information is an integral part of a Nature Cell Biology publication and should be prepared and presented with as much care as the main display item, but it must not include non-essential data or text, which may be removed at the editor's discretion. All supplementary material is fully peer-reviewed and published online as part of the HTML version of the manuscript. Supplementary Figures and Supplementary Notes are appended at the end of the main PDF of the published manuscript.

Supplementary items should relate to a main text figure, wherever possible, and should be mentioned sequentially in the main manuscript, designated as Supplementary Figure, Table, Video, or Note, and numbered continuously (e.g. Supplementary Figure 1, Supplementary Figure 2, Supplementary Table 1, Supplementary Table 2 etc.).

Unprocessed scans of all key data generated through electrophoretic separation techniques need to be presented in a supplementary figure that should be labelled and numbered as the final supplementary figure, and should be mentioned in every relevant figure legend. This figure does not count towards the total number of figures and is the only figure that can be displayed over multiple pages, but should be provided as a single file, in PDF or TIFF format. Data in this figure can be displayed in a relatively informal style, but size markers and the figures panels corresponding to the presented data must be indicated.

The total number of Supplementary Figures (not including the “unprocessed scans” Supplementary Figure) should not exceed the number of main display items (figures and/or tables (see our Guide to Authors and March 2012 editorial http://www.nature.com/ncb/authors/submit/index.html#suppinfo; http://www.nature.com/ncb/journal/v14/n3/index.html#ed). No restrictions apply to Supplementary Tables or Videos, but we advise authors to be selective in including supplemental data.

Each Supplementary Figure should be provided as a single page and as an individual file in one of our accepted figure formats and should be presented according to our figure guidelines (see above). Supplementary Tables should be provided as individual Excel files. Supplementary Videos should be provided as .avi or .mov files up to 50 MB in size. Supplementary Figures, Tables and Videos much be accompanied by a separate Word document including titles and legends.

GUIDELINES FOR EXPERIMENTAL AND STATISTICAL REPORTING

REPORTING REQUIREMENTS – We are trying to improve the quality of methods and statistics reporting in our papers. To that end, we are now asking authors to complete a reporting summary that collects information on experimental design and reagents. The Reporting Summary can be found here https://www.nature.com/documents/nr-reporting-summary.pdf)If you would like to reference the guidance text as you complete the template, please access these flattened versions at http://www.nature.com/authors/policies/availability.html.

STATISTICS – Wherever statistics have been derived the legend needs to provide the n number (i.e. the sample size used to derive statistics) as a precise value (not a range), and define what this value represents. Error bars need to be defined in the legends (e.g. SD, SEM) together with a measure of centre (e.g. mean, median). Box plots need to be defined in terms of minima, maxima, centre, and percentiles. Ranges are more appropriate than standard errors for small data sets. Wherever statistical significance has been derived, precise p values need to be provided and the statistical test used needs to be stated in the legend. Statistics such as error bars must not be derived from n<3. For sample sizes of n<5 please plot the individual data points rather than providing bar graphs. Deriving statistics from technical replicate samples, rather than biological replicates is strongly discouraged. Wherever statistical significance has been derived, precise p values need to be provided and the statistical test stated in the legend.

Information on how many times each experiment was repeated independently with similar results needs to be provided in the legends and/or Methods for all experiments, and in particular wherever representative experiments are shown.

We strongly recommend the presentation of source data for graphical and statistical analyses as a separate Supplementary Table, and request that source data for all independent repeats are provided when representative experiments of multiple independent repeats, or averages of two independent experiments are presented. This supplementary table should be in Excel format, with data for different figures provided as different sheets within a single Excel file. It should be labelled and numbered as one of the supplementary tables, titled “Statistics Source Data”, and mentioned in all relevant figure legends.

--------- Please don't hesitate to contact NCB@nature.com should you have queries about any of the above requirements ---------

Author Rebuttal to Initial comments

Reviewer #1

Remarks to the Author:

Loubalova et al. describe the phenotypes the golden hamster, following loss of the helicase Mov10l1, a known player in the mammalian piRNA pathway. This animal likely is a better model for human piRNA biology than the mouse, as the latter have a rather atypical upregulation of Dicer-mediated RNAi in the female germline, something that is absent in most other mammals. In addition, previous work had shown that a specific Piwi protein, Piwil3 is specifically expressed in bovine oocytes, followed by studies in human oocytes thatconfirmed this finding, and expanded it by demonstrating Hiwi3 binds piRNAs. The manuscript by Loubalova et al. is very important, as it demonstrates the relevance of these female piRNAs and Piwi proteins for female fertility. This is a very important conclusion, asit provides the final piece of evidence that piRNAs are most certainly relevant in female reproduction. In addition to this insight, this manuscript delves into the hamster TE content, demonstrates nicely that also in males piRNA mediated silencing displays differences when compared with mice. A specific TE is pinpointed as the likely culprit behind the earlier spermatogenesis defect in hamster compared to mouse: MYSERV. I found this part of the manuscript particularly clear and interesting.

The authors conclude that the piRNA pathway is highly adaptive to novel TEs, and that phenotypes associated with piRNA loss will be affected by the specific biology of the dominant TE in the studied animal. Even though the reasons behind the female infertility ofmov10l1 mutant hamsters remains unclear, I believe this manuscript is a very strong candidate for NCB, and I strongly support its publication therein, when the authors have addressed the following issues:

1) Line 142: Hasuwa et al. (the companion paper) describes an MII defect. Even though this defect is minor, the two manuscripts should be concise in citing each other. In fact, given thatthe mov10l1 mutant can be expected to be more severe than the piwil3 mutant, this discrepancy is remarkable and should be resolved. Is this a real difference between the two mutants, is a phenotype missed in the mov10l1 mutant, or is the phenotype in piwil3 mutantsnot really there? Did the authors check spindle sizes in mov10l1 mutant oocytes?

The hamster *Mov10l*$^{-/-}$ MII spindle appearance was discussed earlier with two

experts on spindle formation, Melina Schuh and Petr Solc, and during the revision with Haruhiko Siomiand Atsuo Ogura, the co-author of this manuscript. The consensus was that *Mov10l*$^{-/-}$ oocyte spindles do not show an apparent defect.

For the revision, we produced 3D reconstructions from confocal data and estimated spindle'slength and volume (defined by tubulin signal) as well as volume of the metaphase plate (defined by DNA (DAPI) signal). We provide these data in a revised supplementary Figure S5; please, note that labeling of *Mov10l*$^{-/-}$ and *Mov10l1*$^{+/+}$ in the previous supplementary figure was accidentally inversed when the figure was converted to a landscape format.

Quantification did not reveal a significant spindle phenotype in the *Mov10l1*$^{-/-}$ mutant. Although chromosomes appeared many times larger relative to the spindle (Fig. S5A), the volume of chromosomes was not statistically different. Dr. Ogura pointed out that accordingto his experience, hamster MII spindles are highly sensitive to manipulations and temperature, hence we cannot rule out that the variability of the volume of the spindles (Fig.S5C) was affected by sample preparation in particular experiments.

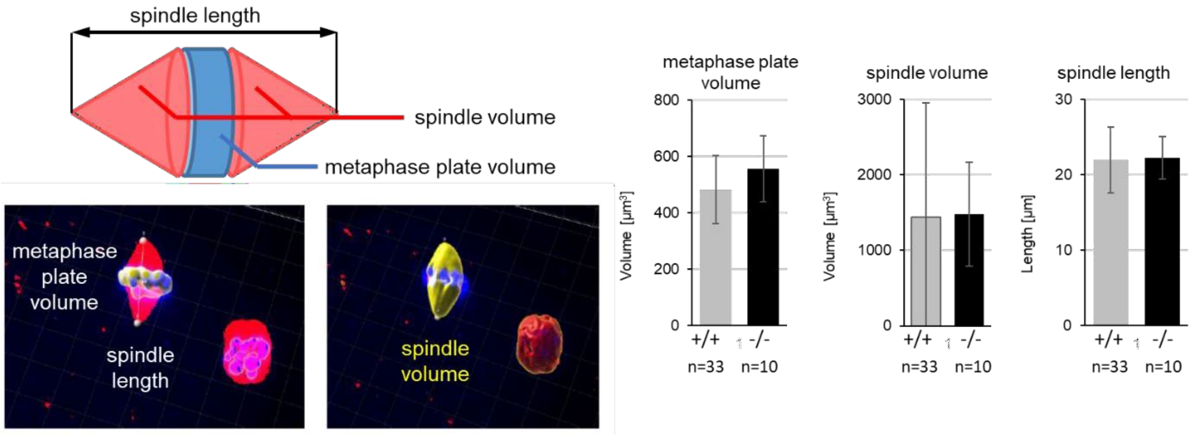

Regarding the phenotypic differences between *Mov10l1* and *Piwil* mutants, it may have a biological cause and *Mov10l1* mutants do not have to necessarily exhibit stronger phenotypes. Maternal piRNA population in *Mov10l1* mutants is affected differently than those in *Piwil1* and *Piwil3* mutants. In *Piwil* mutants, piRNA populations would be reshapedcorrespondingly to the lost PIWIL protein. In *Mov10l1* mutants, oocyte piRNAs are not lost completely like piRNAs in 9 dpp testes (compare Fig. S8D and S13B). Some specific piRNAs remain produced in the oocyte despite the absence of MOV10L1 and could also account for phenotypic

differences between the piRNA pathway mutants. While we disclosethis in the manuscript, we consider the issue of oocyte-specific Mov10L1-independent piRNAs beyond the scope of the article and we plan to address it separately.

2) Line 144: the mere fact that Mov10l1 and Piwil are maternally expressed does not show that the phenotype is a maternal effect. When is zygotic transcription initiated in hamster? If that only starts later, than that fact would show it is a maternal effect. If it starts before 2 cell,the authors need to show absence of zygotic expression to claim they describe a maternal effect. To be fair, it is a rather minor point, and I agree that the effect very likely is maternal,but it needs to be treated correctly. Also, only RNA is examined. It would be good if the authors could also demonstrate protein expression in this context.

We used the term maternal effect because this was the case where the phenotype was determined by the genotype of the mother and not by the genotype of the zygote. *Mov10l1*$^{-/-}$ females mated with *Mov10l1*$^{+/-}$ males produce zygotes but no progeny. A half of those zygotes that do not develop to the term would be *Mov10l1*$^{+/-}$, the other half *Mov10l1*$^{-/-}$. At thesame time, a cross of two *Mov10l1*$^{+/-}$ animals yields normal Mendelian segregation (Fig. 1H) demonstrating normal viability and development to the term of *Mov10l1*$^{+/-}$ and *Mov10l1*$^{-/-}$ zygotes arising from heterozygous mothers. This concept of the maternal effect designation isindependent of the zygotic genome activation (ZGA) timing and elimination of the *Mov10l1* and *Piwil1-4* gene products – such features would be related to the mechanism of the effect.

Regarding the phenotype onset and relationship with piRNA pathway factors. ZGA in goldenhamsters takes place at the 2-cell stage (DOI: 10.1002/mrd.1080320307). *Mov10l1* and *Piwil1-4* genes are expressed maternally and, with exception of the *Piwil1* transcript, their transcripts are not detected at the 2-cell stage (Fig. 2D). Unfortunately, MOV10L1 antibody used in Fig. 1 did not work well for immunofluorescent staining of hamster cells, thus wecould not address the protein level in zygotes.

To address the reviewer’s comment, we clarified our reasoning for designating the phenotypea maternal effect and description of the phenotype onset, ZGA timing and maternal expression of *Mov10l1* and *Piwil* genes. In the original version, the maternal effect was brought up in the context of expression of piRNA factors, which could have been misleading.In the revised version, we bring up the maternal effect properly in the context of the genotype.

3) Lines160-162: the authors conclude that IAPs are only partially released in

mov10l1 mutant oocytes, based on a 2-3 fold higher activation of these elements in mouse Mili mutants. I do not believe one can make that comparison. It may well be that full activation ofIAPs in hamster results in lower expression than in mice. A claim to only partial release would require the demonstration of residual repression, and not only a comparison to a valuefrom another species. Especially when the difference is only 2-3 fold (and not several orders of magnitude).

The notion of partial release of IAP repression comes from additional observations than the relatively low fold-change. First, detailed analysis of the coverage of full-length intact (FLI)IAP elements revealed incomplete coverage of most FLI elements by perfectly mapping reads, suggesting that FLI elements are actually not expressed (at least not above the detection limit for expression by RNA-seq). Second, we did not observe IAP signal upon staining oocytes with the same antibody, which detected IAP signal at 9 dpp testes. Third, bisulfite sequencing data do not indicate a global reduction of IAP methylation. To address the comment, we revised the text – the revised version does not make a direct comparison with IAP expression changes in mouse mutants as that would be indeed difficult to interpret.

4) Line 165: what exactly was BS sequenced? And what was the average coverage. Only a short statement on the latter is given: "covered only a fraction of the hamster genome …". This seems to indicate that the average coverage is in fact below 1, which would prevent any detection of DNA methylation changes, except maybe global loss over extensive loci. If this is the case, I do not believe that this analysis is very informative. In any case, results as depicted in 2H and S11 do not seem to be warranted, as they must rely on the piling up of multi-mapping reads on a consensus sequence of a TE. Either more extensive sequencing willneed to be done, or validation with cheaper amplicon BS sequencing needs to be done.

We bisulfite-sequenced a sample of ten fully-grown oocytes, which corresponds to hypothetical 80 converted single stranded genomic DNA strands from which sequence information could be obtained. The genome coverage upon mapping the converted reads (depicted in Fig. S9A) was relatively low (~10%), primarily because of poor mapability of the sequenced reads to the genome. The resolution of such data obviously does not allow to detect methylation changes at unique loci and could reveal only global loss over large regionsor repetitive loci. We mentioned that in the original text at the end of the paragraph (line 173). In the revised text, we bring the comment on the limiting sequencing depth earlier.

In any case, we are convinced that analyzing IAP retrotransposons by sampling

multimapingreads mapped to the consensus sequence of full-length intact elements is equivalent to performing the traditional bisulfite sequencing of a retrotransposon subfamily – the main difference being that we first bisulfite-sequence the genome at random and then identify informative regions within inspected repetitive elements. In contrast, the traditional bisulfite sequencing chooses the sequence through primer design first and then samples bisulfite- treated sequences through PCR amplification and sequencing –for repetitive elements PCR amplification is actually an equivalent of sampling multimapping reads. However, the traditional bisulfite sequencing with PCR amplification, which reveals only what one amplifies from the bisulfite-converted genome, seemed a worse option for analyzing repetitive elements in Golden hamster oocytes where the information on its active retrotransposons was limited. Furthermore, bisulfite sequencing with PCR amplification is prone to clonal effects, especially when working with low amounts of material and multiple amplicons, which would require to split the limited amount of bisulfite-converted DNA into several PCR reactions. This would in turn reduce the number of available template moleculesin each PCR reaction and increase clonal effect risks. In contrast, duplicate genomic fragments in DNA-seq can be identified and removed from the analysis (they were).

In the end, we obtained 20-40x or even higher coverage of several regions in IAPLTR3 and IAPLTR4 elements, which is sufficient to determine whether the retrotransposition- competent IAP subfamilies are globally regulated by piRNA-dependent DNA methylation. Thus, while we agree that our global bisulfite sequencing data offer a limited insight into methylation changes, we believe it is still better to include them in the manuscript to show theabsence of massive loss of DNA methylation over repetitive elements, which yield abundant piRNAs in the germline,

note: We revised data displayed in Fig. 2H, the original figure depicted cytosines in CpG dinucleotides on + and – genome strands (consecutively), which was not mentioned in the figure legend and was causing striking up-and-down patterns in methylation levels. We combined the + and – strand data to depict specific CpG dinucleotides whose position in theconsensus sequence is indicated on the x-axis.

5) Figure 3E, but also other IF panels, need better visualization. The defect described in Line200, referring to 3E, is not clear in the provided images (at least not to me). Higher magnification and better annotation are required.

We revised the figures and their legends, including a higher magnification in Fig. 3E showingchanged distribution of the DDX4 signal.

6) Line 225: the small effects are being explained by arguing that genes may also beexpressed in the somatic cells. This should be tested with smFISH for a few genes.

Somatic expression can be inferred from RNA-seq data from 13 dpp and 21 dpp testes. As *Mov10l1* knock-outs at 13 dpp and 21 dpp are essentially "germ cell-free", well-detectable gene expression and unchanged or relatively increased transcript levels provide solid evidence of somatic expression. In Fig. S13D, we sort gene expression along two axes: on y-axis is shown relative change at 9 dpp, which seems to reflect gene expression changes in germ cells as 9 dpp testes contain comparable number of germ cells in *Mov10l1* mutants andwild types (see the newly added Fig. S12E). The x-axis then provides relative gene expression change in 13 dpp *Mov10l1*$^{-/-}$ testes. As such testes contain minimum surviving germ cells, germ cell-specific gene expression appears as strongly reduced in whole testes while somatic gene expression shows no change or even a relative increase. Distribution of genes with significant relative upregulation in 9 dpp mutant (red points) is concentrated above 0 relative change in 13 dpp testes. This implies that these genes are well-expressed in 13 dpp *Mov10l1*$^{-/-}$ (essentially germ cell-free) testes. Data from 21 dpp samples, which showthe same pattern, were also added.

Minor points:

7) Line 64: It should be made clear that previous studies showed Piwil3 in present specifically in oocytes (Roovers et al. 2015 and Yang et al. 2019), already suggesting a majordifference between piRNA biology in mouse oocytes versus other mammals.

The text was revised to accommodate this comment: *… mice lack PIWIL3, which binds 19-20nt piRNAs in human and golden hamster oocytes and exists in many mammals, suggesting a major difference between oocytes piRNA biology in mouse versus other mammals*[10,11,20].

8) "…as for them THERE

was…"Corrected.

9) Line 117: Figure S4D does not provide data that supports the statement. S4D is aboutsubstitution rates in L1s, while the text talks about absolute numbers of elements in the genomes of various species.

We are sorry for this inaccuracy, which arose during revision and length reduction where theoriginal meaning was referring to different L1 retrotransposon subfamilies

that expanded in mice and hamsters.

10) Line 152: the writing confused me. It occurred to me that the difference that is pointedout is that Hasuwa et al. look at MII stage while Loubalova et al. look at an earlier, pre- ovulation stage. If that is spelled out in this sentence that would help a lot.

The text was revised to: *We analyzed pre-ovulatory oocytes while Piwil1$^{-/-}$ analysis concerned a later MII stage where meiotic transcriptome remodeling could enhance transcriptome changes considered statistically not significant in fully grown oocytes*.

11) Line 158: a 'space' is missing before

"RNA-seq"Corrected

12) Lines 230-250: the authors should not over-interpret the results. Causality between TEeffects and phenotypes are not demonstrated. Wording should be toned down a bit.

We revised that part and added new data from day 0 sequencing of testes, which show thatthe loss of gene-targeting piRNAs during embryonic development does not cause a major transcriptome perturbation in germ cells. Thus, the onset of the phenotype correlates with major transcriptional activation of MYSERV elements. The revised part of the text reads:

De-repression of MYSERV retrotransposons, which is apparent already at day 0 (Fig. S14)implies genome-wide failure to silence MYSERV insertion loci, which could subsequently contribute to the failure of germ cells to form spermatogonia.

Reviewer #2

This paper is very straightforward and descriptive—mostly phenotypic analyses at cellular and molecular levels. Ordinarily, it would be difficult to imagine that such a paper merits publication in NCB. However, its findings are novel and paradigm-breaking—it changed thecurrent dogma in the PWIWI-piRNA field that this pathway is not required for female reproductive function. This dogma is mostly based on studies in mice. For example, female Mov10l1(–/–) mice have normal fertility. This manuscripts report is consistent with a recently published BioRxiv

preprint by Hasuwa et al. (2021) showing that PIWIL1- and PIWIL3-deficiency causes female sterility in golden hamster. In addition to this paradigm- shifting discovery, the manuscript reported critical functions of the mammalian piRNA pathway in pre-meiotic, meiotic, and post-meiotic phases of germline development, which validate the discoveries from mouse studies for other mammals. The Data in the manuscript are robust, straight forward, and clearly presented. The conclusions are properly drawn. The manuscript is a pleasure to read. This review supports its publication in NCB after a revisionto address the following questions to make the manuscript intellectually complete.

1. In Mov10l1(–/–) oocytes, only 57 genes were differentially expressed, transposons are moderately increase at best, and there is little or no global change in DNA methylation. Then,how does Move10L serve as a maternal factor to achieve its essential function in early embryogenesis? Ideally, the authors need to provide positive data on the effect of Mov10L during early embryogenesis. For example, maybe a few of the 57 genes are very important. Ifone reverts the level of these important mRNA candidates in the oocyte back to the wildtype level, embryogenesis might be improved or possibly back to normal. For another example, what do maternal piRNAs target in addition to transposons and protein-coding genes? Maybethis will give clues to the Mov10L function. None of these data were described and analyzed in depth at least bioinformatically in the paper. Without an explanation on how Mov10L becomes an essential maternal factor, it leaves the reader an unsatisfied feeling about what one can learn from the paper beyond the phenotypic information.

Bioinformatics analyses of differentially expressed genes in *Mov10l*$^{-/-}$ oocytes did not yield atestable hypothesis regarding how these genes or their subset could contribute to the loss of developmental competence of *Mov10l*$^{-/-}$ oocytes. Gene Ontology analysis did not yield any significantly enriched term in any of the tested categories. The list of the genes appears stochastic and either reflects stochastic nature of evolution of piRNA-mediated regulation of gene expression, or differentially expressed genes are probably an effect of targeting retrotransposons. The situation is analogous to the analysis of miRNA-mediated control of gene expression where the loss of miRNAs has pleiotropic effects and only in specific cases it is possible to identify and validate genes, which significantly contribute to the phenotype. Given the restricted length of the article and inconclusive outcome of the profiling, we limited our comments on the transcriptome remodeling in *Mov10l*$^{-/-}$

oocytes to the necessary minimum. For the revised version, we integrated our results with those of Hasuwa et al. from*Piwil1* mutants, who observed much stronger effect on the maternal transcriptome. There seems to be three factors influencing the difference between the observed number of differentially expressed genes on both mutants.

1) **Different parameters for DEGs identification.** When comparing RNA-seq data from *Piwil1*$^{-/-}$ and *Mov10l*$^{-/-}$ mutants using the same pipeline and p-adjusted cut-off for DESeq2 (we used 0.01 resulting in detection of 57 DEGs in *Mov10l*$^{-/-}$ mutants), we identified 456 DEGs in *Piwil1*$^{-/-}$ MII oocytes (originally were reported 1612). A similar trend was observed with cut-off 0.05 when we obtained 736 (*Piwil1*$^{-/-}$) vs. 126 (*Mov10l*$^{-/-}$) DEGs, respectively. For analysis of DEGs, we prefer to use higher statistical power than the default DESeq2 cut- off 0.1. Thus, data variability and filtering stringency can explain a part of the difference in DEG numbers in the two manuscripts while we think that the rest of the difference can be attributed to difference in the analyzed stages and possibly different impacts of the introduced mutation on piRNA populations.

2) **Different analyzed oocyte stages**. *Mov10l*$^{-/-}$ transcriptome was analyzed in an earlier stage (pre-ovulatory fully-grown oocyte) than the *Piwil1*$^{-/-}$ transcriptome (ovulated MII oocytes). The later underwent meiotic maturation associated with a global post- transcriptional transcriptome remodeling, which includes changes in mRNA polyadenylationand stability - 10.1016/bs.ctdb.2015.06.004. A limited aberrant gene expression in fully grown oocytes can lead to a larger transcriptome dysregulation during meiotic maturation. The two stages are thus distinct and each has its own advantages and disadvantages for phenotype analysis. The analysis of MII stage has a better chance to reveal whether the maternal transcriptome is strongly affected while the analysis of fully-grown oocytes has a better chance to identify primary defects leading to bigger changes observed at MII. In specific cases, as was, for example, our analysis of *Cnot6l* deadenylase knock-out (10.26508/lsa.201800084), fully-grown oocytes may appear essentially normal while strong transcriptome changes build up during the meiotic maturation and beyond.

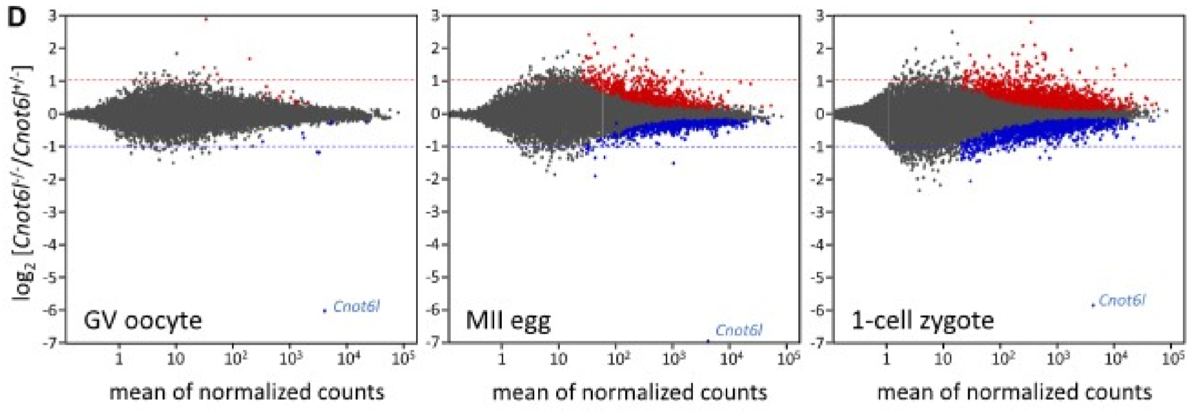

Figure 3. Transcriptome changes in *Cnot6l* knockout oocytes and zygotes.

In case of *Mov10l1* hamster mutants, we can observe that the loss of piRNAs has a small detectable impact on the transcriptome and *Piwil1* mutant implies that transcriptome is more dysregulated at the end of meiotic maturation. However, the list of DEGs in *Mov10l*$^{-/-}$ fully- grown GV oocytes does not contain apparent candidates that could explain the transcriptomeremodeling and phenotype. We lack a model how directly or indirectly piRNAs affect the expression of these particular DEGs. From the data it seems that piRNAs affect maternal mRNA expression in multiple ways, which cannot be delineated bioinformatically with the available data. On a positive side, we were able to analyze *Piwil1*$^{-/-}$ and *Mov10l*$^{-/-}$ data using the same pipeline and filtering conditions and this analysis shows that, for upregulated genes,the transcriptome changes in both mutants are appear related and that *Piwil1* mutants show higher magnitude of changes (Fig. S7E). There were five genes, which stood out as stronglyupsregulated ones in both mutants but only one of them, *Kif2a* had an apparent possible connection to the piRNA pathway as it employs an LTR oocyte-specific promoter – but thistwas a unique feature. We included it into the revised version as it illustrates that transcriptome changes in oocytes may include such unique examples.

3) **Different impact of the mutants on maternal piRNA populations.** For the revised version, we analyzed how the loss of MOV10L1 affects piRNAs in the oocyte. This analysis,summarized in the new supplementary Figure S8, shows that *Mov10l*$^{-/-}$ oocutes lost most piRNAs – this is also consistent with data from Zhang et al., which were put in Biorxiv during the review of our article:

G

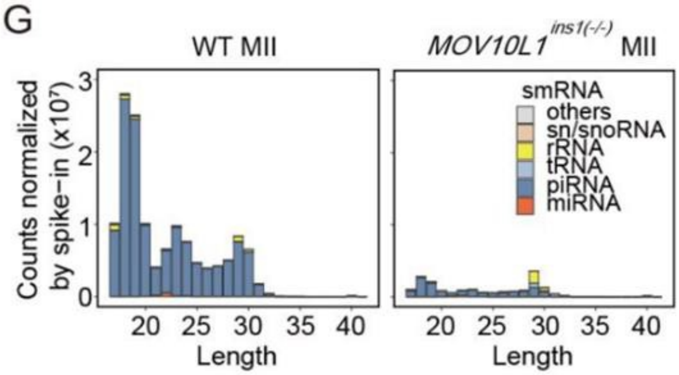

At the same time, the inspection of specific piRNA clusters implies that unique piRNAs remain produced in the oocyte despite the loss of MOV10L1 (Fig. S8D). This could contribute to observed differences between *Mov10l1* and *Piwil1* phenotypes especially in cases wherethe reported *Piwil1* phenotypes was stronger (e.g. spindle defect).

Given the scope of the article and limited space, we kept discussion of the transcriptome changes and phenotype rather short. However, we believe that the revised version providesextended transcriptome analysis, which allows to better understand the complexity of the issue and the relationship between *Mov10l1* and *Piwil1* data.

2. The 57 and >900 differentially expressed genes in the oocyte and the testis, respectively, belong to two opposing categories: up-regulated and down-regulated. How can Mov10L deficiency achieve contrasting effects of regulation towards different differentially expressedgenes? How many of these genes are direct targets of piRNAs and how many are likely due to indirect effect? These questions at least need to be addressed bioinformatically. Otherwise,a mere gene profiling exercise does not yield much useful information.

The two biological systems, oocytes end testes, apparently represent two distinct cases. The impact of the loss of MOV10L1 on oocytes was discussed above. It seems that there is a limited number of differentially expressed genes and a mild but detectable derepression of retrotransposons in ovarian oocytes. Such defects, some of which seem to accumulate (*Piwil1*MII transcriptome) are not associated with a significant impact on the transcriptionally quiescent part of the oocyte-to-zygote transition. However, when the zygotic genome activation takes place at the 2-cell stage, development fails. This is a maternal effect phenotype where it may be difficult to pinpoint the original defect in the oocyte, which underlies the 2-cell arrest, especially given limited availability of hamster's 2-cell zygotes forthe analysis.

In testes, we observed full loss of piRNAs, which results in a failure to differentiate into spermatogonia. The failure of germ cells brings robust reduction of expression of genes specific for spermatogenic cells – likely this is a secondary effect of the failure. We examined how many genes (transcripts) might be direct targets of piRNAs (Fig. S13), and it seems thatit is only a minor fraction having higher piRNAs abundance (approx. >5 RPM). At the sametime, mRNAs having >5 RPM complementary piRNAs are clearly more upregulated than downregulated, suggesting that this group is indeed enriched in direct piRNA targets – but the group is rather small.

For the revision, we also extended transcriptome analysis, which now includes day 0 testicular transcriptomes (new supplementary Figure S14). In newborn testes, we observe detectable changes in the transcriptome but they are very different from those observed at 9 dpp. Germ cell-specific expression appears intact at 0 dpp, and we observe a mild dysregulation of MYSERVx elements, which escalates during the 9 dpp failure. A notable difference is that we identified yet another retrotransposon family, which is derepressed in testes and is associated with high amount of testicular piRNAs: MMERVKx (see Figs. S14 and S3A). This implies that a part of the variability of DEGs involves effects of derepressionof different subfamilies of retrotransposons.

Given the limited space and the fact that newborn testes do not exhibit any apparent defect inspermatogenesis, we have focused on 9 dpp defects and MYSERV6 retrotransposons and discuss additional data in the context of the 9 dpp phenotype.

In addition, the authors need to take care of the following minor issues:

1. Line 158-159, In Mov10l1(–/–) oocytes, 57 genes were differentially expressed in Mov10l1(+/+) oocytes. How many of the differentially expressed 57 genes Mov1l0(-/-)oocytes are also differentially expressed in PIWIL1-/- and PIWIL3-/- oocytes?

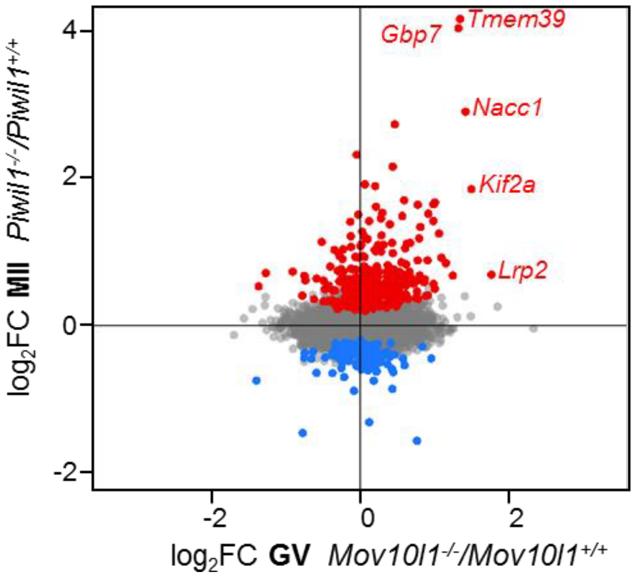

Piwil3 mutation has virtually no impact on the MII transcriptome even with the original stringency for DEGs used by Kasuwa et al., whilethere is only a subfertile phenotype. We obtained the list of 21 *Piwil3*$^{-/-}$ DEGs – and there was no overlap with *Mov10l1*$^{-/-}$ DEGs.

Regarding Piwil1, we re-analyzed original sequencing data kindly provided by Haruhiko Siomi's group. As already mentioned above, we provide a combined analysis of *Mov10l1*$^{-/-}$ and *Piwil1*$^{-/-}$ oocytes (Fig. 7E), which shows that genes upregulated in *Piwil1*$^{-/-}$ have an apparent tendency to have higher expression in *Mov10l1*$^{-/-}$(manifested as enrichment of *Piwil1*$^{-/-}$ DEGs in upper right quarter of the graph). In terms of the overlaps, when using the same pipeline and filtering conditions for DEGs, we identified 456 DEGs in *Piwil1*$^{-/-}$ MII oocytes and 13 of the upregulated DEGs are among the 45 DEGs upregulated DEGs *Mov10l1*$^{-/-}$ oocytes. There wasessentially on overlap among downregulated genes (1 gene).

2. Lines 184-5: "Histology of old males (>50 weeks) revealed aspermatogenic seminiferoustubules (Fig. 3B and S12B)". The Histology of wildtype old males (>50 weeks) is need in Fig. 3B as a control.

The wild-type testis H&E staining in Fig. 3B is from a 102 weeks-old male hamster showingthat spermatogenesis appears histologically intact until that age.

3. "Approximately 3% of adult seminiferous tubules contained small clusters of cells positivefor the germ cell marker DDX4 (VASA) and the marker of meiotic cells Synaptonemal Complex Protein 3 (SCP3) (Fig. 3C and S12C). It's important to indicate the age of the testeshere since the phenotype could be quite age-dependent.

The age of animals is indicated in figure legends.

4. Supplementary materials are not well organized e.g., Supplementary reference list (lines920-1171) was not included in the Table of contents (lines 365-369). In addition, ExtendedData Figures were not serially labelled.

For the first submission, the manuscript had a single reference list for both, the main text andthe Supplement as suggested in the instructions for authors. Extended Data Figures in the revised version are provided according to the journal's rules, we apologize for any inconvenience with the Extended Data Figures in the first submission.

Reviewer #3

Remarks to the Author:

The manuscript "Formation of hamster's spermatogonia and fertile oocytes requires piRNAs"submitted by Loubalova et al. reported that loss of MOV10L1 helicase, an essential protein inpiRNA biogenesis, generates novel phenotypes in male and female hamsters compared with mice. The authors chose golden hamster as a model to study the essential roles of piRNAs in spermatogenesis and oogenesis because of its similarity with human. Golden hamster has four distinct PIWI proteins, including PIWIL1, PIWIL2, PIWIL3 and PIWIL4. Unlike mice, mutant male hamsters have impaired establishment of spermatogonia. More strikingly, the female mutant hamsters are infertile while female mutant mice are fertile. Overall, this study has logical and nice design and the phenotypes are convincing. The significance is two-fold. First, this is the first study of the function of MOV10L1 in a non-mouse mammalian model. Second, mouse MOV10L1/PIWI mutants do not display female fertility defects. This study shows that MOV10L1 in golden hamster is important for female fertility. This finding is novel and may be relevant to human. However, the mechanisms of the sterile phenotypes for both males and females are not clear, which tempered the significance of the study.

Major concerns:

1) Small RNAs are not sequenced in MOV10L1 mutants. Small RNAs are sequenced in wildtype testes at 9, 13, and 21 dpp, but surprisingly not in MOV10L1 mutant. It is assumed that piRNAs are absent in hamster MOV10L1 mutants, because it is required for piRNA production in mouse. Direct experimental evidence is necessary

to demonstrate this. Small RNAs need to be sequenced in MOV10L1-/- oocytes and MOV10L1-/- 9 dpp testes to see if MOV10L1 is essential for piRNA production in golden hamster. Such data will strengthen this study. Without it, this opportunity is missed.

When analysing the *Mov10l1*$^{-/-}$ phenotype, we indeed sequenced small RNAs from mutant testes at 9 dpp, 13 dpp, 21 dpp, and adult. Although germ cells are mostly absent in 13 dpp and older testes, these data were nonetheless useful to define germline piRNA clusters. Sincethese data served mainly as controls, we did not highlight in the text that we analysed 9 dpp *Mov10l1*$^{-/-}$ testes except of a supplementary Figure S1 and listing knock-out libraries in TableS7 and used to define piRNA clusters at 9 dpp, so this fact could be easily overlooked.
Sequencing data from 9 dpp, when germ cells are still present, show that piRNAs are absent, i.e. MOV10L1 seems to be essential for piRNA biogenesis in the male germline.

Sequencing of small RNAs from oocytes was challenging because of the limited amount ofmaterial, and we did not succeed to produce this sequencing for the first submission.
However, we recently managed to sequence small RNAs from hamster oocytes. For the revised version, we added analysis how the loss of MOV10L1 affects piRNAs in the oocyte.These data, summarized in the new supplementary Figure S8, show that *Mov10l*$^{-/-}$ oocytes lost most piRNAs (which is also consistent with data from Zhang et al., which were put in Biorxiv during the review of our article (doi: https://doi.org/10.1101/2021.03.21.434510)):

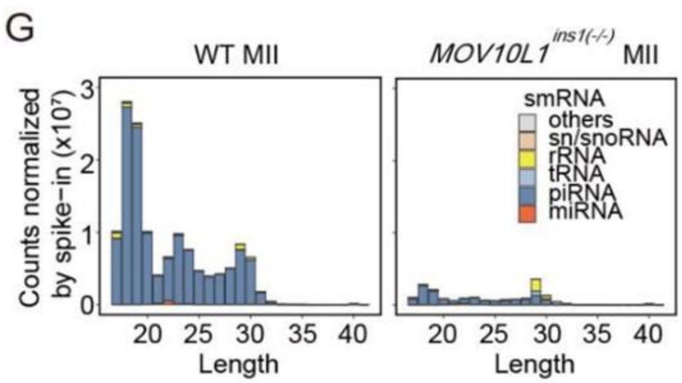

Importantly, the inspection of specificpiRNA clusters implies that unique piRNAs remain produced in the oocyte despite the loss of MOV10L1 (Fig. S8D). This could contribute to observed differences between *Mov10l1* and *Piwil1* phenotypes especially in cases where the reported*Piwil1* phenotypes was stronger (e.g. spindle defect).

Given the scope of the article and limited space, we kept discussion of the transcriptome changes and phenotype rather short. However, we believe that the revised version providesextended transcriptome analysis, which allows to better understand the complexity of the issue and the relationship between *Mov10l1* and *Piwil1* data.

2) In Abstract, the statement "Our results not only refute the notion that in mammals the piRNA pathway is important only in males" is not accurate. Line 177: "…and proves wrongthe notion that in mammals the piRNA pathway is important only in males." This notion is not widely held in the field. On the contrary, it is widely accepted that mouse oocyte has evolved the oocyte-specific dicer isoform and the siRNA pathway to suppress TEs and thus does not need the piRNA pathway. It is widely believed that mouse is an exception rather than the rule. These sentences need to be deleted or revised. Indeed, this study and a PIWI study in preprint are the first to show the importance of piRNA pathways in mammalian female fertility.

We revised the sentence in the abstract to: *Our results bring evidence that the mammalian piRNA pathway can play essential roles in both sexes and demonstrate the adaptive nature ofthe mammalian piRNA pathway, which allows to confront emerging genomic threats and acquire new critical roles in the germline.*

Regarding the statement "proves wrong the notion that in mammals the piRNA pathway isimportant only in males", we believe that it is still relevant for a broader readership, whichincludes researchers who do not follow the piRNA field in depth but are aware that mouseknockout of the piRNA pathway yields sterile males but fertile females.

3) RNA-seq of 9 dpp testes in Fig 4A. The RNA-seq results was very likely complicated bythe different cellular composition between MOV10L1-/- and WT. The KO testes likely contained fewer germ cells than wild type. This is a common problem for RNA-seq analysisusing whole testes. This caveat needs to be acknowledged.

In the revised version, we address this problem. The issue of fewer or even absent germ cellsstrongly distorts expression data at 13 dpp and later stages (but, consequently, also allows toidentify hamster's germ cell-specific genes). Notably, our data suggest that the observed transcriptome changes at 9 dpp would not be strongly biased by the number of germ cells at that timepoint because the number of

germ cells does not seem to be significantly lower at 9 dpp. We analyzed germ cells in ~130 seminiferous tubules in several histological sections and, while we observed slightly increased percentage of tubules lacking germ cells, the difference was not statistically significant – we added these data as Fig. S12.

4) Lines 147-154: The authors found that compared with MOV10L1+/+ and MOV10L1+/– profiling, only 13 genes increased significantly in MOV10L1–/– oocytes, and concluded that"even small transcriptome changes in MOV10L1–/– oocytes still could contribute to the observed developmental incompetence". However, if I understand Fig. S9C correctly, 25 genes were upregulated in MOV10L1+/– compared with MOV10L1+/+, and presumably MOV10L1+/– females are fertile. Therefore, it is not convincing to make the conclusion thatthe minor transcriptome change in the mutant causes the infertile phenotype in the mutant females.

The point we were trying to make was that even a small number of differentially expressed genes in the oocyte can cause infertility, i.e. despite the number of the changes was small, wecannot rule out that this causes sterility. However, we did not have an intent to go further in our interpretation in the text, i.e. to claim that this is indeed the cause of the infertile phenotype. This part was thoroughly revised, including a more accurate comparison with transcriptome changes in *Piwil1* mutant eggs and we leave the conclusion open.

5) The mutant oocytes were able to ovulate and mutant MII eggs can be fertilized but fail to support development after fertilization. It would be more informative to know at which stagethe embryos cease developing.

Zygotes from knock-out oocytes arrest at the 2-cell stage. We added later 61h time-point datato the manuscript, which show that Mov10l1$^{-/-}$ zygotes remain at the 2-cell stage. Notably, a 2-cell arrest was also reported for an independently produced *Mov10l1* knock-out in a pre- print by Zhang et al., which was posted on March 22, 2021, while our work was under review(10.1101/2021.03.21.434510).

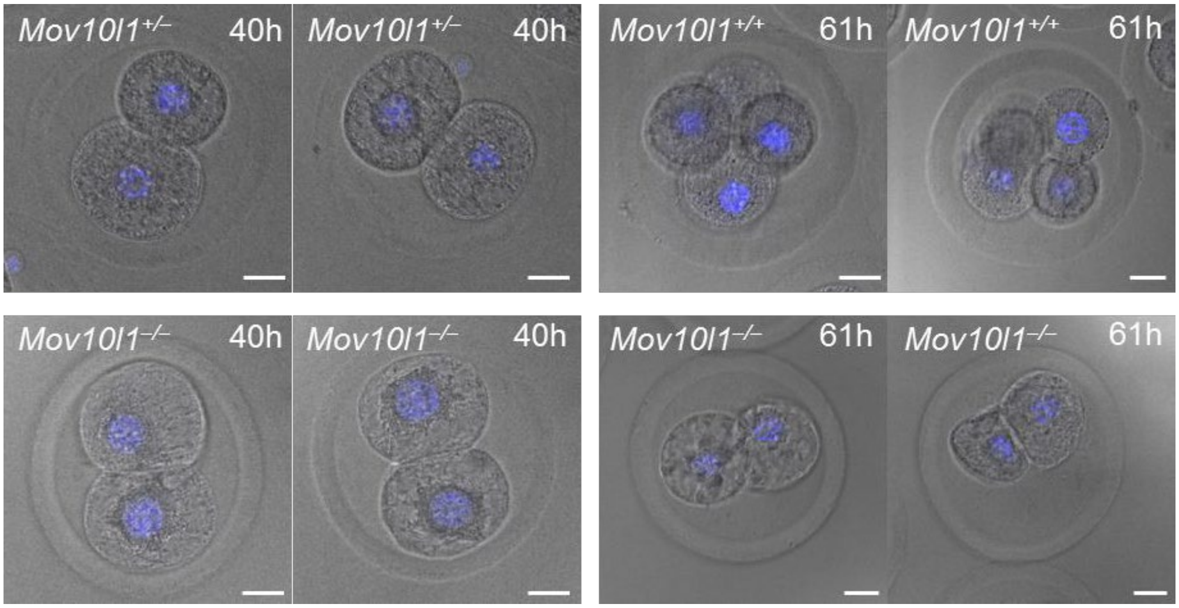

Minor concerns:

1) Some figure legends and text descriptions are too simple to understand, and it might be due to the page or word limit. Specifically, in Lines 100-101, it was said that "MYSERV and IAP-derived piRNAs were the most abundant piRNAs targeting a transposable element (Fig. 1C and S4A)", however, clearly we can see ORR1 has higher abundance than IAP in Fig. 1C.Another example is that the authors used Fig. S4D to indicate that the number of L1 elements are comparable to human genome but smaller than rat genome, however, I did not see thedirect information in Fig. S4D or its figure legend.

We apologize for imprecise statements. In the first case, we overlooked ORR1 as it is a peculiar case – it is a non-autonomous element, which requires activity of autonomous elements, and its LTR is often exapted as a promoter for genes expressed during oogenesis and spermatogenesis. We corrected the text to: *MYSERV and IAP-derived piRNAs were alsothe most abundant piRNAs targeting autonomous transposable elements.*

In the second case, the reference to Fig. S4D was originally included to point out that active mouse L1 elements come from a different L1 subfamily. When we were reducing the text, thespecific L1 families were removed while the reference was left in the sentence. The sentence was revised to: *L1 analysis revealed 110 intact L1 elements from the Lx5/6 subfamily (Data S5), which is comparable to the 146 intact L1s in the human genome but much smaller than the 2,811 in the mouse genome, which come from a different L1 subfamily (Fig. S3D).*

2) Lines 120: How were young retrotransposons defined? I believe the result is true since ithas the similar conclusion with a preprint paper published by Hasuwa et al, 2021. But moredetailed information is necessary for clarity.

“Young” retrotransposons were defined by low nucleotide substitution rate within a subfamily - close to 0 for a high number of 200 randomly selected genomic insertions. SeeFig. S3C and S3D for L1 and IAP subfamilies and Data S1-S3 for other LTR element subfamilies. We added a comment to the Figure S3 legend.

3) Lines 241-242: It would be more convincing to perform real-time PCR to confirm theupregulation of MYSERV retrotransposons in the mutant testes.

We performed real-time PCR, which confirmed strong upregulation of the MYSERV6 element (and expression changes of IAP3, IAP4, Lx6, Kif5C, and Ddx4 in 9 dpp knock-outtestes), which validates observations made by RNA-seq. These data were added to the supplementary Fig. S14.

Decision Letter, first revision:

Date:	21st June 21 01:28:23
Last Sent:	21st June 21 01:28:23
From:	jie.wang@nature.com
To:	svobodap@img.cas.cz
CC:	ncb@springernature.com
Subject:	Your manuscript, NCB-S44937A
Message:	Our ref: NCB-S44937A

21st June 2021

Dear Dr. Svoboda,

Thank you for submitting your revised manuscript "Formation of hamster’s spermatogonia and fertile oocytes requires piRNAs" (NCB-S44937A). It has now been seen by the original referees and their comments are below. The reviewers find that the paper has improved in revision, and therefore we'll be happy in principle to publish it in Nature Cell Biology, pending minor revisions to satisfy the referees' final requests and to comply with our editorial and formatting guidelines.

We are now performing detailed checks on your paper and will send you a checklist detailing our editorial and formatting requirements in about a week. Please do not upload the final materials and make any revisions until you receive this additional information from us.

Thank you again for your interest in Nature Cell Biology Please do not hesitate to contact me if you have any questions.

Sincerely,

Jie Wang, PhD
Senior Editor
Nature Cell Biology

Tel: +44 (0) 207 843 4924
email: jie.wang@nature.com

Reviewer #1 (Remarks to the Author):

The authors have addressed and/or clarified the issues that had been raised. I have no further comments and support publication.

Reviewer #2 (Remarks to the Author):

In the revision, the authors have made an earnest attempt to address this reviewer’s questions by additional bioinformatic analysis. Although the new results do not clarify much of the originally raised mechanistic puzzles, they do provide more information to readers and reflect the complex nature of biology that we need to recognize. Further analysis would be a significant investment that is much beyond the scope of this paper. I support the publication of the revised version.

One minor comment: text Lines 55-56: “Loss of MOV10L1 or PIWI proteins in mice revealed specific essential roles of piRNAs in spermatogenesis but not in oogenesis”. A key paper to cite here is by Cheng

et al (2014) PLoS One (Volume 9 | Issue 9 | e97821), which is the only report showing that knocking out all three Piwi genes in mice only causes male sterility.

Reviewer #3 (Remarks to the Author):

The revised manuscript has improved a great deal. Significant new data have been added to the revision and strengthened the manuscript. In particular, the small RNA-seq data from oocytes are very informative. I support its publication.

Decision letter, final requests:

Date:	1st July 21 03:51:10
Last Sent:	1st July 21 03:51:10
From:	ziqian.li@nature.com
To:	svobodap@img.cas.cz
CC:	ncb@springernature.com,jie.wang@nature.com
BCC:	ziqian.li@nature.com
Subject:	NCB: Your manuscript, NCB-S44937A
Message:	Our ref: NCB-S44937A

1st July 2021

Dear Dr. Svoboda,

Thank you for your patience as we've prepared the guidelines for final submission of your Nature Cell Biology manuscript, "Formation of hamster's spermatogonia and fertile oocytes requires piRNAs" (NCB-S44937A). Please carefully follow the step-by-step instructions provided in the attached file, and add a response in each row of the table to indicate the changes that you have made. Ensuring that each point is addressed will help to ensure that your revised manuscript can be swiftly handed over to our production team.

We would like to start working on your revised paper, with all of the requested files and forms, as soon as possible (preferably within two weeks). Please get in contact with us if you anticipate delays.

When you upload your final materials, please include a point-by-point response to any remaining reviewer comments.

If you have not done so already, please alert us to any related manuscripts from your group that are under consideration or in press at other journals, or are being written up for submission to other journals (see: https://www.nature.com/nature-research/editorial-policies/plagiarism#policy-on-duplicate-publication for details).

In recognition of the time and expertise our reviewers provide to Nature Cell Biology's editorial process, we would like to formally acknowledge their contribution to the external peer review of your manuscript entitled "Formation of hamster's spermatogonia and fertile oocytes requires piRNAs". For those reviewers who give their assent, we will be publishing their names alongside the published article.

Nature Cell Biology offers a Transparent Peer Review option for new original research manuscripts submitted after December 1st, 2019. As part of this initiative, we encourage our authors to support increased transparency into the peer review process by agreeing to have the reviewer comments, author rebuttal letters, and editorial decision letters published as a Supplementary item. When you submit your final files please clearly state in your cover letter whether or not you would like to participate in this initiative. Please note that failure to state your preference will result in delays in accepting your manuscript for publication.

Cover suggestions

As you prepare your final files we encourage you to consider whether you have any images or illustrations that may be appropriate for use on the cover of Nature Cell Biology.

Covers should be both aesthetically appealing and scientifically relevant, and should be supplied at the best quality available. Due to the prominence of these images, we do not generally select images featuring faces, children, text, graphs, schematic drawings, or collages on our covers.

We accept TIFF, JPEG, PNG or PSD file formats (a layered PSD file would be ideal), and the image should be at least 300ppi resolution (preferably 600-1200 ppi), in CMYK colour mode.

If your image is selected, we may also use it on the journal website as a banner image, and may need to make artistic alterations to fit our journal style.

Please submit your suggestions, clearly labeled, along with your final files. We'll be in touch if more information is needed.

Nature Cell Biology has now transitioned to a unified Rights Collection system which will allow our Author Services team to quickly and easily collect the rights and permissions required to publish your

work. Approximately 10 days after your paper is formally accepted, you will receive an email in providing you with a link to complete the grant of rights. If your paper is eligible for Open Access, our Author Services team will also be in touch regarding any additional information that may be required to arrange payment for your article.

Please note that Nature Cell Biology is a Transformative Journal (TJ). Authors may publish their research with us through the traditional subscription access route or make their paper immediately open access through payment of an article-processing charge (APC). Authors will not be required to make a final decision about access to their article until it has been accepted. Find out more about Transformative Journals

Authors may need to take specific actions to achieve compliance with funder and institutional open access mandates. For submissions from January 2021, if your research is supported by a funder that requires immediate open access (e.g. according to Plan S principles) then you should select the gold OA route, and we will direct you to the compliant route where possible. For authors selecting the subscription publication route our standard licensing terms will need to be accepted, including our self-archiving policies. Those standard licensing terms will supersede any other terms that the author or any third party may assert apply to any version of the manuscript.

Please note that you will not receive your proofs until the publishing agreement has been received through our system.

For information regarding our different publishing models please see our Transformative Journals page. If you have any questions about costs, Open Access requirements, or our legal forms, please contact ASJournals@springernature.com.

Please use the following link for uploading these materials:
[REDACTED]

If you have any further questions, please feel free to contact me.

Best regards,

Ziqian Li
Editorial Assistant
Nature Cell Biology

On behalf of

Jie Wang, PhD
Senior Editor
Nature Cell Biology

Tel: +44 (0) 207 843 4924
email: jie.wang@nature.com

Reviewer #1:
Remarks to the Author:
The authors have addressed and/or clarified the issues that had been raised. I have no further comments and support publication.

Reviewer #2:
Remarks to the Author:
In the revision, the authors have made an earnest attempt to address this reviewer's questions by additional bioinformatic analysis. Although the new results do not clarify much of the originally raised mechanistic puzzles, they do provide more information to readers and reflect the complex nature of biology that we need to recognize. Further analysis would be a significant investment that is much beyond the scope of this paper. I support the publication of the revised version.

One minor comment: text Lines 55-56: "Loss of MOV10L1 or PIWI proteins in mice revealed specific essential roles of piRNAs in spermatogenesis but not in oogenesis". A key paper to cite here is by Cheng et al (2014) PLoS One (Volume 9 | Issue 9 | e97821), which is the only report showing that knocking out all three Piwi genes in mice only causes male sterility.

Reviewer #3:
Remarks to the Author:
The revised manuscript has improved a great deal. Significant new data have been added to the revision and strengthened the manuscript. In particular, the small RNA-seq data from oocytes are very informative. I support its publication.

Final Decision Letter:

Date: 27th July 21 02:25:23

From: jie.wang@nature.com

To: svobodap@img.cas.cz

CC: ncb@springernature.com; ziqian.li@nature.com

BCC: rjsproduction@springernature.com;rjsart@springernature.com

Subject: Decision on Nature Cell Biology submission NCB-S44937B

Message: Dear Dr Svoboda,

I am pleased to inform you that your manuscript, "Formation of spermatogonia and fertile oocytes in hamsters requires piRNAs", has now been accepted for publication in Nature Cell Biology.

Thank you for sending us the final manuscript files to be processed for print and online production, and for returning the manuscript checklists and other forms. Your manuscript will now be passed to our production team who will be in contact with you if there are any questions with the production quality of supplied figures and text.

In approximately 10 business days you will receive an email with a link to choose the appropriate publishing options for your paper and our Author Services team will be in touch regarding any additional information that may be required.

You will not receive your proofs until the publishing agreement has been received through our system.

If you have any questions about our publishing options, costs, Open Access requirements, or our legal forms, please contact ASJournals@springernature.com

Before the manuscript is sent to our printers, we will make changes in the text that may be necessary either to make it conform with house style or to make it intelligible to our wide readership. We look particularly carefully at the titles of all papers to ensure that indexing will be accurate and that they are not unreasonably long. We will ask your approval before the copy is finalized, and you will soon receive the edited proofs. Please check the text and figures carefully. Once your manuscript is typeset and you have completed the appropriate grant of rights, you will receive a link to your electronic proof via email with a request to make any corrections within 48 hours. If, when you receive your proof, you cannot meet this deadline, please inform us at rjsproduction@springernature.com immediately.

Once your paper has been scheduled for online publication, the Nature press office will be in touch to confirm the details. An online order form for reprints of your paper is available at https://www.nature.com/reprints/author-reprints.html. All co-authors, authors' institutions and authors' funding agencies can order reprints using the form appropriate to their geographical region.

Publication is conditional on the manuscript not being published elsewhere and on there being no announcement of this work to any media outlet until the online publication date in Nature Cell Biology.

Please note that *Nature Cell Biology* is a Transformative Journal (TJ). Authors may publish their research with us through the traditional subscription access route or make their paper immediately open access through payment of an article-processing charge (APC). Authors will not be required to make a final decision about access to their article until it has been accepted. Find out more about Transformative Journals

Authors may need to take specific actions to achieve compliance with funder and institutional open access mandates. For submissions from January 2021, if your research is supported by a funder that requires immediate open access (e.g. according to Plan S principles) then you should select the gold OA route, and we will direct you to the compliant route where possible. For authors selecting the subscription publication route our standard licensing terms will need to be accepted, including our self-archiving policies. Those standard licensing terms will supersede any other terms that the author or any third party may assert apply to any version of the manuscript.

To assist our authors in disseminating their research to the broader community, our SharedIt initiative provides you with a unique shareable link that will allow anyone (with or without a subscription) to read the published article. Recipients of the link with a subscription will also be able to download and print the PDF.

If your paper includes color figures, please be aware that in order to help cover some of the additional cost of four-color reproduction, Nature Research charges our authors a fee for the printing of their color figures. Please contact our offices for exact pricing and details.

As soon as your article is published, you will receive an automated email with your shareable link.

If you have not already done so, we strongly recommend that you upload the step-by-step protocols used in this manuscript to the Protocol Exchange (www.nature.com/protocolexchange), an open online resource established by Nature Protocols that allows researchers to share their detailed experimental know-how. All uploaded protocols are made freely available, assigned DOIs for ease of citation and are fully searchable through nature.com. Protocols and the Nature and Nature research journal papers in which they are used can be linked to one another, and this link is clearly

and prominently visible in the online versions of both papers. Authors who performed the specific experiments can act as primary authors for the Protocol as they will be best placed to share the methodology details, but the Corresponding Author of the present research paper should be included as one of the authors. By uploading your Protocols to Protocol Exchange, you are enabling researchers to more readily reproduce or adapt the methodology you use, as well as increasing the visibility of your protocols and papers. You can also establish a dedicated page to collect your lab Protocols. Further information can be found at www.nature.com/protocolexchange/about

You can use a single sign-on for all your accounts, view the status of all your manuscript submissions and reviews, access usage statistics for your published articles and download a record of your refereeing activity for the Nature journals.

Please feel free to contact us if you have any questions.

With kind regards,

Jie Wang, PhD
Senior Editor
Nature Cell Biology

Tel: +44 (0) 207 843 4924
email: jie.wang@nature.com

Click here if you would like to recommend Nature Cell Biology to your librarian
http://www.nature.com/subscriptions/recommend.html#forms

** Visit the Springer Nature Editorial and Publishing website at www.springernature.com/editorial-and-publishing-jobs for more information about our career opportunities. If you have any questions please click here.**
